# CD44 alternative splicing senses intragenic DNA methylation in tumors via direct and indirect mechanisms

**DOI:** 10.1093/nar/gkab437

**Published:** 2021-06-04

**Authors:** Eric Batsché, Jia Yi, Oriane Mauger, Etienne Kornobis, Benjamin Hopkins, Charlotte Hanmer-Lloyd, Christian Muchardt

**Affiliations:** Epigenetics and RNA metabolism in human diseases. CNRS UMR8256 - Biological Adaptation and Ageing. Institut de Biologie Paris-Seine. Sciences Sorbonne Université. 7–9 Quai Saint Bernard, 75005 Paris, France; Unité de Régulation Epigénétique, Institut Pasteur, Paris, France; UMR3738, CNRS, Paris, France; Unité de Régulation Epigénétique, Institut Pasteur, Paris, France; UMR3738, CNRS, Paris, France; Ecole Doctorale Complexite du Vivant (ED515), Sorbonne Université, Paris, France; Unité de Régulation Epigénétique, Institut Pasteur, Paris, France; UMR3738, CNRS, Paris, France; Ecole Doctorale Complexite du Vivant (ED515), Sorbonne Université, Paris, France; Unité de Régulation Epigénétique, Institut Pasteur, Paris, France; UMR3738, CNRS, Paris, France; Unité de Régulation Epigénétique, Institut Pasteur, Paris, France; UMR3738, CNRS, Paris, France; Keele University, Keele, Staffordshire ST5 5BG UK; Unité de Régulation Epigénétique, Institut Pasteur, Paris, France; UMR3738, CNRS, Paris, France; Keele University, Keele, Staffordshire ST5 5BG UK; Epigenetics and RNA metabolism in human diseases. CNRS UMR8256 - Biological Adaptation and Ageing. Institut de Biologie Paris-Seine. Sciences Sorbonne Université. 7–9 Quai Saint Bernard, 75005 Paris, France; Unité de Régulation Epigénétique, Institut Pasteur, Paris, France; UMR3738, CNRS, Paris, France

## Abstract

DNA methylation (meDNA) is a modulator of alternative splicing, and splicing perturbations are involved in tumorigenesis nearly as frequently as DNA mutations. However, the impact of meDNA on tumorigenesis via splicing-mediated mechanisms has not been thoroughly explored. Here, we found that HCT116 colon carcinoma cells inactivated for the DNA methylases DNMT1/3b undergo a partial epithelial to mesenchymal transition associated with increased CD44 variant exon skipping. These skipping events are directly mediated by the loss of intragenic meDNA and the chromatin factors MBD1/2/3 and HP1γ and are also linked to phosphorylation changes in elongating RNA polymerase II. The role of meDNA in alternative splicing was confirmed by using the dCas9/DNMT3b tool. We further tested whether the meDNA level could have predictive value in the MCF10A model for breast cancer progression and in patients with acute lymphoblastic leukemia (B ALL). We found that a small number of differentially spliced genes, mostly involved in splicing and signal transduction, are correlated with the local modulation of meDNA. Our observations suggest that, although DNA methylation has multiple avenues to affect alternative splicing, its indirect effect may also be mediated through alternative splicing isoforms of these meDNA sensors.

## INTRODUCTION

DNA methylation involves the addition of a methyl group at position 5 of cytosines (5mC) by a small family of DNA cytosine-5 methyltransferase enzymes (DNMTs), which transfer methyl groups from the co-factor *S*-adenosyl-l-methionine to DNA (1). This heritable epigenetic modification, which is crucial for mammalian development and cell differentiation, occurs predominantly at CpG dinucleotide sequences in mammals (2–4).

DNA methylation (meDNA) patterns are established during development by DNMT3a/b and maintained in differentiated cells mainly by DNMT1, which ensures CpG-specific propagation in the newly synthesized strand by recognizing hemimethylated DNA (5,6). The 5mC levels at individual CpG result from an equilibrium between methylation and demethylation that can occur either passively or actively through hydroxymethylases (TET1/2/3). MeDNA has mostly been described for its role in the inhibition of transcriptional initiation, particularly in the context of imprinting, X inactivation, retrotransposon silencing and at promoters containing CpG-rich regions. The majority of CpGs in the human genome are methylated, with the exception of those located in active promoters, enhancers and insulators. Within the body of genes, meDNA-enrichment prevents spurious RNA-polymerase II (RNAPII) entry, and cryptic transcriptional initiation (7–9), while also regulating the activity of intragenic enhancers (10,11).

In eukaryotes, pre-messenger RNAs (pre-mRNAs) undergo splicing, a process of maturation by which large intervening sequences (introns) are removed, leaving a mature transcript composed only of exons spliced together. This process serves as a crucial regulatory step of gene expression and transcriptome diversification, as almost all genes produce alternative transcripts with differing exon compositions (12,13). Exon-exon junction assembly results from the coordinated actions of the spliceosomal complexes and the highly regulated recruitment of hundreds of splicing factors. Splicing occurs while the genes are transcribed by the RNAPII. Cotranscriptional splicing favors coupling between the RNA maturation and the chromatin template, which can impose variations in the RNAPII elongation kinetics and differential splicing factor recruitment through interaction with chromatin readers. These mechanisms can both influence the recognition of splice sites and modulate alternative splicing decisions (14–16).

Several reports have demonstrated that specific modifications of the histone tails play roles in alternative splicing (17). In parallel, gene body-enriched meDNA is positively associated with active transcriptional elongation (3). There is also a positive correlation between the level of 5mC and exon inclusion in mRNA. Conversely, introns, pseudoexons and intronless genes exhibit weaker levels of 5mC (18–21). These findings have suggested that meDNA plays a role in exon recognition by the spliceosome.

Furthermore, there is a complex crosstalk between meDNA and the histone code. For instance, the trimethylation of histone H3 on lysine 36 (H3K36me3) brought by the elongative RNAPII is enriched at exons along with meDNA (22,20). This H3K36me3 was shown to be bound by DNMT3b in gene bodies (9,23). Currently, the impact of H3K36me3 on alternative splicing is relatively well documented, with evidence for influence on the recruitment of splicing factors (24,25) and a dependency on splicing activity (26,27).

DNA methylation is also linked to H3K9me3 through a complex combination of interactions between the lysine-methyltransferases (KMTs) EHMT2 and SUV39H1, and the HP1 proteins (28–30) that are involved in the modulation of the RNAPII elongation speed and alternative splicing (31–33). However, meDNA and H3K9me3/HP1 are not always correlated, particularly in gene bodies with low CpG density (7,34). Thus, it is still unclear how HP1 contributes to the influence of meDNA on the alternative splicing regulation.

The direct influence of meDNA on alternative splicing decisions has mainly been investigated either by studying *in vitro* methylated minigene reporters integrated into chromatin (33), or by targeting TET DNA hydroxymethylases to highly methylated CpG-rich exons (35). These approaches were designed to follow the output of splicing without modifying the cellular context, and strongly suggest that meDNA affects splicing decisions. Other observations have suggested a reciprocal effect of splicing on meDNA by the recruitment of hydroxymethylases via splicing factors (36). Such a mechanism will, however, require further investigation, as a study on integrated reporter genes concluded that meDNA remains unmodified when splicing is changed (37). The mechanisms behind the impact of meDNA on splicing largely rely on methyl binding proteins including CTCF, MeCP2 and CTCFL (21,36,38–40), that may assist the recruitment of splicing factors to pre-mRNA while it is transcribed. Methyl-binding-domain (MBD1 to 4) family members, frequently mutated in cancers, have, to our knowledge, never been associated with alternative splicing (41,42).

Pervasive changes in meDNA patterns are one characteristic of human malignant tumors (43). These changes include global hypomethylation in tumor cell genomes and focal hypermethylation of numerous CpG islands (34,44). Differential CpG methylation also occurs within the body of genes, although the impact of these methylation changes has not yet been clearly characterized. The link between meDNA and splicing raises the interesting possibility that modified meDNA may affect cancer progression not only by interfering with the activity of promoters, but also by generating a bias in the outcome of alternative splicing.

Aberrant splicing is frequently observed in human tumors and is usually explained by modified splicing factor expression (45,46). For example, PRPF6, a component of the tri-snRNP complex, is overexpressed in a subset of primary and metastatic colon cancers, and its depletion by RNAi in cell lines reduces cell growth and decreases the production of the oncogenic ZAK kinase splice variant (47). Other examples include the roles of SRSF6 and SRSF10 in colon cancers and that of SRSF1 in breast cancer (48–50). Changes in alternative splicing during epithelial to mesenchymal transition (EMT) have been particularly well studied (51). EMT is a developmental program underlying the acquisition of mesenchymal properties by epithelial cells. This process, also linked to meDNA variations (52,53), is fundamental during embryogenesis, when the regulated migration of a restricted cell population is required for organogenesis. However, it is also reactivated by cancer cells to invade adjacent tissues and to disseminate towards distant organs, representing essential steps during the progression of epithelial cancers to more aggressive stages. Differentially spliced genes during EMT programs are associated with migration and invasion (FGFR2, RON and CD44), polarity and cytoskeleton organization (NUMB, RAC and p120) and transcription regulation (TCF4/TCF7L2) (51). In the case of CD44, normal EMT is associated with a switch from long epithelial isoforms (CD44v) to a shorter CD44s and is considered to have a causative impact on EMT. This switch results from the skipping of a series of alternative exons encoding regulatory regions involved in interactions between this cell surface glycoprotein and membrane receptors or components of the extracellular matrix. This is frequently correlated with decreased expression of the splicing factor ESRP1 associated with EMT (54), but several other splicing factors are known to have an impact on CD44 exon inclusion, such as SAM68 responding to external cues (55,56).

Here, we have explored the possibility of an impact of meDNA on alternative splicing that favors tumor progression. We show that the inactivation of DNMT1 and DNMT3b in HCT116 cells activates multiple markers of EMT, including production of the CD44 short isoform skipping the variant exons. EMT was associated with the modified expression and splicing of several regulators of CD44 splicing, providing a possible explanation for the modified splicing of CD44. However, we also noted changes in the chromatin structure within the body of the CD44 gene, including a decreased accumulation of MBD1 protein accompanied by a large decrease in the recruitment of HP1γ, which suggests a direct link between meDNA and CD44 alternative splicing. The decrease in variant exon inclusion in these cells was also linked to a decrease in the phophorylation of Serine5 in the RNAPII CTD. The direct effect of the DNA methylation on these alternative splicing decisions has been shown by using the dCas9/DNMT3b catalytic domain with guide RNAs targeting the variable exons. This was also confirmed in HeLa cells where the short-term depletion of DNMT1 was sufficient to cause reduced levels of DNA methylation inside the CD44 gene body and reduced usage of CD44 variant exons, without affecting splicing factors regulating CD44. Likewise, the CD44 variant exons were skipped upon the depletion of MBD1, MBD2, and MBD3. Furthermore, we showed that the MBD1/2 splicing effect was dependent on the DNA methylation while the HP1γ splicing effect was not.

The examination of a model for breast cancer tumor progression and of a cohort of patients with acute lymphoblastic leukemia (B ALL) further substantiated the correlation between the level of intragenic CD44 methylation and that of CD44 variant intron inclusion. Several genes encoding RNA binding proteins, extracellular matrix (ECM)-binding proteins, or proteins involved in signaling, were also found to be differentially spliced according to local variations in DNA methylation, as evidenced by different cellular models.

In summary, our observations suggest that meDNA data may prove informative when evaluating the likelihood of producing splice variants from genes involved in tumor progression, which can subsequently play a role as direct sensors of the meDNA.

## MATERIALS AND METHODS

### Cell culture, siRNA transfections and PMA treatment

Colorectal carcinoma cell line HCT116 (CCL-247) and double knock-out (DKO) cells for DNMT1 and DNMT3b have been purchased from Horizon Discovery. HeLa cells (CCL-2) were from ATCC. MCF10A, MCF10A(ras), DCIS and CA1A were a kind gift from Annick Harel-Bellan (INSERM, France). Cells were maintained in Dulbecco's modified Eagle's medium (Gibco) supplemented with 7% (v/v) fetal bovine serum (Thermo Scientific) and 100 U/ml penicillin-streptomycin (Gibco). MCF10A were grown in the same medium supplemented with 0.5 μg/ml hydroxycortisone, 10 μg/ml Insulin and 20 ng/ml EGF. HeLa cells were transfected with a mix containing siRNA (20 nM) and RNAi Max reagent (Life Technologies) according to the reverse transfection protocol then collected 72 h after for analysis. For experiments of DNMTs depletion, the cells were transfected twice, the second round of transfection was performed 48 h after the first one, and the cells were collected after 72h. siRNAs and gRNAs were synthesized by Qiagen or SIGMA compagnies, and their sequences are in Supplementary Table S6. PMA was purchased from SIGMA and resuspended in DMSO at 1 mM. Cells were treated with 10 nM of PMA.

### RNA extraction, reverse transcription and PCR

Cytoplasmic enriched total RNA was extracted by homogenizing the cells in TMS buffer (10 mM Tris pH 8, 250 mM NaCl, 1 mM MgCl_2_, 1% NP40, 20 mM DTT, 1 U/μl RNasin) for 15 min on ice. After quickly spinning out the nuclei and debris, supernatant was supplemented with 0.5% SDS and 2 mM EDTA before extraction by phenol–chloroform, and isopropanol–precipitation. RNase-free TURBO DNase (Ambion; Sigma)-treated RNAs were checked on agarose gel for their quality. Reverse transcription was performed using M-MLV and affinityScript (Agilent) reverse transcriptases with oligo dT and random hexamers. Equivalent to 0.2 μl was used for polymerase chain reaction (PCR) assays. PCR products of semi-quantitative PCR were resolved on agarose gel and verified by DNA sequencing. Quantitative real-time PCR (qPCR) was assayed in 10 μl reactions with Brillant III Ultra Fast SYBR-Green Mix (Agilent) using a Stratagene MX3005p system. Most of the used qPCR were a fast run with two steps : 12 s at 62°C and 10 s at 95°C. To quantify larger variant isoforms containing exons C5 to v6, C5 to v7, v6 to C17, v7 to C17 and v8 to C17 (∼500/700 bp) (Figures 5C and 6C) the long run qPCR protocol have three steps: 30 s at 62°C, 30 s at 72°C and 15 s at 95°C. Final PCR products were analysed with dissociation curves and agarose gel. Longer amplicons (Supplementary Figure S5E) were verified by sequencing and the majority of the products (>90%) correspond to the indicated isoforms in the figures. The analysis of real-time PCR was performed using the MxPro software. The sequences of primers used for PCR are in Supplementary Table S6.

### Affymetrix exon arrays

Transfected HeLa cells with siRNA targeting DNMT1 or GAPDH in independent biological triplicate were extracted. RNAs were hybridized on GeneChip Human Exon 1.0 ST Arrays (Affymetrix), and scanned following the manufacturer's instructions. Analysis was performed by GenoSplice (http://www.genosplice.com). Only genes expressed in the cells for at least in one of the two compared conditions and giving a signal of good quality probes were considered for further analysis. Significant variations (*P* < 0.05) in exon variation >20% were taken into account. Suspected events were counted manually in Arrays data visualized on the EASANA^®^ visualization interface. GEO accession GSE135277.

### Guide RNA cloning, lentiviruses mediated stable transformation of DKO cells

Guide RNAs were cloned into the lentiviral transfer vector pSB700 (Addgene 64046) (57) by following the One-step gRNA cloning protocol shared by ‘SF’ from google groups. Briefly, in a final volume of 20 μl of 1× digestion buffer, 100 ng of pSB700, 0.5 μM of annealed oligonucleotides, 1 μl Bbs I (NEB), and 1 μl T4 ligase (NEB) were incubated at 37°C for 2 h. 1 μl of this reaction mix was used to transform competent DH5α. These gRNA transfer vectors, as well as the expression vectors for dCas9-DNMT3b and dCas9-DNMT3b(E697A) (Addgene #71217, #71219) (58) were used to produce lentiviruses by transfecting 10 cm dishes of 293T cells with plasmids mixture comprising 3 μg gag/pol vector (p8.7, Addgene #14887), 0.6 μg pCMV-VSV-G vector (Addgene #8454), 6 μg transfer vector and 42 μl of FuGENE (Promega). After 3 days, medium from three plates were combined, filtrated onto 0.45 μm PVDF membrane (Millipore,SCHVU05RE) and concentrated by ultra-centrifugation. DKO-HCT116 cells were firstly transduced with dCas9-DNMT3b and dCas9-DNMT3b(E697A) lentiviruses followed by Blasticidin selection (40 μg/ml) for 7 days. Expression of dCas9-DNMT3b proteins were checked by western-blot (anti-Cas9). Blasticidin selected cells were then transduced by gRNA viral particles and enriched through CFP based cell sorting (pSB700 gRNA expression) and maintained with blasticidin selection.

### Antibodies

Antibodies were purchased from Diagenode for 5mC antibody (3D33), MBD1 N-terminal (#078-050), MeCP2 (#052-050), H3K27ac (C15410174); from Active motif for DNMT1 (#39-906), MBD2 (#39-548); from Abcam for RNAPII pS2 (ab5095), RNAPII pS5 (ab5131), histone H3 (Ab1791), H3K9me3 (ab8898); from Euromedex for HP1a (2HP-2G9-AS), BRG1 (2SN-2E12-AS); from Millipore for HP1γ (42s2, 05-690), H3K9me2 (07–441); from Santa-Cruz for Sam68 (7-1, sc-1238), ASF/SF2 (sc-33652), U2AF65 (MC3, sc-53942), RNAPII (N20, sc-899), N-cadherin (CDH2, sc-7939); from Cell Signaling for SUV39H1 (D11B6, #8729); from SIGMA for EHMT2 (HPA050550); from Novusbio for N-terminal-Cas9/dCas9 (79A-3A3); from ThermoFisher for ESRP1 (PA5-25833), E-cadherin (CDH1, PA5-32178), Vimentin (PA5-27231), CD44v5 (VFF-8, MA5-16967); and from eBioscience for CD44v7-v8 (VFF-17, BMS118). Hermes3 ascite directed against CD44 constant exons was a kind gift from Larry Sherman and Peter Herrlich (University of Karlsruhe, Germany).

### Protein extraction, cell-fractionation, western blot and immunofluorescence

Protein extract was prepared by boiling cells for 10 min in protein lysis buffer (50 mM Tris pH 8, 150 mM NaCl, 0.5 mM EDTA, 1% SDS, 1 mM DTT and protease cocktail inhibitors – Roche). Proteins were quantified by Bradford method. 5–20 μg were separated by electrophoresis on 4–12% gradient PAGE gels and transferred on nitrocellulose membrane for western blot. Cell fractionation of proteins resolved on western blot and immunofluorescence were performed as previously described (59). Briefly, 60 cm^2^ plate of HeLa cells was resuspended in 600 μl of F1 buffer (10 mM Tris pH 7.9, 10 mM KCL, 1.5 mM MgCl_2_, 0.34 M sucrose, 10% glycerol) complemented with 0.2% triton X-100 and incubated 5 min on ice. The supernatant F1 was harvested after centrifugation 1000 g, 1 min, 4°C. Packed nuclei washed once with B1 buffer before incubation in 600 μl of F2 Buffer (3 mM EDTA/0.2 mM EGTA) 30 min on ice. The F2 supernatant was collected after centrifugation at 1400 g, 1 min, 4°C. The pellet was resuspended in 600 μl of F3 buffer (25 mM Tris pH 7.4, 450 mM KCl, 2.5% glycerol, 0.3% NP-40) complemented with 12 μl of Turbo DNase (Applied, #AM1907) and incubated 15 min at 37°C with gentle shaking. This extract was sonicated 2 min with a Bioruptor (Diagenode; 10 s ON, 15 s OFF, high intensity) and incubated again 15 min at 37°C before centrifugation at 12 000 g, 10 min, 4°C to collect the supernatant containing the ‘active’ chromatin. The pellet containing the ‘inactive’ chromatin is solubilized by boiling in 600 μl of protein lysis buffer. All buffers were complemented with 1 mM DTT, 10 μM PMSF and protease inhibitor cocktail (Roche). Immunofluorescence were performed as previously described (59). Cells grown on coverslips were fixed 10 min with 4% PFA in PBS buffer, permeabilized 5 min with triton X-100 0.2% (v/v) in PBS, saturated 30 min with 2% horse serum, 0.1% Tween20 in PBS, and incubated overnight at 4°C with primary antibodies. After 3 washes of 10 min in PBS 0.1% Tween20, revelation is performed with Alexa Fluor 488-conjugated goat anti mouse or rabbit IgG antibodies (Life Technologies, #A-11029 or #A-11034, 1:1000 dilution) incubated for 1 h and washed. The coverslips were mounted onto slides using antifade reagent (Life Technologies, #S36939) and immunofluorescence images were acquired on a Carl Zeiss Axio Observer Z1 microscope equipped with ApoTome module.

### Chromatin (ChIP) and methylated DNA immunoprecipitation (MeDIP)

ChIP assays were performed as previously described (32,59). For MeDIP assay, nuclei from RNA extracted cells in TMS buffer were resuspended in 300 μl Tris pH 8 10 mM, EDTA 2 mM, SDS 0.5% and proteinase K (0.8 mg/ml), incubated 5h at 55°C before purification by phenol (pH7)/chloroform and precipitation by isopropanol and NaAcetate at room temperature for 10 min. Genomic DNA was recovered by low centrifuge speed of 4700 rpm for 10 min, treated by RNase A (20 μg/ml) for 1h at 37°C in TE buffer and sonicated for 8 min with BioRuptor (Diagenode) (15 s ON/15 s OFF) at low intensity and at 4°C. Sheared DNA was checked on agarose gel electrophoresis to be ∼500 bp. DNA was boiled for 10 min, then chilled 10 min on ice, and diluted in IP buffer (10 mM Na-Phosphate (pH 7.0), 140 mM NaCl and 0.05% Triton X-100). One microgram of DNA was incubated for 4 h with 1 μg of 5mC antibody 3D33 or non-immune IgG, followed by a 2 h incubation with 40 μl of anti-Mouse-magnetic beads (Dynabeads). Beads were washed three times for 5 min with 1 ml of IP buffer, and two times in TE NP40 0.01%. Beads were eluted by boiling for 10 min in 100 μl H2O containing 10% (v/w) chelex resin (BioRad), PK-digested for 30 min and then finally incubated 10 min at 95°C. Equivalent to 0.5 μl was used for qPCR assays.

### Bioinformatic analysis

6 RNA-seq libraries from wild type and DKO (double knockouts *DNMT1 DNMT3b)* HCT116 cells were obtained from four different studies (10,21,60, and a publically available dataset referenced in GEO *GSE45332*) (listed in Supplementary Figure S1C). RNA-seq libraries from acute lymphoblastic leukemia (ALL, *n* = 19) and pre-B healthy controls (*n* = 8) were as well obtained from the SRA database (61). Reads quality and statistics were assessed using FASTQC 0.10.1 (https://www.bioinformatics.babraham.ac.uk/projects/fastqc/), MultiQC 1.0 (62) and in house python script. After a first alignment using STAR 2.5.0a (63), principal component analysis of gene expression based on the 500 most variable genes evaluated by DESeq2 with Rlog normalization indicated two separable clusters for ALL samples (Supplementary Figure S4A). The cluster of seven ALL RNA-seq (SRR2031977, SRR2032039, SRR2032043, SRR2032105, SRR2032108, SRR2032111, SRR2032112) contained low quality libraries with a percentage of uniquely mapped reads <60% and a percentage of reads assigned to an annotation <10% (Supplementary Figure S4B). Consequently, these data were not included in our further analyses.

Genome index was produced with STAR using Ensembl GRC37 release 75 primary genome assembly and annotations. According to STAR manual and for more sensitive novel junction discovery, the junctions detected in a first round of mapping were used in a second mapping round. To discard reads potentially originating from pseudogenes, reads were mapped with only one mismatch allowed and multi-mapper reads were not counted.

When not clearly stated in the original study, read strandedness was inferred using infer_experiment.py from the RseQC 2.6.4 package (64). ALL dataset was further considered as un-stranded. Read counts were then computed with featureCounts 1.5.2 (65) at the gene meta-feature level. The obtained counts were further investigated for evidences of gene differential regulation with DeSeq2 1.14.1 (66). Principal component analysis is based on logCPM counts corrected with TMM method and obtained from EdgeR 3.16.5 (67). Analysis for HCT116 and DKO cells was conducted as explained (Supplementary Figure S1D). Alternative splicing analysis was conducted using Majiq 1.0.4 (68) using the annotation provided with the software and the mappings obtained with STAR separately for ALL, polyA DKO and totalRNA DKO datasets and with polyA and totalRNA DKO datasets grouped together (Supplementary Figure S1F).

Thirty-eight MIRA-seq alignments from the Almamun *et al.* study were downloaded from SRA (61). Broad DNA methylation peaks were detected using MACS 2.1.1 (69). Differentially methylated regions of interests (ROI, i.e. peaks detected by macs2) between ALL and healthy samples were identified using R bioconductor MEDIPS 1.24 package (70) using the EdgeR method which calculates scale factors using the TMM method. Bigwigs were generated from Almamun et al. (2015) alignments using deeptools (71). Raw coverage from the MIRA-seq alignments were described graphically with in-house python scripts.

The categories of RNA events and differential methylated regions of DKO cells and ALL shown in Figure 8 were performed manually by using IGV (Integrative Genomics Viewer) of ALL MIRA-seq and DKO BSS and by using the visualization tool VOILA for differential RNA events detected by Majiq.

## RESULTS

### Inactivation of DNMT1 and DNMT3b in HCT116 cells affects epithelial differentiation

As a first approach to meDNA-guided alternative splicing and its possible impact on cancer, we examined HCT116 human colorectal cancer cells with the DNA methylases DNMT1 and DNMT3b inactivated. These extensively studied cells, known as DKO cells, are essentially depleted of all genomic meDNA (Supplementary Figure S1A and S1B, and (72)).

**Figure 1.**
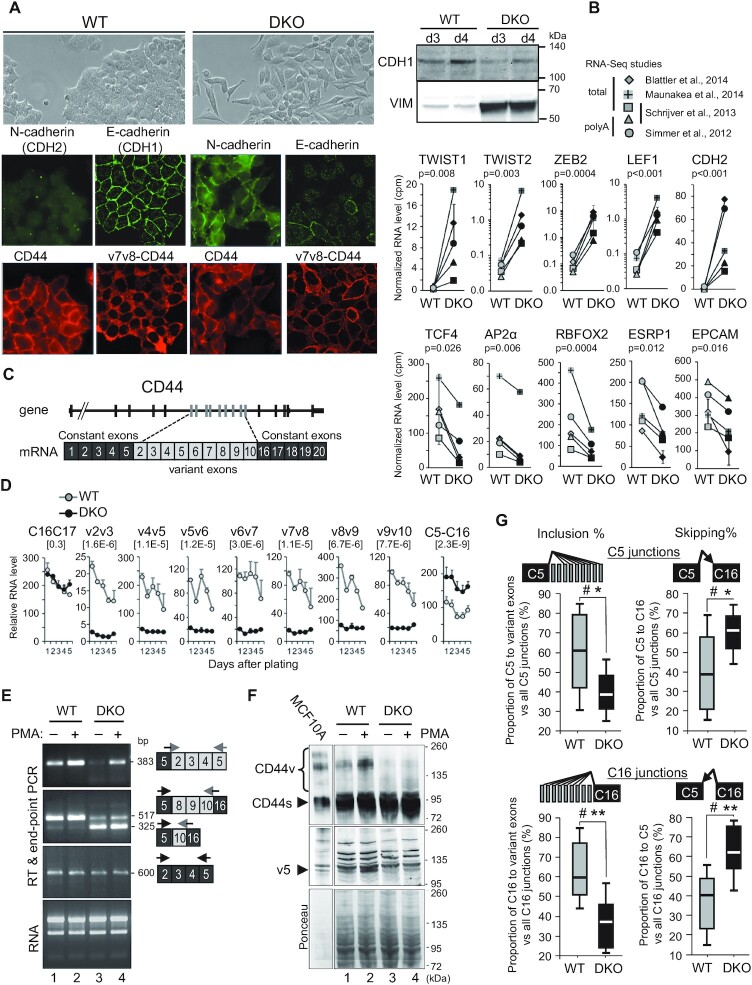
Loss of DNA methylation leads to epithelial-to-mesenchymal Transition (EMT) and a decrease in variant CD44 protein isoforms. (**A**) Left panels: immunofluorescence analysis of WT or DKO HCT116 cells with mouse antibodies (red panels) against CD44, either with Hermes3 antibody (CD44) recognizing an epitope in the C5 exon (126) or with an antibody against v7v8 variant exons (v7v8-CD44), and analysis with rabbit antibodies (green panels) against E (CDH1) or N-(CDH2) cadherins. Right panels: western blot analysis of E-cadherin (CDH1) and Vimentin (VIM) expression levels in whole cell extracts from WT or DKO HCT116 cells, 3 and 4 days after plating as in Figure 2C which shows identical protein loading between samples. (**B**) RNA-seq from 4 studies were combined (*n* = 5 samples) and matched for studies and types of extracts. The average of duplicates from the Blatter's study has been considered as one sample. Error bars show the mean absolute deviation (dev.) (see Supplementary Figure S1C). The relative levels were cpm normalized for each librairy sizes. Statistical analysis has been carried out on Rlog (DESeq2) normalized counts using a paired *t*-test (two-tailed) to compare WT to DKO levels. Top and bottom panels show mesenchymal-specific and epithelial-specific genes, respectively. (**C**) Map of the human CD44 gene and mRNA. (**D**) Relative mRNA levels of CD44 variant exons in WT and DKO HCT116 cells at each indicated day after plating, analysed by RT-qPCR using primers pairs spanning at least two different exons. Relative quantities were normalized between WT and DKO for each individual day using genes with unchanged levels (RPLP0, CDK9, CCNT1 or SAM68) as a reference. Data are the averages (± dev.) of two individual experiments. Statistical significance of the differences between DKO and WT was assessed by a paired Student's *t* test (two-tailed) using the five matched days (*n* = 10). The *P*-values are indicated in brackets for each exon pairs. (**E**) RT-PCR of total RNA from WT or DKO HCT116 cells treated (+) or not (–) with PMA for 6 h. The primers and sequenced products of each PCR assay are depicted on the right with their size (bp). RT-PCR performed on constitutive exons only (black boxes) are shown as a control. (**F**) Whole protein extracts from WT or DKO HCT116 cells treated (+) or not (–) with PMA for 20 h, were analysed by western blot using antibodies against constant exons of CD44 (Hermes3 antibody; top panel) and against the v5 exon of CD44 (VVF8 antibody; middle panel). Ponceau staining of the membrane is shown as a loading control (bottom). A 1/10 diluted extract of MCF10A was used to indicate the size of variant CD44 isoforms (CD44v). Arrowheads highlight the constitutive CD44 isoforms and the main isoforms containing the v5 exon. (**G**) The inclusion of CD44 variant exons, from the RNA-Seq studies in panel B. Differential splicing was evaluated by counting the reads covering the indicated exon-exon junctions. The relative levels of each junction are indicated as the percentage of inclusion or skipping junctions among all junctions involving the C5 exon (top panels) or involving the C16 exon (bottom panels). Significance were evaluated by using Wilcoxon signed-rank test, for α = 0.05 (two-tailed). Hashtag (#) indicates that there is sufficient evidence to suggest a difference between DKO and WT cells. The differences were also evaluated using a Student's *t*-test for matched-samples (two-tailed), with *P* < 0.05 (*), *P* < 0.01 (**).

Observation of these cells by phase-contrast microscopy showed that they had largely lost the cobblestone shape of the original HCT116 cells but had acquired an elongated spindle shape (Figure 1A, phase contrast). This fibroblastoid appearance was highly evocative of the HCT116 cells that initiated EMT upon inactivation of the two DNA methylase activities.

Consistently, immunofluorescent staining of the cells revealed a loss of the E-cadherin (CDH1) epithelial marker in the DKO cells, as well as a dramatic increase in the mesenchymal N-cadherin (CDH2) (Figure 1A). Western blot analysis confirmed the quantitative decrease in E-cadherin and revealed increased accumulation of Vimentin (VIM) in the DKO cells (Figure 1A).

To ensure that the mesenchymal drift of the DKO cells was not a recent consequence of their culture conditions, we reanalyzed five existing sets of publicly available RNAseq data on DKO HCT116 cells (Supplementary Figures S1C, S1D, S1E, S2). This analysis confirmed the increased expression of several mesenchymal markers, including CDH2, TWIST1/2, ZEB2, LEF1 and VIM, in DKO cells compared to the parental HCT116 cells. In contrast, the expression of the epithelial markers TCF4 (TCF7L3), AP2α, ESRP1 (RBM35a), RBFOX2, EPCAM and DSP (*desmoplakin*) was reduced (Figure 1B, Supplementary Figure S1E) (51,73,74). Additional regulators or markers of EMT were upregulated such as TGFβ2, WNT5B and WNT11, while WNT16 and FGFR2 were repressed (Supplementary Table S1).

EMT is associated with multiple alternative splicing events, including the transition from a CD44v isoform including alternative exons to a shorter CD44s isoform lacking these variant exons (Figure 1C). Decreased levels of CD44 variant exons were readily observed by RT-qPCR comparing DKO to WT cells. Monitoring several variant exon pairs over five days of culture showed that the inclusion of variant exons remained different between DKO and the parental HCT116 WT cells regardless of these cells were proliferating or confluent, which indicates that reduced variant exon inclusion in DKO is independent of their growth conditions. The increase in CD44s skipping all the variant exons (C5–C16 exon pair) in DKO cells confirmed the decrease in the variable exon inclusion. (Figure 1D). RT-PCR detecting larger variant isoforms confirmed that the inclusion of variant exons in the mature CD44 mRNA remained different between DKO and WT cells, while the expression of constant exons (C2 to C5) was comparable (Figure 1E and Supplementary Figure S1E). A reduction in the complexity of the pool of CD44 isoforms was also apparent at the protein level, as visualized by Western blots using two different antibodies, either detecting all forms of CD44 (Hermes3) or specifically recognizing an epitope on the v5 exon (Figure 1F).

We also tested the effects of PMA, a phorbol ester that activates the PKC pathway and increases the inclusion of CD44 v5 variant exons by affecting splicing factors activities (55,75), and of other exons by promoting a slowing-down of the elongating process of RNAPII and the recruitment of splicing factors (55,76). In both DKO and WT cells, PMA treatment increased the inclusion of variant exons, indicating that in the absence of meDNA, splicing is still responsive to regulation by external cues (Figure 1E). However, variant exon inclusion remained less frequent in the DKO cells than in the WT cells, as documented at both the RNA and protein levels (Figure 1E and F). Immunofluorescent staining with the anti-CD44v antibody further revealed that the levels of the longer isoforms of CD44 (containing the v7v8 exons) were also decreased in the DKO cells (Figure 1A). Finally, RNA-seq from earlier studies confirmed the different levels of CD44 global expression and exon skipping between DKO and WT cells (Figure 1G and Supplementary Figure S1E).

Meta-analysis of these RNA-seq data further revealed that several genes important for EMT other than CD44 were differentially spliced in DKO cells, including CTNNB1 (β-catenin) and ECT2 (Supplementary Tables S1 and S2A).

In conclusion, DNMT1/3b-depleted HCT116 cells, which are widely used to investigate the consequences of meDNA-loss on gene expression, undergo a clear loss of epithelial differentiation. This process is correlated with the skipping of CD44 variant exons, while the responsiveness to the PMA is conserved. These profound changes in differentiation status may blur or hide potential direct effects of meDNA on alternative splicing decisions.

### Inactivation of DNMT1 and DNMT3b in HCT116 cells results in the modified expression and splicing of a wide range of regulators of transcription and splicing.

**Figure 2.**
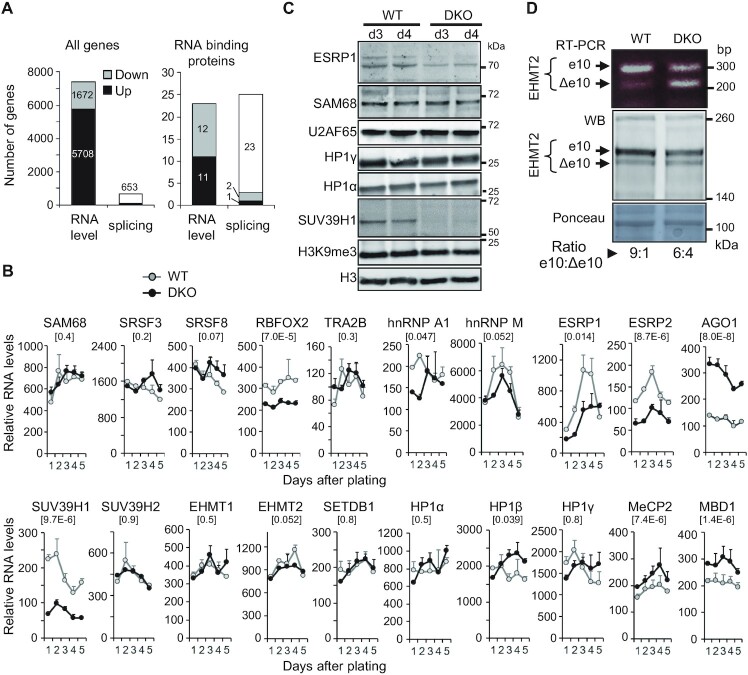
Modified expression and splicing of regulators of transcription and splicing in DKO HCT116 cells. (**A**) Number of genes found differentially expressed or spliced in DKO versus WT HCT116 cells in the meta-analysis of 4 independent studies containing 6 RNA-seq datasets for each cell. Genes with a log2 fold change >1 and *P*-value <0.05 (paired Test) were considered differentially expressed as explained in Supplementary Figure S1C, S1D. White portions of the bars indicate the genes which are not differentially expressed but found alternatively spliced. The MAJIQ package (68) was used to detect differentially spliced genes with high confidence P(|dPSI|>0.2)>0.95, (differential Percent of Splicing Index) between DKO and the parental HCT116 cells, as explained in Supplementary Figure S1F. The list of the tested RNA binding proteins is available in Supplementary Table S1. (**B**) Transcript levels of the indicated genes were evaluated as in Figure 1D by RT-qPCR in DKO (black line) and WT (grey line) HCT116 cells harvested over 5 days after plating. (**C**) Whole protein extracts from WT or DKO HCT116 cells, 3 and 4 days after plating were analysed as in Figure 1A by western blot using antibodies directed against the indicated proteins. (**D**) Skipping of the EHMT2 variant exon 10 in DKO cells. Top panel, semi-quantitative RT-PCR performed on total RNA to detect the inclusion of e10 or its skipping (Δe10) using previously described primers (59). Bottom panels, western blot analysis (middle) of EHMT2 isoforms in total protein extract, and ponceau-stained membrane (bottom) as a loading control. Ratio of EHMT2 protein spliced isoforms in cells were quantified by Image J using two independent sets of extracts on non-saturated images.

To gain insights into the mechanisms linking the loss of meDNA to modified alternative splicing of CD44, we first examined the impact of DNMT1 and DNMT3b inactivation on the expression of known regulators of CD44 splicing. For that purpose, we analyzed publicly available RNA-seq data (Supplementary Figure S1C). Inactivation of the two DNMTs caused extensive reprogramming of the transcriptome and resulted mostly in gene activation, as expected from the reduced promoter methylation (Supplementary Figure S1D and Figure 2A). Transcriptome variations also originated from differentially spliced genes, with 653 genes producing variant transcripts (Figure 2A and Supplementary Table S2). Note that a very small proportion of these genes were also differentially expressed (56 were increased and 52 decreased), indicating that variant mRNAs induced by the loss of meDNA were rarely a consequence of modified transcription or RNA stability.

When examining a series of 277 genes encoding RNA binding proteins with reported activity in splicing regulation, we observed that 48 were modified either in their expression or in their splicing (Figure 2A right panel and Supplementary Table S2B). This indicated that the splicing machinery in DKO cells is subject to numerous changes. Charts for genes of interest are shown in Supplementary Figure S2, while RT-qPCR validation experiments conducted over a period of five days to avoid the influence of cell proliferation and confluency, are shown in Figure 2B.

When specifically examining splicing factors known to affect the exon composition of the CD44 message, we particularly noted the downregulation of ESRP1 (Figures 1B and 2B, C), a splicing factor previously reported to be repressed during EMT (54,77). Likewise, RBFOX2 and NBML1, which are known to affect CD44 splicing, were downregulated in both DKO cells and during EMT (78–80). In contrast, the expression of many other splicing factors affecting CD44 splicing and associated with EMT (reviewed in (51)) was not significantly affected in the mutant. These included SAM68 (55,75,76), SR proteins (SRSF1, SRSF3, SRSF8, SRSF10) (81–83), SRRM1 (75) and hnRNPs (hnRNPA1, hnRNPM) (84–86) (Figure 2B, C; Supplementary Figure S2, Table S2A). Finally, TRA2B (87) showed no change in expression, but was found to be differentially spliced (Supplementary Table S2A, S2B; and Figure 8F)

In addition to splicing factors, alternative splicing was shown to be affected locally—at the gene body locus - through chromatin marks and their associated readers. We therefore investigated whether some of the epigenetic modifications were altered in DKO cells.

We first examined the expression of chromatin modifiers in the DKO cells. Among these, we noted a clear decrease in both SUV39H1 expression and protein accumulation (Figure 2B, C and Supplementary Figure S2). In parallel, we noted increased expression of AGO1 transcripts (Figure 2B and Supplementary Figure S2), a component of the RNAi machinery involved in recruiting H3K9 methylase to the coding region of CD44 (32). Other factors in the PIWI family, including PIWIL3 and PIWIL4 (Supplementary Table S1) were also increased in DKO cells. In addition, we identified chromatin modifiers whose splicing but not their overall expression was affected (Supplementary Table S2A). For instance, cassette exon 10 of *EHMT2*, which does not modify the catalytic activity of the enzyme (59), but affects its nuclear localization (88) was decreased in DKO cells, as visualized both at the RNA level by RT-PCR and at the protein level by Western blot (Figure 2D). This splicing change may also participate in the epithelial differentiation since the interaction of EHMT2 with SNAI1 favors E-cadherin transcription (89).

Taken together, these data highlight an extensive reprogramming of the splicing machinery in the DKO cells. This supports that changes in CD44 alternative splicing are caused by a complex combination of modified expression and alternative splicing of splicing factors.

### DKO cells exhibit modifications of H3K9me3 epigenetic mark, HP1γ and MBD1 recruitment, and of RNAPII phosphorylation within the CD44 gene

**Figure 3.**
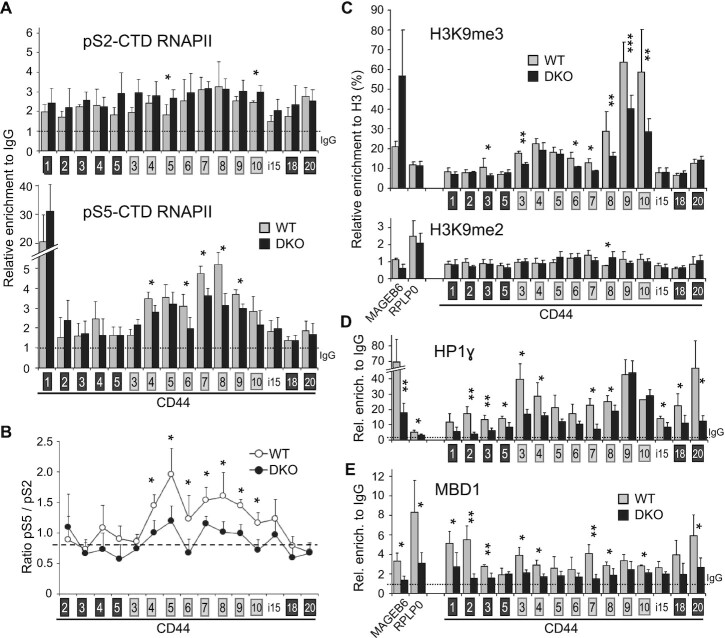
Modifications in DKO HCT116 cells of RNAPII phosphorylation and of epigenetic markers on the CD44 gene. ChIP walking experiments were carried out with chromatin from DKO (black bars) and WT (grey bars) HCT116 cells, with antibodies against RNAPII CTD serine5 (S5p) or serine2 (pS2) phosphorylation (**A**), H3, H3K9me3, H3K9me2 (**C**), HP1γ (**D**) and MBD1 (**E**). Immunoprecipitated DNA was quantified by qPCR with primers targeting indicated regions. Enrichment in HP1γ, MBD1 and RNAPII are expressed relatively to the signal obtained for ChIP using non-immune IgG and relative enrichment in H3K9me3 and H3K9me2 are expressed as a percentage of H3 detection. Values are means (±dev.) of at least three independent experiments. Statistical significance of the differential levels were evaluated using Student's t-test (two-tailed), with *P* < 0.05 (*), *P* < 0.01 (**), *P* < 0.001 (***). (**B**) The ratios between pS5 and pS2 RNAPII CTD were calculated for each loci by using the averages shown in panel A. The asterisks indicate the positions where a significant difference has been found for either pS5 or pS2. The dashed line indicates the average level covering the constitutive exons.

We next investigated the impact of meDNA on RNAPII phosphorylation within the CD44 gene using antibodies enriched for either for the phospho-Ser2 (pS2) or phospho-Ser5 (pS5) versions of the C-terminal domain (CTD). In the parental WT cells (gray bars), both antibodies detected the expected accumulation of RNAPII over the variant region (Figure 3A, v4 to v10), reflecting a reduced elongation rate over this region (76). This accumulation was best observed with the anti-pS5-CTD antibody, consistent with the enrichment of this phosphorylation within the pool of paused RNAPII. The levels of pS2-CTD RNAPII were essentially unaffected in the DKO cells (Figure 3A top panel). In contrast, the accumulation of pS5-CTD RNAPII was specifically reduced over the variant region, and calculating the ratio of pS5 to pS2 revealed a clear decrease in the proportion of pS5-CTD in the DKO mutant in the variant region (Figure 3A bottom panel and Figure 3B). The reduced accumulation of pS5-CTD RNAPII was suggestive of an increased RNAPII elongation rate in the variant region, possibly as a consequence of a modified chromatin environment.

Analysis of ChIP-Seq data from either WT or DKO HCT116 cells (11,90) did not uncover any significant variations in epigenetic marks specific for either promoters (H3K4me3, H2AZ), enhancers (H3K27ac, H3K4me1), or transcribed gene bodies (H3K36me3) within the CD44 variant region. Likewise, CTCF binding also seemed unaffected (Supplementary Figure S3). In contrast, ChIP-PCR assays revealed reduced levels of H3K9me3 on CD44 variant exons v8, v9, v10 and, to a lesser extent, on v3, v6 and v7 (Figure 3C). These locally reduced levels of H3K9me3 could be correlated with reduced accumulation of the histone methylase SUV39H1, and modified splicing of EHMT2 (Figure 2C and D). We also observed reduced recruitment of HP1γ over the entire length of the CD44 gene body (Figure 3D). Western blots confirmed that these changes were not due to genome-wide effects, as the overall levels of H3K9me3 and HP1 proteins were unaffected in the DKO cells (Figure 2C). Notably, the distribution of H3K9me3 and HP1γ over the HCT116 CD44 gene body was equivalent to that described previously in HeLa cells (31,32).

We finally examined the binding of methyl-CpG-binding domain (MBD) proteins, which are primary candidates for the readout of meDNA events and likely recruiters of chromatin remodelers or modifiers. ChIP-PCR assays using a validated antibody documented a reduction in the recruitment of MBD1 to the body of the CD44 gene in the DKO cells, an expected consequence of the reduced levels of meDNA (Figure 3E), while the expression of MBD1 was weakly increased in the DKO cells (Figure 2B). Notably, as MBD1 was subject to alternative splicing at the level of one of its carboxyl-terminal exons, we cannot rule out that the activity of MBD1 may be affected to some extent in the DKO cells (Supplementary Table S2A).

Taken together, our observations suggest that the reduced recruitment of MBD proteins, HP1γ and possibly other H3K9me3-binding proteins downstream of the loss of meDNA may generate a chromatin environment promoting an increased RNAPII elongation rate and a subsequent modification of splicing decisions.

### Short term depletion of DNMT1 in HeLa cells decreased DNA methylation and inclusion of CD44 variant exons

**Figure 4.**
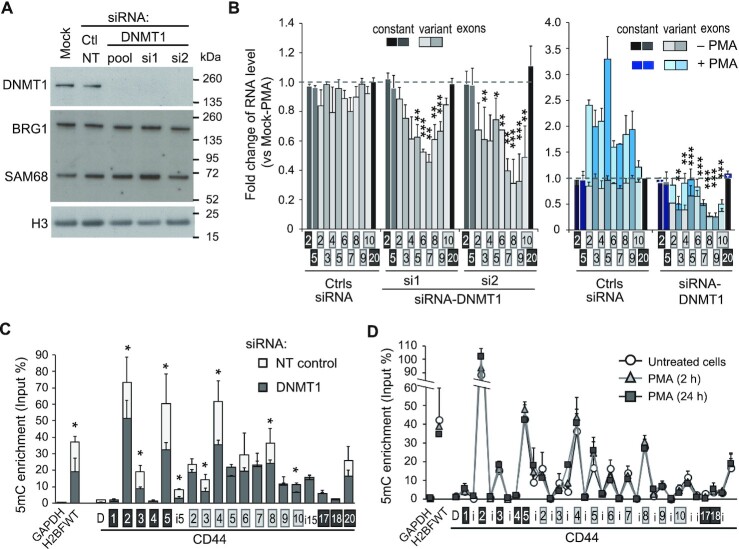
CD44 variant exon levels depend on DNA methylation in DNMT1 depleted HeLa cells. (A, B) HeLa cells were transfected with two individual siRNAs or a commercially available pool of 3 siRNAs targeting DNMT1,or control siRNAs, either targeting GAPDH or non-targeting (NT). The cells were treated with or without PMA for 2 h before harvesting. (**A**) Western blot analysis of cell extracts using the indicated antibodies (see Supplementary Figure S4B for PMA-treated cell extract). (**B**) RT-qPCR analysis with primers aligning on the indicated exons. Relative RNA levels normalized to the RPLP0 gene as a reference, were used to calculate the fold change relative to the mock untreated cells (set to 1, dashed line). Statistical significance of the exonic differential levels upon DNMT1 depletion (without PMA; left panel) was calculated based on 4 independent experiments using Student's test (two-tailed), with *P* < 0.05 (*), *P* < 0.01 (**), *P* < 0.001 (***). The right panel shows the effect of PMA (blue bars) in these conditions of transfection with the DNMT1 siRNAs pool. Constitutive exons showed no significant difference between NT siRNA transfected cells (Ctrls) and mock transfected cells. The variant exons were increased by PMA in the presence of DNMT1. (C, D) CD44 intragenic DNA methylation is dependent on DNMT1 expression and independent of PKC activation. (**C**) DNA from HeLa cells transfected with the pool of DNMT1 siRNAs were analyzed by methylated DNA immunoprecipitation (MeDIP) using the 3D33 antibodies directed against methylated DNA (5mC) or non-immune IgG as negative control. The levels of 5mC were expressed as a percentage of the input DNA quantities for each of the qPCR primers covering the CD44 gene : intronic sequences (i) are indicated by white boxes, constant exons are indicated by black boxes and variant exons by light grey boxes, D is a distal sequence upstream to the TSS. The germline-specific H2BWT gene was used as a positive control for DNA methylation in the somatic HeLa cells, while the GAPDH promoter, a non-methylated CpG-rich region, was used to evaluate the background signal. The control IgG was at least 100 times less enriched and is not represented. Data are the average (±dev.) of 3 independent experiments. (**D**) HeLa cells were treated with or without PMA for the indicated time and the relative enrichment in 5mC was evaluated by MeDIP as in panel C.

To further explore the potential direct effect of meDNA on alternative splicing in a simplified model, we turned to short-term depletion of individual DNA methyltransferases, thereby overcoming transcriptomic changes linked to adaptation to a permanent loss of these enzymes. To accomplish this, we depleted DNMT1 in HeLa cells using two different siRNAs independently. As expected, both the mRNA and protein of DNMT1 were dramatically reduced, and the expression of the imprinted gene H19 was increased (Figure 4A and Supplementary Figure S4B). Interestingly, the depletion of DNMT1 resulted in significant skipping of CD44 variant exons, similar to what we observed in DKO cells (Figure 4B). This decrease in CD44 variant exon inclusion was also observed when DNMT1 was depleted using a commercially available pool of three siRNAs. Consistent with our observation in DKO cells (Figure 1D), stimulation of the cells with PMA did not compensate for the decreased inclusion of the variant exons (Figure 4B, right panel). Note that the decreased inclusion was not correlated with modified expression of the SAM68 splicing factor (Figure 4A).

To correlate the decrease in variant exon inclusion with CD44 gene body meDNA, we carefully monitored 5mC levels by methylated DNA immunoprecipitation (MeDIP). As expected, the DNMT1 depletion promoted a significant decrease in meDNA inside the body of the CD44 gene, especially in the regions with the highest levels of 5mC (note changes at C2, C5 and v4 in Figure 4C). In addition, we observed that the absolute levels of 5mC were not correlated with the local CpG contents of the DNA, such as v4 and C3, which exhibit differences in 5mC but similar CpG contents (Figure 4C and Supplementary Figure S4C, arrows). This suggests some specificity in the selection of loci where DNMT1 maintains meDNA inside the CD44 gene. A part of this specificity could also be consequences of TET hydroxymethylase action or recruitment. These possibilities are compatible with meDNA affecting cotranscriptional events such as splicing.

We next used the MeDIP approach to explore possible feedback from splicing to the meDNA. Indeed, this phenomenon was documented for H3K36me3 (26,27), a histone mark involved in recruiting the *de novo* DNA methylase DNMT3b (9,23), which suggested a potential effect of alternative splicing on the 5mC level of variant exons. Here, we took advantage of the increased inclusion of CD44 alternative exons upon stimulation of the cells with PMA. In these experiments, PMA was added for short (2 h) and longer (24 h) periods to evaluate a potential delay in the establishment of meDNA changes (Figure 4D). We did not observe any change in intragenic CD44 meDNA upon PMA treatment, indicating that the equilibrium between *de novo* DNA methylation and demethylation is not modified by the regulation of alternative splicing. This is in accordance with the lack of inhibitory effect on the variant exon level of the depletion of the *de novo* DNA methylases DNMT3a or DNMT3b (Supplementary Figure S4D and S4E). In contrast, DNMT3a caused increased inclusion of several CD44 exons, but the reason for this effect will require further investigation.

To further investigate the pathways by which meDNA affects to impact on CD44 splicing, we explored whether a short-term depletion of DNMT1 would affect the expression of splicing regulators. In these experiments, we depleted DNMT1 using the two different siRNAs (Supplementary Figure S4A) and then measured gene expression using exon arrays. This approach confirmed the increased expression of H19 in the absence of DNMT1. However, the overall impact of DNMT1 depletion on the transcriptome was very limited, with only 117 genes affected by both siRNAs at the transcription level (Supplementary Figure S4F and Supplementary Table S3). Remarkably, among the 277 annotated splicing factors, only two spliceosome-associated cyclophilins (PPIH and PPWD1) and RBMX were downregulated by ∼1.5-fold. The skipping of CD44 variant exons upon DNMT1 depletion could not be explained by this weak downregulation of RBMX since it was shown to promote the skipping of a CD44v8-based splicing reporter minigene (91). The arrays confirmed that ESRP1 was not expressed in HeLa cells. None of the known regulators of CD44 alternative splicing were dysregulated at the expression or the splicing level. We cannot totally exclude that some alternative splicing events may not be detected by this exon-array technology, but these potential modifications did not change the levels of known variant exons or the average RNA level of the genes.

Thus, DNMT1 depletion appeared to be an opportunity to explore the effects of decreased meDNA on alternative splicing in the absence of major dysregulation of splicing factor expression. Examination of the exon array at the splicing level revealed that 12 genes were affected by both siRNAs (Supplementary Figure S4G, H). Several of these genes are related to cancer, including CD44, DST, GLS, GNAS, KIF1B and AHRR.

Taken together, these results supported the argument in favor of the direct effects of meDNA at a small number of genes, and suggested that the maintenance of meDNA by DNMT1 is necessary to stabilize the production of specific splicing isoforms.

### MBD1/2/3, but not MeCP2 and MBD4 contribute to the effects of DNA methylation on the inclusion of CD44 variant exons

**Figure 5.**
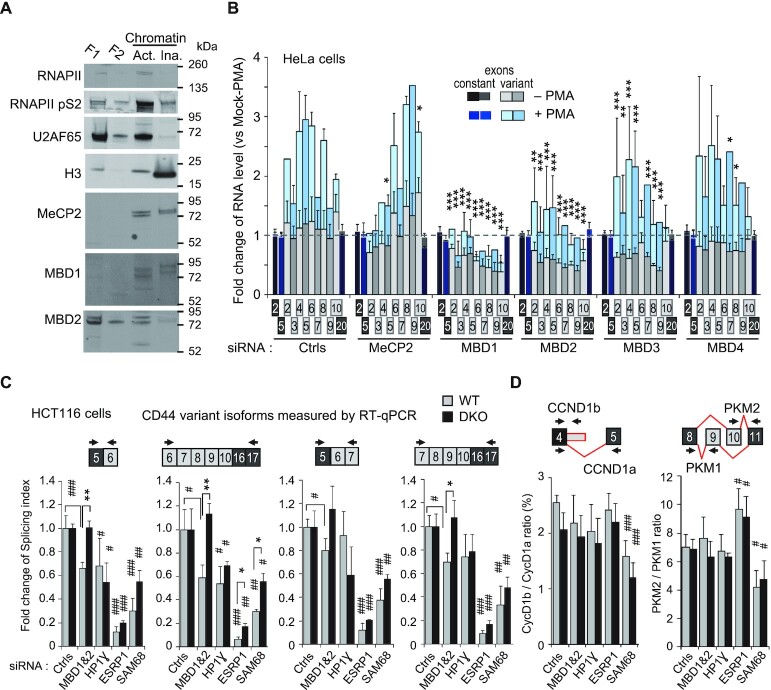
CD44 variant exon levels is dependent on MBD proteins in a meDNA-dependent manner. (**A**) HeLa cells were fractionated as described in Methods. Extracts were from detergent and hypotonic treatment (F1), no salt treatment (F2), allowing to detect active (‘Act.’) and inactive (‘Ina.’) chromatin after DNase treatment. Equivalent volumes of each fraction were loaded on an SDS-PAGE and proteins detected by western blot with the indicated antibodies. (**B**) HeLa cells were transfected with pool of 3 siRNAs targeting the indicated MBD proteins or with control siRNAs and then treated (+) or not (–) for 2 h by PMA. The RT-qPCR data were processed as described in Figure **4**B. (**C**) WT and DKO HCT116 cells were transfected with siRNA targeting the indicated genes or with control siRNAs (NT or against GAPDH). The indicated spliced forms were the major PCR products (∼90%) detected by RT-qPCR (Supplementary Figure S5E). Levels of spliced forms are expressed as splicing index using C5-C16C17 skipped form as a reference for the formula SI = variant / (variant + skipped)*100. CD44 v3v4 variant isoforms and unmodified constitutive C2-C5 exons are shown in Supplementary Figure S5F in addition of siRNA-targeted genes shown in Supplementary Figure S5D). (**D**) Alternative spliced genes CyclinD1 and PKM are shown as controls independent of the DNA methylation but dependent of SAM68 (see the text). Alternative splicing decisions depicted in red and PCR primers are indicated above the graphs. The data are expressed as the ratio between the two alternative isoforms. Data are expressed for each cell-type as fold change versus the average of control NT and GAPDH siRNAs (Ctrls). Statistical significance of the differential levels of isoform upon indicated depletions is indicated by hashtags (#) relative to GAPDH siRNA, or by asterisks (*) for comparison between WT and DKO cells.

To further explore a possible direct effect of DNA methylation on alternative splicing, we investigated the implication methyl-binding proteins (MBPs), under the assumption that these proteins were good candidates to mediate epigenetic information from the chromatin to the splicing machinery. Fractionation experiments on HeLa cells revealed that the MBPs MeCP2, MBD1, and MBD2 cosegregated at least partially with the elongating form of RNAPII (RNAPII pS2), which was compatible with a role for these proteins at actively transcribed genes (Figure 5A). As HeLa cells also express MBD3 and MBD4 (Supplementary Figure S5A), we methodically knocked down the products of the five MBP-encoding genes (Supplementary Figure S5B and S5C). The depletion of MBD1, MBD2, or MBD3 had significant inhibitory effects (*P* < 0.05) on the inclusion of CD44 alternative exons, while the depletion of MeCP2 and MBD4 did not (Figure 5B). These observations indicated that the outcome of CD44 pre-mRNA splicing was affected by the availability of a subset of MBPs.

We next investigated whether the effects of MBPs on alternative splicing were dependent on their 5mC-reader function. To that end, we simultaneously depleted MBD1 and MBD2 in in either WT or DKO HCT116 cells. The efficiencies of the depletions were similar in WT and DKO cells (Supplementary Figure S5D). Because HCT116 cells use a wider range of variant exons than do HeLa cells, we examined CD44 alternative splicing using RT-qPCR spanning over multiple exons. This reported on the population of produced transcripts more accurately than pair-wise amplification of variant exons, as such pairs may be present in several different transcripts. Whether these longer PCR products correspond mainly to the indicated isoform was systematically verified on gel analysis (Supplementary Figure S5E) and matched with their predicted sequence.

The depletion of MBD1 and MBD2 in the WT HCT116 cells, as in HeLa cells, resulted in reduced the levels of transcripts including variant CD44 exons (Figure 5C and Supplementary Figure S5G). Remarkably, this decrease was not observed in the DKO cells, indicating a strict dependency on DNA methylation (Figure 5C). The levels of CD44 variant exon inclusions were constitutively lower in DKO cells than in WT cells (Figure 1D, 1E, and Supplementary Figure S5F), we verified that these levels remained subject to regulation in the DKO cells. In agreement with this regulation, depletion of the splicing regulators HP1γ, ESRP1 and SAM68 reduced CD44 variant exon inclusion (Figure 5C, and Supplementary Figure S5G). Depletion of the splicing regulator TRA2B had a similar effect, but only at a subset of variant transcripts (Supplementary Figure S5H). None of these depletions had any effect on the relative level of the CD44 constant exons (Supplementary Figure S5G). Depletion of the splicing regulators also suggested that HP1γ may not be systematically affected by DNA methylation, but ESRP1, SAM68 and TRA2B displayed a so far overlooked sensitivity to this epigenetic mark as their depletion had a stronger impact on exon inclusion in WT than in DKO cells at most positions. To document that this phenomenon was specific to alternative splicing events that are sensitive to intragenic DNA methylation, we further tested the effects on splicing events affecting Cyclin D1 (CCND1) and Pyruvate Kinase M (PKM), which are known to be dependent on SAM68, but independent of DNA methylation and ESRP1 splicing factor ((92–94) and Figure 1). Despite high levels of 5mC (39), alternative splicing of the PKM exon 10 was not detected in our meta-analysis of the DKO transcriptome (Supplementary Table S2A). In parallel, our RT-qPCR approach showed little or no decrease in the inclusion of CCND1b (less than 15%, *P* < 0.05) and no change in PKM variants, when comparing DKO to WT HCT116 cells (Figure 5D). Furthermore, we observed no difference in the impact of SAM68 depletion in WT and DKO cells, with relative levels of CCND1b reduced by 40%, and PKM2 reduced by 35%, in both cell lines (Figure 5D). We also noted that ESRP1 favors the PKM1 nononcogenic form at an equivalent amplitude in both cell types. These data suggest that alternative splicing regulation by splicing factors can be dependent on intragenic DNA methylation when alternative splicing events are dependent on DNA methylation.

Together, these knockdown experiments support a direct effect of meDNA maintenance on CD44 gene splicing, detected by MBPs and deciphered by splicing regulators.

### Targeted DNA methylation increases CD44 variant exon inclusion

To provide unbiased evidence for a direct effect of DNA methylation on alternative splicing, we next targeted the catalytic domain (CD) of DNMT3b to multiple CD44 variant exons using a CRISPR/dCas9 fusion in HCT116-DKO cells (95,96). Possible effects mediated by steric hindrance were controlled using a CRISPR/dCas9 fusion with the CD from the catalytically inactive DNMT3b(E697A) mutant.

**Figure 6.**
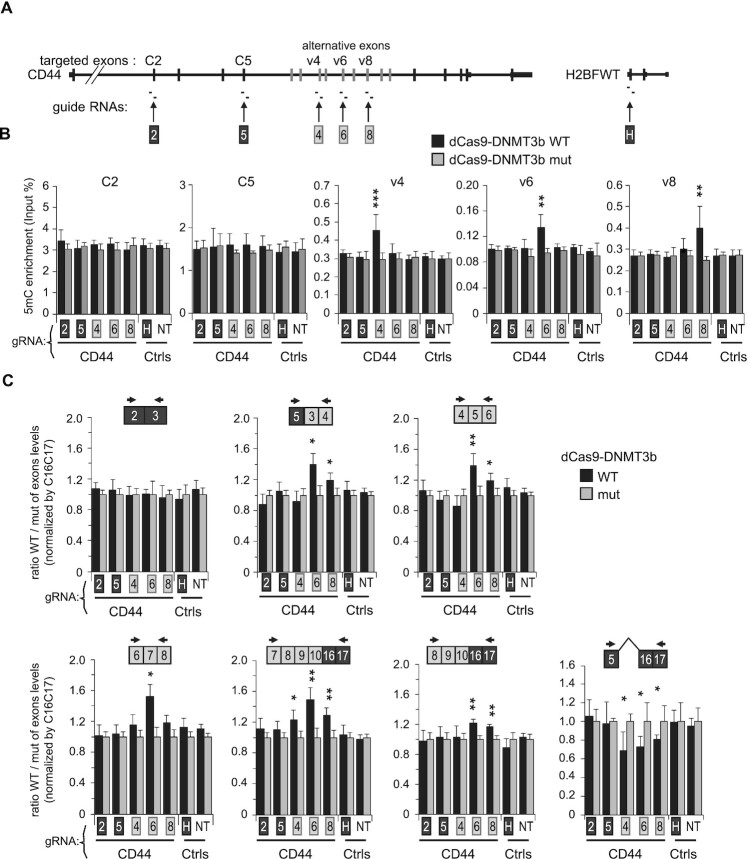
dCas9-DNMT3b mediated DNA methylation of CD44 variant exons in HCT116 DKO cells promotes their inclusion. (**A**) Two guide RNAs targeting indicated exons were used to drive dCas9 fused to the catalytic domain of DNMT3b in a wild-type version (WT) or in an inactive mutant form (mut). Two guide RNAs targeting H2BFWT gene (H), or one non-targeting guide RNA (NT) were used as negative controls (Ctrls). (**B**) MeDIP assays were conducted as previously described with DNA extracted from DKO-dCas-DNMT3b WT/mut cells with indicated guide RNAs. Each targeted exon indicated on the top of panels were analysed by qPCR and 5-mC enrichment is expressed as proportion of Input DNA (%). Values are means (± std dev.) of at least three independent experiments. (**C**) Analysis of CD44 alternative splicing by RT-qPCR. The levels of the indicated forms on top of each panel were normalized to the level of C16C17 constitutive exons and expressed as a fold change over the dCas9-DNMT3b/mut level (set to one for each gRNA). Ratio of CD44 constant exons C2-C3 is shown as unchanged exon junctions and CD44 isoform skipping the variant exons (meaning the C5 to C16C17 exon junction) is shown on right. Values are means (± dev.) of at least three independent experiments. Statistical significance relative to H and NT negative control guide RNAs is calculated by unpaired Student's *t*-test (two-tails) and indicated by asterisks (*P* < 0.05 (*), *P* < 0.01 (**), *P* < 0.001 (***)).

Guide ARNs (gRNAs) were designed to target variant exons with the highest levels of DNA methylation in HeLa or and WT HCT116 cells (Figures 4C and 6A). Targeting of the DNMT3b CD successfully increased DNA methylation at three positions, namely, exons v4, v6 and v8 as determined by MeDIP (Figure 6B). The targeting of exons C2 and C5 did not impact on local DNA methylation for unknown reasons, but the gRNAs matching these positions were maintained in the experiments as negative controls. When the DNMT3b CD construct was replaced by the catalytically inactive construct, none of the gRNAs modified local DNA methylation.

Targeted DNA methylation significantly increased the proportion of transcripts including variant exons, as assayed by RT-qPCR at multiple positions (Figure 6C). Increased inclusion was observed for exons directly targeted for modified DNA methylation, but also for neighboring exons. The reflectance of the targeted DNA methylation on inclusion of neighboring exons may be explained by a slowing-down of the RNAPII encountering DNA methylation (Figure 3B). Modified recruitment of splicing factors to regions of splice site commitment may also be a source of ectopic effects of DNA methylation (14–16). Finally, we note that inclusion of variable exons naturally tracks from 5′ to 3′, such that an isoform containing any given variable exon is likely to contain many downstream variable exons (97–99). These suspected sources of ectopic effects may also explain why the v4 gRNA significantly increased inclusion of downstream variant exons without inducing any detectable change in v4 exon inclusion. For increased robustness of our splicing analysis, we verified that the abundance of the isoforms resulting from the skipping of the all the alternative exons was reduced when variant exon inclusion was increased (Figure 6C, right panel). In addition, guide RNAs not targeting variant exons (C2 and C5) and not resulting in modified DNA methylation in the region of variant exons were shown not to affect alternative splicing.

Taken together, these results illustrated that local intragenic DNA methylation directly impacts on alternative splicing decisions, but that the alternative splicing events can be also influenced at distance of the DNA methylation site.

### Correlation between meDNA and alternative splicing at a subset of splicing regulator genes in patients with acute lymphocytic leukemia

The observations described above suggested that the splicing of a subset of genes is regulated by meDNA. With the objective of validating this observation in the context of cancer, we examined a cohort of pediatric patients with acute lymphocytic leukemia (B-cell ALL) for which both methylome and transcriptome data were available with their normal counterparts as controls, i.e. healthy precursor B-cells isolated from umbilical cord blood (HBC) (61).

The methylome of these cells was mapped using a methylated-CpG island recovery assay (MIRA) which uses the methyl binding activity of MBD2B and MBD3L1 to enrich in methylated genomic DNA fragments (100). Serendipitously, this assay also provides indications on the loci of MBD binding. The original analysis of these data revealed multiple changes in meDNA associated with ALL, with approximately two thirds of the differentially methylated regions (DMRs) showing hypomethylation and preponderant intergenic and intronic localization (61). For the purpose of the current study, we focused on DMRs located at regions involved in alternative splicing.

In addition to extensive transcriptional reprogramming as described previously (61), the ALL cells exhibited many variations of transcript isoform expression, since 2034 genes were differentially spliced with high confidence when comparing the patient cells to the HBC cells (Supplementary Figure S6C and Table S4). Almost 30% of the genes differentially spliced in ALL were also downregulated in their overall expression and 5% were upregulated. This suggests that in ALL, unlike what we observed in the DKO cells, a proportion of the alternative splicing could arise from transcriptional misregulation or result in nonsense-mediated decay. Notably, we recovered the differentially spliced genes CD45/PTPRC and PKM which are dependent on the DNA methylation and on the factors CTCF and CTCFL, respectively (38,39,101). DNMT genes were upregulated, while TET genes were downregulated ((61) and Supplementary Figure S6E), indicating that any change in meDNA is unlikely to be a simple consequence of variation in the levels of their gene products.

We next examined the 12 genes affected whose splicing was affected by DNMT1 knockdown in HeLa cells (Supplementary Figure S4G). Among these, 7 were expressed in ALL and pre-B cells, including 5, namely CD44, RABGAP1L, DST, GNAS and GLS, that displayed modified splicing between ALL patient cells and HBCs. Among these, CD44 and GLS displayed changes in splicing involving the same exons, and only CD44 showed variations consistent with those observed in DNMT1-depleted HeLa cells.

**Figure 7.**
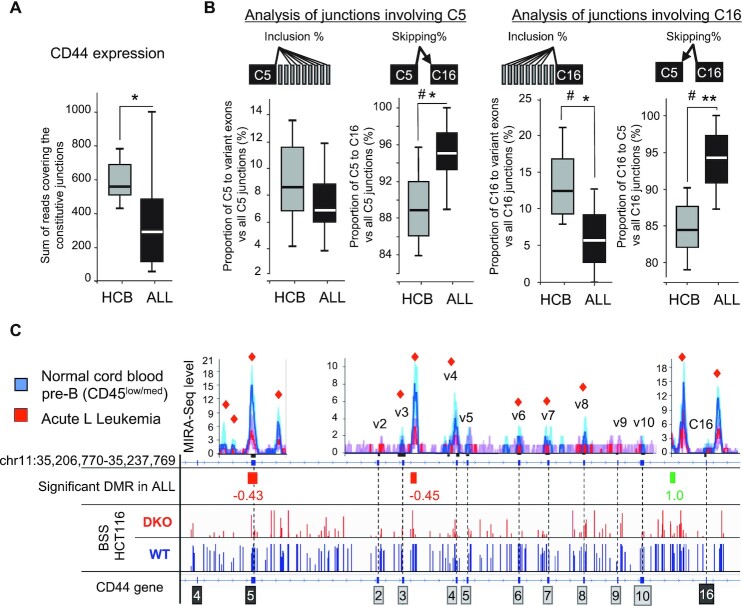
CD44 variant exons are significantly less included in ALL samples compared to pre-B cells from Human Cord Blood (HCB). (**A**) Global expression of CD44 was evaluated by counting the total of normalized reads covering the constitutive exon-exon junctions in each of the 8 HCB control or the 9 selected ALL samples showing comparable levels of CD44 expression (Supplementary Figure S6D). (**B**) The proportions of skipped and included junctions were calculated by counting reads of all the junctions, detected by at least two reads, between the indicated constant exon and all the variant exons. Significance was evaluated by using Student's t test (one-tailed), where *P*-values are indicated as < 0.05 (*) or < 0.01 (**)). Significance was also evaluated using the Wilcoxon ranked test: where # indicates that there is sufficient evidence to suggest a difference between ALL and HCB cells with α = 0.05 (one-tailed). (**C**) MIRA-Seq counts, represented by bin levels (top graphs) from the 18 ALL samples(red) and the 20 pre-B samples (blue). Individual tracks are shown in Supplementary Figure S6F. The lines indicate the median while the shadowed areas represent quartiles. The red squares indicate a significant difference (*P* < 0.05) between bin levels. Values indicate the log2 fold change of the Differentially Methylated Regions (DMRs) in ALL versus normal pre-B after normalization and Bonferroni's correction for multi-testing *P* < 0.05. For comparison, genome view of Bisulphite-Seq (BBS) from HCT116 and DKO cells is shown at the bottom.

Since CD44 appears to be a potential marker linking cancer and meDNA, we analyzed its splicing in more detail in ALL patients. As the expression of CD44 was significantly reduced and more variable in the patient cells (Figure 7A), we focused splicing analysis of this gene on the 9 ALL samples where the accumulation of CD44 mRNA was within the range observed in the HCB controls (Supplementary Figure S6D). To evaluate the inclusion level of the CD44 variant exons in these RNA-seq samples, we used the same strategy as the one described for HCT116 cells. We observed that the number of reads covering junctions from variant to constitutive exons was decreased in ALL cells in comparison to normal HCB cells while the abundance of reads spanning the C5–C16 junction of constitutive exons was significantly increased (Figure 7B).

As in the HCT116 DKO cells and in the HeLa cells subjected to DNMT1 knockdown, the reduced inclusion of CD44 variant exons was associated with decreased levels of meDNA at several positions inside the body of the gene as illustrated by MIRA-seq data (Figure 7C and Supplementary Figure S6F for individual tracks of MIRA-Seq). These data show that CD44 variant exons may function as sensors of intragenic 5mC variations that occur during tumor processes.

### Only rare alternative splicing events may function as sensors of DNA methylation

**Figure 8.**
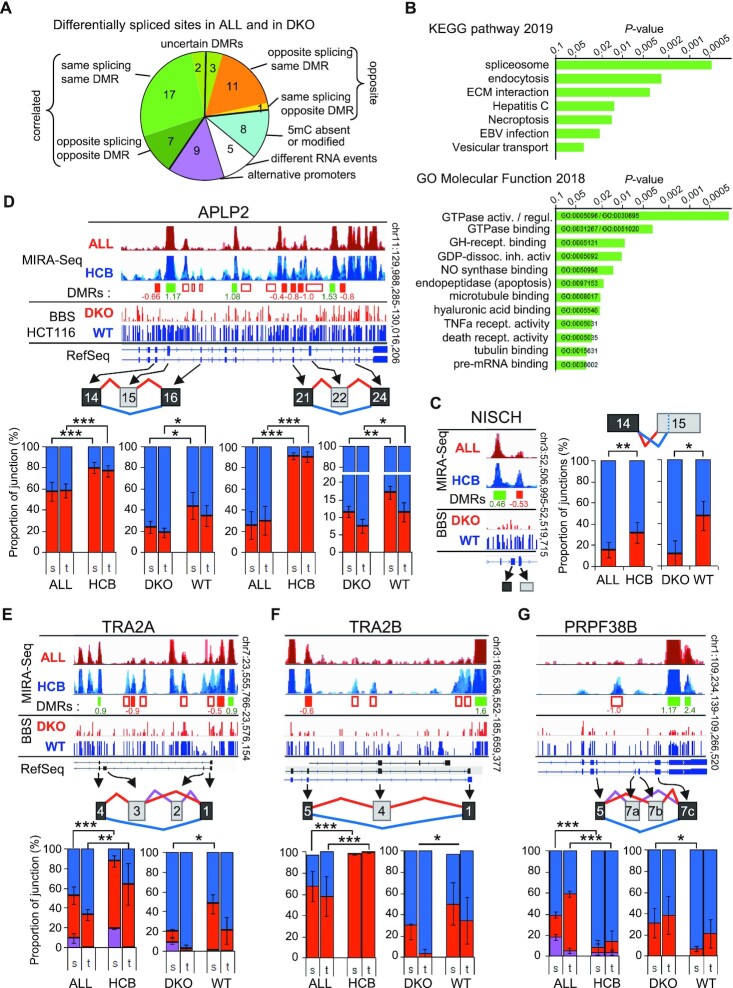
A few differentially spliced genes may be an indication of DNA methylation and its biological relevance. (**A**) Differentially spliced genes from DKO (Supplementary Table S2) and from ALL (Supplementary Table S4) were compared to identify common splicing sites. RNA events were categorized as indicated. (**B**) Pathway analysis performed using Enrichr (127) on the 23 differentially spliced genes correlated with changes in DNA methylation from panel **A** (green quarters). (**C–G**). Examples of genes with common splicing events in DKO versus WT HCT116 cells and in ALL versus HCB pre-B cells. The overlaid MIRA-seq tracks set at the same scale, for ALL and HCB samples spanning the region containing the regulated cassette exon (in grey boxes). Changes in the DNA methylation levels were evaluated, and displayed as decreased (red boxes) or increased (green boxes) DMRs below. When found to be significant (*P* < 0.05) after normalization and Bonferroni's correction for multitesting, log_2_ fold change is indicated and boxes are colored. In addition, empty boxes indicates obvious DMRs. Below, the Methyl-seq (BBS) from DKO and WT HCT116 cells is displayed above genome view of the RefSeq genes. The graphs are the quantification of the RNA alternative splicing of each indicated variant exons detected with high confidence *P*(|dPSI| > 0.2) > 0.95 by MAJIQ. For each sample, counts of reads covering the indicated junctions were used to calculate the proportion of each junction involving the source exons (s) or the target exons (t), and represented as histogrammes of percentage of inclusion (red and pink) and skipping (blue). The six RNA-seq samples (APLP2), or the polyA+ RNA samples (NISCH, TRA2A, TRA2B, PRPF38B) of DKO and HCT116 cells were used to calculate the averages (± dev.) and the Student's *t* test (one-tailed): *P* < 0.05 (*), *P* < 0.01(**), *P* < 0.001 (***). The eight HCB samples and the 12 ALL samples selected in Supplementary Figure S6A and S6B, were used for the same calculation.

We next investigated whether DNA methylation would enable the prediction of alternative splicing events affecting genes other than CD44. For that purpose, we compared the genes that were differentially spliced in ALL with those affected by DNMT1/3b inactivation in HCT116 cells (DKO). Among the differentially spliced genes in ALL, we identified 63 genes sharing at least one differentially regulated splice site with DKO cells. Manual curation with IGV and VOILA identified 49 genes subject to the same local variation in splicing in both cellular models (Figure 8A, Supplementary Table S5). The remaining genes included 9 cases of alternative promoters, and five cases where alternative splicing events were different in ALL and DKO cells, even when sharing a common splice site. Among the common splicing events 75% (41/49) were located in the neighborhood of regions subject to differential methylation (DMRs). Among these, modified alternative splicing was consistent with the changes in 5mC levels in ALL and in DKO cells only in 58% (24/41) of cases (Figure 8A, green wedges), while there seemed to be no correlation in 30% (12/41) of the events (Figure 8A, orange and yellow wedges). Thus, statistically, genes whose DNA methylation is predictive of the outcome of alternative splicing are rare.

For the 24 genes for at which splicing was linked to DNA methylation in both ALL and DKO cells, KEGG pathway analysis revealed an enrichment in the pathway ‘spliceosome’ (ko03040, adjusted *P* value = 0.021) (Figure 8B). This highlighted, in the list of 24 genes, the presence of the regulator of CD44 splicing TRA2B, and of the splicing factors TRA2A and PRPF38B, for which variations at the same DMR correlated with the same splicing event in both ALL and DKO cells (Figure 8E–G). Gene Ontology (GO) analysis also identified enrichment in genes involved in cell-matrix interactions, such as CD44, CD47 and NISCH (Figure 8C), and several others involved in cell signaling pathways, such as TYK2, RELT, RAF1, ARHGAP12, ARFGAP2, RGS14, ARRB2 and APLP2 (Figure 8D), some defined as ‘GTPase activators’ (GO:0005096, adjusted *P* = 5.6E–3). As a control for these pathway analyses, examining the 25 genes from the remaining clusters (‘opposite’/‘5mC absent’/‘uncertain DMRs’) did not yield enrichment in the pathways listed above (Supplementary Figure S7A). This suggested that a subset of splicing factors or GTPase regulators could be directly sensitive to variations in meDNA through the modulation of their alternative splicing. Such sensors of meDNA may in turn trigger more global changes in alternative splicing associated with meDNA.

### Variant exon inclusion of CD44 is correlated with meDNA levels in an in vitro model of breast-tumor progression

In each model system examined in the present study, increased meDNA inside the body of the CD44 gene unvaryingly translated into increased inclusion of CD44 variant exons. In parallel, increased inclusion of CD44 variant exons is known as a marker of carcinoma metastasis and cancer stem cells (102,103). This prompted us to explore the meDNA-to-splicing correlation in an *ex vivo* model of epithelial breast cancer progression. This isogenic cellular model included the nontumorigenic human breast epithelial cell line MCF10A cells, cells derived from MCF10A by transformation with activated RAS(T-24), and two cell lines derived therefrom that reproducibly form either ductal carcinoma in situ (DCIS)-like lesions or metastatic carcinomas (CA1A) in xenografts (104–106). This series of cell lines, sharing the same genetic background and origin, constitutes a good model for tumor progression to compare different cellular and molecular properties (107).

**Figure 9.**
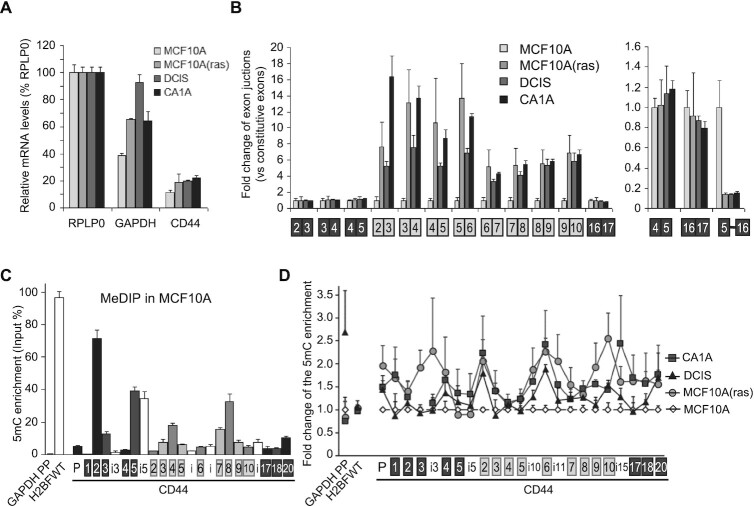
DNA methylation increases with the inclusion of CD44 variant exons in tumorigenic-derived MCF10A-cell lines. (A, B) RNAs extracted from the cell lines were subjected to RT-qPCR using primers specific to the indicated regions. (**A**) The CD44 mRNA level were the average of constitutive exons relative to RPLP0 used as an unmodified reference gene. (**B**) Left, levels of the variant exons (grey boxes) were normalized to the average of constitutive exons (black) and expressed as a fold change relative to the levels in MCF10A (set to one for each primer pair). Right; levels of the CD44 isoform skipping the variant exons (meaning the C5 to C16C17 exon junction). (C, D) MeDIP assays performed on purified DNA from MCF10A using the 3D33 antibody directed against methylated DNA or non-immune IgG as negative control. Enrichment in 5mC is shown relative to the DNA amount in each input sample. The levels of DNA methylation in MCF10A parental cells is shown in **C**. The control IgG was at least 100 times less enriched and is not represented. (**D**) Enrichment in meDNA in other MCF10A-transformed cell lines relative to the levels in MCF10A at indicated loci. Data are the average (±dev.) of two independent experiments with triplicates used for qPCR. The statistical analysis is in Table 1.

RT-qPCR assays showed that the transition from MCF10A to the more aggressive downstream tumor cell populations resulted in only minor increases in the global expression of CD44 (Figure 9A). In contrast, the inclusion of variant CD44 exons was drastically increased (Figure 9B, left panel), and symmetrically, the levels of transcripts skipping all the variant exons were decreased (Figure 9B, right panel). This modified processing of the CD44 pre-mRNA was consistent with earlier observations from tumors in vivo (104,108–110). It was initiated upon transformation of the MCF10A by RAS(T-24), and then persisted during the subsequent steps of tumor progression. For meDNA, the overall pattern in the parent MCF10A cells was very similar to that observed in HeLa cells (compare Figure 9C to Figure 4C). In contrast, in the derived tumor cell lines, the levels of meDNA were increased at several locations inside the body of the CD44 gene as determined by MeDIP (Figure 9D). These changes in meDNA occurred mostly outside highly methylated regions, the main contributors being variant exons and their intervening introns (Table 1). Thus, our results, obtained with four independent cellular models, suggest that intragenic meDNA is predictive of the outcome of CD44 alternative splicing.

**Table 1.** Statistical analysis of the differential levels of DNA methylation within the CD44 gene among the MCF10A-derived cells analysed in Figure 9D. Indicated Groups of loci for comparison were tested by pairwise Student's t test (two-tailed) evaluating differences from locus to locus. The *P*-values of these tests for each cell line compared to MCF10A parental cells or CA1A versus DCIS are reported in the Table. The non-significant *P*-values are in italic.

	Comparison versus MCF10A			versus DCIS
	MCF10A(Ras)	DCIS	CA1A	CA1A
Differences on:				
all loci tested	1.4E-07	2.8E-07	5.7E-04	4.3E-05
constant exons	0.001	*0.107*	*0.127*	*0.298*
variant region inluding introns (i10, i11, i15)	1.1E-04	5.0E-04	0.006	0.004
variant exons only	0.003	0.005	0.029	0.012
introns only	0.035	*0.050*	0.043	*0.068*

## DISCUSSION

In this paper we sought a relationship between the dysregulation of alternative splicing and the modified meDNA frequently observed during tumorigenesis. For the CD44 gene, we observed a very general correlation between intragenic meDNA levels and the inclusion of alternative exons in four different model systems (Figures 1, 4, 7 and 9).

At this gene, locally increased DNA methylation engineered with the dCas9/DNMT3b construct functioned as a switch promoting variable exon inclusion in cells devoid of DNA methylation (Figure 6). To our knowledge, this is the first time this approach has been used to demonstrate how local DNA methylation can participate in the alternative splicing regulation of an endogenous gene. The successful outcome of this approach was likely to rely largely on the use of DKO cells lacking DNA methylation, exacerbating the difference between methylated and unmethylated DNA, even in regions relatively poor in CpGs. The experiments also provide some insight on the mechanisms connecting DNA methylation to alternative splicing. For example, we noted that methylation targeted to v4 modified inclusion levels of several downstream exons, but not of v4 itself. This strongly suggested that the epigenetic information was relayed from the transcription to the splicing machinery. In particular, a slowing-down of transcriptional elongation may mechanically modify the relative availability of downstream splice sites during co-transcriptional splicing. Alternatively, methylation marks may nucleate the recruitment of chromatin readers such as HP1ɣ or MBD proteins, that may then propagate the local chromatin state while also recruiting splicing factors to the affected region (Figures 3 and 5). The particular organization of the CD44 gene containing 9 adjacent variant exons may be particularly sensitive to these mechanisms of signal transmission, but the phenomenon could also be at important for other genes. In this context, we noted that several DMRs (in DKO cells and in ALL tumors) were not located in the immediate neighborhood of variant exons (Figure 8E, F,G). Although we showed a local increase in 5mC by targeting the catalytic domain of DNMT3b on CD44 variant exons we cannot rule out that other unexplored sites near the targeted locus may have an influence on the fate of the splicing decision.

**Figure 10.**
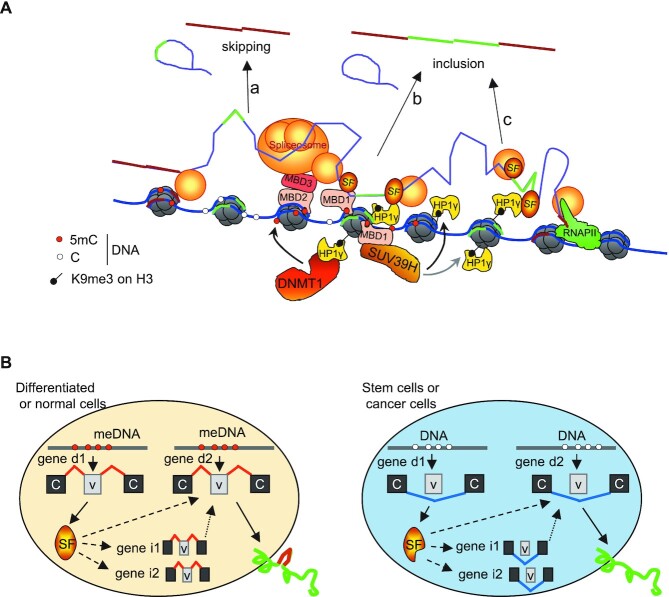
Models for the roles of DNA methylation in the modulation of alternative splicing. (**A**) Absence of DNMT1/3b decreases CpG methylation (white circle), inhibits MBD recruitment, prevents the HP1γ spreading on the gene body, and favors the skipping of variant exons (A). DNA methylation (5mC) can be recognized by MBD proteins that may help regulate RNA alternative splicing by promoting S5p-mediated RNAPII pausing, by interacting with the spliceosome and/or splicing factors (SF), or by promoting HP1γ recruitment on chromatin via H3K9me3 provided by SUV39H1 (B). In the absence of CpG, high levels of H3K9me3 still favor the local recruitment of HP1γ (**C**) without counteracting the impact of the 5mC defect on splicing. (**B**) At directly impacted genes (gene d), some splicing factors, such as TRA2Aa/b, might act as a DNA methylation sensor by expressing specific isoforms which promote alternative splicing decision on genes indirectly regulated (genes i) and also on genes directly regulated (genes d) by DNA methylation. This last situation creates a positive loop reinforcing the modulation of alternative splicing by local 5mC levels.

Thus, further studies will be needed to determine how other intragenic regions (in introns for example) may also change the alternative splicing of genes. It may also be considered that these experiments actually indicate on the amplitude of the splicing modifications that can be expected from variations in local meDNA levels. Thus, while our study provides clear evidence for a direct effect of meDNA on splicing, it also highlights the importance of considering the contribution of indirect effects when interpreting splicing variations in samples showing modified DNA methylation (Figures 1, 2 and 10).

### How meDNA can directly modulate the alternative splicing

We showed that the magnitude of regulation of alternative splicing events by certain splicing factors also depended on intragenic DNA methylation (Figure 5C). Remarkably, this splicing factor dependence (at least for SAM68) does not appear to occur for meDNA-insensitive events as is the case for the CCND1 and PKM genes in DKO cells (Figure 5D). We note that we did not find, in HCT116 cells, the sensitivity to DNA methylation for the PKM gene which has been described previously as dependent on the CTCFL factor (39), or that we found in ALL patients (Supplementary Table S4), but this may be due to differences in cell type.

Several mechanisms have been proposed to explain a direct connection between meDNA and alternative splicing, the best documented being the implication of methyl-binding proteins (MBDs). In this context, two factors, namely CTCF and MeCP2, have been examined in depth (21,36,38,40,111). In the case of CD44 alternative splicing, CTCF is unlikely to be involved since we did not observe binding sites or recruitment in or near the CD44 variant region in either of the cell lines we examined, including HCT116, DKO (ChIP-seq Supplementary Figure S3), and HeLa cells (ChIP-seq from ENCODE). In addition, we observed that reduced meDNA resulted in reduced inclusion of CD44 variant exons, while a roadblock mediated by CTCF recruitment after demethylation of the DNA would be expected to increase inclusion of these exons.

For MeCP2, we found that depletion of this protein did not have a clear inhibitory effect on CD44 variant exons in HeLa cells (Figure 5B). This seems inconsistent with earlier observations in MeCP2 mutant mice where CD44 is aberrantly spliced in cerebral cortex mRNA without requiring binding to meDNA (111). These differences may be due to the template, which in our study is the endogenous gene, or they may depend on neuron-specific factors absent in HeLa cells.

The role of other MBDs in splicing has not yet been characterized and we showed here for the first time that several MBD family members are at the interface between methylated DNA and splicing/chromatin factors. In particular, MBD1 depletion resulted in a profound inhibition of CD44 variant exon inclusion (Figure 5B), while its recruitment to the CD44 gene body depended on the presence of meDNA (Figure 3E). We noted that MBD1 recruitment did not match the level of 5mC on individual loci detected in HeLa cells by MeDIP (Figure 4C). These differences may be due to cell type variations, but also to differences in chromatin-shearing efficiencies between ChIP and MeDIP assays. Alternatively, the enrichment of MBD1 at a particular locus may not be well correlated to the density in methylated CpG.

Like for MBD1, the depletion of MBD2 also had an important negative effect on CD44 variant exon inclusion in HeLa cells (Figure 5B) and the depletion of both MBD1 and MBD2 resulted in decreased variant exon inclusion in HCT116 cells but not in DKO cells lacking meDNA (Figure 5C). This indicates that the effects of MBD on alternative splicing modulation is dependent on DNA methylation. Interestingly, MBD2 has also been detected on methylated exons/gene bodies of active genes and was thought to modulate RNAPII pausing (112). Thus, reduced levels of MBD2 on the CD44 gene body are expected to alleviate potential RNAPII pausing, a process that would favor the observed skipping of CD44 variant exons (31,76). In accordance with this expectation, we found that the reduced level of 5mC in DKO cells correlated with decreased levels of S5p-CTD RNAPII in the variant CD44 region, while the levels of S2p-CTD RNAPII were essentially unchanged (Figure 3AB). This suggests that the elongating RNAPII speeds up or pauses less within the variant region. This reduction in S5p-CTD RNAPII could also have a negative effect on the recruitment of splicing factors, or on the use of splice sites by spliceosome as shown in the study of RNAPII-related nascent RNAs (113).

MDB3 does not bind preferentially to methylated DNA (114), but is recruited via MBD2. It is therefore enriched on exons of active genes (41,115). MBD3 also copurifies with an in vivo assembled U2 snRNP spliceosomal complex (116). This suggests that MBD3 recruitment to gene bodies may favor the spliceosome recruitment to spliced exons or instead be dependent in part on splicing decisions, in a way similar to that shown for H3K36me3 methyltransferase SETD2 (26).

### Relationship between meDNA, H3K9me3 and HP1γ

Several studies have suggested that MBD1-mediated transcriptional repression relies on the recruitment of HP1α and histone methylases, including SUV39H1 (117) and SETDB1 (118). Remarkably, the distribution of H3K9me3 within CD44 in HCT116 cells (Figure 3C) was similar to that observed in HeLa cells (31,32), i.e. with the higher enrichment covering the end of the region containing the variant exons (v8, v9 and v10). This pattern is different from that observed for 5mC within CD44, as variant exons v9 and v10 are particularly poor in CpGs, and therefore in 5mC (Supplementary Figure S4C, Figures 4C, D and 7C). This alone is evidence for the absence of a positive correlation between H3K9me3 and meDNA. Earlier studies have suggested that H3K9me3 may compensate for 5mC in gene body regions with low CpG density (7,34). The same mechanism of compensation may be at play in the body of genes for the regulation of alternative splicing.

The inverse correlation between the levels of H3K9me3 and CpG density also argues against the implication of histone methylation marks in the guiding DNA methylases to the coding region of CD44. However, we found that the level of H3K9me3 within the endogenous chromatin of CD44 variable exons was decreased in the absence of meDNA in DKO cells (Figure 3C). Accordingly, the reintroduction of exogenous either methylated or unmethylated DNA templates into the chromatin has revealed a dependency of meDNA on H3K9me3 (33,119). We noted that even when the levels of H3K9me3 were reduced by DNMT inactivation, the accumulation of this histone mark remained higher in the variant region than in the constant regions. This is consistent with the relative stability previously reported for pericentromeric H3K9me3 in DNMT null cells (29). In this context we have shown earlier that in CD44, H3K9me3 deposition at the end of the variant region is guided by Argonaute-dependent chromatin associated complexes (32), and we noted that the expression of AGO1 is increased in the DKO cells (Figure 2B).

Although little evidence exists for a connection between HP1γ recruitment and meDNA in the regulation of splicing (33), this link may be more complex than anticipated. Indeed, in HCT116 cells, we observed that a fraction of HP1γ on the chromatin of the CD44 gene body was dependent on the presence of DNA methylation (Figure 3D), but independent of the level of H3K9 methylation, especially where the H3K9me3 level is low and unmodified in DKO cells. For most of the CD44 gene body, it seems that in the DKO cells, the loss of 5mC renders HP1γ accumulation independent of H3K9me3, since it declined to a greater extent than the decrease in H3K9me3. Seemingly, the presence of HP1γ at places with low H3K9me3-levels depended on the presence of MBDs and on a spreading mechanism over competent chromatin, i.e., with methylated DNA. A nucleus of HP1γ located on v9 v10, where H3K9me3 is at a peak, would be the starting point of this recruitment, in accordance with an RNAi mechanism (32). The amount of HP1γ remaining on the chromatin in DKO cells is large enough to permit the influence of the HP1γ knockdown on the inclusion of variant exons, which is not the case for MBD1 and 2 proteins (Figure 5C).

Taken together, these data suggest that the recruitment of HP1γ to the CD44 gene body may be dependent on MBD1 (and possibly on MBD2/3) in CpG-containing region, while it relies on H3K9me3 in regions with low CpG density (i.e. v9 and v10) (Figure 10A). This shows that HP1 and MBD could work together to modulate alternative splicing decisions, potentially via the elongation of RNAPII or recruitment of splicing factors. These data also suggest that meDNA is not required to cover exactly the sensitive variant exon since a certain degree of spreading of the chromatin readers is achieved via the regulation of RNAPII kinetic or protein-protein interactions.

### Discriminating between the direct and indirect effects of meDNA on the modulation of alternative splicing

We observed that a loss of meDNA in somatic human cells is associated with extensive changes in alternative splicing. However, the analysis of gene expression that we carried out in parallel suggested that the meDNA-splicing correlation may be largely rooted in a complex combination of indirect effects due to the modified expression or splicing of splicing regulators (Figure 2A) and transcriptional regulators (Supplementary Table S2A).

Thus, by documenting the modified expression of ESRP1 and other factors upon loss of DNA methylation, we provided evidence for the involvement of splicing factors and transcriptional regulators in the splicing modifications observed in DKO cells. Therefore, regulatory loops linking the expression of splicing/chromatin factors to the overall chromatin state may also contribute to splicing dysregulations imputed to local chromatin changes. Notably, there was a very good correlation between the inclusion of CD44 variant exons and the local levels of meDNA in three different cell types : HCT116/DKO, DNMT1-depleted HeLa cells and MCF10A-derived breast tumor cells.

As short-term depletion of DNMT1 in HeLa cells had little effect on the transcriptome, including no detectable change in the expression of splicing factors, it offered a good opportunity to explore the direct effect of meDNA on alternative splicing. Depletion of this DNA methylase modified splicing at only a limited number of genes. Interestingly, several of these genes were highly relevant for cancer and EMT (Supplementary Figure S4G). These genes included CD44 and DST, which are required for anchoring intermediate filaments to hemidesmosomes in epithelial cells and are involved in human metastatic processes; kinesin KIF1B (120), Glutaminase GLS (121), and GNAS, a stimulatory G-protein alpha subunit (G s-α) that is mutated in colon cancer and associated with familial nonmedullary thyroid cancer(122).

In the ALL-related changes in meDNA in gene bodies, a decrease was observed, as reported in (61). This hypomethylation of gene bodies is thought to be a consequence of passive dilution during the uncontrolled cell division in tumor transformation (44). This has consequences not only for the pool of expressed genes but also for the quality of the mature transcripts since more than two thousand genes were found to be differentially spliced in these leukemias (Supplementary Figure S6C). DNMT1/3b depletion in HCT116 cells to some extent mimics this phenomenon and allows us to identify the few alternative splicing events that can be linked to a decrease in meDNA. Since these splicing events occur in very different cell-types with different developmental origins, they are likely to be a consequence of changes in meDNA. Differentially spliced genes detected in both DKO cells and in ALL cells within regions where meDNA was perturbed, showed strong enrichment in genes that participate in pre-mRNA binding, GTPase regulatory networks, and cell adhesion processes (Figure 8B). This indicates that the meDNA status of cells is linked to the activity of the RNA processing machinery, GTPase regulators and the cell–cell/cell–ECM interactions through splicing modifications. This is potentially important, as it suggests that the genes subject to splicing perturbations in cancers are also those participating in cellular interactions within the tumor niche (123,124)

### Genes subject to meDNA-dependent splicing in different cells share common molecular functions

An important conclusion from this study is that DNA methylation, which at best was considered a way of fine-tuning alternative splicing may, for a small number of genes, have an impact such that the level of meDNA present inside the body of these genes can have predictive value when estimating the splicing isoforms produced in a given cell type. This is further supported by a recent study showing that splicing variations at the single-cell level can be accurately predicted based on local DNA methylation (125).

Another possibility, and perhaps a more interesting one, is that these predictable genes may serve as meDNA sensors to adapt gene expression to external cues (Figure 10B). In other words, some RNA binding proteins/splicing factors such as PRPF38B, TRA2B, and TRA2A may translate changes in meDNA into modified activities of the splicing machinery by changing their exon composition. A similar mechanism may apply for other pathways/cellular functions, such as metabolic enzymes (e.g. GTPase activators) or membrane proteins (e.g., CD44, CD47, APLP2, NISCH). Remarkably, TRA2A/B splicing factors have been described as binding factors of MeCP2 (36,40). Further investigations will be required to determine whether/how specific isoforms of TRA2A or TRA2B can bind differentially to MeCP2 for instance, and mediate particular alternative splicing decisions. The question of the potential differential activity of TRA2B isoforms on the CD44 alternative splicing will also require further investigation.

In conclusion, we discovered a causative role of meDNA in the regulation of alternative splicing via the factors MBD1, MBD2 and MBD3. Our findings reveal that most variations in alternative splicing are consequences of indirect effects. However, careful examination of splicing and intragenic methylation in two different cellular contexts also suggests that a limited number of splicing factors are directly affected by intragenic meDNA and may therefore function as mediators of some of the indirect effects. These splicing factors may also contribute to the splicing regulation of genes directly affected by intragenic 5mC, thus creating a potential positive loop of regulation between chromatin modifications and splicing factor expression. In summary, we propose that the relationships between DNA methylation and alternative splicing decisions are an integration of multiple intricate and subtle mechanisms. Additionally, some genes including CD44 are directly sensitive to intragenic 5mC levels. In this context, the variation in intragenic meDNA occurring during tumorigenesis may be predictive of alternative splicing outcomes of these genes.

## Supplementary Material

gkab437_Supplemental_Files

## FUNDINGS

Ministry of Research (MENRT; to O.M.); ERASMUS program and University (to B.H. and C.H.L.); Centre National de Recherche Scientifique (CNRS; to E.B. and C.M.); Agence Nationale de la Recherche [ANR-11-BSV8-0013]; REVIVE––Investissement d’Avenir (to J.Y., C.M., O.M. and E. K.). Funding for open access charge: CNRS recurrent funding; J.Y. is part of the Pasteur - Paris University (PPU) International PhD Program; European Union's Horizon 2020 research and innovation programme under the Marie Sklodowska-Curie [665807].
