# Supplementary material for: CD44 alternative splicing senses intragenic DNA methylation in tumors via direct and indirect mechanisms
Source: Nucleic Acids Res. 2021 Jun 4;49(11):6213–37. doi: 10.1093/nar/gkab437 (PMC8216461; doi:10.1093/nar/gkab437)
Supplement: gkab437_Supplemental_Files [file gkab437_supplemental_files.zip › Batsche_Sup Figs S1-S7_NARv3_2021.pdf]

• Batsché et al. Suplementary figures

<u>Supplementary Figure S1 : Double knock-out of DNMT1 and DNMT3b in HCT116 cells promotes epithelial-to-mesenchym transition (EMT).</u>

A) Dot blot of methylated DNA from HCT116 WT or DKO using 3D33 antibodies directed against meDNA. Methyl blue staining of the blot shows the DNA loading.

B) RT-qPCR of RNA from HCT116 WT or DKO showing reference genes in these cell series

C) Four studies-based meta-analysis of gene differential expression between HCT116 WT and HCT116 DKO (knock-out of DNMT1 and DNMT3b). Fastq files of indicated GEO data were aligned on the hg19 genome using the STAR software. Multimapper reads or reads with more than one mismatch were not considered, Software detection indicates that data were not oriented except for Schrijver2012 (Schrijver et al., 2013) . The data from Maunakea et al. (Maunakea et al., 2013) were considered as not oriented even though software is unable to determine it. The number of reads uniquely aligned on hg19 are indicated in brackets for each dataset.

D) Test of normalization methods. After normalization the DKO and WT samples were tested for significant differences by using a paired T test (two-tailed) on the 5 comparisons as depicted in the table in **C**). The average of duplicate from the Blatter's study has been considered as one sample, the dev. is indicated in the error bar in **E**). The limit of change has been considered significant when the mean of the fold change is >1.5 and the p-value of the paired-T-test on the log2(cpm) is <0.05. The log2 normalization allowed to detect a more important fraction of upregulated genes compared to the « Rlog » normalization (proceeded with DESeq2) used as reference. The union of the two normalization methods predicted more differentially expressed genes than the individual tests.

E) Normalized RNA level (count per millions of reads in librairies) of reference genes (DNMTs and H19). Expression of Vimentin (VIM), Desmoplakin (DSP) two markers of EMT and CD44 were shown to complete the **Figure 1**. RPLP0 is shown as a non-modified gene. The statistical paired T-test (two tail) was used to calculate the p-value on the log2 normalized counts and indicated when <0.05 (n.s., non significant).

F) Differentially spliced genes in RNA-seq of DKO cells compared to the parental HCT116 cells. In order to take into account the statistical dispersion of the data due to the differences of RNA extraction methods, three different comparisons as indicated have been conducted using MAJIQ algorithm. "Local Splicing Variation" (LSV) estimated by MAJIQ were used to detect differentially spliced genes with high confidence between the conditions P(|dPSI|>0.2)>0.95, (Vaquero-Garcia et al., 2016) where dPSI is the "differential Percent of Splicing Index". The number of predicted genes having at least one alternative splicing event is indicated for each comparison. Finally, all the genes differentially spliced with high confidence in each comparison were considered.

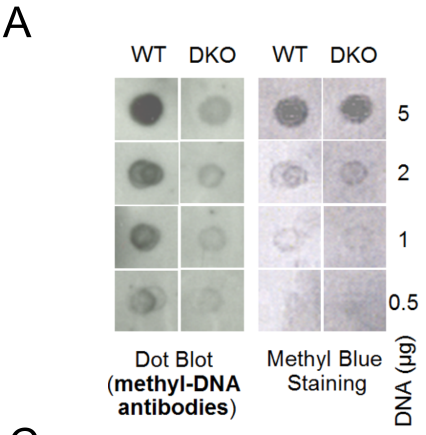

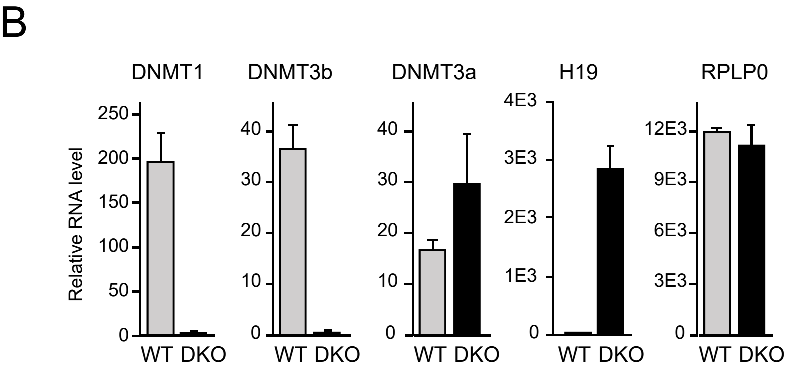

C

RNA-Seq from :	RNA extract :	WT	DKO
Simmer et al., 2012 GSE39068	polyA+ ●	SRR517830 (19 095 262)	SRR517831 (20 651 768)
Schrijver et al., 2013 GSE45332	polyA+ △	SRR787303 (30 609 125)	SRR787305 (29 480 692)
	total ■	SRR787296 (13 879 253)	SRR787304 (15 741 044)
Maunakea et al., 2013 GSE47488	total +	SRR869304 (41 087 539)	SRR869305 (43 391 672)
Blattler et al., 2014 GSE60106	total ◇	SRR1030462 (36 281 078)	SRR1536578 (34 864 087) average
		SRR1030463 (37 769 873)	SRR1536577 (33 287 125) average

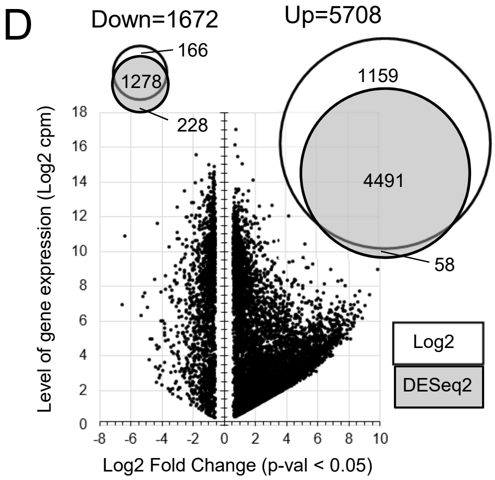

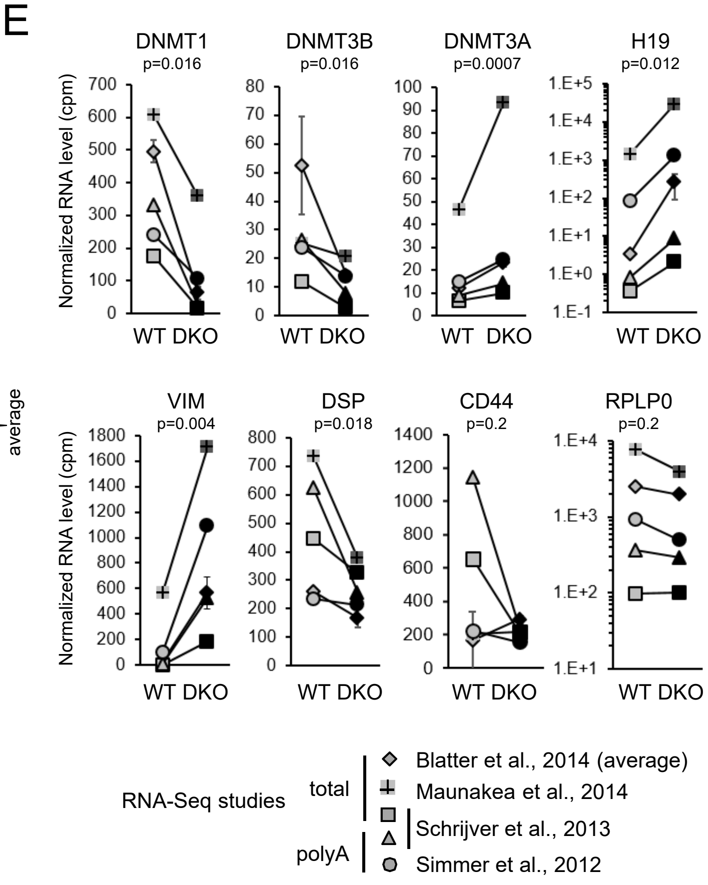

F

RNA extracts	DKO vs WT	Genes
Total RNA	4 vs 4	387
polyA RNA	2 vs 2	298
All RNA	6 vs 6	217
Combinaison of all comparisons :		653

Batsché *et al.,* Suplementary figure S1

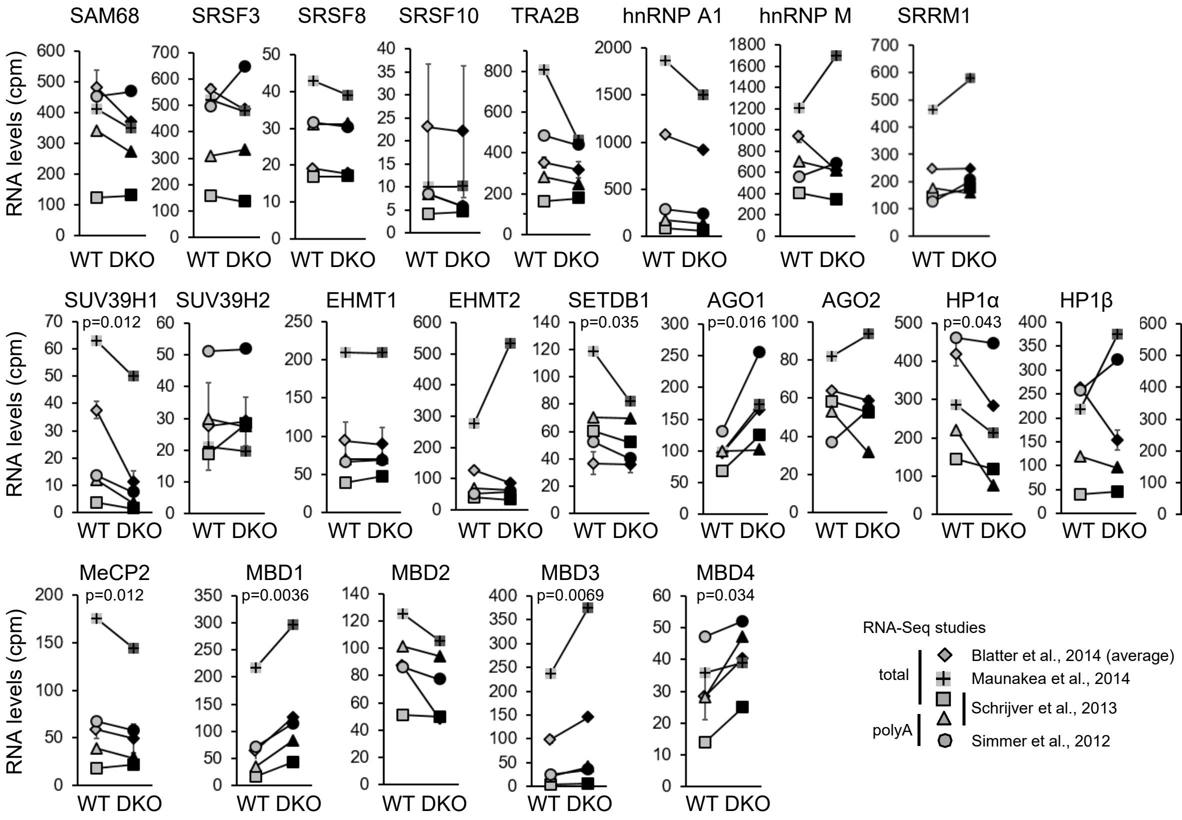

Supplementary Figure S2 : Meta-analysis of gene expression in HCT116 DKO cells

Normalized RNA level (count per millions of reads in libraries) of indicated genes confirming the RT-qPCR of the **Fig. 2B** and completing the **Fig. 1B** and the **Sup. Fig. S1E**. The statistical paired T-test (two tail) was used to calculate the p-value on the log2 normalized counts which are indicated only when <0.05 and the average of the fold change was >1.5.

Batsché *et al.*, Suplementary Figure S2

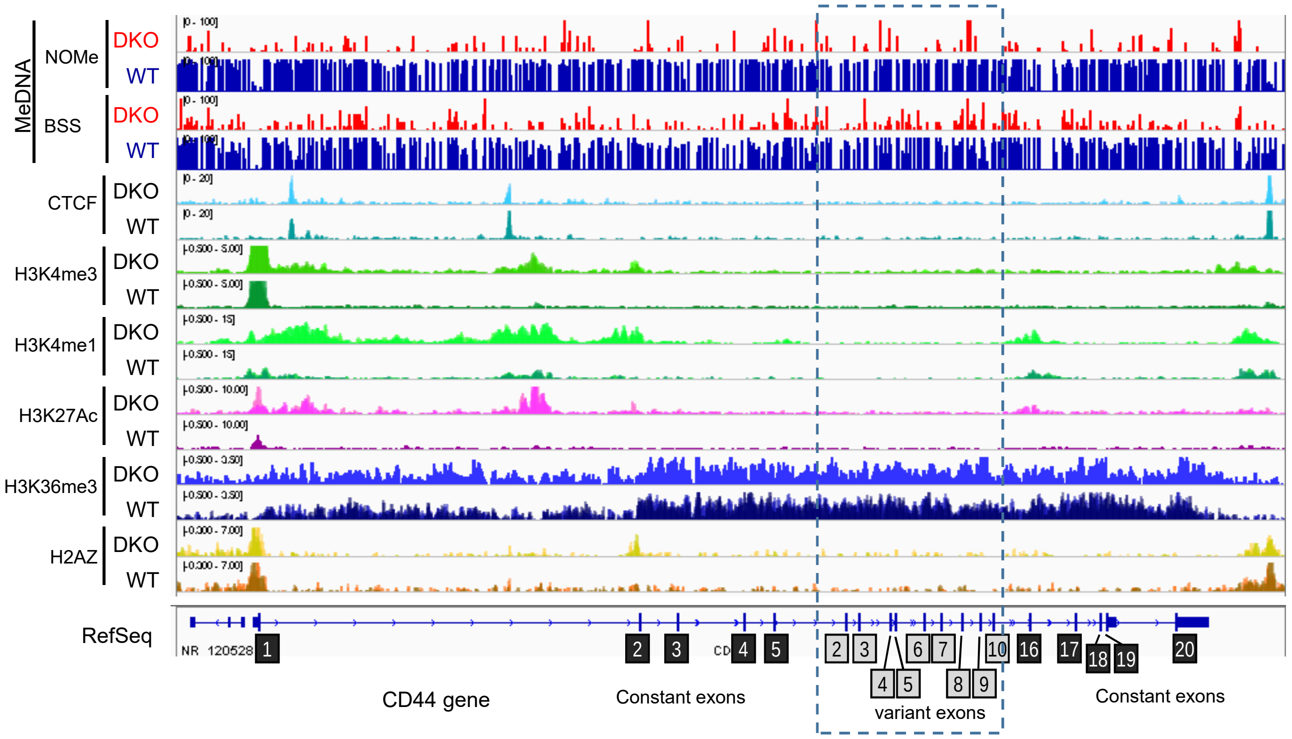

Supplementary Figure S3 : Distribution of epigenetic marks covering the CD44 gene
IGV view of DNA methylation from Nucleosome Occupancy and Methylome Sequencing (MOMe-Seq, MOMe) and Bisulfite-seq (BSS), and of ChIP-seq for the indicated histone marks (Lay et al., 2015) and CTCF (Maurano et al., 2015) covering CD44 in DKO and in WT HCT116 cells. Tracks were set at the same range for DKO and WT and are showed with HCT116 tracks below the DKO tracks for each indicated feature. The region containing the variant exons is indicated by the dotted line.
Duplicated ChIP-Seq were overlaid on one track. The used datasets are listed here:
.

	WT		DKO	
BigWig files used from GSE58638 (files around 2 Go) (Lay et al., 2015)				
H3K27Ac		GSM1415876	GSM1420161	GSM1415884
H3K4me1	GSM1420154	GSM1415875	GSM1420160	GSM1415883
H3K4me3	GSM1420153	GSM1415874	GSM1420159	GSM1415882
H2AZ	GSM1420152	GSM1415873	GSM1420158	GSM1415881
H3K36me3	GSM1420157	GSM1415879		GSM1415887
NOMe-Seq HCG (250 Mo)	GSM1420150		GSM1420151	
BigWig files used from GSE58695 (files around 250 Mo)				
Bisulfite-Seq (BSS) HCG	GSM1416976		GSM1416977	
BigWig files used from GSE50610 (files around 45 Mo) (Maurano et al., 2015)				
CTCF	GSM1224649	GSM1224650	GSM1224654	GSM1224655

Batsché *et al.*, Suplementary Figure S3

Supplementary Figure S4 : DNA methylation effects on alternative splicing in HeLa cells

A, B, D) Knock-down efficiencies of DNMTs in HeLa cells. Transfection of HeLa with the indicated siRNA targeting DNMT1 were used to extract total RNAs or proteins. **A, D**) RNAs were extracted and subjected to RT-qPCR to quantify the RNA levels of DNMT genes. Relative RNA levels were expressed as fold change over the mock control. Non-targeting (NT) and GAPDH siRNA are used as negative controls. The right panel shows the relative levels of RNA corresponding to the imprinted H19 gene expressed as percent of RPLP0 reference gene. The two bars correspond to different primer pairs amplifying two separated regions of the transcripts.

B) Prior to extraction, cells were treated with PMA for 2h and DNMT1 and histone H3 proteins were revealed by western blot.

C) The proportion of CpG in the indicated loci ±200 bp surrounding the displayed qPCR amplicons in the MeDIP assays. CpG were counted on both DNA strands and expressed as percent of total dinucleotides.

E) Relative levels of CD44 variant exons are decreased upon depletion of DNMT1 but not of DNMT3A or DNMT3B. Levels of each indicated exons were normalized by the average levels between mock and NT siRNA of corresponding exons. Data are average (± dev.) of at least three independent experiments. Statistical comparison have been were evaluated using Student's t-test (two-tailed), with $p<0.05$(*), $p<0.01$(**) , $p<1E-3$(***).

F) Transcriptome-wide analysis of DNMT1 depleted HeLa cells by Affymetrix exon arrays. In three independent assays, transfected HeLa cells by the indicated siRNAs targeting DNMT1 and by GAPDH siRNA used as negative controls during 5 days have been extracted. Total RNA extracts were used on exon arrays. Gene expression levels have been calculated by the means of the triplicate on all the exons after normalization (analysis conducted by GenoSplice). The differentially spliced genes were detected by calculating for each exon a splicing index (the exon level versus the gene level) and comparing them in DNMT siRNAs versus GAPDH siRNA. The genes were considered differentially expressed or spliced with a fold change >1.5 and p-value <0.05.

G) List of the differentially spliced genes found with the two different DNMT1 siRNAs. Genosplice analysis of exon arrays calculated average signal of the 4 probes (at least 4) for each exon on the triplicates. These levels were compared to the average signal of corresponding genes to evaluate their Splicing Index (PSI). Exon changes were considered significant with a fold change>1.2 and P-value <0.05 for each individual siRNA. Visual curation using Genosplice interface allowed selecting RNA events common for the two siRNAs. A comment indicates whether there is or not a CpG-rich region in the vicinity.

H) Exon array covering CD44, GLS and DST, according GenoSplice visualization. Each bar corresponds to one probe, and four probes were used for each exon; Bar height (log 2 scale) corresponds to signal intensity for the indicated condition and colors correspond to the change ratio of the GAPDH siRNA control: up-regulated in red, down-regulated in green, and unmodified in black. The changes of RNA after PMA-treatment of the cells were shown as positive control of variant exon increasing for CD44. Background signal is indicated by the yellow line. Exon numbers are indicated below, in grey rectangles. Maps of GLS and DST genes are showed indicating the annotated alternative splicing event (red lines), alternative promoters (red arrows) and alternative termination (noted "pA"). 3'UTR and 5'UTR are symbolized by red boxes in the maps and the thinner grey boxes below the probes.

.

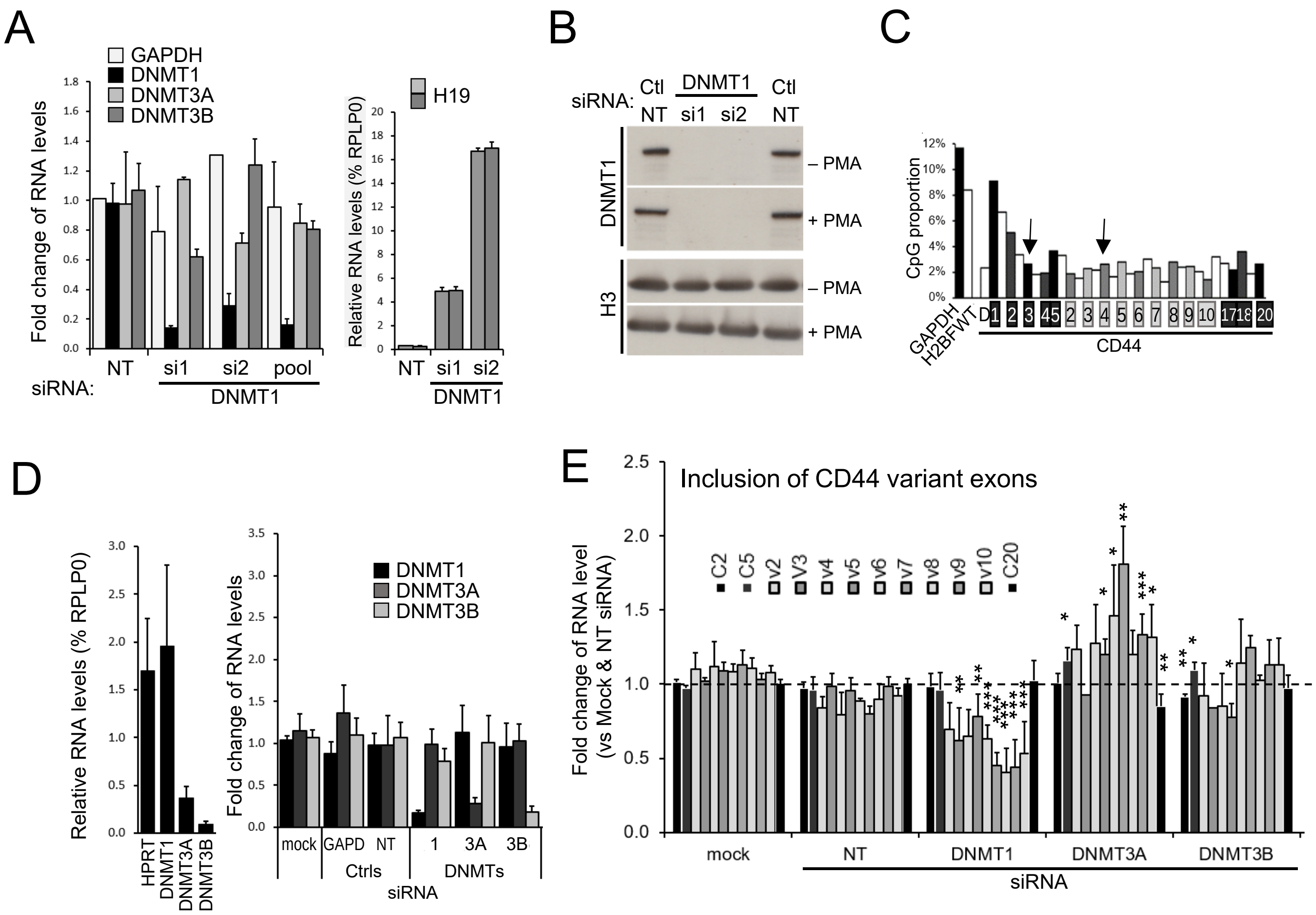

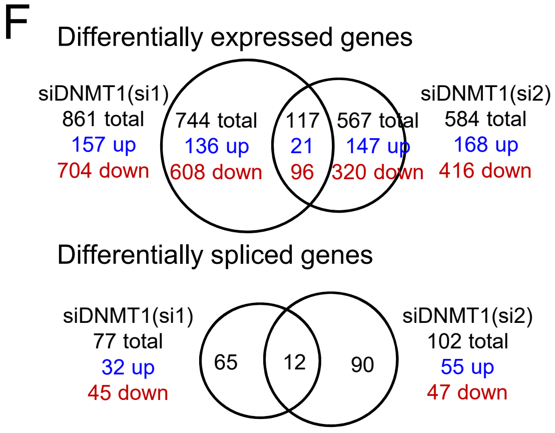

G

Commonly differentially spliced genes in both the 2 DNMT1 siRNAs after manual curation

AHRR e15 up (cassette exon) _ meCpG
DST ae39, e40 & e41/45 down (alt. Termination)_meCpG
GLS1 e18 to e20 up _ meCpG _ same splicing than HP1 (31)
KIF1B e28 to e54 up (e31=alt prom/end)_meCpG, e29 close CTCF paek
RABGAP1L e30 down / e32 to e37 up (e32=alt prom)_ CpG
CDRT1/TRIM16 ae6 to ae10 up (gene23ex)=alt start & end_ no CpG rich
GNAS e5e6 up / e9 to ae10 down (alt promoter ?)
CD44 e6 to e14 down (cassette exons) – no CpG rich

More strongly in one siRNA than in another
NOTCH2NL e4e5e6 down / gene up
TXNIP ae2 down / gene up
MRPL11 alt prom e2 up / gene down
DAB1 e10e11 down / gene up

Batsché *et al.*, Suplementary figure S4 part1/2

H

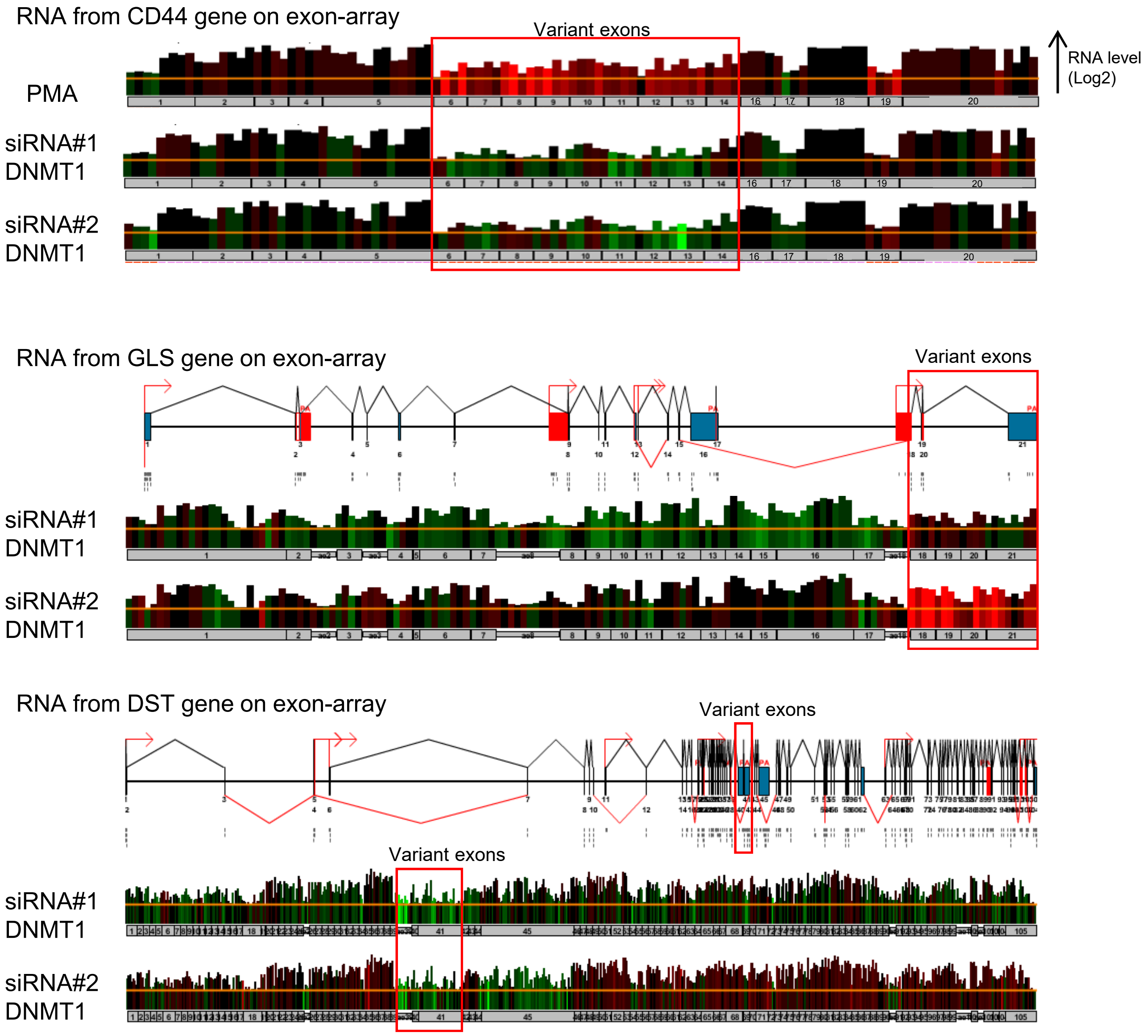

Batsché *et al.*, Suplementary figure S4 part2/2

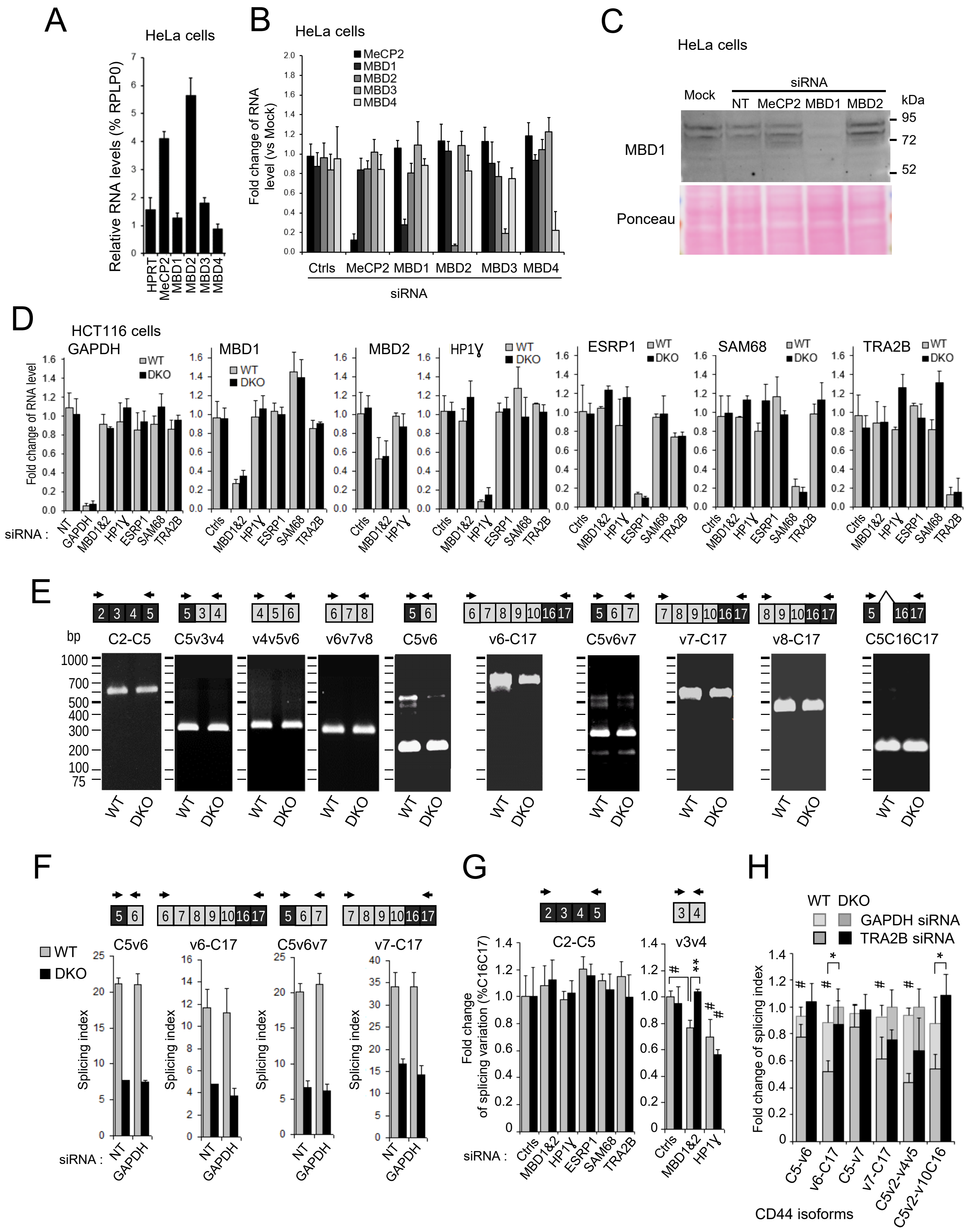

Batsché *et al.*, Suplementary figure S5

Supplementary Figure S5 : knocking-down of MBD genes affect the CD44 alternative splicing in HeLa and HCT116 cells

A) RNA levels of indicated genes in HeLa cells. RT-qPCR relative levels are expressed in percent of RPLP0.

B, C, D) Knock-down efficiency of indicated siRNA in HeLa and HCT116 cells. Three days after transfection, cells were extracted and the RNA levels were evaluated by RT-qPCR (**B, D**). Relative levels for each cell type are expressed relative to the mock-transfected transfected cells (set to 1). Ctrl siRNAs are the mean of non-targeting and GAPDH. The data are averages (±dev.) of at least three independent experiments. **C**) MBD1 protein level in HeLa cells were evaluated by Western blot. The ponceau-stained membrane is shown as loading control.

E) Qualitative analysis of end-point PCR products by agarose gel electrophoresis. The majority of the PCR products, as identified by sequencing, correspond to the variant transcripts indicated on the top. Few alternative isoforms were detectable for C5-v6 and C5-v7 but never exceed 10% of the total amplicons. Note that the differences between WT and DKO are not quantitative because the PCRs were saturated.

F, G, H) Alternative splicing of CD44 by RT-qPCR in HCT116 WT or DKO cells transfected by siRNA controls. Variation of indicated isofom transcripts in transfected cells were measured by RT-qPCR using a long time for extension step. Variation were expressed in % of C16C17 constant exons for v3v4 and C2-C5, and as splicing index using C5-C17 as reference for the formula LSV = variant / (variant + C5-C17) * 100 for other CD44 variant forms in H. **G, H**) These data were then expressed for each cell-types as a fold change over the averages of control non-targeting (NT) and GAPDH siRNAs (noted Ctrls) shown in **F**). The data are averages (±dev.) of at least three independent experiments. Statistical significance of the isoform differential levels upon indicated depletion were indicated by hashtag (#) for comparison with GAPDH siRNA, or by asterix (*) for comparison between WT and DKO cells

Supplementary figure S6. Analysis of expression pattern of Acute Leukemia (ALL).

A) Principal component analysis of gene expression based on the 500 most variable genes evaluated by DESeq2 with Rlog normalization.

B) Proportion of reads aligning onto the hg19 human genome for each indicated library. The libraries with less than 60% of alignment were not considered for further analysis (white bars).

C) Differential gene expression between ALL and HCB cells evaluated by DESeq2 with a log2 fold change >1 and adjusted p-value <0.001 on 8 HCB and the 12 ALL samples which aligned equivalently onto the genome. Note that the genes from ChrX and ChrY were excluded from this analysis due to their absence in the SRR data of ALL samples from Almamun et al., (Almamun et al., 2015). 13244 genes were found expressed considering that their level exceeds the threshold defined at log2(cpm)=0.7 which correspond approx. to a minimum of 32 raw count per genes. Differential splicing of the genes evaluated by MAJIQ with high confidence P(|dPSI|>0.2)>0.95. The number of predicted genes having at least one alternative spliced event is indicated as well as those that are regulated downward (grey partition) or upward (black partition).

D) The CD44 expression level in each sample was calculated by averaging the reads covering the junction of constant exons. The data were presented as averages of exon-exon junctions (± dev.). To analyze the inclusion of CD44 variant exons **(Fig. 7A and 7B**), the scared samples were used including the 8 normal samples (HCB) and the 9 ALL samples showing comparable range of CD44 global expression. Significant differences with T-Student p<0.05 (one-tail, equal var.) of CD44 alternative splicing were still found if only the 5 ALL expressing the highly CD44 level in the range of HCB (i.e. A20, A30, A37, A31, A17) were considered for comparison with the HCB.

E) The relative RNA levels of the indicated genes in the cohort of ALL patient versus the control cells. The RNA expression of indicated genes were evaluated by the normalized count of reads (cpm) covering the exons of the 12 selected ALL (**Sup. fig. S6A**) versus the 8 HCB. The indicated *P* value were calculated using the Student's t-test (two-tails) on the Rlog transformed read counts. Fold change (FC) were calculated on the median. RPLP0 reference gene is shown.

F) The separated tracks corresponding to the MIRA-seq from 18 ALL (blue) and 20 pre-B HCB (red), set at the same range (0-30), which are used for the overlay in the **figure 7C**.

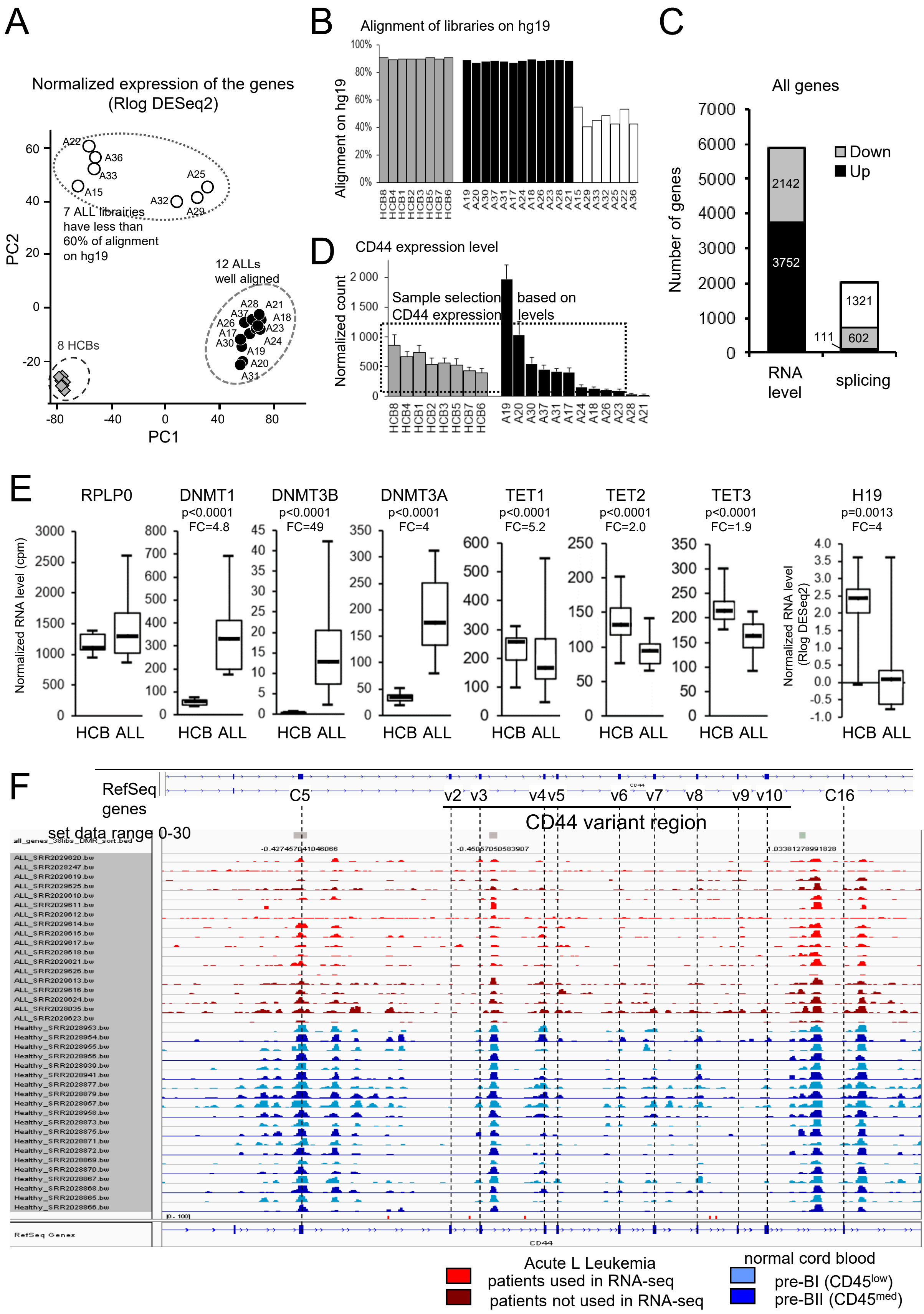

Batsché *et al.*, Suplementary figure S6

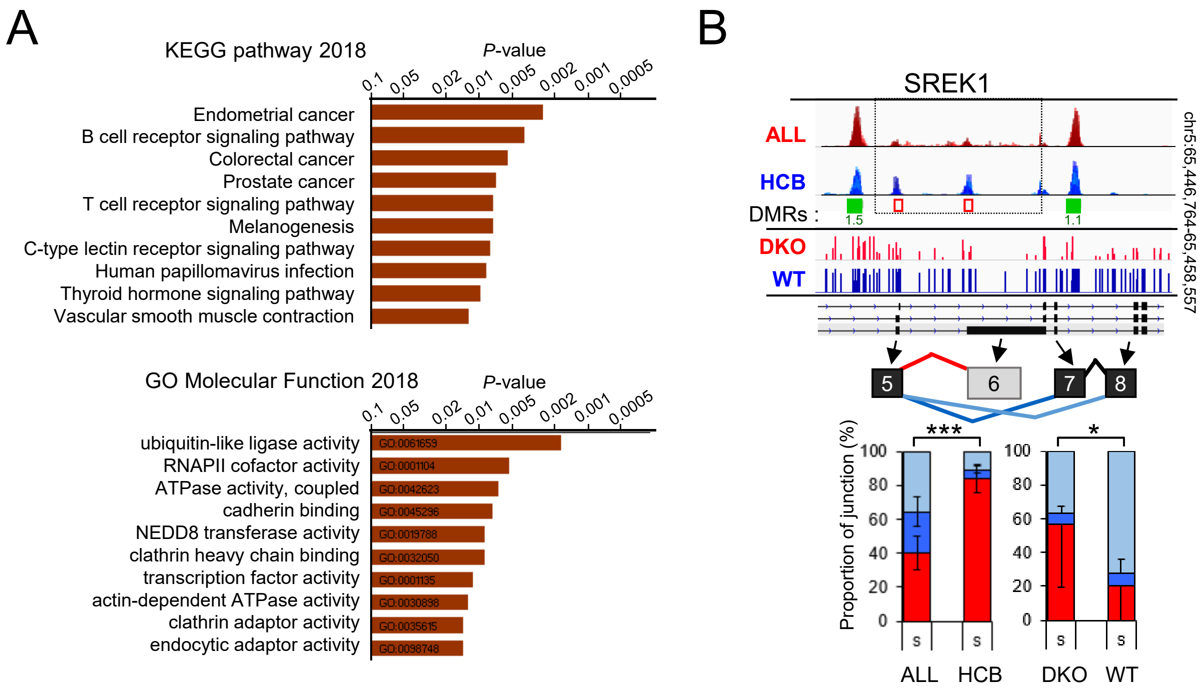

Supplementary figure S7. **pathway analysis of genes differentially spliced both in DKO and in ALL but not correlated with variation of local DNA methylation.**

A) Pathway analysis was performed using the Enrichr tool (Kuleshov et al., 2016) and the 26 genes with splice changes were not correlated with changes in DNA methylation (yellow, orange and khaki quarters of the **figure 8A**).

B). Example of gene corresponding to the categories with no correlation between splice changes and modification of meDNA. The data are presented as in the **figures 8D-G**

Batsché *et al.*, Table S2B

Differential expression and splicing of RNA-binding protein/splicing factors in DKO cells versus WT HCT116 cells. This table is a hub of Table S1 and Table S2A for splicing factors.

Regulated RNA binding factors

Ensembl ID	Gene	Parent (<br>eudog	RNA level<br>Log2 cpm	Differential RNA expression<br>Regulation	Log2 FC	pval	Differentially spliced<br>curation	LSV ID
ENSG00000109536	FRG1	p	4.8				CE8	190878553-190878657:S
ENSG00000107625	DDX50	p	5.6				CE3	70666467-70666763:T
ENSG00000126653	NSRP1	p	6.2				CE9…	28499560-28499616:T<br>:28443664-28443881:S
ENSG00000116001	TIA1		6.2				5'SS E10/Ee11	70443536-70443631:S
ENSG00000013441	CLK1		6.4				FE1 or FE6	201724403-201724469:S
ENSG00000198563	DDX39B		6.6				3'SS E6	31508099-31508441:S<br>31509727-31510225:T
ENSG00000117360	PRPF3	p	6.6				CE4	150300234-150300925:T
ENSG00000100296	THOC5		6.7				CE2+3 &<br>CE15	29924926-29925228:S<br>29949660-29950243:T
ENSG00000164548	TRA2A	p	7.4				CE4	:23571408-23571660:T
ENSG00000134186	PRPF38B		7.5				CE7	109240322-109241449:T<br>109238899-109238959:S
ENSG00000179950	PUF60		7.7				3'SS CE6	144911450-144912029:T
ENSG00000197111	PCBP2	p	7.7				5'SS FE	53835525-53835584:S
ENSG00000153914	SREK1		7.7				CE6 or TE	65449396-65449618:S
ENSG00000151923	TIAL1		7.9				3'SS e7	121347664-121347760:T<br>121336288-121336417:T
ENSG00000029363	BCLAF1	p	8.1				CE13+14	136590575-136591097:T
ENSG00000196504	PRPF40A		8.2				CE11	153533965-153533989:S<br>153535643-153535986:T
ENSG00000154473	BUB3	p	8.3				TE8 or TE9	124922128-124922757:S
ENSG00000145833	DDX46		8.3				5'SS e22	134152120-134152296:S
ENSG00000136527	TRA2B		8.4				FE1 or FE2	:185644389-185646861:S<br>:185655613-185655924:T
ENSG00000124193	SRSF6	p	8.6				CE7 or TE	42089343-42092245:T
ENSG00000135829	DHX9	p	8.6				CE4	182821368-182821479:T<br>182811680-182811812:S
ENSG00000160710	ADAR		8.9				CE4	154574861-154575102:S
ENSG00000168566	SNRNP48		5.6	down	-0.7	0.035	CE5a	7599906-7601757:T
ENSG00000060138	YBX3	p	7.7	down	-0.9	0.006	CE12+13	10856622-10857037:S
ENSG00000092847	AGO1		6.9	up	0.7	0.016	FE1 or FE2	36354028-36354211:T
ENSG00000100320	RBFOX2		6.8	down	-1.3	0.000		
ENSG00000152601	MBNL1		7.0	down	-1.3	0.018		
ENSG00000136231	IGF2BP3	p	5.8	down	-1.2	0.011		
ENSG00000099622	CIRBP		7.8	down	-1.1	0.004		
ENSG00000104413	ESRP1		6.6	down	-1.0	0.013		
ENSG00000065978	YBX1	p	7.5	down	-0.9	0.010		
ENSG00000148690	FRA10AC1		5.3	down	-0.9	0.002		
ENSG00000137944	CCBL2		4.5	down	-0.8	0.001		
ENSG00000056097	ZFR	p	7.1	down	-0.8	0.010		
ENSG00000117614	SYF2	p	6.0	down	-0.6	0.006		
ENSG00000100056	DGCR14		4.4	up	0.6	0.038		
ENSG00000123136	DDX39A	p	7.2	up	0.6	0.038		
ENSG00000169217	CD2BP2	p	6.1	up	0.7	0.011		
ENSG00000131043	AAR2		5.1	up	0.7	0.037		
ENSG00000071859	FAM50A		6.2	up	0.7	0.043		
ENSG00000126803	HSPA2		2.6	up	1.2	0.003		
ENSG00000204389	HSPA1A		2.4	up	3.8	0.010		
ENSG00000154548	SRSF12		-0.7	up	4.1	0.002		
ENSG00000185272	RBM11		-1.2	up	5.4	0.002		
ENSG00000128739	SNRPN	p	-1.6	up	5.4	0.001		

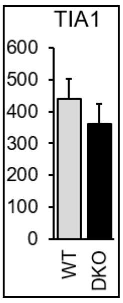

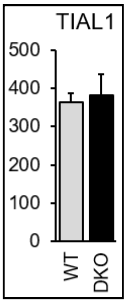

(MAJIQ) LSV Type | Regulation dPSI>20% [95%] <-More in DKO // More in WT->

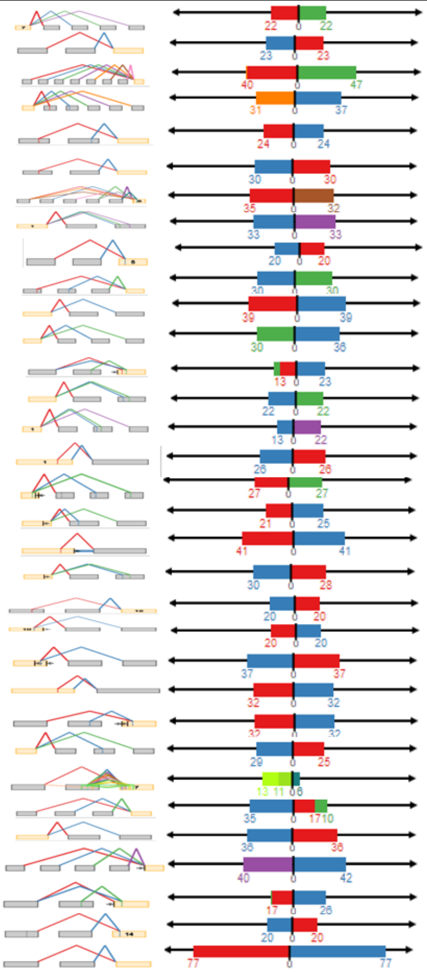

In the Manual curation column : CE=cassette exon, FE=First exon (promoter), TE=terminal exon (3'end), SS= splice site

In the LSV ID column: T=target, S=source

P indicated that these genes have been described as parent of pseudogene (Pei et al., 2012 in Genome Biol. 13,9,R51, The GENCODE pseudogene resource), which means that the splicing analysis can be misled by the expression of the retropseudogene or pseudogene.

Inset shows RT-qPCR validation of RNA-Seq analysis
