# Supplementary material for: CD44 alternative splicing senses intragenic DNA methylation in tumors via direct and indirect mechanisms
Source: Nucleic Acids Res. 2021 Jun 4;49(11):6213–37. doi: 10.1093/nar/gkab437 (PMC8216461; doi:10.1093/nar/gkab437)
Supplement: gkab437_Supplemental_Files [file gkab437_supplemental_files.zip › Batsche_Sup Table S2A_ΔΨ DKOvsWT.pdf]

Suplementary Table S2A :

Differential alternative RNA events in DKO cells compared to the parental HCT116 cells

Meta-analysis of RNA-seq data to detect splicing differences between HCT116 and HCT116-DKO cells, using the MAJIQ/Voila package (Vaquero-Garcia et al., 2016), as explained in Supplementary Figure S1F. In order to take into account the statistical dispersion of the data due to the differences in RNA extraction methods, three different comparisons as indicated have been conducted. The comparison of the 4 total RNAs for each cell-types has been conducted using data from (Blattler et al., 2014; Maunakea et al., 2013; Schrijver et al., 2013). The comparison of the 2 poly-adenylated RNAs for each cell-types has been conducted using data from (Schrijver et al., 2013; Simmer et al., 2012). The comparison of all 6 RNA-seq available has also been calculated. The results were presented in the Tables generated by the Voila visualization output.

The tables corresponding to these comparisons can be found in the following pages

RNA extracts	DKO vs WT	Genes	pages
Total RNA	4 vs 4	387	2 – 25
polyA RNA	2 vs 2	298	26 – 43
All RNA	6 vs 6	217	44 - 58

Combinaison of all comparisons : 653

Blattler, A., Yao, L., Witt, H., Guo, Y., Nicolet, C.M., Berman, B.P., and Farnham, P.J. (2014). Global loss of DNA methylation uncovers intronic enhancers in genes showing expression changes. Genome Biol. *15*, 469.

Maunakea, A.K., Chepelev, I., Cui, K., and Zhao, K. (2013). Intragenic DNA methylation modulates alternative splicing by recruiting MeCP2 to promote exon recognition. Cell Res.

Schrijver, W.A.M.E., Jiwa, L.S., van Diest, P.J., and Moelans, C.B. (2015). Promoter hypermethylation profiling of distant breast cancer metastases. Breast Cancer Res. Treat. *151*, 41–55.

Simmer, F., Brinkman, A.B., Assenov, Y., Matarese, F., Kaan, A., Sabatino, L., Villanueva, A., Huertas, D., Esteller, M., Lengauer, T., et al. (2012). Comparative genome-wide DNA methylation analysis of colorectal tumor and matched normal tissues. Epigenetics *7*, 1355–1367.

Vaquero-Garcia, J., Barrera, A., Gazzara, M.R., Gonzalez-Vallinas, J., Lahens, N.F., Hogenesch, J.B., Lynch, K.W., and Barash, Y. (2016). A new view of transcriptome complexity and regulation through the lens of local splicing variations. Elife *5*, e11752.

1) Differential alternative RNA events in DKO cells compared to the parental HCT116 cells in total RNA.

LSV ΔΨ Index: DKO Vs WT

#	Gene	LSV ID	LSV Type	← More in DKO | More in WT →
0	SCYL3	ENSG00000000457:169831703-169831938:target	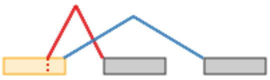	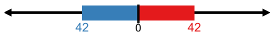 42 0 42
1	ALDH3B1	ENSG00000006534:67786535-67786724:source	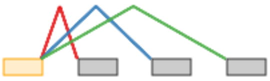	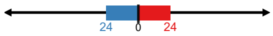 24 0 24
2	ALDH3B1	ENSG00000006534:67788954-67788995:target	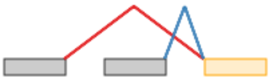	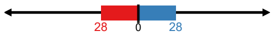 28 0 28
3	PKD1	ENSG00000008710:2141424-2141615:source	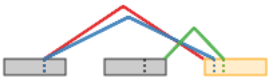	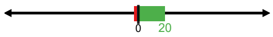 0 20
4	STARD3NL	ENSG00000010270:38254629-38254706:source	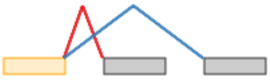	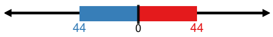 44 0 44
5	STARD3NL	ENSG00000010270:38256789-38256906:target	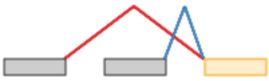	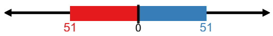 51 0 51
6	LRRC23	ENSG00000010626:7015573-7015919:target	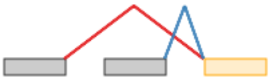	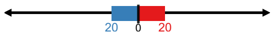 20 0 20
7	PIK3C2A	ENSG00000011405:17153463-17153585:target	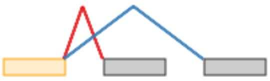	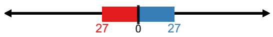 27 0 27
8	PHLDB1	ENSG00000019144:118521306-118526400:target	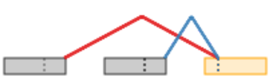	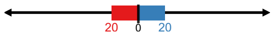 20 0 20
9	AKAP11	ENSG00000023516:42888030-42888076:target	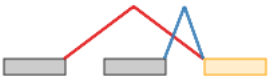	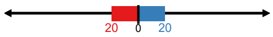 20 0 20
10	DEPDC1	ENSG00000024526:68949633-68949773:target	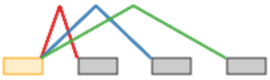	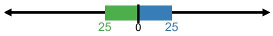 25 0 25
11	CD44	ENSG00000026508:35211557-35211976:source	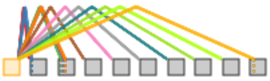	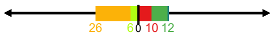 26 6 0 10 12
12	CD44	ENSG00000026508:35232793-35232996:target	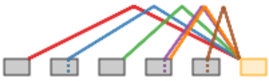	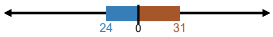 24 0 31
13	CD44	ENSG00000026508:35236213-35236461:target	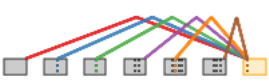	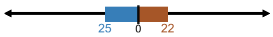 25 0 22
14	ARNTL2	ENSG00000029153:27538414-27538493:source	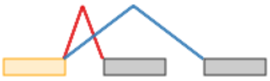	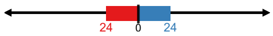 24 0 24
15	ARNTL2	ENSG00000029153:27542112-27542229:target	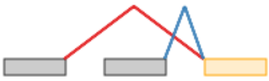	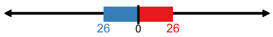 26 0 26
16	FAM13B	ENSG00000031003:137353991-137354203:source	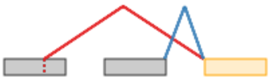	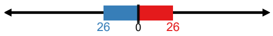 26 0 26
17	ALG1	ENSG00000033011:5134751-5135058:target	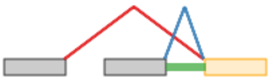	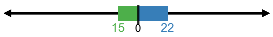 15 0 22
18	UBA6	ENSG00000033178:68547837-68547931:source	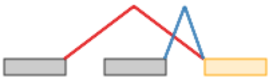	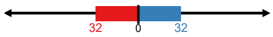 32 0 32

Gene LSV ID LSV Type ← More in DKO | More in WT →

#	Gene	LSV ID
19	ZZZ3	ENSG00000036549:78107207-78107340:source
20	MAT2B	ENSG00000038274:162939008-162939406:target
21	FAM65A	ENSG00000039523:67571981-67572453:target
22	JADE2	ENSG00000043143:133871548-133871605:target
23	DTNBP1	ENSG00000047579:15637975-15638035:source
24	ARID1B	ENSG00000049618:157256600-157256710:source
25	NEDD4L	ENSG00000049759:55989657-55989718:source
26	RELT	ENSG00000054967:73104289-73104962:target
27	CROCC	ENSG00000058453:17265404-17266183:source
28	CROCC	ENSG00000058453:17279797-17279976:target
29	YIPF1	ENSG00000058799:54317392-54317943:source
30	YBX3	ENSG00000060138:10856622-10857037:source
31	MEF2BNB-MEF2B	ENSG00000064489:19291495-19291570:target
32	SBNO2	ENSG00000064932:1109134-1109210:source
33	SBNO2	ENSG00000064932:1109500-1109597:target
34	SBNO2	ENSG00000064932:1154183-1154401:source
35	ZNF76	ENSG00000065029:35248833-35249001:target
36	SLK	ENSG00000065613:105767935-105768114:source
37	SLK	ENSG00000065613:105777918-105778047:target
38	NGEF	ENSG00000066248:233752743-233752817:source
39	RRP15	ENSG00000067533:218458629-218458797:source

LSV Type

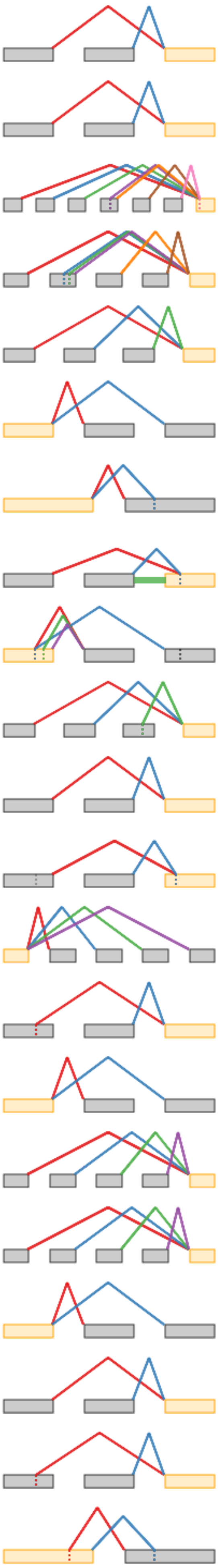

← More in DKO | More in WT →

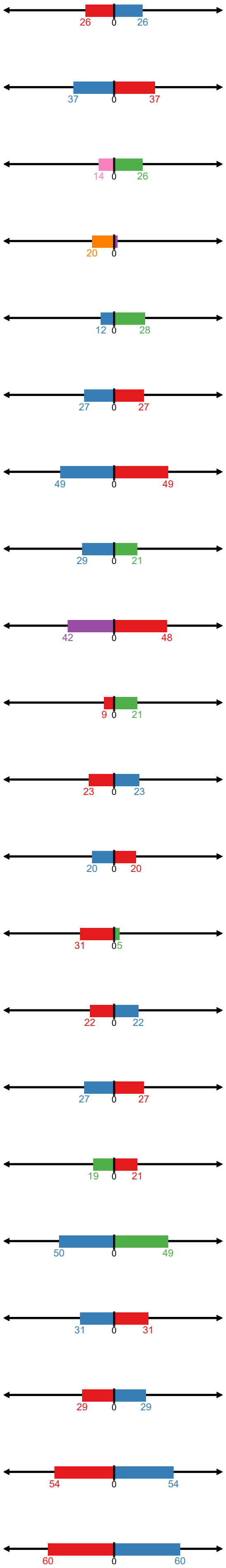

Gene LSV ID LSV Type ← More in DKO | More in WT →

#	Gene	LSV ID	LSV Type	← More in DKO | More in WT →
40	ST6GALNAC2	ENSG00000070731:74559792-74562353:source		38 0 38
41	OSBPL3	ENSG00000070882:24901232-24901388:source		33 0 33
42	NCK2	ENSG00000071051:106471504-106471745:target		27 0 29
43	PRKACA	ENSG00000072062:14218160-14218221:source		35 0 33
44	SELO	ENSG00000073169:50644746-50644949:target		35 0 35
45	PICALM	ENSG00000073921:85687666-85688048:source		27 0 27
46	PICALM	ENSG00000073921:85692172-85692271:target		17 0 25
47	CLASP1	ENSG00000074054:122187649-122187753:source		23 0 23
48	DPP8	ENSG00000074603:65782527-65782637:source		23 0 23
49	ZNF638	ENSG00000075292:71582849-71582910:target		24 0
50	ARHGEF1	ENSG00000076928:42410091-42410487:source		22 0 22
51	DGKD	ENSG00000077044:234368403-234368537:source		33 0 33
52	NFKB2	ENSG00000077150:104155645-104155737:target		25 0 35
53	ENSG00000077809	ENSG00000077809:74129177-74129264:source		21 0 21
54	DNM2	ENSG00000079805:10906048-10906422:source		24 0 24
55	DNM2	ENSG00000079805:10909063-10909248:target		23 0 23
56	DNM2	ENSG00000079805:10916592-10916643:source		21 0 21
57	DNM2	ENSG00000079805:10922940-10923053:target		23 0 23
58	SRCAP	ENSG00000080603:30711229-30711302:target		35 0 14
59	APLP2	ENSG00000084234:129992200-129992408:source		22 0 22

Gene LSV ID LSV Type ← More in DKO | More in WT →

#	Gene	LSV ID	LSV Type	← More in DKO | More in WT →
60	APLP2	ENSG00000084234:129996595-129996725:target		22 0 22
61	TAF9	ENSG00000085231:68646811-68648080:source		24 0 23
62	NFX1	ENSG00000086102:33366627-33366772:target		22 0 22
63	KHSRP	ENSG00000088247:6414468-6415312:target		20 0 25
64	DNMT3B	ENSG00000088305:31350191-31350505:source		86 0 86
65	DNMT3B	ENSG00000088305:31395568-31397162:target		87 0 26 62
66	EPB41L1	ENSG00000088367:34742664-34742818:source		25 0 25
67	DOCK9	ENSG00000088387:99452542-99452732:target		28 0 28
68	RAB11FIP3	ENSG00000090565:538851-539101:source		28 0 28
69	RAB11FIP3	ENSG00000090565:546824-547158:target		34 0 34
70	ITGA6	ENSG00000091409:173362703-173362828:source		29 0 29
71	ITGA6	ENSG00000091409:173368819-173371181:target		31 0 31
72	MYL6	ENSG00000092841:56554027-56554127:source		25 0 24
73	MYL6	ENSG00000092841:56555171-56555371:target		22 0 22
74	AGO1	ENSG00000092847:36354028-36354211:target		77 0 77
75	LRRFIP2	ENSG00000093167:37125127-37125297:source		24 0 24
76	LRRFIP2	ENSG00000093167:37136283-37136399:target		26 0 26
77	FAM21A	ENSG00000099290:51885129-51885209:source		22 0 11
78	FAM21A	ENSG00000099290:51885817-51886268:source		26 0 26
79	FAM21A	ENSG00000099290:51887343-51887555:target		26 0 12

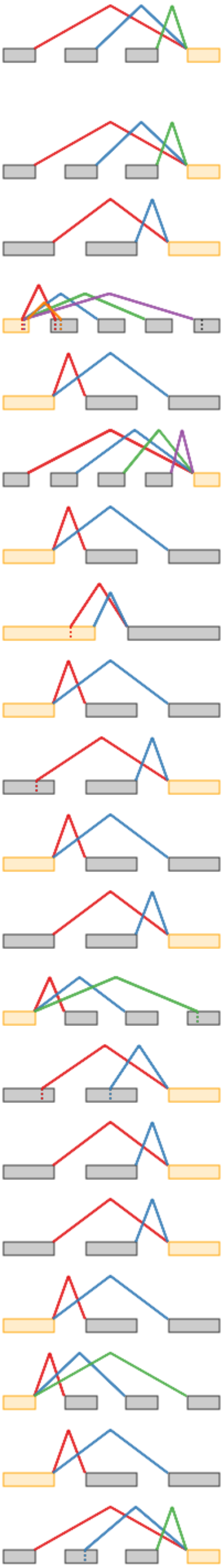

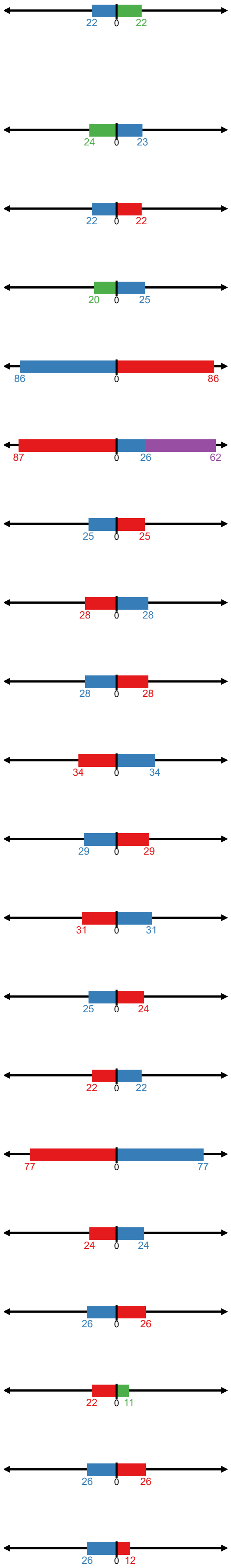

Gene LSV ID LSV Type ← More in DKO | More in WT →

#	Gene	LSV ID	LSV Type	← More in DKO | More in WT →
80	MYO9B	ENSG00000099331:17286472-17286550:source	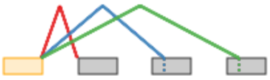	24 0 6
81	ARVCF	ENSG00000099889:19959409-19959494:target	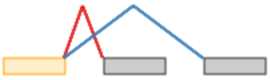	29 0 29
82	HIRA	ENSG00000100084:19385515-19385610:source	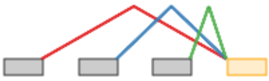	23 0 25
83	HIRA	ENSG00000100084:19393309-19393403:target	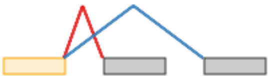	27 0 27
84	RP1-37E16.12	ENSG00000100101:38147779-38147835:target	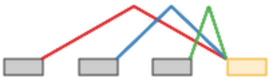	16 0 22
85	TRIOBP	ENSG00000100106:38147779-38147835:target	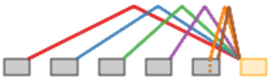	24 0 21
86	SBF1	ENSG00000100241:50894921-50895102:source	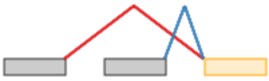	29 0 29
87	SBF1	ENSG00000100241:50897684-50897821:target	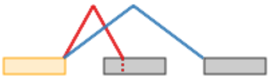	20 0 20
88	FOXRED2	ENSG00000100350:36897288-36897454:source	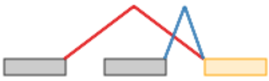	24 0 24
89	NIN	ENSG00000100503:51226575-51227077:target	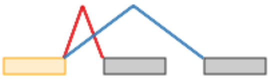	24 0 24
90	ZFYVE21	ENSG00000100711:104198957-104199099:target	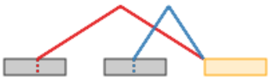	21 0 21
91	CCNB1IP1	ENSG00000100814:20784573-20784719:source	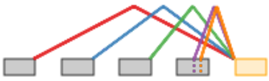	26 0 26
92	ACOT8	ENSG00000101473:44483798-44483931:target	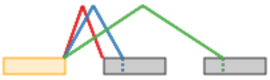	7 0 24
93	NXT2	ENSG00000101888:108781274-108781360:target	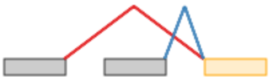	21 0 21
94	FNDC3A	ENSG00000102531:49688791-49688867:target	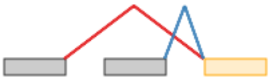	38 0 38
95	FNDC3A	ENSG00000102531:49752704-49752790:target	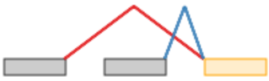	27 0 27
96	TSNAXIP1	ENSG00000102904:67864293-67864791:target	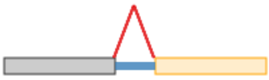	27 0 27
97	TAF1C	ENSG00000103168:84220507-84220669:target	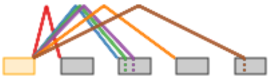	27 0
98	HAGHL	ENSG00000103253:778794-778985:source	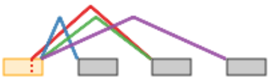	26 0 10
99	HAGHL	ENSG00000103253:779288-779733:target	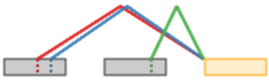	22 0 10

\# Gene LSV ID LSV Type ← More in DKO | More in WT →

#	Gene	LSV ID	LSV Type	← More in DKO | More in WT →
100	FAM173A	ENSG00000103254:771598-771702:target	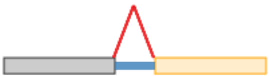	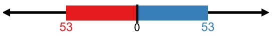
101	CAPN15	ENSG00000103326:583946-583998:source	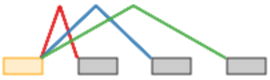	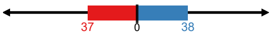
102	MAZ	ENSG00000103495:29821398-29821739:source	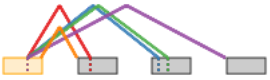	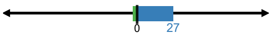
103	AAGAB	ENSG00000103591:67528968-67529158:source	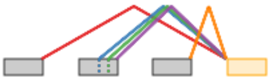	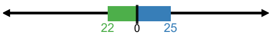
104	TRIM35	ENSG00000104228:27151557-27151827:target	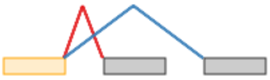	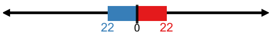
105	ZFAND1	ENSG00000104231:82630417-82630459:source	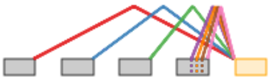	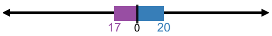
106	ARHGEF10	ENSG00000104728:1808060-1808350:source	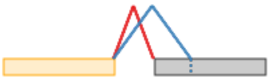	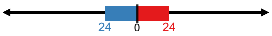
107	KCTD9	ENSG00000104756:25298059-25298189:source	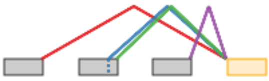	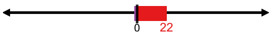
108	SARS2	ENSG00000104835:39435609-39435928:source	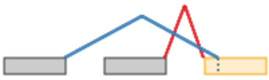	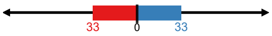
109	SARS2	ENSG00000104835:39435609-39435928:target	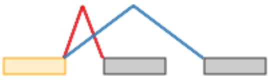	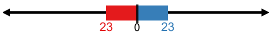
110	OLFM2	ENSG00000105088:9971321-9971470:source	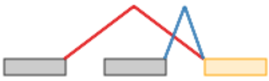	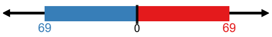
111	URI1	ENSG00000105176:30506443-30506611:target	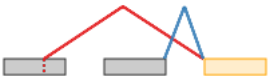	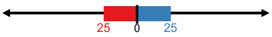
112	PLD3	ENSG00000105223:40872200-40872417:target	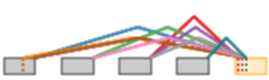	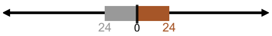
113	CD33	ENSG00000105383:51738773-51738931:source	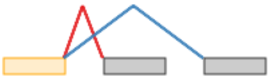	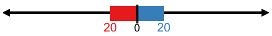
114	TYK2	ENSG00000105397:10464587-10464910:target	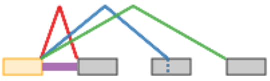	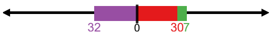
115	MEIS3	ENSG00000105419:47909213-47909783:target	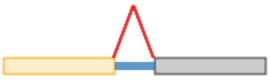	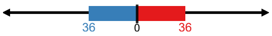
116	ARRDC2	ENSG00000105643:18119520-18119670:target	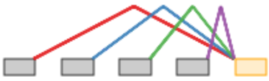	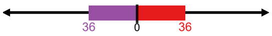
117	ZC3HAV1	ENSG00000105939:138728266-138732599:source	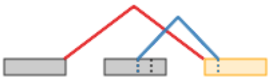	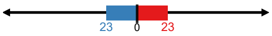
118	ADAP1	ENSG00000105963:959605-959687:source	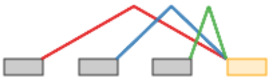	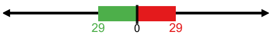
119	USP42	ENSG00000106346:6175472-6175582:target	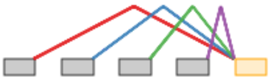	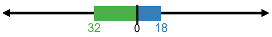

Gene LSV ID LSV Type ← More in DKO | More in WT →

#	Gene	LSV ID	LSV Type	← More in DKO | More in WT →
120	COA1	ENSG00000106603:43769028-43769316:target	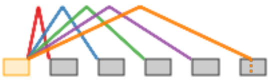	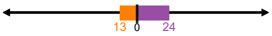
121	SPIN1	ENSG00000106723:91063856-91063904:source	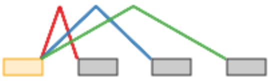	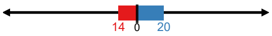
122	SPIN1	ENSG00000106723:91083287-91083520:target	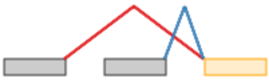	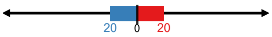
123	DVL1	ENSG00000107404:1274667-1274819:target	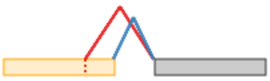	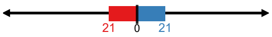
124	DDX50	ENSG00000107625:70666467-70666763:target	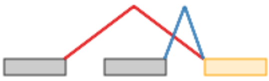	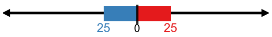
125	PLEKHA1	ENSG00000107679:124189140-124191867:target	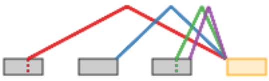	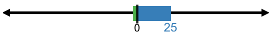
126	NEURL1	ENSG00000107954:105344293-105345113:source	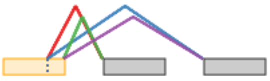	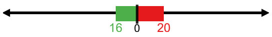
127	GALK1	ENSG00000108479:73753497-73754208:target	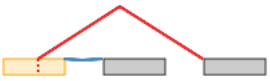	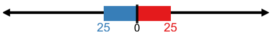
128	OCIAD1	ENSG00000109180:48834637-48834778:target	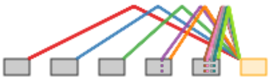	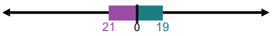
129	CLCN3	ENSG00000109572:170601201-170601358:target	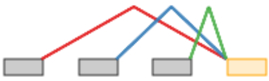	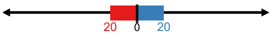
130	MFSD10	ENSG00000109736:2932788-2932877:target	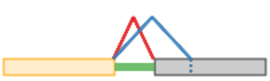	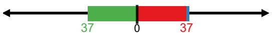
131	CTSC	ENSG00000109861:88053979-88059611:source	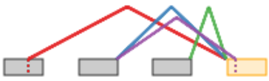	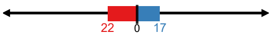
132	PVRL1	ENSG00000110400:119545869-119546379:target	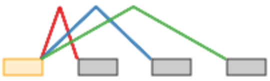	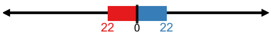
133	FOXM1	ENSG00000111206:2973849-2974020:source	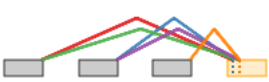	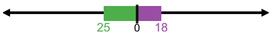
134	FOXM1	ENSG00000111206:2975559-2975687:target	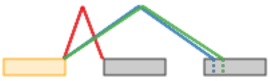	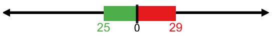
135	GNB3	ENSG00000111664:6955889-6956557:target	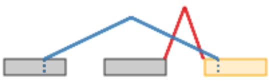	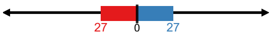
136	CDCA3	ENSG00000111665:6955889-6956067:target	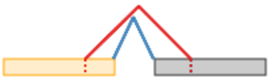	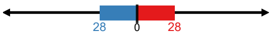
137	RP11-22B23.1	ENSG00000111788:9463737-9463886:target	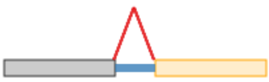	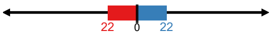
138	SOD2	ENSG00000112096:160169223-160169401:source	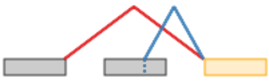	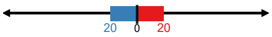
139	ZNF451	ENSG00000112200:56963859-56963939:source	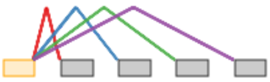	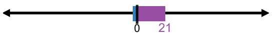

Gene LSV ID LSV Type ← More in DKO | More in WT →

#	Gene	LSV ID	LSV Type	← More in DKO | More in WT →
140	CEP72	ENSG00000112877:642325-642335:target	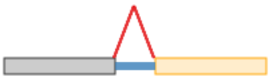	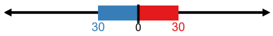
141	DROSHA	ENSG00000113360:31486598-31486669:target	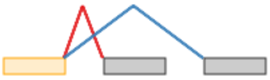	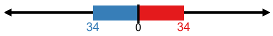
142	H2AFY	ENSG00000113648:134686504-134687234:source	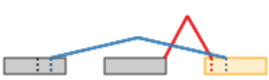	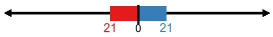
143	CRBN	ENSG00000113851:3195108-3195172:source	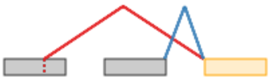	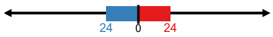
144	ARMC8	ENSG00000114098:138003273-138003368:source	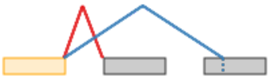	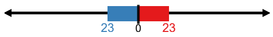
145	TFDP2	ENSG00000114126:141724283-141724386:source	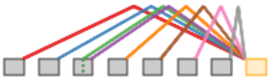	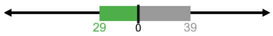
146	ECT2	ENSG00000114346:172473085-172473164:source	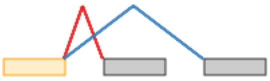	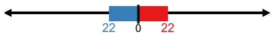
147	ECT2	ENSG00000114346:172474773-172474955:target	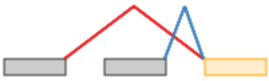	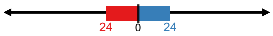
148	NKTR	ENSG00000114857:42661156-42661200:target	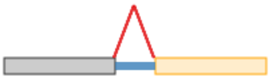	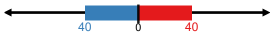
149	NEK4	ENSG00000114904:52800194-52800391:target	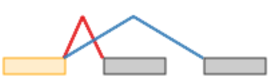	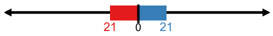
150	EPB41L5	ENSG00000115109:120858275-120858390:source	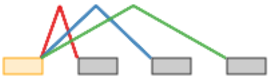	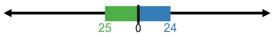
151	STK16	ENSG00000115661:220110614-220111146:target	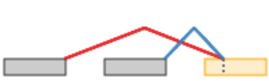	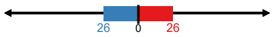
152	ORC4	ENSG00000115947:148733471-148733544:source	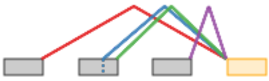	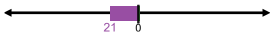
153	IVNS1ABP	ENSG00000116679:185274668-185274775:source	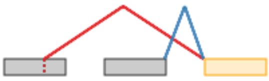	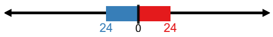
154	IVNS1ABP	ENSG00000116679:185275882-185276271:target	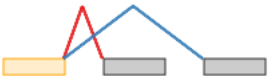	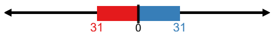
155	UAP1	ENSG00000117143:162560113-162560301:source	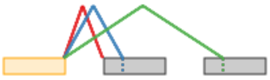	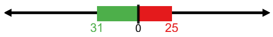
156	UAP1	ENSG00000117143:162567031-162567648:target	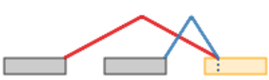	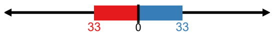
157	KLHL12	ENSG00000117153:202863312-202863410:target	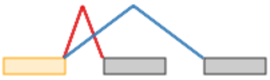	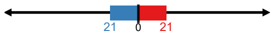
158	OSBPL9	ENSG00000117859:52225296-52226452:target	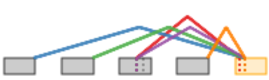	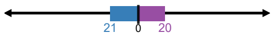
159	KMT2A	ENSG00000118058:118390274-118390507:source	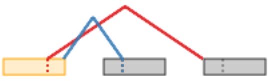	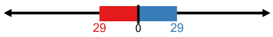

| # | Gene | LSV ID | LSV Type | ← More in DKO \| More in WT → |

#	Gene	LSV ID	LSV Type	← More in DKO | More in WT →
160	KMT2A	ENSG00000118058:118391981-118392132:target	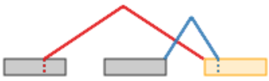	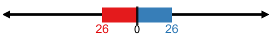
161	PPP2R4	ENSG00000119383:131904724-131904831:target	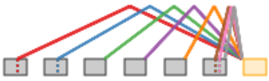	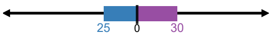
162	MAPKAP1	ENSG00000119487:128246722-128246862:source	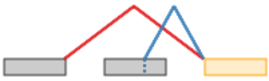	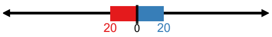
163	SLC17A5	ENSG00000119899:74354130-74354326:source	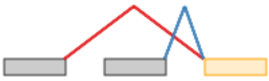	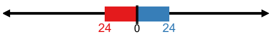
164	KANSL1	ENSG00000120071:44249093-44249598:source	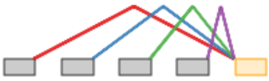	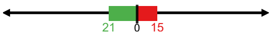
165	HSPH1	ENSG00000120694:31722096-31722229:source	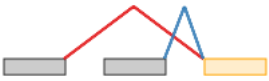	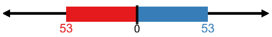
166	HSPH1	ENSG00000120694:31724091-31724319:target	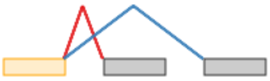	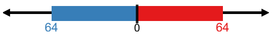
167	ZNF644	ENSG00000122482:91383609-91383948:source	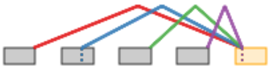	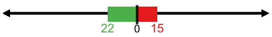
168	CALD1	ENSG00000122786:134613505-134613651:target	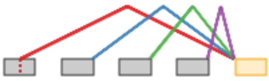	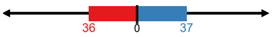
169	PKN1	ENSG00000123143:14551955-14552255:target	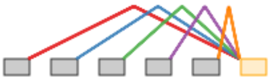	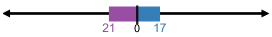
170	USP45	ENSG00000123552:99916413-99916494:source	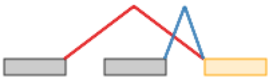	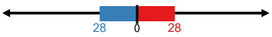
171	PLA2G12A	ENSG00000123739:110638704-110638869:source	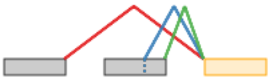	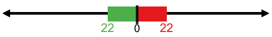
172	SRSF6	ENSG00000124193:42089343-42092245:target	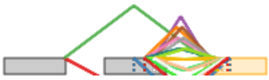	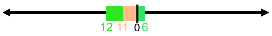
173	STX16	ENSG00000124222:57245568-57245659:target	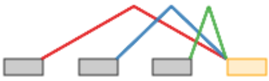	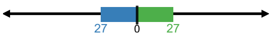
174	ZNF576	ENSG00000124444:44101246-44101697:target	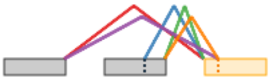	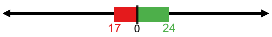
175	MYRF	ENSG00000124920:61533097-61533184:target	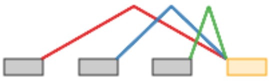	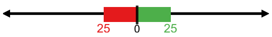
176	AHNAK	ENSG00000124942:62289526-62289624:target	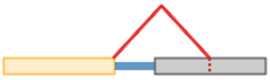	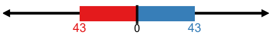
177	AHNAK	ENSG00000124942:62303941-62304039:source	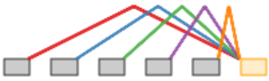	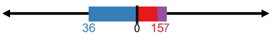
178	EMC3	ENSG00000125037:10016068-10016172:source	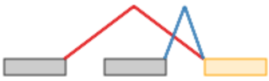	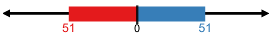
179	EMC3	ENSG00000125037:10019073-10019130:target	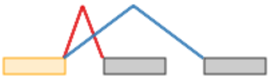	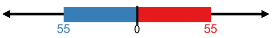
#	Gene	LSV ID	LSV Type	← More in DKO | More in WT →

#	Gene	LSV ID	LSV Type	← More in DKO | More in WT →
180	TTF1	ENSG00000125482:135275422-135275645:source		21 0 21
181	NSRP1	ENSG00000126653:28445056-28445191:source		21 0 20
182	ABHD8	ENSG00000127220:17402940-17403663:source		32 0 32
183	TMEM175	ENSG00000127419:944209-944306:target		0 27
184	EPS15L1	ENSG00000127527:16495940-16496022:target		0 29
185	MACF1	ENSG00000127603:39929284-39930581:source		26 0 26
186	MACF1	ENSG00000127603:39934265-39934404:target		25 0 25
187	SPECC1	ENSG00000128487:20135513-20135764:source		20 0 12
188	MYO1B	ENSG00000128641:192265108-192265194:source		17 0 30
189	MYO1B	ENSG00000128641:192272384-192272915:target		30 0 30
190	ARPP19	ENSG00000128989:52861309-52861436:target		26 0 22
191	KIF1C	ENSG00000129250:4903145-4903265:target		23 0 23
192	MPDU1	ENSG00000129255:7490479-7490589:target		9 0 23
193	SLC44A2	ENSG00000129353:10736929-10736977:target		25 0 26
194	BCL2L2	ENSG00000129473:23777150-23777408:source		24 0 24
195	SAT1	ENSG00000130066:23801917-23802330:source		22 0 22
196	SAT1	ENSG00000130066:23802870-23803546:target		25 0 10 11
197	USHBP1	ENSG00000130307:17379565-17379900:target		17 8 0 36
198	MRPL34	ENSG00000130312:17403418-17403663:source		32 0 32
199	LAMA5	ENSG00000130702:60898339-60898726:target		0 23

Gene LSV ID LSV Type ← More in DKO | More in WT →

#	Gene	LSV ID	LSV Type	← More in DKO | More in WT →
200	CLIP1	ENSG00000130779:122825300-122828591:source		24 0 24
201	CLIP1	ENSG00000130779:122837273-122837333:target		43 0 43
202	DNMT1	ENSG00000130816:10305496-10305811:target		24 13 0 39
203	DUSP9	ENSG00000130829:152913373-152913780:target		27 0 28
204	AKAP12	ENSG00000131016:151669846-151674887:target		69 0 74
205	GSE1	ENSG00000131149:85682158-85682357:target		20 0 20
206	DIAPH1	ENSG00000131504:140966609-140966764:source		30 0 30
207	DIAPH1	ENSG00000131504:140998365-140998622:target		28 0 28
208	RAF1	ENSG00000132155:12627180-12627404:target		24 0 24
209	UNK	ENSG00000132478:73780809-73781065:source		35 0 30
210	SDF2	ENSG00000132581:26982305-26983340:source		21 0 21
211	ARHGEF11	ENSG00000132694:156907041-156907288:source		26 0 26
212	ARHGEF11	ENSG00000132694:156909340-156909702:target		31 0 31
213	SLC41A1	ENSG00000133065:205770081-205770188:source		21 0 21
214	MICAL2	ENSG00000133816:12229272-12229916:source		23 0 23
215	PRPF38B	ENSG00000134186:109238899-109238959:source		22 0 22
216	PRPF38B	ENSG00000134186:109240322-109241449:target		12 0 24
217	NAV1	ENSG00000134369:201755557-201755705:source		22 0 22
218	FADS2	ENSG00000134824:61605250-61605360:target		76 0 8 58
219	DMTF1	ENSG00000135164:86823041-86823418:source		22 0 22

Gene LSV ID LSV Type ← More in DKO | More in WT →

#	Gene	LSV ID	LSV Type	← More in DKO | More in WT →
220	DMTF1	ENSG00000135164:86824347-86825653:target	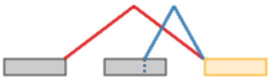	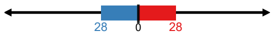 28 0 28
221	MAP3K7	ENSG00000135341:91246056-91246937:source	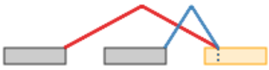	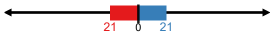 21 0 21
222	B4GALNT1	ENSG00000135454:58022831-58022957:source	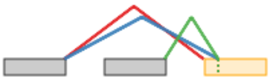	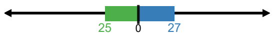 25 0 27
223	B4GALNT1	ENSG00000135454:58023935-58024115:target	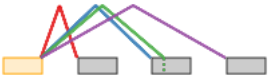	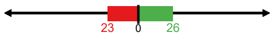 23 0 26
224	KRT7	ENSG00000135480:52639135-52639416:target	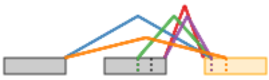	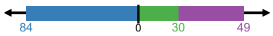 84 0 30 49
225	DHX9	ENSG00000135829:182811680-182811812:source	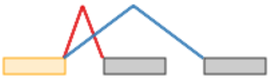	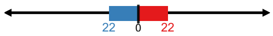 22 0 22
226	DHX9	ENSG00000135829:182821368-182821479:target	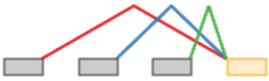	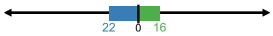 22 0 16
227	FLNB	ENSG00000136068:58124009-58124256:source	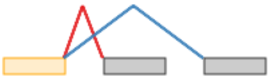	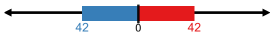 42 0 42
228	FLNB	ENSG00000136068:58128377-58128479:target	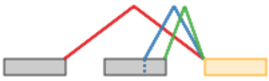	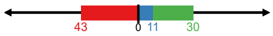 43 0 11 30
229	CIDEB	ENSG00000136305:24775153-24775343:target	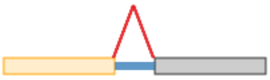	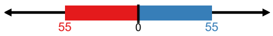 55 0 55
230	VEZF1	ENSG00000136451:56060271-56060754:source	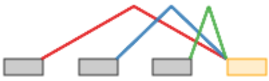	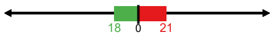 18 0 21
231	BIN1	ENSG00000136717:127821509-127821594:source	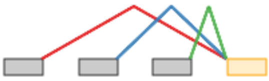	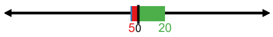 5 0 20
232	DNAJC1	ENSG00000136770:22217969-22218070:target	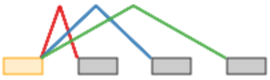	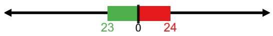 23 0 24
233	NUMA1	ENSG00000137497:71721832-71721900:source	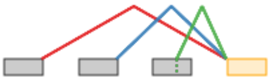	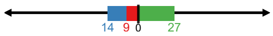 14 9 0 27
234	NUMA1	ENSG00000137497:71723941-71725082:target	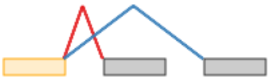	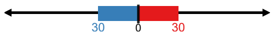 30 0 30
235	RNF121	ENSG00000137522:71671796-71671937:target	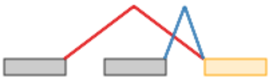	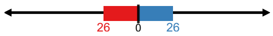 26 0 26
236	KIF23	ENSG00000137807:69718214-69718521:target	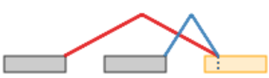	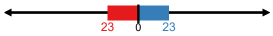 23 0 23
237	CENPO	ENSG00000138092:25042223-25045245:target	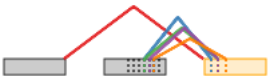	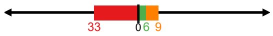 33 0 6 9
238	TMEM180	ENSG00000138111:104229750-104229832:source	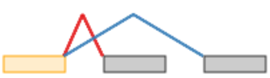	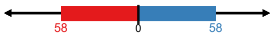 58 0 58
239	TMEM180	ENSG00000138111:104231030-104231153:target	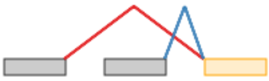	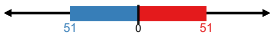 51 0 51

| # | Gene | LSV ID | LSV Type | ← More in DKO \| More in WT → |

#	Gene	LSV ID	LSV Type	← More in DKO | More in WT →
240	MYOF	ENSG00000138119:95148787-95148911:source	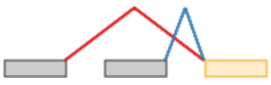	34 0 34
241	DIRC2	ENSG00000138463:122598110-122599986:target	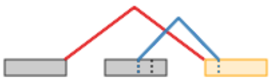	43 0 43
242	USP8	ENSG00000138592:50784898-50785110:target	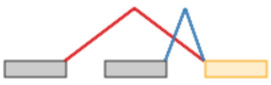	20 0 20
243	USO1	ENSG00000138768:76714843-76715054:source	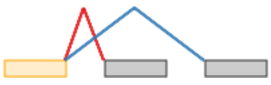	28 0 28
244	USO1	ENSG00000138768:76720775-76720885:target	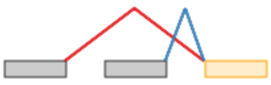	21 0 21
245	RGS3	ENSG00000138835:116356710-116356811:target	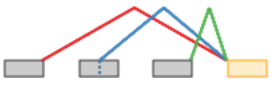	60 0 59
246	POC1B	ENSG00000139323:89860450-89860699:target	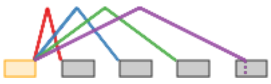	0 22
247	NABP2	ENSG00000139579:56618618-56618719:target	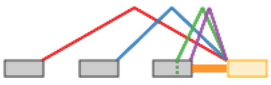	6 7 5 0 21
248	TPM1	ENSG00000140416:63347928-63349317:target	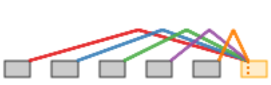	75 0 76
249	NCOR1	ENSG00000141027:16068185-16068475:target	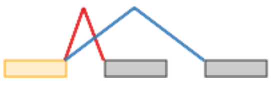	37 0 37
250	G6PC3	ENSG00000141349:42152048-42152138:source	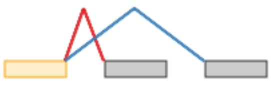	20 0 20
251	ARRB2	ENSG00000141480:4623511-4623594:source	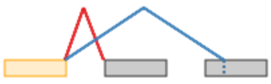	25 0 25
252	ARRB2	ENSG00000141480:4623845-4623935:target	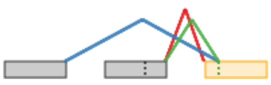	26 0 22
253	MBD1	ENSG00000141644:47798835-47799108:target	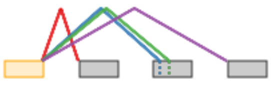	0 25
254	COL6A2	ENSG00000142173:47546417-47546455:source	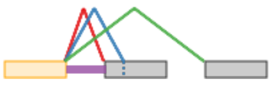	30 6 0 39
255	TMEM50B	ENSG00000142188:34821089-34821496:target	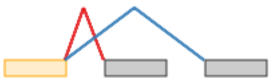	22 0 22
256	CAPN10	ENSG00000142330:241537305-241537504:target	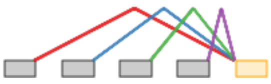	0 20
257	ADAM15	ENSG00000143537:155033239-155033308:source	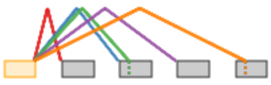	11 21 0 26
258	ADAM15	ENSG00000143537:155034380-155034593:target	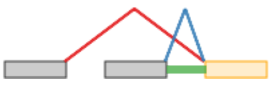	31 0 34
259	ADAM15	ENSG00000143537:155034637-155034845:target	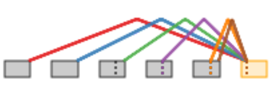	16 0 23
#	Gene	LSV ID	LSV Type	← More in DKO | More in WT →

#	Gene	LSV ID	LSV Type	← More in DKO | More in WT →
260	SLC25A26	ENSG00000144741:66419902-66419966:source		34 0 34
261	SLC25A26	ENSG00000144741:66428114-66428503:target		41 0 41
262	PHLDB2	ENSG00000144824:111632166-111636510:target		19 0 29
263	TNIP1	ENSG00000145901:150444521-150444692:source		25 0 31
264	FAM193B	ENSG00000146067:176951502-176952488:source		0 21
265	TMEM181	ENSG00000146433:159046735-159046786:source		24 0 24
266	WTAP	ENSG00000146457:160169223-160170447:source		20 0 20
267	C7orf50	ENSG00000146540:1049586-1049779:source		29 0 30
268	CASK	ENSG00000147044:41418750-41419100:target		26 0 18
269	PROSER2	ENSG00000148426:11911365-11914276:target		22 0 22
270	TCF7L2	ENSG00000148737:114799784-114799885:target		32 0 32
271	IMMP1L	ENSG00000148950:31455007-31455302:source		29 0 12
272	IMMP1L	ENSG00000148950:31484719-31484852:target		31 0 23
273	PGAP2	ENSG00000148985:3845500-3845962:target		32 0 15
274	ARFGAP2	ENSG00000149182:47193832-47193884:source		41 0 42
275	ARFGAP2	ENSG00000149182:47194649-47195033:target		51 0 51
276	ALDOA	ENSG00000149925:30078555-30078687:target		25 0 25
277	HMGA2	ENSG00000149948:66232299-66232349:source		27 0 16
278	MKX	ENSG00000150051:28023385-28023720:target		21 0 21
279	MTMR12	ENSG00000150712:32239107-32239279:target		22 0 25
#	Gene	LSV ID	LSV Type	← More in DKO | More in WT →

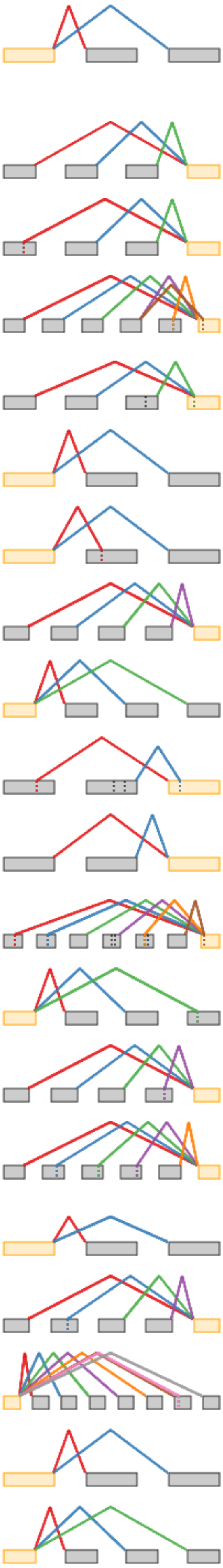

#	Gene	LSV ID	LSV Type	← More in DKO | More in WT →
280	UBE3B	ENSG00000151148:109927724-109927809:source	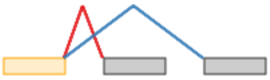	28 0 28
281	FAM177A1	ENSG00000151327:35515659-35515834:target	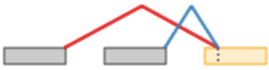	40 0 40
282	DST	ENSG00000151914:56324929-56326866:source	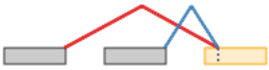	37 0 37
283	DST	ENSG00000151914:56328363-56329114:target	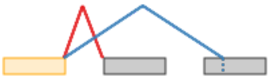	31 0 31
284	AC093838.4	ENSG00000152117:132258754-132258806:target	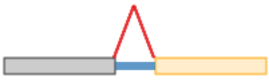	28 0 28
285	ATG10	ENSG00000152348:81571964-81572676:target	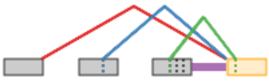	31 0 31
286	RBMS1	ENSG00000153250:161140772-161141379:target	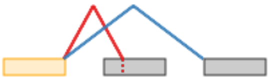	33 0 33
287	SREK1	ENSG00000153914:65449396-65449618:source	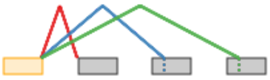	26 0 26
288	SLC25A28	ENSG00000155287:101373226-101373681:target	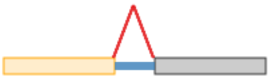	29 0 29
289	LARP1	ENSG00000155506:154173606-154173755:target	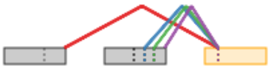	9 0 24
290	BACH1	ENSG00000156273:30693542-30693835:target	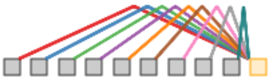	22 0 17
291	PAXIP1	ENSG00000157212:154739542-154739800:source	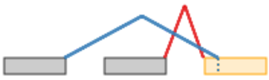	20 0 20
292	AGAP1	ENSG00000157985:236877106-236877267:target	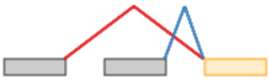	21 0 21
293	EPB41	ENSG00000159023:29391469-29391733:target	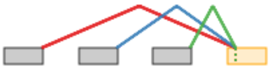	45 0 34
294	RCAN1	ENSG00000159200:35895835-35896587:source	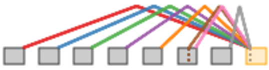	23 0 33
295	SIK3	ENSG00000160584:116734384-116735784:source	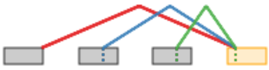	19 0 21
296	ANO10	ENSG00000160746:43407840-43408466:source	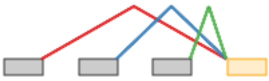	28 0 28
297	RUSC1	ENSG00000160753:155294636-155294734:target	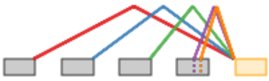	9 0 26
298	LMNA	ENSG00000160789:156109364-156109880:target	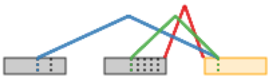	14 0 33
299	ENSG00000160828	ENSG00000160828:74129177-74129236:source	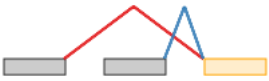	21 0 21
#	Gene	LSV ID	LSV Type	← More in DKO | More in WT →

#	Gene	LSV ID	LSV Type	← More in DKO | More in WT →
300	DMKN	ENSG00000161249:35996620-35996667:target		21 0 20
301	DMKN	ENSG00000161249:35999107-35999459:source		24 0 24
302	CLPB	ENSG00000162129:72013183-72013417:source		26 0 26
303	CLPB	ENSG00000162129:72013183-72013417:target		33 0 40
304	NOL9	ENSG00000162408:6593340-6593501:source		19 0 32
305	NOL9	ENSG00000162408:6609316-6609758:target		26 0 26
306	KIAA1522	ENSG00000162522:33233388-33233558:target		27 0 27
307	C1orf52	ENSG00000162642:85725041-85725355:target		22 0 22
308	C2orf47	ENSG00000162972:200824477-200824603:source		25 0 25
309	C2orf47	ENSG00000162972:200828456-200828848:target		20 0 20
310	RPP14	ENSG00000163684:58295781-58296133:target		18 0 24
311	APBB2	ENSG00000163697:40936631-40936716:source		54 0 54
312	ARFIP1	ENSG00000164144:153750777-153750878:target		9 0 28
313	CCDC112	ENSG00000164221:114604579-114604697:source		30 0 30
314	DNAAF5	ENSG00000164818:810108-812612:target		22 0 22
315	NOS3	ENSG00000164867:150707987-150708074:source		7 0 21
316	KIAA0196	ENSG00000164961:126044483-126044956:source		20 0
317	FAM69B	ENSG00000165716:139612029-139612646:source		22 0 22
318	NDEL1	ENSG00000166579:8339240-8339363:source		22 0 22
319	VPS39	ENSG00000166887:42483459-42483758:source		20 0 20

Gene LSV ID LSV Type ← More in DKO | More in WT →

#	Gene	LSV ID	LSV Type	← More in DKO | More in WT →
320	VPS39	ENSG00000166887:42492094-42492159:target		23 0 23
321	ENGASE	ENSG00000167280:77082015-77084681:target		0 25
322	TRAPPC9	ENSG00000167632:141415392-141415797:source		37 0 37
323	FAM57A	ENSG00000167695:636338-636421:source		24 10 0 38
324	FAM57A	ENSG00000167695:643745-643840:target		20 0 20
325	SRR	ENSG00000167720:2226430-2226639:target		50 0 50
326	RAB26	ENSG00000167964:2198625-2198979:source		20 0 20
327	ATXN2L	ENSG00000168488:28836687-28836723:target		20 0 12
328	SNRNP48	ENSG00000168566:7599906-7601757:target		14 0 26
329	FABP6	ENSG00000170231:159640734-159640826:source		30 0 21
330	FABP6	ENSG00000170231:159659042-159659280:target		22 0 22
331	KRT8	ENSG00000170421:53292453-53292683:target		29 0 30
332	POLH	ENSG00000170734:43543887-43544177:source		26 0 22
333	ENSG00000171282	ENSG00000171282:79424436-79424859:source		13 36 0 53
334	MALT1	ENSG00000172175:56377208-56377304:source		39 0 39
335	MALT1	ENSG00000172175:56381315-56381341:target		36 0 36
336	RAD9A	ENSG00000172613:67160125-67160253:target		38 0 38
337	SSH3	ENSG00000172830:67077239-67077439:source		23 0 23
338	PHF8	ENSG00000172943:54019169-54019442:source		28 0 28
339	SNAPC5	ENSG00000174446:66789828-66790151:target		23 0 12

Gene LSV ID LSV Type ← More in DKO | More in WT →

#	Gene	LSV ID	LSV Type	← More in DKO | More in WT →
340	RSRC1	ENSG00000174891:157839892-157840087:target		23 0
341	ZDHHC14	ENSG00000175048:158014020-158014178:target		34 0 39
342	LRRC75A-AS1	ENSG00000175061:16342635-16342728:source		20 0 10
343	POLD4	ENSG00000175482:67120364-67120631:source		18 0 32
344	FOSL1	ENSG00000175592:65664280-65664477:target		25 0 18
345	CCDC57	ENSG00000176155:80141650-80141808:target		21 0 21
346	FBXO46	ENSG00000177051:46213887-46216831:source		21 0 21
347	PUS1	ENSG00000177192:132416060-132417225:source		23 0 24
348	PAWR	ENSG00000177425:80083834-80084171:source		24 0 26
349	CPNE7	ENSG00000178773:89654159-89654183:target		29 0 29
350	CPNE7	ENSG00000178773:89662892-89663654:target		15 0 21
351	PER1	ENSG00000179094:8048069-8048488:source		24 0 24
352	SAMD4B	ENSG00000179134:39873848-39873931:source		20 0 12
353	NME9	ENSG00000181322:138003273-138003368:source		23 0 23
354	IBA57	ENSG00000181873:228362393-228362730:target		31 0 31
355	TNRC18	ENSG00000182095:5434071-5434226:source		28 0 17
356	FBXL6	ENSG00000182325:145581288-145581633:target		26 0 28
357	TTC3	ENSG00000182670:38519781-38519903:source		20 0 20
358	ANXA2	ENSG00000182718:60689454-60689537:target		25 0 25
359	RRP7BP	ENSG00000182841:42976223-42976365:target		31 0 31

Gene LSV ID LSV Type ← More in DKO | More in WT →

#	Gene	LSV ID	LSV Type	← More in DKO | More in WT →
360	MTA1	ENSG00000182979:105931671-105932915:source	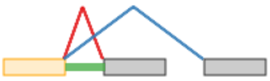	23 0 23
361	MTA1	ENSG00000182979:105935804-105935835:target	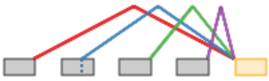	24 0 14
362	NAA38	ENSG00000183011:7761567-7761814:target	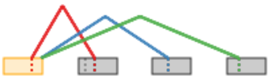	48 0 48
363	EP400	ENSG00000183495:132537888-132538142:source	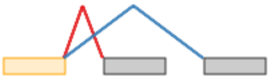	48 0 48
364	EP400	ENSG00000183495:132539644-132539753:target	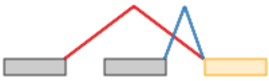	43 0 43
365	UPP1	ENSG00000183696:48134360-48134424:source	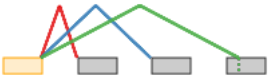	9 22 0 33
366	UPP1	ENSG00000183696:48141421-48141579:target	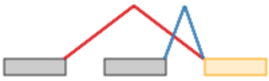	29 0 29
367	TBL3	ENSG00000183751:2028553-2032934:target	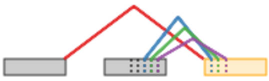	50 0 14 12 20
368	SMTN	ENSG00000183963:31484088-31484239:source	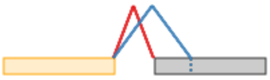	24 0 24
369	SMTN	ENSG00000183963:31493255-31493324:target	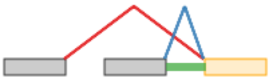	29 0 136
370	VPS33B	ENSG00000184056:91549602-91549675:source	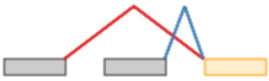	20 0 20
371	TOP1MT	ENSG00000184428:144413394-144413509:source	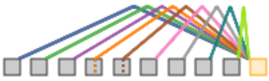	9 50 0 74
372	TXNRD2	ENSG00000184470:19868145-19868240:source	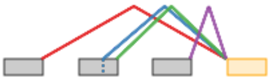	25 0 31
373	JAG2	ENSG00000184916:105617202-105617248:source	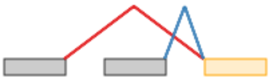	20 0 20
374	JAG2	ENSG00000184916:105617620-105617733:target	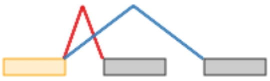	22 0 22
375	PARPBP	ENSG00000185480:102517664-102517819:source	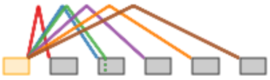	23 0 10
376	PCYT2	ENSG00000185813:79858841-79862820:source	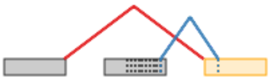	33 0 33
377	GPAT2	ENSG00000186281:96688706-96688771:source	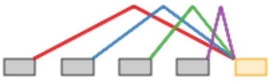	21 0 22
378	GPAT2	ENSG00000186281:96689671-96689748:target	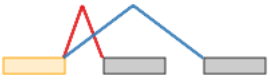	21 0 21
379	GPAT2	ENSG00000186281:96691931-96692053:source	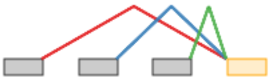	10 0 24

| # | Gene | LSV ID | LSV Type | ← More in DKO \| More in WT → |

#	Gene	LSV ID	LSV Type	← More in DKO | More in WT →
380	GPAT2	ENSG00000186281:96693739-96693838:target		34 0 34
381	NF2	ENSG00000186575:30077428-30077590:source		26 0 26
382	NF2	ENSG00000186575:30090741-30094587:target		26 0 25
383	ESPN	ENSG00000187017:6508701-6508862:target		20 0 21
384	FANCA	ENSG00000187741:89865574-89865640:target		31 0 22
385	UROS	ENSG00000188690:127483449-127483796:target		6 0 20
386	ZDHHC9	ENSG00000188706:128947653-128947701:source		25 0 25
387	S100A4	ENSG00000196154:153517434-153517573:target		37 0 37
388	XPNPEP3	ENSG00000196236:41322273-41328819:target		38 0 38
389	ZNF775	ENSG00000196456:150068317-150068327:target		54 0 54
390	PRPF40A	ENSG00000196504:153533965-153533989:source		22 0 22
391	PRPF40A	ENSG00000196504:153535643-153535986:target		20 0 20
392	ERI2	ENSG00000196678:20807662-20810389:source		38 0 25
393	ERI2	ENSG00000196678:20810585-20810695:target		26 0 12
394	CD47	ENSG00000196776:107762145-107766139:source		10 0 29
395	CD47	ENSG00000196776:107770786-107773586:target		22 0 22
396	WDR45	ENSG00000196998:48935700-48935771:source		19 0 20
397	ABCB8	ENSG00000197150:150731360-150731515:target		23 0 17
398	HTT	ENSG00000197386:3124616-3125193:source		58 0 58
399	MIB2	ENSG00000197530:1564414-1564691:target		36 0 39

Gene LSV ID LSV Type ← More in DKO | More in WT →

#	Gene	LSV ID	LSV Type	← More in DKO | More in WT →
400	SPTAN1	ENSG00000197694:131353756-131353904:source		
401	SPTAN1	ENSG00000197694:131356454-131356652:target		
402	PSAP	ENSG00000197746:73585594-73585650:target		
403	TPM2	ENSG00000198467:35684485-35684547:source		
404	TPM2	ENSG00000198467:35685142-35685336:target		
405	CTNND1	ENSG00000198561:57529234-57529591:source		
406	MDM4	ENSG00000198625:204511912-204512072:source		
407	MDM4	ENSG00000198625:204515925-204516005:target		
408	TTC37	ENSG00000198677:94845298-94845391:target		
409	FAN1	ENSG00000198690:31200321-31200461:target		
410	SMURF1	ENSG00000198742:98639741-98639855:target		
411	INF2	ENSG00000203485:105155943-105156076:source		
412	GIGYF2	ENSG00000204120:233568131-233568199:target		
413	PFDN6	ENSG00000204220:33257547-33257697:target		
414	C12orf73	ENSG00000204954:104350082-104350526:target		
415	IPO7	ENSG00000205339:9459301-9459592:source		
416	ITSN1	ENSG00000205726:35186217-35186811:source		
417	HLA-H	ENSG00000206341:29855732-29856170:source		
418	HLA-H	ENSG00000206341:29894501-29895176:target		
419	HLA-H	ENSG00000206341:29911899-29912393:target		

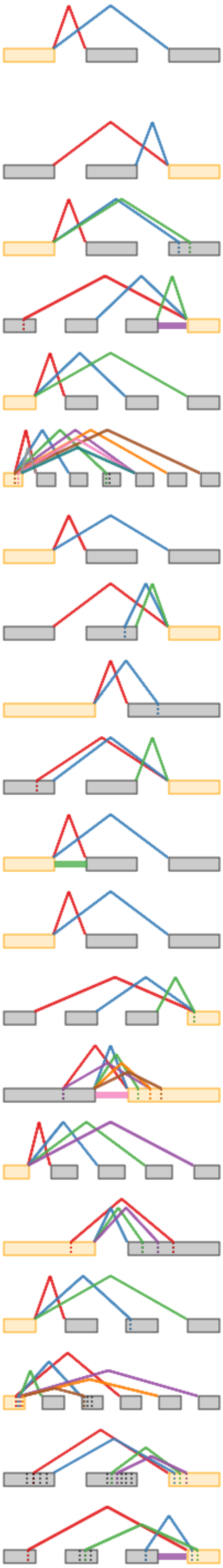

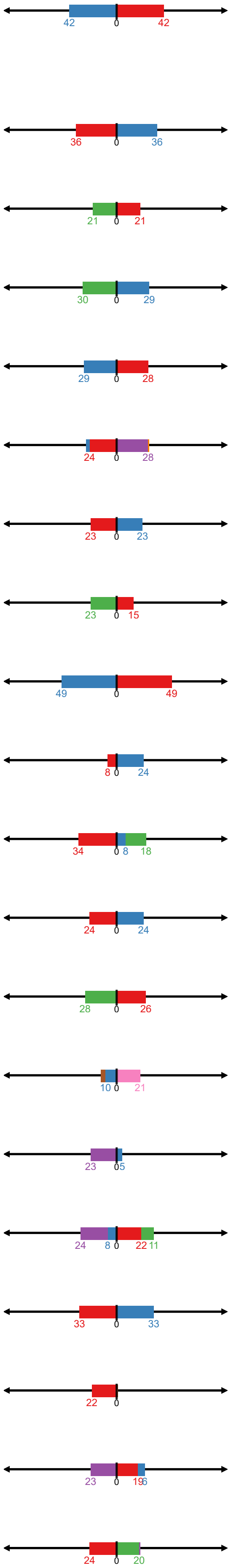

| # | Gene | LSV ID | LSV Type | ← More in DKO \| More in WT → |

#	Gene	LSV ID	LSV Type	← More in DKO | More in WT →
420	ARHGEF35	ENSG00000213214:143971053-143971214:target	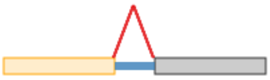	42 0 42
421	MEF2B	ENSG00000213999:19291495-19291570:target	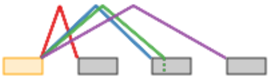	31 0 6
422	AC024560.3	ENSG00000214135:197348575-197348739:source	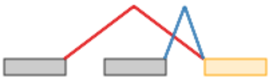	41 0 41
423	AC024560.3	ENSG00000214135:197350091-197350253:target	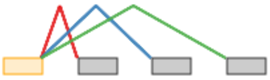	33 0 33
424	GOLGA8B	ENSG00000215252:34845946-34846157:source	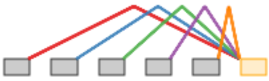	26 0 19
425	ENSG00000215513	ENSG00000215513:20387159-20387732:target	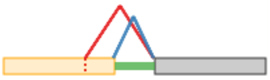	20 0 20
426	RP11-465B22.3	ENSG00000217801:1001210-1001836:target	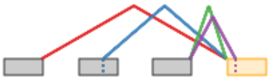	36 24 0 35 24
427	TRIM16	ENSG00000221926:15546035-15546278:source	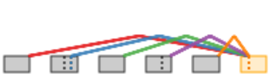	25 0 15
428	AC093818.1	ENSG00000225205:173362703-173362828:source	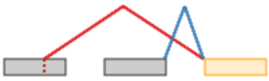	30 0 30
429	AC093818.1	ENSG00000225205:173368167-173368870:target	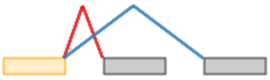	31 0 31
430	PROSER2-AS1	ENSG00000225778:11891612-11894214:source	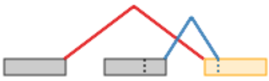	46 0 46
431	PROSER2-AS1	ENSG00000225778:11911365-11911500:target	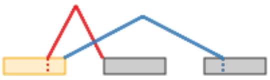	22 0 22
432	POLR2J2	ENSG00000228049:102306524-102306610:target	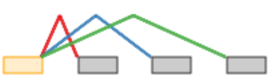	5 0 20
433	RP4-613B23.1	ENSG00000230084:42661156-42661200:target	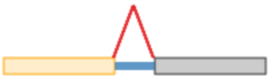	40 0 40
434	IPO9-AS1	ENSG00000231871:201755557-201755705:source	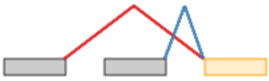	22 0 22
435	ENSG00000232637	ENSG00000232637:146032648-146034408:source	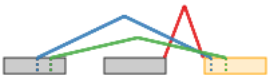	21 0 21
436	AC083884.8	ENSG00000232729:74129177-74129236:source	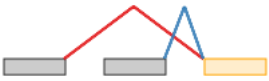	21 0 21
437	RP11-166B2.1	ENSG00000234719:12021230-12021736:source	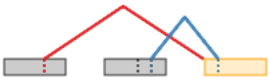	24 0 24
438	COX19	ENSG00000240230:959605-959687:source	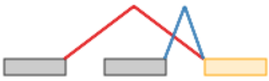	28 0 28
439	ARHGAP8	ENSG00000241484:45204187-45204318:source	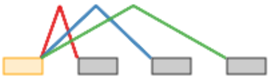	49 0 49

Gene LSV ID LSV Type ← More in DKO | More in WT →

#	Gene	LSV ID	LSV Type	← More in DKO | More in WT →
440	ARHGAP8	ENSG00000241484:45210552-45210638:target	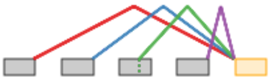	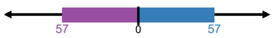
441	C22orf39	ENSG00000242259:19385515-19385610:source	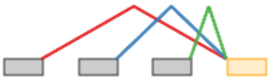	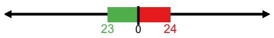
442	C22orf39	ENSG00000242259:19393309-19393403:target	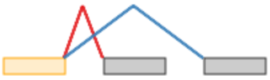	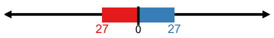
443	DECR2	ENSG00000242612:460677-460784:target	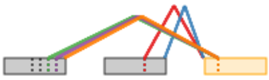	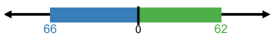
444	AC004893.11	ENSG00000242687:98639741-98639855:target	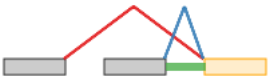	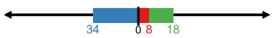
445	ENSG00000243452	ENSG00000243452:148560843-148561115:target	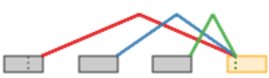	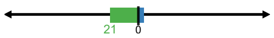
446	AC004967.7	ENSG00000243554:97599082-97599157:source	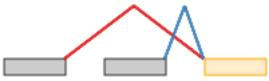	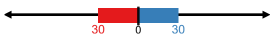
447	RP4-545C24.1	ENSG00000244198:143971053-143971214:target	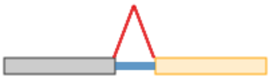	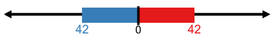
448	OR2A1-AS1	ENSG00000244479:143971053-143971214:target	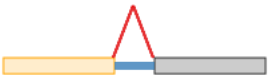	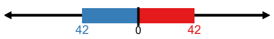
449	PRR5-ARHGAP8	ENSG00000248405:45204187-45204318:source	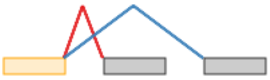	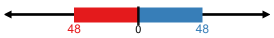
450	PRR5-ARHGAP8	ENSG00000248405:45210552-45210638:target	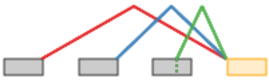	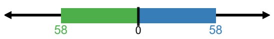
451	AP000304.12	ENSG00000249209:35186217-35186376:source	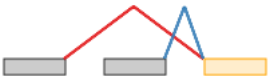	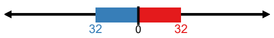
452	LINC01234	ENSG00000249550:114204880-114205014:target	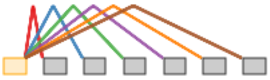	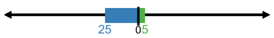
453	ZFPM2-AS1	ENSG00000251003:106799677-106799824:target	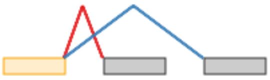	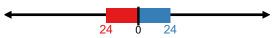
454	ALG11	ENSG00000253710:52602455-52603800:target	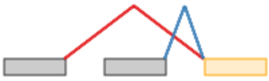	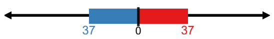
455	STX16-NPEPL1	ENSG00000254995:57245568-57245659:target	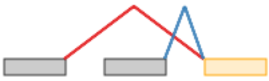	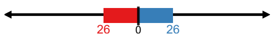
456	RP11-770J1.3	ENSG00000255435:118390100-118390507:source	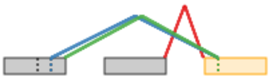	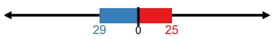
457	RP11-770J1.3	ENSG00000255435:118391961-118392132:target	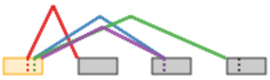	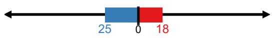
458	RP11-386G11.10	ENSG00000258017:49579302-49579704:target	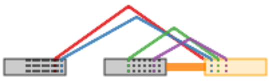	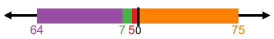
459	RP11-12J10.3	ENSG00000258539:126395205-126395456:source	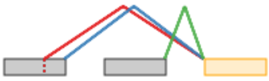	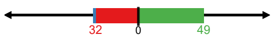

| # | Gene | LSV ID | LSV Type | ← More in DKO \| More in WT → |

#	Gene	LSV ID	LSV Type	← More in DKO | More in WT →
460	BCL2L2-PABPN1	ENSG00000258643:23776969-23777408:source	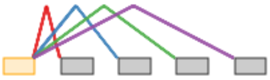	28 0 19 5
461	RP11-430B1.2	ENSG00000259577:52497062-52498071:target	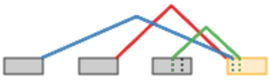	23 0 22
462	RP11-304L19.1	ENSG00000259933:2141437-2141615:source	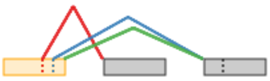	0 20
463	FAM157C	ENSG00000260528:90235330-90235426:target	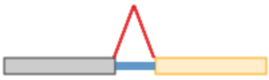	20 0 20
464	VPS9D1-AS1	ENSG00000261373:89778264-89778630:source	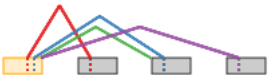	18 0 20
465	VPS9D1-AS1	ENSG00000261373:89782870-89783312:target	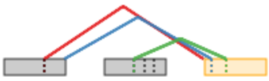	12 0 23
466	AC004702.2	ENSG00000263494:20135513-20135718:source	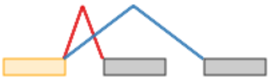	20 0 20
467	RP11-589P10.7	ENSG00000267047:6916985-6917061:target	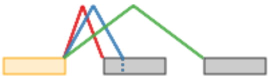	22 0 13
468	CTD-2265O21.3	ENSG00000267424:12937589-12937706:source	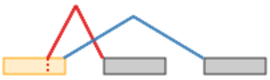	28 0 28
469	RP11-126O1.4	ENSG00000267476:56377208-56377304:source	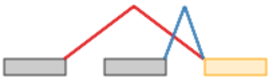	39 0 39
470	RP11-126O1.4	ENSG00000267476:56381315-56381341:target	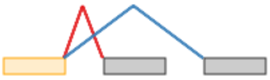	36 0 36
471	POLR2J2	ENSG00000267645:102306524-102306610:target	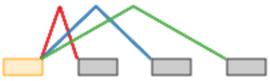	50 20
472	FBXO17	ENSG00000269190:39435368-39436051:source	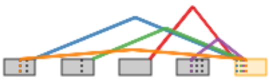	23 12 0 46
473	FBXO17	ENSG00000269190:39435368-39436051:target	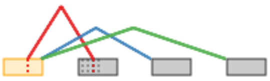	627 0 42
474	FBXO17	ENSG00000269190:39436392-39438058:target	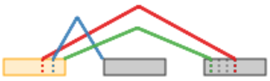	30 0 30
475	CTD-3138B18.4	ENSG00000269545:58758077-58758160:source	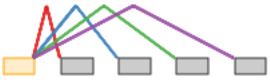	28 0 20
476	CTC-360G5.8	ENSG00000269547:39435609-39435928:source	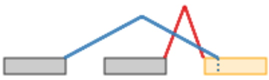	33 0 33
477	CTC-360G5.8	ENSG00000269547:39435609-39435928:target	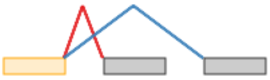	
#	Gene	LSV ID	LSV Type	← More in DKO | More in WT →

LSV filters

- [x] 5-prime
- [x] 3-prime
- [x] Exon skipping
- [x] Single Source
- [x] Single Target

2) Differential alternative RNA events in DKO cells compared to the parental HCT116 cells in polyA+ RNA.

BioCiphers

LSV ΔΨ Index: DKO Vs WT

LSV filters

- [x] 5-prime
- [x] 3-prime
- [x] Exon skipping
- [x] Single Source
- [x] Single Target

Number of junctions:
from:
to:

Number of exons:
from:
to:

#	Gene	LSV ID	LSV Type	← More in DKO | More in WT →
0	CFLAR	ENSG00000003402:202000695-202000830:source	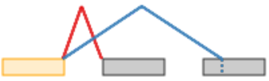	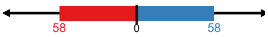
1	CFLAR	ENSG00000003402:202004909-202005458:target	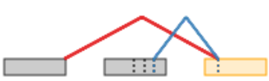	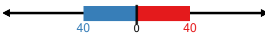
2	KDM1A	ENSG00000004487:23356962-23357127:source	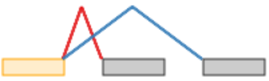	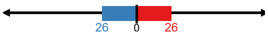
3	CDC27	ENSG00000004897:45232038-45232230:target	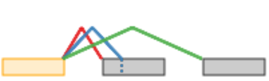	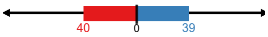
4	RALA	ENSG00000006451:39726230-39726380:source	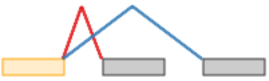	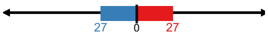
5	VPS41	ENSG00000006715:38948754-38948829:target	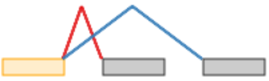	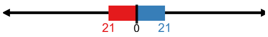
6	NFIX	ENSG00000008441:13183861-13183923:target	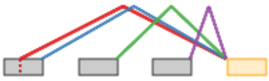	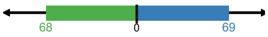
7	MED24	ENSG00000008838:38209722-38210345:source	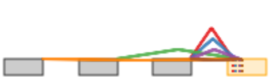	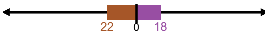
8	MED24	ENSG00000008838:38210614-38210995:target	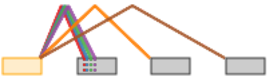	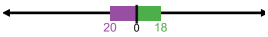
9	SEC62	ENSG00000008952:169711443-169716161:target	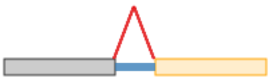	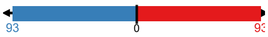
10	STARD3NL	ENSG00000010270:38254629-38254706:source	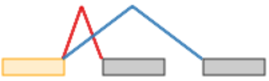	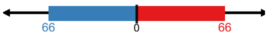
11	STARD3NL	ENSG00000010270:38256789-38256906:target	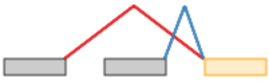	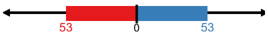
12	NISCH	ENSG00000010322:52512113-52512298:source	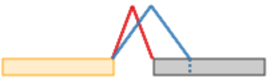	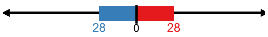
13	CLK1	ENSG00000013441:201724403-201724469:source	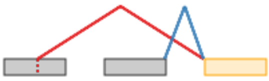	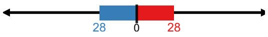
14	RTFDC1	ENSG00000022277:55048357-55048451:target	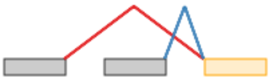	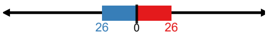
15	RNH1	ENSG00000023191:500847-502181:source	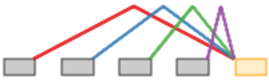	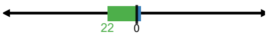
16	DEPDC1	ENSG00000024526:68944827-68945003:source	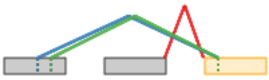	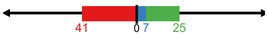
17	CD44	ENSG00000026508:35211557-35211976:source	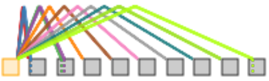	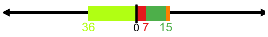
18	CD44	ENSG00000026508:35236213-35236461:target	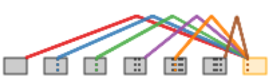	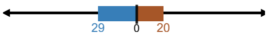
19	AGPAT4	ENSG00000026652:161570219-161570321:source	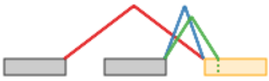	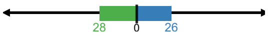

| # | Gene | LSV ID | LSV Type | ← More in DKO \| More in WT → |

LSV filters

- [x] 5-prime
- [x] 3-prime
- [x] Exon skipping
- [x] Single Source
- [x] Single Target

Number of junctions:
from:
to:

Number of exons:
from:
to:

#	Gene	LSV ID	LSV Type	← More in DKO | More in WT →
20	BCLAF1	ENSG00000029363:136590575-136591097:target	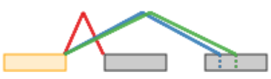	29 0 26
21	FAM136A	ENSG00000035141:70528736-70529222:target	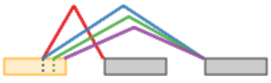	22 0 21
22	BOD1L1	ENSG00000038219:13582752-13582831:target	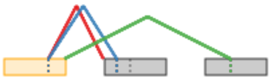	21 0
23	MAT2B	ENSG00000038274:162939008-162939406:target	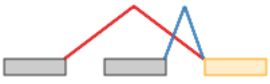	42 0 42
24	VCAN	ENSG00000038427:82807921-82808759:source	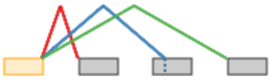	45 0 43
25	MAP4	ENSG00000047849:47963255-47963368:target	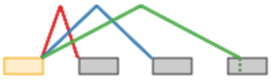	28 0 29
26	NEDD4L	ENSG00000049759:55912659-55912740:target	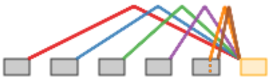	742 0 53
27	NEDD4L	ENSG00000049759:55989657-55989718:source	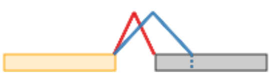	41 0 41
28	PUM2	ENSG00000055917:20462954-20463221:source	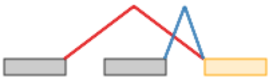	36 0 36
29	PUM2	ENSG00000055917:20482708-20482992:target	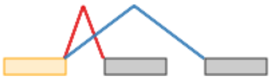	38 0 38
30	ZC3H11A	ENSG00000058673:203767974-203771389:target	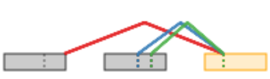	35 0 32
31	CCAR1	ENSG00000060339:70480971-70481420:source	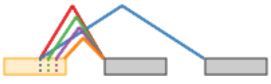	22 0 13
32	CCAR1	ENSG00000060339:70482212-70482334:target	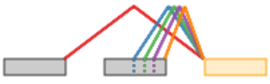	23 0 15
33	SLK	ENSG00000065613:105767935-105768114:source	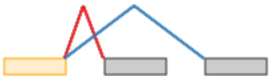	24 0 24
34	RRP15	ENSG00000067533:218458629-218458797:source	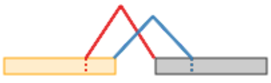	75 0 75
35	PDCD2	ENSG00000071994:170887945-170888809:target	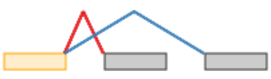	22 0 22
36	PRKACA	ENSG00000072062:14218160-14218221:source	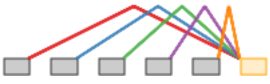	17 0 22
37	SREBF1	ENSG00000072310:17723322-17724178:source	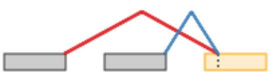	29 0 29
38	DERL2	ENSG00000072849:5383374-5383593:source	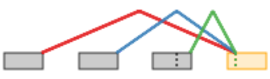	24 0 7
39	PICALM	ENSG00000073921:85707869-85707972:target	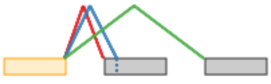	31 0 22
40	NT5C2	ENSG00000076685:104866346-104866463:source	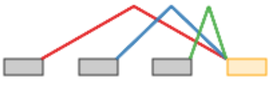	31 0 1510

| # | Gene | LSV ID | LSV Type | ← More in DKO \| More in WT → |

LSV filters

- [x] 5-prime
- [x] 3-prime
- [x] Exon skipping
- [x] Single Source
- [x] Single Target

Number of junctions:
from:
to:

Number of exons:
from:
to:

#	Gene	LSV ID	LSV Type	← More in DKO | More in WT →
41	SAR1A	ENSG00000079332:71921614-71921687:source		23 0 34
42	DNM2	ENSG00000079805:10909063-10909248:target		23 0 23
43	EPB41L2	ENSG00000079819:131206236-131206408:target		26 0 24
44	ITGAE	ENSG00000083457:3623601-3623913:source		20 0 20
45	CD59	ENSG00000085063:33757928-33757991:target		41 0 40
46	KLHL42	ENSG00000087448:27950648-27955973:target		27 0 27
47	AURKA	ENSG00000087586:54963212-54963258:source		0 39
48	NDUFB2	ENSG00000090266:140396471-140396642:source		22 0 15
49	LRRFIP2	ENSG00000093167:37125127-37125297:source		42 0 42
50	RP1-37E16.12	ENSG00000100101:38147779-38147835:target		31 0 39
51	TRIOBP	ENSG00000100106:38147779-38147835:target		33 0 37
52	THOC5	ENSG00000100296:29924926-29925228:source		30 0 30
53	THOC5	ENSG00000100296:29949660-29950243:target		40 0 40
54	EIF3D	ENSG00000100353:36922046-36922178:source		0 21
55	PMM1	ENSG00000100417:41979963-41980062:source		21 0 21
56	EIF5	ENSG00000100664:103800339-103800597:source		43 0 43
57	ZC3H14	ENSG00000100722:89073587-89073707:target		14 0 30
58	GMPR2	ENSG00000100938:24702425-24702546:target		27 0 20
59	TPD52L2	ENSG00000101150:62505021-62505169:source		28 0 28
60	ACTR5	ENSG00000101442:37384500-37384682:source		45 0 45
61	NXT2	ENSG00000101888:108781274-108781360:target		29 0 29

Gene LSV ID LSV Type ← More in DKO | More in WT →

LSV filters

- [x] 5-prime
- [x] 3-prime
- [x] Exon skipping
- [x] Single Source
- [x] Single Target

Number of junctions:
from:
to:

Number of exons:
from:
to:

#	Gene	LSV ID	LSV Type	← More in DKO | More in WT →
62	MOSPD1	ENSG00000101928:134030844-134031064:target		45 0 46
63	FBXO31	ENSG00000103264:87380780-87380856:source		21 0 21
64	CSPP1	ENSG00000104218:68107617-68108498:target		23 0 23
65	CCNE1	ENSG00000105173:30312629-30312724:source		57 0 57
66	FBL	ENSG00000105202:40336931-40337054:target		31 0 31
67	LSR	ENSG00000105699:35757262-35757330:target		24 0 11
68	LMBR1	ENSG00000105983:156526555-156526951:target		43 0 43
69	PRKAG2	ENSG00000106617:151329155-151329439:target		16 0 22
70	CLIP2	ENSG00000106665:73794354-73795371:target		34 0 34
71	FUBP3	ENSG00000107164:133507589-133507665:source		61 0 61
72	RAPGEF1	ENSG00000107263:134518626-134518804:source		27 0 27
73	ATE1	ENSG00000107669:123661906-123662135:target		44 0 36
74	ACBD5	ENSG00000107897:27512267-27512381:target		48 0 48
75	EDRF1	ENSG00000107938:127421973-127422120:target		34 0 34
76	XPNPEP1	ENSG00000108039:111646004-111646099:source		28 0 28
77	XPNPEP1	ENSG00000108039:111648254-111648353:target		29 0 29
78	FRG1	ENSG00000109536:190881903-190881994:target		21 0 17
79	CLCN3	ENSG00000109572:170601201-170601358:target		24 0 24
80	CTSC	ENSG00000109861:88067904-88068250:target		27 0 27
81	FOXM1	ENSG00000111206:2973849-2974020:source		32 0 8
82	FOXM1	ENSG00000111206:2975559-2975687:target		45 0 45

Gene LSV ID LSV Type ← More in DKO | More in WT →

LSV filters

- [x] 5-prime
- [x] 3-prime
- [x] Exon skipping
- [x] Single Source
- [x] Single Target

Number of junctions:
from:
to:

Number of exons:
from:
to:

#	Gene	LSV ID
83	CEP72	ENSG00000112877:644253-644540:target
84	ERGIC1	ENSG00000113719:172341568-172341841:target
85	SELK	ENSG00000113811:53920878-53920961:source
86	UBE3A	ENSG00000114062:25599180-25599573:source
87	UBE3A	ENSG00000114062:25601039-25601203:target
88	ECT2	ENSG00000114346:172473085-172473164:source
89	ECT2	ENSG00000114346:172474773-172474955:target
90	GNAI2	ENSG00000114353:50289532-50289574:target
91	DGUOK	ENSG00000114956:74153953-74154179:source
92	FANCL	ENSG00000115392:58449077-58449177:target
93	PLEKHB2	ENSG00000115762:131862420-131862971:source
94	C2orf42	ENSG00000115998:70443885-70444126:target
95	TIA1	ENSG00000116001:70443536-70443631:source
96	UCHL5	ENSG00000116750:192997215-192997278:target
97	CRYZ	ENSG00000116791:75190395-75190518:source
98	ACADM	ENSG00000117054:76253182-76253260:target
99	CD46	ENSG00000117335:207930885-207930987:target
100	OSBPL9	ENSG00000117859:52117663-52117713:source
101	SENP5	ENSG00000119231:196654667-196654750:target
102	PPP2R4	ENSG00000119383:131904724-131904831:target
103	MAPKAP1	ENSG00000119487:128246722-128246862:source

LSV Type | ← More in DKO | More in WT →

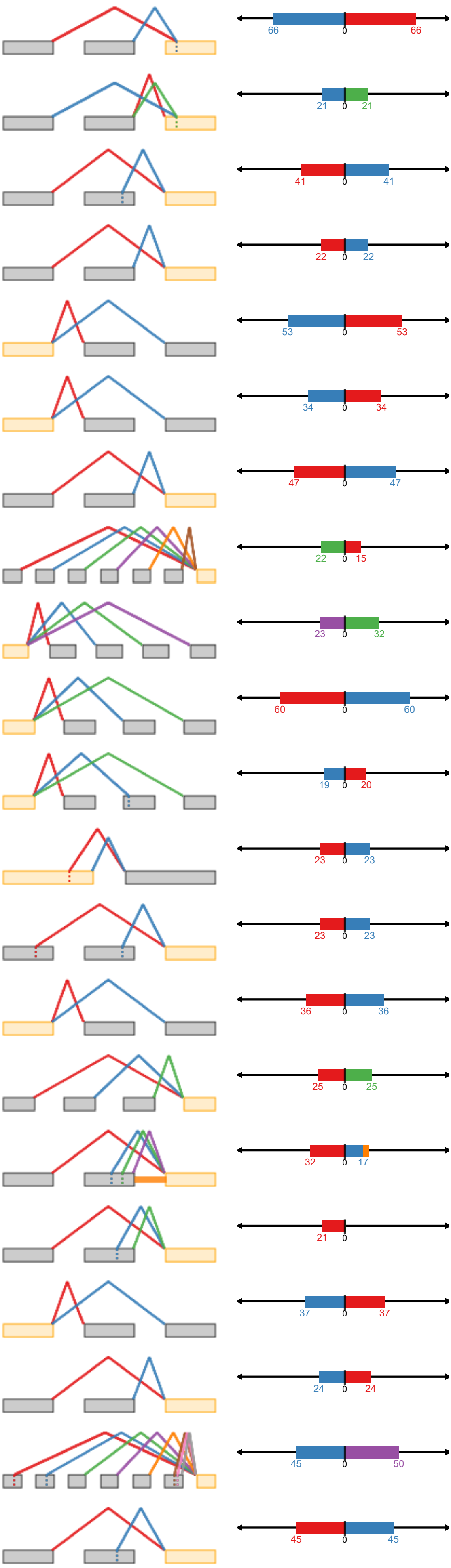

| Gene | LSV ID | LSV Type | ← More in DKO | More in WT →

LSV filters

- [x] 5-prime
- [x] 3-prime
- [x] Exon skipping
- [x] Single Source
- [x] Single Target

Number of junctions:
from:
to:

Number of exons:
from:
to:

#	Gene	LSV ID	LSV Type	← More in DKO | More in WT →
104	MAPKAP1	ENSG00000119487:128305338-128305447:target		7 0 31
105	ZNF410	ENSG00000119725:74353320-74353653:source		32 0 43
106	MND1	ENSG00000121211:154318371-154318485:source		21 0 21
107	C7orf49	ENSG00000122783:134853056-134853812:source		6 0 33
108	CALD1	ENSG00000122786:134625843-134625988:target		20 0 20
109	ACOT9	ENSG00000123130:23751289-23751334:source		34 0 19
110	EBPL	ENSG00000123179:50234859-50235344:source		21 0 7
111	SMUG1	ENSG00000123415:54577201-54578080:source		51 0 49
112	MORF4L2	ENSG00000123562:102933427-102933579:source		23 0 28
113	EXOSC9	ENSG00000123737:122737524-122737602:target		35 0 30 6
114	USP9X	ENSG00000124486:41088820-41089149:source		24 0 24
115	SERPINB6	ENSG00000124570:2959402-2961561:source		20 0 25
116	AHNAK	ENSG00000124942:62289186-62289624:target		19 0 21
117	MIF4GD	ENSG00000125457:73264019-73264273:source		22 0 22
118	RP11-51F16.8	ENSG00000125695:61791366-61791468:source		28 0 23
119	ITPA	ENSG00000125877:3193815-3193872:source		20 0 20
120	ITPA	ENSG00000125877:3194631-3194704:target		48 0 50
121	KLC1	ENSG00000126214:104037960-104038157:source		33 0 33
122	KLC1	ENSG00000126214:104053611-104053701:target		20 0 20
123	KLC1	ENSG00000126214:104145646-104146135:source		28 0 36
124	KLC1	ENSG00000126214:104166259-104167064:target		16 0 26

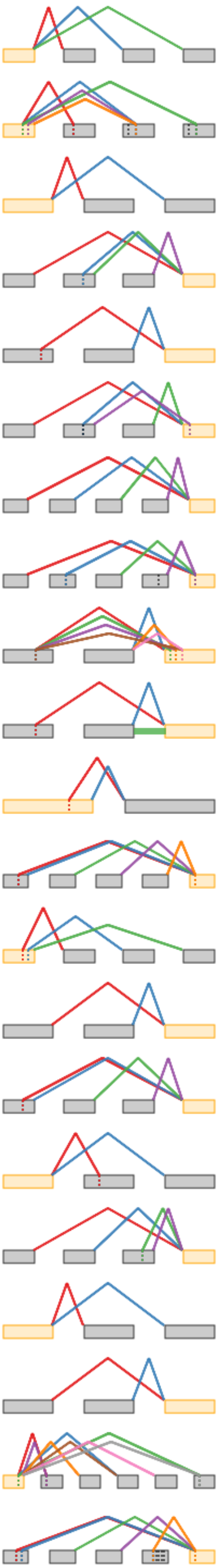

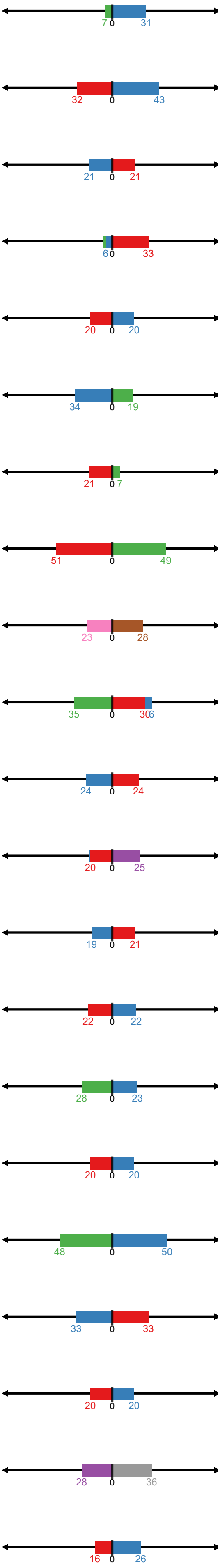

#	Gene	LSV ID	LSV Type	← More in DKO | More in WT →

LSV filters

- [x] 5-prime
- [x] 3-prime
- [x] Exon skipping
- [x] Single Source
- [x] Single Target

Number of junctions:
from:
to:

Number of exons:
from:
to:

#	Gene	LSV ID	LSV Type	← More in DKO | More in WT →
125	NSRP1	ENSG00000126653:28443664-28443881:source		31 | 37
126	NSRP1	ENSG00000126653:28499560-28499616:target		39 | 45
127	RHOT1	ENSG00000126858:30535126-30535328:source		30 | 28
128	MKLN1	ENSG00000128585:131172366-131181395:target		23 | 22
129	MYO1B	ENSG00000128641:192265108-192265194:source		39 | 41
130	KNSTRN	ENSG00000128944:40678563-40679049:target		35 | 33
131	ARPP19	ENSG00000128989:52849297-52849941:source		29 | 32
132	CDC16	ENSG00000130177:115000362-115000607:source		22 | 22
133	MLLT4	ENSG00000130396:168314221-168314993:target		21 | 21
134	TRPM4	ENSG00000130529:49705221-49705398:source		32 | 32
135	AKAP12	ENSG00000131016:151669846-151674887:target		61 | 61
136	COQ3	ENSG00000132423:99828065-99828217:source		21 | 21
137	COQ3	ENSG00000132423:99831574-99831700:target		26 | 26
138	PPHLN1	ENSG00000134283:42768758-42769100:source		36 | 36
139	STK26	ENSG00000134602:131202440-131202597:source		20 | 20
140	CARS2	ENSG00000134905:111296732-111297980:target		21 | 21
141	SNX14	ENSG00000135317:86246510-86246742:source		25 | 25
142	ORC3	ENSG00000135336:88304071-88304125:source		21 | 6
143	MAP3K7	ENSG00000135341:91246056-91246937:source		23 | 23
144	REPS1	ENSG00000135597:139251114-139251235:target		20 | 12
145	DHX9	ENSG00000135829:182811680-182811812:source		35 | 35

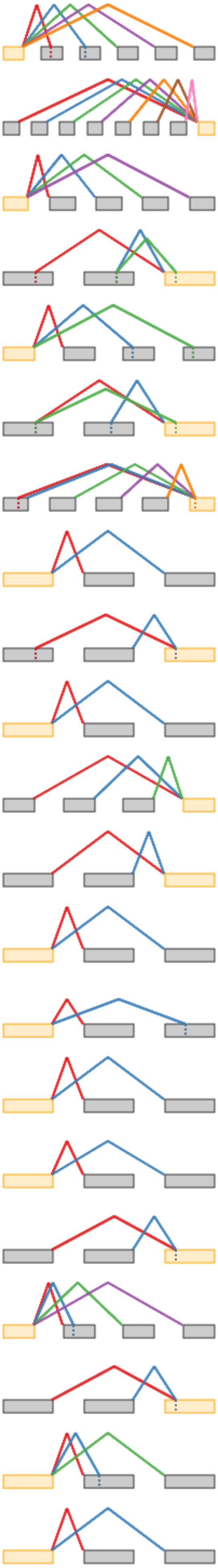

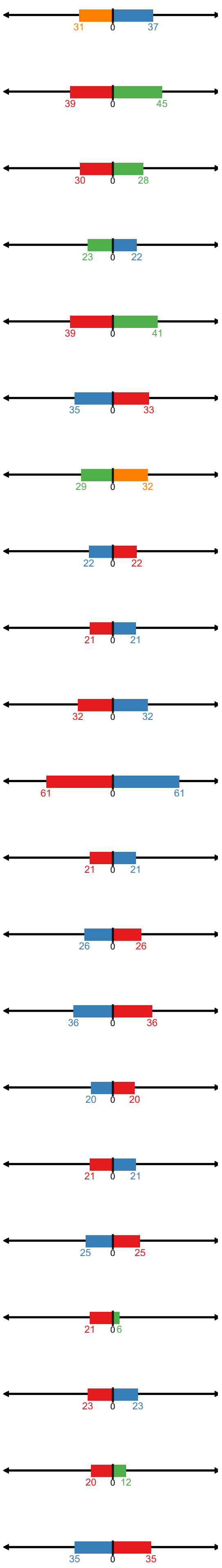

Gene LSV ID LSV Type ← More in DKO | More in WT →

LSV filters

- [x] 5-prime
- [x] 3-prime
- [x] Exon skipping
- [x] Single Source
- [x] Single Target

Number of junctions:
from:
to:

Number of exons:
from:
to:

#	Gene	LSV ID	← More in DKO | More in WT →
146	DHX9	ENSG00000135829:182821368-182821479:target	35 0 177
147	EIF4E2	ENSG00000135930:233431788-233432730:source	42 0 34
148	FLNB	ENSG00000136068:58128377-58128479:target	45 0 36
149	TRA2B	ENSG00000136527:185644389-185646861:source	31 0 31
150	TRA2B	ENSG00000136527:185655613-185655924:target	30 0 25
151	DNAJC1	ENSG00000136770:22217969-22218070:target	26 0 42
152	NUMA1	ENSG00000137497:71721832-71721900:source	43 0 43
153	NUMA1	ENSG00000137497:71723941-71725082:target	31 0 31
154	SLTM	ENSG00000137776:59193167-59193486:target	25 0 267
155	KIF23	ENSG00000137807:69718214-69718521:target	25 0 25
156	RABGGTB	ENSG00000137955:76253181-76254468:target	31 0 165
157	RABGGTB	ENSG00000137955:76255637-76255798:source	25 0 25
158	CENPO	ENSG00000138092:25042223-25045245:target	51 0 12 27
159	RPS24	ENSG00000138326:79796921-79797062:source	23 0 22
160	RPS24	ENSG00000138326:79800373-79800471:target	22 0 22
161	VWA9	ENSG00000138614:65903436-65903627:target	20 0 15
162	FGF2	ENSG00000138685:123813366-123819391:target	49 0 49
163	NUP54	ENSG00000138750:77057339-77057565:source	26 0 26
164	RGS3	ENSG00000138835:116356710-116356811:target	85 0 85
165	TPM1	ENSG00000140416:63347928-63349317:target	54 0 55
166	ZSCAN32	ENSG00000140987:3447192-3447787:source	29 0 11

LSV Type

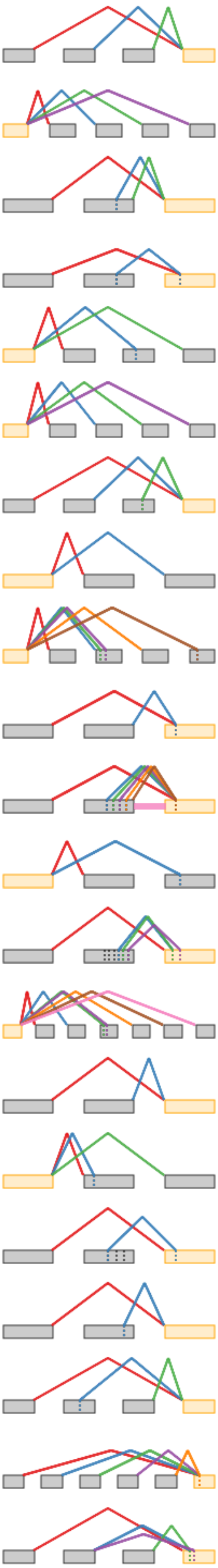

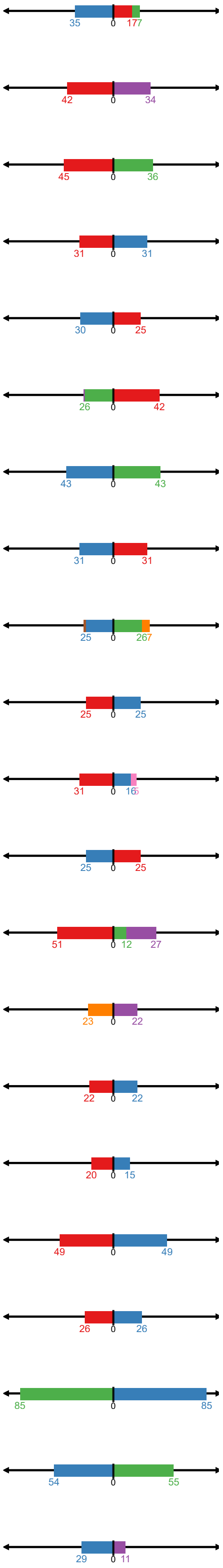

| # | Gene | LSV ID | LSV Type | ← More in DKO \| More in WT → |

LSV filters

- [x] 5-prime
- [x] 3-prime
- [x] Exon skipping
- [x] Single Source
- [x] Single Target

Number of junctions:
from:
to:

Number of exons:
from:
to:

#	Gene	LSV ID	LSV Type	← More in DKO | More in WT →
167	BCAS3	ENSG00000141376:58967056-58967103:target	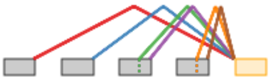	36 0 33
168	TRIM65	ENSG00000141569:73885041-73887428:source	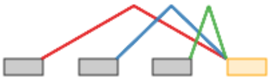	34 0 37
169	MBD1	ENSG00000141644:47795211-47796455:source	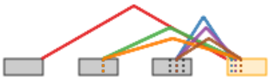	0 27
170	APP	ENSG00000142192:27354657-27354790:source	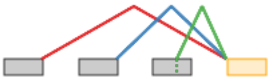	0 21
171	EMP3	ENSG00000142227:48828582-48828867:source	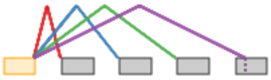	26 0 33
172	KIF2C	ENSG00000142945:45215981-45216236:source	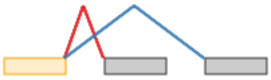	25 0 25
173	PTPRF	ENSG00000142949:44003869-44003948:target	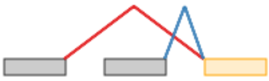	52 0 52
174	GULP1	ENSG00000144366:189248490-189248616:target	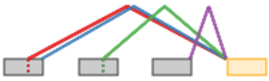	43 0 35
175	UBA3	ENSG00000144744:69112590-69112654:target	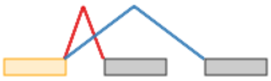	24 0 24
176	PHLDB2	ENSG00000144824:111639129-111639266:source	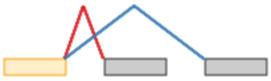	25 0 25
177	CBR4	ENSG00000145439:169922320-169923356:target	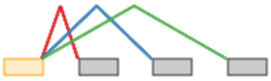	17 0 23
178	DDX46	ENSG00000145833:134152120-134152296:source	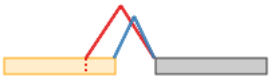	31 0 31
179	RPL7L1	ENSG00000146223:42851057-42851514:target	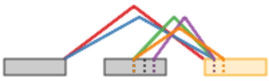	14 0 25
180	ZMYM4	ENSG00000146463:35790961-35791006:target	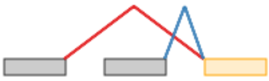	63 0 63
181	RPL10	ENSG00000147403:153628144-153628507:source	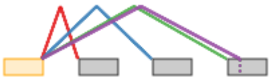	22 0 22
182	SLC25A37	ENSG00000147454:23386318-23386725:source	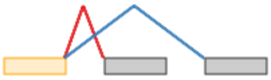	27 0 27
183	WHSC1L1	ENSG00000147548:38176413-38176809:target	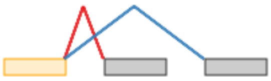	26 0 26
184	TATDN1	ENSG00000147687:125551169-125551699:target	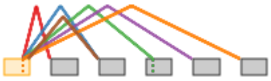	9 0 20
185	MKI67	ENSG00000148773:129900843-129907687:source	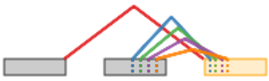	0 21
186	HYOU1	ENSG00000149428:118926782-118926879:source	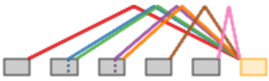	32 0 12
187	ALDOA	ENSG00000149925:30078555-30078687:target	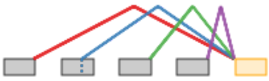	41 0 43

| # | Gene | LSV ID | LSV Type | ← More in DKO \| More in WT → |

LSV filters

- [x] 5-prime
- [x] 3-prime
- [x] Exon skipping
- [x] Single Source
- [x] Single Target

Number of junctions:
from:
to:

Number of exons:
from:
to:

#	Gene	LSV ID	LSV Type	← More in DKO | More in WT →
188	THYN1	ENSG00000151500:134118173-134118378:source	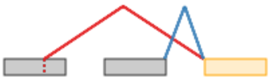	21 0 21
189	THYN1	ENSG00000151500:134118933-134119156:target	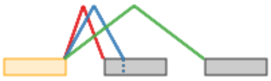	31 0 37
190	DST	ENSG00000151914:56330876-56330993:source	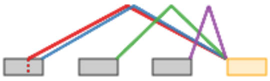	43 0 43
191	DST	ENSG00000151914:56334663-56334755:target	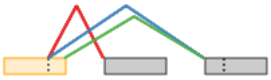	38 0 38
192	TIAL1	ENSG00000151923:121347664-121347760:target	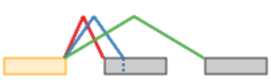	21 0 24
193	MZT2B	ENSG00000152082:130948042-130948302:target	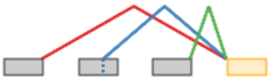	23 0 22
194	ATG10	ENSG00000152348:81571964-81572676:target	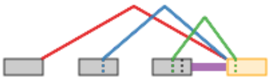	24 0 24
195	XRCC4	ENSG00000152422:82400727-82400877:target	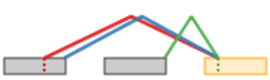	43 0 34
196	FAM49B	ENSG00000153310:130883621-130883742:source	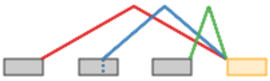	26 0 26
197	TBRG1	ENSG00000154144:124495567-124495799:source	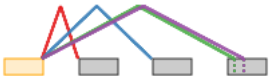	32 0 32
198	BUB3	ENSG00000154473:124922128-124922757:source	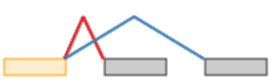	37 0 37
199	DPH3	ENSG00000154813:16306102-16306479:target	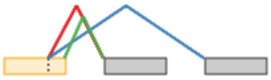	29 0 34
200	RHOC	ENSG00000155366:113246115-113246428:source	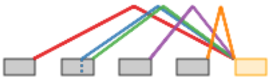	22 0 11
201	FAM122B	ENSG00000156504:133921502-133921563:target	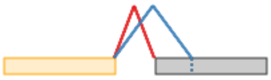	47 0 47
202	DHRS4	ENSG00000157326:24429111-24429212:source	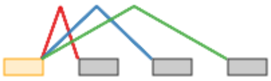	21 0 21
203	RNF111	ENSG00000157450:59193459-59193486:target	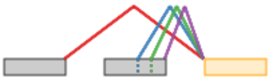	28 0 7 27
204	WIPI2	ENSG00000157954:5229819-5230124:source	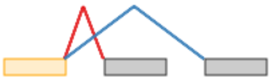	22 0 22
205	ADAR	ENSG00000160710:154574861-154575102:source	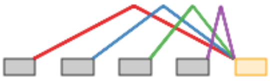	40 0 42
206	DMKN	ENSG00000161249:35988122-35988440:source	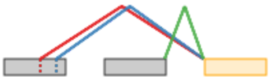	11 0 32
207	DMKN	ENSG00000161249:35996620-35996667:target	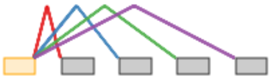	14 0 20
208	DMKN	ENSG00000161249:35996841-35996974:source	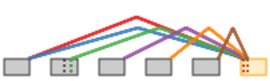	26 0 12

| # | Gene | LSV ID | LSV Type | ← More in DKO \| More in WT → |

LSV filters

- [x] 5-prime
- [x] 3-prime
- [x] Exon skipping
- [x] Single Source
- [x] Single Target

Number of junctions:
from:
to:

Number of exons:
from:
to:

#	Gene	LSV ID	← More in DKO	More in WT →
209	SMARCAD1	ENSG00000163104:95129497-95129735:target	35	35
210	ENSG00000163486	ENSG00000163486:206634382-206637783:target	42	42
211	SNHG16	ENSG00000163597:74553848-74553939:source	16	25
212	SLMAP	ENSG00000163681:57850969-57851019:source	40	40
213	MFI2	ENSG00000163975:196748165-196748342:target	28	28
214	TRA2A	ENSG00000164548:23571408-23571660:target	31	36
215	PEX2	ENSG00000164751:77898423-77898541:source	38	39
216	NUDT2	ENSG00000164978:34329504-34329597:source	31	31
217	NUDT2	ENSG00000164978:34339022-34339164:target	40	40
218	METTL2B	ENSG00000165055:128119212-128119567:target	30	31
219	ARHGAP12	ENSG00000165322:32120667-32120728:source	32	38
220	ARHGAP12	ENSG00000165322:32150323-32150586:target	37	37
221	FAM204A	ENSG00000165669:120101782-120101840:target	43	43
222	ENSG00000166008	ENSG00000166008:148867808-148869397:target	20	20
223	BTRC	ENSG00000166167:103113820-103113985:source	28	27
224	ILK	ENSG00000166333:6625410-6625956:source	24	29
225	ILK	ENSG00000166333:6629496-6629719:target	24	24
226	CASC4	ENSG00000166734:44705534-44705741:target	24	24
227	KIAA0101	ENSG00000166803:64657193-64658274:source	29	29
228	KIAA0101	ENSG00000166803:64673157-64673354:target	31	31
229	VPS39	ENSG00000166887:42483459-42483758:source	36	36

LSV Type

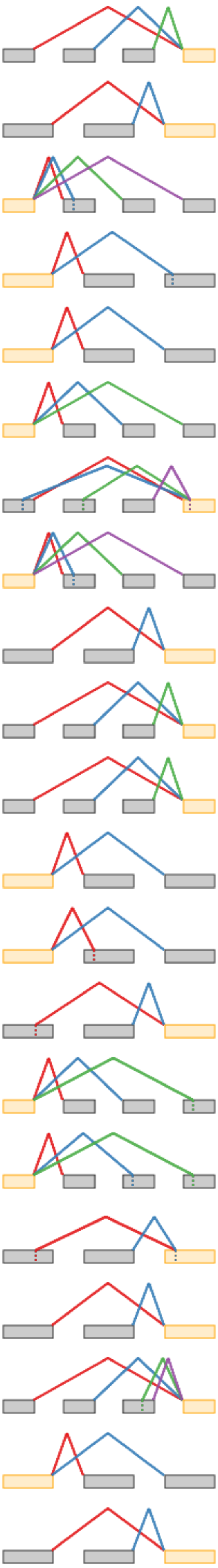

| Gene | LSV ID | LSV Type | ← More in DKO | More in WT →

LSV filters

- [x] 5-prime
- [x] 3-prime
- [x] Exon skipping
- [x] Single Source
- [x] Single Target

Number of junctions:
from:
to:

Number of exons:
from:
to:

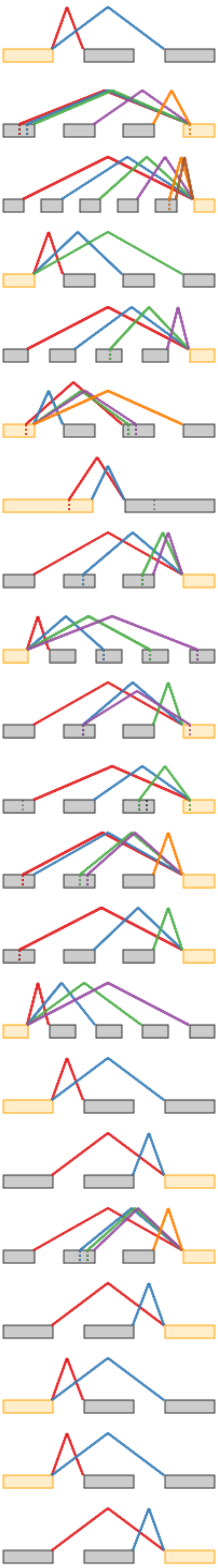

#	Gene	LSV ID	← More in DKO | More in WT →
230	VPS39	ENSG00000166887:42492094-42492159:target	45 0 45
231	ZNF146	ENSG00000167635:36719361-36719721:target	27 0 9
232	NXN	ENSG00000167693:728806-729318:source	28 0 34
233	SDHAF2	ENSG00000167985:61197514-61197654:source	23 0 11
234	SDHAF2	ENSG00000167985:61213413-61215001:target	21 0 13
235	CTNNB1	ENSG00000168036:41280624-41280845:source	25 0 24
236	CDKN2AIP	ENSG00000168564:184366688-184367174:source	31 0 31
237	TSPAN5	ENSG00000168785:99403156-99403326:source	20 0 22
238	MFF	ENSG00000168958:228205008-228205277:source	20 0 24
239	E2F6	ENSG00000169016:11593708-11593924:source	10 0 29
240	GTSF1	ENSG00000170627:54856955-54857117:source	21 0 8
241	CHCHD7	ENSG00000170791:57127157-57128137:target	36 0 26
242	NDUFA3	ENSG00000170906:54609241-54609318:target	15 0 30
243	FAM86JP	ENSG00000171084:125639668-125639748:source	25 0 5
244	ZDHHC16	ENSG00000171307:99213375-99213420:source	22 0 22
245	ZDHHC16	ENSG00000171307:99214471-99214556:target	31 0 31
246	MRPL36	ENSG00000171421:1798500-1799061:source	21 0 17
247	CLSTN1	ENSG00000171603:9795943-9796100:source	24 0 24
248	CLSTN1	ENSG00000171603:9801152-9801314:target	21 0 21
249	MALT1	ENSG00000172175:56377208-56377304:source	38 0 38
250	MALT1	ENSG00000172175:56381315-56381341:target	53 0 53

Gene LSV ID LSV Type ← More in DKO | More in WT →

LSV filters

- [x] 5-prime
- [x] 3-prime
- [x] Exon skipping
- [x] Single Source
- [x] Single Target

Number of junctions:
from:
to:

Number of exons:
from:
to:

#	Gene	LSV ID	LSV Type	← More in DKO | More in WT →
251	MIR4435-1HG	ENSG00000172965:112186886-112188485:source		17 0 20
252	AHSA2	ENSG00000173209:61413581-61413632:target		21 0 20
253	CEP83	ENSG00000173588:94794626-94794897:source		56 0 56
254	CEP83	ENSG00000173588:94805473-94805623:target		22 0 22
255	GOLIM4	ENSG00000173905:167754624-167754782:source		32 0 32
256	GOLIM4	ENSG00000173905:167758906-167759262:target		29 0 29
257	STAG3L3	ENSG00000174353:72473544-72473660:target		35 0 35
258	ARL10	ENSG00000175414:175815236-175815974:source		31 0 31
259	DLEU1	ENSG00000176124:50656307-50656693:source		39 0 40
260	SEC24C	ENSG00000176986:75504120-75504195:source		20 0 21
261	CHID1	ENSG00000177830:910641-911461:target		40 0 40
262	MLF1	ENSG00000178053:158288952-158289180:source		33 0 33
263	MLF1	ENSG00000178053:158310223-158310370:target		5 25 0 37
264	MPI	ENSG00000178802:75183720-75183920:target		31 0 31
265	PUF60	ENSG00000179950:144911450-144912029:target		13 0 22
266	RCC1	ENSG00000180198:28856370-28856451:target		29 0 16
267	HIGD1A	ENSG00000181061:42835649-42835791:source		23 0 23
268	HIGD1A	ENSG00000181061:42845928-42846023:target		22 0 24
269	CHST15	ENSG00000182022:125801817-125801963:target		40 0 40
270	ANXA2	ENSG00000182718:60689454-60689537:target		19 0 20
271	NAA38	ENSG00000183011:7760303-7760581:source		21 0 23

Gene LSV ID LSV Type ← More in DKO | More in WT →

LSV filters

- [x] 5-prime
- [x] 3-prime
- [x] Exon skipping
- [x] Single Source
- [x] Single Target

Number of junctions:
from:
to:

Number of exons:
from:
to:

#	Gene	LSV ID	LSV Type	← More in DKO | More in WT →
272	HMCES	ENSG00000183624:128998553-128998758:target	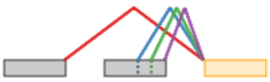	11 0 20
273	UPP1	ENSG00000183696:48134360-48134424:source	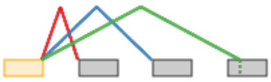	54 0 40 9
274	AC138969.4	ENSG00000183889:16427504-16427741:source	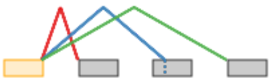	8 0 28
275	UQCR10	ENSG00000184076:30163358-30163596:source	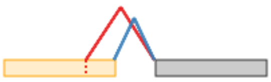	30 0 30
276	NR2C2AP	ENSG00000184162:19312867-19313207:target	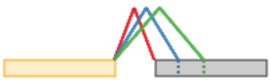	26 0
277	UBE2F	ENSG00000184182:238903386-238903451:source	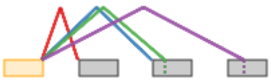	24 0 18
278	APOO	ENSG00000184831:23858430-23858522:target	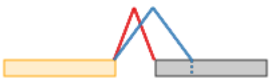	35 0 35
279	AP3M1	ENSG00000185009:75897865-75898140:source	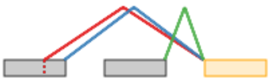	26 0 25
280	AP3M1	ENSG00000185009:75910093-75910821:target	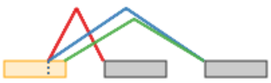	26 0 26
281	CDK10	ENSG00000185324:89753076-89753205:source	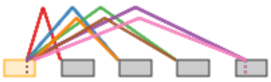	35 0 20
282	CDK10	ENSG00000185324:89755660-89755732:target	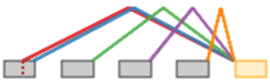	13 0 28
283	CDK10	ENSG00000185324:89755911-89757032:target	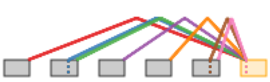	24 0 12
284	ENSG00000185928	ENSG00000185928:29830876-29831831:source	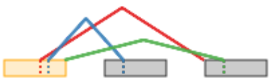	47 0 42
285	RNF220	ENSG00000187147:45097907-45098057:target	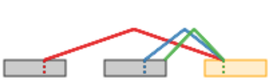	21 0 15
286	KIAA1598	ENSG00000187164:118738767-118738819:source	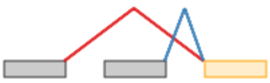	20 0 20
287	BLOC1S5	ENSG00000188428:8026600-8026658:source	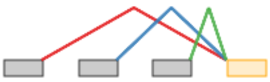	27 0 7
288	ALKBH2	ENSG00000189046:109530060-109530742:target	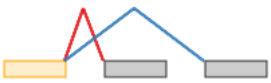	21 0 21
289	SPATS2L	ENSG00000196141:201194154-201194203:target	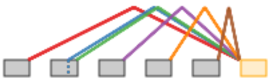	25 0 26
290	HIATL2	ENSG00000196312:99711837-99711964:target	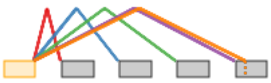	0 21
291	ZNF565	ENSG00000196357:36719650-36719721:target	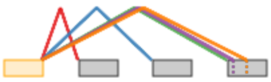	27 0 9
292	AMZ2	ENSG00000196704:66244645-66244846:target	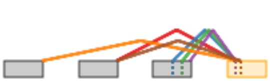	17 0 20
#	Gene	LSV ID	LSV Type	← More in DKO | More in WT →

LSV filters

- [x] 5-prime
- [x] 3-prime
- [x] Exon skipping
- [x] Single Source
- [x] Single Target

Number of junctions:
from:
to:

Number of exons:
from:
to:

#	Gene	LSV ID	LSV Type	← More in DKO | More in WT →
293	AP2A1	ENSG00000196961:50306206-50306262:target		31 0 31
294	ANXA6	ENSG00000197043:150489314-150489407:source		32 0 32
295	ANXA6	ENSG00000197043:150496688-150496741:target		45 0 45
296	PCBP2	ENSG00000197111:53835525-53835584:source		26 0 26
297	SPTAN1	ENSG00000197694:131356454-131356652:target		31 0 31
298	FAM118B	ENSG00000197798:126104890-126104982:target		26 0 23
299	SNX29P2	ENSG00000198106:29465337-29465434:target		24 0 18
300	TPM2	ENSG00000198467:35684485-35684547:source		43 0 43
301	TPM2	ENSG00000198467:35685142-35685336:target		49 0 49
302	DDX39B	ENSG00000198563:31508099-31508441:source		35 0 32
303	DDX39B	ENSG00000198563:31509727-31510225:target		33 0 33
304	TLK1	ENSG00000198586:171884849-171884915:source		20 0 21
305	APRT	ENSG00000198931:88876478-88876806:target		31 0 31
306	BAG6	ENSG00000204463:31607976-31608083:target		32 0 32
307	PRR3	ENSG00000204576:30527808-30529285:target		23 0 23
308	C12orf73	ENSG00000204954:104350082-104350526:target		26 0 21
309	PRR13	ENSG00000205352:53836479-53837061:target		29 0 17
310	CSNK1E	ENSG00000213923:38694586-38694939:target		0 22
311	DHRS4-AS1	ENSG00000215256:24429111-24429212:source		21 0 21
312	RPL17-C18orf32	ENSG00000215472:47017996-47018203:target		45 6 0 30 5 10
313	SNHG14	ENSG00000224078:25599500-25599573:source		22 0 22

| # | Gene | LSV ID | LSV Type | ← More in DKO \| More in WT → |

LSV filters

- [x] 5-prime
- [x] 3-prime
- [x] Exon skipping
- [x] Single Source
- [x] Single Target

Number of junctions:
from:
to:

Number of exons:
from:
to:

#	Gene	LSV ID	LSV Type	← More in DKO | More in WT →
314	SNHG14	ENSG00000224078:25601039-25601203:target		53 0 53
315	PET117	ENSG00000232838:18118517-18119088:source		23 0 23
316	RP4-694A7.2	ENSG00000233589:68944812-68945003:source		41 0 257
317	CTA-256D12.11	ENSG00000234208:29925151-29925228:source		30 0 30
318	AC004967.7	ENSG00000243554:97599082-97599157:source		48 0 48
319	N4BP2L2	ENSG00000244754:33101012-33101669:source		45 0 9 16
320	N4BP2L2	ENSG00000244754:33101012-33101669:target		22 0 22
321	AC002116.8	ENSG00000248101:36501469-36501979:source		21 0 33
322	SMG1P6	ENSG00000254634:29465337-29465435:target		25 0 18
323	ATP6V1G2-DDX39B	ENSG00000254870:31509727-31509925:target		33 0 33
324	APOPT1	ENSG00000256053:104037960-104038157:source		33 0 34
325	RP11-73M18.2	ENSG00000256500:104029299-104029716:source		31 0
326	RP11-73M18.2	ENSG00000256500:104037960-104038157:source		34 0 34
327	POLG2	ENSG00000256525:62479036-62479116:target		35 0 31
328	RP11-286N22.8	ENSG00000256591:61197386-61197654:source		22 0 11
329	RP11-793H13.8	ENSG00000257379:53835525-53835584:source		26 0 26
330	PPT2-EGFL8	ENSG00000258388:32138192-32139334:source		29 0 29
331	PPT2-EGFL8	ENSG00000258388:32138192-32139334:target		31 0 18
332	UBE2F-SCLY	ENSG00000258984:238903386-238903451:source		23 0 18
333	BLOC1S5-TXNDC5	ENSG00000259040:8026600-8026658:source		28 0 7
334	CSPG4P12	ENSG00000259295:85750933-85757136:target		25 0 24

Gene LSV ID LSV Type ← More in DKO | More in WT →

LSV filters

- [x] 5-prime
- [x] 3-prime
- [x] Exon skipping
- [x] Single Source
- [x] Single Target

Number of junctions:
from:
to:

Number of exons:
from:
to:

#	Gene	LSV ID	LSV Type	← More in DKO | More in WT →
335	RP11-430B1.2	ENSG00000259577:52497062-52498071:target	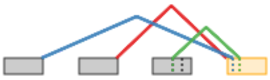	48 0 45
336	HERC2P5	ENSG00000260644:32779596-32779783:source	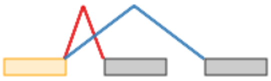	61 0 61
337	RP11-345J4.5	ENSG00000261740:29463430-29465434:target	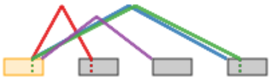	23 0 18
338	MIR1539	ENSG00000265496:47017996-47018248:target	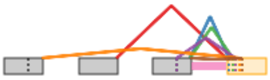	29 0 6 20
339	ENSG00000265500	ENSG00000265500:7470244-7470322:target	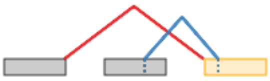	30 0 30
340	EEF1E1-BLOC1S5	ENSG00000265818:8026600-8026658:source	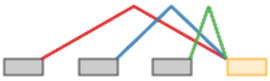	27 0 7
341	RP11-159D12.5	ENSG00000266086:56082284-56082614:target	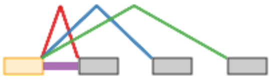	38 0 29 8
342	STRADA	ENSG00000266173:61791366-61791914:source	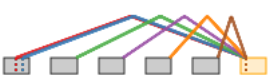	28 0 23
343	AC002398.11	ENSG00000267439:36242862-36244316:target	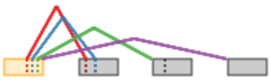	36 0 31
344	RP11-126O1.4	ENSG00000267476:56377208-56377304:source	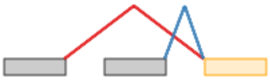	38 0 38
345	RP11-126O1.4	ENSG00000267476:56381315-56381341:target	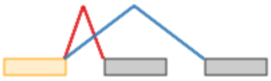	52 0 52
346	CTD-2587H24.5	ENSG00000267577:55670790-55671381:source	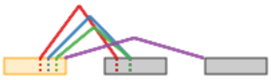	31 0 17
347	CTD-2587H24.5	ENSG00000267577:55670790-55671381:target	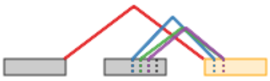	31 0 17
348	RP1-283E3.8	ENSG00000268575:1654027-1654270:source	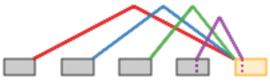	26 0 26
349	SNHG8	ENSG00000269893:119200089-119200292:target	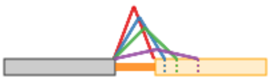	45 0 28 13
350	RP11-426L16.10	ENSG00000271810:113246266-113246428:source	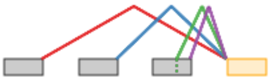	22 0 10
351	LINC01578	ENSG00000272888:93425937-93426416:source	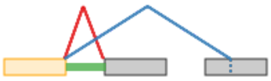	21 0 20
352	RP11-201K10.3	ENSG00000273088:155145041-155145368:source	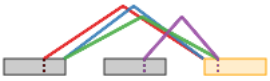	21 0 27
353	RP11-212P7.3	ENSG00000273184:128119212-128119567:target	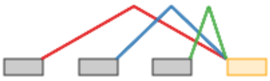	32 0 31

Gene LSV ID LSV Type ← More in DKO | More in WT →

1/1 All

© BIOCIPHERS · Department of Genetics Perelman School of Medicine Department of Computer and Information Science School of Engineering University of Pennsylvania Penn

3) Differential alternative RNA events in DKO cells compared to the parental HCT116 cells in all RNA combined.

LSV ΔΨ Index: DKO Vs WT

#	Gene	LSV ID	LSV Type	← More in DKO | More in WT →
0	SPATA20	ENSG00000006282:48627569-48627673:target	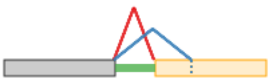	24 0 24
1	NFIX	ENSG00000008441:13183861-13183923:target	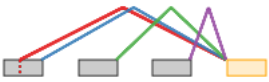	23 0 23
2	STARD3NL	ENSG00000010270:38254629-38254706:source	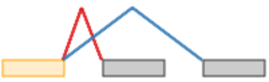	49 0 49
3	STARD3NL	ENSG00000010270:38256789-38256906:target	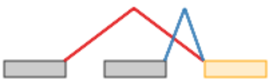	52 0 52
4	PHLDB1	ENSG00000019144:118521306-118526400:target	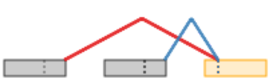	20 0 20
5	NLRP2	ENSG00000022556:55485723-55485912:target	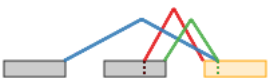	21 0 21
6	AKAP11	ENSG00000023516:42888030-42888076:target	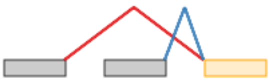	21 0 21
7	DEPDC1	ENSG00000024526:68944827-68945003:source	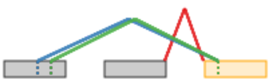	23 0 17
8	CD44	ENSG00000026508:35211557-35211976:source	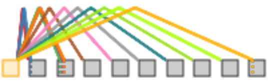	27 5 0 10 12
9	CD44	ENSG00000026508:35232793-35232996:target	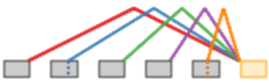	23 0 24
10	CD44	ENSG00000026508:35236213-35236461:target	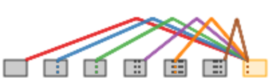	26 0 23
11	ARNTL2	ENSG00000029153:27538414-27538493:source	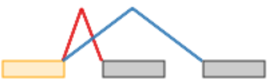	21 0 21
12	MAT2B	ENSG00000038274:162939008-162939406:target	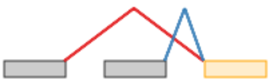	38 0 38
13	FAM65A	ENSG00000039523:67571981-67572453:target	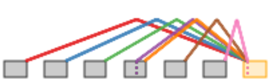	15 0 27
14	DTNBP1	ENSG00000047579:15637975-15638035:source	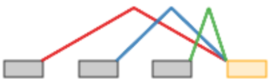	15 0 25
15	NEDD4L	ENSG00000049759:55989657-55989718:source	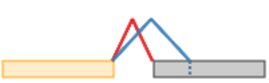	47 0 47
16	PUM2	ENSG00000055917:20482708-20482992:target	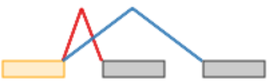	22 0 22
17	CROCC	ENSG00000058453:17266389-17266588:target	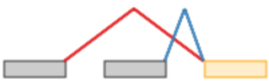	45 0 45
18	SBNO2	ENSG00000064932:1109134-1109210:source	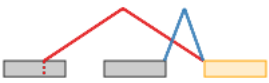	22 0 22

Gene LSV ID LSV Type ← More in DKO | More in WT →

#	Gene	LSV ID	LSV Type	← More in DKO | More in WT →
19	SBNO2	ENSG00000064932:1109500-1109597:target		27 0 27
20	SLK	ENSG00000065613:105767935-105768114:source		29 0 29
21	SLK	ENSG00000065613:105777918-105778047:target		25 0 25
22	NGEF	ENSG00000066248:233752743-233752817:source		53 0 53
23	RRP15	ENSG00000067533:218458629-218458797:source		64 0 64
24	SMG6	ENSG00000070366:1972072-1972225:source		20 0 20
25	NCK2	ENSG00000071051:106471504-106471745:target		23 0 24
26	PRKACA	ENSG00000072062:14218160-14218221:source		35 0 34
27	DERL2	ENSG00000072849:5383374-5383593:source		20 0 95
28	SELO	ENSG00000073169:50644746-50644949:target		34 0 34
29	PICALM	ENSG00000073921:85687666-85688048:source		31 0 31
30	PICALM	ENSG00000073921:85692172-85692271:target		20 0 29
31	ZNF638	ENSG00000075292:71582849-71582910:target		21 0 6
32	ARHGEF1	ENSG00000076928:42410091-42410487:source		20 0 20
33	DNM2	ENSG00000079805:10906048-10906422:source		23 0 23
34	DNM2	ENSG00000079805:10909063-10909248:target		23 0 23
35	DNM2	ENSG00000079805:10916592-10916643:source		20 0 20
36	DNM2	ENSG00000079805:10922940-10923053:target		22 0 22
37	SRCAP	ENSG00000080603:30711229-30711302:target		34 0 14
38	APLP2	ENSG00000084234:129992200-129992408:source		21 0 21
39	APLP2	ENSG00000084234:129996595-129996725:target		21 0 21
#	Gene	LSV ID	LSV Type	← More in DKO | More in WT →

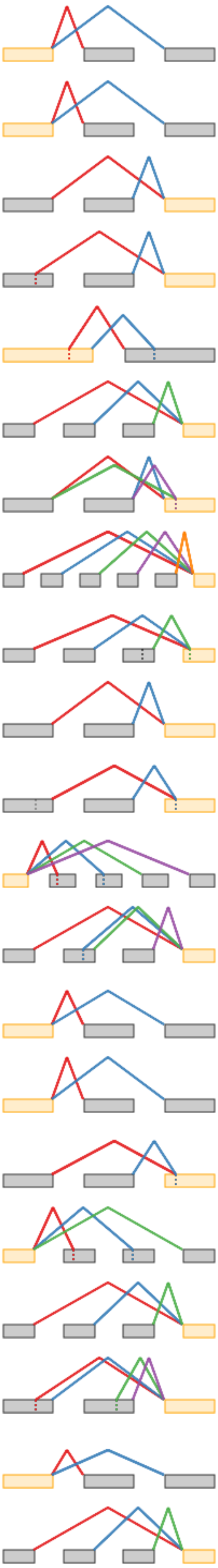

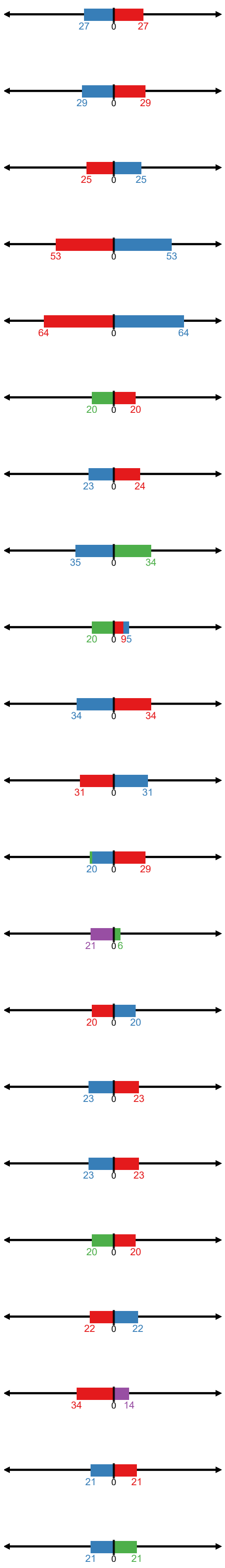

#	Gene	LSV ID	LSV Type	← More in DKO | More in WT →
40	KHSRP	ENSG00000088247:6414468-6415312:target	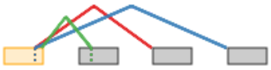	23 0 29
41	RAB11FIP3	ENSG00000090565:546824-547158:target	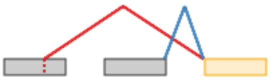	35 0 35
42	MYL6	ENSG00000092841:56554027-56554127:source	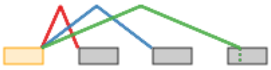	24 0 22
43	LRRFIP2	ENSG00000093167:37125127-37125297:source	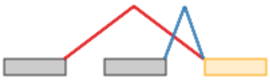	30 0 30
44	LRRFIP2	ENSG00000093167:37136283-37136399:target	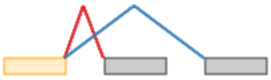	25 0 25
45	FAM21A	ENSG00000099290:51885817-51886268:source	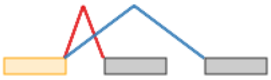	22 0 22
46	MYO9B	ENSG00000099331:17286472-17286550:source	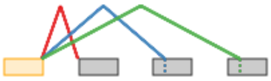	23 0 8
47	SBF1	ENSG00000100241:50894921-50895102:source	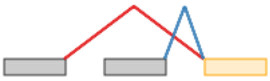	29 0 29
48	SBF1	ENSG00000100241:50897684-50897821:target	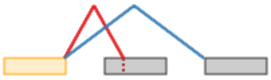	20 0 20
49	VTI1B	ENSG00000100568:68113792-68118198:source	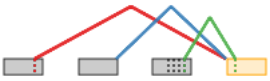	20 0 21
50	CCNB1IP1	ENSG00000100814:20784573-20784719:source	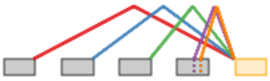	24 0 24
51	ACOT8	ENSG00000101473:44483798-44483931:target	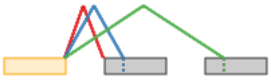	7 0 23
52	NXT2	ENSG00000101888:108781274-108781360:target	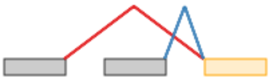	23 0 23
53	TAF1C	ENSG00000103168:84220507-84220669:target	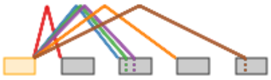	25 0 6
54	HAGHL	ENSG00000103253:778794-778985:source	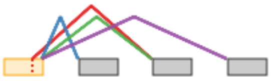	24 0 9
55	HAGHL	ENSG00000103253:779288-779733:target	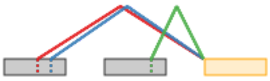	21 0 8
56	EEF1D	ENSG00000104529:144670998-144671486:target	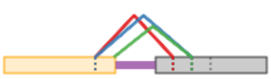	31 0 32
57	URI1	ENSG00000105176:30506443-30506611:target	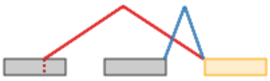	23 0 23
58	PLD3	ENSG00000105223:40872200-40872417:target	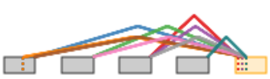	25 0 23
59	LMBR1	ENSG00000105983:156526555-156526951:target	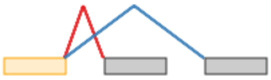	21 0 21

| # | Gene | LSV ID | LSV Type | ← More in DKO \| More in WT → |

#	Gene	LSV ID	LSV Type	← More in DKO | More in WT →
60	COA1	ENSG00000106603:43769028-43769316:target	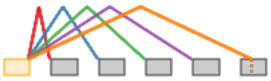	0 22
61	DVL1	ENSG00000107404:1274667-1274819:target	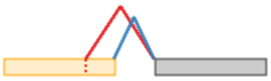	20 0 20
62	PLEKHA1	ENSG00000107679:124189140-124191867:target	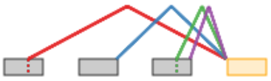	0 24
63	GALK1	ENSG00000108479:73753497-73754208:target	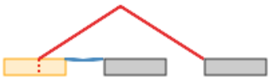	26 0 26
64	PTGES3L-AARSD1	ENSG00000108825:41106893-41106984:target	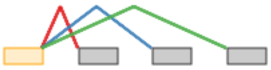	15 0 22
65	OCIAD1	ENSG00000109180:48834637-48834778:target	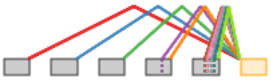	22 0 21
66	FRG1	ENSG00000109536:190878553-190878657:source	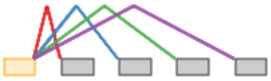	22 0 22
67	CLCN3	ENSG00000109572:170601201-170601358:target	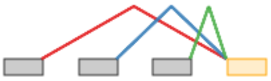	22 0 22
68	FOXM1	ENSG00000111206:2973849-2974020:source	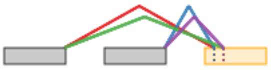	26 0 5 19
69	FOXM1	ENSG00000111206:2975559-2975687:target	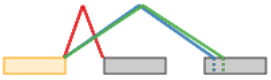	27 0 30
70	SOD2	ENSG00000112096:160169223-160169401:source	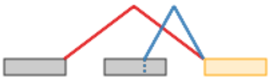	20 0 20
71	CEP72	ENSG00000112877:644253-644540:target	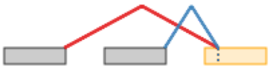	23 0 23
72	DROSHA	ENSG00000113360:31486598-31486669:target	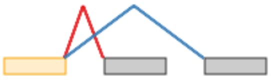	27 0 27
73	ECT2	ENSG00000114346:172473085-172473164:source	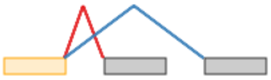	25 0 25
74	ECT2	ENSG00000114346:172474773-172474955:target	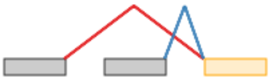	30 0 30
75	STK16	ENSG00000115661:220110614-220111146:target	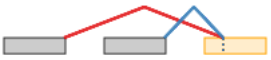	22 0 22
76	IVNS1ABP	ENSG00000116679:185275882-185276271:target	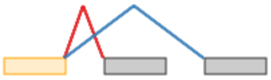	25 0 25
77	UAP1	ENSG00000117143:162560113-162560301:source	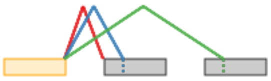	28 0 21
78	UAP1	ENSG00000117143:162567031-162567648:target	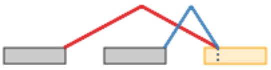	30 0 30
79	PRPF3	ENSG00000117360:150300234-150300925:target	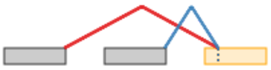	20 0 20

\# Gene LSV ID LSV Type ← More in DKO | More in WT →

#	Gene	LSV ID	LSV Type	← More in DKO | More in WT →
80	AKR1A1	ENSG00000117448:46016638-46016827:target	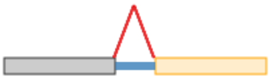	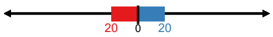
81	KMT2A	ENSG00000118058:118391981-118392132:target	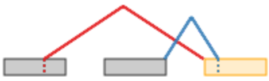	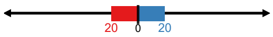
82	PPP2R4	ENSG00000119383:131904724-131904831:target	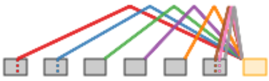	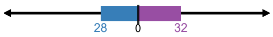
83	MAPKAP1	ENSG00000119487:128246722-128246862:source	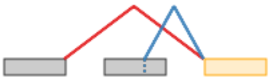	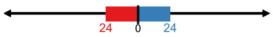
84	MAPKAP1	ENSG00000119487:128305338-128305447:target	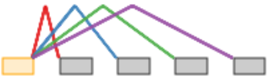	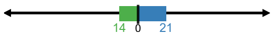
85	PKN1	ENSG00000123143:14551955-14552255:target	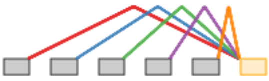	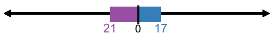
86	SRSF6	ENSG00000124193:42089343-42092245:target	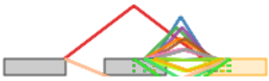	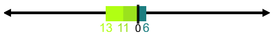
87	STX16	ENSG00000124222:57245568-57245659:target	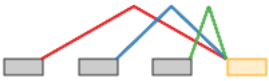	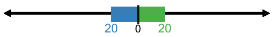
88	MYRF	ENSG00000124920:61533097-61533184:target	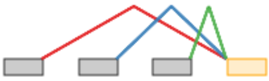	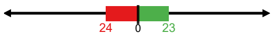
89	AHNAK	ENSG00000124942:62289319-62289417:target	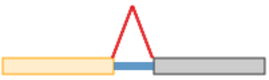	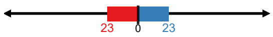
90	AHNAK	ENSG00000124942:62299672-62299682:target	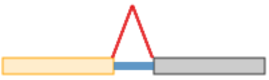	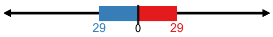
91	AHNAK	ENSG00000124942:62303941-62304039:source	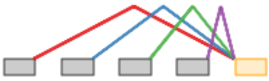	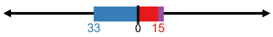
92	MACF1	ENSG00000127603:39929284-39930581:source	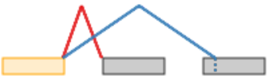	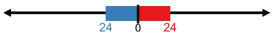
93	MACF1	ENSG00000127603:39934265-39934404:target	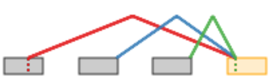	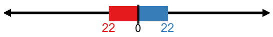
94	MYO1B	ENSG00000128641:192265108-192265194:source	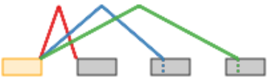	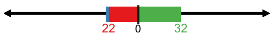
95	MYO1B	ENSG00000128641:192272384-192272915:target	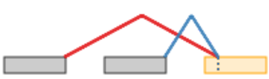	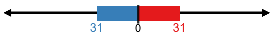
96	ARPP19	ENSG00000128989:52861309-52861436:target	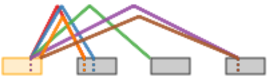	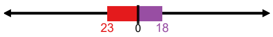
97	SAT1	ENSG00000130066:23801917-23802330:source	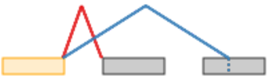	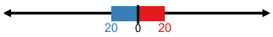
98	LAMA5	ENSG00000130702:60898339-60898726:target	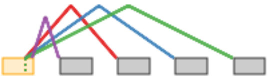	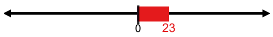
99	DNMT1	ENSG00000130816:10305496-10305811:target	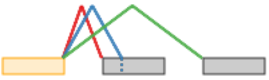	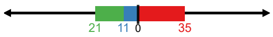

| # | Gene | LSV ID | LSV Type | ← More in DKO \| More in WT → |

#	Gene	LSV ID	LSV Type	← More in DKO | More in WT →
100	AKAP12	ENSG00000131016:151669846-151674887:target		68 0 69
101	DIAPH1	ENSG00000131504:140966609-140966764:source		24 0 24
102	DIAPH1	ENSG00000131504:140998365-140998622:target		25 0 25
103	RAF1	ENSG00000132155:12627180-12627404:target		21 0 21
104	COQ3	ENSG00000132423:99831574-99831700:target		21 0 21
105	UNK	ENSG00000132478:73780809-73781065:source		38 0 33
106	SDF2	ENSG00000132581:26982305-26983340:source		19 0 20
107	ARHGEF11	ENSG00000132694:156907041-156907288:source		26 0 26
108	ARHGEF11	ENSG00000132694:156909340-156909702:target		30 0 30
109	ISCA1	ENSG00000135070:88886922-88887027:source		20 0 20
110	DMTF1	ENSG00000135164:86824347-86825653:target		27 0 27
111	MAP3K7	ENSG00000135341:91246056-91246937:source		22 0 22
112	DHX9	ENSG00000135829:182811680-182811812:source		26 0 26
113	DHX9	ENSG00000135829:182821368-182821479:target		25 0 17
114	FLNB	ENSG00000136068:58124009-58124256:source		42 0 42
115	FLNB	ENSG00000136068:58128377-58128479:target		43 0 11 31
116	VEZF1	ENSG00000136451:56060271-56060754:source		20 0 23
117	UGGT1	ENSG00000136731:128914826-128914920:target		20 0 20
118	DNAJC1	ENSG00000136770:22217969-22218070:target		25 0 29
119	NUMA1	ENSG00000137497:71721832-71721900:source		22 6 0 31
#	Gene	LSV ID	LSV Type	← More in DKO | More in WT →

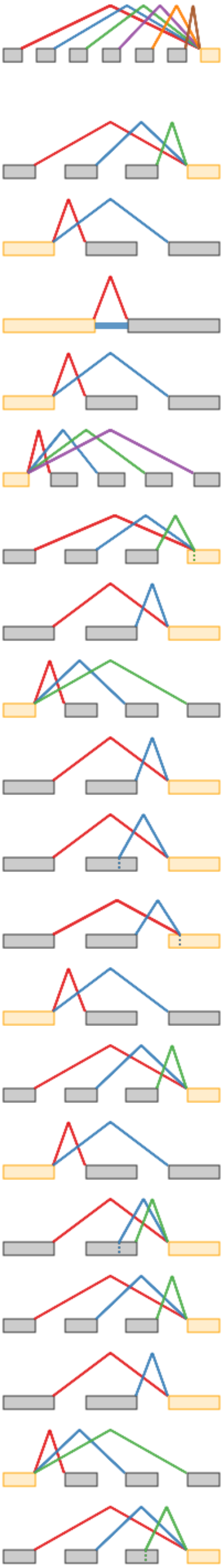

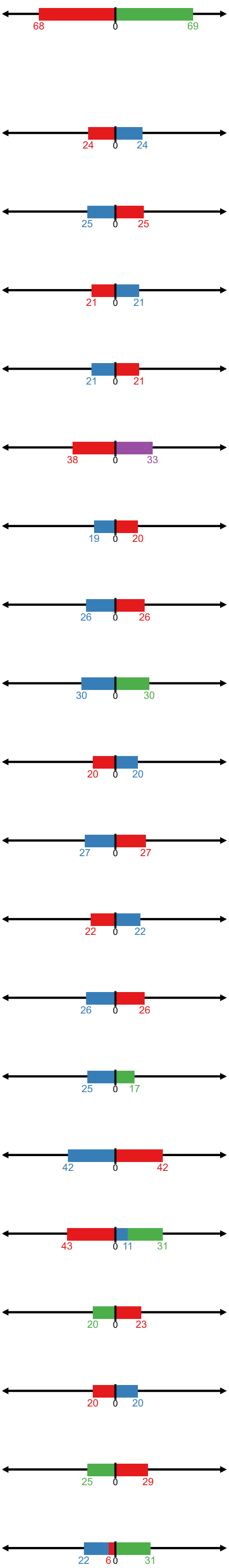

#	Gene	LSV ID	LSV Type	← More in DKO | More in WT →
120	NUMA1	ENSG00000137497:71723941-71725082:target	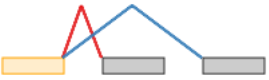	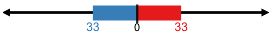 33 0 33
121	KIF23	ENSG00000137807:69718214-69718521:target	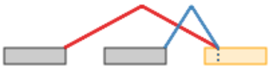	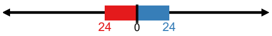 24 0 24
122	CENPO	ENSG00000138092:25042223-25045245:target	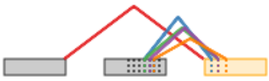	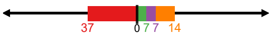 37 0 7 7 14
123	MYOF	ENSG00000138119:95148787-95148911:source	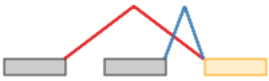	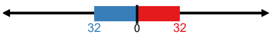 32 0 32
124	FGF2	ENSG00000138685:123813366-123819391:target	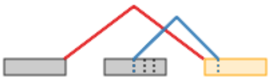	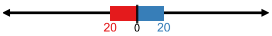 20 0 20
125	USO1	ENSG00000138768:76714843-76715054:source	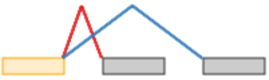	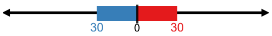 30 0 30
126	USO1	ENSG00000138768:76720775-76720885:target	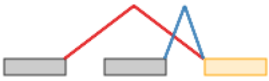	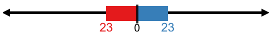 23 0 23
127	RGS3	ENSG00000138835:116356710-116356811:target	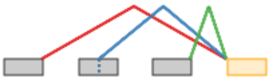	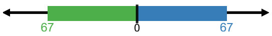 67 0 67
128	MAN2C1	ENSG00000140400:75649134-75649708:target	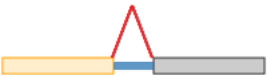	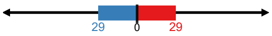 29 0 29
129	TPM1	ENSG00000140416:63347928-63349317:target	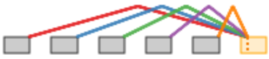	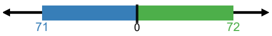 71 0 72
130	NCOR1	ENSG00000141027:16068185-16068475:target	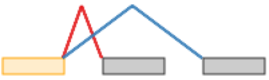	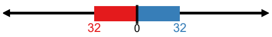 32 0 32
131	G6PC3	ENSG00000141349:42152048-42152138:source	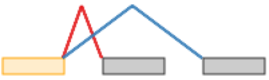	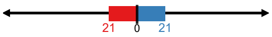 21 0 21
132	ARRB2	ENSG00000141480:4623511-4623594:source	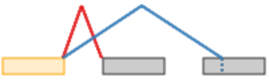	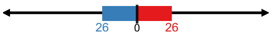 26 0 26
133	ARRB2	ENSG00000141480:4623845-4623935:target	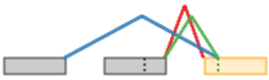	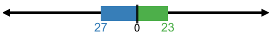 27 0 23
134	MBD1	ENSG00000141644:47798835-47799108:target	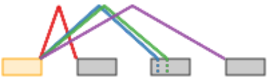	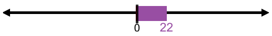 0 22
135	COL6A2	ENSG00000142173:47546417-47546455:source	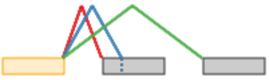	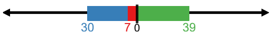 30 7 0 39
136	APP	ENSG00000142192:27354657-27354790:source	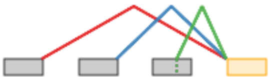	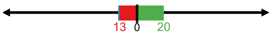 13 0 20
137	ADAM15	ENSG00000143537:155033239-155033308:source	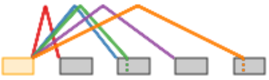	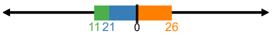 11 21 0 26
138	ADAM15	ENSG00000143537:155034637-155034845:target	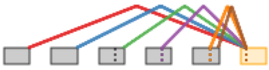	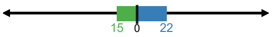 15 0 22
139	GULP1	ENSG00000144366:189248490-189248616:target	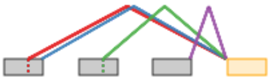	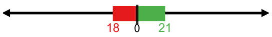 18 0 21

| # | Gene | LSV ID | LSV Type | ← More in DKO \| More in WT → |

#	Gene	LSV ID	LSV Type	← More in DKO | More in WT →
140	SLC25A26	ENSG00000144741:66419902-66419966:source	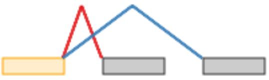	34 0 34
141	UBA3	ENSG00000144744:69111228-69111330:source	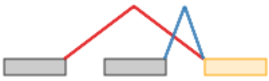	21 0 21
142	PHLDB2	ENSG00000144824:111632166-111636510:target	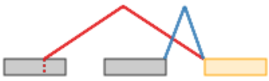	33 0 33
143	TNIP1	ENSG00000145901:150444521-150444692:source	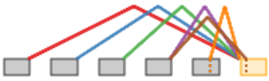	25 0 31
144	TMEM181	ENSG00000146433:159046735-159046786:source	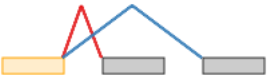	22 0 22
145	WTAP	ENSG00000146457:160169223-160170447:source	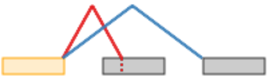	20 0 20
146	C7orf50	ENSG00000146540:1049586-1049779:source	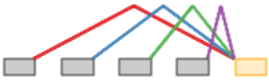	29 0 30
147	PROSER2	ENSG00000148426:11911365-11914276:target	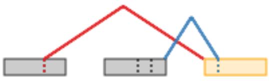	25 0 25
148	TCF7L2	ENSG00000148737:114799784-114799885:target	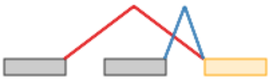	33 0 33
149	IMMP1L	ENSG00000148950:31455007-31455302:source	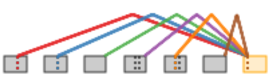	28 0
150	ARFGAP2	ENSG00000149182:47193832-47193884:source	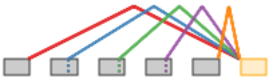	41 0 42
151	ARFGAP2	ENSG00000149182:47194649-47195033:target	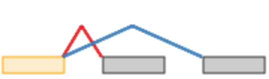	51 0 51
152	ALDOA	ENSG00000149925:30078555-30078687:target	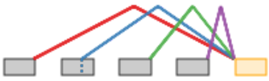	27 0 26
153	HMGA2	ENSG00000149948:66232299-66232349:source	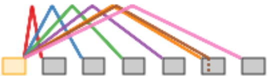	22 0 14
154	MKX	ENSG00000150051:28023385-28023720:target	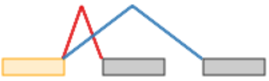	21 0 21
155	FAM177A1	ENSG00000151327:35515659-35515834:target	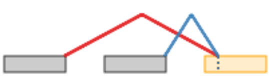	39 0 39
156	DST	ENSG00000151914:56324929-56326866:source	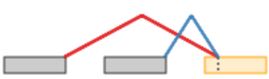	37 0 37
157	DST	ENSG00000151914:56328363-56329114:target	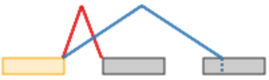	29 0 29
158	XRCC4	ENSG00000152422:82400727-82400877:target	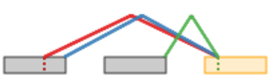	41 0 41
159	SLC25A28	ENSG00000155287:101373226-101373681:target	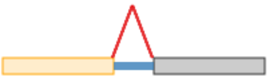	28 0 28
#	Gene	LSV ID	LSV Type	← More in DKO | More in WT →

#	Gene	LSV ID	LSV Type	← More in DKO | More in WT →
160	LARP1	ENSG00000155506:154173606-154173755:target		5 0 20
161	FAM122B	ENSG00000156504:133915851-133915947:target		20 0 20
162	EPB41	ENSG00000159023:29391469-29391733:target		52 0 16 37
163	ANO10	ENSG00000160746:43407840-43408466:source		26 0 26
164	RUSC1	ENSG00000160753:155294636-155294734:target		9 0 29
165	CLPB	ENSG00000162129:72013183-72013417:source		24 0 24
166	CLPB	ENSG00000162129:72013183-72013417:target		35 0 35
167	NOL9	ENSG00000162408:6609316-6609758:target		23 0 23
168	KIAA1522	ENSG00000162522:33233388-33233558:target		27 0 27
169	C1orf52	ENSG00000162642:85725041-85725355:target		25 0 25
170	RPP14	ENSG00000163684:58295781-58296133:target		12 0 25
171	RNF123	ENSG00000164068:49758453-49758538:target		22 0 22
172	CCDC112	ENSG00000164221:114604579-114604697:source		21 0 21
173	DNAAF5	ENSG00000164818:810108-812612:target		21 0 21
174	FAM69B	ENSG00000165716:139612029-139612646:source		21 0 21
175	CASC4	ENSG00000166734:44705534-44705741:target		20 0 20
176	KIAA0101	ENSG00000166803:64657193-64658274:source		33 0 33
177	KIAA0101	ENSG00000166803:64673157-64673354:target		21 0 21
178	VPS39	ENSG00000166887:42483459-42483758:source		25 0 25
179	VPS39	ENSG00000166887:42492094-42492159:target		31 0 31

Gene LSV ID LSV Type ← More in DKO | More in WT →

#	Gene	LSV ID	LSV Type	← More in DKO | More in WT →
180	SRR	ENSG00000167720:2226430-2226639:target	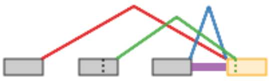	43 0 43
181	GNG4	ENSG00000168243:235747040-235747148:source	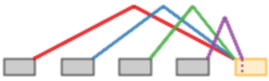	19 0 21
182	ATXN2L	ENSG00000168488:28836687-28836723:target	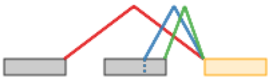	22 0 14
183	TMUB2	ENSG00000168591:42264985-42265111:source	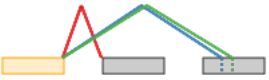	17 0 20
184	RGS14	ENSG00000169220:176798874-176799602:target	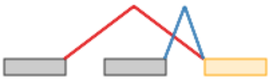	20 0 20
185	FABP6	ENSG00000170231:159640734-159640826:source	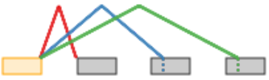	31 0 21
186	KRT8	ENSG00000170421:53292453-53292683:target	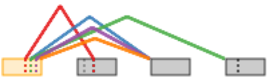	29 0 29
187	CHCHD7	ENSG00000170791:57127157-57128137:target	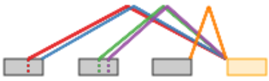	23 0 13
188	MALT1	ENSG00000172175:56377208-56377304:source	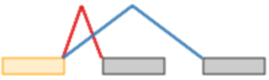	40 0 40
189	MALT1	ENSG00000172175:56381315-56381341:target	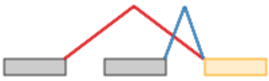	40 0 40
190	SNAPC5	ENSG00000174446:66789828-66790151:target	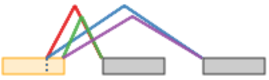	23 0 15
191	FOSL1	ENSG00000175592:65664280-65664477:target	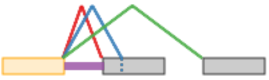	22 0 14
192	FBXO46	ENSG00000177051:46213887-46216831:source	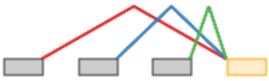	21 0 21
193	PUS1	ENSG00000177192:132416060-132417225:source	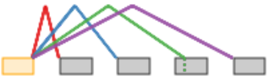	25 0 27
194	CPNE7	ENSG00000178773:89662892-89663654:target	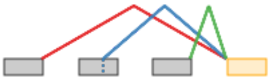	15 0 21
195	PER1	ENSG00000179094:8048069-8048488:source	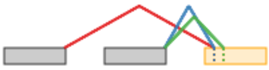	24 0 24
196	TNRC18	ENSG00000182095:5434071-5434226:source	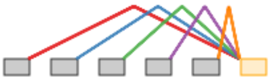	28 0 17
197	FBXL6	ENSG00000182325:145581288-145581633:target	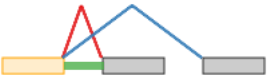	7 19 0 26
198	ANXA2	ENSG00000182718:60689454-60689537:target	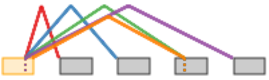	24 0 24
199	MTA1	ENSG00000182979:105931671-105932915:source	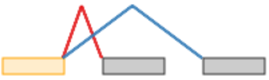	23 0 23

| # | Gene | LSV ID | LSV Type | ← More in DKO \| More in WT → |

#	Gene	LSV ID	LSV Type	← More in DKO | More in WT →
200	MTA1	ENSG00000182979:105935804-105935835:target	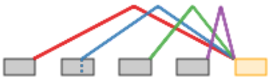	24 0 15
201	NAA38	ENSG00000183011:7761567-7761814:target	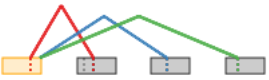	49 0 49
202	UPP1	ENSG00000183696:48134360-48134424:source	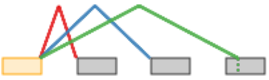	17 16 0 34
203	UPP1	ENSG00000183696:48141421-48141579:target	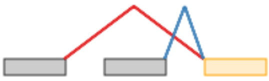	20 0 20
204	TBL3	ENSG00000183751:2028553-2032934:target	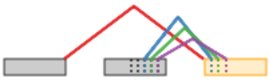	50 0 14 12 20
205	SMTN	ENSG00000183963:31484088-31484239:source	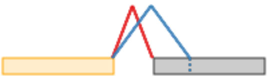	20 0 20
206	TOP1MT	ENSG00000184428:144413394-144413509:source	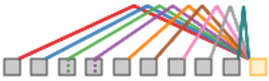	1051 0 72
207	TXNRD2	ENSG00000184470:19868145-19868240:source	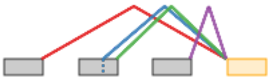	24 0 30
208	JAG2	ENSG00000184916:105617202-105617248:source	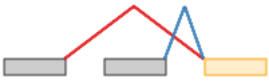	20 0 20
209	JAG2	ENSG00000184916:105617620-105617733:target	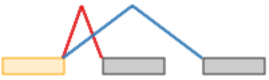	23 0 23
210	PARPBP	ENSG00000185480:102517664-102517819:source	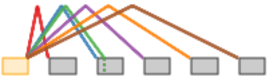	22 0 10
211	PCYT2	ENSG00000185813:79858841-79862820:source	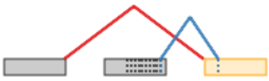	33 0 33
212	GPAT2	ENSG00000186281:96688706-96688771:source	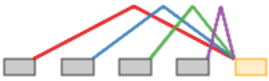	21 0 22
213	GPAT2	ENSG00000186281:96689671-96689748:target	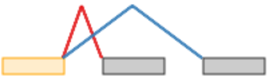	22 0 22
214	GPAT2	ENSG00000186281:96691931-96692053:source	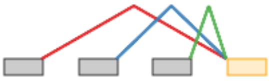	12 0 25
215	GPAT2	ENSG00000186281:96693739-96693838:target	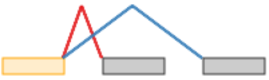	34 0 34
216	NF2	ENSG00000186575:30077428-30077590:source	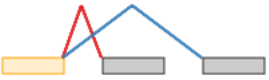	29 0 29
217	NF2	ENSG00000186575:30090741-30094587:target	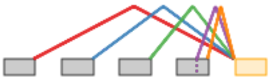	23 0 22
218	ESPN	ENSG00000187017:6508701-6508862:target	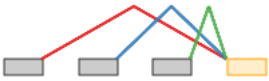	23 0 23
219	RP11-43F13.1	ENSG00000188002:1632960-1633021:target	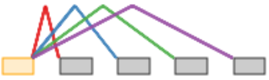	23 0

| # | Gene | LSV ID | LSV Type | ← More in DKO \| More in WT → |

#	Gene	LSV ID	LSV Type	← More in DKO | More in WT →
220	HES4	ENSG00000188290:935072-935167:target	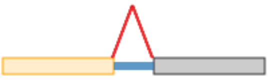	23 0 23
221	XPNPEP3	ENSG00000196236:41322273-41328819:target	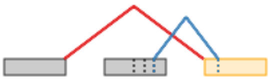	33 0 33
222	PRPF40A	ENSG00000196504:153533965-153533989:source	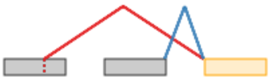	20 0 20
223	CD47	ENSG00000196776:107762145-107766139:source	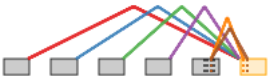	5 0 23
224	WDR45	ENSG00000196998:48935700-48935771:source	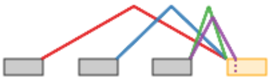	21 0 21
225	ANXA6	ENSG00000197043:150496688-150496741:target	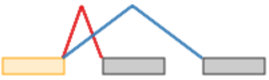	21 0 21
226	ABCB8	ENSG00000197150:150731360-150731515:target	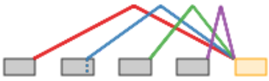	24 0 19
227	MIB2	ENSG00000197530:1564414-1564691:target	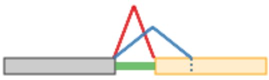	36 5 0 39
228	SPTAN1	ENSG00000197694:131353756-131353904:source	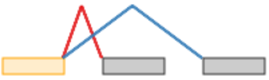	42 0 42
229	SPTAN1	ENSG00000197694:131356454-131356652:target	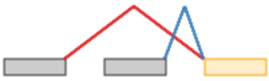	35 0 35
230	PSAP	ENSG00000197746:73585594-73585650:target	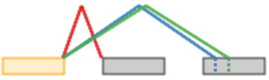	21 0 21
231	TPM2	ENSG00000198467:35684485-35684547:source	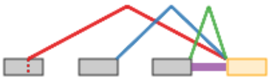	32 0 31
232	TPM2	ENSG00000198467:35685142-35685336:target	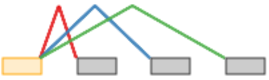	31 0 30
233	CTNND1	ENSG00000198561:57529234-57529591:source	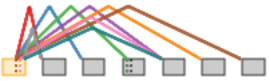	19 0 21
234	PFDN6	ENSG00000204220:33257547-33257697:target	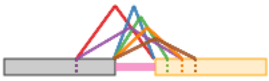	9 8 0 15
235	C12orf73	ENSG00000204954:104350082-104350526:target	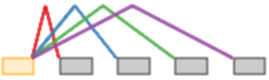	27 0
236	IPO7	ENSG00000205339:9459301-9459592:source	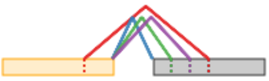	22 0 22 5
237	ITSN1	ENSG00000205726:35186217-35186811:source	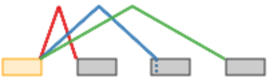	29 0 29
238	HLA-H	ENSG00000206341:29855732-29856170:source	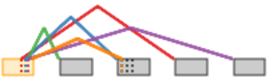	23 0
239	HLA-H	ENSG00000206341:29894501-29895176:target	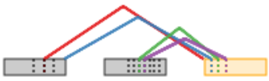	23 0 18 5

| # | Gene | LSV ID | LSV Type | ← More in DKO \| More in WT → |

#	Gene	LSV ID	LSV Type	← More in DKO | More in WT →
240	HLA-H	ENSG00000206341:29911899-29912393:target	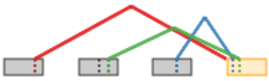	21 0 22
241	AP1G2	ENSG00000213983:24035487-24035628:source	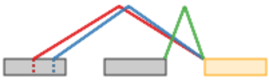	21 0 21
242	TRIM16	ENSG00000221926:15546035-15546278:source	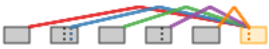	24 0 14
243	AFG3L1P	ENSG00000223959:90038994-90039173:source	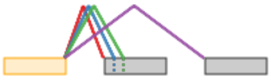	16 0 24
244	PROSER2-AS1	ENSG00000225778:11891612-11894214:source	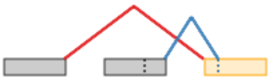	45 0 45
245	PROSER2-AS1	ENSG00000225778:11911365-11911500:target	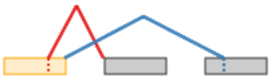	25 0 25
246	FAHD2CP	ENSG00000231584:96688416-96688501:target	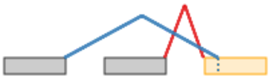	25 0 25
247	RP4-694A7.2	ENSG00000233589:68944812-68945003:source	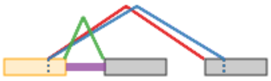	20 0 15 5
248	DECR2	ENSG00000242612:460572-460784:target	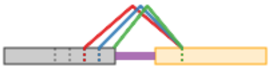	25 6 0 32
249	AC004967.7	ENSG00000243554:97599082-97599157:source	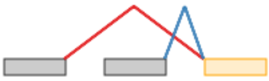	34 0 34
250	N4BP2L2	ENSG00000244754:33101012-33101669:source	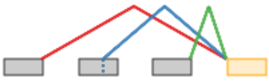	20 0 16
251	AP000304.12	ENSG00000249209:35186217-35186376:source	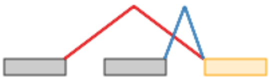	30 0 30
252	ZFPM2-AS1	ENSG00000251003:106799677-106799824:target	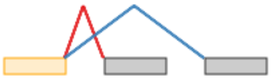	29 0 29
253	STX16-NPEPL1	ENSG00000254995:57245568-57245659:target	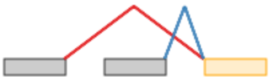	20 0 20
254	RP11-386G11.10	ENSG00000258017:49579302-49579704:target	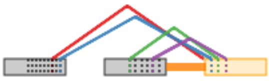	64 7 5 0 75
255	RP11-430B1.2	ENSG00000259577:52497062-52498071:target	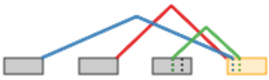	28 0 27
256	RP11-304L19.1	ENSG00000259933:2141437-2141615:source	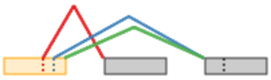	0 20
257	RP11-315D16.2	ENSG00000260007:68521840-68522056:target	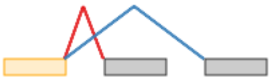	20 0 20
258	HERC2P5	ENSG00000260644:32779596-32779783:source	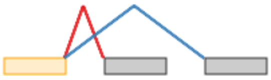	75 0 75
259	VPS9D1-AS1	ENSG00000261373:89782870-89783312:target	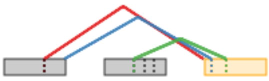	13 0 21

Gene LSV ID LSV Type ← More in DKO | More in WT →

#	Gene	LSV ID	LSV Type	← More in DKO | More in WT →
260	MIR1539	ENSG00000265496:47017996-47018248:target	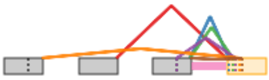	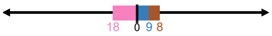
261	AARSD1	ENSG00000266967:41106866-41107051:target	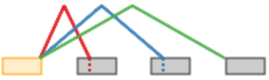	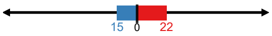
262	RP11-589P10.7	ENSG00000267047:6916985-6917061:target	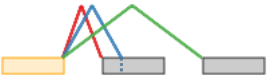	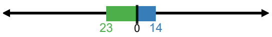
263	RP11-126O1.4	ENSG00000267476:56377208-56377304:source	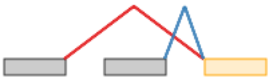	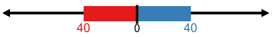
264	RP11-126O1.4	ENSG00000267476:56381315-56381341:target	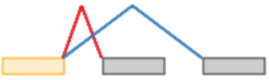	
#	Gene	LSV ID	LSV Type	← More in DKO | More in WT →

10 ▼

© BIOCIPHERS · Department of Genetics Perelman School of Medic
Department of Computer and Information Science School of Enginee
University of Pennsylvania Penn

LSV filters

- ☑ 5-prime
- ☑ 3-prime
- ☑ Exon skipping
- ☑ Single Source
- ☑ Single Target

Number of junctions:
from:
to:

Number of exons:
from:
to:
