# Supplementary material for: CD44 alternative splicing senses intragenic DNA methylation in tumors via direct and indirect mechanisms
Source: Nucleic Acids Res. 2021 Jun 4;49(11):6213–37. doi: 10.1093/nar/gkab437 (PMC8216461; doi:10.1093/nar/gkab437)
Supplement: gkab437_Supplemental_Files [file gkab437_supplemental_files.zip › Batsche_Sup Table S2B.pdf]

Batsché *et al.*, Supplementary Table S2B

Differential expression and splicing of RNA-binding protein/splicing factors in DKO cells versus WT HCT116 cells. This Table is a hub of Supplementary Table S1 and Supplementary Table S2A for splicing factors.

Regulated RNA binding factors

Ensembl ID	Gene	Parent (eudog	RNA level Log2 cpm	Differential RNA expression Regulation	Log2 FC	pval	Differentially spliced curation	LSV ID
ENSG00000109536	FRG1	p	4.8				CE8	190878553-190878657:S
ENSG00000107625	DDX50	p	5.6				CE3	70666467-70666763:T
ENSG00000126653	NSRP1	p	6.2				CE9…	28499560-28499616:T<br>:28443664-28443881:S
ENSG00000116001	TIA1		6.2				5'SS E10/Ee11	70443536-70443631:S
ENSG00000013441	CLK1		6.4				FE1 or FE6	201724403-201724469:S
ENSG00000198563	DDX39B		6.6				3'SS E6	31508099-31508441:S<br>31509727-31510225:T
ENSG00000117360	PRPF3	p	6.6				CE4	150300234-150300925:T
ENSG00000100296	THOC5		6.7				CE2+3 & CE15	29924926-29925228:S<br>29949660-29950243:T
ENSG00000164548	TRA2A	p	7.4				CE4	:23571408-23571660:T
ENSG00000134186	PRPF38B		7.5				CE7	109240322-109241449:T<br>109238899-109238959:S
ENSG00000179950	PUF60		7.7				3'SS CE6	144911450-144912029:T
ENSG00000197111	PCBP2	p	7.7				5'SS FE	53835525-53835584:S
ENSG00000153914	SREK1		7.7				CE6 or TE	65449396-65449618:S
ENSG00000151923	TIAL1		7.9				3'SS e7	121347664-121347760:T<br>121336288-121336417:T
ENSG00000029363	BCLAF1	p	8.1				CE13+14	136590575-136591097:T
ENSG00000196504	PRPF40A		8.2				CE11	153533965-153533989:S<br>153535643-153535986:T
ENSG00000154473	BUB3	p	8.3				TE8 or TE9	124922128-124922757:S
ENSG00000145833	DDX46		8.3				5'SS e22	134152120-134152296:S
ENSG00000136527	TRA2B		8.4				FE1 or FE2	:185644389-185646861:S<br>:185655613-185655924:T
ENSG00000124193	SRSF6	p	8.6				CE7 or TE	42089343-42092245:T
ENSG00000135829	DHX9	p	8.6				CE4	182821368-182821479:T<br>182811680-182811812:S
ENSG00000160710	ADAR		8.9				CE4	154574861-154575102:S
ENSG00000168566	SNRNP48		5.6	down	-0.7	0.035	CE5a	7599906-7601757:T
ENSG00000060138	YBX3	p	7.7	down	-0.9	0.006	CE12+13	10856622-10857037:S
ENSG00000092847	AGO1		6.9	up	0.7	0.016	FE1 or FE2	36354028-36354211:T
ENSG00000100320	RBFOX2		6.8	down	-1.3	0.000		
ENSG00000152601	MBNL1		7.0	down	-1.3	0.018		
ENSG00000136231	IGF2BP3	p	5.8	down	-1.2	0.011		
ENSG00000099622	CIRBP		7.8	down	-1.1	0.004		
ENSG00000104413	ESRP1		6.6	down	-1.0	0.013		
ENSG00000065978	YBX1	p	7.5	down	-0.9	0.010		
ENSG00000148690	FRA10AC1		5.3	down	-0.9	0.002		
ENSG00000137944	CCBL2		4.5	down	-0.8	0.001		
ENSG00000056097	ZFR	p	7.1	down	-0.8	0.010		
ENSG00000117614	SYF2	p	6.0	down	-0.6	0.006		
ENSG00000100056	DGCR14		4.4	up	0.6	0.038		
ENSG00000123136	DDX39A	p	7.2	up	0.6	0.038		
ENSG00000169217	CD2BP2	p	6.1	up	0.7	0.011		
ENSG00000131043	AAR2		5.1	up	0.7	0.037		
ENSG00000071859	FAM50A		6.2	up	0.7	0.043		
ENSG00000126803	HSPA2		2.6	up	1.2	0.003		
ENSG00000204389	HSPA1A		2.4	up	3.8	0.010		
ENSG00000154548	SRSF12		-0.7	up	4.1	0.002		
ENSG00000185272	RBM11		-1.2	up	5.4	0.002		
ENSG00000128739	SNRPN	p	-1.6	up	5.4	0.001		

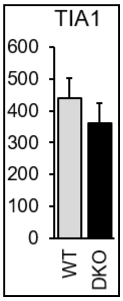

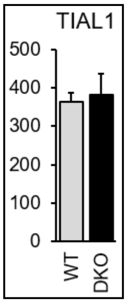

(MAJIQ) LSV Type — Regulation dPSI>20% [95%] <-More in DKO // More in WT->

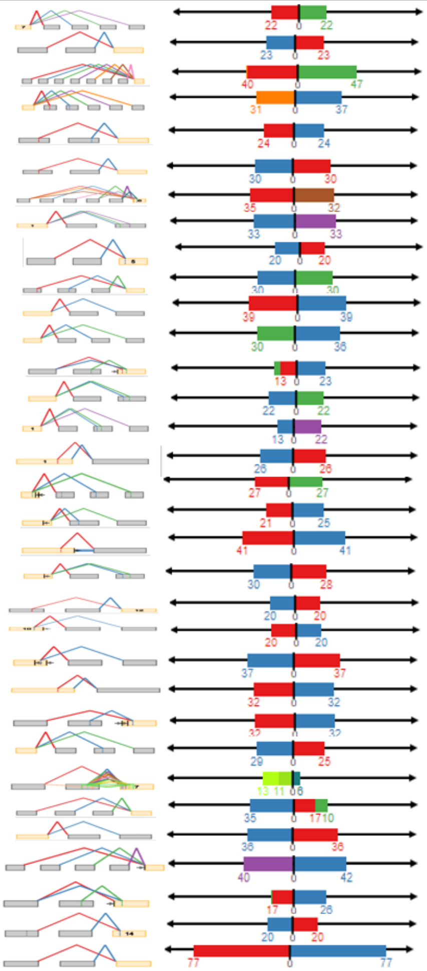

In the Manual curation column : CE=cassette exon, FE=First exon (promoter), TE=terminal exon (3'end), SS= splice site

In the LSV ID column: T=target, S=source

P indicated that these genes have been described as parent of pseudogene (Pei et al., 2012 in Genome Biol. 13,9,R51, The GENCODE pseudogene resource), which means that the splicing analysis can be misled by the expression of the retropseudogene or pseudogene.

Inset shows RT-qPCR validation of RNA-Seq analysis
