# Supplementary material for: CD44 alternative splicing senses intragenic DNA methylation in tumors via direct and indirect mechanisms
Source: Nucleic Acids Res. 2021 Jun 4;49(11):6213–37. doi: 10.1093/nar/gkab437 (PMC8216461; doi:10.1093/nar/gkab437)
Supplement: gkab437_Supplemental_Files [file gkab437_supplemental_files.zip › Batsche_Sup Table S4_ΔΨ ALLvsHCB_May2019.pdf]

BioCiphers

LSV ΔΨ Index: Healthy Vs ALL

#	Gene	LSV ID	LSV Type	← More in Healthy | More in ALL →
0	MAD1L1	ENSG00000002822:1976323-1976533:source	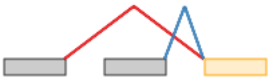	75 0 75
1	CFLAR	ENSG00000003402:201994366-201994869:target	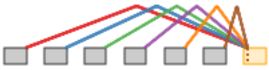	28 0 28
2	RBM5	ENSG00000003756:50137415-50137605:source	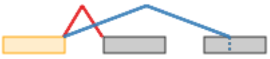	20 0 20
3	RBM5	ENSG00000003756:50139770-50140599:source	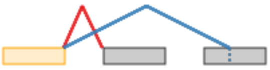	32 0 32
4	RBM5	ENSG00000003756:50139770-50140599:target	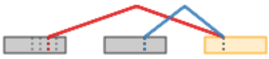	34 0 34
5	RBM5	ENSG00000003756:50142010-50142575:target	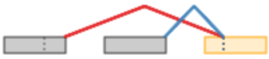	34 0 34
6	RBM5	ENSG00000003756:50147036-50147256:source	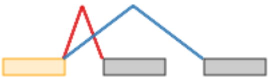	26 0 26
7	RBM5	ENSG00000003756:50148112-50148325:target	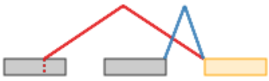	26 0 26
8	RBM6	ENSG00000004534:50085678-50085752:target	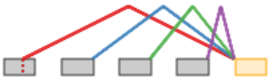	31 0 8 22
9	RBM6	ENSG00000004534:50095835-50096348:source	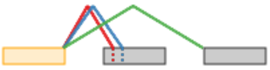	38 0 38
10	RBM6	ENSG00000004534:50098389-50098431:target	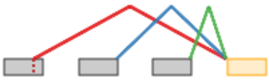	29 0 30
11	RBM6	ENSG00000004534:50098895-50099181:source	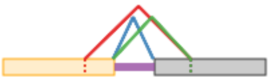	12 13 0 31
12	RBM6	ENSG00000004534:50099316-50099541:target	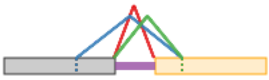	12 13 0 31
13	CDC27	ENSG00000004897:45219595-45219747:target	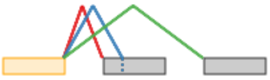	20 0 15
14	CDC27	ENSG00000004897:45234596-45234750:source	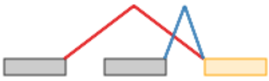	23 0 23
15	CDC27	ENSG00000004897:45247283-45247408:source	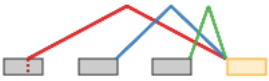	39 0 37
16	CDC27	ENSG00000004897:45247283-45247408:target	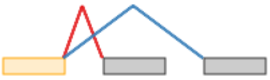	26 0 26
17	CDC27	ENSG00000004897:45258648-45259003:target	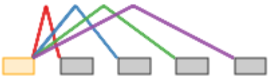	41 0 43
18	UPF1	ENSG00000005007:18963796-18963913:source	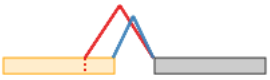	28 0 28

| # | Gene | LSV ID | LSV Type | ← More in Healthy \| More in ALL → |

#	Gene	LSV ID	LSV Type	← More in Healthy | More in ALL →
19	UPF1	ENSG00000005007:18973742-18974421:source	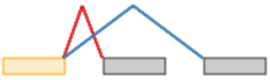	30 0 30
20	UPF1	ENSG00000005007:18976098-18976259:target	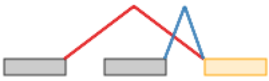	29 0 29
21	CREBBP	ENSG00000005339:3786509-3787000:source	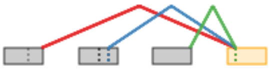	44 0 39
22	CREBBP	ENSG00000005339:3790400-3793469:target	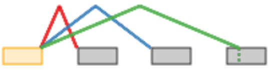	38 0 40
23	CREBBP	ENSG00000005339:3795278-3795655:source	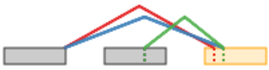	24 19 0 43
24	CREBBP	ENSG00000005339:3832685-3832927:source	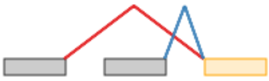	21 0 21
25	CREBBP	ENSG00000005339:3843387-3843627:target	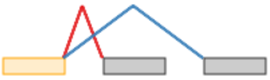	31 0 31
26	MYCBP2	ENSG00000005810:77644766-77644846:source	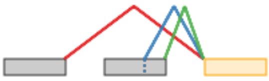	12 9 0 23
27	MYCBP2	ENSG00000005810:77661619-77661768:source	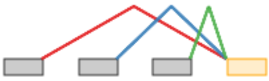	42 21 0 61
28	MYCBP2	ENSG00000005810:77664241-77664398:target	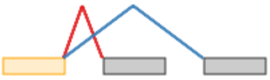	50 0 50
29	MYCBP2	ENSG00000005810:77695360-77695630:target	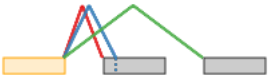	25 0 18
30	MYCBP2	ENSG00000005810:77699471-77699603:source	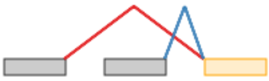	22 0 22
31	MYCBP2	ENSG00000005810:77713331-77713471:target	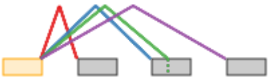	29 0 27
32	MYCBP2	ENSG00000005810:77817194-77817295:target	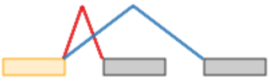	32 0 32
33	ENSG00000005955	ENSG00000005955:34933087-34934622:source	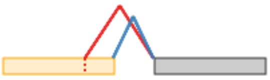	22 0 22
34	ENSG00000006114	ENSG00000006114:35880641-35880751:target	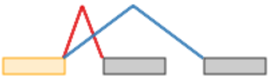	24 0 24
35	ENSG00000006114	ENSG00000006114:35913217-35914161:target	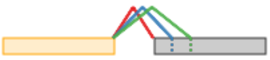	31 0 6 24
36	ENSG00000006114	ENSG00000006114:35944430-35944867:target	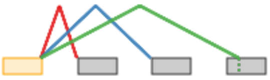	29 0 22
37	RALA	ENSG00000006451:39736284-39736458:source	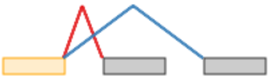	72 0 72
38	RALA	ENSG00000006451:39745722-39747723:target	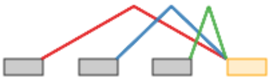	62 0 62
39	DBF4	ENSG00000006634:87507368-87507872:source	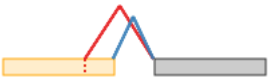	20 0 20

| # | Gene | LSV ID | LSV Type | ← More in Healthy \| More in ALL → |

#	Gene	LSV ID	LSV Type	← More in Healthy | More in ALL →
40	DBF4	ENSG00000006634:87530079-87530193:source	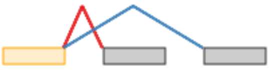	22 0 22
41	PAF1	ENSG00000006712:39877334-39877439:source	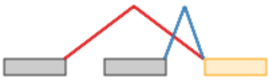	23 0 23
42	PAFAH1B1	ENSG00000007168:2573457-2573668:source	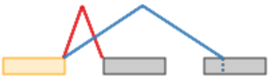	21 0 21
43	PAFAH1B1	ENSG00000007168:2576959-2577582:target	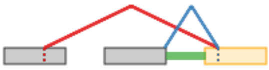	39 0 37
44	KIAA0100	ENSG00000007202:26943636-26943771:source	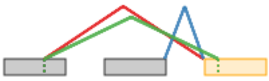	6 14 0 20
45	KIAA0100	ENSG00000007202:26944246-26944336:target	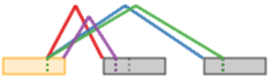	11 17 0 31
46	KIAA0100	ENSG00000007202:26951251-26951408:source	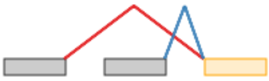	20 0 20
47	KIAA0100	ENSG00000007202:26958497-26958699:target	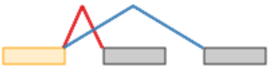	28 0 28
48	CD79B	ENSG00000007312:62007130-62007248:source	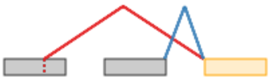	24 0 24
49	LUC7L	ENSG00000007392:258077-258507:source	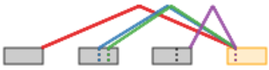	25 0 26
50	LUC7L	ENSG00000007392:277241-277685:source	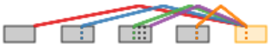	30 0 6 20
51	MYLIP	ENSG00000007944:16129356-16129640:source	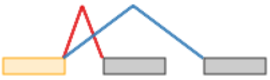	27 0 27
52	MYLIP	ENSG00000007944:16130788-16130978:source	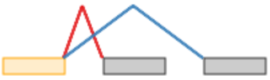	30 0 30
53	MYLIP	ENSG00000007944:16141856-16142041:target	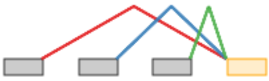	6 16 0 25
54	MYLIP	ENSG00000007944:16143251-16143448:target	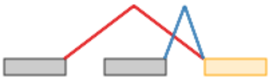	23 0 23
55	CDK11A	ENSG00000008128:1635263-1635434:source	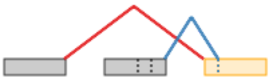	29 0 29
56	CDK11A	ENSG00000008128:1635662-1635783:target	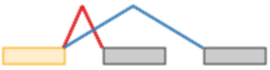	21 0 21
57	CDK11A	ENSG00000008128:1653035-1653318:source	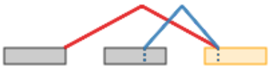	28 0 28
58	SPAG9	ENSG00000008294:49118862-49119012:target	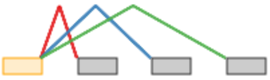	12 0 21
59	TRAF3IP3	ENSG00000009790:209946293-209946364:target	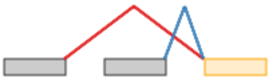	27 0 27

Gene LSV ID LSV Type ← More in Healthy | More in ALL →

#	Gene	LSV ID	LSV Type	← More in Healthy | More in ALL →
60	BAZ1B	ENSG00000009954:72876794-72877429:source		37 0 37
61	BAZ1B	ENSG00000009954:72879769-72879895:target		31 0 31
62	RANBP9	ENSG00000010017:13697017-13697128:target		23 0 23
63	CLCN6	ENSG00000011021:11879545-11879611:source		6 32 0 42
64	CLCN6	ENSG00000011021:11883764-11883890:target		41 0 41
65	CLCN6	ENSG00000011021:11889253-11889774:source		6 6 37 0 60
66	CLCN6	ENSG00000011021:11900200-11903201:target		15 6 0 22
67	AKAP8L	ENSG00000011243:15508525-15509214:source		21 0 21
68	MBTD1	ENSG00000011258:49278953-49279057:source		69 0 69
69	MBTD1	ENSG00000011258:49280062-49280296:target		65 0 65
70	RNF216	ENSG00000011275:5780604-5781446:target		43 0 47
71	RNF216	ENSG00000011275:5792477-5792610:target		31 0 31
72	RNF216	ENSG00000011275:5800634-5800769:source		21 0 21
73	PIK3C2A	ENSG00000011405:17121407-17121516:source		22 0 22
74	CD22	ENSG00000012124:35828658-35828924:source		26 0 26
75	CD22	ENSG00000012124:35832004-35832041:source		24 0 23
76	ELOVL5	ENSG00000012660:53156614-53156761:source		41 0 41
77	ELOVL5	ENSG00000012660:53160440-53160505:target		49 0 49
78	ATP2C1	ENSG00000017260:130711775-130711845:target		13 8 0 24
79	RUFY3	ENSG00000018189:71668588-71668893:source		24 0 8 11

Gene LSV ID LSV Type ← More in Healthy | More in ALL →

#	Gene	LSV ID	LSV Type	← More in Healthy | More in ALL →
80	ZRANB1	ENSG00000019995:126671744-126671873:source	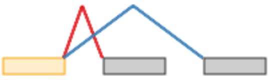	38 0 38
81	RNF10	ENSG00000022840:121004756-121004783:source	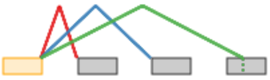	21 0 20
82	RNF10	ENSG00000022840:121012940-121013491:target	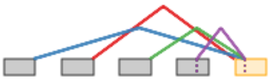	20 0 19
83	RB1CC1	ENSG00000023287:53537277-53537347:target	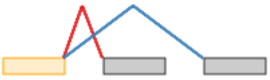	25 0 25
84	STRAP	ENSG00000023734:16035325-16035753:source	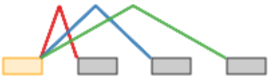	51 0 51
85	STRAP	ENSG00000023734:16042862-16042943:target	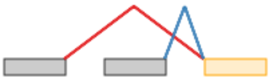	53 0 53
86	STRAP	ENSG00000023734:16055851-16056412:target	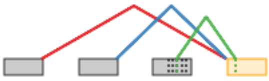	20 0 20
87	UBR2	ENSG00000024048:42600543-42600642:source	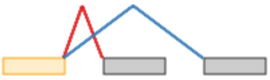	26 0 26
88	UBR2	ENSG00000024048:42630996-42631157:source	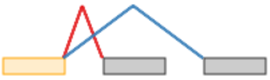	24 0 24
89	UBR2	ENSG00000024048:42633905-42633983:target	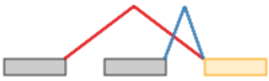	35 0 35
90	UBR2	ENSG00000024048:42652532-42652609:source	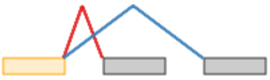	38 0 38
91	UBR2	ENSG00000024048:42657307-42657408:target	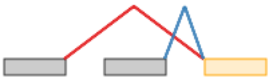	26 0 26
92	HSF2	ENSG00000025156:122744726-122744831:source	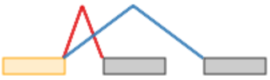	31 0 31
93	HSF2	ENSG00000025156:122752575-122752659:target	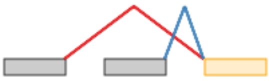	32 0 32
94	PHF20	ENSG00000025293:34459572-34459815:source	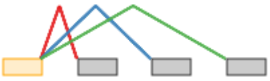	19 0 20
95	TOMM34	ENSG00000025772:43572094-43572220:source	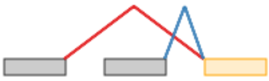	42 0 42
96	TOMM34	ENSG00000025772:43580474-43580643:target	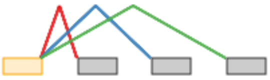	38 16 0 57
97	KPNA6	ENSG00000025800:32620189-32620322:target	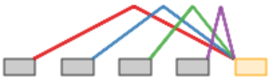	50 0 49
98	KPNA6	ENSG00000025800:32622454-32622639:source	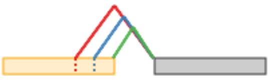	24 0 24
99	CD44	ENSG00000026508:35240476-35240934:source	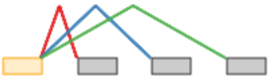	36 0 38

| # | Gene | LSV ID | LSV Type | ← More in Healthy \| More in ALL → |

#	Gene	LSV ID	LSV Type	← More in Healthy | More in ALL →
100	CD44	ENSG00000026508:35250676-35253949:target	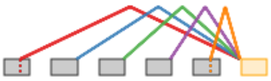	20 0 26
101	IFNGR1	ENSG00000027697:137524636-137524867:target	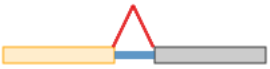	58 0 58
102	VEZT	ENSG00000028203:95645716-95646216:source	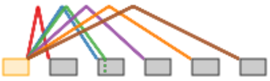	25 0 23
103	SNX1	ENSG00000028528:64404772-64404883:source	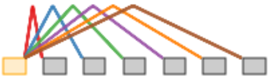	5 10 0 21
104	SNX1	ENSG00000028528:64429060-64429131:source	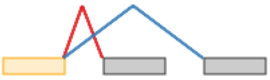	36 0 36
105	RABEP1	ENSG00000029725:5250085-5250220:source	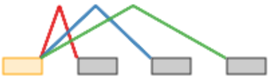	17 0 25
106	RABEP1	ENSG00000029725:5257654-5257785:source	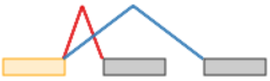	21 0 21
107	RABEP1	ENSG00000029725:5264503-5264970:source	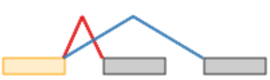	21 0 21
108	FAM13B	ENSG00000031003:137353991-137354203:source	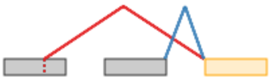	37 0 37
109	FAM13B	ENSG00000031003:137356452-137356886:target	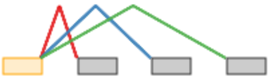	53 0 16 35
110	ARID4A	ENSG00000032219:58771662-58771727:source	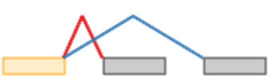	36 0 36
111	ARID4A	ENSG00000032219:58785260-58785339:target	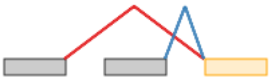	29 0 29
112	UBA6	ENSG00000033178:68496172-68496253:source	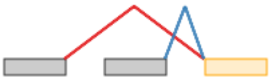	28 0 28
113	UBA6	ENSG00000033178:68496172-68496253:target	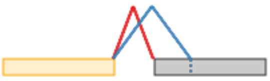	28 0 28
114	UBA6	ENSG00000033178:68497426-68497489:target	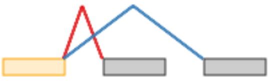	29 0 29
115	PIAS1	ENSG00000033800:68378644-68379088:source	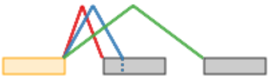	44 0 48
116	PIAS1	ENSG00000033800:68434284-68434368:target	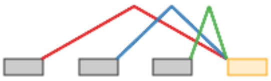	7 32 0 41
117	RNF19A	ENSG00000034677:101273770-101273983:source	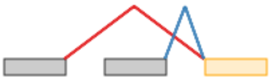	45 0 45
118	RNF19A	ENSG00000034677:101276899-101277013:target	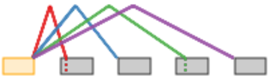	35 0 40
119	STAP1	ENSG00000035720:68449292-68449420:source	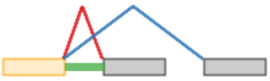	22 0 22

| # | Gene | LSV ID | LSV Type | ← More in Healthy \| More in ALL → |

#	Gene	LSV ID	LSV Type	← More in Healthy | More in ALL →
120	STAP1	ENSG00000035720:68456602-68456671:target		31 0 31
121	RFC1	ENSG00000035928:39304680-39304813:target		22 0 22
122	TBC1D23	ENSG00000036054:100034707-100035031:target		35 0 35
123	CUL3	ENSG00000036257:225370466-225370849:source		6 10 0 20
124	CUL3	ENSG00000036257:225400245-225400358:target		15 12 0 27
125	NSUN2	ENSG00000037474:6609939-6610035:target		24 0 24
126	NSUN2	ENSG00000037474:6620219-6621602:source		22 0 21
127	NSUN2	ENSG00000037474:6623327-6623398:target		23 0 23
128	BOD1L1	ENSG00000038219:13570362-13571752:source		21 0 21
129	BOD1L1	ENSG00000038219:13579205-13579825:source		29 0 29
130	BOD1L1	ENSG00000038219:13581552-13581690:target		22 0 22
131	TRIO	ENSG00000038382:14471427-14471575:source		41 0 41
132	TRIO	ENSG00000038382:14474103-14474206:target		39 0 39
133	TRIO	ENSG00000038382:14496988-14497126:source		20 0 20
134	TRIO	ENSG00000038382:14504502-14504783:source		51 0 51
135	TRIO	ENSG00000038382:14507989-14510313:target		36 0 36
136	TDP1	ENSG00000042088:90429449-90430017:target		25 0 16
137	MED17	ENSG00000042429:93529576-93529706:source		45 0 45
138	MED17	ENSG00000042429:93533248-93535142:target		22 0 22
139	JADE2	ENSG00000043143:133887742-133887899:target		29 0 29

Gene LSV ID LSV Type ← More in Healthy | More in ALL →

#	Gene	LSV ID	LSV Type	← More in Healthy | More in ALL →
140	JADE2	ENSG00000043143:133909335-133911427:source	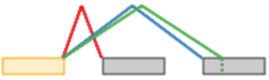	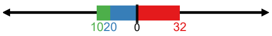 1020 0 32
141	JADE2	ENSG00000043143:133914184-133918918:target	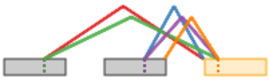	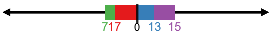 717 0 13 15
142	OFD1	ENSG00000046651:13785246-13785403:target	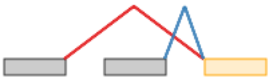	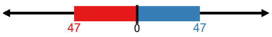 47 0 47
143	FAM214A	ENSG00000047346:52876940-52877520:source	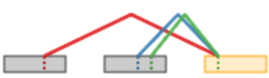	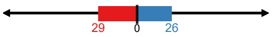 29 0 26
144	FAM214A	ENSG00000047346:52885614-52885933:target	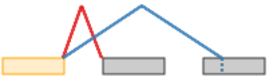	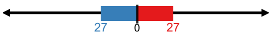 27 0 27
145	ARAP2	ENSG00000047365:36067620-36069900:source	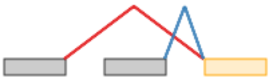	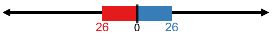 26 0 26
146	ARAP2	ENSG00000047365:36083873-36083908:target	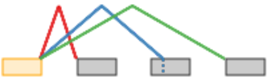	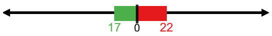 17 0 22
147	ARAP2	ENSG00000047365:36161734-36162263:target	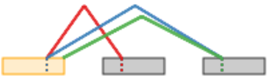	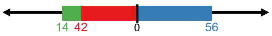 14 42 0 56
148	DTNBP1	ENSG00000047579:15523794-15524900:target	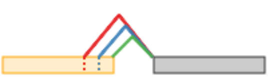	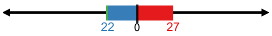 22 0 27
149	DTNBP1	ENSG00000047579:15652318-15652371:target	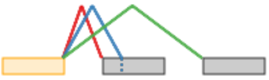	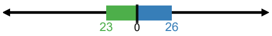 23 0 26
150	MAP4	ENSG00000047849:47892182-47894529:source	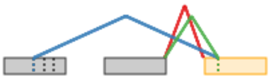	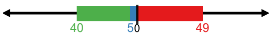 40 50 49
151	MAP4	ENSG00000047849:47896237-47896850:target	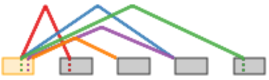	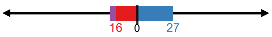 16 0 27
152	USP28	ENSG00000048028:113679020-113679159:target	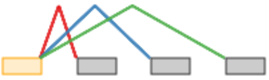	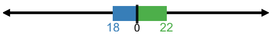 18 0 22
153	USP28	ENSG00000048028:113699919-113700189:source	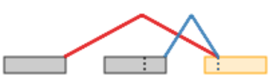	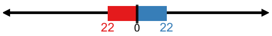 22 0 22
154	USP28	ENSG00000048028:113711320-113711479:target	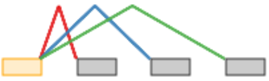	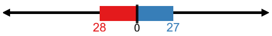 28 0 27
155	RSF1	ENSG00000048649:77409532-77409738:source	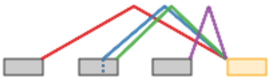	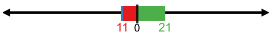 11 0 21
156	RSF1	ENSG00000048649:77436590-77436744:target	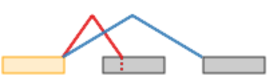	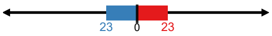 23 0 23
157	VPS13D	ENSG00000048707:12381866-12382021:target	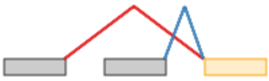	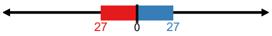 27 0 27
158	VPS13D	ENSG00000048707:12416455-12416581:source	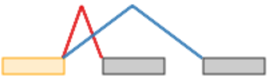	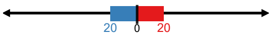 20 0 20
159	CELF2	ENSG00000048740:11291116-11291164:target	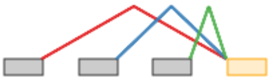	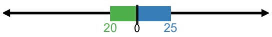 20 0 25

Gene LSV ID LSV Type ← More in Healthy | More in ALL →

#	Gene	LSV ID	LSV Type	← More in Healthy | More in ALL →
160	CELF2	ENSG00000048740:11299701-11299835:source	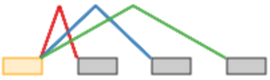	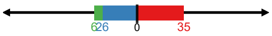
161	CELF2	ENSG00000048740:11312629-11312787:target	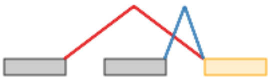	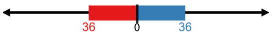
162	CELF2	ENSG00000048740:11356102-11356233:source	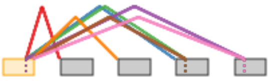	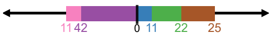
163	CELF2	ENSG00000048740:11363152-11363349:source	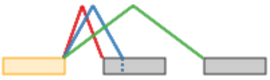	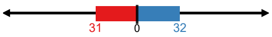
164	CELF2	ENSG00000048740:11363152-11363349:target	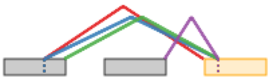	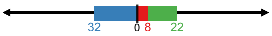
165	CELF2	ENSG00000048740:11367425-11367942:target	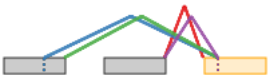	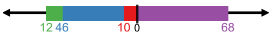
166	CELF2	ENSG00000048740:11372419-11374591:target	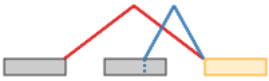	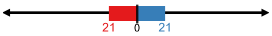
167	FAM120A	ENSG00000048828:96261072-96261168:source	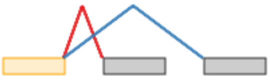	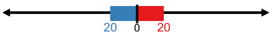
168	FAM120A	ENSG00000048828:96305504-96305753:source	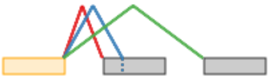	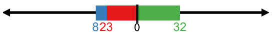
169	FAM120A	ENSG00000048828:96312859-96312973:source	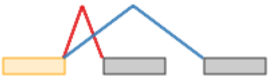	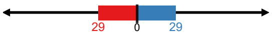
170	FAM120A	ENSG00000048828:96318664-96318873:target	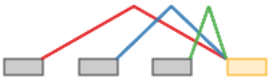	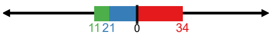
171	FAM120A	ENSG00000048828:96324490-96324586:source	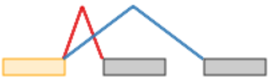	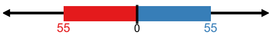
172	FAM120A	ENSG00000048828:96326511-96328397:target	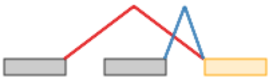	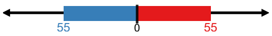
173	ARID1B	ENSG00000049618:157405796-157406039:target	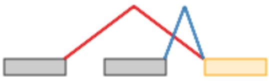	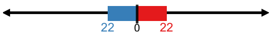
174	ARID1B	ENSG00000049618:157495142-157495251:source	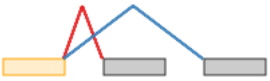	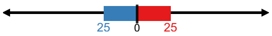
175	ARID1B	ENSG00000049618:157502103-157502332:target	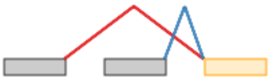	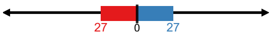
176	ARID1B	ENSG00000049618:157517299-157517449:source	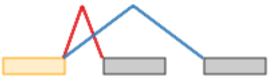	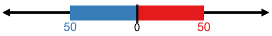
177	ARID1B	ENSG00000049618:157521839-157522622:target	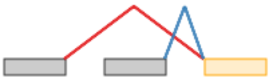	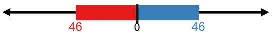
178	RRP12	ENSG00000052749:99126499-99126666:source	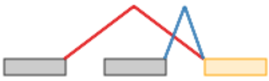	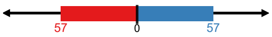
179	RRP12	ENSG00000052749:99129182-99129339:target	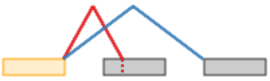	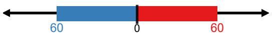

Gene LSV ID LSV Type ← More in Healthy | More in ALL →

#	Gene	LSV ID	LSV Type	← More in Healthy | More in ALL →
180	RRP12	ENSG00000052749:99131830-99131923:source		35 0 35
181	RRP12	ENSG00000052749:99132803-99132957:target		37 0 37
182	RRP12	ENSG00000052749:99144876-99145014:target		16 10 0 30
183	RRP12	ENSG00000052749:99153441-99153517:source		33 0 33
184	RRP12	ENSG00000052749:99160062-99160291:target		37 0 37
185	TTC17	ENSG00000052841:43436140-43436326:source		24 0 24
186	TTC17	ENSG00000052841:43464875-43465134:target		20 0 20
187	TTC17	ENSG00000052841:43465606-43466055:source		51 0 51
188	THRAP3	ENSG00000054118:36724983-36725085:target		30 18 0 42
189	ARID4B	ENSG00000054267:235323826-235323962:target		37 0 35
190	ARID4B	ENSG00000054267:235359346-235359430:source		31 0 27
191	ARID4B	ENSG00000054267:235383108-235383283:target		38 0 38
192	ARID4B	ENSG00000054267:235490106-235490283:source		21 0 21
193	OPN3	ENSG00000054277:241803184-241803701:target		47 0 21 27
194	SYNE2	ENSG00000054654:64689907-64690098:target		26 0 26
195	RELT	ENSG00000054967:73101800-73101966:source		61 0 61
196	RELT	ENSG00000054967:73103027-73103513:target		62 0 62
197	NOP58	ENSG00000055044:203157500-203157626:source		46 0 46
198	NOP58	ENSG00000055044:203162102-203162236:target		45 0 45
199	SZRD1	ENSG00000055070:16719723-16719977:target		16 0 21

Gene LSV ID LSV Type ← More in Healthy | More in ALL →

#	Gene	LSV ID	LSV Type	← More in Healthy | More in ALL →
200	CUL1	ENSG00000055130:148483643-148483691:source		22 0 22
201	CYFIP2	ENSG00000055163:156712346-156712488:target		54 0 57
202	CYFIP2	ENSG00000055163:156721792-156721868:target		7 6 0 21
203	CYFIP2	ENSG00000055163:156753179-156753275:source		32 0 32
204	CYFIP2	ENSG00000055163:156787290-156787380:source		18 27 0 48
205	CYFIP2	ENSG00000055163:156788413-156788606:target		53 0 53
206	USP36	ENSG00000055483:76808942-76809083:source		17 5 0 24
207	USP36	ENSG00000055483:76810494-76810634:target		28 0 32
208	USP36	ENSG00000055483:76832064-76832454:target		27 0 25
209	KMT2C	ENSG00000055609:151836143-151836344:target		21 0 21
210	KMT2C	ENSG00000055609:151932902-151933018:source		20 0 20
211	RC3H2	ENSG00000056586:125659558-125660389:source		15 0 23
212	ATP11B	ENSG00000058063:182547460-182547540:source		52 0 52
213	ATP11B	ENSG00000058063:182553823-182553941:target		28 0 28
214	ATP11B	ENSG00000058063:182598667-182599157:source		16 14 0 31
215	CDK14	ENSG00000058091:90546916-90547039:target		25 0 25
216	PPP1R12A	ENSG00000058272:80192274-80192364:source		23 0 5 17
217	PPP1R12A	ENSG00000058272:80200596-80201110:target		24 0 8 5
218	POLR3E	ENSG00000058600:22330147-22330267:source		38 0 38
219	POLR3E	ENSG00000058600:22335675-22335734:target		25 0 25

Gene LSV ID LSV Type ← More in Healthy | More in ALL →

#	Gene	LSV ID	LSV Type	← More in Healthy | More in ALL →
220	POLR3E	ENSG00000058600:22336740-22337599:source	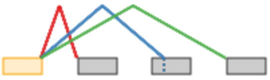	80 0 10 69
221	POLR3E	ENSG00000058600:22344965-22346424:target	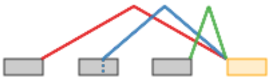	51 0 48
222	ZC3H11A	ENSG00000058673:203764782-203764922:source	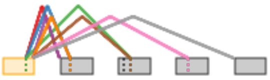	20 0 7 13
223	ZC3H11A	ENSG00000058673:203802919-203802981:source	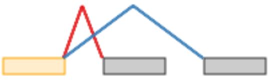	20 0 20
224	ZC3H11A	ENSG00000058673:203807094-203807192:target	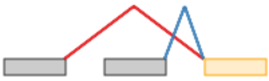	21 0 21
225	PARP12	ENSG00000059378:139737515-139738111:source	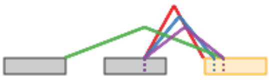	31 0 35
226	PARP12	ENSG00000059378:139741179-139741639:target	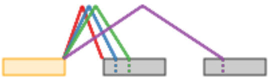	29 0 29
227	PARP12	ENSG00000059378:139757699-139757834:target	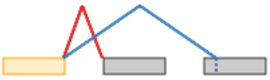	22 0 22
228	CDK17	ENSG00000059758:96717726-96717890:target	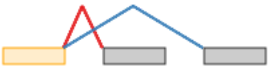	30 0 30
229	YBX3	ENSG00000060138:10862507-10862719:source	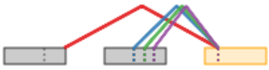	8 5 37 0 56
230	YBX3	ENSG00000060138:10865810-10865932:target	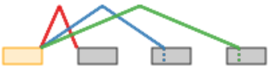	45 0 26 17
231	WNK1	ENSG00000060237:1006645-1006847:source	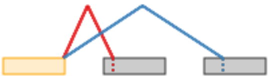	32 0 32
232	WNK1	ENSG00000060237:1016886-1017367:target	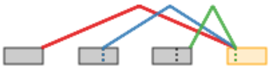	21 0 22
233	WNK1	ENSG00000060237:987181-987527:source	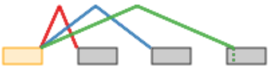	22 0 11
234	ACAA1	ENSG00000060971:38170781-38170879:source	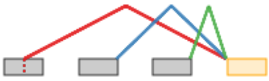	34 0 35
235	ACAA1	ENSG00000060971:38173417-38174043:target	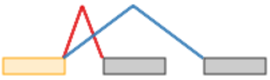	32 0 32
236	HDAC7	ENSG00000061273:48189450-48189633:source	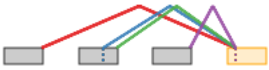	36 0 44
237	HDAC7	ENSG00000061273:48189990-48190081:target	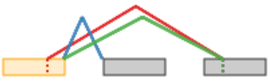	35 0 35
238	GPBP1	ENSG00000062194:56509901-56510023:source	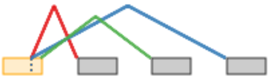	51 0 51
239	GPBP1	ENSG00000062194:56526673-56526796:target	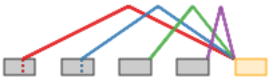	48 0 52

Gene LSV ID LSV Type ← More in Healthy | More in ALL →

#	Gene	LSV ID	LSV Type	← More in Healthy | More in ALL →
240	GPBP1	ENSG00000062194:56531793-56531859:source	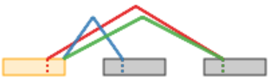	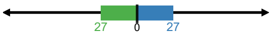
241	GPBP1	ENSG00000062194:56541295-56542311:target	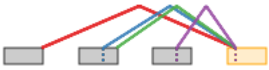	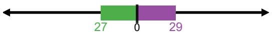
242	WAPAL	ENSG00000062650:88231965-88232101:source	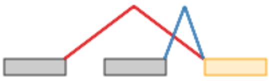	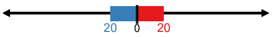
243	U2AF2	ENSG00000063244:56172404-56172683:source	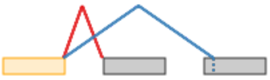	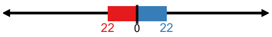
244	U2AF2	ENSG00000063244:56173865-56173984:target	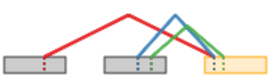	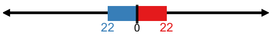
245	CASP8	ENSG00000064012:202141550-202141827:source	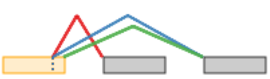	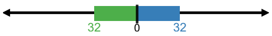
246	CASP8	ENSG00000064012:202149539-202150040:target	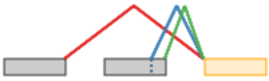	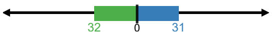
247	TNPO3	ENSG00000064419:128622302-128622378:target	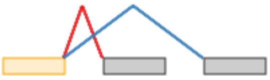	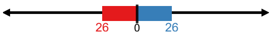
248	BTBD1	ENSG00000064726:83698888-83699518:target	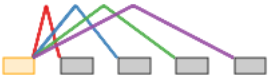	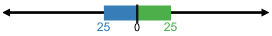
249	ANKRD44	ENSG00000065413:197865143-197865226:target	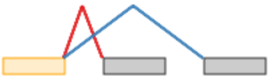	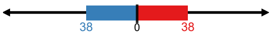
250	ANKRD44	ENSG00000065413:197889920-197889991:source	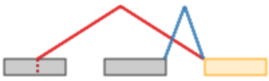	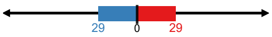
251	ANKRD44	ENSG00000065413:197942536-197943538:target	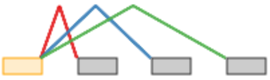	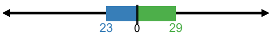
252	ANKRD44	ENSG00000065413:197948093-197948233:source	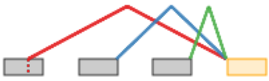	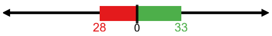
253	ANKRD44	ENSG00000065413:197964201-197964654:target	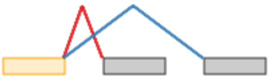	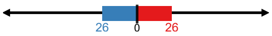
254	ANKRD44	ENSG00000065413:197986056-197986268:source	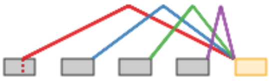	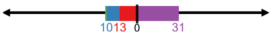
255	KARS	ENSG00000065427:75675462-75675621:source	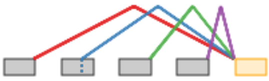	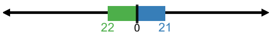
256	SPEN	ENSG00000065526:16265791-16266955:target	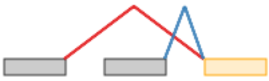	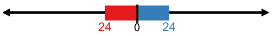
257	CYB5R4	ENSG00000065615:84634189-84634311:source	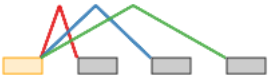	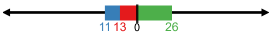
258	CYB5R4	ENSG00000065615:84644314-84644454:target	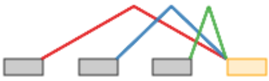	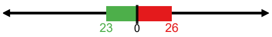
259	FAM107B	ENSG00000065809:14572331-14572514:source	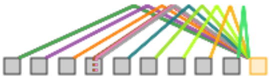	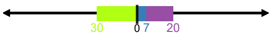

Gene LSV ID LSV Type ← More in Healthy | More in ALL →

#	Gene	LSV ID	LSV Type	← More in Healthy | More in ALL →
260	CDK13	ENSG00000065883:40127725-40127930:source		33 0 33
261	DIP2B	ENSG00000066084:51089050-51089143:target		11 0 25
262	DIP2B	ENSG00000066084:51090844-51091027:source		21 0 21
263	DIP2B	ENSG00000066084:51125152-51125320:source		55 0 55
264	DIP2B	ENSG00000066084:51127918-51127979:target		40 0 40
265	ELOVL1	ENSG00000066322:43830409-43830465:source		23 0 23
266	SPI1	ENSG00000066336:47399860-47400127:target		26 0 26
267	ZNRD1	ENSG00000066379:30029292-30029446:source		64 0 64
268	ZNRD1	ENSG00000066379:30030001-30030110:target		63 0 63
269	ARFGEF1	ENSG00000066777:68189517-68189692:target		24 0 24
270	MTFR1	ENSG00000066855:66639405-66639536:source		26 0 26
271	MTFR1	ENSG00000066855:66651737-66651832:target		28 0 38
272	IDI1	ENSG00000067064:1089939-1090111:source		38 0 38
273	IDI1	ENSG00000067064:1094804-1095110:target		25 0 25
274	SP100	ENSG00000067066:231311534-231311617:source		5 19 0 36
275	SP100	ENSG00000067066:231314276-231314425:target		21 0 21
276	SP100	ENSG00000067066:231327150-231327344:source		20 0 23
277	SP100	ENSG00000067066:231331016-231331069:target		28 0 28
278	SP100	ENSG00000067066:231367781-231367825:source		30 0 30
279	PKM	ENSG00000067225:72492815-72492996:source		15 0 21

Gene LSV ID LSV Type ← More in Healthy | More in ALL →

#	Gene	LSV ID	LSV Type	← More in Healthy | More in ALL →
280	PKM	ENSG00000067225:72498600-72499221:target	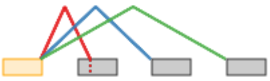	30 0 35
281	TP53BP1	ENSG00000067369:43712816-43712933:source	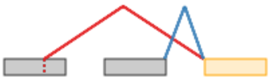	41 0 41
282	TP53BP1	ENSG00000067369:43713870-43714324:target	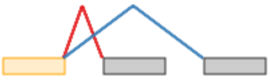	50 0 50
283	TP53BP1	ENSG00000067369:43738585-43738788:source	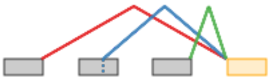	35 0 35
284	TP53BP1	ENSG00000067369:43739353-43739683:target	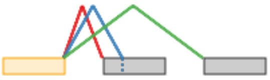	9 39 0 49
285	TP53BP1	ENSG00000067369:43762056-43762264:source	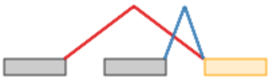	27 0 27
286	TP53BP1	ENSG00000067369:43769791-43769957:target	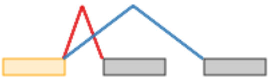	34 0 34
287	RHOA	ENSG00000067560:49398361-49398499:target	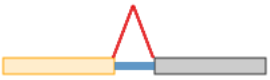	24 0 24
288	MEF2A	ENSG00000068305:100173181-100173378:target	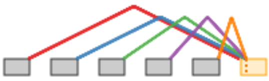	30 0 29
289	TTC7A	ENSG00000068724:47202112-47202242:target	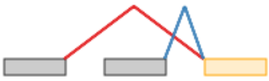	21 0 21
290	TTC7A	ENSG00000068724:47220589-47220667:source	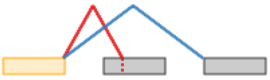	41 0 41
291	TTC7A	ENSG00000068724:47222275-47222338:target	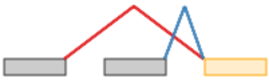	30 0 30
292	TTC7A	ENSG00000068724:47238470-47238574:source	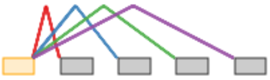	16 15 0 35
293	TTC7A	ENSG00000068724:47250547-47250604:target	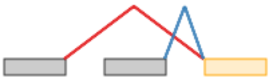	27 0 27
294	TTC7A	ENSG00000068724:47256363-47256523:source	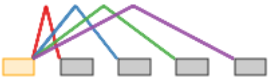	40 0 49
295	TTC7A	ENSG00000068724:47256363-47256523:target	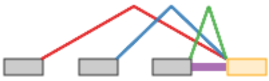	24 0 32
296	TTC7A	ENSG00000068724:47277088-47277185:source	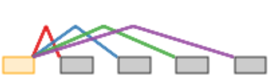	16 6 0 26
297	TTC7A	ENSG00000068724:47277088-47277185:target	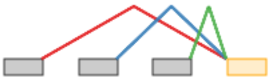	40 0 37
298	TTC7A	ENSG00000068724:47287908-47288110:target	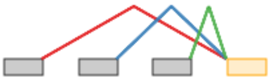	11 5 0 20
299	TTC7A	ENSG00000068724:47300841-47303276:target	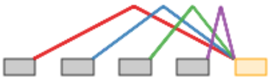	8 7 0 27

Gene LSV ID LSV Type ← More in Healthy | More in ALL →

#	Gene	LSV ID	LSV Type	← More in Healthy | More in ALL →
300	PSME4	ENSG00000068878:54115858-54115949:source	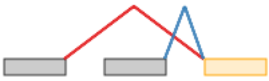	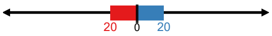
301	PSME4	ENSG00000068878:54119945-54120097:target	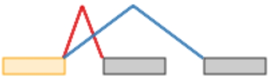	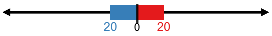
302	BCL3	ENSG00000069399:45251804-45252303:source	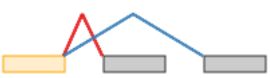	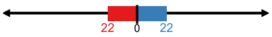
303	BCL3	ENSG00000069399:45254484-45254637:target	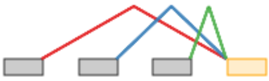	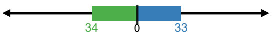
304	KCNAB2	ENSG00000069424:6157329-6157615:source	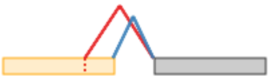	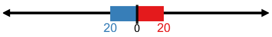
305	CLEC2D	ENSG00000069493:9847356-9848413:target	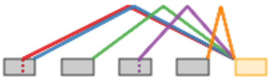	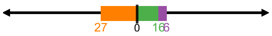
306	ATP1B3	ENSG00000069849:141626009-141626116:source	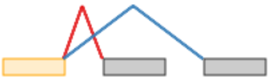	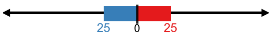
307	ATP1B3	ENSG00000069849:141634812-141634862:target	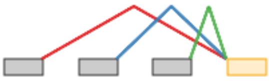	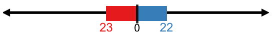
308	GNB5	ENSG00000069966:52413117-52414965:source	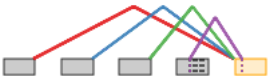	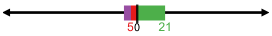
309	UFD1L	ENSG00000070010:19462591-19462623:source	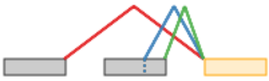	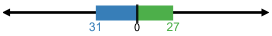
310	UFD1L	ENSG00000070010:19462993-19463125:target	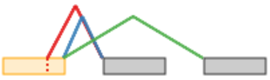	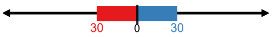
311	DAPP1	ENSG00000070190:100786029-100787278:target	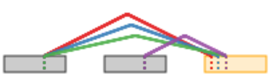	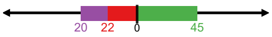
312	POLB	ENSG00000070501:42195972-42196203:source	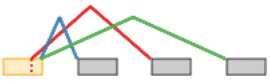	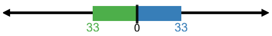
313	POLB	ENSG00000070501:42202471-42202537:target	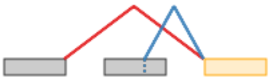	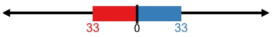
314	POLB	ENSG00000070501:42206534-42206807:source	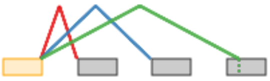	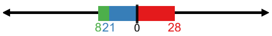
315	POLB	ENSG00000070501:42210037-42210086:target	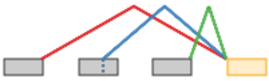	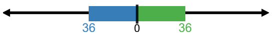
316	POLB	ENSG00000070501:42220130-42220216:source	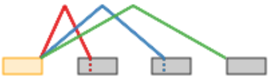	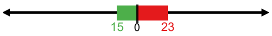
317	POLB	ENSG00000070501:42226454-42226853:source	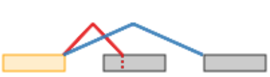	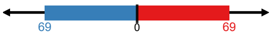
318	POLB	ENSG00000070501:42229081-42229326:target	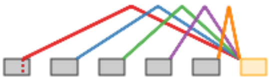	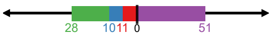
319	GBA2	ENSG00000070610:35740206-35740359:source	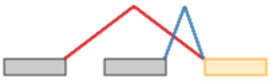	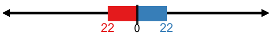
#	Gene	LSV ID	LSV Type	← More in Healthy | More in ALL →

#	Gene	LSV ID	LSV Type	← More in Healthy | More in ALL →
320	PABPC1	ENSG00000070756:101727690-101727829:target	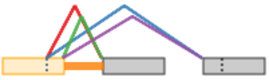	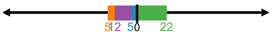
321	CDC42	ENSG00000070831:22404922-22405076:target	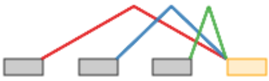	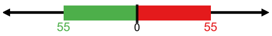
322	CDC42	ENSG00000070831:22413162-22413359:source	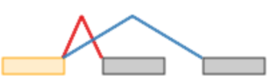	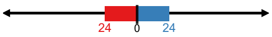
323	ATP2B1	ENSG00000070961:90010579-90010816:target	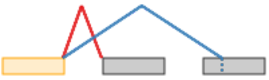	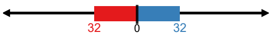
324	MAP4K4	ENSG00000071054:102486757-102487095:source	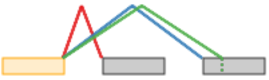	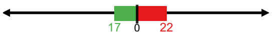
325	MAP4K4	ENSG00000071054:102490109-102490226:target	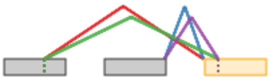	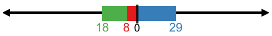
326	MAP4K4	ENSG00000071054:102490544-102490714:source	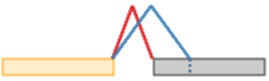	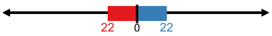
327	ING3	ENSG00000071243:120595613-120595678:source	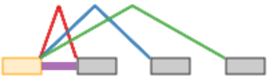	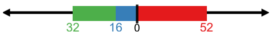
328	TCF3	ENSG00000071564:1611509-1611848:target	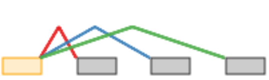	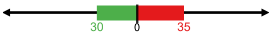
329	TCF3	ENSG00000071564:1615685-1615740:target	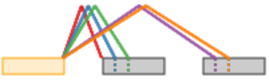	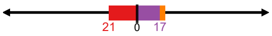
330	TCF3	ENSG00000071564:1627358-1627425:source	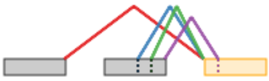	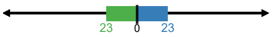
331	AFF4	ENSG00000072364:132223789-132223852:source	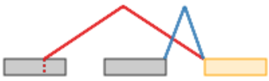	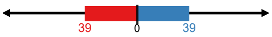
332	AFF4	ENSG00000072364:132227509-132228225:target	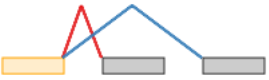	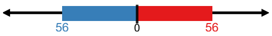
333	AFF4	ENSG00000072364:132232015-132232932:source	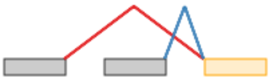	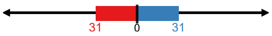
334	AFF4	ENSG00000072364:132234796-132234833:target	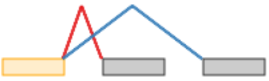	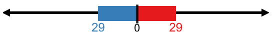
335	AFF4	ENSG00000072364:132298951-132299326:target	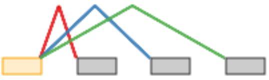	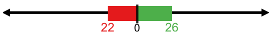
336	STK10	ENSG00000072786:171544485-171544634:target	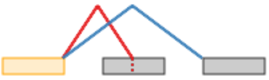	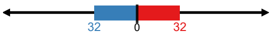
337	FBXW11	ENSG00000072803:171297752-171297862:source	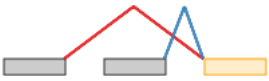	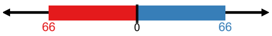
338	FBXW11	ENSG00000072803:171303289-171303295:target	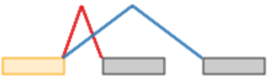	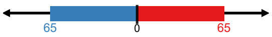
339	PDE8A	ENSG00000073417:85659000-85659540:source	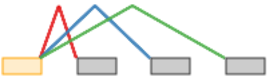	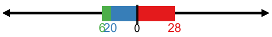

| # | Gene | LSV ID | LSV Type | ← More in Healthy \| More in ALL → |

#	Gene	LSV ID	LSV Type	← More in Healthy | More in ALL →
340	SDHA	ENSG00000073578:224475-224958:source	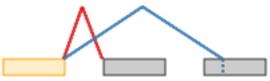	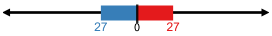
341	SDHA	ENSG00000073578:228300-228448:source	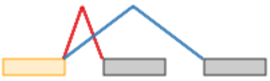	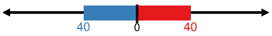
342	SDHA	ENSG00000073578:233592-234253:target	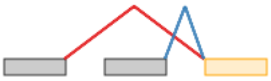	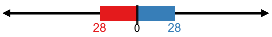
343	KDM5A	ENSG00000073614:389295-394828:source	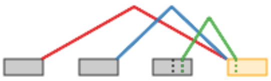	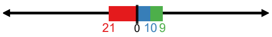
344	KDM5A	ENSG00000073614:416620-417171:source	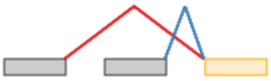	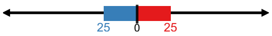
345	KDM5A	ENSG00000073614:442234-442815:target	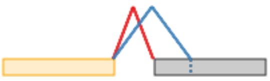	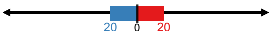
346	ST6GAL1	ENSG00000073849:186756530-186756661:target	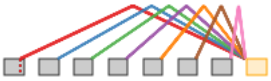	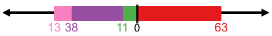
347	PICALM	ENSG00000073921:85694909-85695016:source	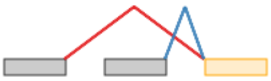	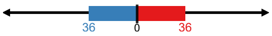
348	PICALM	ENSG00000073921:85707869-85707972:target	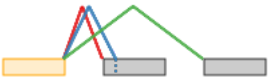	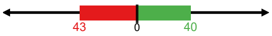
349	PICALM	ENSG00000073921:85779693-85780026:target	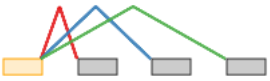	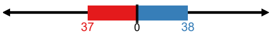
350	NSF	ENSG00000073969:44770269-44770434:target	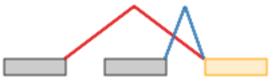	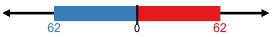
351	CLASP1	ENSG00000074054:122161947-122162010:target	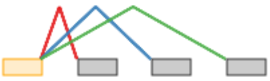	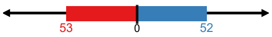
352	CLASP1	ENSG00000074054:122187649-122187753:source	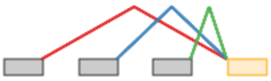	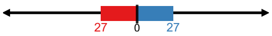
353	CLASP1	ENSG00000074054:122204913-122205083:target	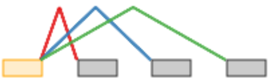	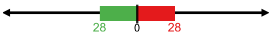
354	CLASP1	ENSG00000074054:122217198-122217701:target	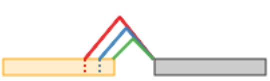	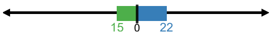
355	ATP2A3	ENSG00000074370:3850685-3851149:target	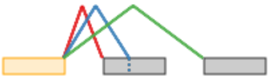	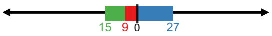
356	ZZEF1	ENSG00000074755:3917284-3917482:target	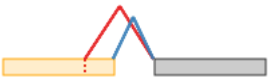	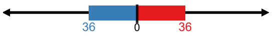
357	ZZEF1	ENSG00000074755:3973978-3974218:source	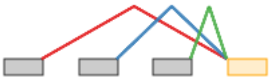	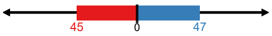
358	ZZEF1	ENSG00000074755:3977444-3977645:target	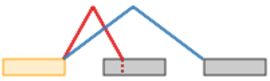	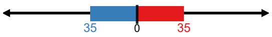
359	ENO1	ENSG00000074800:8928047-8928116:source	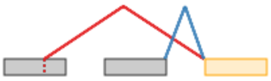	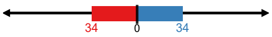

Gene LSV ID LSV Type ← More in Healthy | More in ALL →

#	Gene	LSV ID	LSV Type	← More in Healthy | More in ALL →
360	ENO1	ENSG00000074800:8931411-8932045:target	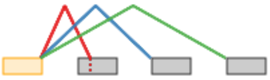	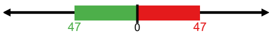
361	SRI	ENSG00000075142:87839870-87840240:source	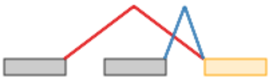	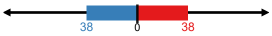
362	SRI	ENSG00000075142:87846437-87846506:target	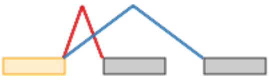	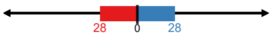
363	EIF4G3	ENSG00000075151:21151593-21151691:source	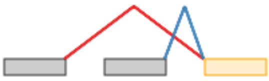	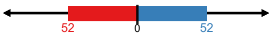
364	EIF4G3	ENSG00000075151:21155640-21155765:target	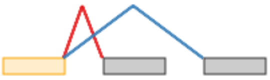	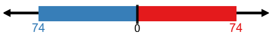
365	ZNF638	ENSG00000075292:71607643-71607850:source	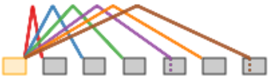	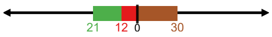
366	MARK3	ENSG00000075413:103871413-103871604:target	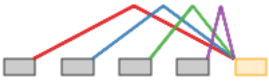	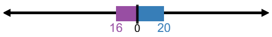
367	MARK3	ENSG00000075413:103928742-103928798:source	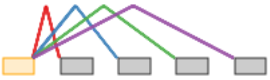	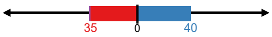
368	MARK3	ENSG00000075413:103928742-103928798:target	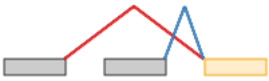	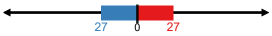
369	MARK3	ENSG00000075413:103931894-103932043:target	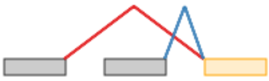	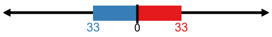
370	MARK3	ENSG00000075413:103932051-103932235:source	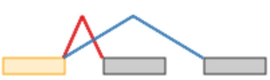	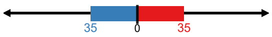
371	MARK3	ENSG00000075413:103932680-103932779:target	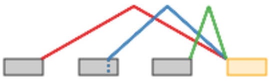	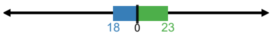
372	SLC25A3	ENSG00000075415:98987753-98988281:source	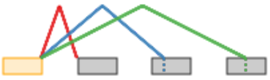	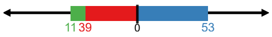
373	SLC25A3	ENSG00000075415:98990979-98991851:target	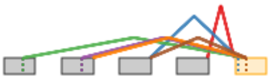	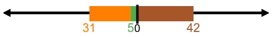
374	FRYL	ENSG00000075539:48512852-48513001:target	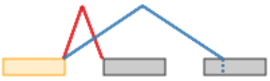	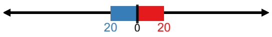
375	FRYL	ENSG00000075539:48530192-48530353:source	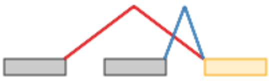	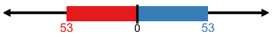
376	FRYL	ENSG00000075539:48536562-48536702:target	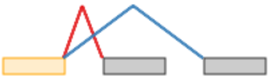	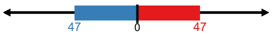
377	FRYL	ENSG00000075539:48542370-48542977:target	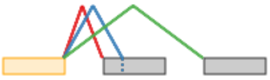	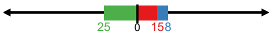
378	FRYL	ENSG00000075539:48550707-48550810:source	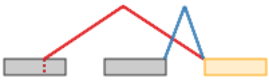	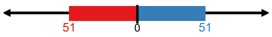
379	FRYL	ENSG00000075539:48552519-48552721:target	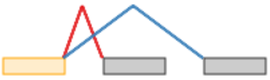	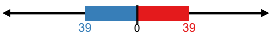
#	Gene	LSV ID	LSV Type	← More in Healthy | More in ALL →

#	Gene	LSV ID	LSV Type	← More in Healthy | More in ALL →
380	FRYL	ENSG00000075539:48575203-48575260:target		60 0 60
381	TMEM131	ENSG00000075568:98382941-98383065:source		10 17 0 30
382	TMEM131	ENSG00000075568:98409753-98410016:source		34 0 34
383	TMEM131	ENSG00000075568:98412681-98412867:target		32 0 32
384	SART3	ENSG00000075856:108930484-108930561:source		49 0 49
385	SART3	ENSG00000075856:108931841-108931979:target		68 0 68
386	ARHGAP15	ENSG00000075884:144192883-144193268:source		26 0 26
387	ARHGAP15	ENSG00000075884:144460988-144461529:source		49 23 0 71
388	BAZ2A	ENSG00000076108:56998857-56999111:target		22 0 22
389	BAZ2A	ENSG00000076108:57000031-57000196:target		21 0 21
390	BAZ2A	ENSG00000076108:57007737-57008090:source		21 0 15
391	ANKRD13A	ENSG00000076513:110454193-110454238:source		33 0 33
392	ANKRD13A	ENSG00000076513:110456944-110457133:target		24 21 0 45
393	ANKRD13A	ENSG00000076513:110468450-110468563:source		46 0 46
394	ANKRD13A	ENSG00000076513:110471602-110471762:target		32 0 32
395	TRAF4	ENSG00000076604:27075529-27075760:source		28 0 28
396	NT5C2	ENSG00000076685:104850693-104850753:target		23 0 23
397	NT5C2	ENSG00000076685:104860992-104861083:source		43 0 43
398	NT5C2	ENSG00000076685:104865463-104865558:target		44 0 40
399	XAB2	ENSG00000076924:7685161-7685332:source		22 0 22

Gene LSV ID LSV Type ← More in Healthy | More in ALL →

#	Gene	LSV ID	LSV Type	← More in Healthy | More in ALL →
400	ARHGEF1	ENSG00000076928:42400450-42400945:source	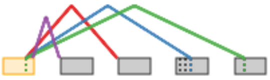	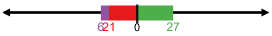
401	ARHGEF1	ENSG00000076928:42402573-42402736:target	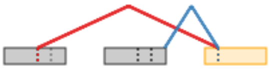	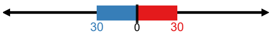
402	STXBP2	ENSG00000076944:7708101-7708472:source	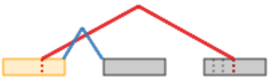	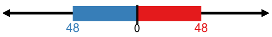
403	DGKD	ENSG00000077044:234346863-234347025:target	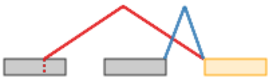	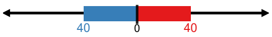
404	DGKD	ENSG00000077044:234356676-234356827:target	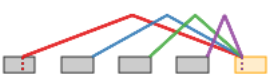	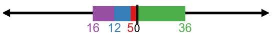
405	TOP2B	ENSG00000077097:25642591-25642716:source	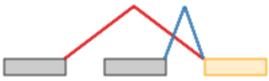	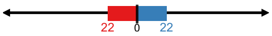
406	TOP2B	ENSG00000077097:25647534-25647613:target	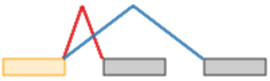	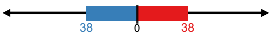
407	TM9SF3	ENSG00000077147:98319416-98319493:source	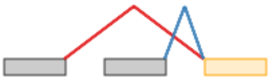	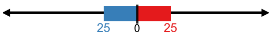
408	TM9SF3	ENSG00000077147:98321735-98321895:target	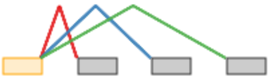	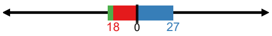
409	NFKB2	ENSG00000077150:104156206-104156246:source	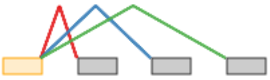	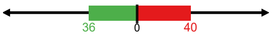
410	NFKB2	ENSG00000077150:104157059-104157165:target	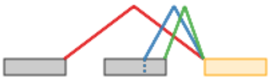	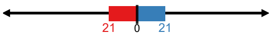
411	DNAJC10	ENSG00000077232:183600976-183601113:source	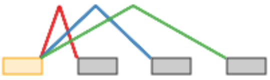	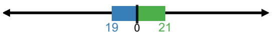
412	IL4R	ENSG00000077238:27353352-27353580:source	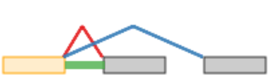	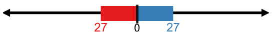
413	IL4R	ENSG00000077238:27356190-27356411:source	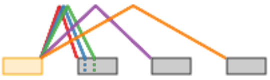	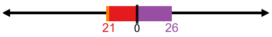
414	IL4R	ENSG00000077238:27363861-27364017:source	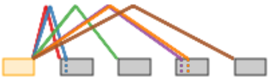	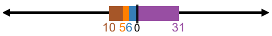
415	IL4R	ENSG00000077238:27370237-27370315:source	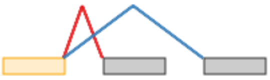	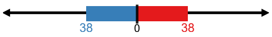
416	IL4R	ENSG00000077238:27373573-27375224:target	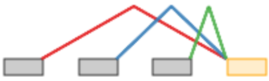	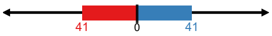
417	USP33	ENSG00000077254:78178885-78179193:source	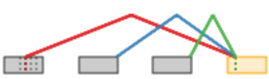	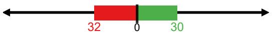
418	USP33	ENSG00000077254:78183225-78183732:target	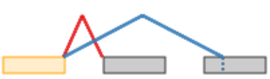	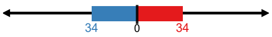
419	USP33	ENSG00000077254:78207302-78207433:source	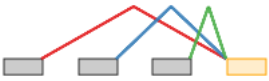	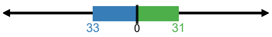

Gene LSV ID LSV Type ← More in Healthy | More in ALL →

#	Gene	LSV ID	LSV Type	← More in Healthy | More in ALL →
420	FAM76B	ENSG00000077458:95511986-95512121:source	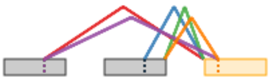	25 7 0 29 10
421	FAM76B	ENSG00000077458:95512653-95512851:target	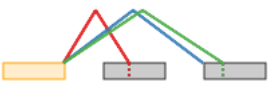	41 0 31 10
422	UBE2K	ENSG00000078140:39776454-39776553:source	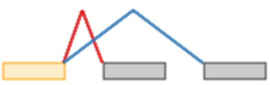	24 0 24
423	NRD1	ENSG00000078618:52258049-52258096:target	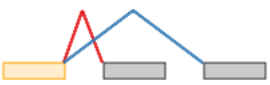	21 0 21
424	PCM1	ENSG00000078674:17830182-17830196:source	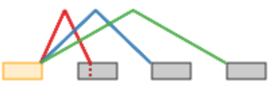	38 0 43
425	PCM1	ENSG00000078674:17842956-17843128:target	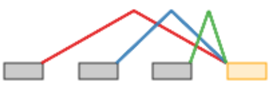	38 0 19 18
426	PCM1	ENSG00000078674:17850992-17851128:source	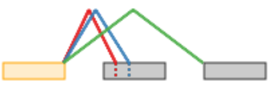	47 0 41
427	PCM1	ENSG00000078674:17867056-17867253:target	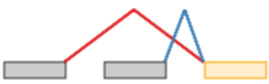	35 0 35
428	SP140	ENSG00000079263:231175869-231175946:target	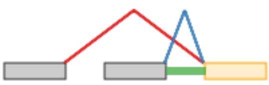	13 8 0 24
429	SAR1A	ENSG00000079332:71921614-71921687:source	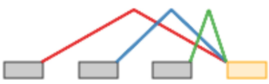	27 0 28
430	CIC	ENSG00000079432:42796238-42796359:source	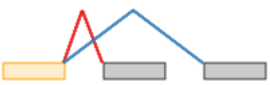	35 0 35
431	CIC	ENSG00000079432:42796718-42797011:target	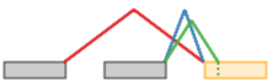	51 0 41 8
432	FDFT1	ENSG00000079459:11660120-11660440:source	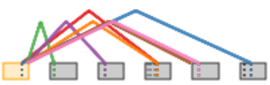	10 0 20
433	DNM2	ENSG00000079805:10906048-10906422:source	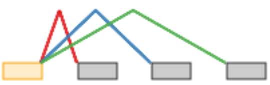	15 0 25
434	STX7	ENSG00000079950:132767006-132781989:source	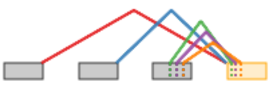	6 12 0 22
435	STX7	ENSG00000079950:132785132-132785214:target	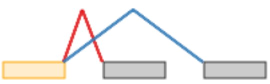	38 0 38
436	RAB21	ENSG00000080371:72176194-72176438:target	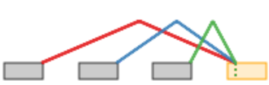	23 0 27
437	SMARCA2	ENSG00000080503:2161686-2161903:source	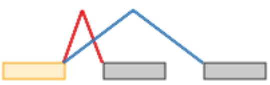	26 0 26
438	SMARCA2	ENSG00000080503:2181571-2181676:target	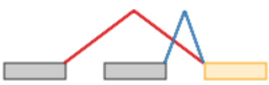	25 0 25
439	SMARCA2	ENSG00000080503:2182141-2182242:source	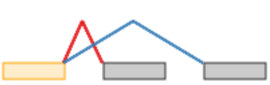	29 0 29

| # | Gene | LSV ID | LSV Type | ← More in Healthy \| More in ALL → |

#	Gene	LSV ID	LSV Type	← More in Healthy | More in ALL →
440	PSEN1	ENSG00000080815:73614651-73614814:source	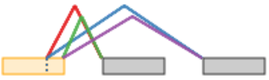	21 0 21
441	PSEN1	ENSG00000080815:73637505-73637758:target	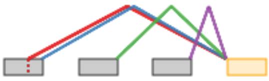	21 0 20
442	CLDND1	ENSG00000080822:98235896-98236189:target	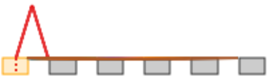	732 0 42
443	HSP90AA1	ENSG00000080824:102549002-102549639:target	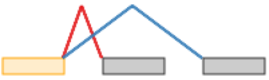	40 0 40
444	DLGAP4	ENSG00000080845:35125108-35125469:source	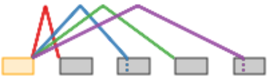	50 0 47
445	DLGAP4	ENSG00000080845:35128600-35128763:target	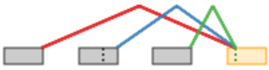	52 0 53
446	PCNP	ENSG00000081154:101292939-101293123:source	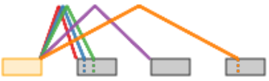	43 0 39
447	PCNP	ENSG00000081154:101298433-101298848:target	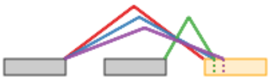	45 0 47
448	MEF2C	ENSG00000081189:88024310-88024445:target	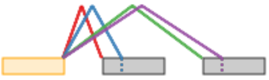	21 0 17
449	MEF2C	ENSG00000081189:88047674-88047860:source	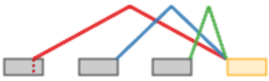	53 0 52
450	MEF2C	ENSG00000081189:88119613-88120092:source	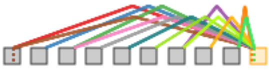	17 7 0 6 9
451	PTPRC	ENSG00000081237:198661476-198662335:source	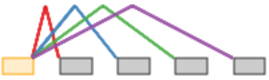	29 0 21
452	PTPRC	ENSG00000081237:198668693-198669275:source	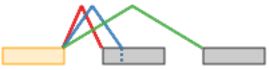	15 50 18
453	PTPRC	ENSG00000081237:198668693-198669275:target	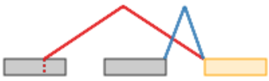	25 0 25
454	PTPRC	ENSG00000081237:198672427-198673003:target	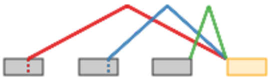	20 0 15
455	PTPRC	ENSG00000081237:198681919-198682201:target	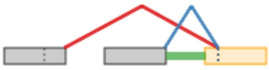	17 16 0 38
456	PTPRC	ENSG00000081237:198698266-198698300:source	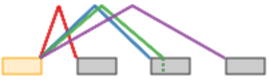	525 0 33
457	PTPRC	ENSG00000081237:198700746-198700855:target	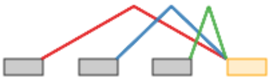	12 22 0 36
458	ZNF506	ENSG00000081665:19932410-19932560:target	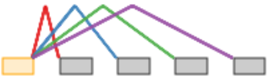	22 0 21
459	C1QTNF3	ENSG00000082196:34019553-34020847:source	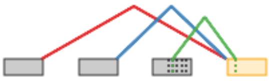	21 0 27

| # | Gene | LSV ID | LSV Type | ← More in Healthy \| More in ALL → |

#	Gene	LSV ID	LSV Type	← More in Healthy | More in ALL →
460	ME2	ENSG00000082212:48458628-48458730:source	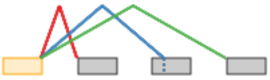	62 0 63
461	ME2	ENSG00000082212:48466500-48466756:target	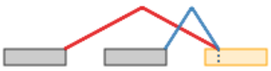	56 0 56
462	CCNT2	ENSG00000082258:135703671-135704035:source	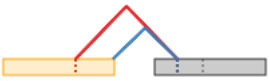	35 0 35
463	CCNT2	ENSG00000082258:135703671-135704035:target	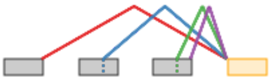	8 26 0 37
464	TRAF5	ENSG00000082512:211542271-211542934:target	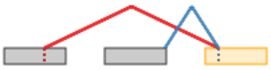	20 0 20
465	GSK3B	ENSG00000082701:119624602-119624699:source	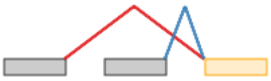	31 0 31
466	GSK3B	ENSG00000082701:119634891-119635021:target	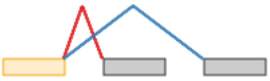	31 0 31
467	GSK3B	ENSG00000082701:119720893-119721086:target	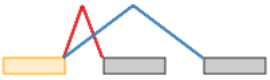	39 0 39
468	XPO1	ENSG00000082898:61749746-61749818:source	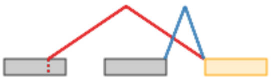	20 0 20
469	KAT6A	ENSG00000083168:41905896-41906820:source	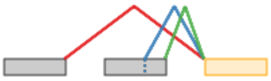	9 16 0 27
470	ZCCHC6	ENSG00000083223:88916191-88916516:source	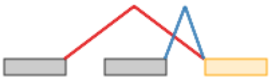	26 0 26
471	ZCCHC6	ENSG00000083223:88918001-88918116:target	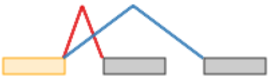	27 0 27
472	ZCCHC6	ENSG00000083223:88932134-88932191:source	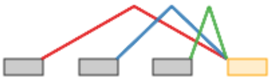	46 0 49
473	ZCCHC6	ENSG00000083223:88934499-88934585:target	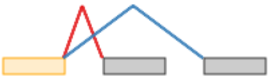	56 0 56
474	TNPO1	ENSG00000083312:72147032-72147146:source	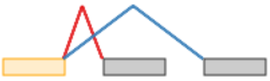	20 0 20
475	TNPO1	ENSG00000083312:72157635-72157742:target	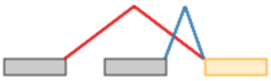	22 0 22
476	TNPO1	ENSG00000083312:72189204-72189362:target	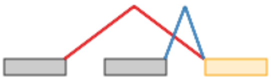	24 0 24
477	DIS3	ENSG00000083520:73352325-73352518:source	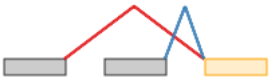	20 0 20
478	CYLD	ENSG00000083799:50813576-50813955:source	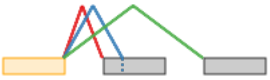	21 0 23
479	CYLD	ENSG00000083799:50816236-50816377:target	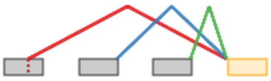	23 0 24

Gene LSV ID LSV Type ← More in Healthy | More in ALL →

#	Gene	LSV ID	LSV Type	← More in Healthy | More in ALL →
480	YTHDC1	ENSG00000083896:69185841-69185923:source	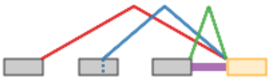	42 0 9 32
481	YTHDC1	ENSG00000083896:69188210-69188633:target	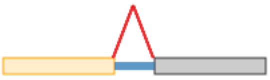	60 0 60
482	SMAP2	ENSG00000084070:40839369-40839895:source	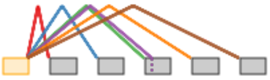	10 9 0 25
483	SMAP2	ENSG00000084070:40872408-40872541:target	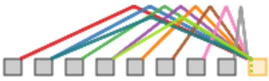	10 6 0 20
484	PPIE	ENSG00000084072:40214575-40214760:target	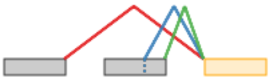	81 0 77
485	APLP2	ENSG00000084234:129992200-129992408:source	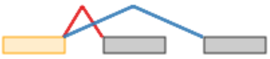	24 0 24
486	APLP2	ENSG00000084234:130005458-130005610:source	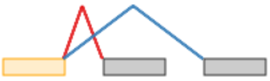	58 0 58
487	APLP2	ENSG00000084234:130010293-130010378:target	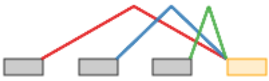	59 0 59
488	WBP11	ENSG00000084463:14947471-14947670:source	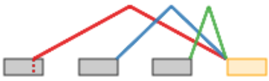	22 6 0 31
489	WBP11	ENSG00000084463:14949741-14949937:target	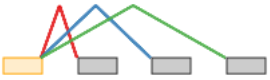	34 0 35
490	EIF3I	ENSG00000084623:32690011-32690076:source	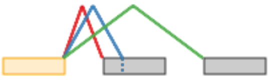	31 0 31
491	EIF3I	ENSG00000084623:32694100-32694210:target	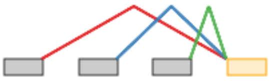	40 0 42
492	RRN3	ENSG00000085721:15185172-15185228:target	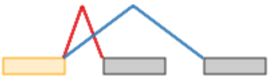	23 0 23
493	EPS15	ENSG00000085832:51912632-51912777:source	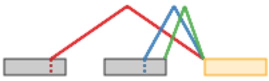	34 0 26
494	EPS15	ENSG00000085832:51926616-51926822:target	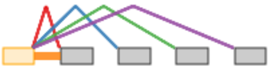	19 38 0 56
495	EPS15	ENSG00000085832:51946945-51946986:source	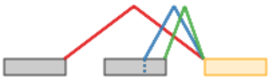	18 23 0 42
496	EPS15	ENSG00000085832:51984871-51985000:target	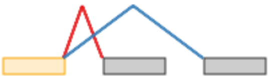	34 0 34
497	ATG16L1	ENSG00000085978:234172638-234172747:source	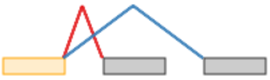	21 0 21
498	ATG16L1	ENSG00000085978:234178648-234178713:target	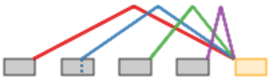	21 0 21
499	ATG16L1	ENSG00000085978:234191328-234191641:source	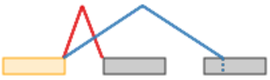	21 0 21

| # | Gene | LSV ID | LSV Type | ← More in Healthy \| More in ALL → |

#	Gene	LSV ID	LSV Type	← More in Healthy | More in ALL →
500	DNAJA1	ENSG00000086061:33026473-33026614:source	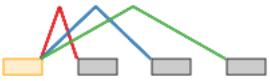	25 8 0 33
501	DNAJA1	ENSG00000086061:33029883-33029987:target	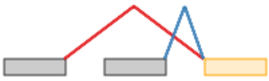	34 0 34
502	DNAJA1	ENSG00000086061:33030438-33030665:target	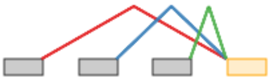	22 0 24
503	NFX1	ENSG00000086102:33303189-33303266:source	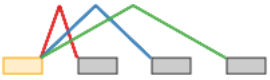	15 0 21
504	NFX1	ENSG00000086102:33352644-33352717:source	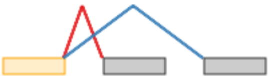	36 0 36
505	NFX1	ENSG00000086102:33354849-33354890:target	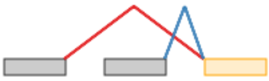	21 0 21
506	EIF2AK1	ENSG00000086232:6088438-6088475:target	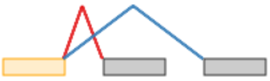	27 0 27
507	LAT2	ENSG00000086730:73630277-73630399:target	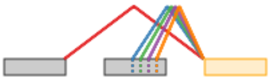	26 0 14 12
508	ALG9	ENSG00000086848:111708191-111708338:source	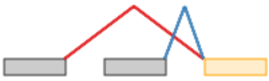	25 0 25
509	SRRT	ENSG00000087087:100485324-100485480:source	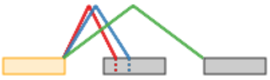	31 0 30
510	UIMC1	ENSG00000087206:176395556-176395890:target	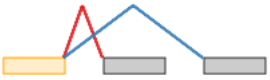	28 0 28
511	UIMC1	ENSG00000087206:176396054-176396292:source	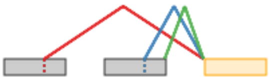	47 0 47
512	UIMC1	ENSG00000087206:176396942-176397867:target	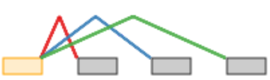	26 0 31
513	SH3BP2	ENSG00000087266:2834058-2834139:target	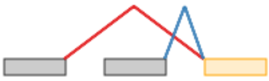	27 0 27
514	SF3B2	ENSG00000087365:65824309-65824426:target	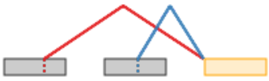	22 0 22
515	GNAS	ENSG00000087460:57475004-57475465:target	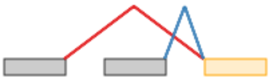	26 0 26
516	DNM1L	ENSG00000087470:32891198-32891230:target	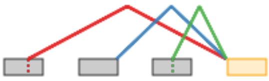	20 0 13
517	ANKRD10	ENSG00000088448:111535458-111536140:target	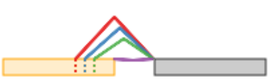	13 21 0 37
518	ANKRD10	ENSG00000088448:111545544-111545610:source	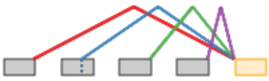	40 0 18 18
519	ANKRD10	ENSG00000088448:111558380-111558471:target	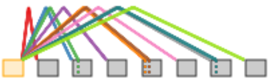	20 0

Gene LSV ID LSV Type ← More in Healthy | More in ALL →

#	Gene	LSV ID	LSV Type	← More in Healthy | More in ALL →
520	XRN2	ENSG00000088930:21307128-21307239:source	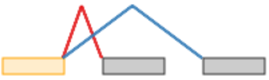	44 0 44
521	XRN2	ENSG00000088930:21311119-21311177:target	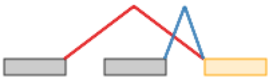	50 0 50
522	XRN2	ENSG00000088930:21335427-21335510:source	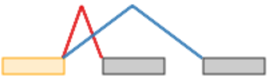	27 0 27
523	XRN2	ENSG00000088930:21336718-21336815:target	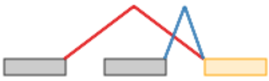	30 0 30
524	XRN2	ENSG00000088930:21349101-21349228:target	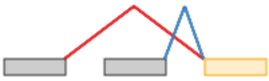	20 0 20
525	SNX5	ENSG00000089006:17930935-17931040:source	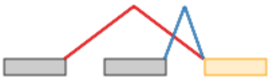	32 0 32
526	SNX5	ENSG00000089006:17932144-17932239:target	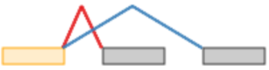	36 0 36
527	ANAPC5	ENSG00000089053:121766060-121766300:source	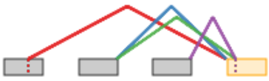	26 0 13 14
528	ANAPC5	ENSG00000089053:121768386-121768475:source	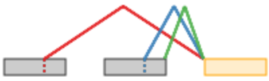	24 0 25
529	ANAPC5	ENSG00000089053:121773320-121773526:target	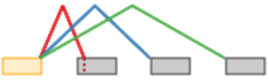	20 0 28
530	SLC23A2	ENSG00000089057:4865399-4865469:source	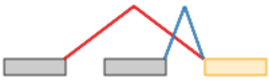	20 0 20
531	SLC23A2	ENSG00000089057:4880201-4880358:target	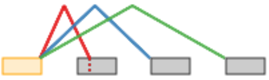	25 0 29
532	KDM2B	ENSG00000089094:121890923-121891147:source	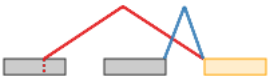	47 0 47
533	GCN1L1	ENSG00000089154:120568450-120568557:source	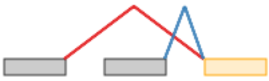	45 0 45
534	GCN1L1	ENSG00000089154:120569730-120569826:target	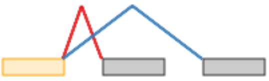	54 0 54
535	GCN1L1	ENSG00000089154:120606012-120606218:source	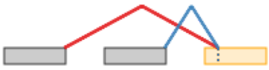	63 0 63
536	ERP29	ENSG00000089248:112451120-112451413:source	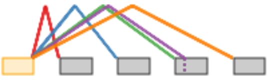	21 0 21
537	FUS	ENSG00000089280:31199473-31199678:target	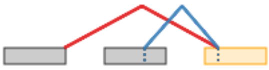	34 0 34
538	GRAMD1A	ENSG00000089351:35502378-35502458:source	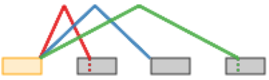	11 35 0 48
539	GRAMD1A	ENSG00000089351:35504445-35504488:target	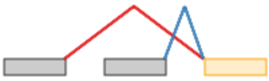	41 0 41

| # | Gene | LSV ID | LSV Type | ← More in Healthy \| More in ALL → |

#	Gene	LSV ID	LSV Type	← More in Healthy | More in ALL →
540	GANAB	ENSG00000089597:62394332-62394406:source	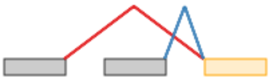	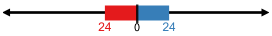
541	GANAB	ENSG00000089597:62394761-62394825:target	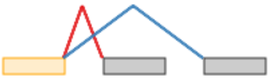	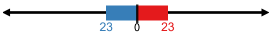
542	DDX24	ENSG00000089737:94524168-94524243:target	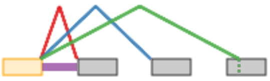	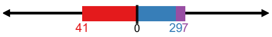
543	PAPOLA	ENSG00000090060:97009066-97009230:source	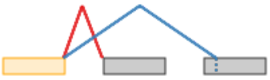	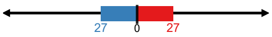
544	PAPOLA	ENSG00000090060:97022512-97022750:source	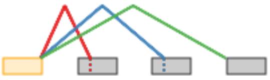	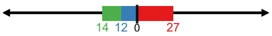
545	PAPOLA	ENSG00000090060:97022512-97022750:target	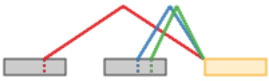	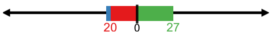
546	CCNK	ENSG00000090061:99947675-99947951:source	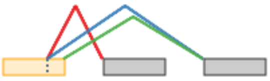	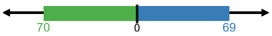
547	CCNK	ENSG00000090061:99958963-99959440:target	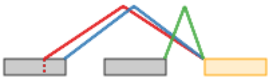	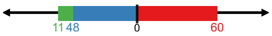
548	MAEA	ENSG00000090316:1305767-1306374:source	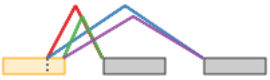	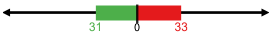
549	STRN4	ENSG00000090372:47231151-47231383:target	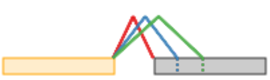	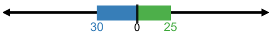
550	DNAJB11	ENSG00000090520:186295457-186295849:source	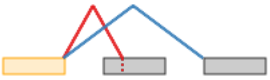	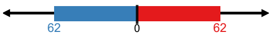
551	DNAJB11	ENSG00000090520:186299784-186299866:target	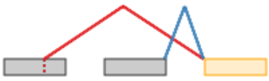	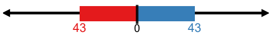
552	PABPC4	ENSG00000090621:40029118-40029413:source	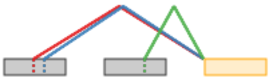	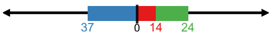
553	PABPC4	ENSG00000090621:40029905-40030214:target	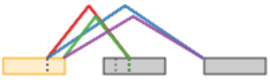	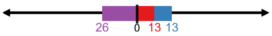
554	PABPC4	ENSG00000090621:40035535-40035674:source	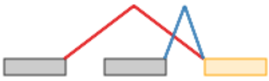	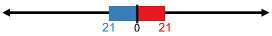
555	USP48	ENSG00000090686:22032220-22032330:target	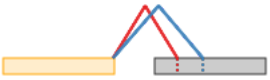	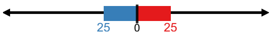
556	USP48	ENSG00000090686:22056197-22056325:source	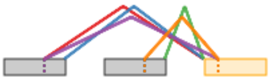	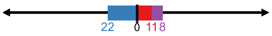
557	USP48	ENSG00000090686:22083039-22083195:source	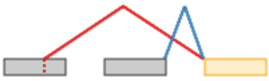	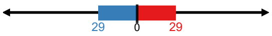
558	USP48	ENSG00000090686:22083954-22084276:target	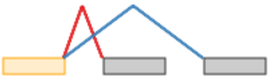	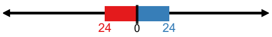
559	GLG1	ENSG00000090863:74503883-74503971:target	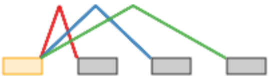	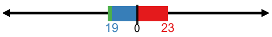
#	Gene	LSV ID	LSV Type	← More in Healthy | More in ALL →

#	Gene	LSV ID	LSV Type	← More in Healthy | More in ALL →
560	GLG1	ENSG00000090863:74508070-74508530:target	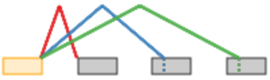	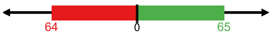
561	OSBPL8	ENSG00000091039:76804344-76804414:target	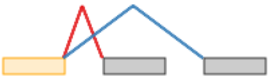	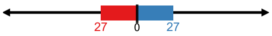
562	TXNL1	ENSG00000091164:54291519-54291692:source	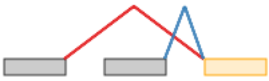	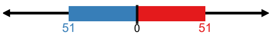
563	TXNL1	ENSG00000091164:54293592-54293688:target	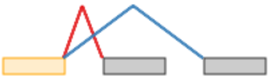	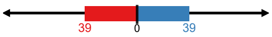
564	SEL1L3	ENSG00000091490:25759156-25759228:target	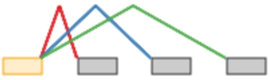	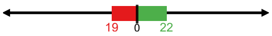
565	SEL1L3	ENSG00000091490:25760563-25761599:target	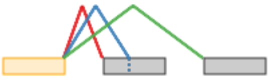	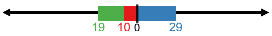
566	SEL1L3	ENSG00000091490:25848916-25849486:target	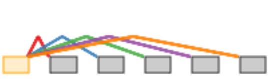	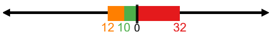
567	CDV3	ENSG00000091527:133305407-133305566:source	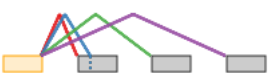	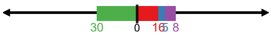
568	NLRP1	ENSG00000091592:5402747-5403270:source	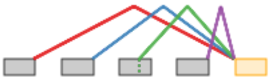	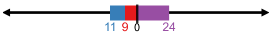
569	NLRP1	ENSG00000091592:5442735-5443010:target	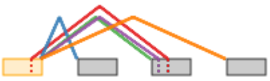	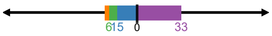
570	SCFD1	ENSG00000092108:31175029-31175091:source	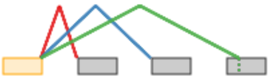	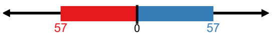
571	HECTD1	ENSG00000092148:31591727-31592253:target	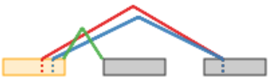	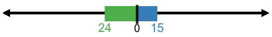
572	HECTD1	ENSG00000092148:31596725-31597212:source	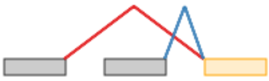	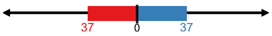
573	HECTD1	ENSG00000092148:31602444-31602620:target	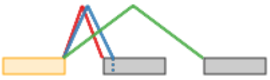	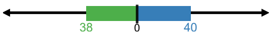
574	HECTD1	ENSG00000092148:31676222-31677010:target	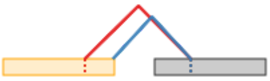	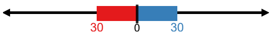
575	HNRNPC	ENSG00000092199:21737457-21737653:target	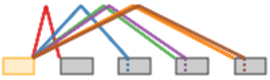	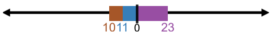
576	SUPT16H	ENSG00000092201:21840033-21840203:source	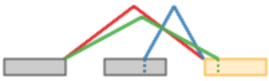	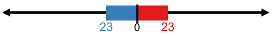
577	SUPT16H	ENSG00000092201:21841497-21841589:target	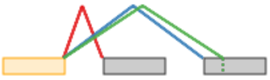	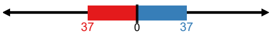
578	TRPM7	ENSG00000092439:50896144-50897341:target	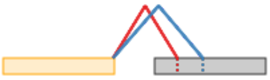	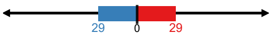
579	SNAP23	ENSG00000092531:42805153-42805323:source	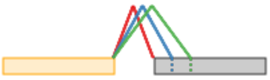	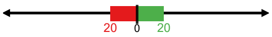

Gene LSV ID LSV Type ← More in Healthy | More in ALL →

#	Gene	LSV ID	LSV Type	← More in Healthy | More in ALL →
580	SNAP23	ENSG00000092531:42807435-42807552:source	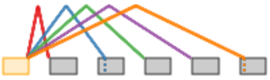	20 0 21
581	EZR	ENSG00000092820:159206341-159206615:source	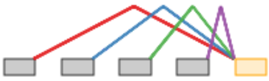	16 6 0 24
582	MYL6	ENSG00000092841:56552465-56552495:source	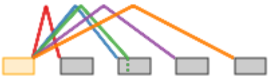	33 0 32
583	XXbac-B461K10.4	ENSG00000093100:18273968-18274067:source	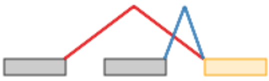	29 0 29
584	XXbac-B461K10.4	ENSG00000093100:18293469-18294263:target	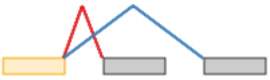	20 0 20
585	SUCO	ENSG00000094975:172571836-172572345:target	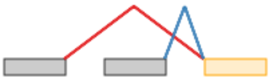	21 0 21
586	MAP3K1	ENSG00000095015:56181759-56181890:source	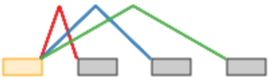	40 0 41
587	MAP3K1	ENSG00000095015:56184053-56184184:target	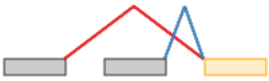	30 0 30
588	DHPS	ENSG00000095059:12790271-12790357:target	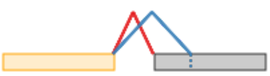	22 0 22
589	ARCN1	ENSG00000095139:118443105-118443265:source	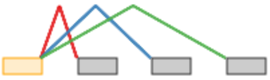	44 0 44
590	ARCN1	ENSG00000095139:118453894-118454073:target	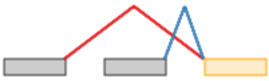	36 0 36
591	NUP188	ENSG00000095319:131733037-131733237:target	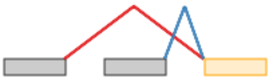	30 0 30
592	NUP188	ENSG00000095319:131757135-131757479:target	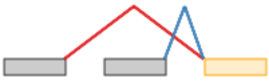	23 0 23
593	NUP188	ENSG00000095319:131760430-131760507:source	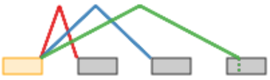	20 11 0 34
594	NUP188	ENSG00000095319:131761880-131762084:target	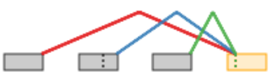	15 0 22
595	CWF19L1	ENSG00000095485:102013178-102014376:target	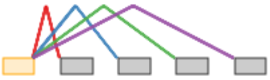	21 0 20
596	BTAF1	ENSG00000095564:93711160-93711327:source	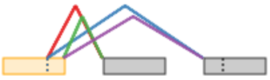	21 0 25
597	BTAF1	ENSG00000095564:93718822-93719039:source	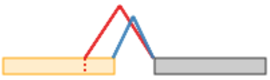	21 0 21
598	BTAF1	ENSG00000095564:93756126-93756327:source	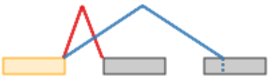	34 0 34
599	IKZF5	ENSG00000095574:124757743-124758187:source	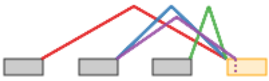	627 0 30

Gene LSV ID LSV Type ← More in Healthy | More in ALL →

#	Gene	LSV ID	LSV Type	← More in Healthy | More in ALL →
600	BLNK	ENSG00000095585:97969594-97970059:source	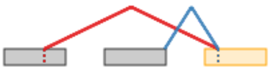	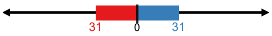 31 0 31
601	BLNK	ENSG00000095585:97975605-97976490:target	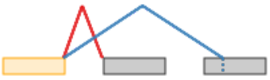	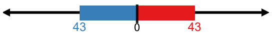 43 0 43
602	BLNK	ENSG00000095585:98002491-98002540:target	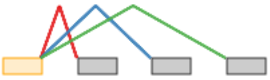	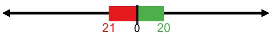 21 0 20
603	WAC	ENSG00000095787:28824195-28824686:source	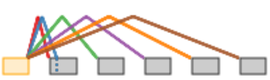	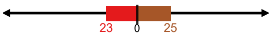 23 0 25
604	SRPK1	ENSG00000096063:35837366-35837678:source	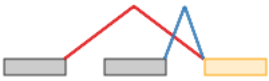	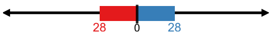 28 0 28
605	ITPR3	ENSG00000096433:33638448-33638592:source	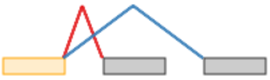	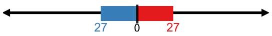 27 0 27
606	HNRNPH3	ENSG00000096746:70096563-70097090:source	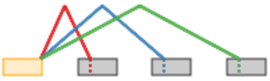	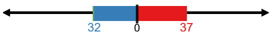 32 0 37
607	HNRNPH3	ENSG00000096746:70098097-70098444:target	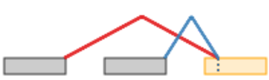	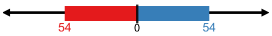 54 0 54
608	HNRNPH3	ENSG00000096746:70098563-70098983:target	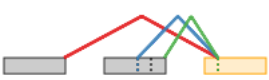	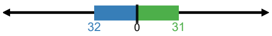 32 0 31
609	ABLIM1	ENSG00000099204:116205071-116205162:source	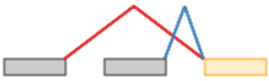	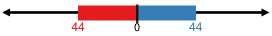 44 0 44
610	ABLIM1	ENSG00000099204:116211383-116211430:target	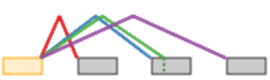	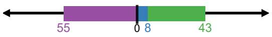 55 0 8 43
611	RAB18	ENSG00000099246:27798804-27799419:source	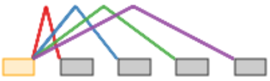	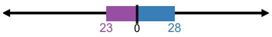 23 0 28
612	RAB18	ENSG00000099246:27815757-27815818:source	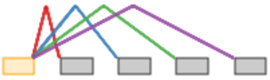	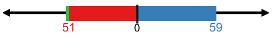 51 0 59
613	RAB18	ENSG00000099246:27821436-27821508:target	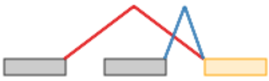	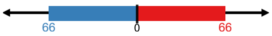 66 0 66
614	RAB18	ENSG00000099246:27822664-27822782:target	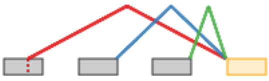	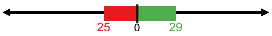 25 0 29
615	CIRBP	ENSG00000099622:1269330-1269409:source	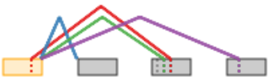	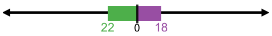 22 0 18
616	CIRBP	ENSG00000099622:1274306-1274594:target	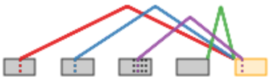	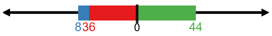 836 0 44
617	HNRNPM	ENSG00000099783:8536210-8536311:source	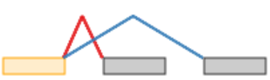	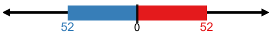 52 0 52
618	HNRNPM	ENSG00000099783:8539051-8539128:target	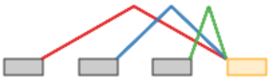	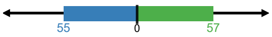 55 0 57
619	MKNK2	ENSG00000099875:2043498-2043581:source	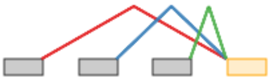	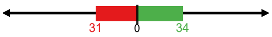 31 0 34

Gene LSV ID LSV Type ← More in Healthy | More in ALL →

#	Gene	LSV ID	LSV Type	← More in Healthy | More in ALL →
620	MKNK2	ENSG00000099875:2050800-2050946:target	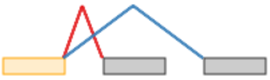	60 0 60
621	MED15	ENSG00000099917:20922808-20922918:source	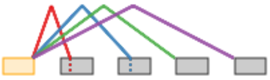	23 0 29
622	SF3A1	ENSG00000099995:30738536-30738868:source	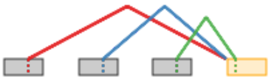	21 0 21
623	SF3A1	ENSG00000099995:30738536-30738868:target	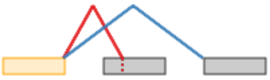	29 0 29
624	MAPK1	ENSG00000100030:22126938-22127271:target	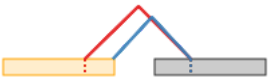	29 0 29
625	MAPK1	ENSG00000100030:22153301-22153417:target	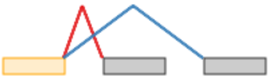	45 0 45
626	CYTH4	ENSG00000100055:37695267-37695347:source	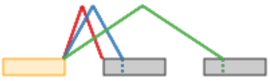	18 0 24
627	DGCR14	ENSG00000100056:19131570-19132197:target	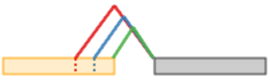	45 0 36
628	ADRBK2	ENSG00000100077:26099477-26099543:target	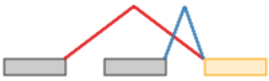	34 0 34
629	ADRBK2	ENSG00000100077:26110375-26110537:source	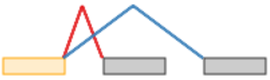	59 0 59
630	ADRBK2	ENSG00000100077:26117251-26117364:target	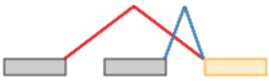	51 0 51
631	ADRBK2	ENSG00000100077:26118256-26125261:target	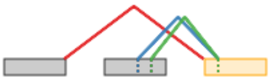	15 18 0 36
632	GGA1	ENSG00000100083:38010140-38010281:target	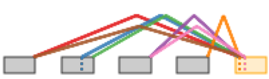	51 0 52
633	HIRA	ENSG00000100084:19362720-19363315:target	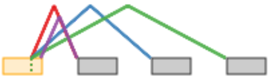	11 5 0 20
634	HIRA	ENSG00000100084:19373044-19373259:source	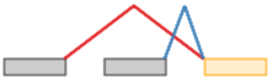	35 0 35
635	HIRA	ENSG00000100084:19376007-19376077:target	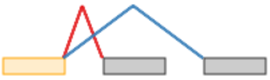	42 0 42
636	HIRA	ENSG00000100084:19393309-19393403:target	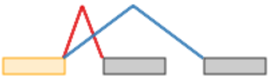	73 0 73
637	HIRA	ENSG00000100084:19395797-19396116:target	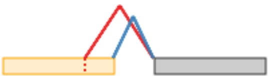	25 0 25
638	HPS4	ENSG00000100099:26871336-26873102:source	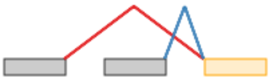	21 0 21
639	PIK3IP1	ENSG00000100100:31677579-31679274:source	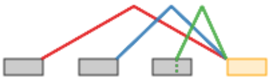	46 0 49

| # | Gene | LSV ID | LSV Type | ← More in Healthy \| More in ALL → |

#	Gene	LSV ID	LSV Type	← More in Healthy | More in ALL →
640	PIK3IP1	ENSG00000100100:31685485-31685753:target	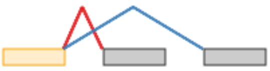	56 0 56
641	SRRD	ENSG00000100104:26887429-26890624:target	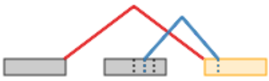	20 0 20
642	PATZ1	ENSG00000100105:31731404-31731849:target	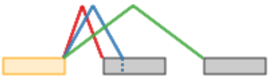	22 0 20
643	NHP2L1	ENSG00000100138:42076248-42076368:source	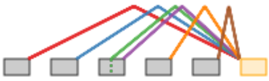	80 0 54 25
644	NHP2L1	ENSG00000100138:42078360-42078626:target	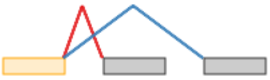	71 0 71
645	DDX17	ENSG00000100201:38883878-38884120:target	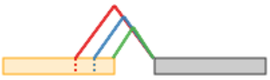	28 0 28
646	DDX17	ENSG00000100201:38888057-38888120:target	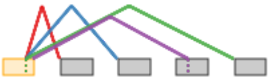	51 0 48
647	RTCB	ENSG00000100220:32797549-32797890:source	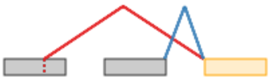	65 0 65
648	RTCB	ENSG00000100220:32803866-32804229:target	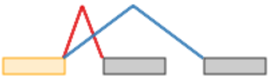	62 0 62
649	GTPBP1	ENSG00000100226:39111912-39112245:target	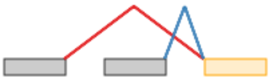	21 0 21
650	GTPBP1	ENSG00000100226:39120302-39120416:target	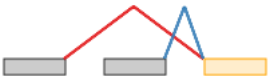	22 0 22
651	POLDIP3	ENSG00000100227:42995704-42995799:source	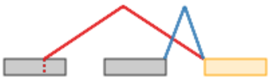	43 0 43
652	POLDIP3	ENSG00000100227:42998774-42999166:target	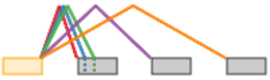	36 0 35
653	SBF1	ENSG00000100241:50894921-50895102:source	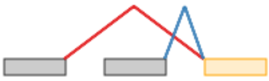	25 0 25
654	SBF1	ENSG00000100241:50897684-50897821:target	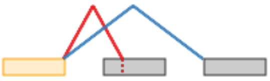	21 0 21
655	AP1B1	ENSG00000100280:29723669-29724884:source	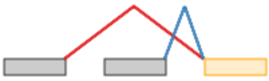	23 0 23
656	AP1B1	ENSG00000100280:29726367-29726521:target	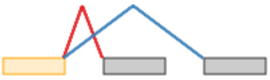	23 0 23
657	AP1B1	ENSG00000100280:29736645-29736846:source	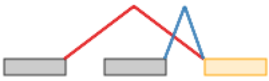	37 0 37
658	AP1B1	ENSG00000100280:29738274-29738372:target	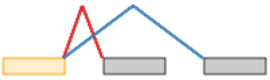	34 0 34
659	AP1B1	ENSG00000100280:29763196-29763264:source	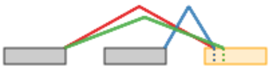	21 0 24

| # | Gene | LSV ID | LSV Type | ← More in Healthy \| More in ALL → |

#	Gene	LSV ID	LSV Type	← More in Healthy | More in ALL →
660	HMGXB4	ENSG00000100281:35684231-35684400:source	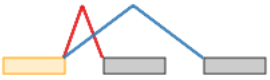	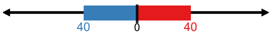
661	HMGXB4	ENSG00000100281:35689600-35691800:target	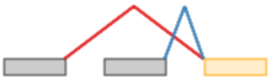	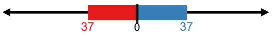
662	TOM1	ENSG00000100284:35718992-35719170:target	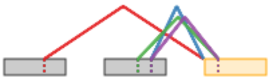	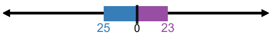
663	MTMR3	ENSG00000100330:30403890-30404021:target	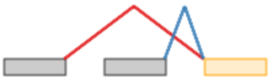	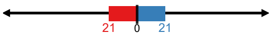
664	MTMR3	ENSG00000100330:30415469-30416873:source	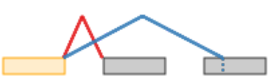	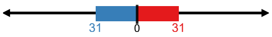
665	MTMR3	ENSG00000100330:30418452-30418686:target	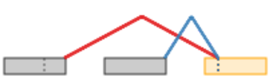	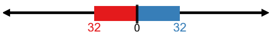
666	TNRC6B	ENSG00000100354:40642019-40642366:target	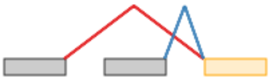	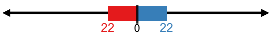
667	RANGAP1	ENSG00000100401:41650312-41650670:target	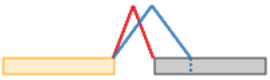	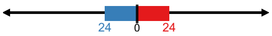
668	ZC3H7B	ENSG00000100403:41739419-41739580:source	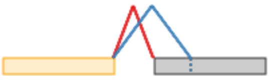	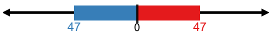
669	CERK	ENSG00000100422:47087469-47087674:source	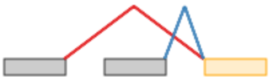	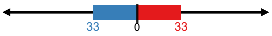
670	KHNYN	ENSG00000100441:24902142-24902238:source	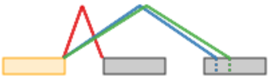	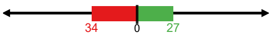
671	KHNYN	ENSG00000100441:24904663-24905385:target	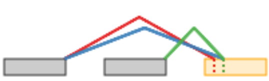	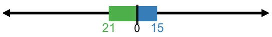
672	KHNYN	ENSG00000100441:24906242-24910540:target	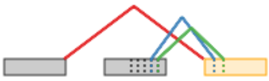	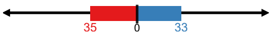
673	RBM23	ENSG00000100461:23375224-23375577:source	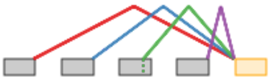	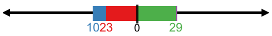
674	RBM23	ENSG00000100461:23378691-23378804:target	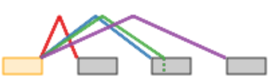	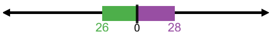
675	RBM23	ENSG00000100461:23388208-23388393:target	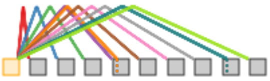	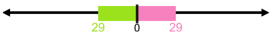
676	SOS2	ENSG00000100485:50622610-50623839:source	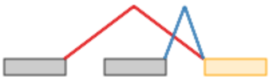	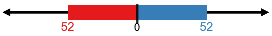
677	SOS2	ENSG00000100485:50626149-50626804:target	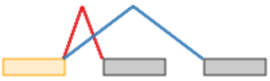	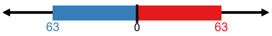
678	NIN	ENSG00000100503:51221460-51221585:source	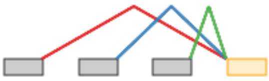	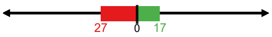
679	NIN	ENSG00000100503:51228508-51228690:target	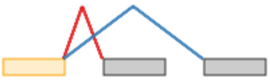	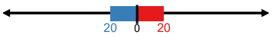

Gene LSV ID LSV Type ← More in Healthy | More in ALL →

#	Gene	LSV ID	LSV Type	← More in Healthy | More in ALL →
680	NIN	ENSG00000100503:51230544-51230682:target	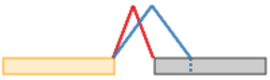	22 0 22
681	PSMC6	ENSG00000100519:53187828-53187908:source	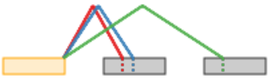	17 0 24
682	PSMC6	ENSG00000100519:53190667-53190754:target	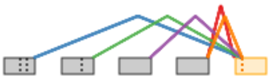	22 0 20
683	DDHD1	ENSG00000100523:53558503-53558650:source	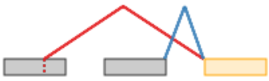	29 0 29
684	PSMA3	ENSG00000100567:58714468-58714550:source	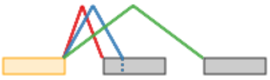	19 0 20
685	SPTLC2	ENSG00000100596:77987789-77987958:source	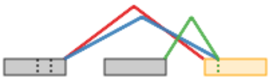	30 0 30
686	SPTLC2	ENSG00000100596:78018439-78018565:target	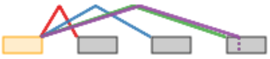	41 0 49
687	RIN3	ENSG00000100599:93125506-93125814:target	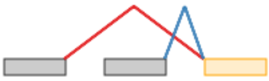	20 0 20
688	SNW1	ENSG00000100603:78187054-78187171:source	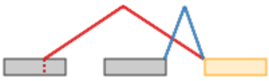	46 0 46
689	SNW1	ENSG00000100603:78197199-78197472:target	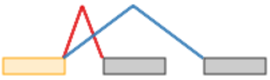	39 0 39
690	DICER1	ENSG00000100697:95599652-95599840:source	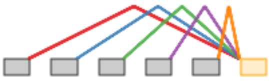	19 0 18
691	PCNX	ENSG00000100731:71514524-71514701:source	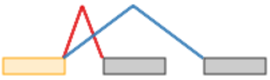	67 0 67
692	SMEK1	ENSG00000100796:91940985-91942310:target	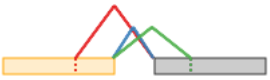	19 0 24
693	YY1	ENSG00000100811:100741035-100741095:source	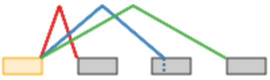	21 0 17
694	ACIN1	ENSG00000100813:23538242-23538826:source	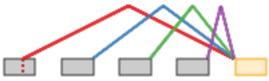	20 0 5 11
695	ACIN1	ENSG00000100813:23550957-23551045:source	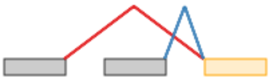	21 0 21
696	APEX1	ENSG00000100823:20923401-20923587:source	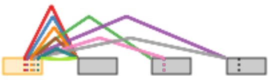	20 0 16
697	APEX1	ENSG00000100823:20923737-20923862:target	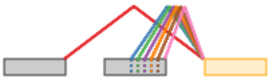	21 0 16
698	CHD8	ENSG00000100888:21895847-21896413:target	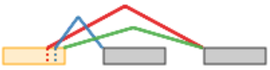	16 0 23
699	PSMA6	ENSG00000100902:35761555-35761806:source	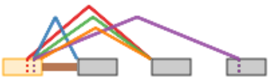	27 0 25
#	Gene	LSV ID	LSV Type	← More in Healthy | More in ALL →

#	Gene	LSV ID	LSV Type	← More in Healthy | More in ALL →
700	PSMA6	ENSG00000100902:35777200-35777642:target	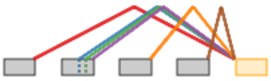	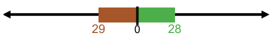 29 0 28
701	PSMA6	ENSG00000100902:35780039-35780258:source	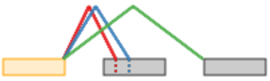	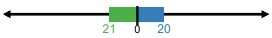 21 0 20
702	PCIF1	ENSG00000100982:44569692-44569846:source	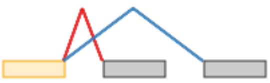	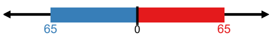 65 0 65
703	PCIF1	ENSG00000100982:44571736-44571883:source	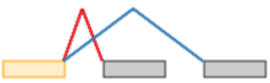	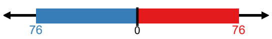 76 0 76
704	PCIF1	ENSG00000100982:44572305-44572404:target	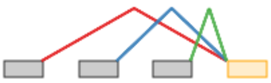	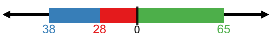 38 28 0 65
705	PCIF1	ENSG00000100982:44573507-44573669:source	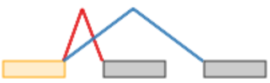	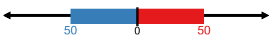 50 0 50
706	PCIF1	ENSG00000100982:44574668-44574754:target	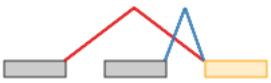	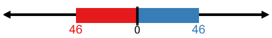 46 0 46
707	TRPC4AP	ENSG00000100991:33590207-33591086:source	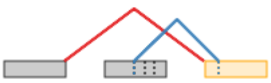	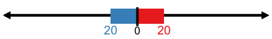 20 0 20
708	TRPC4AP	ENSG00000100991:33596467-33596550:target	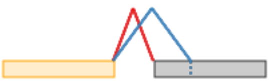	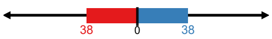 38 0 38
709	ZMYND8	ENSG00000101040:45837859-45839542:source	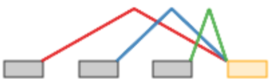	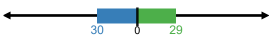 30 0 29
710	ZMYND8	ENSG00000101040:45848909-45848975:target	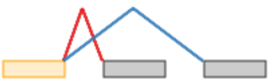	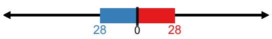 28 0 28
711	ZMYND8	ENSG00000101040:45865070-45865260:source	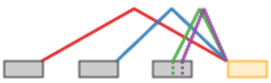	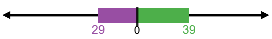 29 0 39
712	PABPC1L	ENSG00000101104:43572094-43572220:source	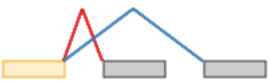	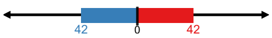 42 0 42
713	PABPC1L	ENSG00000101104:43580474-43580643:target	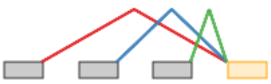	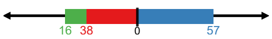 16 38 0 57
714	STK4	ENSG00000101109:43607084-43607212:target	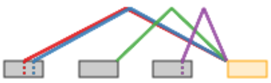	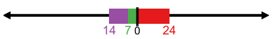 14 7 0 24
715	STK4	ENSG00000101109:43653614-43653771:source	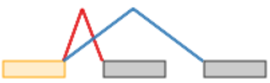	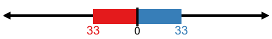 33 0 33
716	STK4	ENSG00000101109:43703659-43708600:target	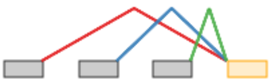	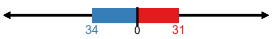 34 0 31
717	PSMA7	ENSG00000101182:60714752-60714961:target	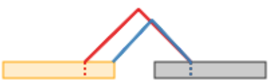	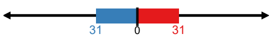 31 0 31
718	RASSF2	ENSG00000101265:4768279-4768400:source	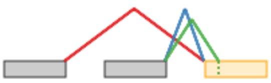	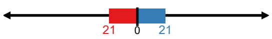 21 0 21
719	CDS2	ENSG00000101290:5154169-5154305:target	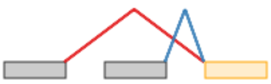	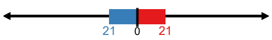 21 0 21
#	Gene	LSV ID	LSV Type	← More in Healthy | More in ALL →

#	Gene	LSV ID	LSV Type	← More in Healthy | More in ALL →
720	CDS2	ENSG00000101290:5159463-5159602:source	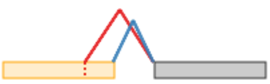	51 0 51
721	CDS2	ENSG00000101290:5169713-5169832:target	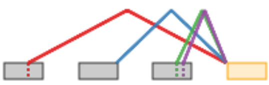	17 0 28
722	SEC23B	ENSG00000101310:18505579-18505664:source	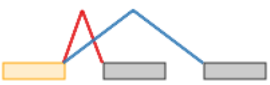	21 0 21
723	SEC23B	ENSG00000101310:18507017-18507175:target	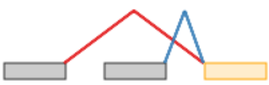	31 0 31
724	SEC23B	ENSG00000101310:18516297-18516386:source	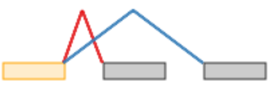	20 0 20
725	TM9SF4	ENSG00000101337:30730785-30730908:source	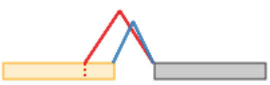	27 0 27
726	IDH3B	ENSG00000101365:2641343-2641475:target	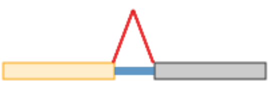	23 0 23
727	USP14	ENSG00000101557:180236-180882:source	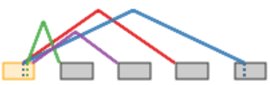	15 0 23
728	SMCHD1	ENSG00000101596:2666869-2667030:source	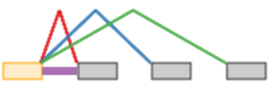	48 0 49
729	SMCHD1	ENSG00000101596:2673280-2673362:target	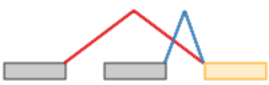	47 0 47
730	SMCHD1	ENSG00000101596:2718313-2718635:source	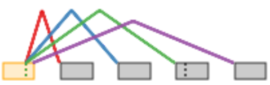	30 0 34
731	SMCHD1	ENSG00000101596:2724897-2724993:target	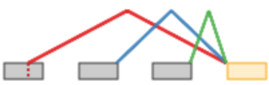	24 0 25
732	CEP192	ENSG00000101639:13117584-13117642:source	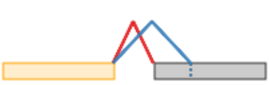	55 0 55
733	ANKRD12	ENSG00000101745:9195549-9195696:source	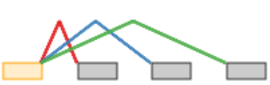	23 0 27
734	ANKRD12	ENSG00000101745:9216756-9216898:source	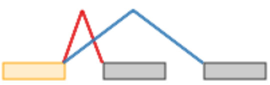	26 0 26
735	ANKRD12	ENSG00000101745:9221850-9221997:target	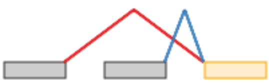	25 0 25
736	KIAA0226L	ENSG00000102445:46933621-46933766:source	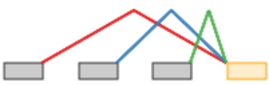	15 0 20
737	KIAA0226L	ENSG00000102445:46935576-46935708:source	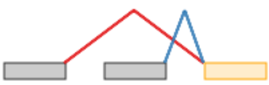	24 0 24
738	KIAA0226L	ENSG00000102445:46937250-46937348:target	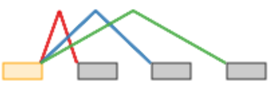	15 12 0 29
739	KIAA0226L	ENSG00000102445:46942173-46942384:source	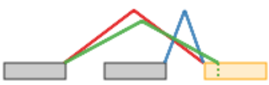	31 0 27
#	Gene	LSV ID	LSV Type	← More in Healthy | More in ALL →

#	Gene	LSV ID	LSV Type	← More in Healthy | More in ALL →
740	KIAA0226L	ENSG00000102445:46942868-46942950:target		30 0 30
741	KIAA0226L	ENSG00000102445:46946076-46946732:source		14 7 5 0 11 23
742	KIAA0226L	ENSG00000102445:46960453-46960860:target		16 27 11 0 53
743	KIAA0226L	ENSG00000102445:46960861-46962101:target		12 16 0 27
744	FNDC3A	ENSG00000102531:49688791-49688867:target		24 0 24
745	FNDC3A	ENSG00000102531:49752704-49752790:target		56 0 56
746	FNDC3A	ENSG00000102531:49765345-49765520:source		25 0 25
747	FNDC3A	ENSG00000102531:49772498-49772710:source		36 0 36
748	STK24	ENSG00000102572:99115945-99116090:source		44 0 44
749	STK24	ENSG00000102572:99118630-99118815:target		21 0 21
750	ARHGEF7	ENSG00000102606:111935175-111935746:source		18 11 0 34
751	ARHGEF7	ENSG00000102606:111938494-111938587:target		19 6 13 0 45
752	PARP4	ENSG00000102699:25005515-25005614:source		43 0 43
753	PARP4	ENSG00000102699:25008533-25009612:target		47 0 47
754	RGCC	ENSG00000102760:42032421-42032606:source		24 0 22
755	PHKB	ENSG00000102893:47545576-47545683:source		21 0 21
756	PHKB	ENSG00000102893:47549432-47549512:target		33 0 33
757	PHKB	ENSG00000102893:47614206-47614269:source		40 0 40
758	PHKB	ENSG00000102893:47621579-47621674:target		20 0 20
759	PHKB	ENSG00000102893:47682444-47682527:source		21 0 21

| # | Gene | LSV ID | LSV Type | ← More in Healthy \| More in ALL → |

#	Gene	LSV ID	LSV Type	← More in Healthy | More in ALL →
760	PHKB	ENSG00000102893:47684455-47684537:target		20 0 20
761	PHKB	ENSG00000102893:47730292-47730399:source		31 0 31
762	NFAT5	ENSG00000102908:69680931-69681489:target		49 0 49
763	LONP2	ENSG00000102910:48286042-48286276:source		24 0 24
764	ZNF821	ENSG00000102984:71893583-71894575:source		28 0 28
765	ZNF821	ENSG00000102984:71898041-71898145:target		27 0 27
766	SLC7A6	ENSG00000103064:68300494-68300624:source		22 0 22
767	CMC2	ENSG00000103121:81009698-81010076:source		18 0 17
768	CMC2	ENSG00000103121:81030919-81031212:target		27 0 23
769	ABCC1	ENSG00000103222:16219667-16219768:target		22 0 22
770	CLCN7	ENSG00000103249:1509108-1509413:source		8 16 0 26
771	CLCN7	ENSG00000103249:1509930-1510528:target		12 14 0 27
772	CLCN7	ENSG00000103249:1515268-1515339:source		49 0 49
773	NUBP1	ENSG00000103274:10846467-10846535:source		24 0 25
774	NUBP1	ENSG00000103274:10850547-10850637:target		25 0 25
775	PIEZO1	ENSG00000103335:88783431-88783619:source		21 0 23
776	PIEZO1	ENSG00000103335:88785380-88786129:target		25 0 25
777	GGA2	ENSG00000103365:23490715-23491208:target		10 0 20
778	HERC1	ENSG00000103657:63915014-63915090:source		36 0 36
779	HERC1	ENSG00000103657:63916357-63916526:target		44 0 44

Gene LSV ID LSV Type ← More in Healthy | More in ALL →

#	Gene	LSV ID	LSV Type	← More in Healthy | More in ALL →
780	HERC1	ENSG00000103657:63955183-63955403:target	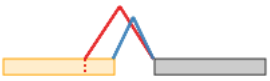	29 0 29
781	HERC1	ENSG00000103657:63956669-63956793:target	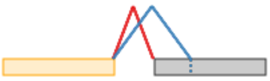	29 0 29
782	HERC1	ENSG00000103657:63998979-63999030:source	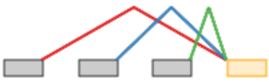	21 7 0 31
783	HERC1	ENSG00000103657:64004974-64005094:source	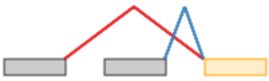	23 0 23
784	HERC1	ENSG00000103657:64015381-64015554:source	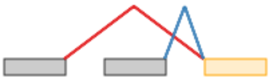	26 0 26
785	HERC1	ENSG00000103657:64019846-64020037:target	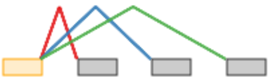	32 0 37
786	GABPB1	ENSG00000104064:50569389-50570981:source	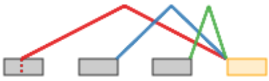	29 0 30
787	GABPB1	ENSG00000104064:50593310-50593565:target	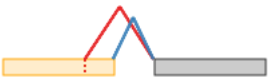	20 0 20
788	BMF	ENSG00000104081:40380091-40384089:source	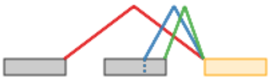	27 0 26
789	BMF	ENSG00000104081:40397996-40398294:target	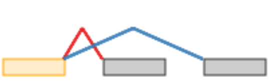	23 0 23
790	SPG11	ENSG00000104133:44858415-44858503:target	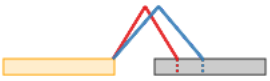	35 0 35
791	SPG11	ENSG00000104133:44892458-44892830:source	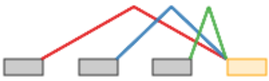	30 0 28
792	SPG11	ENSG00000104133:44900642-44900803:target	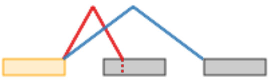	22 0 22
793	SPG11	ENSG00000104133:44903038-44903183:source	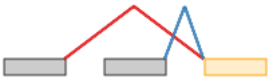	21 0 21
794	SPG11	ENSG00000104133:44907561-44907764:target	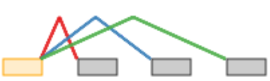	11 23 0 37
795	BLOC1S6	ENSG00000104164:45890078-45891760:target	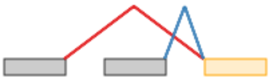	23 0 23
796	IKBKB	ENSG00000104365:42174228-42174742:source	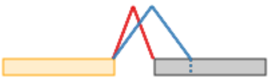	25 0 25
797	UBR5	ENSG00000104517:103277328-103277506:source	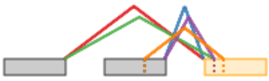	10 7 0 18
798	UBR5	ENSG00000104517:103281206-103281336:target	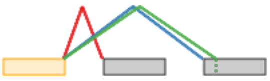	9 14 0 25
799	UBR5	ENSG00000104517:103282267-103282411:source	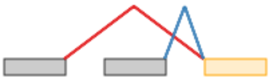	32 0 32

Gene LSV ID LSV Type ← More in Healthy | More in ALL →

#	Gene	LSV ID	LSV Type	← More in Healthy | More in ALL →
800	UBR5	ENSG00000104517:103284757-103285159:target	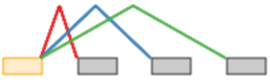	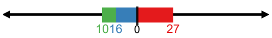 10 16 0 27
801	UBR5	ENSG00000104517:103289200-103289420:source	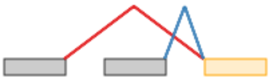	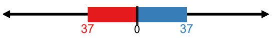 37 0 37
802	UBR5	ENSG00000104517:103291264-103291436:target	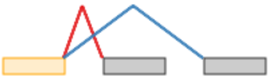	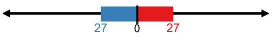 27 0 27
803	UBR5	ENSG00000104517:103307864-103308010:source	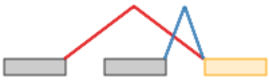	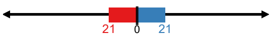 21 0 21
804	UBR5	ENSG00000104517:103309686-103309834:target	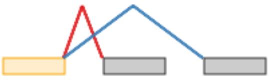	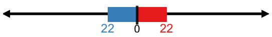 22 0 22
805	UBR5	ENSG00000104517:103335539-103335723:target	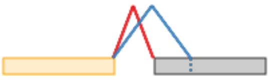	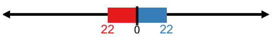 22 0 22
806	LEPROTL1	ENSG00000104660:30032601-30032706:target	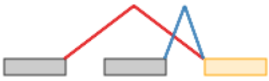	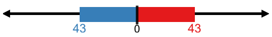 43 0 43
807	DCTN6	ENSG00000104671:30032601-30032706:target	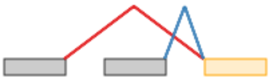	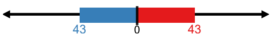 43 0 43
808	MAN2B1	ENSG00000104774:12769038-12769158:source	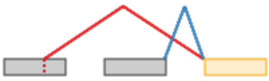	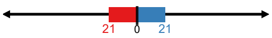 21 0 21
809	MAP4K1	ENSG00000104814:39106818-39106899:target	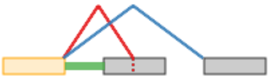	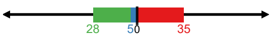 28 5 0 35
810	HNRNPL	ENSG00000104824:39334011-39334554:source	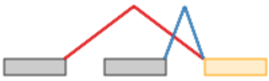	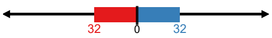 32 0 32
811	HNRNPL	ENSG00000104824:39336493-39336730:target	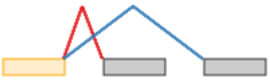	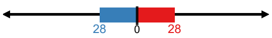 28 0 28
812	HNRNPL	ENSG00000104824:39337956-39338272:source	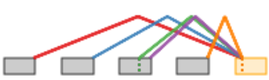	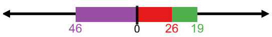 46 0 26 19
813	CLASRP	ENSG00000104859:45571273-45571438:source	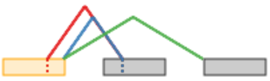	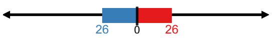 26 0 26
814	PIH1D1	ENSG00000104872:49950281-49950434:target	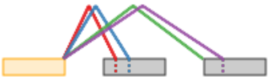	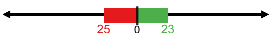 25 0 23
815	PIH1D1	ENSG00000104872:49954625-49955158:target	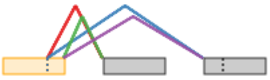	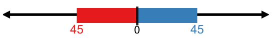 45 0 45
816	OAZ1	ENSG00000104904:2269485-2269743:source	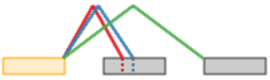	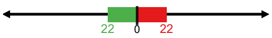 22 0 22
817	OAZ1	ENSG00000104904:2271781-2272261:source	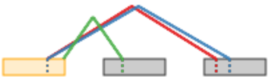	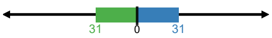 31 0 31
818	OAZ1	ENSG00000104904:2271781-2272261:target	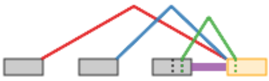	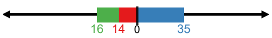 16 14 0 35
819	PTOV1	ENSG00000104960:50361806-50362120:target	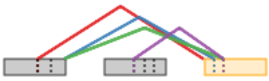	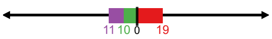 11 10 0 19

Gene LSV ID LSV Type ← More in Healthy | More in ALL →

#	Gene	LSV ID	LSV Type	← More in Healthy | More in ALL →
820	VRK3	ENSG00000105053:50510826-50511083:source	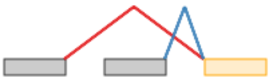	55 0 55
821	VRK3	ENSG00000105053:50519281-50519420:target	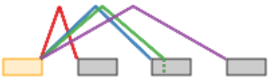	48 0 45
822	PPP6R1	ENSG00000105063:55758245-55758740:source	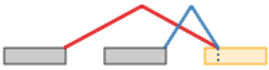	22 0 22
823	HNRNPUL1	ENSG00000105323:41773983-41774250:target	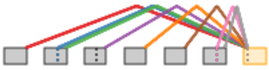	20 0 6
824	TGFB1	ENSG00000105329:41808529-41808854:source	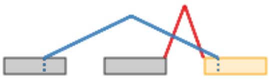	25 0 25
825	DENND3	ENSG00000105339:142148121-142148236:source	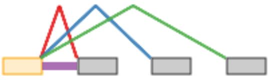	16 43 0 63
826	DENND3	ENSG00000105339:142160933-142161052:target	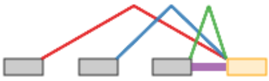	42 0 49
827	DENND3	ENSG00000105339:142165948-142166069:source	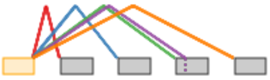	7 7 0 30
828	DENND3	ENSG00000105339:142170731-142170886:source	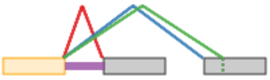	9 15 0 26
829	DENND3	ENSG00000105339:142173432-142173528:target	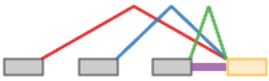	20 16 0 41
830	DENND3	ENSG00000105339:142175285-142175388:target	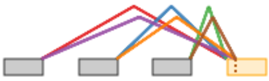	10 0 31
831	DENND3	ENSG00000105339:142187186-142188295:source	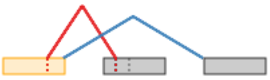	20 0 20
832	DENND3	ENSG00000105339:142187186-142188295:target	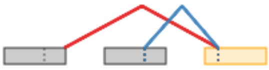	57 0 57
833	NAPA	ENSG00000105402:48003890-48004006:source	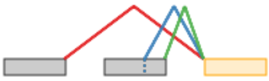	21 0 20
834	NAPA	ENSG00000105402:48003890-48004006:target	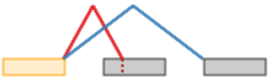	29 0 29
835	CARD8	ENSG00000105483:48744219-48744320:source	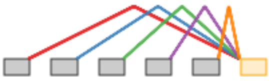	28 0 25
836	CARD8	ENSG00000105483:48753008-48753135:target	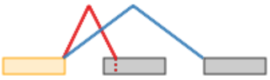	20 0 20
837	LIG1	ENSG00000105486:48623424-48624579:target	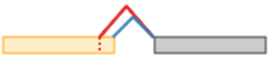	45 0 45
838	ARRDC2	ENSG00000105643:18119520-18119670:target	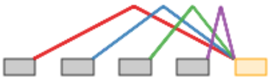	28 0 28
839	ARRDC2	ENSG00000105643:18119780-18119927:source	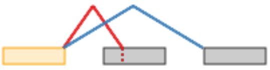	20 0 20

| # | Gene | LSV ID | LSV Type | ← More in Healthy \| More in ALL → |

#	Gene	LSV ID	LSV Type	← More in Healthy | More in ALL →
840	COPE	ENSG00000105669:19016385-19016438:source		30 0 31
841	ATP13A1	ENSG00000105726:19763360-19764205:target		28 0 28
842	SIPA1L3	ENSG00000105738:38610289-38610522:source		28 0 28
843	SIPA1L3	ENSG00000105738:38631824-38632075:target		33 0 33
844	SIPA1L3	ENSG00000105738:38682785-38682950:target		30 0 30
845	AVL9	ENSG00000105778:32919047-32919554:source		23 0 23
846	PMPCB	ENSG00000105819:102949399-102949706:target		29 0 29
847	PMPCB	ENSG00000105819:102966990-102967131:source		22 0 22
848	DNAJC2	ENSG00000105821:102966990-102967131:source		22 0 22
849	TWISTNB	ENSG00000105849:19735085-19738350:source		22 0 22
850	PIK3CG	ENSG00000105851:106507995-106510001:target		26 0 21
851	PIK3CG	ENSG00000105851:106522562-106522652:source		46 0 46
852	HBP1	ENSG00000105856:106830321-106830762:target		28 0 29
853	CBLL1	ENSG00000105879:107389325-107389761:source		14 0 22
854	CASP2	ENSG00000106144:142988633-142988783:target		40 0 40
855	BUD31	ENSG00000106245:99007657-99007792:target		28 0 27
856	EZH2	ENSG00000106462:148508717-148508915:source		33 0 32
857	EZH2	ENSG00000106462:148512598-148512638:source		47 0 52
858	EZH2	ENSG00000106462:148512598-148512638:target		34 0 31
859	EZH2	ENSG00000106462:148514969-148515209:source		20 0 20

Gene LSV ID LSV Type ← More in Healthy | More in ALL →

#	Gene	LSV ID	LSV Type	← More in Healthy | More in ALL →
860	EZH2	ENSG00000106462:148514969-148515209:target	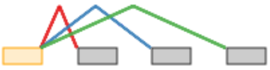	49 0 3910
861	EZH2	ENSG00000106462:148526820-148526940:source	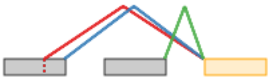	26 0 28
862	TSPAN13	ENSG00000106537:16818628-16818741:source	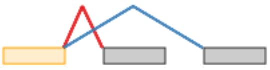	26 0 26
863	COA1	ENSG00000106603:43687787-43689895:target	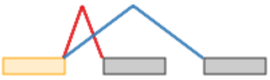	23 0 23
864	RHEB	ENSG00000106615:151181823-151181890:target	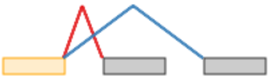	25 0 25
865	PRKAG2	ENSG00000106617:151257461-151257703:target	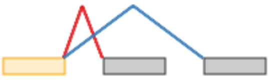	91 0 91
866	EIF4H	ENSG00000106682:73604152-73604303:source	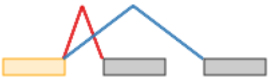	26 0 26
867	EIF4H	ENSG00000106682:73609071-73609208:target	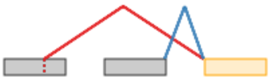	29 0 29
868	SEC61B	ENSG00000106803:101984828-101985462:source	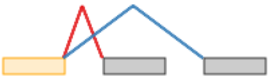	44 0 44
869	SEC61B	ENSG00000106803:101990181-101990282:target	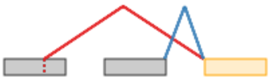	52 0 52
870	TLE4	ENSG00000106829:82242289-82242667:source	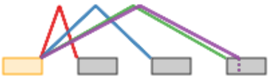	62 0 16 46
871	TLE4	ENSG00000106829:82267505-82267544:target	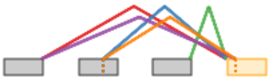	62 0 15 40
872	AKNA	ENSG00000106948:117104289-117104405:source	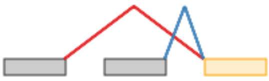	29 0 29
873	AKNA	ENSG00000106948:117108143-117108289:target	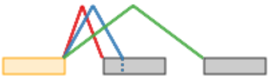	34 0 40
874	AKNA	ENSG00000106948:117129823-117130341:source	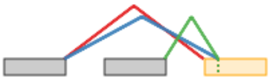	35 0 37
875	AKNA	ENSG00000106948:117130719-117130875:target	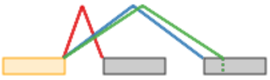	35 0 35
876	AKNA	ENSG00000106948:117143340-117143726:source	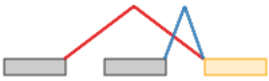	22 0 22
877	RIC1	ENSG00000107036:5732388-5732479:source	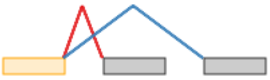	58 0 58
878	RIC1	ENSG00000107036:5742869-5743013:target	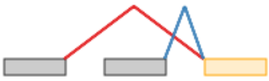	61 0 61
879	RIC1	ENSG00000107036:5743689-5743737:source	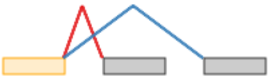	26 0 26

Gene LSV ID LSV Type ← More in Healthy | More in ALL →

#	Gene	LSV ID	LSV Type	← More in Healthy | More in ALL →
880	RIC1	ENSG00000107036:5753200-5753238:source		35 0 35
881	RIC1	ENSG00000107036:5754841-5754930:target		31 0 31
882	KDM4C	ENSG00000107077:7046862-7046917:target		12 9 0 20
883	KDM4C	ENSG00000107077:7049092-7049200:source		43 0 43
884	KDM4C	ENSG00000107077:7103685-7104017:target		38 0 42
885	DOCK8	ENSG00000107099:432166-432324:source		30 0 30
886	DOCK8	ENSG00000107099:434783-434975:source		15 12 0 29
887	DOCK8	ENSG00000107099:434783-434975:target		28 0 28
888	DOCK8	ENSG00000107099:441875-442009:source		55 0 60
889	DOCK8	ENSG00000107099:446370-446606:target		715 0 32
890	BAG1	ENSG00000107262:33261085-33261167:source		61 0 61
891	BAG1	ENSG00000107262:33262700-33262828:target		66 0 66
892	RAPGEF1	ENSG00000107263:134473586-134473697:source		1016 0 25
893	RAPGEF1	ENSG00000107263:134477448-134477536:target		20 0 20
894	RAPGEF1	ENSG00000107263:134504489-134504641:source		21 0 24
895	RAPGEF1	ENSG00000107263:134513896-134514178:target		27 0 32
896	RAPGEF1	ENSG00000107263:134526197-134526336:source		31 0 37
897	RAPGEF1	ENSG00000107263:134526197-134526336:target		29 0 29
898	SETX	ENSG00000107290:135201711-135205886:target		33 0 33
899	SETX	ENSG00000107290:135221648-135221858:target		33 0 33

\# Gene LSV ID LSV Type ← More in Healthy | More in ALL →

#	Gene	LSV ID	LSV Type	← More in Healthy | More in ALL →
900	ZFAND5	ENSG00000107372:74975220-74975703:source		37 0 38
901	ZFAND5	ENSG00000107372:74979612-74980163:target		18 0 20
902	DDX50	ENSG00000107625:70666467-70666763:source		35 0 35
903	DDX50	ENSG00000107625:70670063-70670138:target		34 0 34
904	NSMCE4A	ENSG00000107672:123719433-123719921:source		28 0 28
905	NSMCE4A	ENSG00000107672:123719433-123719921:target		14 36 0 53
906	TNKS2	ENSG00000107854:93593610-93593781:source		22 0 22
907	GBF1	ENSG00000107862:104121475-104121669:target		32 0 32
908	GBF1	ENSG00000107862:104128968-104129144:target		25 0 29
909	GBF1	ENSG00000107862:104129468-104129532:source		50 0 50
910	GBF1	ENSG00000107862:104129931-104130002:target		53 0 53
911	SH3PXD2A	ENSG00000107957:105376954-105377071:source		20 0 20
912	XPNPEP1	ENSG00000108039:111667449-111667577:target		18 0 21
913	SHOC2	ENSG00000108061:112723883-112724487:target		34 0 38
914	CUL2	ENSG00000108094:35338611-35338693:source		20 0 20
915	CUL2	ENSG00000108094:35360127-35360267:source		24 0 1210
916	PPIF	ENSG00000108179:81113463-81115093:target		23 0 23
917	ENSG00000108294	ENSG00000108294:36918304-36918758:target		24 0 24
918	ENSG00000108296	ENSG00000108296:36965955-36966061:source		27 0 27
919	RPL19	ENSG00000108298:37356536-37356996:source		66 0 66

Gene LSV ID LSV Type ← More in Healthy | More in ALL →

#	Gene	LSV ID	LSV Type	← More in Healthy | More in ALL →
920	CASC3	ENSG00000108349:38323050-38323333:target		22 0 22
921	DHX40	ENSG00000108406:57642886-57643161:source		41 0 41
922	DHX40	ENSG00000108406:57663514-57663594:target		536 0 43
923	KPNB1	ENSG00000108424:45745552-45745776:source		55 0 55
924	KPNB1	ENSG00000108424:45748068-45748198:target		57 0 57
925	KPNB1	ENSG00000108424:45752004-45752148:target		22 0 22
926	RPS6KB1	ENSG00000108443:57987923-57987972:target		20 0 19
927	RPS6KB1	ENSG00000108443:58013825-58013902:source		41 0 41
928	MED13	ENSG00000108510:60043823-60044012:source		26 0 26
929	NUP88	ENSG00000108559:5264258-5265381:source		37 11 0 60
930	NUP88	ENSG00000108559:5302872-5302970:source		31 0 31
931	NUP88	ENSG00000108559:5308377-5308563:source		19 0 26
932	NUP88	ENSG00000108559:5308377-5308563:target		37 0 42
933	NUP88	ENSG00000108559:5312053-5312229:target		15 16 0 33
934	GOSR1	ENSG00000108587:28811681-28812152:source		53 0 53
935	GOSR1	ENSG00000108587:28819697-28819784:target		56 0 56
936	AKAP10	ENSG00000108599:19843026-19843162:source		27 0 27
937	AKAP10	ENSG00000108599:19845139-19845223:target		30 0 30
938	CYTH1	ENSG00000108669:76698204-76698322:source		45 0 45
939	CYTH1	ENSG00000108669:76704279-76704343:target		36 0 36

Gene LSV ID LSV Type ← More in Healthy | More in ALL →

#	Gene	LSV ID	LSV Type	← More in Healthy | More in ALL →
940	CYTH1	ENSG00000108669:76704994-76705814:source		24 0 21
941	PSMD11	ENSG00000108671:30781513-30781637:target		27 0 27
942	KAT2A	ENSG00000108773:40271072-40271454:target		27 0 27
943	PPP1R9B	ENSG00000108819:48218634-48218738:target		32 25 0 64
944	HDAC5	ENSG00000108840:42156512-42156704:source		39 0 39
945	HDAC5	ENSG00000108840:42157769-42157866:target		37 0 37
946	HDAC5	ENSG00000108840:42161923-42162043:source		56 0 56
947	HDAC5	ENSG00000108840:42163940-42164123:target		49 0 49
948	HDAC5	ENSG00000108840:42169068-42169199:source		25 0 25
949	LUC7L3	ENSG00000108848:48796905-48797192:source		21 0 21
950	LUC7L3	ENSG00000108848:48814648-48814840:source		20 0 22
951	LUC7L3	ENSG00000108848:48817628-48817706:target		24 0 31
952	LUC7L3	ENSG00000108848:48827862-48828055:source		17 0 20
953	LUC7L3	ENSG00000108848:48828085-48828374:source		50 6 0 58
954	LUC7L3	ENSG00000108848:48829561-48833574:target		7 55 0 49 13
955	PRKAR1A	ENSG00000108946:66511535-66511956:target		19 0 23
956	YWHAE	ENSG00000108953:1264386-1264592:source		53 12 0 65
957	YWHAE	ENSG00000108953:1264965-1265302:target		66 0 66
958	WSB1	ENSG00000109046:25621102-25621461:source		21 0 19
959	WSB1	ENSG00000109046:25629461-25630775:target		24 0 24

Gene LSV ID LSV Type ← More in Healthy | More in ALL →

#	Gene	LSV ID	LSV Type	← More in Healthy | More in ALL →
960	WSB1	ENSG00000109046:25636126-25636298:source	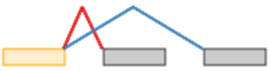	50 0 50
961	WSB1	ENSG00000109046:25638535-25638642:target	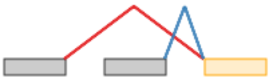	62 0 62
962	UNC119	ENSG00000109103:26874542-26874867:source	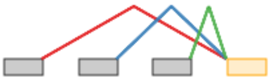	10 0 22
963	UNC119	ENSG00000109103:26879356-26879686:target	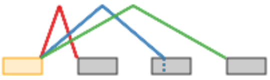	18 0 20
964	SUPT6H	ENSG00000109111:27002419-27002503:source	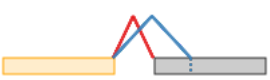	23 0 23
965	PHF12	ENSG00000109118:27236588-27237387:target	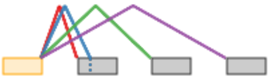	14 0 27
966	OCIAD1	ENSG00000109180:48834637-48834778:target	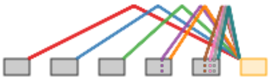	21 0 18
967	OCIAD1	ENSG00000109180:48857989-48859382:target	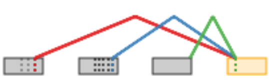	28 9 0 40
968	CHIC2	ENSG00000109220:54914998-54915277:target	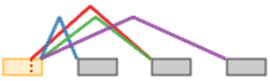	23 0 22
969	NFKB1	ENSG00000109320:103450993-103451071:source	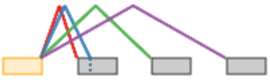	15 5 0 23
970	NFKB1	ENSG00000109320:103488144-103488292:target	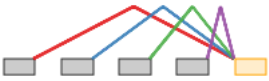	5 16 0 24
971	NFKB1	ENSG00000109320:103501692-103501796:target	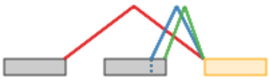	18 0 21
972	MANBA	ENSG00000109323:103555945-103556202:source	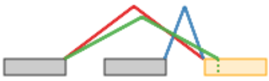	7 55 0 63
973	MANBA	ENSG00000109323:103560870-103561014:target	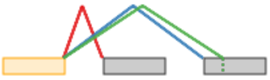	6 49 0 56
974	MANBA	ENSG00000109323:103590120-103590206:source	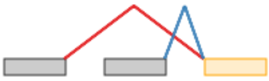	62 0 62
975	MANBA	ENSG00000109323:103592438-103592555:target	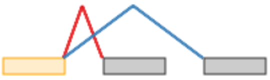	55 0 55
976	MANBA	ENSG00000109323:103643530-103644198:target	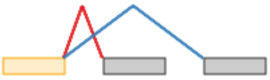	26 0 26
977	TBC1D9	ENSG00000109436:141580571-141580859:source	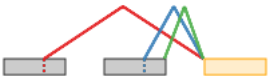	12 16 0 31
978	TBC1D9	ENSG00000109436:141582782-141583357:target	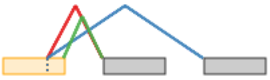	42 0 42
979	ZNF330	ENSG00000109445:142142041-142142262:source	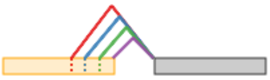	30 0 26
#	Gene	LSV ID	LSV Type	← More in Healthy | More in ALL →

#	Gene	LSV ID	LSV Type	← More in Healthy | More in ALL →
980	KLHL2	ENSG00000109466:166232616-166232717:source	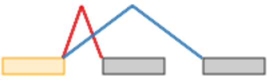	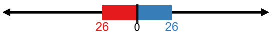
981	KLHL2	ENSG00000109466:166234390-166234518:target	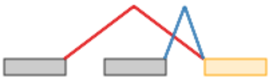	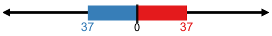
982	FRG1	ENSG00000109536:190882977-190883087:target	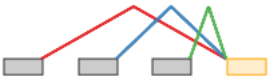	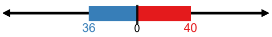
983	CLCN3	ENSG00000109572:170618512-170618885:source	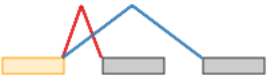	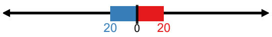
984	DHX15	ENSG00000109606:24577866-24578301:source	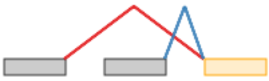	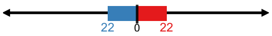
985	FBXW7	ENSG00000109670:153271194-153271276:source	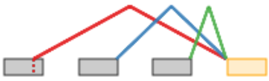	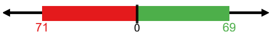
986	STIM2	ENSG00000109689:26921126-26921256:target	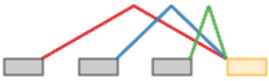	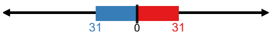
987	STIM2	ENSG00000109689:27024141-27027003:target	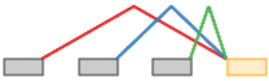	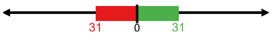
988	MFSD10	ENSG00000109736:2934327-2934485:target	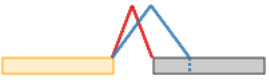	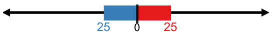
989	SNX25	ENSG00000109762:186253749-186253913:source	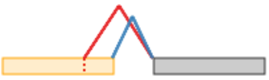	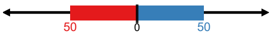
990	SNX25	ENSG00000109762:186267691-186267804:source	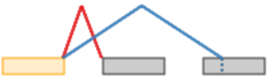	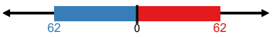
991	SNX25	ENSG00000109762:186274212-186274756:target	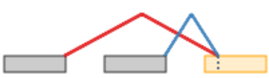	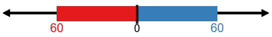
992	SNX25	ENSG00000109762:186278825-186278891:target	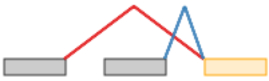	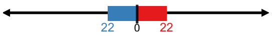
993	SNX25	ENSG00000109762:186283078-186283259:source	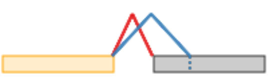	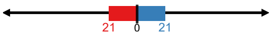
994	FNBP4	ENSG00000109920:47752836-47753114:target	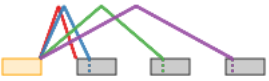	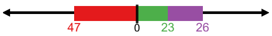
995	FNBP4	ENSG00000109920:47753669-47753949:target	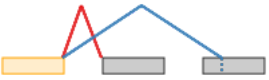	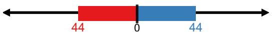
996	HSPA8	ENSG00000109971:122930824-122931134:source	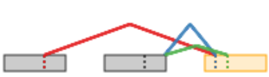	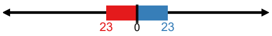
997	HSPA8	ENSG00000109971:122931533-122932040:target	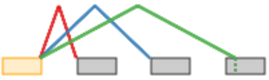	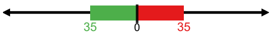
998	OSBP	ENSG00000110048:59348924-59349027:source	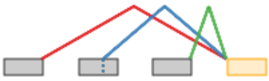	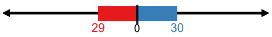
999	PPP6R3	ENSG00000110075:68272613-68272739:source	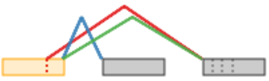	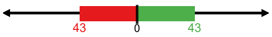

Gene LSV ID LSV Type ← More in Healthy | More in ALL →

#	Gene	LSV ID	LSV Type	← More in Healthy | More in ALL →
1000	PPP6R3	ENSG00000110075:68286969-68287119:source	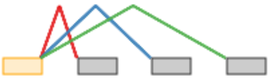	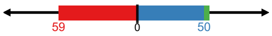
1001	PPP6R3	ENSG00000110075:68286969-68287119:target	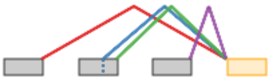	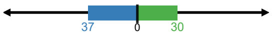
1002	PPP6R3	ENSG00000110075:68305127-68305249:target	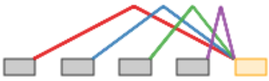	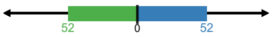
1003	PPP6R3	ENSG00000110075:68315535-68315672:source	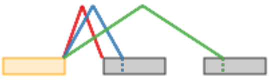	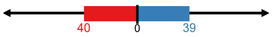
1004	PPP6R3	ENSG00000110075:68331892-68331900:source	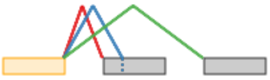	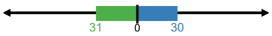
1005	PPP6R3	ENSG00000110075:68337216-68337365:target	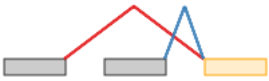	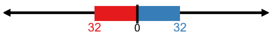
1006	HIPK3	ENSG00000110422:33369398-33369559:source	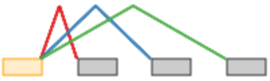	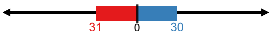
1007	FBXO3	ENSG00000110429:33790397-33790560:target	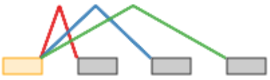	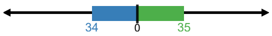
1008	MADD	ENSG00000110514:47312161-47312367:target	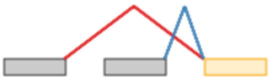	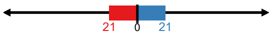
1009	SOX6	ENSG00000110693:16769644-16769704:target	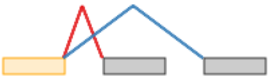	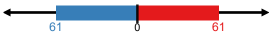
1010	NUP98	ENSG00000110713:3697739-3697850:source	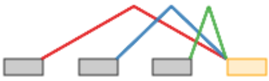	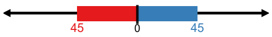
1011	NUP98	ENSG00000110713:3704430-3704671:target	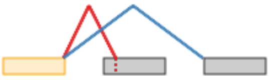	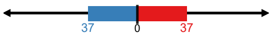
1012	NUP98	ENSG00000110713:3726430-3726586:target	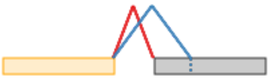	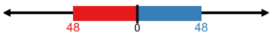
1013	NUP98	ENSG00000110713:3735048-3735225:target	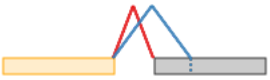	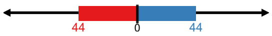
1014	PRDM4	ENSG00000110851:108135998-108136370:source	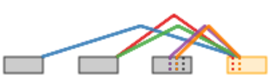	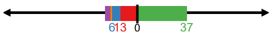
1015	PRDM4	ENSG00000110851:108136974-108137059:source	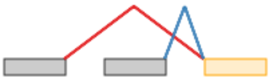	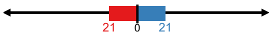
1016	PRDM4	ENSG00000110851:108140052-108140201:target	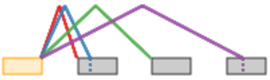	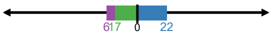
1017	RSRC2	ENSG00000111011:122989190-122990253:source	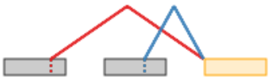	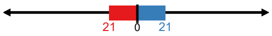
1018	RSRC2	ENSG00000111011:122995656-122995735:source	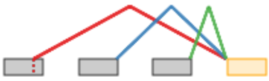	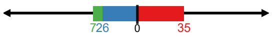
1019	RSRC2	ENSG00000111011:122999320-122999774:target	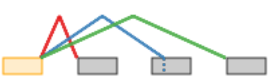	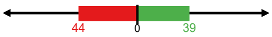
#	Gene	LSV ID	LSV Type	← More in Healthy | More in ALL →

#	Gene	LSV ID	LSV Type	← More in Healthy | More in ALL →
1020	RSRC2	ENSG00000111011:123006549-123006989:source	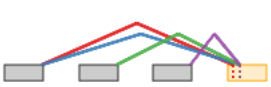	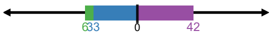 6 33 0 42
1021	RSRC2	ENSG00000111011:123011395-123011547:target	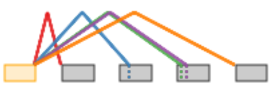	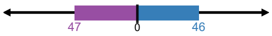 47 0 46
1022	LTA4H	ENSG00000111144:96406966-96407036:target	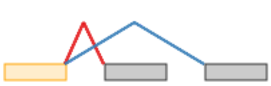	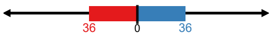 36 0 36
1023	ARPC3	ENSG00000111229:110873915-110874009:source	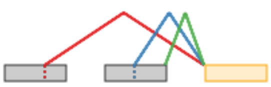	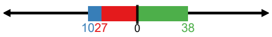 10 27 0 38
1024	ARPC3	ENSG00000111229:110874540-110875104:target	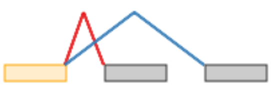	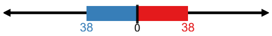 38 0 38
1025	NAA25	ENSG00000111300:112516438-112516545:target	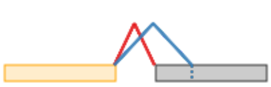	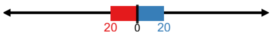 20 0 20
1026	CNOT2	ENSG00000111596:70712804-70713144:source	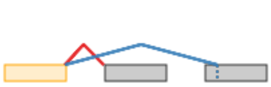	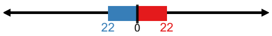 22 0 22
1027	CNOT2	ENSG00000111596:70728215-70729613:target	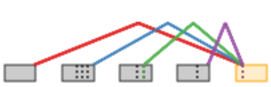	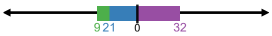 9 21 0 32
1028	CHD4	ENSG00000111642:6690815-6690980:source	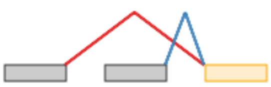	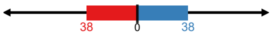 38 0 38
1029	CHD4	ENSG00000111642:6691781-6691914:target	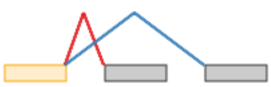	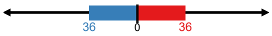 36 0 36
1030	CHD4	ENSG00000111642:6696878-6697115:source	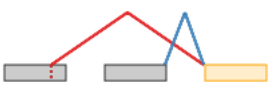	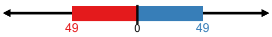 49 0 49
1031	CHD4	ENSG00000111642:6700406-6700751:target	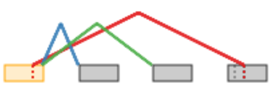	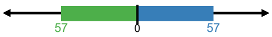 57 0 57
1032	CHD4	ENSG00000111642:6709700-6709835:source	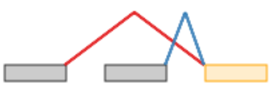	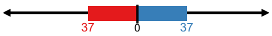 37 0 37
1033	CHD4	ENSG00000111642:6709700-6709835:target	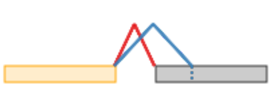	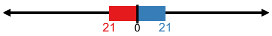 21 0 21
1034	CHD4	ENSG00000111642:6710455-6710696:target	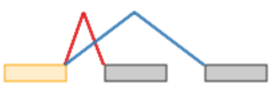	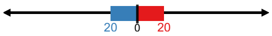 20 0 20
1035	CHPT1	ENSG00000111666:102120072-102120805:source	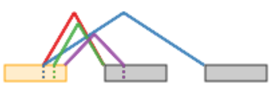	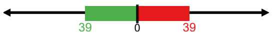 39 0 39
1036	GNPTAB	ENSG00000111670:102164774-102164935:target	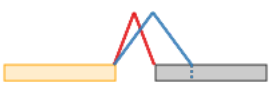	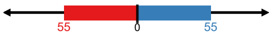 55 0 55
1037	C2CD5	ENSG00000111731:22624901-22624999:source	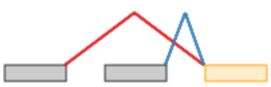	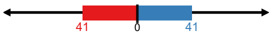 41 0 41
1038	C2CD5	ENSG00000111731:22625678-22625741:target	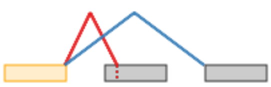	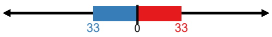 33 0 33
1039	PHC1	ENSG00000111752:9087720-9087834:source	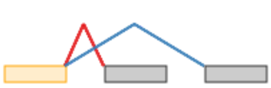	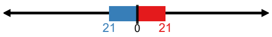 21 0 21
#	Gene	LSV ID	LSV Type	← More in Healthy | More in ALL →

#	Gene	LSV ID	LSV Type	← More in Healthy | More in ALL →
1040	RNGTT	ENSG00000111880:89323991-89324114:source		47 27 0 71
1041	FAM65B	ENSG00000111913:24832484-24832619:source		31 0 31
1042	FAM65B	ENSG00000111913:24835931-24836278:source		24 0 24
1043	FAM65B	ENSG00000111913:24843090-24843782:target		7 9 0 13 8
1044	FAM65B	ENSG00000111913:24852803-24852846:source		34 0 34
1045	FAM65B	ENSG00000111913:24861201-24861264:target		29 0 29
1046	FAM65B	ENSG00000111913:24875919-24876045:source		46 0 9 35
1047	KCTD20	ENSG00000112078:36437829-36438034:source		42 0 30 9
1048	KCTD20	ENSG00000112078:36447368-36447488:target		14 49 0 65
1049	KCTD20	ENSG00000112078:36449339-36449615:source		33 0 33
1050	SOD2	ENSG00000112096:160148161-160148735:source		25 0 5 12
1051	SOD2	ENSG00000112096:160157290-160157327:target		26 0 18
1052	SOD2	ENSG00000112096:160174492-160174646:target		21 0 20
1053	MDN1	ENSG00000112159:90399589-90399862:target		30 0 30
1054	BACH2	ENSG00000112182:90647863-90648069:source		15 6 0 36
1055	BACH2	ENSG00000112182:90916288-90916400:source		29 0 16 14
1056	BACH2	ENSG00000112182:90916288-90916400:target		11 0 20
1057	BACH2	ENSG00000112182:90981568-90981660:target		33 0 32
1058	BACH2	ENSG00000112182:91006199-91006627:target		6 13 0 22
1059	ZNF451	ENSG00000112200:56963859-56963939:source		16 10 0 29

| # | Gene | LSV ID | LSV Type | ← More in Healthy \| More in ALL → |

#	Gene	LSV ID	LSV Type	← More in Healthy | More in ALL →
1060	ZNF451	ENSG00000112200:57011888-57013491:source	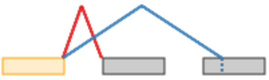	23 0 23
1061	ZNF451	ENSG00000112200:57016955-57017149:target	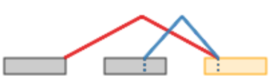	26 0 26
1062	MED23	ENSG00000112282:131921178-131921302:target	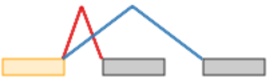	46 0 46
1063	MED23	ENSG00000112282:131931186-131931404:source	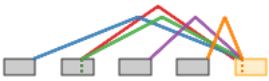	30 0 28
1064	MED23	ENSG00000112282:131939547-131939659:target	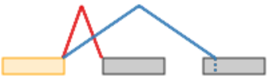	42 0 42
1065	SNX3	ENSG00000112335:108532426-108533458:source	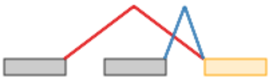	36 0 36
1066	SNX3	ENSG00000112335:108544154-108544249:target	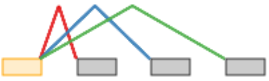	51 0 54
1067	HBS1L	ENSG00000112339:135323759-135323980:source	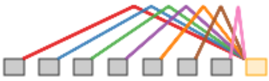	22 9 0 34
1068	HBS1L	ENSG00000112339:135360711-135360905:target	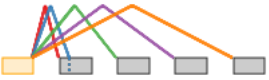	19 0 128
1069	ZBTB24	ENSG00000112365:109783797-109787777:source	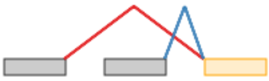	21 0 21
1070	PHF1	ENSG00000112511:33381186-33381334:source	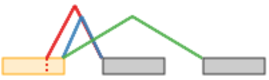	61 05 51
1071	PHF1	ENSG00000112511:33381818-33381886:target	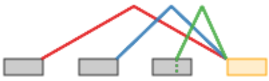	39 0 45
1072	CCND3	ENSG00000112576:41902671-41903845:source	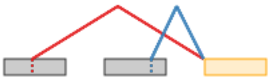	35 0 35
1073	CCND3	ENSG00000112576:41904522-41905132:source	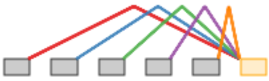	21 0 18
1074	CCND3	ENSG00000112576:41904522-41905132:target	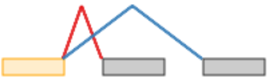	29 0 29
1075	CCND3	ENSG00000112576:41908108-41908464:source	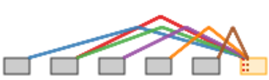	57 12 0 71
1076	SENP6	ENSG00000112701:76333616-76333676:target	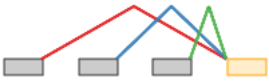	21 23 0 44
1077	PRPF4B	ENSG00000112739:4063308-4063790:target	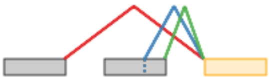	719 0 28
1078	ERBB2IP	ENSG00000112851:65290575-65290692:source	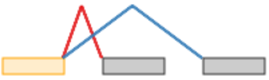	48 0 48
1079	ERBB2IP	ENSG00000112851:65349234-65350779:source	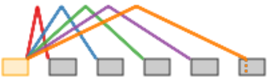	16 31 0 30 15

| # | Gene | LSV ID | LSV Type | ← More in Healthy \| More in ALL → |

#	Gene	LSV ID	LSV Type	← More in Healthy | More in ALL →
1080	ERBB2IP	ENSG00000112851:65370852-65370896:target		45 0 42
1081	MAN2A1	ENSG00000112893:109159424-109159538:source		35 0 35
1082	HMGCS1	ENSG00000112972:43297104-43297268:source		53 0 53
1083	HMGCS1	ENSG00000112972:43298246-43299077:target		60 0 60
1084	HSPA9	ENSG00000113013:137897269-137897478:source		40 0 40
1085	HSPA9	ENSG00000113013:137902690-137902852:target		45 0 45
1086	IK	ENSG00000113141:140034266-140034313:source		31 0 31
1087	HMGCR	ENSG00000113161:74650133-74650522:source		43 0 43
1088	HMGCR	ENSG00000113161:74651190-74651347:target		40 0 40
1089	CLK4	ENSG00000113240:178044698-178045779:source		11 9 0 23
1090	CLK4	ENSG00000113240:178044698-178045779:target		30 0 30
1091	CLK4	ENSG00000113240:178050257-178050417:target		38 0 11 25
1092	CLINT1	ENSG00000113282:157216371-157217569:source		8 11 16 0 25 12
1093	CLINT1	ENSG00000113282:157218628-157218732:target		14 15 0 23
1094	SUB1	ENSG00000113387:32588618-32588690:target		24 0 24
1095	SUB1	ENSG00000113387:32591354-32591793:source		20 0 21
1096	LNPEP	ENSG00000113441:96358004-96358188:source		34 0 34
1097	LNPEP	ENSG00000113441:96363437-96363518:target		32 0 36
1098	SKP1	ENSG00000113558:133502861-133502934:source		32 0 32
1099	PPP2CA	ENSG00000113575:133534777-133535141:source		48 0 48

Gene LSV ID LSV Type ← More in Healthy | More in ALL →

#	Gene	LSV ID	LSV Type	← More in Healthy | More in ALL →
1100	PPP2CA	ENSG00000113575:133536665-133536765:target	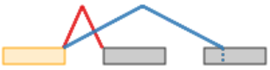	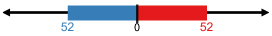
1101	H2AFY	ENSG00000113648:134681380-134683296:source	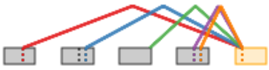	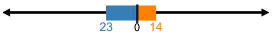
1102	H2AFY	ENSG00000113648:134696106-134698591:target	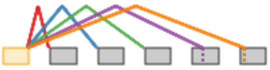	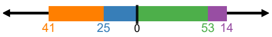
1103	TCERG1	ENSG00000113649:145834619-145834844:target	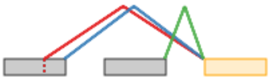	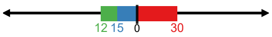
1104	TCERG1	ENSG00000113649:145859591-145860081:source	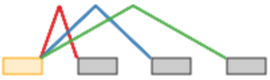	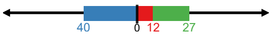
1105	TCERG1	ENSG00000113649:145862155-145862290:target	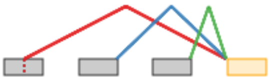	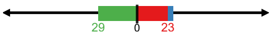
1106	TCERG1	ENSG00000113649:145882595-145883102:source	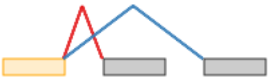	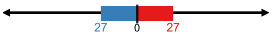
1107	TCERG1	ENSG00000113649:145886591-145886911:target	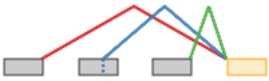	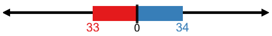
1108	CSNK1A1	ENSG00000113712:148871760-148876423:source	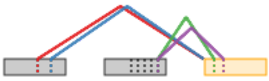	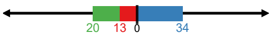
1109	CSNK1A1	ENSG00000113712:148884409-148885158:target	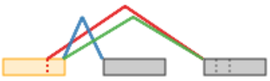	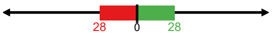
1110	ATP6V0E1	ENSG00000113732:172421778-172421825:target	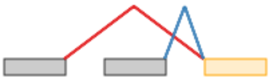	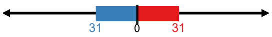
1111	ACTR8	ENSG00000113812:53906411-53906551:source	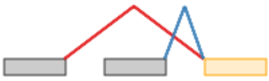	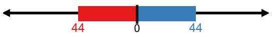
1112	ACTR8	ENSG00000113812:53908238-53908391:target	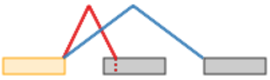	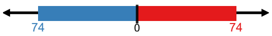
1113	ACTR8	ENSG00000113812:53909975-53910107:source	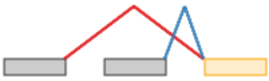	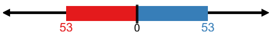
1114	ACTR8	ENSG00000113812:53911241-53911414:target	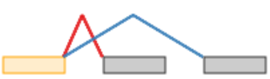	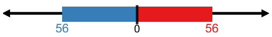
1115	NPHP3	ENSG00000113971:132297640-132298402:source	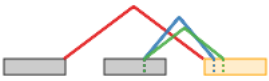	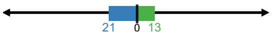
1116	KPNA1	ENSG00000114030:122186169-122186276:source	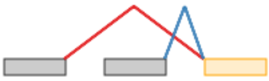	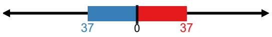
1117	KPNA1	ENSG00000114030:122215284-122215484:target	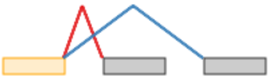	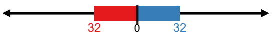
1118	UBE3A	ENSG00000114062:25599180-25599573:source	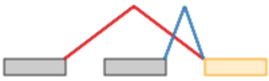	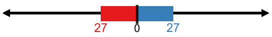
1119	UBE3A	ENSG00000114062:25601039-25601203:target	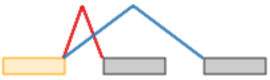	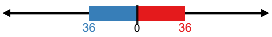

Gene LSV ID LSV Type ← More in Healthy | More in ALL →

#	Gene	LSV ID	LSV Type	← More in Healthy | More in ALL →
1120	UBE3A	ENSG00000114062:25650608-25650653:source	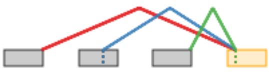	17 0 21
1121	SLC25A36	ENSG00000114120:140689207-140689835:target	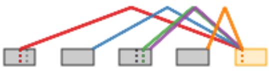	20 19 0 24 5 5
1122	XRN1	ENSG00000114127:142037434-142037536:source	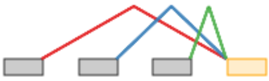	21 0 15
1123	XRN1	ENSG00000114127:142051217-142051376:source	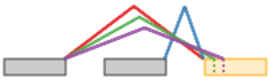	25 0 14 9
1124	XRN1	ENSG00000114127:142135982-142136071:source	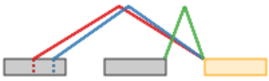	24 0 25
1125	XRN1	ENSG00000114127:142136683-142137451:target	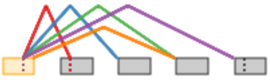	11 16 0 26
1126	XRN1	ENSG00000114127:142144271-142144380:source	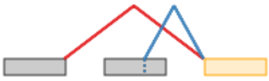	26 0 26
1127	XRN1	ENSG00000114127:142151503-142151735:target	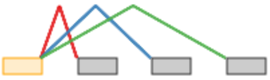	17 0 20
1128	KAT2B	ENSG00000114166:20167397-20167605:source	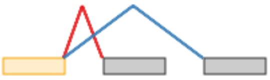	23 0 23
1129	KAT2B	ENSG00000114166:20178434-20178544:target	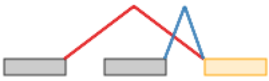	28 0 28
1130	USP4	ENSG00000114316:49348947-49349087:source	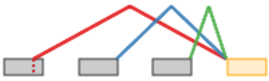	39 0 39
1131	FXR1	ENSG00000114416:180650824-180651174:target	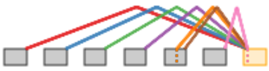	54 7 0 61
1132	FXR1	ENSG00000114416:180687946-180688449:source	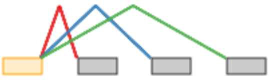	20 0 18
1133	FXR1	ENSG00000114416:180693910-180700541:target	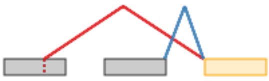	20 0 20
1134	CBLB	ENSG00000114423:105400568-105400974:target	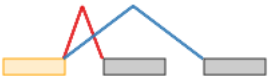	21 0 21
1135	CBLB	ENSG00000114423:105495240-105495386:source	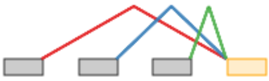	20 0 27
1136	CBLB	ENSG00000114423:105572258-105572508:target	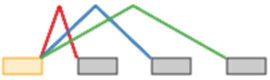	21 0 22
1137	BBX	ENSG00000114439:107429299-107429469:target	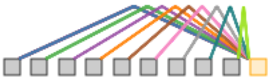	9 11 0 27
1138	IFT57	ENSG00000114446:107879659-107881502:source	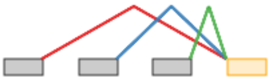	31 0 35
1139	IFT57	ENSG00000114446:107886640-107886711:target	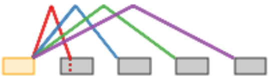	36 0 46

| # | Gene | LSV ID | LSV Type | ← More in Healthy \| More in ALL → |

#	Gene	LSV ID	LSV Type	← More in Healthy | More in ALL →
1140	KLHL24	ENSG00000114796:183353356-183353581:source	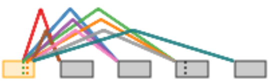	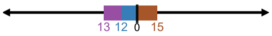
1141	KLHL24	ENSG00000114796:183361268-183361330:target	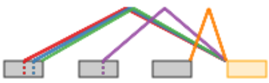	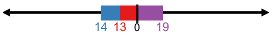
1142	FOXP1	ENSG00000114861:71161687-71161788:source	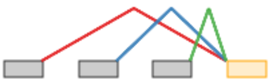	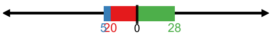
1143	EIF4G1	ENSG00000114867:184052515-184053146:target	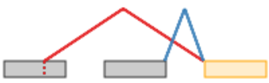	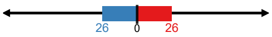
1144	SPCS1	ENSG00000114902:52740660-52740719:source	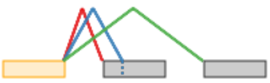	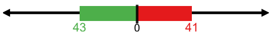
1145	SPCS1	ENSG00000114902:52741703-52742182:target	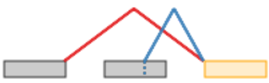	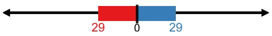
1146	KANSL3	ENSG00000114982:97267853-97268075:source	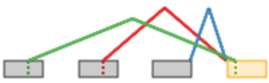	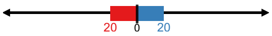
1147	KANSL3	ENSG00000114982:97268490-97268599:target	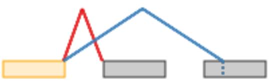	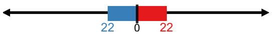
1148	KANSL3	ENSG00000114982:97269037-97270117:target	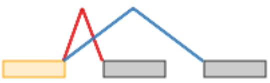	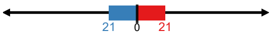
1149	KANSL3	ENSG00000114982:97276079-97276620:source	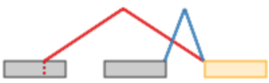	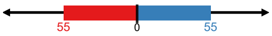
1150	KANSL3	ENSG00000114982:97277969-97278090:target	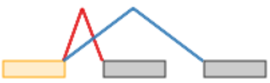	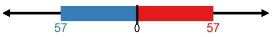
1151	PIKFYVE	ENSG00000115020:209168895-209169042:source	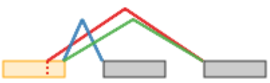	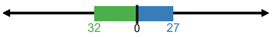
1152	PIKFYVE	ENSG00000115020:209176960-209177019:target	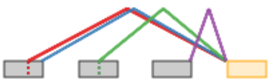	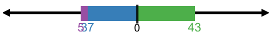
1153	PIKFYVE	ENSG00000115020:209192904-209193004:source	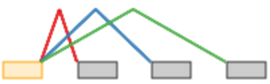	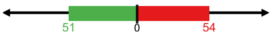
1154	PIKFYVE	ENSG00000115020:209195247-209195418:source	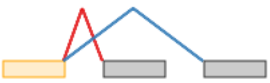	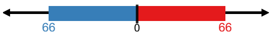
1155	PIKFYVE	ENSG00000115020:209200030-209200143:target	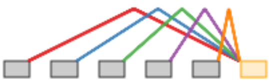	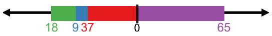
1156	PIKFYVE	ENSG00000115020:209210774-209210871:source	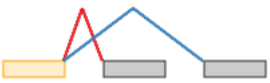	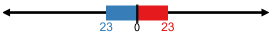
1157	PIKFYVE	ENSG00000115020:209215495-209215671:source	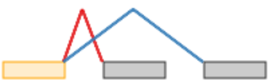	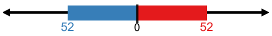
1158	PIKFYVE	ENSG00000115020:209217378-209217506:target	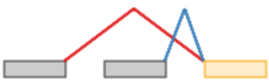	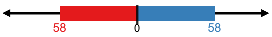
1159	NCL	ENSG00000115053:232320112-232320335:source	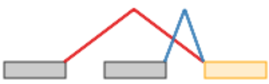	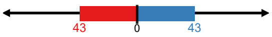
#	Gene	LSV ID	LSV Type	← More in Healthy | More in ALL →

#	Gene	LSV ID	LSV Type	← More in Healthy | More in ALL →
1160	NCL	ENSG00000115053:232321342-232321475:target		48 0 48
1161	ACTR3	ENSG00000115091:114688878-114688973:source		37 0 37
1162	GTF3C2	ENSG00000115207:27558784-27558895:target		28 0 28
1163	ITGA4	ENSG00000115232:182339687-182339793:source		57 0 22
1164	ITGA4	ENSG00000115232:182339886-182340017:source		15 13 0 30
1165	ITGA4	ENSG00000115232:182363008-182363509:source		11 21 0 33
1166	ITGA4	ENSG00000115232:182363008-182363509:target		31 0 31
1167	ITGA4	ENSG00000115232:182374385-182374534:target		30 0 30
1168	GLS	ENSG00000115419:191774989-191775047:source		21 0 17
1169	GLS	ENSG00000115419:191785750-191785782:target		18 0 20
1170	CCT4	ENSG00000115484:62095224-62095841:source		43 0 43
1171	CCT4	ENSG00000115484:62099217-62099451:target		64 0 64
1172	KDM3A	ENSG00000115548:86701899-86702113:source		25 0 20
1173	CNPPD1	ENSG00000115649:220038072-220038189:source		37 0 37
1174	CNPPD1	ENSG00000115649:220039500-220039628:target		32 0 32
1175	UXS1	ENSG00000115652:106774514-106774574:source		24 0 15
1176	STK16	ENSG00000115661:220111244-220111689:source		53 0 53
1177	STK16	ENSG00000115661:220112137-220112353:target		46 0 45
1178	HDLBP	ENSG00000115677:242175523-242176186:source		20 0 20
1179	HDLBP	ENSG00000115677:242179017-242179151:target		23 0 23

| # | Gene | LSV ID | LSV Type | ← More in Healthy \| More in ALL → |

#	Gene	LSV ID	LSV Type	← More in Healthy | More in ALL →
1180	ODC1	ENSG00000115758:10585057-10585175:target	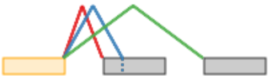	21 0 21
1181	BIRC6	ENSG00000115760:32701281-32701412:source	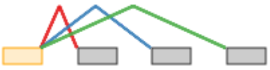	33 0 39
1182	BIRC6	ENSG00000115760:32703703-32703886:target	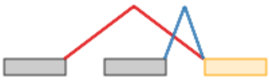	33 0 33
1183	BIRC6	ENSG00000115760:32818982-32819377:source	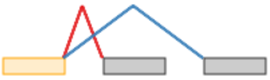	50 0 50
1184	BIRC6	ENSG00000115760:32822818-32823035:target	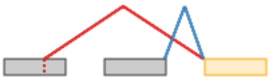	63 0 63
1185	GORASP2	ENSG00000115806:171807838-171808124:source	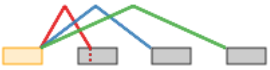	10 8 0 22
1186	CEBPZ	ENSG00000115816:37428755-37429046:source	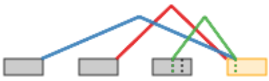	22 0 5 15
1187	PRKD3	ENSG00000115825:37487366-37487527:source	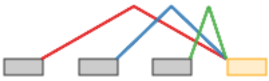	26 0 30
1188	PRKD3	ENSG00000115825:37496758-37496830:target	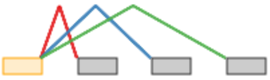	20 0 17
1189	PRKD3	ENSG00000115825:37502910-37502987:source	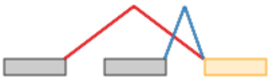	25 0 25
1190	PRKD3	ENSG00000115825:37506889-37507072:target	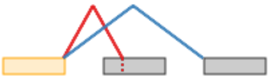	28 0 28
1191	PRKD3	ENSG00000115825:37518011-37518142:source	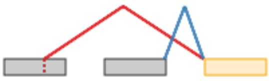	29 0 29
1192	RAB3GAP1	ENSG00000115839:135893134-135893502:source	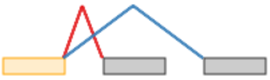	22 0 22
1193	RAB3GAP1	ENSG00000115839:135911219-135911446:source	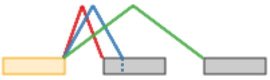	6 15 0 24
1194	WIPF1	ENSG00000115935:175435897-175437174:source	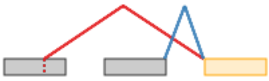	21 0 21
1195	ATF2	ENSG00000115966:175944991-175945493:source	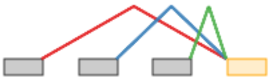	41 0 41
1196	ATF2	ENSG00000115966:175957789-175957995:target	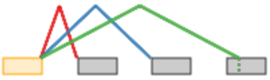	44 0 45
1197	ATF2	ENSG00000115966:176001140-176001214:source	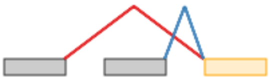	26 0 26
1198	ATF2	ENSG00000115966:176015788-176015886:target	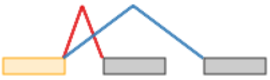	20 0 20
1199	C2orf42	ENSG00000115998:70454867-70454954:source	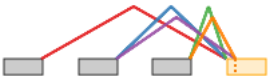	8 17 0 5 17

| # | Gene | LSV ID | LSV Type | ← More in Healthy \| More in ALL → |

#	Gene	LSV ID	LSV Type	← More in Healthy | More in ALL →
1200	C2orf42	ENSG00000115998:70456191-70456223:target	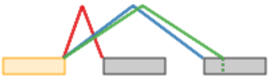	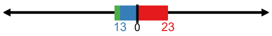 13 0 23
1201	TIA1	ENSG00000116001:70454867-70454971:source	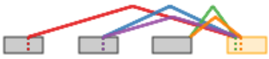	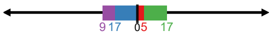 9 17 0 5 17
1202	TIA1	ENSG00000116001:70456030-70456223:target	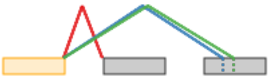	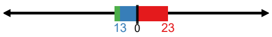 13 0 23
1203	MSH6	ENSG00000116062:48033918-48037240:target	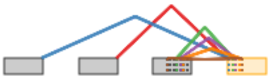	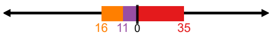 16 11 0 35
1204	RALGPS2	ENSG00000116191:178802559-178802685:source	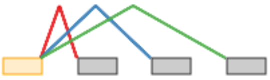	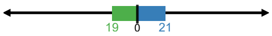 19 0 21
1205	SRSF4	ENSG00000116350:29486887-29487307:source	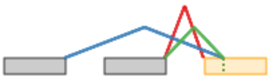	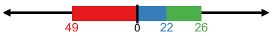 49 0 22 26
1206	SRSF4	ENSG00000116350:29508158-29508499:target	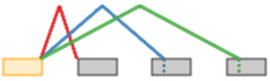	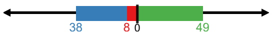 38 8 0 49
1207	HDAC1	ENSG00000116478:32797691-32797843:source	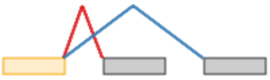	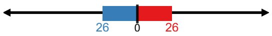 26 0 26
1208	CAPZA1	ENSG00000116489:113196220-113196283:target	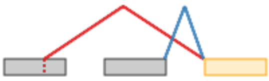	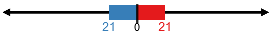 21 0 21
1209	CAPZA1	ENSG00000116489:113197087-113197293:target	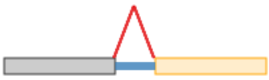	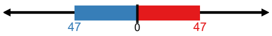 47 0 47
1210	CAPZA1	ENSG00000116489:113201951-113202401:source	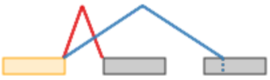	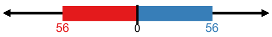 56 0 56
1211	CAPZA1	ENSG00000116489:113209542-113209767:target	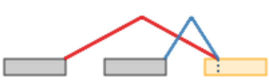	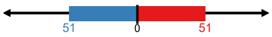 51 0 51
1212	SFPQ	ENSG00000116560:35642810-35643262:target	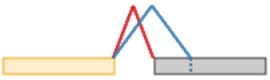	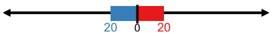 20 0 20
1213	GON4L	ENSG00000116580:155744598-155744964:target	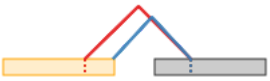	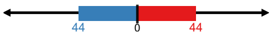 44 0 44
1214	GON4L	ENSG00000116580:155783425-155783595:source	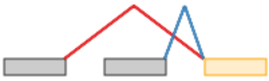	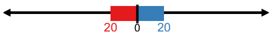 20 0 20
1215	GON4L	ENSG00000116580:155785596-155785691:target	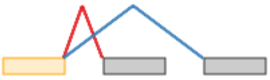	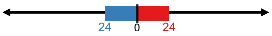 24 0 24
1216	ARHGEF2	ENSG00000116584:155920060-155920273:target	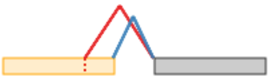	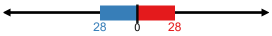 28 0 28
1217	ARHGEF2	ENSG00000116584:155935094-155935203:source	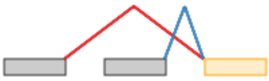	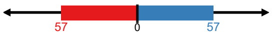 57 0 57
1218	ARHGEF2	ENSG00000116584:155936207-155936270:target	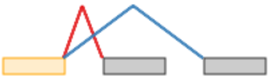	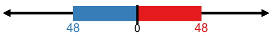 48 0 48
1219	MEF2D	ENSG00000116604:156438572-156438812:source	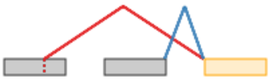	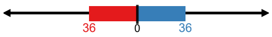 36 0 36
#	Gene	LSV ID	LSV Type	← More in Healthy | More in ALL →

#	Gene	LSV ID	LSV Type	← More in Healthy | More in ALL →
1220	MEF2D	ENSG00000116604:156446721-156446994:target		45 0 39
1221	IVNS1ABP	ENSG00000116679:185274668-185274775:source		51 0 51
1222	IVNS1ABP	ENSG00000116679:185275882-185276271:target		45 0 45
1223	SMG7	ENSG00000116698:183510119-183510238:source		20 0 15
1224	SMG7	ENSG00000116698:183513488-183513632:target		39 0 10 26
1225	SMG7	ENSG00000116698:183518076-183518423:target		24 0 24
1226	SMG7	ENSG00000116698:183520180-183520325:source		28 0 30
1227	PRDM2	ENSG00000116731:14068500-14068652:target		32 0 32
1228	SRSF11	ENSG00000116754:70687145-70687522:source		19 0 20
1229	SRSF11	ENSG00000116754:70694105-70694410:target		47 0 47
1230	SRSF11	ENSG00000116754:70712413-70712590:source		19 0 20
1231	LGALS8	ENSG00000116977:236711312-236716281:target		16 5 0 25
1232	KDM5B	ENSG00000117139:202698444-202699155:target		20 0 20
1233	KDM5B	ENSG00000117139:202731827-202731936:source		46 0 46
1234	KDM5B	ENSG00000117139:202742246-202742416:target		50 12 0 60
1235	APH1A	ENSG00000117362:150241098-150241703:target		32 0 39
1236	PRRC2C	ENSG00000117523:171537386-171537478:source		22 0 22
1237	PRRC2C	ENSG00000117523:171544066-171544267:target		15 5 0 24
1238	PTBP2	ENSG00000117569:97250504-97250810:target		26 0 26
1239	RSRP1	ENSG00000117616:25569030-25569196:target		23 0 23
#	Gene	LSV ID	LSV Type	← More in Healthy | More in ALL →

#	Gene	LSV ID	LSV Type	← More in Healthy | More in ALL →
1240	RCOR3	ENSG00000117625:211447552-211447604:target		40 0 40
1241	RCOR3	ENSG00000117625:211449599-211449760:source		27 0 27
1242	RCOR3	ENSG00000117625:211474803-211477482:source		67 0 26 41
1243	RCOR3	ENSG00000117625:211474803-211477482:target		9 18 0 29
1244	RCOR3	ENSG00000117625:211486062-211486640:target		53 0 53
1245	RCOR3	ENSG00000117625:211486766-211489727:target		41 0 40
1246	ESYT2	ENSG00000117868:158542340-158542414:source		38 0 38
1247	ESYT2	ENSG00000117868:158552177-158552272:target		41 0 41
1248	ESYT2	ENSG00000117868:158580695-158580784:target		26 0 26
1249	KMT2A	ENSG00000118058:118354898-118355046:source		59 0 59
1250	KMT2A	ENSG00000118058:118354898-118355046:target		29 0 29
1251	KMT2A	ENSG00000118058:118359329-118359475:target		66 0 66
1252	KMT2A	ENSG00000118058:118377276-118377361:source		24 0 31
1253	KMT2A	ENSG00000118058:118379851-118379915:target		25 0 25
1254	KLF7	ENSG00000118263:207988498-207989132:source		44 0 47
1255	KLF7	ENSG00000118263:208030191-208030430:target		32 0 32
1256	LRMP	ENSG00000118308:25232178-25232224:target		19 0 23
1257	LRMP	ENSG00000118308:25255132-25255712:source		73 0 73
1258	LRMP	ENSG00000118308:25255132-25255712:target		32 0 32
1259	HMGN3	ENSG00000118418:79911993-79912106:target		22 12 0 36
#	Gene	LSV ID	LSV Type	← More in Healthy | More in ALL →

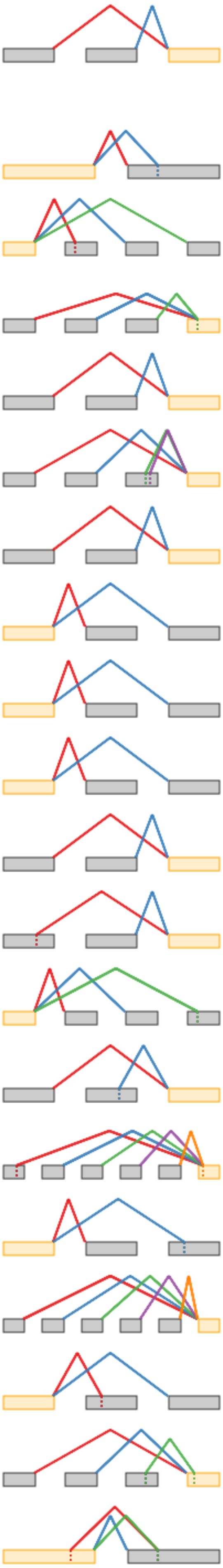

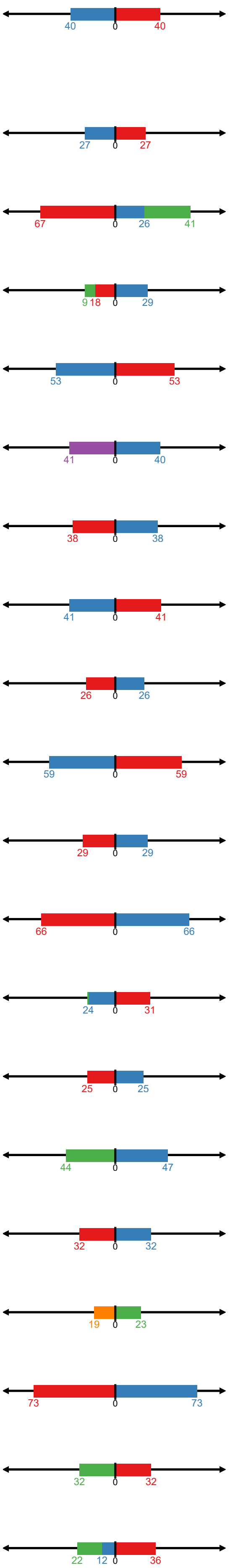

#	Gene	LSV ID	LSV Type	← More in Healthy | More in ALL →
1260	PHF3	ENSG00000118482:64345725-64346055:source	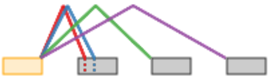	38 0 32
1261	PHF3	ENSG00000118482:64389901-64390062:target	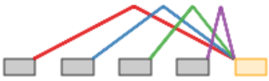	9 17 0 27
1262	PHF3	ENSG00000118482:64415919-64416114:source	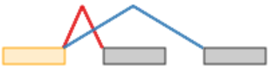	40 0 40
1263	PHF3	ENSG00000118482:64419047-64419136:target	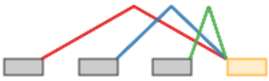	25 0 27
1264	TNFAIP3	ENSG00000118503:138192350-138192659:target	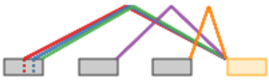	20 0 18
1265	RAB3GAP2	ENSG00000118873:220340844-220341017:source	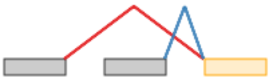	41 0 41
1266	RAB3GAP2	ENSG00000118873:220345231-220345391:target	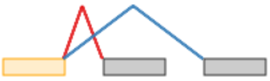	47 0 47
1267	RAB3GAP2	ENSG00000118873:220363406-220363488:source	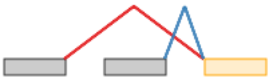	47 0 47
1268	RAB3GAP2	ENSG00000118873:220364410-220364626:target	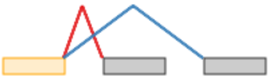	47 0 47
1269	PTBP3	ENSG00000119314:115030329-115030475:source	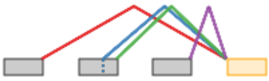	22 0 118
1270	PTBP3	ENSG00000119314:115060112-115060196:source	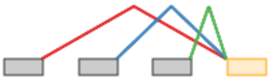	26 0 26
1271	CNTRL	ENSG00000119397:123887994-123888214:source	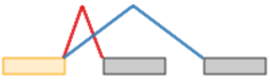	25 0 25
1272	CNTRL	ENSG00000119397:123900823-123900957:target	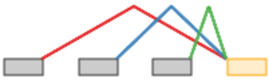	28 0 26
1273	CNTRL	ENSG00000119397:123908386-123908534:source	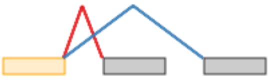	20 0 20
1274	CNTRL	ENSG00000119397:123919736-123919878:source	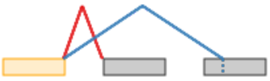	45 0 45
1275	CNTRL	ENSG00000119397:123920864-123921297:target	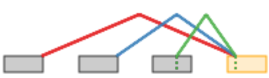	38 0 35
1276	CNTRL	ENSG00000119397:123922421-123922580:source	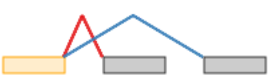	48 0 48
1277	CNTRL	ENSG00000119397:123931889-123932094:source	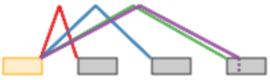	22 0 10
1278	CNTRL	ENSG00000119397:123935521-123935787:target	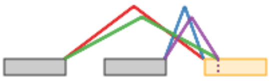	26 0 125
1279	PPP6C	ENSG00000119414:127920520-127920661:source	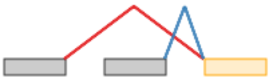	21 0 21

Gene LSV ID LSV Type ← More in Healthy | More in ALL →

#	Gene	LSV ID	LSV Type	← More in Healthy | More in ALL →
1280	PPP6C	ENSG00000119414:127933364-127933459:target	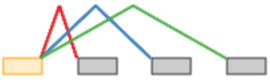	17 16 0 31
1281	MAPKAP1	ENSG00000119487:128246722-128246862:target	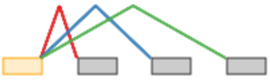	27 0 28
1282	MAPKAP1	ENSG00000119487:128305338-128305447:source	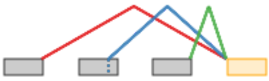	22 0 23
1283	DLST	ENSG00000119689:75352289-75352727:target	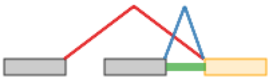	20 0 18
1284	DLST	ENSG00000119689:75359537-75359689:target	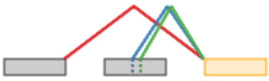	30 0 30
1285	DLST	ENSG00000119689:75360051-75360127:source	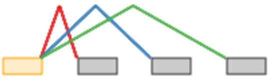	47 0 51
1286	DLST	ENSG00000119689:75365072-75365202:target	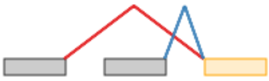	49 0 49
1287	SLIRP	ENSG00000119705:78186859-78187450:source	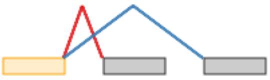	45 0 45
1288	SLIRP	ENSG00000119705:78197331-78197472:target	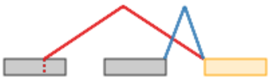	40 0 40
1289	RBM25	ENSG00000119707:73566375-73566458:source	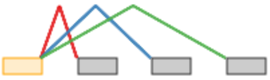	31 0 28
1290	ZNF410	ENSG00000119725:74353320-74353653:source	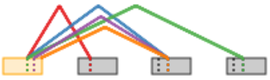	29 0 27
1291	ZNF410	ENSG00000119725:74358553-74358911:target	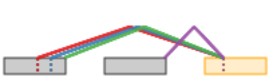	45 0 45
1292	ZNF410	ENSG00000119725:74386849-74388030:source	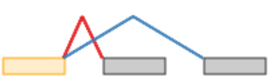	30 0 30
1293	ZNF410	ENSG00000119725:74390098-74390228:target	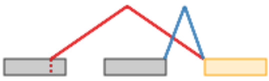	24 0 24
1294	RHOQ	ENSG00000119729:46808067-46810260:target	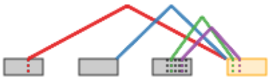	11 27 0 36
1295	ATL2	ENSG00000119787:38536520-38536648:source	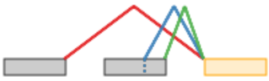	38 19 0 57
1296	ATL2	ENSG00000119787:38540293-38540385:target	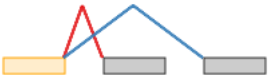	22 0 22
1297	YPEL5	ENSG00000119801:30369807-30369928:source	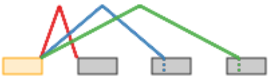	17 0 24
1298	YPEL5	ENSG00000119801:30378681-30378776:target	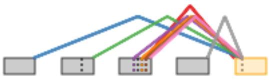	39 15 0 66
1299	YPEL5	ENSG00000119801:30379244-30379658:target	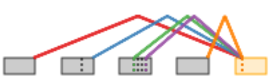	19 0 26

Gene LSV ID LSV Type ← More in Healthy | More in ALL →

#	Gene	LSV ID	LSV Type	← More in Healthy | More in ALL →
1300	AFTPH	ENSG00000119844:64800080-64800202:source		23 0 23
1301	AFTPH	ENSG00000119844:64812556-64812679:source		23 0 23
1302	AFTPH	ENSG00000119844:64812556-64812679:target		25 0 11 11
1303	BCL11A	ENSG00000119866:60695867-60695968:source		6 24 0 34
1304	BCL11A	ENSG00000119866:60773106-60773435:target		18 0 23
1305	WDR11	ENSG00000120008:122630682-122630858:source		20 0 20
1306	WDR11	ENSG00000120008:122643292-122643400:target		20 14 0 36
1307	WDR11	ENSG00000120008:122664831-122665168:source		14 0 21
1308	GNA13	ENSG00000120063:63006833-63010947:source		20 7 0 30
1309	GNA13	ENSG00000120063:63014371-63014421:target		46 0 46
1310	KANSL1	ENSG00000120071:44248221-44248485:target		17 5 0 22
1311	KANSL1	ENSG00000120071:44249093-44249598:source		9 18 0 29
1312	CAAP1	ENSG00000120159:26884808-26884900:target		49 0 49
1313	TCP1	ENSG00000120438:160201475-160201598:source		27 0 27
1314	TCP1	ENSG00000120438:160204973-160205099:target		30 0 30
1315	MASTL	ENSG00000120539:27469882-27469995:target		21 0 21
1316	UFM1	ENSG00000120686:38928376-38929022:target		45 0 45
1317	ELF1	ENSG00000120690:41556119-41556418:source		27 0 10 15
1318	HSPH1	ENSG00000120694:31722096-31722229:source		27 0 27
1319	KDM3B	ENSG00000120733:137713409-137713514:source		22 0 22

Gene LSV ID LSV Type ← More in Healthy | More in ALL →

#	Gene	LSV ID	LSV Type	← More in Healthy | More in ALL →
1320	KDM3B	ENSG00000120733:137717205-137717437:target		25 0 27
1321	TMPO	ENSG00000120802:98931253-98931491:source		20 0 5 10
1322	APAF1	ENSG00000120868:99093186-99093347:source		55 0 55
1323	APAF1	ENSG00000120868:99093186-99093347:target		20 0 20
1324	APAF1	ENSG00000120868:99100263-99100388:target		59 0 59
1325	PTK2B	ENSG00000120899:27254969-27255306:target		23 0 16 5
1326	PTK2B	ENSG00000120899:27301723-27301788:source		23 0 9 13
1327	PTK2B	ENSG00000120899:27308266-27308412:target		22 0 22
1328	KIAA0922	ENSG00000121210:154478118-154478234:source		30 0 30
1329	KIAA0922	ENSG00000121210:154512205-154512346:source		35 0 33
1330	KIAA0922	ENSG00000121210:154514951-154515023:target		37 0 38
1331	PSPC1	ENSG00000121390:20251864-20252078:target		28 0 28
1332	PSPC1	ENSG00000121390:20304379-20304484:target		25 7 0 35
1333	PILRB	ENSG00000121716:99949786-99950101:target		34 0 37
1334	ZMYM2	ENSG00000121741:20534098-20534166:source		31 0 9 20
1335	ZMYM2	ENSG00000121741:20544165-20544211:target		40 0 40
1336	ZMYM2	ENSG00000121741:20567203-20567704:target		33 0 33
1337	ZMYM2	ENSG00000121741:20593687-20594103:source		66 0 66
1338	ZMYM2	ENSG00000121741:20600659-20600902:target		69 0 69
1339	ZMYM2	ENSG00000121741:20641379-20641530:source		21 6 5 0 36

Gene LSV ID LSV Type ← More in Healthy | More in ALL →

#	Gene	LSV ID	LSV Type	← More in Healthy | More in ALL →
1340	TBC1D15	ENSG00000121749:72265850-72265948:target	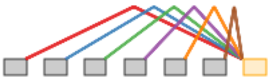	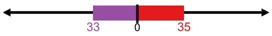
1341	PDS5A	ENSG00000121892:39847438-39850181:source	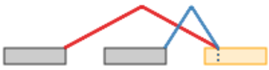	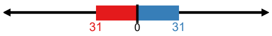
1342	PDS5A	ENSG00000121892:39851140-39851272:target	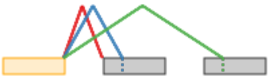	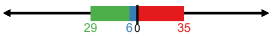
1343	PDS5A	ENSG00000121892:39864956-39865079:source	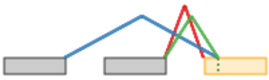	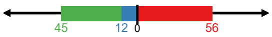
1344	PDS5A	ENSG00000121892:39871014-39871082:target	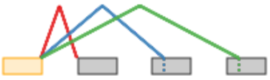	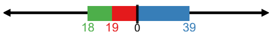
1345	PDS5A	ENSG00000121892:39910015-39910504:source	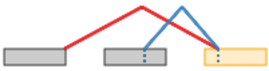	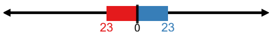
1346	PDS5A	ENSG00000121892:39915231-39915346:target	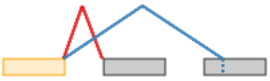	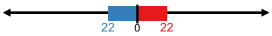
1347	TMEM156	ENSG00000121895:39033846-39034063:target	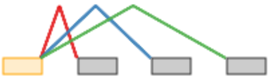	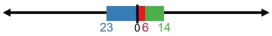
1348	ZRANB3	ENSG00000121988:135911219-135911446:source	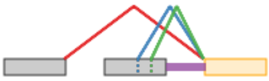	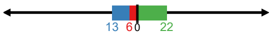
1349	RPL21	ENSG00000122026:27827842-27827980:target	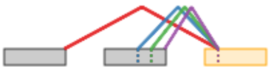	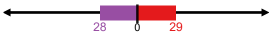
1350	GTF3A	ENSG00000122034:28004670-28004758:source	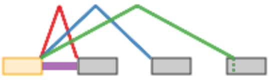	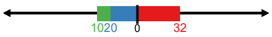
1351	GTF3A	ENSG00000122034:28008276-28008356:target	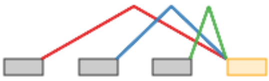	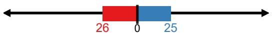
1352	FYTTD1	ENSG00000122068:197482969-197483428:target	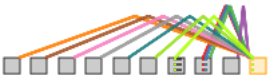	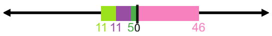
1353	COPA	ENSG00000122218:160263176-160263253:source	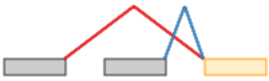	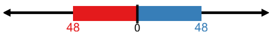
1354	COPA	ENSG00000122218:160264558-160264647:target	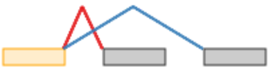	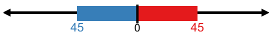
1355	COPA	ENSG00000122218:160276144-160276283:source	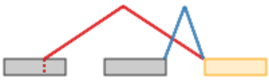	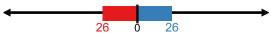
1356	COPA	ENSG00000122218:160278720-160278966:target	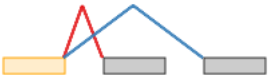	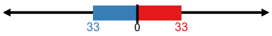
1357	COPA	ENSG00000122218:160282659-160282957:target	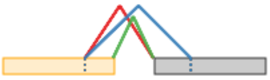	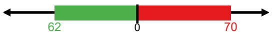
1358	LY9	ENSG00000122224:160769476-160770017:source	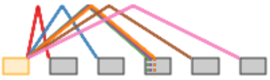	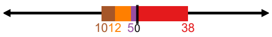
1359	LY9	ENSG00000122224:160779539-160783701:target	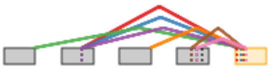	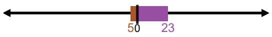

Gene LSV ID LSV Type ← More in Healthy | More in ALL →

#	Gene	LSV ID	LSV Type	← More in Healthy | More in ALL →
1360	LY9	ENSG00000122224:160784210-160785129:source		5 30 0 39
1361	LY9	ENSG00000122224:160788008-160788109:target		20 0 35
1362	LY9	ENSG00000122224:160789111-160789164:source		25 0 28
1363	LY9	ENSG00000122224:160789111-160789164:target		10 0 24
1364	LY9	ENSG00000122224:160793152-160793325:target		13 5 0 23
1365	LY9	ENSG00000122224:160793416-160793586:source		29 0 29
1366	LY9	ENSG00000122224:160797538-160798051:target		25 25 0 46
1367	RBBP6	ENSG00000122257:24580066-24581820:target		28 0 28
1368	ZC3H7A	ENSG00000122299:11855766-11855899:source		24 0 24
1369	ZC3H7A	ENSG00000122299:11862171-11862377:source		33 0 34
1370	ZC3H7A	ENSG00000122299:11864639-11864843:target		28 0 28
1371	ANXA11	ENSG00000122359:81910645-81915668:source		28 0 33
1372	ODF2L	ENSG00000122417:86848614-86848787:target		21 0 21
1373	ZMIZ2	ENSG00000122515:44800024-44800192:target		10 25 0 30 7
1374	HNRNPA2B1	ENSG00000122566:26229547-26230080:source		45 0 39
1375	HNRNPA2B1	ENSG00000122566:26230613-26231298:target		42 0 35
1376	HNRNPA2B1	ENSG00000122566:26232115-26232197:target		41 0 40
1377	HNRNPA2B1	ENSG00000122566:26232871-26232993:source		44 0 44
1378	HNRNPA2B1	ENSG00000122566:26235332-26235529:target		32 0 32
1379	SMU1	ENSG00000122692:33057596-33057712:source		28 0 28

| # | Gene | LSV ID | LSV Type | ← More in Healthy \| More in ALL → |

#	Gene	LSV ID	LSV Type	← More in Healthy | More in ALL →
1380	SMU1	ENSG00000122692:33062047-33062175:target	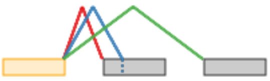	38 0 34
1381	SMU1	ENSG00000122692:33068822-33068932:source	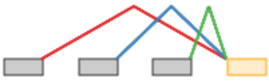	52 0 51
1382	SMU1	ENSG00000122692:33073594-33073804:target	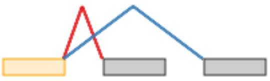	54 0 54
1383	CLTA	ENSG00000122705:36233917-36234129:source	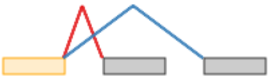	41 0 41
1384	ECD	ENSG00000122882:74916033-74916211:target	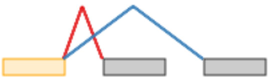	21 0 21
1385	ECD	ENSG00000122882:74920192-74920309:source	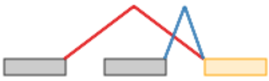	35 0 35
1386	ECD	ENSG00000122882:74927624-74928144:target	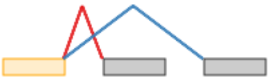	38 0 38
1387	P4HA1	ENSG00000122884:74806683-74806859:target	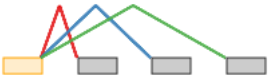	21 0 31
1388	HVCN1	ENSG00000122986:111093039-111093143:source	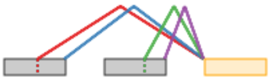	48 10 0 60
1389	HVCN1	ENSG00000122986:111098969-111099253:target	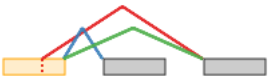	57 0 59
1390	DDX54	ENSG00000123064:113600523-113600850:source	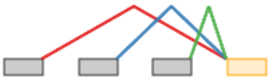	20 7 0 30
1391	MED13L	ENSG00000123066:116424914-116425093:target	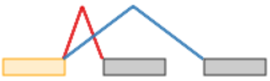	29 0 29
1392	ADGRE5	ENSG00000123146:14501736-14502009:source	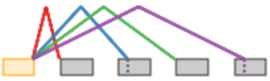	33 0 33
1393	ADGRE5	ENSG00000123146:14508226-14508616:target	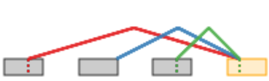	23 0 23
1394	ADGRE5	ENSG00000123146:14516560-14516751:source	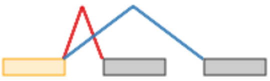	31 0 31
1395	ADGRE5	ENSG00000123146:14517681-14517772:source	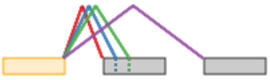	26 0 36
1396	NCKAP1L	ENSG00000123338:54911590-54911722:source	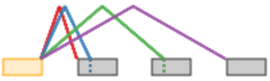	15 13 0 31
1397	NCKAP1L	ENSG00000123338:54912971-54913116:target	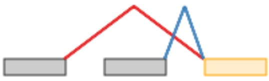	22 0 22
1398	NR4A1	ENSG00000123358:52447202-52449090:target	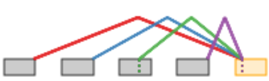	29 0 35
1399	BAZ2B	ENSG00000123636:160252280-160252345:source	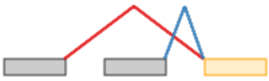	50 0 50

| # | Gene | LSV ID | LSV Type | ← More in Healthy \| More in ALL → |

#	Gene	LSV ID	LSV Type	← More in Healthy | More in ALL →
1400	BAZ2B	ENSG00000123636:160253856-160253900:target		48 0 48
1401	BAZ2B	ENSG00000123636:160335086-160335232:target		11 0 22
1402	ACSL3	ENSG00000123983:223789174-223789313:source		53 0 53
1403	ACSL3	ENSG00000123983:223793580-223793654:target		59 0 59
1404	PREX1	ENSG00000124126:47274674-47274766:source		26 0 26
1405	PREX1	ENSG00000124126:47282821-47282893:target		35 0 35
1406	NCOA3	ENSG00000124151:46262762-46262939:target		17 10 0 8 20
1407	NCOA3	ENSG00000124151:46268321-46268566:target		22 0 25
1408	VAPB	ENSG00000124164:56993267-56993419:source		52 0 47
1409	VAPB	ENSG00000124164:57014001-57014081:target		42 0 42
1410	CHD6	ENSG00000124177:40041964-40043705:source		29 0 36
1411	CHD6	ENSG00000124177:40041964-40043705:target		21 0 21
1412	CHD6	ENSG00000124177:40117068-40117244:target		21 0 21
1413	SRSF6	ENSG00000124193:42087001-42087149:source		30 0 30
1414	SRSF6	ENSG00000124193:42088411-42088535:target		28 0 24
1415	ARFGEF2	ENSG00000124198:47585684-47585814:source		35 0 43
1416	ARFGEF2	ENSG00000124198:47585684-47585814:target		21 0 21
1417	ARFGEF2	ENSG00000124198:47592553-47592736:target		33 0 40
1418	ZNFX1	ENSG00000124201:47873954-47874201:target		22 0 23
1419	ZNFX1	ENSG00000124201:47877000-47877114:source		39 0 39

| # | Gene | LSV ID | LSV Type | ← More in Healthy \| More in ALL → |

#	Gene	LSV ID	LSV Type	← More in Healthy | More in ALL →
1420	CSE1L	ENSG00000124207:47700563-47700699:source	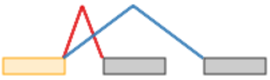	32 0 32
1421	CSE1L	ENSG00000124207:47704546-47704643:target	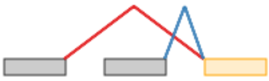	44 0 44
1422	CSE1L	ENSG00000124207:47710677-47710823:source	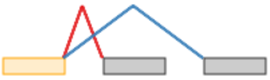	63 0 63
1423	CSE1L	ENSG00000124207:47710677-47710823:target	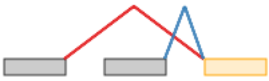	23 0 23
1424	CSE1L	ENSG00000124207:47711269-47711500:target	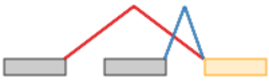	64 0 64
1425	RAB22A	ENSG00000124209:56886099-56886178:source	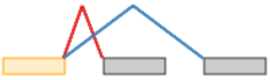	33 0 33
1426	STAU1	ENSG00000124214:47770470-47770608:source	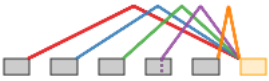	26 36 0 64
1427	STAU1	ENSG00000124214:47790732-47790806:target	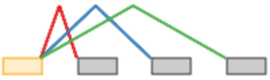	14 49 0 64
1428	STX16	ENSG00000124222:57242857-57243183:source	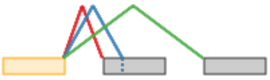	23 0 23
1429	PPP4R1L	ENSG00000124224:56823171-56823329:source	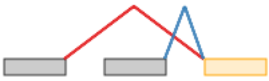	20 0 20
1430	MTRR	ENSG00000124275:7875275-7875488:target	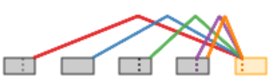	25 0 24
1431	NAGK	ENSG00000124357:71303734-71303831:target	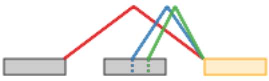	22 0 28
1432	ATP8A1	ENSG00000124406:42448600-42448661:source	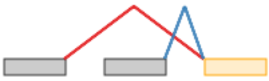	22 0 22
1433	ATP8A1	ENSG00000124406:42457314-42457450:target	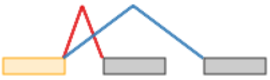	31 0 31
1434	ATP8A1	ENSG00000124406:42466910-42467093:source	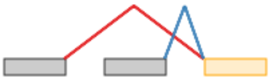	31 0 31
1435	ATP8A1	ENSG00000124406:42505467-42505531:target	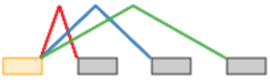	13 30 0 45
1436	ATP8A1	ENSG00000124406:42509033-42509171:source	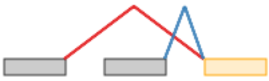	22 0 22
1437	ATP8A1	ENSG00000124406:42590277-42590634:source	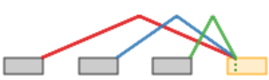	44 0 40
1438	CDKN1A	ENSG00000124762:36646435-36646716:source	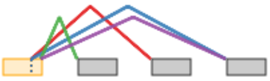	32 0 33
1439	CDKN1A	ENSG00000124762:36651874-36652323:target	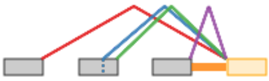	629 0 39
#	Gene	LSV ID	LSV Type	← More in Healthy | More in ALL →

#	Gene	LSV ID	LSV Type	← More in Healthy | More in ALL →
1440	RIOK1	ENSG00000124784:7393332-7393536:target		20 0 20
1441	RIOK1	ENSG00000124784:7402836-7402948:source		22 0 22
1442	NUP153	ENSG00000124789:17632881-17633075:source		20 0 20
1443	NUP153	ENSG00000124789:17637384-17638001:target		21 0 14
1444	NUP153	ENSG00000124789:17649394-17649531:source		21 0 13
1445	LRRFIP1	ENSG00000124831:238600788-238601164:source		80 0 80
1446	LRRFIP1	ENSG00000124831:238657007-238657042:target		68 0 61
1447	AHNAK	ENSG00000124942:62289186-62289417:source		21 0 20
1448	SH3TC1	ENSG00000125089:8217930-8217984:source		23 5 0 30
1449	SH3TC1	ENSG00000125089:8232869-8233883:source		47 0 43
1450	SH3TC1	ENSG00000125089:8237160-8237317:source		66 0 66
1451	SH3TC1	ENSG00000125089:8239201-8239397:target		37 0 37
1452	CNOT1	ENSG00000125107:58570872-58571124:target		30 0 30
1453	CNOT1	ENSG00000125107:58585694-58585723:source		32 0 32
1454	CNOT1	ENSG00000125107:58589155-58589441:target		32 0 32
1455	CNOT1	ENSG00000125107:58590751-58590897:source		41 0 348
1456	CNOT1	ENSG00000125107:58594116-58594266:target		737 0 47
1457	CNOT1	ENSG00000125107:58632914-58633415:source		42 0 255 11
1458	TM9SF2	ENSG00000125304:100189993-100190117:source		60 0 60
1459	TM9SF2	ENSG00000125304:100192765-100193047:target		70 0 70

Gene LSV ID LSV Type ← More in Healthy | More in ALL →

#	Gene	LSV ID	LSV Type	← More in Healthy | More in ALL →
1460	IRF1	ENSG00000125347:131820054-131820189:source	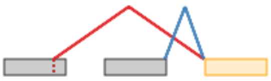	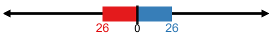 26 0 26
1461	IRF1	ENSG00000125347:131821359-131821408:source	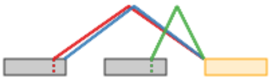	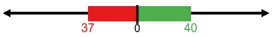 37 0 40
1462	IRF1	ENSG00000125347:131822214-131822378:target	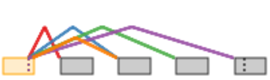	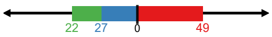 22 27 0 49
1463	IRF1	ENSG00000125347:131822646-131822822:source	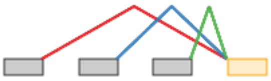	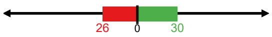 26 0 30
1464	IRF1	ENSG00000125347:131826237-131826490:target	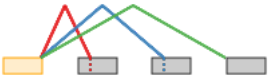	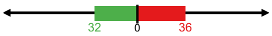 32 0 36
1465	FAM193A	ENSG00000125386:2659530-2659607:target	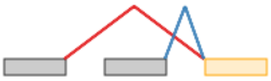	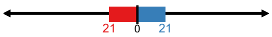 21 0 21
1466	FAM193A	ENSG00000125386:2717750-2717831:target	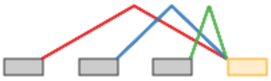	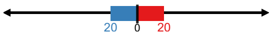 20 0 20
1467	SLC25A35	ENSG00000125434:8192178-8192758:source	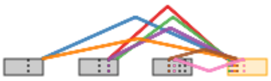	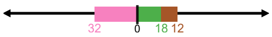 32 0 18 12
1468	SLC25A35	ENSG00000125434:8192178-8192758:target	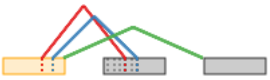	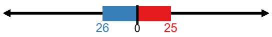 26 0 25
1469	MSTO1	ENSG00000125459:155640111-155640255:source	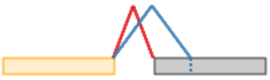	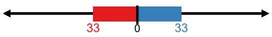 33 0 33
1470	CCDC93	ENSG00000125633:118694088-118694477:source	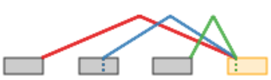	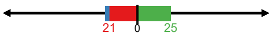 21 0 25
1471	CCDC93	ENSG00000125633:118703105-118703158:target	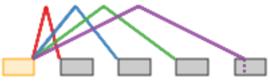	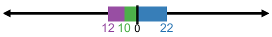 12 10 0 22
1472	CCDC93	ENSG00000125633:118753840-118753938:source	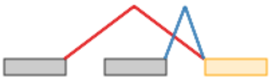	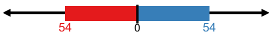 54 0 54
1473	CCDC93	ENSG00000125633:118764298-118764392:target	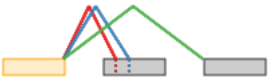	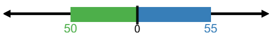 50 0 55
1474	PSD4	ENSG00000125637:113956660-113956803:target	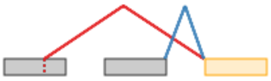	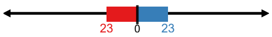 23 0 23
1475	SYMPK	ENSG00000125755:46320056-46320391:target	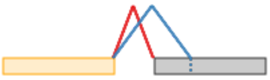	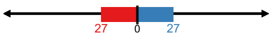 27 0 27
1476	GPCPD1	ENSG00000125772:5564927-5566683:source	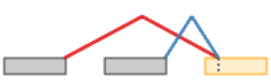	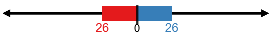 26 0 26
1477	GPCPD1	ENSG00000125772:5573973-5574057:target	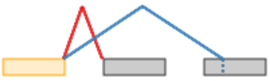	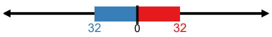 32 0 32
1478	GPCPD1	ENSG00000125772:5585010-5585086:source	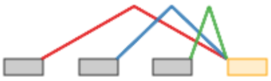	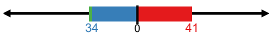 34 0 41
1479	SNRPB	ENSG00000125835:2443735-2443873:source	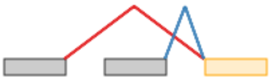	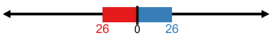 26 0 26

Gene LSV ID LSV Type ← More in Healthy | More in ALL →

#	Gene	LSV ID	LSV Type	← More in Healthy | More in ALL →
1480	SNRPB	ENSG00000125835:2446354-2446465:target	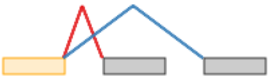	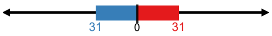
1481	HNRNPR	ENSG00000125944:23648021-23648156:source	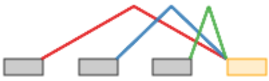	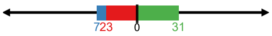
1482	HNRNPR	ENSG00000125944:23650049-23650225:source	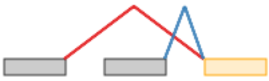	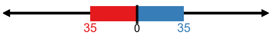
1483	HNRNPR	ENSG00000125944:23664247-23664354:target	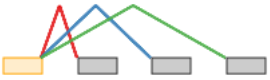	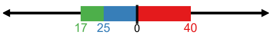
1484	HNRNPR	ENSG00000125944:23664763-23665101:source	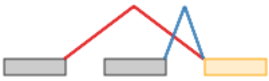	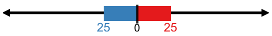
1485	MAX	ENSG00000125952:65544492-65544754:target	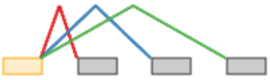	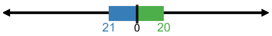
1486	RALY	ENSG00000125970:32678323-32678408:source	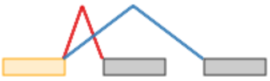	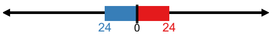
1487	RALY	ENSG00000125970:32685222-32685322:target	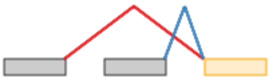	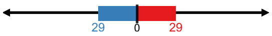
1488	EIF2S2	ENSG00000125977:32678323-32678408:source	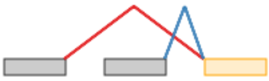	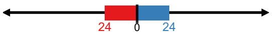
1489	EIF2S2	ENSG00000125977:32685222-32685322:target	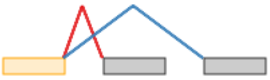	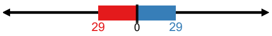
1490	UBA2	ENSG00000126261:34925773-34925873:source	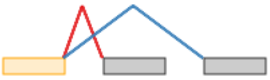	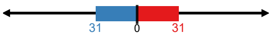
1491	UBA2	ENSG00000126261:34934749-34934816:target	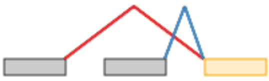	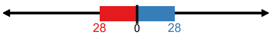
1492	STAT5A	ENSG00000126561:40447637-40447811:target	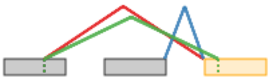	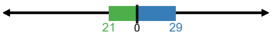
1493	BECN1	ENSG00000126581:40962152-40962946:source	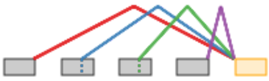	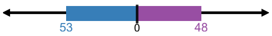
1494	BECN1	ENSG00000126581:40966186-40966611:target	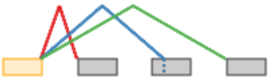	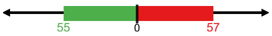
1495	KTN1	ENSG00000126777:56133959-56134048:target	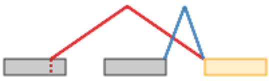	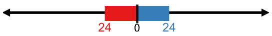
1496	CANX	ENSG00000127022:179135288-179135381:source	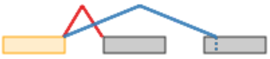	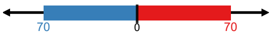
1497	CANX	ENSG00000127022:179136695-179137066:target	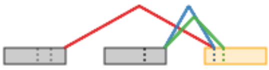	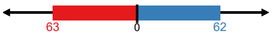
1498	RAP1B	ENSG00000127314:69004619-69004836:source	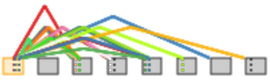	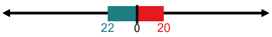
1499	TMEM175	ENSG00000127419:944209-944306:target	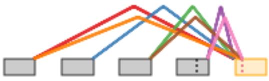	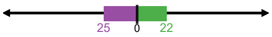
#	Gene	LSV ID	LSV Type	← More in Healthy | More in ALL →

#	Gene	LSV ID	LSV Type	← More in Healthy | More in ALL →
1500	UBR4	ENSG00000127481:19420443-19420633:source	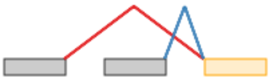	24 0 24
1501	UBR4	ENSG00000127481:19422039-19422416:target	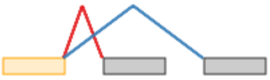	41 0 41
1502	UBR4	ENSG00000127481:19423017-19423144:source	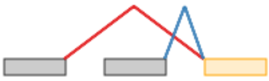	46 0 46
1503	UBR4	ENSG00000127481:19426091-19426171:target	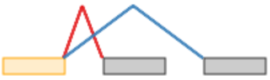	51 0 51
1504	UBR4	ENSG00000127481:19479795-19479996:source	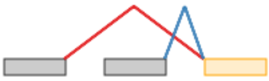	24 0 24
1505	UBR4	ENSG00000127481:19481412-19481572:target	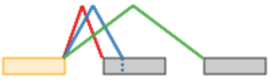	32 0 33
1506	UBR4	ENSG00000127481:19491743-19491843:source	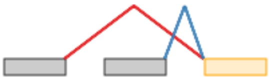	20 0 20
1507	UBR4	ENSG00000127481:19493516-19493725:target	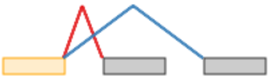	24 0 24
1508	SIN3B	ENSG00000127511:16973163-16973370:source	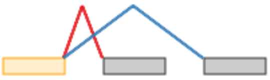	44 0 44
1509	MACF1	ENSG00000127603:39696854-39696915:target	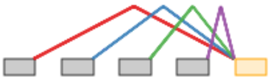	56 0 49
1510	MACF1	ENSG00000127603:39889700-39889866:source	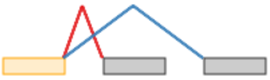	24 0 24
1511	AKAP9	ENSG00000127914:91669988-91671095:source	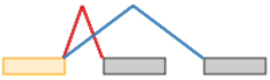	30 0 30
1512	AKAP9	ENSG00000127914:91671982-91672457:source	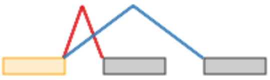	24 0 24
1513	AKAP9	ENSG00000127914:91671982-91672457:target	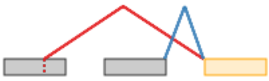	28 0 28
1514	RBM48	ENSG00000127993:92163716-92164284:source	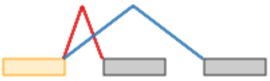	45 0 45
1515	TUBGCP6	ENSG00000128159:50658386-50658444:source	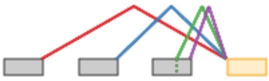	39 0 38
1516	TUBGCP6	ENSG00000128159:50660457-50660531:target	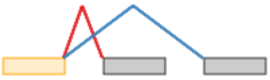	25 0 25
1517	PRKRIP1	ENSG00000128563:102039995-102040095:target	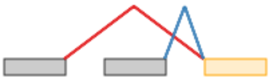	24 0 24
1518	MKLN1	ENSG00000128585:131082026-131082135:source	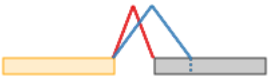	21 0 21
1519	NDUFA5	ENSG00000128609:123190159-123190640:target	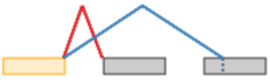	25 0 25
#	Gene	LSV ID	LSV Type	← More in Healthy | More in ALL →

#	Gene	LSV ID	LSV Type	← More in Healthy | More in ALL →
1520	ORMDL1	ENSG00000128699:190647148-190647328:source	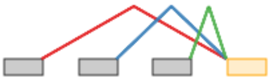	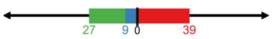
1521	WDFY4	ENSG00000128815:49984795-49985029:source	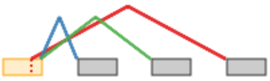	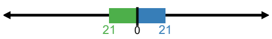
1522	WDFY4	ENSG00000128815:49986656-49986877:target	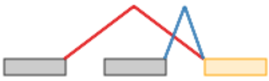	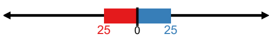
1523	WDFY4	ENSG00000128815:50015904-50016003:source	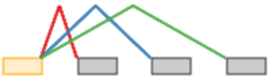	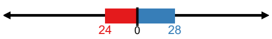
1524	INO80	ENSG00000128908:41313098-41313323:source	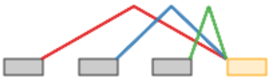	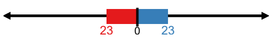
1525	INO80	ENSG00000128908:41337102-41337273:target	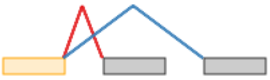	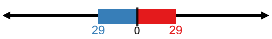
1526	VPS13C	ENSG00000129003:62160858-62160972:target	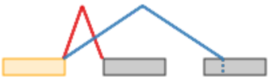	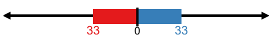
1527	VPS13C	ENSG00000129003:62211488-62211648:target	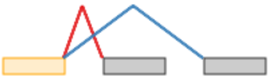	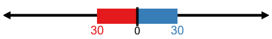
1528	PSMA1	ENSG00000129084:14540543-14540587:source	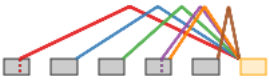	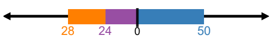
1529	PHF20L1	ENSG00000129292:133829584-133831215:target	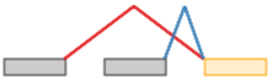	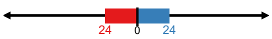
1530	PHF20L1	ENSG00000129292:133849957-133850061:source	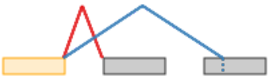	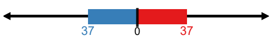
1531	PHF20L1	ENSG00000129292:133853798-133855120:target	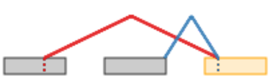	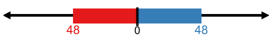
1532	CCNT1	ENSG00000129315:49089542-49089760:source	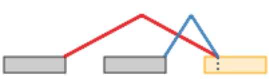	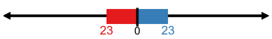
1533	ILF3	ENSG00000129351:10796604-10798790:source	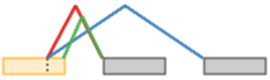	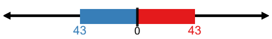
1534	ILF3	ENSG00000129351:10796604-10798790:target	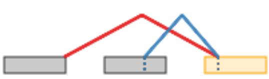	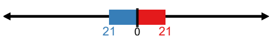
1535	ILF3	ENSG00000129351:10799855-10803093:target	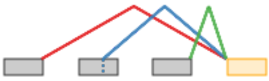	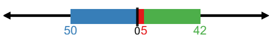
1536	SLC44A2	ENSG00000129353:10736929-10736977:target	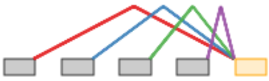	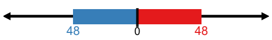
1537	SLC44A2	ENSG00000129353:10745432-10745563:source	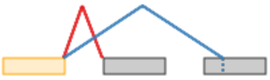	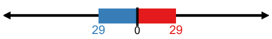
1538	EAPP	ENSG00000129518:35002650-35002745:target	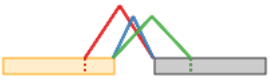	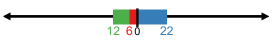
1539	EAPP	ENSG00000129518:35008462-35008916:target	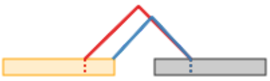	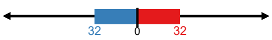

| # | Gene | LSV ID | LSV Type | ← More in Healthy \| More in ALL → |

#	Gene	LSV ID	LSV Type	← More in Healthy | More in ALL →
1540	TEP1	ENSG00000129566:20839648-20839791:source	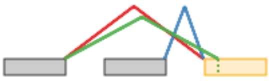	51 0 51
1541	TEP1	ENSG00000129566:20841170-20841306:target	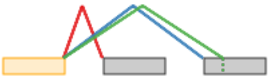	37 0 37
1542	SEC14L1	ENSG00000129657:75190759-75191113:source	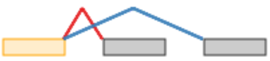	63 0 63
1543	SEC14L1	ENSG00000129657:75196566-75197204:source	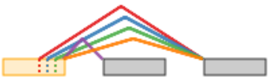	6 0 22
1544	SEC14L1	ENSG00000129657:75196566-75197204:target	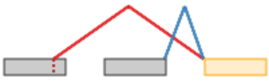	71 0 71
1545	SEC14L1	ENSG00000129657:75199652-75199740:target	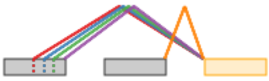	6 0 23
1546	PHF10	ENSG00000130024:170105712-170105820:source	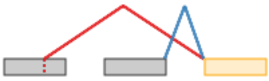	31 0 31
1547	PHF10	ENSG00000130024:170112482-170112635:source	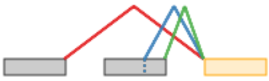	15 21 0 36
1548	XPO7	ENSG00000130227:21847855-21848122:source	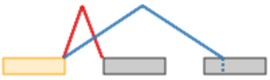	34 0 34
1549	XPO7	ENSG00000130227:21849237-21849485:target	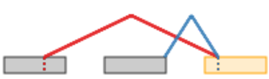	46 0 46
1550	COLGALT1	ENSG00000130309:17670120-17670230:source	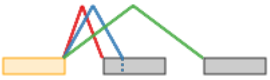	32 0 35
1551	COLGALT1	ENSG00000130309:17678215-17678349:target	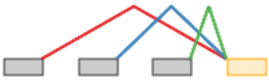	25 0 25
1552	COLGALT1	ENSG00000130309:17690760-17691714:target	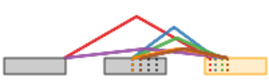	20 0 20
1553	PGLS	ENSG00000130313:17628097-17628198:target	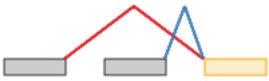	23 0 23
1554	LAMA5	ENSG00000130702:60883011-60883526:source	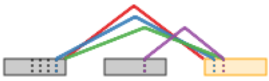	56 0 30 26
1555	ADRM1	ENSG00000130706:60883077-60883234:source	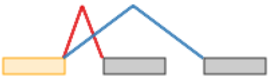	49 0 49
1556	ADRM1	ENSG00000130706:60883711-60883918:target	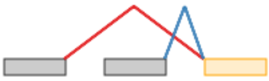	49 0 49
1557	PRRC2B	ENSG00000130723:134308004-134308181:source	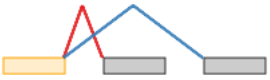	25 0 25
1558	PRRC2B	ENSG00000130723:134357113-134357195:source	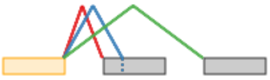	33 0 10 21
1559	PRRC2B	ENSG00000130723:134358097-134358294:target	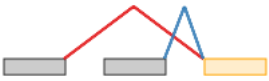	42 0 42

| # | Gene | LSV ID | LSV Type | ← More in Healthy \| More in ALL → |

#	Gene	LSV ID	LSV Type	← More in Healthy | More in ALL →
1560	PRRC2B	ENSG00000130723:134361472-134361569:source		21 0 21
1561	PRRC2B	ENSG00000130723:134362554-134362681:target		27 0 30
1562	PRRC2B	ENSG00000130723:134363243-134363483:source		49 0 51
1563	TRIM28	ENSG00000130726:59058502-59058878:target		23 0 29
1564	C16orf13	ENSG00000130731:684664-684797:source		22 0 8
1565	EIF2S3	ENSG00000130741:24094839-24096088:target		22 0 22
1566	THEMIS2	ENSG00000130775:28206155-28206565:target		20 0 20
1567	ZNF317	ENSG00000130803:9251056-9251268:source		25 0 25
1568	ZNF317	ENSG00000130803:9267944-9268386:source		24 0 25
1569	ZNF317	ENSG00000130803:9269502-9269584:target		30 0 30
1570	ZNF331	ENSG00000130844:54042431-54042778:source		34 0 34
1571	ZNF331	ENSG00000130844:54058932-54059162:target		12 20 0 26
1572	NOL11	ENSG00000130935:65732016-65732139:source		37 0 37
1573	NOL11	ENSG00000130935:65733155-65733261:target		35 0 32
1574	PPIL4	ENSG00000131013:149842196-149842307:target		22 0 22
1575	PPIL4	ENSG00000131013:149854594-149854710:target		21 0 21
1576	SYNE1	ENSG00000131018:152442819-152443811:source		28 0 28
1577	SYNE1	ENSG00000131018:152453257-152453349:target		24 0 24
1578	SYNE1	ENSG00000131018:152462338-152462464:source		20 0 20
1579	RBM39	ENSG00000131051:34326890-34327205:source		36 0 33

| # | Gene | LSV ID | LSV Type | ← More in Healthy \| More in ALL → |

#	Gene	LSV ID	LSV Type	← More in Healthy | More in ALL →
1580	RBM39	ENSG00000131051:34328728-34328911:target	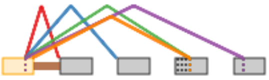	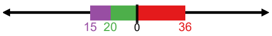
1581	TRAF3	ENSG00000131323:103342695-103343014:source	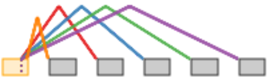	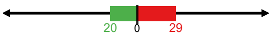
1582	TRAF3	ENSG00000131323:103352526-103352606:source	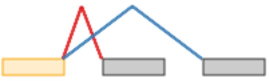	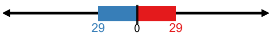
1583	TRAF3	ENSG00000131323:103363598-103363738:target	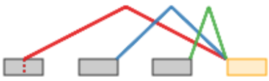	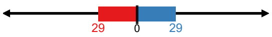
1584	SH3BP5	ENSG00000131370:15300053-15300557:source	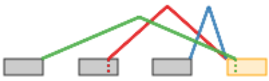	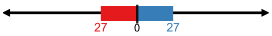
1585	SH3BP5	ENSG00000131370:15303665-15303796:target	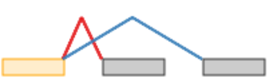	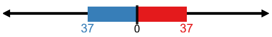
1586	TBC1D5	ENSG00000131374:18419662-18419817:target	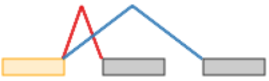	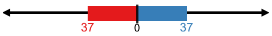
1587	CAPN7	ENSG00000131375:15283685-15283760:source	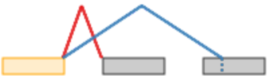	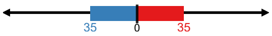
1588	CAPN7	ENSG00000131375:15283685-15283760:target	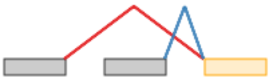	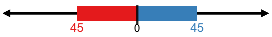
1589	CAPN7	ENSG00000131375:15287394-15288301:target	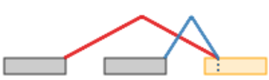	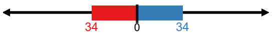
1590	RFTN1	ENSG00000131378:16475358-16475544:source	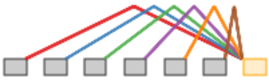	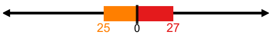
1591	RFTN1	ENSG00000131378:16535232-16535387:target	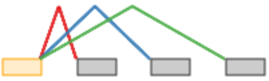	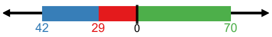
1592	PSME3	ENSG00000131467:40989666-40989772:source	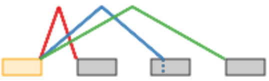	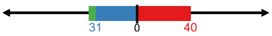
1593	PSME3	ENSG00000131467:40990709-40990816:target	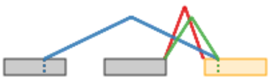	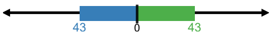
1594	RPL27	ENSG00000131469:41150494-41151296:target	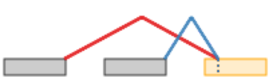	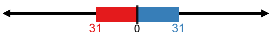
1595	ANKHD1	ENSG00000131503:139889604-139889766:source	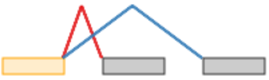	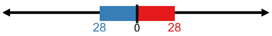
1596	ANKHD1	ENSG00000131503:139892948-139893064:target	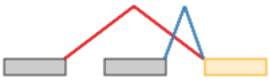	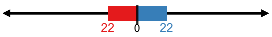
1597	ANKHD1	ENSG00000131503:139917671-139917847:source	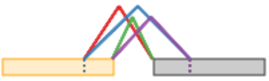	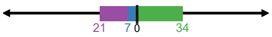
1598	DIAPH1	ENSG00000131504:140913902-140914055:target	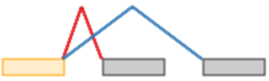	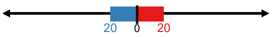
1599	UBE2D2	ENSG00000131508:138940751-138941400:source	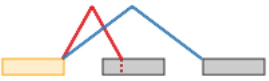	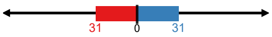
#	Gene	LSV ID	LSV Type	← More in Healthy | More in ALL →

#	Gene	LSV ID	LSV Type	← More in Healthy | More in ALL →
1600	UBE2D2	ENSG00000131508:138979957-138980020:target	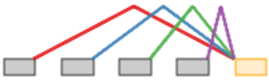	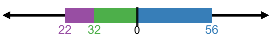
1601	CHD1L	ENSG00000131778:146740441-146740537:source	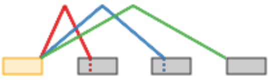	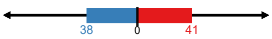
1602	CHD1L	ENSG00000131778:146743830-146744009:target	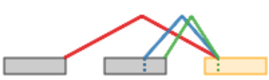	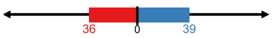
1603	CLUHP3	ENSG00000131797:31714477-31714688:target	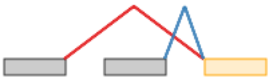	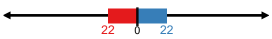
1604	SNRPA1	ENSG00000131876:101825931-101826006:source	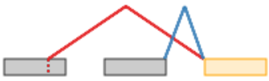	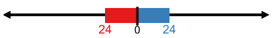
1605	SNRPA1	ENSG00000131876:101835302-101835487:target	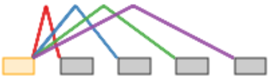	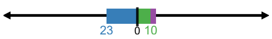
1606	ACTR10	ENSG00000131966:58675717-58675975:source	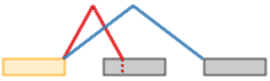	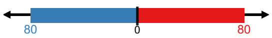
1607	ACTR10	ENSG00000131966:58681923-58682002:target	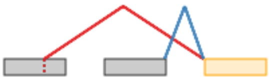	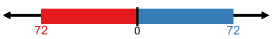
1608	RAF1	ENSG00000132155:12626621-12626905:source	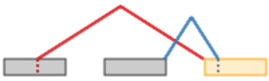	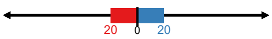
1609	NUP210	ENSG00000132182:13373793-13373884:source	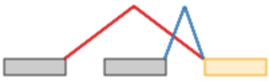	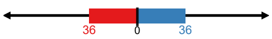
1610	NUP210	ENSG00000132182:13378287-13378529:target	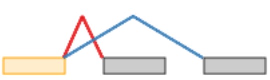	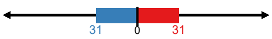
1611	NUP210	ENSG00000132182:13417004-13417141:target	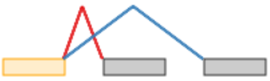	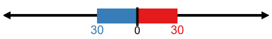
1612	EFR3A	ENSG00000132294:132966065-132966214:source	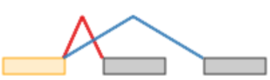	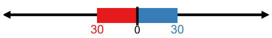
1613	EFR3A	ENSG00000132294:132968015-132968152:target	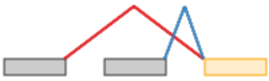	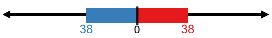
1614	EFR3A	ENSG00000132294:132980542-132980677:target	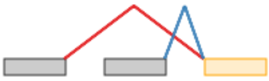	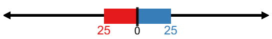
1615	EFR3A	ENSG00000132294:132999822-132999949:source	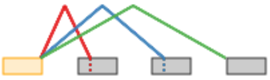	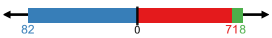
1616	EFR3A	ENSG00000132294:133014006-133014054:target	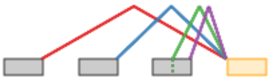	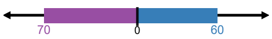
1617	ILKAP	ENSG00000132323:239090706-239090827:source	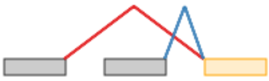	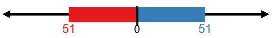
1618	ILKAP	ENSG00000132323:239092661-239092754:target	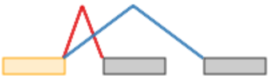	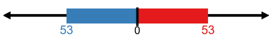
1619	ILKAP	ENSG00000132323:239103423-239103511:target	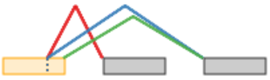	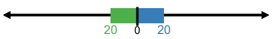
#	Gene	LSV ID	LSV Type	← More in Healthy | More in ALL →

#	Gene	LSV ID	LSV Type	← More in Healthy | More in ALL →
1620	UBE2G1	ENSG00000132388:4210316-4210418:target	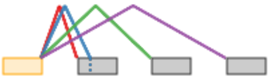	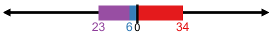
1621	PNISR	ENSG00000132424:99850416-99850586:source	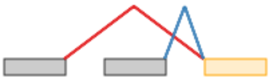	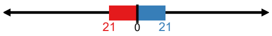
1622	PNISR	ENSG00000132424:99852479-99852578:target	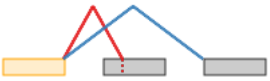	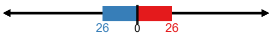
1623	UNK	ENSG00000132478:73789524-73790279:target	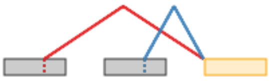	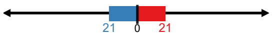
1624	ZRANB2	ENSG00000132485:71528974-71530820:source	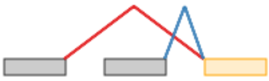	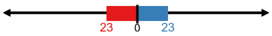
1625	KDM6B	ENSG00000132510:7749190-7749288:target	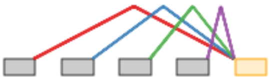	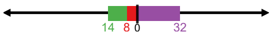
1626	KDM6B	ENSG00000132510:7754796-7754867:source	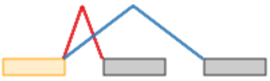	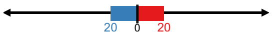
1627	FLOT2	ENSG00000132589:27209758-27210249:source	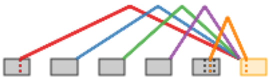	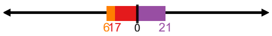
1628	TERF2	ENSG00000132604:69395011-69395392:source	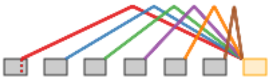	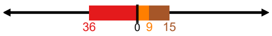
1629	PTPRA	ENSG00000132670:3008353-3008487:source	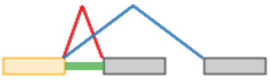	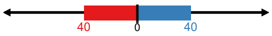
1630	PTPRA	ENSG00000132670:3016231-3016356:target	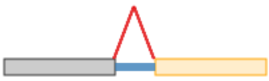	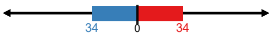
1631	KIAA0907	ENSG00000132680:155895423-155895634:source	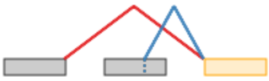	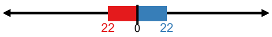
1632	KIAA0907	ENSG00000132680:155896895-155896947:target	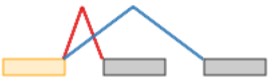	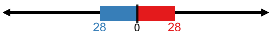
1633	DCAF8	ENSG00000132716:160188639-160189929:source	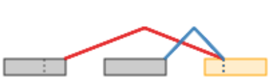	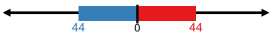
1634	DCAF8	ENSG00000132716:160194232-160194339:source	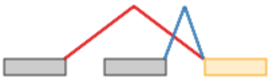	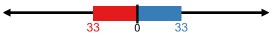
1635	DCAF8	ENSG00000132716:160195381-160195453:target	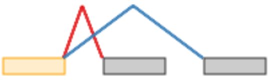	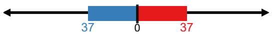
1636	NASP	ENSG00000132780:46056895-46056942:source	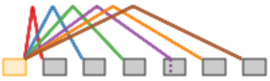	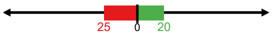
1637	NASP	ENSG00000132780:46067927-46068037:target	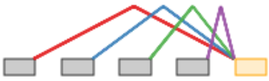	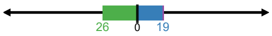
1638	SERINC3	ENSG00000132824:43132456-43132636:target	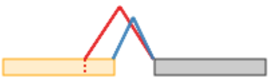	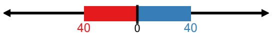
1639	CASP9	ENSG00000132906:15821768-15821947:target	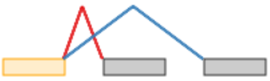	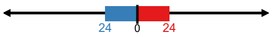

| # | Gene | LSV ID | LSV Type | ← More in Healthy \| More in ALL → |

#	Gene	LSV ID	LSV Type	← More in Healthy | More in ALL →
1640	CASP9	ENSG00000132906:15844605-15844890:target		40 0 41
1641	ZMYM5	ENSG00000132950:20425495-20425588:target		23 0 138
1642	USPL1	ENSG00000132952:31191830-31192193:source		21 0 20
1643	POMP	ENSG00000132963:29236547-29236644:target		74 0 74
1644	MPRIP	ENSG00000133030:16981325-16981390:source		31 0 31
1645	MPRIP	ENSG00000133030:17064554-17064670:source		28 0 28
1646	MPRIP	ENSG00000133030:17076012-17076129:source		24 0 24
1647	MPRIP	ENSG00000133030:17077230-17077389:source		30 0 30
1648	MPRIP	ENSG00000133030:17079740-17079826:target		20 14 0 9 25
1649	PIK3C2B	ENSG00000133056:204426165-204426198:source		26 0 26
1650	PIK3C2B	ENSG00000133056:204426165-204426198:target		33 0 33
1651	PIK3C2B	ENSG00000133056:204426856-204427002:target		25 0 25
1652	CNDP2	ENSG00000133313:72173084-72173263:source		41 0 49
1653	CNDP2	ENSG00000133313:72178048-72178468:target		57 0 57
1654	CNDP2	ENSG00000133313:72179683-72179767:target		36 0 36
1655	CNDP2	ENSG00000133313:72180812-72181554:source		15 816 0 39
1656	WDR74	ENSG00000133316:62600383-62600603:source		42 0 43
1657	WDR74	ENSG00000133316:62601264-62601409:target		47 0 47
1658	ATP13A3	ENSG00000133657:194165454-194165515:source		25 0 25
1659	ATP13A3	ENSG00000133657:194168581-194168738:target		24 0 24

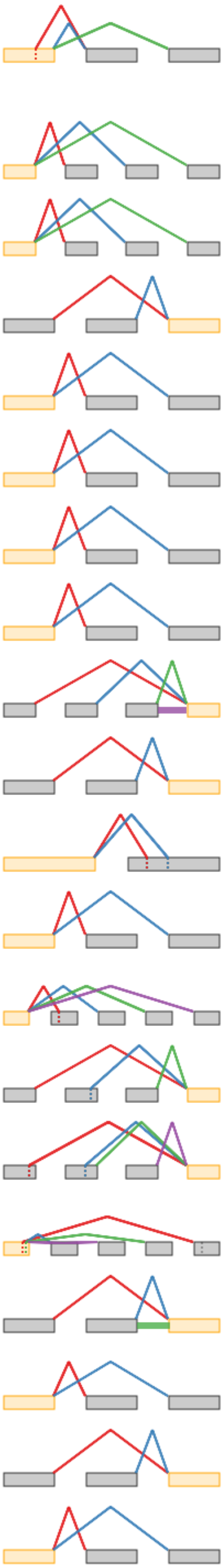

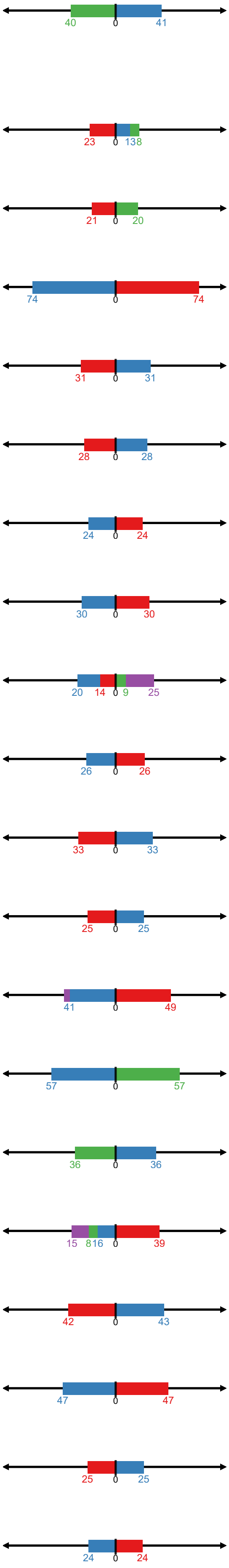

Gene LSV ID LSV Type ← More in Healthy | More in ALL →

#	Gene	LSV ID	LSV Type	← More in Healthy | More in ALL →
1660	KRAS	ENSG00000133703:25357723-25362845:source	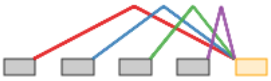	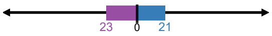
1661	KRAS	ENSG00000133703:25378548-25378707:target	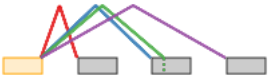	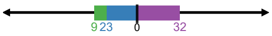
1662	CCDC59	ENSG00000133773:82750623-82751110:source	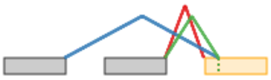	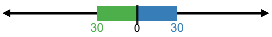
1663	HSD17B4	ENSG00000133835:118809603-118809710:target	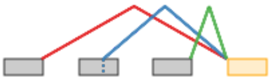	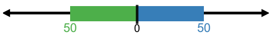
1664	HSD17B4	ENSG00000133835:118844836-118844939:source	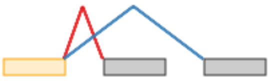	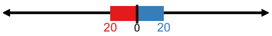
1665	HSD17B4	ENSG00000133835:118865589-118865795:source	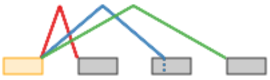	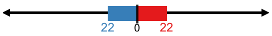
1666	ZFC3H1	ENSG00000133858:72005336-72005667:target	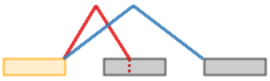	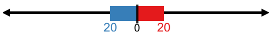
1667	ZFC3H1	ENSG00000133858:72020040-72020226:target	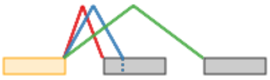	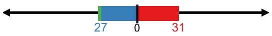
1668	NUMB	ENSG00000133961:73752901-73754022:target	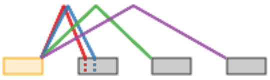	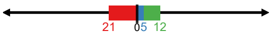
1669	VHL	ENSG00000134086:10191471-10193904:target	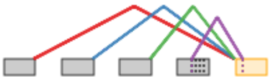	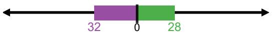
1670	EDEM1	ENSG00000134109:5249778-5249948:source	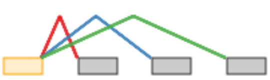	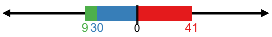
1671	EDEM1	ENSG00000134109:5252805-5253091:source	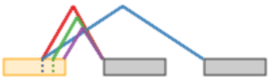	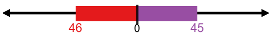
1672	EDEM1	ENSG00000134109:5252805-5253091:target	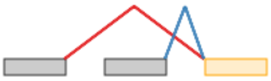	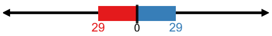
1673	EDEM1	ENSG00000134109:5255004-5255207:target	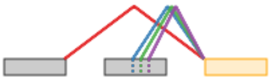	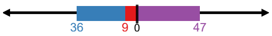
1674	EDEM1	ENSG00000134109:5257514-5261642:target	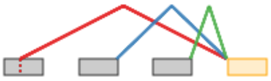	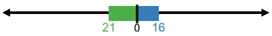
1675	PRPF38B	ENSG00000134186:109234945-109235489:source	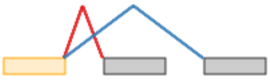	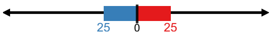
1676	PRPF38B	ENSG00000134186:109238324-109238402:source	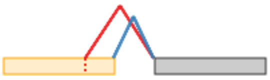	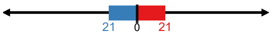
1677	PRPF38B	ENSG00000134186:109238324-109238402:target	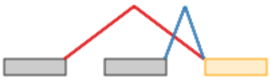	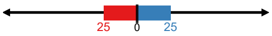
1678	PRPF38B	ENSG00000134186:109238899-109238973:source	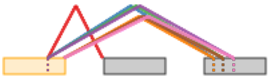	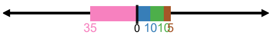
1679	PRPF38B	ENSG00000134186:109240322-109241449:target	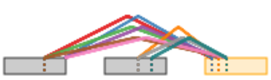	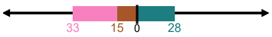

Gene LSV ID LSV Type ← More in Healthy | More in ALL →

#	Gene	LSV ID	LSV Type	← More in Healthy | More in ALL →
1680	LAMTOR5	ENSG00000134248:110948940-110949047:source	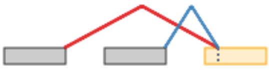	31 0 31
1681	CEPT1	ENSG00000134255:111717501-111717585:source	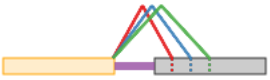	15 12 0 32
1682	CEPT1	ENSG00000134255:111717501-111717585:target	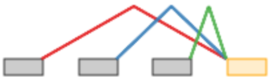	41 0 38
1683	CEPT1	ENSG00000134255:111720836-111724940:target	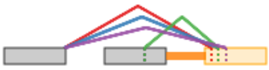	12 5 10 0 41
1684	AP4B1	ENSG00000134262:114442892-114443022:source	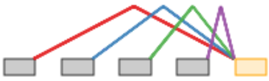	16 6 0 22
1685	PPHLN1	ENSG00000134283:42729685-42729776:source	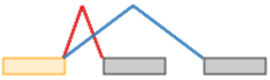	34 0 34
1686	PPHLN1	ENSG00000134283:42748963-42749480:target	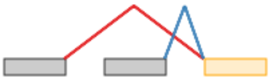	40 0 40
1687	FKBP11	ENSG00000134285:49318344-49318512:source	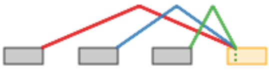	41 0 29 13
1688	SLC38A2	ENSG00000134294:46756277-46756687:source	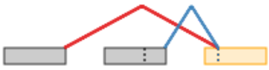	35 0 35
1689	SLC38A2	ENSG00000134294:46757509-46757609:target	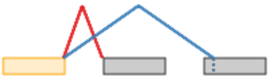	39 0 39
1690	LPIN1	ENSG00000134324:11913746-11913871:source	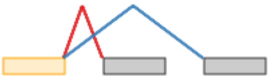	22 0 22
1691	LPIN1	ENSG00000134324:11955214-11955366:source	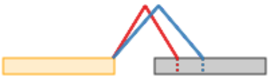	28 0 28
1692	LPIN1	ENSG00000134324:11956279-11959876:source	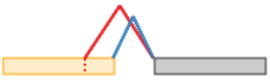	31 0 31
1693	RPS15A	ENSG00000134419:18799866-18800459:source	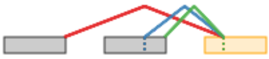	27 0 27
1694	FBXO18	ENSG00000134452:5952901-5953091:source	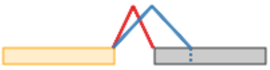	36 0 36
1695	FBXO18	ENSG00000134452:5963411-5963532:source	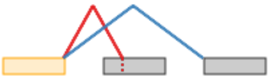	24 0 24
1696	FBXO18	ENSG00000134452:5966274-5966471:target	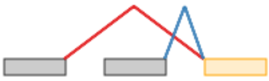	29 0 29
1697	FBXO18	ENSG00000134452:5967227-5967455:source	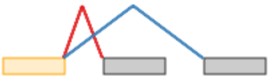	32 0 32
1698	FBXO18	ENSG00000134452:5969398-5969504:target	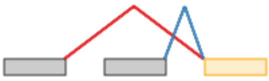	34 0 34
1699	CCNH	ENSG00000134480:86703855-86704003:source	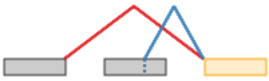	30 0 30
#	Gene	LSV ID	LSV Type	← More in Healthy | More in ALL →

#	Gene	LSV ID	LSV Type	← More in Healthy | More in ALL →
1700	CCNH	ENSG00000134480:86705107-86705180:target		26 0 26
1701	DOCK2	ENSG00000134516:169507167-169507287:source		30 0 30
1702	PUM1	ENSG00000134644:31438829-31439227:source		35 0 38
1703	PUM1	ENSG00000134644:31447496-31447706:source		54 0 54
1704	PUM1	ENSG00000134644:31454159-31454252:source		20 0 23
1705	PUM1	ENSG00000134644:31454159-31454252:target		52 0 27 27
1706	PUM1	ENSG00000134644:31465237-31465507:target		43 6 0 53
1707	YARS	ENSG00000134684:33244983-33245124:source		20 0 20
1708	YARS	ENSG00000134684:33245686-33246312:target		20 0 19
1709	YARS	ENSG00000134684:33248005-33249356:source		23 0 23
1710	YARS	ENSG00000134684:33282789-33283754:target		23 0 23
1711	ZCCHC11	ENSG00000134744:52912262-52912339:source		58 0 58
1712	ZCCHC11	ENSG00000134744:52926629-52926895:target		51 0 51
1713	ZCCHC11	ENSG00000134744:52954581-52954707:target		10 5 0 22
1714	ZCCHC11	ENSG00000134744:52956404-52956473:source		21 0 21
1715	ZCCHC11	ENSG00000134744:52959283-52959334:target		26 0 31
1716	ELP2	ENSG00000134759:33709834-33710034:source		38 0 38
1717	ELP2	ENSG00000134759:33716270-33716595:target		37 0 37
1718	ELP2	ENSG00000134759:33721100-33721164:source		27 0 27
1719	ELP2	ENSG00000134759:33721100-33721164:target		22 0 15
#	Gene	LSV ID	LSV Type	← More in Healthy | More in ALL →

#	Gene	LSV ID	LSV Type	← More in Healthy | More in ALL →
1720	ELP2	ENSG00000134759:33722789-33722929:source	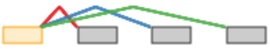	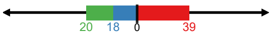
1721	ELP2	ENSG00000134759:33722789-33722929:target	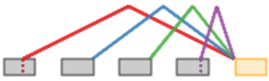	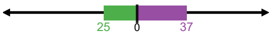
1722	ELP2	ENSG00000134759:33726213-33726344:target	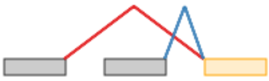	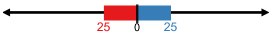
1723	UBAC2	ENSG00000134882:99896769-99896878:source	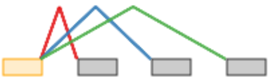	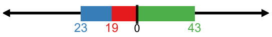
1724	ARGLU1	ENSG00000134884:107209396-107209479:source	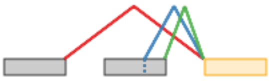	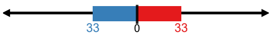
1725	ARGLU1	ENSG00000134884:107211780-107212005:target	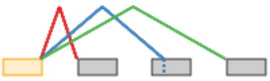	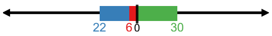
1726	TPP2	ENSG00000134900:103288574-103288742:source	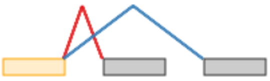	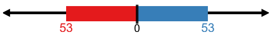
1727	TPP2	ENSG00000134900:103289432-103289589:target	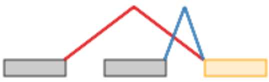	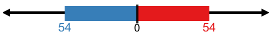
1728	TPP2	ENSG00000134900:103301758-103301836:target	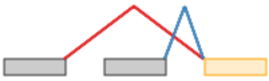	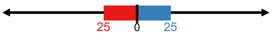
1729	CARS2	ENSG00000134905:111294858-111294868:source	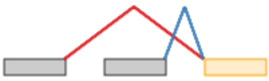	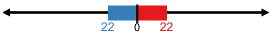
1730	CARS2	ENSG00000134905:111296732-111297980:target	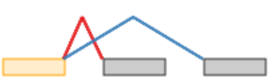	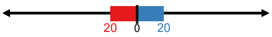
1731	NAA35	ENSG00000135040:88573387-88573501:source	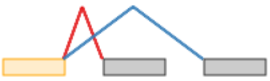	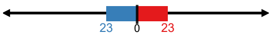
1732	TMEM2	ENSG00000135048:74300659-74302344:source	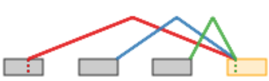	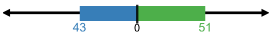
1733	TMEM2	ENSG00000135048:74309425-74309523:target	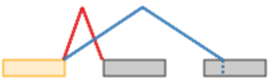	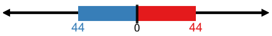
1734	TMEM2	ENSG00000135048:74359934-74360539:source	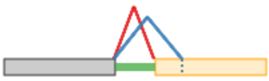	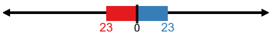
1735	TMEM2	ENSG00000135048:74361117-74361257:target	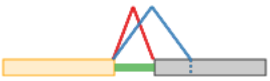	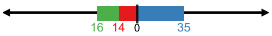
1736	TMEM2	ENSG00000135048:74364917-74365301:source	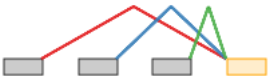	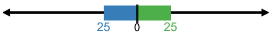
1737	TMEM2	ENSG00000135048:74364917-74365301:target	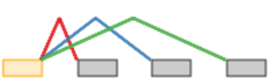	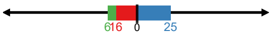
1738	DMTF1	ENSG00000135164:86792811-86792933:target	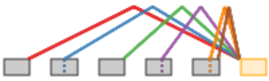	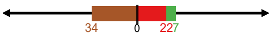
1739	DMTF1	ENSG00000135164:86810512-86810657:source	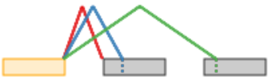	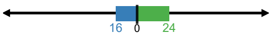

\# Gene LSV ID LSV Type ← More in Healthy | More in ALL →

#	Gene	LSV ID	LSV Type	← More in Healthy | More in ALL →
1740	DMTF1	ENSG00000135164:86815145-86815296:target	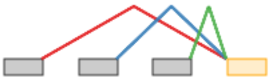	20 0 20
1741	PNPLA8	ENSG00000135241:108154880-108156018:source	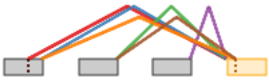	24 0 13
1742	RINT1	ENSG00000135249:105190502-105190612:source	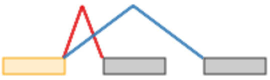	29 0 29
1743	RINT1	ENSG00000135249:105192017-105192154:target	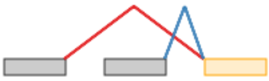	22 0 22
1744	SYNCRIP	ENSG00000135316:86346685-86346861:source	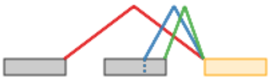	12 23 0 38
1745	SYNCRIP	ENSG00000135316:86349939-86350046:target	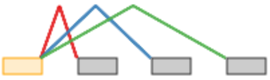	21 0 27
1746	SNX14	ENSG00000135317:86223518-86223613:target	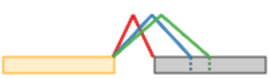	71 0 66
1747	SNX14	ENSG00000135317:86243317-86243518:target	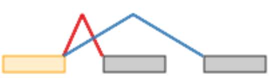	25 0 25
1748	PHF21A	ENSG00000135365:45950871-45955776:source	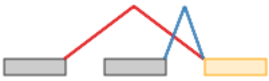	29 0 29
1749	PHF21A	ENSG00000135365:45957187-45957290:target	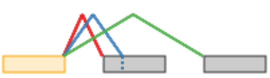	21 0 31
1750	PHF21A	ENSG00000135365:46105717-46105853:source	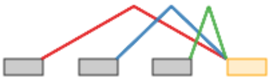	22 0 22
1751	PHF21A	ENSG00000135365:46113734-46113774:target	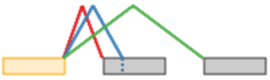	22 0 20
1752	CAPRIN1	ENSG00000135387:34112076-34112225:source	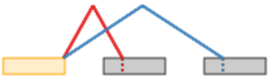	41 0 41
1753	CAPRIN1	ENSG00000135387:34117707-34118172:target	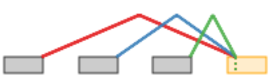	43 0 43
1754	CAPRIN1	ENSG00000135387:34118743-34118843:source	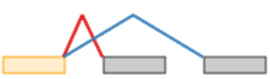	34 0 34
1755	HNRNPA1	ENSG00000135486:54678042-54678155:source	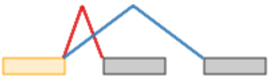	26 0 26
1756	OS9	ENSG00000135506:58112776-58112965:source	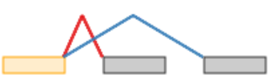	22 0 22
1757	CD164	ENSG00000135535:109689887-109690220:source	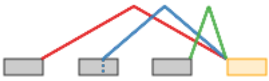	21 0 21
1758	CD164	ENSG00000135535:109697090-109697315:target	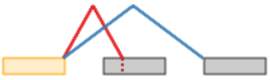	27 0 27
1759	CCT7	ENSG00000135624:73477434-73477566:source	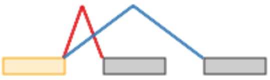	31 0 31

| # | Gene | LSV ID | LSV Type | ← More in Healthy \| More in ALL → |

#	Gene	LSV ID	LSV Type	← More in Healthy | More in ALL →
1760	CCT7	ENSG00000135624:73479768-73480149:target	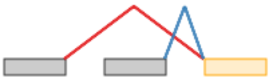	25 0 25
1761	DYNC1LI2	ENSG00000135720:66767876-66768214:target	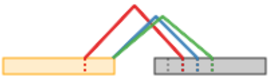	27 0 27
1762	CEP350	ENSG00000135837:179991833-179991983:source	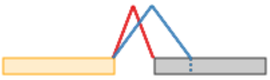	25 0 25
1763	RC3H1	ENSG00000135870:173939643-173939755:source	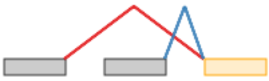	25 0 25
1764	RC3H1	ENSG00000135870:173947626-173947758:target	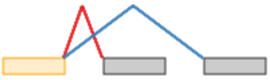	33 0 33
1765	SP110	ENSG00000135899:231032009-231033953:source	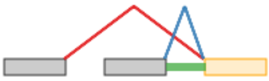	39 0 39
1766	SP110	ENSG00000135899:231036782-231037028:source	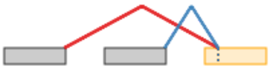	21 0 21
1767	DOCK10	ENSG00000135905:225637874-225638064:source	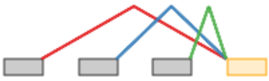	13 8 0 23
1768	DOCK10	ENSG00000135905:225659596-225659843:source	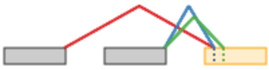	21 0 18
1769	GCC2	ENSG00000135968:109085436-109085540:source	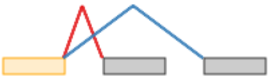	46 0 46
1770	GCC2	ENSG00000135968:109086107-109086683:target	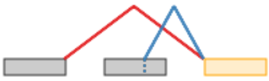	39 0 39
1771	GCC2	ENSG00000135968:109089283-109089784:source	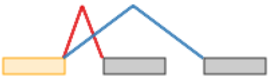	58 0 58
1772	GCC2	ENSG00000135968:109102891-109103104:source	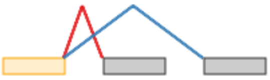	28 0 28
1773	GCC2	ENSG00000135968:109106294-109106591:target	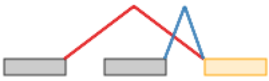	33 0 33
1774	EPC2	ENSG00000135999:149522524-149522715:source	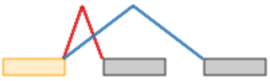	21 0 21
1775	ISCU	ENSG00000136003:108956358-108956512:source	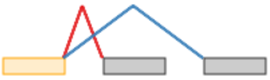	40 0 40
1776	ISCU	ENSG00000136003:108958055-108958168:target	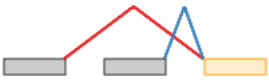	29 0 29
1777	KIAA1033	ENSG00000136051:105534945-105535024:source	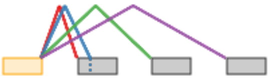	36 0 37
1778	KIAA1033	ENSG00000136051:105536905-105537021:target	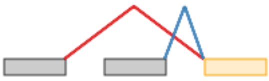	41 0 41
1779	KIAA1033	ENSG00000136051:105546121-105546229:source	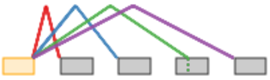	45 0 40
#	Gene	LSV ID	LSV Type	← More in Healthy | More in ALL →

#	Gene	LSV ID	LSV Type	← More in Healthy | More in ALL →
1780	KIAA1033	ENSG00000136051:105551014-105551100:target	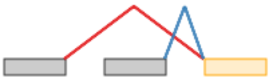	51 0 51
1781	FLNB	ENSG00000136068:58117805-58118658:target	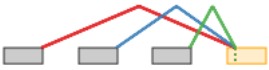	26 0 24
1782	VPS36	ENSG00000136100:53001322-53001354:source	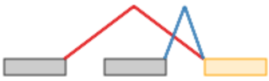	67 0 67
1783	RNASEH2B	ENSG00000136104:51509021-51509135:source	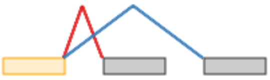	38 0 38
1784	RNASEH2B	ENSG00000136104:51523599-51523641:source	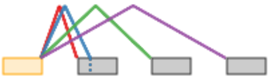	12 16 0 17 11
1785	RNASEH2B	ENSG00000136104:51528041-51528121:target	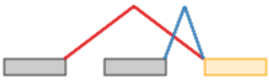	25 0 25
1786	PHF11	ENSG00000136147:50095030-50095169:source	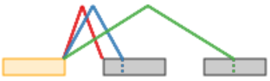	10 9 0 22
1787	ITM2B	ENSG00000136156:48827944-48828072:target	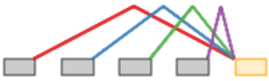	10 0 20
1788	LCP1	ENSG00000136167:46732657-46732786:source	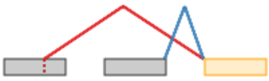	47 0 47
1789	LCP1	ENSG00000136167:46733151-46733821:target	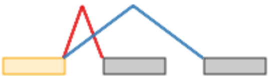	53 0 53
1790	SETDB2	ENSG00000136169:50050612-50051175:source	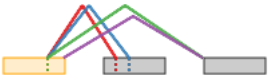	23 0 8
1791	IGF2BP3	ENSG00000136231:23358757-23358873:source	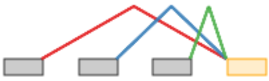	21 7 0 32
1792	TBRG4	ENSG00000136270:45144885-45147063:source	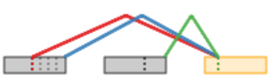	49 0 49
1793	CCM2	ENSG00000136280:45104202-45104245:source	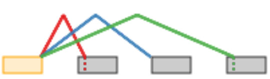	18 0 20
1794	CALCOCO2	ENSG00000136436:46928569-46929060:source	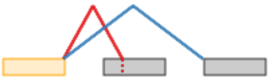	27 0 27
1795	CALCOCO2	ENSG00000136436:46930288-46930597:target	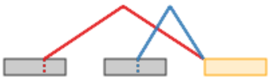	22 0 22
1796	SRSF1	ENSG00000136450:56081949-56082495:source	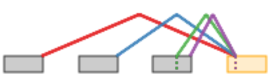	20 0 20
1797	SRSF1	ENSG00000136450:56083162-56083334:target	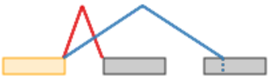	55 0 55
1798	TRA2B	ENSG00000136527:185638892-185638975:target	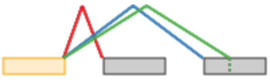	15 0 21
1799	TRA2B	ENSG00000136527:185644389-185646861:source	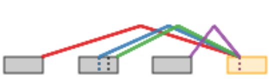	36 0 33

Gene LSV ID LSV Type ← More in Healthy | More in ALL →

#	Gene	LSV ID	LSV Type	← More in Healthy | More in ALL →
1800	TRA2B	ENSG00000136527:185655613-185655924:target	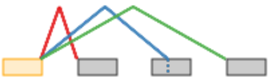	28 0 25
1801	TANK	ENSG00000136560:162059995-162060106:source	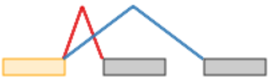	24 0 24
1802	TANK	ENSG00000136560:162061186-162061490:target	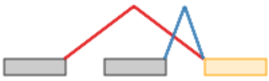	20 0 20
1803	TANK	ENSG00000136560:162081142-162081257:source	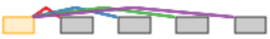	19 0 23
1804	TANK	ENSG00000136560:162087482-162087686:target	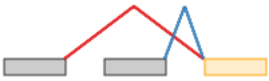	24 0 24
1805	BLK	ENSG00000136573:11403240-11403612:target	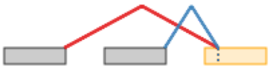	38 0 38
1806	ENSG00000136653	ENSG00000136653:206757719-206758016:source	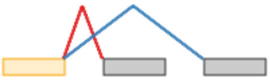	51 0 51
1807	ENSG00000136653	ENSG00000136653:206760158-206762616:target	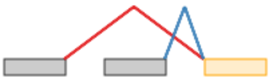	44 0 44
1808	WDR33	ENSG00000136709:128480827-128480921:source	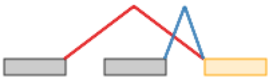	41 0 41
1809	GYPC	ENSG00000136732:127451440-127451523:target	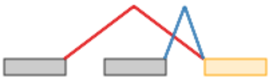	22 0 22
1810	ABI1	ENSG00000136754:27040527-27040712:source	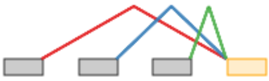	29 0 26
1811	ABI1	ENSG00000136754:27054147-27054247:target	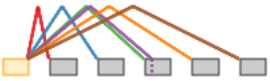	25 7 0 36
1812	ABI1	ENSG00000136754:27059174-27059274:source	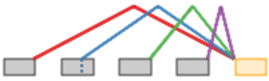	26 0 26
1813	ABI1	ENSG00000136754:27065555-27066170:target	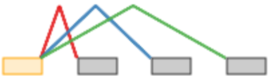	26 0 26
1814	YME1L1	ENSG00000136758:27434348-27434519:target	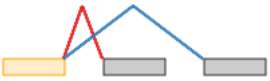	30 0 30
1815	CDK5RAP2	ENSG00000136861:123220728-123220900:source	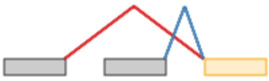	22 0 22
1816	TEX10	ENSG00000136891:103083965-103084053:target	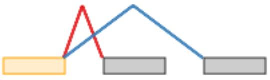	23 0 23
1817	TEX10	ENSG00000136891:103108976-103109688:target	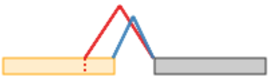	20 0 20
1818	DERL1	ENSG00000136986:124033687-124033739:target	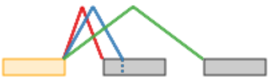	10 16 0 28
1819	RNF38	ENSG00000137075:36375931-36376124:source	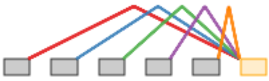	30 0 14 9

Gene LSV ID LSV Type ← More in Healthy | More in ALL →

#	Gene	LSV ID	LSV Type	← More in Healthy | More in ALL →
1820	TLN1	ENSG00000137076:35707069-35707250:target	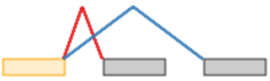	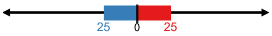
1821	CD72	ENSG00000137101:35616563-35616686:target	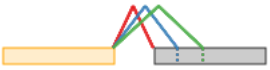	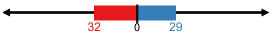
1822	CNPY3	ENSG00000137161:42902213-42902336:source	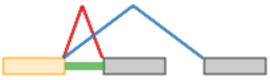	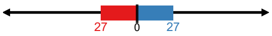
1823	CNPY3	ENSG00000137161:42903312-42903408:target	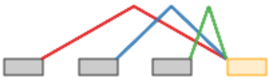	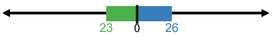
1824	CMTR1	ENSG00000137200:37429325-37429421:source	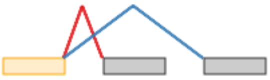	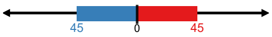
1825	CMTR1	ENSG00000137200:37430605-37430784:target	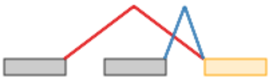	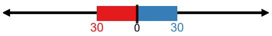
1826	IRF4	ENSG00000137265:397037-397546:source	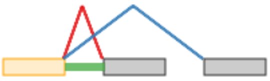	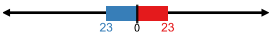
1827	IRF4	ENSG00000137265:401424-401777:target	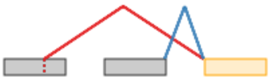	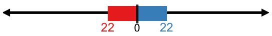
1828	MTCH1	ENSG00000137409:36946281-36946387:source	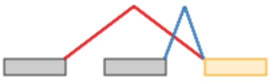	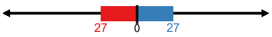
1829	MTCH1	ENSG00000137409:36953629-36954074:target	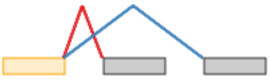	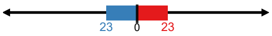
1830	FCHSD2	ENSG00000137478:72553999-72554373:target	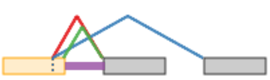	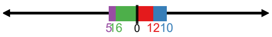
1831	CREBZF	ENSG00000137504:85372997-85373501:source	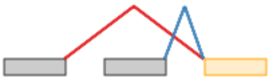	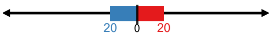
1832	CREBZF	ENSG00000137504:85374568-85374645:target	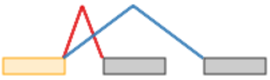	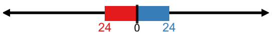
1833	ADAM10	ENSG00000137845:58925395-58925558:source	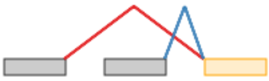	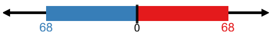
1834	ADAM10	ENSG00000137845:58932976-58933159:target	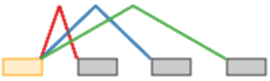	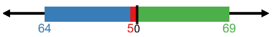
1835	RABGGTB	ENSG00000137955:76254844-76255041:source	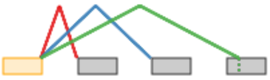	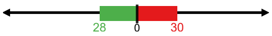
1836	RABGGTB	ENSG00000137955:76257146-76257544:source	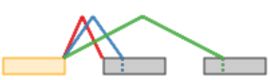	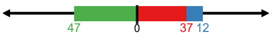
1837	RABGGTB	ENSG00000137955:76259067-76259918:target	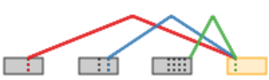	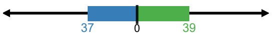
1838	HADHB	ENSG00000138029:26507751-26507825:source	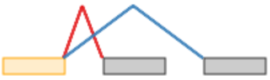	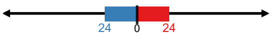
1839	HADHB	ENSG00000138029:26512786-26513336:target	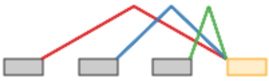	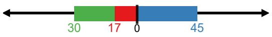

Gene LSV ID LSV Type ← More in Healthy | More in ALL →

#	Gene	LSV ID	LSV Type	← More in Healthy | More in ALL →
1840	ENSG00000138041	ENSG00000138041:55792038-55792177:source	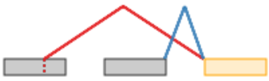	41 0 41
1841	ENSG00000138041	ENSG00000138041:55795165-55795499:target	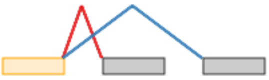	47 0 47
1842	ENSG00000138041	ENSG00000138041:55804451-55804492:source	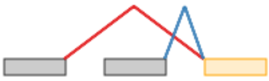	28 0 28
1843	ENSG00000138041	ENSG00000138041:55812187-55812303:source	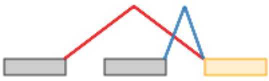	36 0 36
1844	ENSG00000138041	ENSG00000138041:55816015-55816092:source	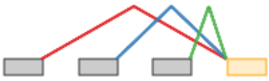	26 0 21
1845	ENSG00000138041	ENSG00000138041:55825552-55826175:target	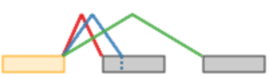	44 0 45
1846	SLC5A6	ENSG00000138074:27422973-27423447:source	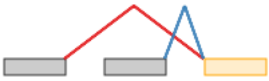	21 0 21
1847	SLC5A6	ENSG00000138074:27434335-27434401:source	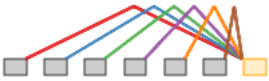	50 0 15 24
1848	FBXO11	ENSG00000138081:48033891-48035386:target	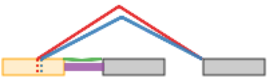	6 24 0 42
1849	LRPPRC	ENSG00000138095:44116873-44117015:source	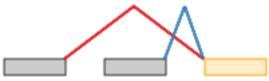	66 0 66
1850	LRPPRC	ENSG00000138095:44121684-44121768:target	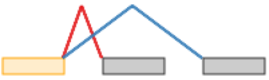	62 0 62
1851	LRPPRC	ENSG00000138095:44170826-44171033:source	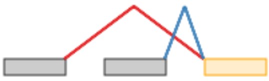	51 0 51
1852	LRPPRC	ENSG00000138095:44173252-44173382:target	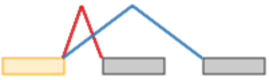	47 0 47
1853	ACTR1A	ENSG00000138107:104240889-104240929:source	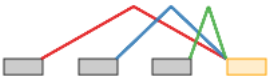	6 15 0 24
1854	ENTPD1	ENSG00000138185:97625934-97637023:target	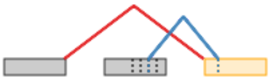	20 0 20
1855	DNAJC13	ENSG00000138246:132212988-132213103:source	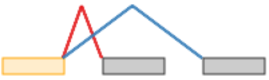	20 0 20
1856	ENSG00000138293	ENSG00000138293:51580556-51580696:source	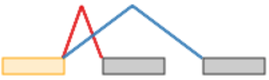	37 0 37
1857	ENSG00000138293	ENSG00000138293:51581270-51581378:target	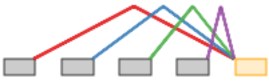	35 0 35
1858	SSB	ENSG00000138385:170664987-170665275:source	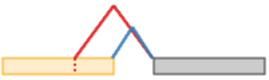	30 0 30
1859	SSFA2	ENSG00000138434:182784023-182784173:source	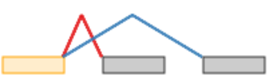	40 0 40
#	Gene	LSV ID	LSV Type	← More in Healthy | More in ALL →

#	Gene	LSV ID	LSV Type	← More in Healthy | More in ALL →
1860	SSFA2	ENSG00000138434:182786675-182787139:target		39 0 35
1861	SENP7	ENSG00000138468:101051614-101052113:source		25 0 25
1862	SENP7	ENSG00000138468:101058939-101059269:source		43 0 43
1863	SENP7	ENSG00000138468:101117705-101117899:source		41 0 38
1864	USP8	ENSG00000138592:50773678-50774262:source		24 0 26
1865	USP8	ENSG00000138592:50781691-50782078:target		21 0 21
1866	USP8	ENSG00000138592:50785257-50786477:source		26 0 31
1867	USP8	ENSG00000138592:50785257-50786477:target		26 41 0 70
1868	SPPL2A	ENSG00000138600:51014321-51014398:target		25 0 25
1869	SPPL2A	ENSG00000138600:51023162-51023236:source		34 0 34
1870	SPPL2A	ENSG00000138600:51028298-51028399:target		32 0 32
1871	ARHGAP24	ENSG00000138639:86896041-86896114:source		44 0 44
1872	HERC3	ENSG00000138641:89583582-89583706:target		24 0 24
1873	HERC3	ENSG00000138641:89591290-89591403:source		22 0 22
1874	HERC3	ENSG00000138641:89597485-89597574:target		21 0 21
1875	HERC3	ENSG00000138641:89607887-89607953:target		26 0 26
1876	SEC31A	ENSG00000138674:83778842-83779151:source		36 0 40
1877	SEC31A	ENSG00000138674:83784364-83784545:target		52 6 0 52
1878	SEC31A	ENSG00000138674:83799883-83800081:source		54 0 54
1879	SEC31A	ENSG00000138674:83801952-83802075:target		53 0 53

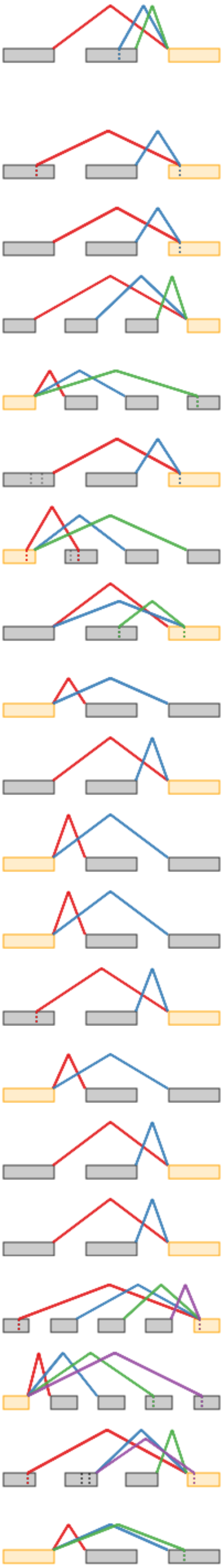

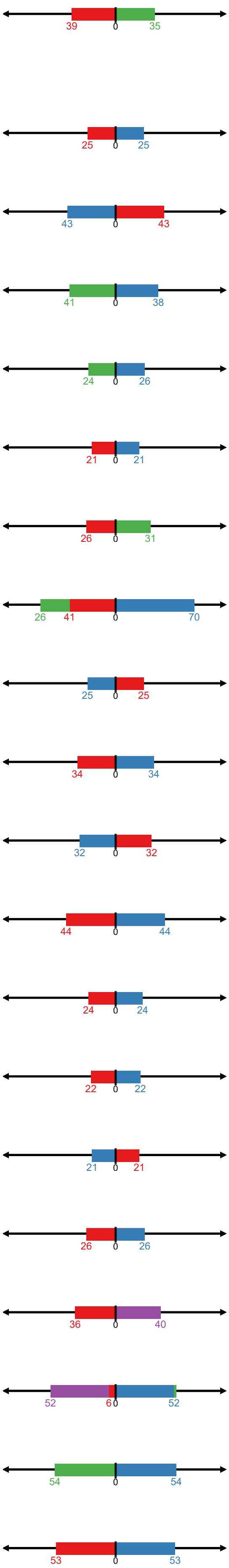

Gene LSV ID LSV Type ← More in Healthy | More in ALL →

#	Gene	LSV ID	LSV Type	← More in Healthy | More in ALL →
1880	SEC31A	ENSG00000138674:83819142-83819215:source	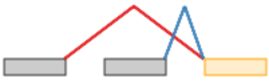	27 0 27
1881	SEC31A	ENSG00000138674:83821230-83821376:target	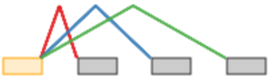	26 0 27
1882	KIAA1109	ENSG00000138688:123195498-123195591:source	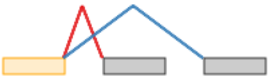	31 0 31
1883	KIAA1109	ENSG00000138688:123195498-123195591:target	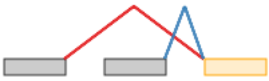	24 0 24
1884	KIAA1109	ENSG00000138688:123239302-123239425:source	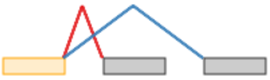	32 0 32
1885	KIAA1109	ENSG00000138688:123255499-123255700:source	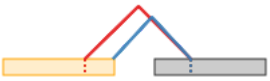	25 0 25
1886	BMP2K	ENSG00000138756:79763539-79763681:source	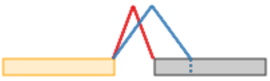	23 0 23
1887	BMP2K	ENSG00000138756:79799888-79801123:source	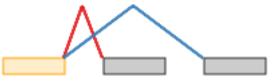	21 0 21
1888	G3BP2	ENSG00000138757:76567966-76570886:source	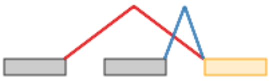	43 0 43
1889	G3BP2	ENSG00000138757:76572213-76572341:target	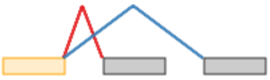	56 0 56
1890	G3BP2	ENSG00000138757:76582741-76582914:target	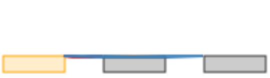	30 0 30
1891	CNOT6L	ENSG00000138767:78665872-78666029:target	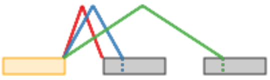	21 0 27
1892	SEC24B	ENSG00000138802:110394160-110394342:source	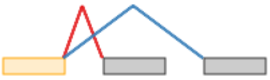	33 0 33
1893	PPP3CA	ENSG00000138814:101982244-101982318:source	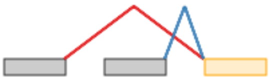	31 0 31
1894	PPP3CA	ENSG00000138814:102001689-102001783:target	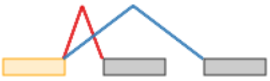	40 0 40
1895	PPP3CA	ENSG00000138814:102117073-102117273:target	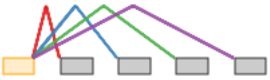	27 0 29
1896	MAPK8IP3	ENSG00000138834:1818200-1818379:target	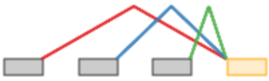	710 0 20
1897	GUCD1	ENSG00000138867:24944885-24944969:source	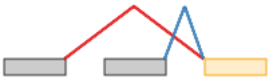	38 0 38
1898	PARVG	ENSG00000138964:44581406-44581994:source	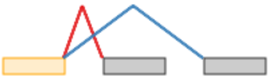	60 0 60
1899	ETV6	ENSG00000139083:11905384-11905809:source	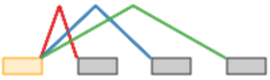	11 17 0 32
#	Gene	LSV ID	LSV Type	← More in Healthy | More in ALL →

#	Gene	LSV ID	LSV Type	← More in Healthy | More in ALL →
1900	FAM60A	ENSG00000139146:31451011-31451455:source		27 0 1610
1901	AEBP2	ENSG00000139154:19615444-19615651:source		52 0 52
1902	AEBP2	ENSG00000139154:19626182-19626289:target		44 0 44
1903	ETNK1	ENSG00000139163:22826434-22826594:source		55 0 55
1904	SCAF11	ENSG00000139218:46316141-46316365:source		21 0 20
1905	SCAF11	ENSG00000139218:46316141-46316365:target		55 0 55
1906	SCAF11	ENSG00000139218:46318511-46318571:target		25 0 25
1907	SCAF11	ENSG00000139218:46345433-46345535:source		27 0 31
1908	SCAF11	ENSG00000139218:46357890-46357971:source		13 26 0 36
1909	SCAF11	ENSG00000139218:46357890-46357971:target		26 0 27
1910	SCAF11	ENSG00000139218:46384136-46384367:target		29 0 31
1911	SLC15A4	ENSG00000139370:129293941-129294018:source		23 0 23
1912	GIT2	ENSG00000139436:110388499-110389121:target		20 0 20
1913	TCHP	ENSG00000139437:110388972-110389121:target		20 0 20
1914	NUPL1	ENSG00000139496:25875662-25876018:source		80 0 77
1915	NUPL1	ENSG00000139496:25881944-25882275:target		59 0 62
1916	NUPL1	ENSG00000139496:25887021-25887170:target		31 0 29
1917	NUPL1	ENSG00000139496:25889495-25889605:source		50 0 50
1918	NUPL1	ENSG00000139496:25894411-25894733:target		51 0 51
1919	NUPL1	ENSG00000139496:25905495-25905696:source		1032 0 43

| # | Gene | LSV ID | LSV Type | ← More in Healthy \| More in ALL → |

#	Gene	LSV ID	LSV Type	← More in Healthy | More in ALL →
1920	SMARCC2	ENSG00000139613:56565055-56565216:source		26 0 26
1921	SMARCC2	ENSG00000139613:56566023-56566274:target		26 0 26
1922	ITGB7	ENSG00000139626:53586114-53586322:source		14 6 0 27
1923	ITGB7	ENSG00000139626:53594027-53594230:source		21 0 5 10
1924	LMBR1L	ENSG00000139636:49491110-49491522:source		32 0 32
1925	LMBR1L	ENSG00000139636:49494171-49494237:target		31 0 31
1926	LMBR1L	ENSG00000139636:49496251-49496323:source		34 0 34
1927	LMBR1L	ENSG00000139636:49496864-49496942:target		33 0 33
1928	LMBR1L	ENSG00000139636:49498231-49498334:source		43 0 42
1929	LMBR1L	ENSG00000139636:49499707-49499863:target		56 0 54
1930	TMBIM6	ENSG00000139644:50146239-50146332:target		43 0 30 9
1931	WDFY2	ENSG00000139668:52301814-52301926:source		30 0 30
1932	WDFY2	ENSG00000139668:52325446-52325551:source		60 0 60
1933	WDFY2	ENSG00000139668:52325446-52325551:target		30 0 30
1934	VPS37B	ENSG00000139722:123380499-123380991:target		17 0 28
1935	CUL4A	ENSG00000139842:113891137-113891204:source		26 0 26
1936	CUL4A	ENSG00000139842:113897282-113897474:target		21 0 21
1937	C14orf37	ENSG00000139971:58675717-58675825:source		80 0 80
1938	C14orf37	ENSG00000139971:58681923-58682002:target		72 0 72
1939	C14orf37	ENSG00000139971:58764673-58764857:target		26 0 26

| # | Gene | LSV ID | LSV Type | ← More in Healthy \| More in ALL → |

#	Gene	LSV ID	LSV Type	← More in Healthy | More in ALL →
1940	ESR2	ENSG00000140009:64689907-64690098:target		26 0 26
1941	NIPA2	ENSG00000140157:23012279-23012439:source		27 0 31
1942	NIPA2	ENSG00000140157:23019800-23019856:target		37 0 37
1943	NIPA2	ENSG00000140157:23021392-23021429:source		45 21 0 11 18 34
1944	NIPA2	ENSG00000140157:23032797-23033413:target		14 7 0 27
1945	SLC12A6	ENSG00000140199:34533264-34533538:source		33 0 33
1946	SLC12A6	ENSG00000140199:34536175-34536273:target		39 0 39
1947	TCF12	ENSG00000140262:57545460-57545666:source		33 0 33
1948	SERF2	ENSG00000140264:44085037-44085620:source		1010 0 23
1949	SERF2	ENSG00000140264:44092621-44092982:source		26 0 26
1950	SERF2	ENSG00000140264:44093716-44093767:target		28 0 28
1951	TLE3	ENSG00000140332:70347388-70347637:target		25 0 26
1952	TLE3	ENSG00000140332:70352869-70352988:source		12 18 0 30
1953	ANP32A	ENSG00000140350:69076610-69076934:source		45 0 49
1954	ANP32A	ENSG00000140350:69080109-69080258:target		43 0 43
1955	TSPAN3	ENSG00000140391:77363234-77363570:target		15 0 22
1956	NCOA2	ENSG00000140396:71060508-71060718:target		21 0 21
1957	NEIL1	ENSG00000140398:75647305-75647592:target		24 0 24
1958	MCTP2	ENSG00000140563:94888358-94888393:source		23 0 23
1959	MCTP2	ENSG00000140563:94899203-94899530:target		34 0 34

Gene LSV ID LSV Type ← More in Healthy | More in ALL →

#	Gene	LSV ID	LSV Type	← More in Healthy | More in ALL →
1960	MCTP2	ENSG00000140563:94913316-94913409:source	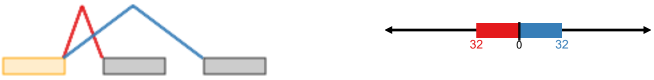	32 0 32
1961	MCTP2	ENSG00000140563:94927251-94927353:target	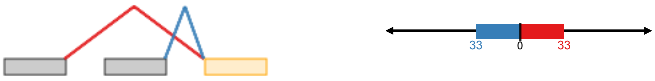	33 0 33
1962	IQGAP1	ENSG00000140575:91009233-91009323:source	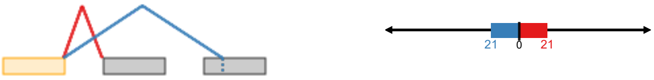	21 0 21
1963	IQGAP1	ENSG00000140575:91016780-91017030:target	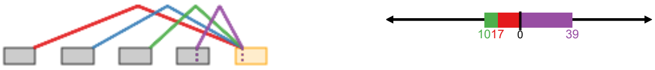	10 17 0 39
1964	IQGAP1	ENSG00000140575:91026437-91026825:target	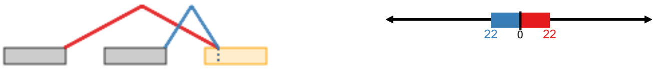	22 0 22
1965	GLYR1	ENSG00000140632:4861624-4861803:source	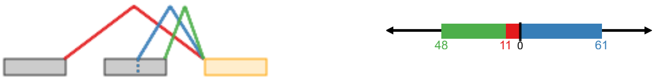	48 11 0 61
1966	GLYR1	ENSG00000140632:4861890-4862249:target	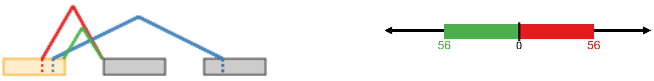	56 0 56
1967	GLYR1	ENSG00000140632:4896175-4896211:source	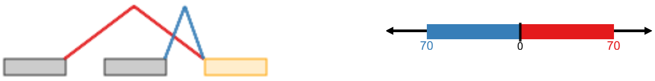	70 0 70
1968	UQCRC2	ENSG00000140740:21969856-21969920:source	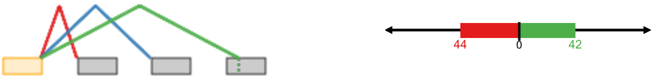	44 0 42
1969	UQCRC2	ENSG00000140740:21973513-21973837:target	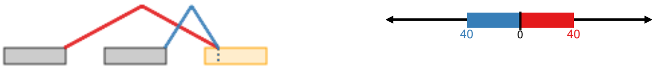	40 0 40
1970	ARHGAP17	ENSG00000140750:24960725-24960805:source	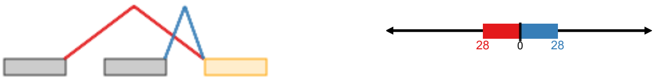	28 0 28
1971	ARHGAP17	ENSG00000140750:24964252-24964363:target	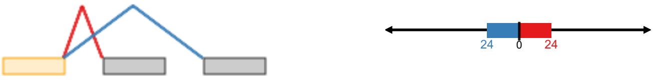	24 0 24
1972	MBTPS1	ENSG00000140943:84089610-84091649:source	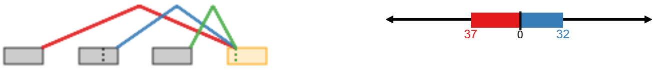	37 0 32
1973	MBTPS1	ENSG00000140943:84096910-84097050:target	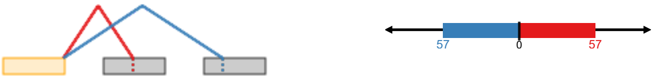	57 0 57
1974	MBTPS1	ENSG00000140943:84115137-84115774:source	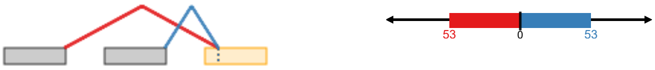	53 0 53
1975	MBTPS1	ENSG00000140943:84120963-84121065:target	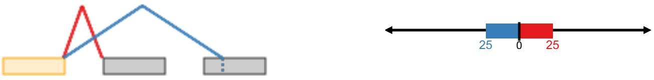	25 0 25
1976	MBTPS1	ENSG00000140943:84125337-84125453:source	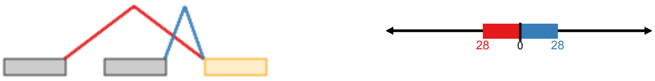	28 0 28
1977	MBTPS1	ENSG00000140943:84127316-84127426:target	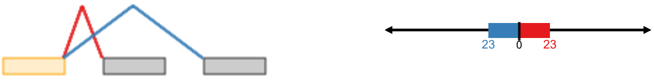	23 0 23
1978	IRF8	ENSG00000140968:85942594-85943960:source	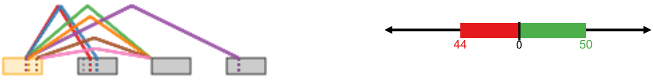	44 0 50
1979	IRF8	ENSG00000140968:85942594-85943960:target	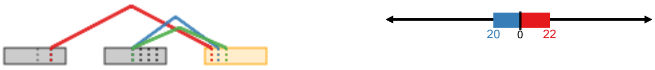	20 0 22
#	Gene	LSV ID	LSV Type	← More in Healthy | More in ALL →

#	Gene	LSV ID	LSV Type	← More in Healthy | More in ALL →
1980	IRF8	ENSG00000140968:85954712-85956215:target		48 0 48
1981	NCOR1	ENSG00000141027:16052765-16052831:source		22 0 22
1982	RANBP10	ENSG00000141084:67771916-67771938:source		34 0 31
1983	RANBP10	ENSG00000141084:67778048-67778358:source		35 0 38
1984	NPEPPS	ENSG00000141279:45646783-45646860:source		21 0 22
1985	NPEPPS	ENSG00000141279:45656756-45656877:target		31 0 31
1986	NPEPPS	ENSG00000141279:45668083-45668247:source		61 12 0 74
1987	NPEPPS	ENSG00000141279:45669851-45670046:target		37 0 37
1988	SSH2	ENSG00000141298:27974712-27975362:target		19 0 19
1989	SSH2	ENSG00000141298:28002848-28003903:target		25 0 25
1990	CLTC	ENSG00000141367:57761236-57761870:source		27 0 27
1991	CLTC	ENSG00000141367:57762948-57763169:target		27 0 27
1992	PTRH2	ENSG00000141378:57761236-57761346:source		27 0 27
1993	PTRH2	ENSG00000141378:57762948-57763169:target		27 0 27
1994	ARRB2	ENSG00000141480:4621183-4621444:source		26 0 26
1995	ARRB2	ENSG00000141480:4621907-4621979:target		24 0 24
1996	TMC6	ENSG00000141524:76115052-76115127:target		20 0 28
1997	EIF4A3	ENSG00000141543:78115494-78115647:target		22 0 22
1998	CSNK1D	ENSG00000141551:80200543-80202707:source		22 0 22
1999	CSNK1D	ENSG00000141551:80206751-80207040:target		29 0 25
#	Gene	LSV ID	LSV Type	← More in Healthy | More in ALL →

#	Gene	LSV ID	LSV Type	← More in Healthy | More in ALL →
2000	CSNK1D	ENSG00000141551:80223176-80223672:target	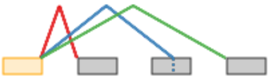	18 0 20
2001	DYM	ENSG00000141627:46858234-46858376:target	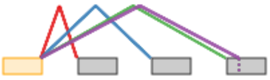	27 0 22
2002	SMAD4	ENSG00000141646:48575665-48575935:source	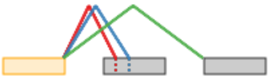	6 26 0 34
2003	SMAD4	ENSG00000141646:48581151-48581363:target	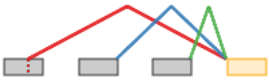	20 0 16
2004	SMAD4	ENSG00000141646:48603008-48603500:source	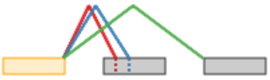	28 0 27
2005	TMEM50B	ENSG00000142188:34804792-34805178:source	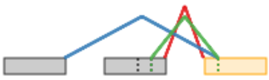	23 0 16 5
2006	EMP3	ENSG00000142227:48828582-48828867:source	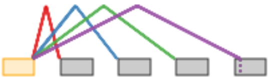	6 20 0 26
2007	ERVK3-1	ENSG00000142396:58817454-58818000:source	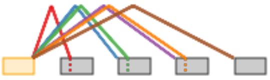	29 0 32
2008	RERE	ENSG00000142599:8420172-8421550:target	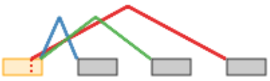	21 0 21
2009	KIAA0319L	ENSG00000142687:35904143-35906739:source	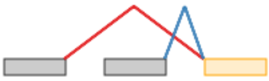	44 0 44
2010	KIAA0319L	ENSG00000142687:35908507-35908856:target	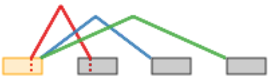	47 0 13 33
2011	SYTL1	ENSG00000142765:27675889-27675989:source	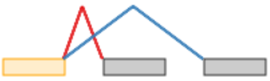	36 0 36
2012	SYTL1	ENSG00000142765:27676880-27676976:target	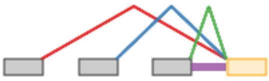	40 0 39
2013	WDTC1	ENSG00000142784:27618706-27618888:source	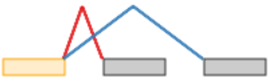	39 0 39
2014	MTF2	ENSG00000143033:93584890-93585831:source	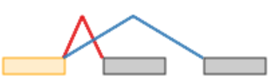	67 0 67
2015	MTF2	ENSG00000143033:93592789-93592856:target	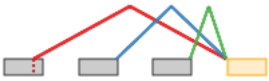	68 0 67
2016	PSMA5	ENSG00000143106:109941653-109944712:source	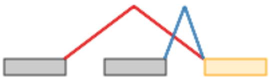	63 0 63
2017	PSMA5	ENSG00000143106:109952550-109952636:source	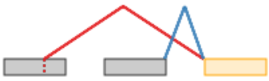	26 0 26
2018	PSMA5	ENSG00000143106:109952550-109952636:target	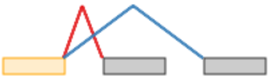	64 0 64
2019	PSMA5	ENSG00000143106:109954575-109954907:source	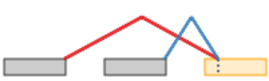	44 0 44

Gene LSV ID LSV Type ← More in Healthy | More in ALL →

#	Gene	LSV ID	LSV Type	← More in Healthy | More in ALL →
2020	PSMA5	ENSG00000143106:109957859-109957987:target		52 0 52
2021	CD53	ENSG00000143119:111415775-111415870:source		21 0 26
2022	CD53	ENSG00000143119:111434014-111434093:source		21 0 19
2023	CD53	ENSG00000143119:111434014-111434093:target		25 0 25
2024	CD53	ENSG00000143119:111434967-111435477:source		29 0 29
2025	CD53	ENSG00000143119:111437582-111437677:source		33 0 31
2026	CD53	ENSG00000143119:111441746-111442550:target		6 20 27 0 5 51
2027	RFWD2	ENSG00000143207:176104146-176104222:source		20 0 20
2028	PRCC	ENSG00000143294:156756400-156756966:source		34 0 25
2029	PRCC	ENSG00000143294:156761168-156761584:target		34 0 37
2030	SETDB1	ENSG00000143379:150900180-150900705:target		20 0 13
2031	MCL1	ENSG00000143384:150551319-150552066:target		30 0 30
2032	RFX5	ENSG00000143390:151317438-151317664:source		24 0 24
2033	RFX5	ENSG00000143390:151318681-151318809:target		20 0 20
2034	RFX5	ENSG00000143390:151318898-151319082:source		39 0 32 5
2035	PI4KB	ENSG00000143393:151288049-151288985:source		35 0 32
2036	PI4KB	ENSG00000143393:151299747-151300191:target		42 0 38
2037	PIP5K1A	ENSG00000143398:151196356-151196755:source		65 0 65
2038	PIP5K1A	ENSG00000143398:151199743-151199876:target		67 0 67
2039	ENSA	ENSG00000143420:150595757-150598284:source		31 0 35

| # | Gene | LSV ID | LSV Type | ← More in Healthy \| More in ALL → |

#	Gene	LSV ID	LSV Type	← More in Healthy | More in ALL →
2040	ENSA	ENSG00000143420:150599500-150600068:target		32 0 34
2041	POGZ	ENSG00000143442:151398665-151400517:target		20 0 20
2042	POGZ	ENSG00000143442:151400599-151400889:source		49 0 49
2043	POGZ	ENSG00000143442:151403142-151403317:target		43 0 47
2044	EIF2D	ENSG00000143486:206757719-206758016:source		51 0 51
2045	TP53BP2	ENSG00000143514:223971817-223972016:source		26 0 26
2046	TP53BP2	ENSG00000143514:223980091-223980224:target		27 0 27
2047	JTB	ENSG00000143543:153948293-153948381:source		30 0 30
2048	JTB	ENSG00000143543:153949452-153949489:target		49 0 49
2049	TPM3	ENSG00000143549:154131085-154131524:source		22 0 22
2050	UBAP2L	ENSG00000143569:154201091-154201201:source		30 0 30
2051	UBAP2L	ENSG00000143569:154207672-154207770:target		28 0 20 6
2052	UBAP2L	ENSG00000143569:154209042-154209087:source		21 0 21
2053	UBAP2L	ENSG00000143569:154213989-154214041:source		24 0 24
2054	UBAP2L	ENSG00000143569:154215406-154215778:target		30 0 30
2055	UBAP2L	ENSG00000143569:154234068-154234175:source		30 0 30
2056	C1orf43	ENSG00000143612:154184933-154185206:source		21 0 21
2057	C1orf43	ENSG00000143612:154186933-154187050:source		43 0 42
2058	C1orf43	ENSG00000143612:154186933-154187050:target		25 0 25
2059	C1orf43	ENSG00000143612:154192818-154193104:target		42 0 39

Gene LSV ID LSV Type ← More in Healthy | More in ALL →

#	Gene	LSV ID	LSV Type	← More in Healthy | More in ALL →
2060	GATAD2B	ENSG00000143614:153800489-153800824:source	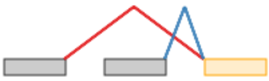	23 0 23
2061	GATAD2B	ENSG00000143614:153895209-153895451:target	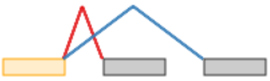	22 0 22
2062	ILF2	ENSG00000143621:153635691-153635752:target	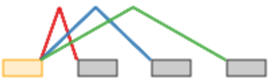	7 12 0 22
2063	LYST	ENSG00000143669:235892896-235892957:target	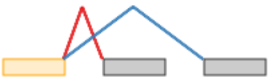	26 0 26
2064	CEP170	ENSG00000143702:243319523-243320123:source	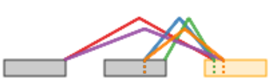	24 0 24
2065	ACP1	ENSG00000143727:276980-277085:target	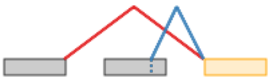	34 0 34
2066	ITPKB	ENSG00000143772:226836373-226836472:source	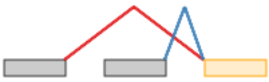	24 0 24
2067	ITPKB	ENSG00000143772:226923228-226925364:target	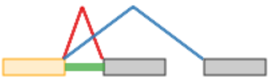	37 0 42
2068	GUK1	ENSG00000143774:228333584-228333936:source	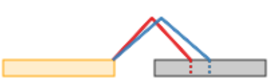	23 0 23
2069	GUK1	ENSG00000143774:228335316-228335411:source	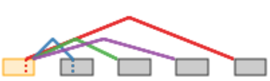	27 0 27
2070	GUK1	ENSG00000143774:228336366-228336425:target	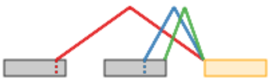	25 0 25
2071	PARP1	ENSG00000143799:226579900-226580015:source	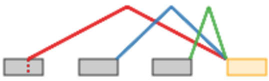	22 0 23
2072	PARP1	ENSG00000143799:226589684-226590080:target	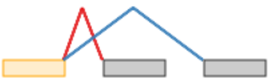	25 0 25
2073	LBR	ENSG00000143815:225592106-225592267:source	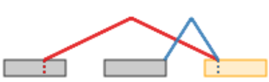	85 0 85
2074	LBR	ENSG00000143815:225594264-225594534:source	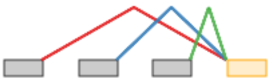	13 14 0 29
2075	LBR	ENSG00000143815:225594264-225594534:target	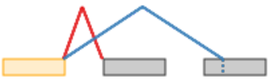	91 0 91
2076	LBR	ENSG00000143815:225597993-225598118:source	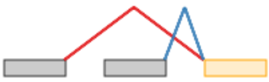	47 0 47
2077	LBR	ENSG00000143815:225599039-225599142:target	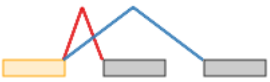	41 0 41
2078	LBR	ENSG00000143815:225600156-225600347:target	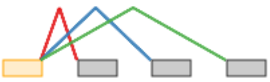	30 41 0 71
2079	PDIA6	ENSG00000143870:10923517-10924452:source	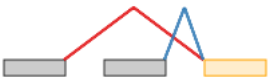	69 0 69

| # | Gene | LSV ID | LSV Type | ← More in Healthy \| More in ALL → |

#	Gene	LSV ID	LSV Type	← More in Healthy | More in ALL →
2080	PDIA6	ENSG00000143870:10927407-10927565:target	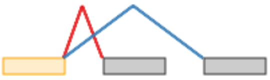	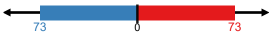
2081	EML4	ENSG00000143924:42483641-42483770:source	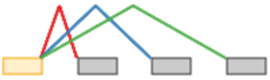	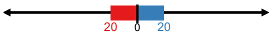
2082	EML4	ENSG00000143924:42509963-42510112:source	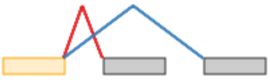	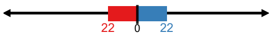
2083	EML4	ENSG00000143924:42522521-42522656:source	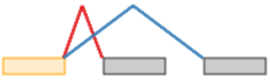	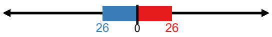
2084	EML4	ENSG00000143924:42530244-42530369:target	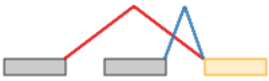	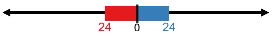
2085	SNRNP200	ENSG00000144028:96948939-96949090:target	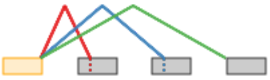	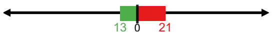
2086	AFF3	ENSG00000144218:100758946-100759057:target	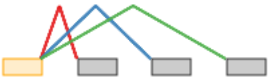	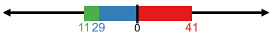
2087	SPOPL	ENSG00000144228:139308473-139308624:source	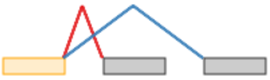	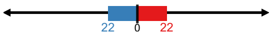
2088	SPOPL	ENSG00000144228:139316592-139316769:target	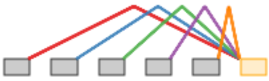	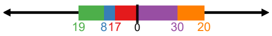
2089	RQCD1	ENSG00000144580:219447694-219447809:source	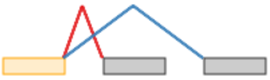	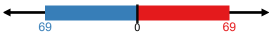
2090	RQCD1	ENSG00000144580:219452309-219452418:target	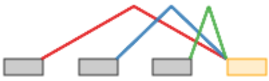	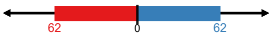
2091	IQSEC1	ENSG00000144711:12950301-12951194:target	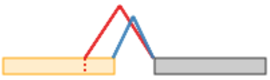	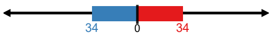
2092	IQSEC1	ENSG00000144711:12983071-12983365:source	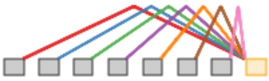	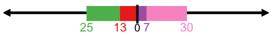
2093	SLC25A26	ENSG00000144741:66293627-66293736:target	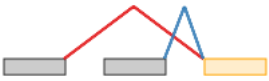	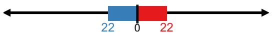
2094	TMF1	ENSG00000144747:69074711-69074863:source	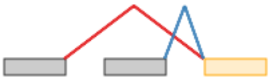	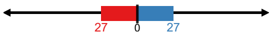
2095	TMF1	ENSG00000144747:69077051-69077143:target	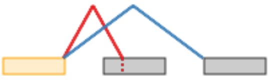	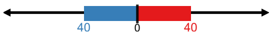
2096	NFKBIZ	ENSG00000144802:101570929-101571068:target	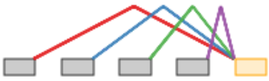	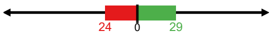
2097	KIAA0226	ENSG00000145016:197404351-197404762:source	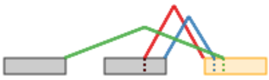	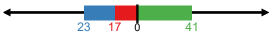
2098	KIAA0226	ENSG00000145016:197411028-197411088:source	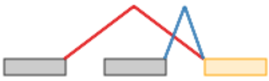	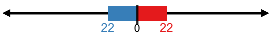
2099	VPRBP	ENSG00000145041:51450736-51450840:source	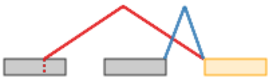	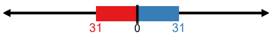

| # | Gene | LSV ID | LSV Type | ← More in Healthy \| More in ALL → |

#	Gene	LSV ID	LSV Type	← More in Healthy | More in ALL →
2100	VPRBP	ENSG00000145041:51452078-51452311:target	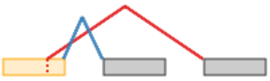	49 0 49
2101	FIP1L1	ENSG00000145216:54244006-54244090:source	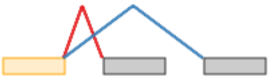	41 0 41
2102	FIP1L1	ENSG00000145216:54245392-54245431:target	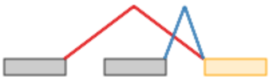	30 0 30
2103	FIP1L1	ENSG00000145216:54257176-54257306:source	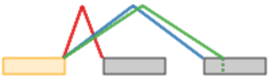	46 0 43
2104	FIP1L1	ENSG00000145216:54294194-54294388:source	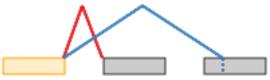	26 0 26
2105	FIP1L1	ENSG00000145216:54308420-54308874:target	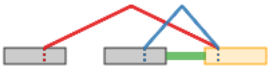	29 0 26
2106	FIP1L1	ENSG00000145216:54915122-54915277:target	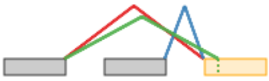	23 0 22
2107	MARCH6	ENSG00000145495:10417382-10417563:source	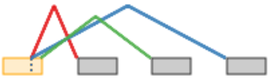	52 0 52
2108	MARCH6	ENSG00000145495:10423847-10423947:target	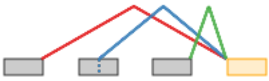	35 0 35
2109	SSBP2	ENSG00000145687:80733249-80733649:source	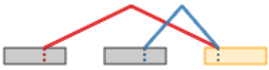	23 0 23
2110	TNFAIP8	ENSG00000145779:118728511-118735383:target	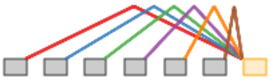	28 0 26
2111	FBXO38	ENSG00000145868:147812796-147813087:target	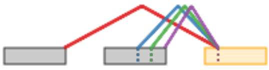	33 0 8 25
2112	TNIP1	ENSG00000145901:150410250-150410308:source	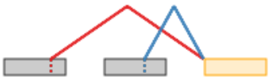	28 0 28
2113	TNIP1	ENSG00000145901:150414057-150414628:target	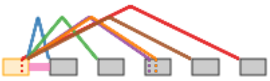	20 5 0 27
2114	TNIP1	ENSG00000145901:150422457-150422522:source	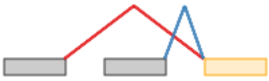	31 0 31
2115	TNIP1	ENSG00000145901:150429385-150429508:target	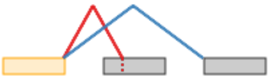	24 0 24
2116	TNIP1	ENSG00000145901:150444521-150444692:source	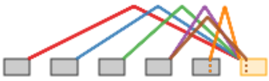	28 0 28
2117	TNIP1	ENSG00000145901:150460441-150460997:target	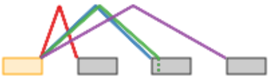	27 0 30
2118	G3BP1	ENSG00000145907:151175040-151175136:target	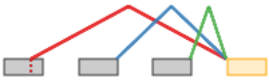	20 0 21
2119	FAM217A	ENSG00000145975:4063308-4063353:target	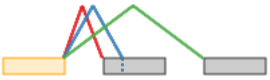	20 7 0 28
#	Gene	LSV ID	LSV Type	← More in Healthy | More in ALL →

#	Gene	LSV ID	LSV Type	← More in Healthy | More in ALL →
2120	RNF44	ENSG00000146083:175958464-175958631:target	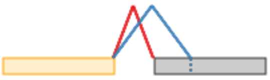	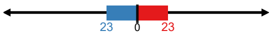
2121	FGD2	ENSG00000146192:36980801-36981541:source	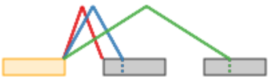	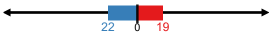
2122	FGD2	ENSG00000146192:36995205-36995351:target	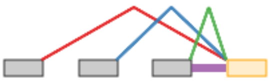	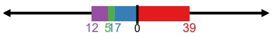
2123	PHIP	ENSG00000146247:79668192-79668317:target	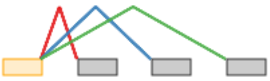	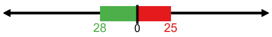
2124	PHIP	ENSG00000146247:79675420-79675481:source	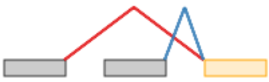	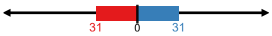
2125	PHIP	ENSG00000146247:79679552-79679634:source	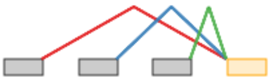	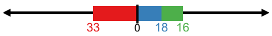
2126	PHIP	ENSG00000146247:79679552-79679634:target	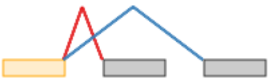	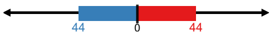
2127	PHIP	ENSG00000146247:79688309-79688428:target	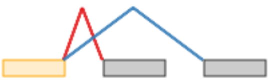	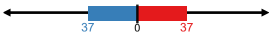
2128	WTAP	ENSG00000146457:160157290-160157750:target	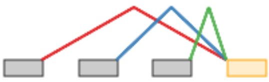	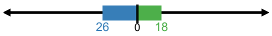
2129	WTAP	ENSG00000146457:160174492-160174646:target	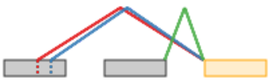	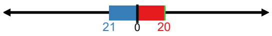
2130	GNA12	ENSG00000146535:2767746-2771384:source	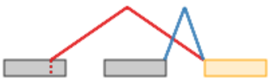	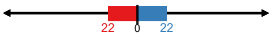
2131	GNA12	ENSG00000146535:2834354-2834779:target	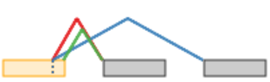	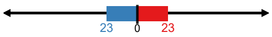
2132	WASH2P	ENSG00000146556:114346127-114346280:source	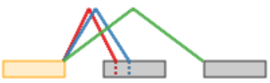	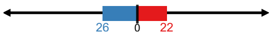
2133	GIGYF1	ENSG00000146830:100285827-100287071:target	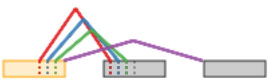	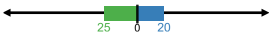
2134	TLK2	ENSG00000146872:60598134-60598205:target	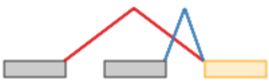	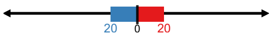
2135	TLK2	ENSG00000146872:60613531-60613698:target	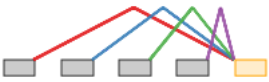	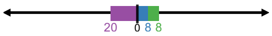
2136	C7orf55-LUC7L2	ENSG00000146963:139044654-139045068:source	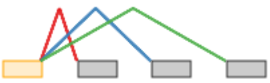	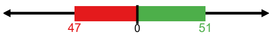
2137	C7orf55-LUC7L2	ENSG00000146963:139060808-139060902:target	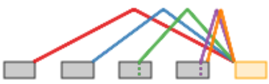	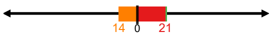
2138	NONO	ENSG00000147140:70510479-70510641:target	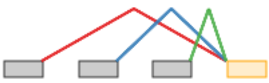	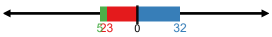
2139	RBMX	ENSG00000147274:135957245-135957327:target	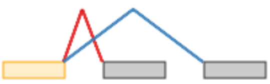	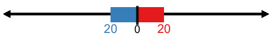

Gene LSV ID LSV Type ← More in Healthy | More in ALL →

#	Gene	LSV ID	LSV Type	← More in Healthy | More in ALL →
2140	HMBOX1	ENSG00000147421:28876277-28876430:source	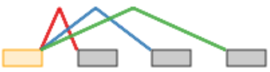	21 0 22
2141	HMBOX1	ENSG00000147421:28876277-28876430:target	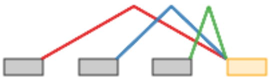	18 0 20
2142	HMBOX1	ENSG00000147421:28902878-28902960:source	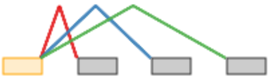	37 0 37
2143	HMBOX1	ENSG00000147421:28904875-28904970:target	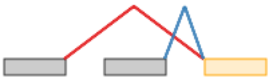	37 0 37
2144	TACC1	ENSG00000147526:38646222-38646337:target	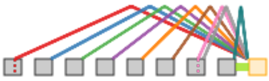	28 0 33
2145	TACC1	ENSG00000147526:38684657-38685585:source	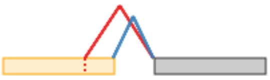	22 0 22
2146	TACC1	ENSG00000147526:38695988-38696064:source	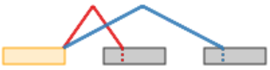	30 0 30
2147	TACC1	ENSG00000147526:38697569-38698136:target	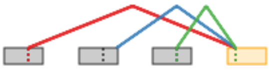	17 0 23
2148	WHSC1L1	ENSG00000147548:38156962-38157312:source	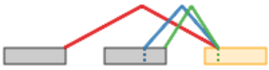	28 11 0 40
2149	WHSC1L1	ENSG00000147548:38162105-38162275:target	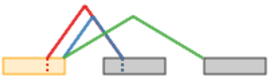	32 0 29
2150	WHSC1L1	ENSG00000147548:38162766-38162963:target	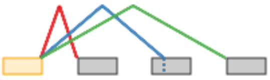	17 0 25
2151	TERF1	ENSG00000147601:73939175-73939287:source	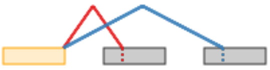	44 0 44
2152	TERF1	ENSG00000147601:73943848-73944368:target	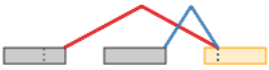	42 0 42
2153	MTDH	ENSG00000147649:98725890-98725997:target	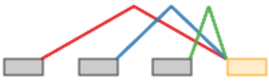	20 0 20
2154	NDUFB9	ENSG00000147684:125567983-125568145:target	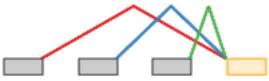	15 0 20
2155	UHRF2	ENSG00000147854:6460573-6460791:source	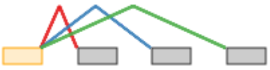	41 05 36
2156	UHRF2	ENSG00000147854:6475391-6475500:target	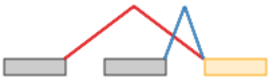	29 0 29
2157	UHRF2	ENSG00000147854:6486821-6486925:source	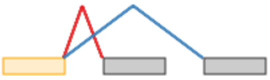	34 0 34
2158	UHRF2	ENSG00000147854:6493826-6493932:target	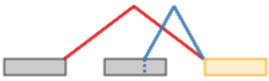	24 0 24
2159	C9orf72	ENSG00000147894:27548555-27548664:source	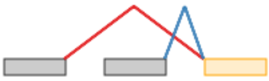	22 0 22
#	Gene	LSV ID	LSV Type	← More in Healthy | More in ALL →

#	Gene	LSV ID	LSV Type	← More in Healthy | More in ALL →
2160	CBWD5	ENSG00000147996:70432004-70432535:source		24 0 24
2161	CBWD5	ENSG00000147996:70433652-70434243:target		30 0 30
2162	SURF4	ENSG00000148248:136230431-136230635:source		32 0 32
2163	SURF4	ENSG00000148248:136232861-136232953:target		37 0 37
2164	GPR107	ENSG00000148358:132869775-132869818:target		21 0 21
2165	SEC16A	ENSG00000148396:139338275-139338534:source		50 0 40 9
2166	SEC16A	ENSG00000148396:139340916-139341467:target		55 0 56
2167	SEC16A	ENSG00000148396:139350505-139350618:source		34 0 34
2168	SEC16A	ENSG00000148396:139353586-139353691:target		56 0 56
2169	SEC16A	ENSG00000148396:139355629-139355726:source		23 0 23
2170	SEC16A	ENSG00000148396:139358872-139359014:target		29 0 29
2171	USP6NL	ENSG00000148429:11532831-11532879:target		20 26 0 48
2172	USP6NL	ENSG00000148429:11639630-11639716:target		20 0 20
2173	FAM188A	ENSG00000148481:15879203-15879317:source		35 0 18 18
2174	ADD3	ENSG00000148700:111860383-111860606:target		41 0 32 5
2175	ADD3	ENSG00000148700:111872535-111872673:source		24 0 28
2176	ADD3	ENSG00000148700:111877100-111877180:target		25 0 25
2177	MTG1	ENSG00000148824:135216145-135216343:source		21 0 21
2178	EIF3M	ENSG00000149100:32610140-32610473:source		24 0 24
2179	ARFGAP2	ENSG00000149182:47196705-47196964:source		22 0 22

| # | Gene | LSV ID | LSV Type | ← More in Healthy \| More in ALL → |

#	Gene	LSV ID	LSV Type	← More in Healthy | More in ALL →
2180	ARFGAP2	ENSG00000149182:47197765-47198185:target	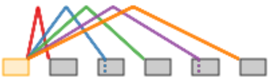	38 0 44
2181	CELF1	ENSG00000149187:47494640-47494783:source	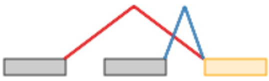	26 0 26
2182	CELF1	ENSG00000149187:47500429-47500504:target	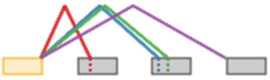	43 0 49
2183	CELF1	ENSG00000149187:47503994-47504408:source	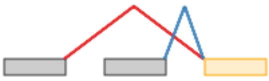	27 0 27
2184	CELF1	ENSG00000149187:47505941-47506075:target	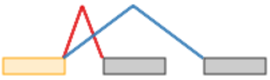	35 0 35
2185	ATM	ENSG00000149311:108106397-108107261:source	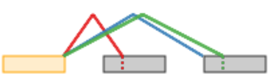	18 0 23
2186	ATM	ENSG00000149311:108114680-108114845:target	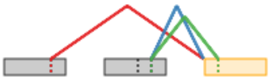	20 0 17
2187	ATM	ENSG00000149311:108157905-108158442:target	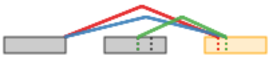	11 13 0 23
2188	CPSF7	ENSG00000149532:61183138-61183256:target	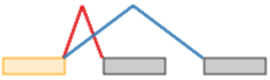	20 0 20
2189	CPSF7	ENSG00000149532:61183650-61183991:source	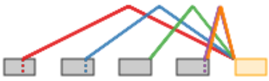	6 16 0 32
2190	CPSF7	ENSG00000149532:61187394-61187566:source	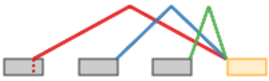	45 0 45
2191	CPSF7	ENSG00000149532:61188786-61189080:target	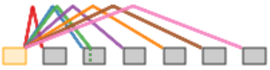	10 25 0 6 26
2192	ALDOA	ENSG00000149925:30078555-30078687:target	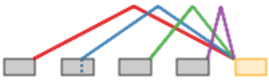	20 0 20
2193	ARID5B	ENSG00000150347:63845461-63845659:target	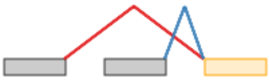	27 0 27
2194	MTMR12	ENSG00000150712:32227100-32230453:source	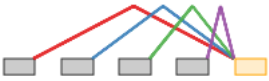	7 32 0 37
2195	MTMR12	ENSG00000150712:32239107-32239279:target	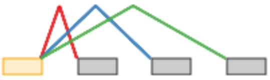	42 0 42
2196	MTMR12	ENSG00000150712:32242163-32242233:target	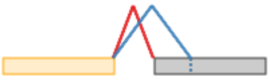	20 0 20
2197	UBC	ENSG00000150991:125397814-125398320:source	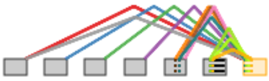	18 0 20
2198	ITPR1	ENSG00000150995:4693807-4693902:source	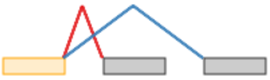	30 0 30
2199	ITPR1	ENSG00000150995:4699808-4699962:target	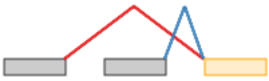	34 0 34

| # | Gene | LSV ID | LSV Type | ← More in Healthy \| More in ALL → |

#	Gene	LSV ID	LSV Type	← More in Healthy | More in ALL →
2200	ITPR1	ENSG00000150995:4829631-4829823:source	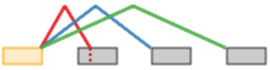	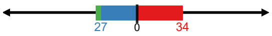
2201	ITPR1	ENSG00000150995:4842109-4842284:target	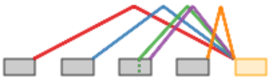	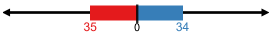
2202	NGLY1	ENSG00000151092:25760435-25761126:source	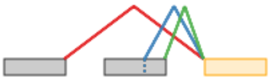	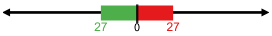
2203	NGLY1	ENSG00000151092:25770624-25772041:source	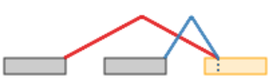	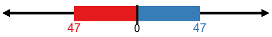
2204	NGLY1	ENSG00000151092:25775363-25775475:source	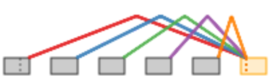	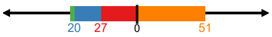
2205	NGLY1	ENSG00000151092:25775363-25775475:target	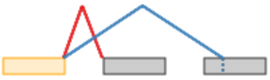	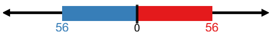
2206	NGLY1	ENSG00000151092:25777495-25777640:source	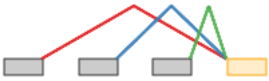	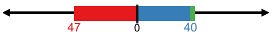
2207	NGLY1	ENSG00000151092:25781068-25781290:source	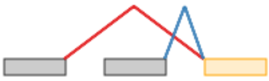	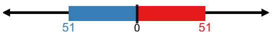
2208	NGLY1	ENSG00000151092:25781068-25781290:target	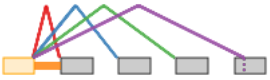	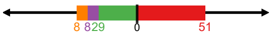
2209	NGLY1	ENSG00000151092:25792589-25792754:target	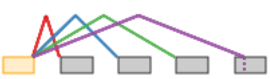	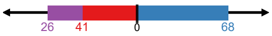
2210	EIF4E	ENSG00000151247:99823027-99823133:source	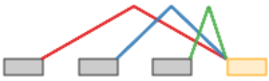	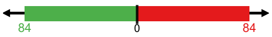
2211	NEK7	ENSG00000151414:198248084-198248191:source	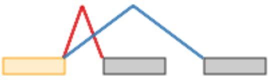	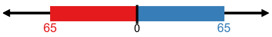
2212	NEK7	ENSG00000151414:198262075-198262169:target	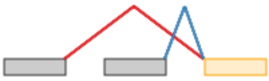	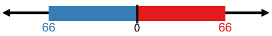
2213	UPF2	ENSG00000151461:11994065-11994248:source	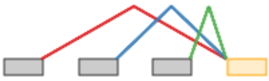	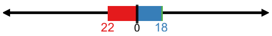
2214	ADAM8	ENSG00000151651:135075907-135076737:source	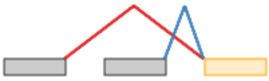	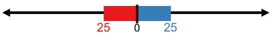
2215	PARP8	ENSG00000151883:49963718-49965351:source	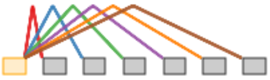	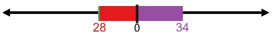
2216	PARP8	ENSG00000151883:50045985-50046022:target	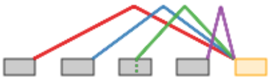	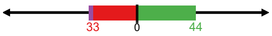
2217	PARP8	ENSG00000151883:50055477-50055566:source	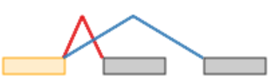	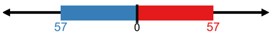
2218	PARP8	ENSG00000151883:50057655-50057736:source	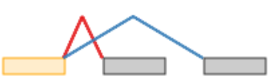	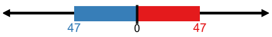
2219	PARP8	ENSG00000151883:50057655-50057736:target	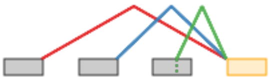	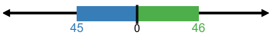

| # | Gene | LSV ID | LSV Type | ← More in Healthy \| More in ALL → |

#	Gene	LSV ID	LSV Type	← More in Healthy | More in ALL →
2220	PARP8	ENSG00000151883:50073903-50073963:target		41 0 41
2221	PARP8	ENSG00000151883:50111266-50111358:source		68 0 68
2222	PARP8	ENSG00000151883:50118169-50118234:source		28 0 28
2223	PARP8	ENSG00000151883:50118169-50118234:target		38 0 38
2224	PARP8	ENSG00000151883:50122589-50122637:target		44 0 44
2225	PARP8	ENSG00000151883:50129813-50129882:source		20 0 20
2226	TIAL1	ENSG00000151923:121336123-121336262:source		28 0 28
2227	TIAL1	ENSG00000151923:121336592-121336726:target		31 0 31
2228	TIAL1	ENSG00000151923:121339447-121339522:source		29 0 29
2229	TIAL1	ENSG00000151923:121339983-121340358:source		55 0 45 10
2230	TIAL1	ENSG00000151923:121341245-121341521:target		48 0 20 29
2231	ENSG00000152042	ENSG00000152042:146052552-146052763:source		22 0 22
2232	RABGAP1L	ENSG00000152061:174926594-174926686:source		26 0 26
2233	AC093838.4	ENSG00000152117:132273887-132273977:target		21 0 21
2234	MGAT5	ENSG00000152127:135119846-135119979:source		25 0 28
2235	MGAT5	ENSG00000152127:135160559-135160708:target		15 0 32
2236	PDK1	ENSG00000152256:173426948-173427019:source		33 0 29
2237	USP12	ENSG00000152484:27640293-27643521:source		47 0 47
2238	USP12	ENSG00000152484:27649328-27649525:target		31 0 31
2239	USP12	ENSG00000152484:27745729-27746033:target		23 0 23

Gene LSV ID LSV Type ← More in Healthy | More in ALL →

#	Gene	LSV ID	LSV Type	← More in Healthy | More in ALL →
2240	PAN3	ENSG00000152520:28771322-28771483:target	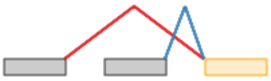	31 0 31
2241	TMEM123	ENSG00000152558:102272475-102272937:source	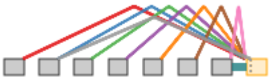	9 5 5 0 36
2242	TMEM123	ENSG00000152558:102319543-102319599:target	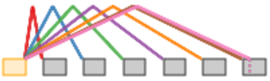	10 0 29
2243	MBNL1	ENSG00000152601:152017983-152018156:source	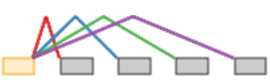	35 0 31
2244	HNRNPDL	ENSG00000152795:83346716-83346947:source	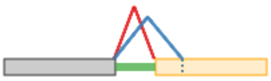	21 0 21
2245	UTRN	ENSG00000152818:144724259-144724320:target	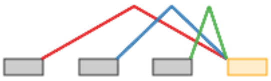	35 0 34
2246	UTRN	ENSG00000152818:145148747-145148812:target	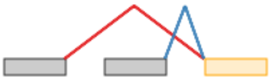	25 0 25
2247	UTRN	ENSG00000152818:145157437-145157749:source	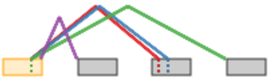	21 0 11 5
2248	SRP19	ENSG00000153037:112200306-112200916:source	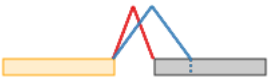	41 0 41
2249	BANK1	ENSG00000153064:102750965-102751363:target	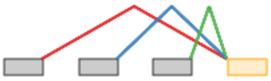	44 0 44
2250	CAST	ENSG00000153113:96065294-96065429:source	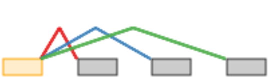	25 0 26
2251	CAST	ENSG00000153113:96071876-96071944:target	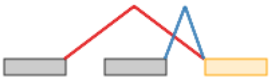	25 0 25
2252	SMARCA5	ENSG00000153147:144459939-144460091:source	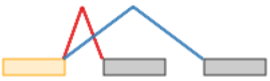	41 0 41
2253	SMARCA5	ENSG00000153147:144464662-144464810:target	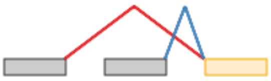	53 0 53
2254	SMARCA5	ENSG00000153147:144466623-144466736:source	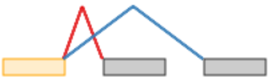	26 0 26
2255	SMARCA5	ENSG00000153147:144467929-144468061:target	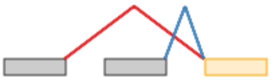	20 0 20
2256	RGPD3	ENSG00000153165:107057749-107057836:target	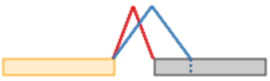	43 0 43
2257	AHCTF1	ENSG00000153207:247002400-247004300:source	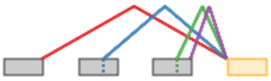	37 0 38
2258	AHCTF1	ENSG00000153207:247012917-247013151:target	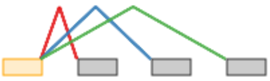	29 0 22
2259	AHCTF1	ENSG00000153207:247053268-247053361:source	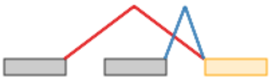	32 0 32
#	Gene	LSV ID	LSV Type	← More in Healthy | More in ALL →

#	Gene	LSV ID	LSV Type	← More in Healthy | More in ALL →
2260	AHCTF1	ENSG00000153207:247055081-247055221:target		34 0 34
2261	NR4A2	ENSG00000153234:157183230-157183432:target		23 0 23
2262	NR4A2	ENSG00000153234:157185835-157187143:target		21 0 21
2263	RBMS1	ENSG00000153250:161140772-161141379:target		22 0 22
2264	UBP1	ENSG00000153560:33444293-33444396:source		34 0 34
2265	UBP1	ENSG00000153560:33450728-33450838:target		23 0 23
2266	CMIP	ENSG00000153815:81694489-81694551:source		20 0 20
2267	TRIP12	ENSG00000153827:230633947-230634041:source		32 0 32
2268	TRIP12	ENSG00000153827:230638277-230638977:target		50 0 50
2269	TRIP12	ENSG00000153827:230744698-230744844:source		20 0 16
2270	SREK1	ENSG00000153914:65449396-65449618:source		43 0 23 21
2271	SREK1	ENSG00000153914:65459411-65459750:source		31 0 31
2272	SREK1	ENSG00000153914:65470452-65470869:source		22 0 20
2273	CHD1	ENSG00000153922:98210776-98210817:source		21 0 21
2274	MSI2	ENSG00000153944:55607037-55607085:target		20 0 18
2275	ZUFSP	ENSG00000153975:116973167-116973355:target		23 0 23
2276	MIA3	ENSG00000154305:222818896-222819477:target		20 0 20
2277	MIA3	ENSG00000154305:222827997-222828135:source		6 29 0 36
2278	LONRF1	ENSG00000154359:12594409-12594649:source		21 0 21
2279	LONRF1	ENSG00000154359:12595504-12595653:target		20 0 20

Gene LSV ID LSV Type ← More in Healthy | More in ALL →

#	Gene	LSV ID	LSV Type	← More in Healthy | More in ALL →
2280	BUB3	ENSG00000154473:124921752-124921929:target	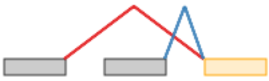	25 0 25
2281	BUB3	ENSG00000154473:124922128-124922757:source	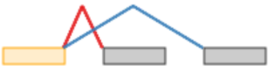	30 0 30
2282	BTG3	ENSG00000154640:18970935-18971409:source	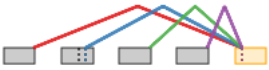	22 0 22
2283	GABPA	ENSG00000154727:27113884-27113986:target	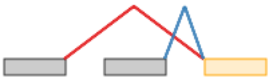	29 0 29
2284	XPC	ENSG00000154767:14211938-14212114:target	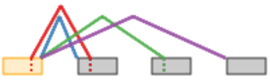	13 12 0 26
2285	PPP4R1	ENSG00000154845:9561978-9562073:source	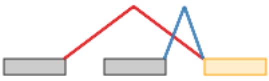	26 0 26
2286	ACSS1	ENSG00000154930:24994597-24994709:source	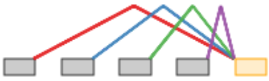	8 14 0 24
2287	ACSS1	ENSG00000154930:25011395-25011594:target	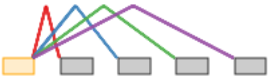	21 15 0 31
2288	AZIN1	ENSG00000155096:103842049-103842164:source	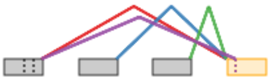	20 0 23
2289	AZIN1	ENSG00000155096:103846417-103846491:target	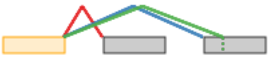	21 0 25
2290	AZIN1	ENSG00000155096:103855779-103855975:source	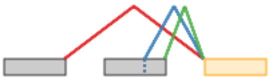	20 0 24
2291	AGPAT5	ENSG00000155189:6565878-6566683:source	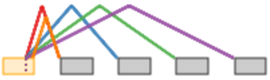	78 0 73
2292	AGPAT5	ENSG00000155189:6590082-6590171:source	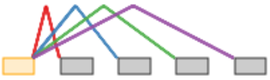	27 0 19
2293	AGPAT5	ENSG00000155189:6605191-6605349:target	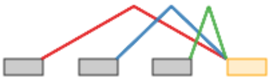	28 0 28
2294	SLC25A28	ENSG00000155287:101373226-101373681:source	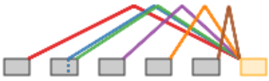	14 15 0 197
2295	USP25	ENSG00000155313:17177440-17177974:source	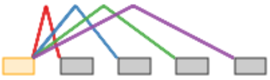	17 0 20
2296	USP25	ENSG00000155313:17197285-17197380:target	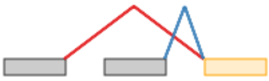	20 0 20
2297	ZCCHC10	ENSG00000155329:132332677-132334542:source	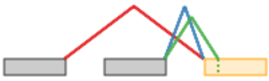	31 0 31
2298	ZCCHC10	ENSG00000155329:132342451-132342612:target	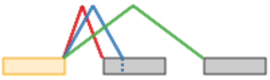	37 0 31
2299	LARP1	ENSG00000155506:154183100-154183305:source	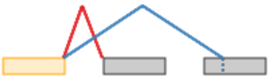	34 0 34

Gene LSV ID LSV Type ← More in Healthy | More in ALL →

#	Gene	LSV ID	LSV Type	← More in Healthy | More in ALL →
2300	LARP1	ENSG00000155506:154185314-154185592:target		39 0 39
2301	LARP1	ENSG00000155506:154193447-154197167:target		10 10 0 25
2302	CNOT8	ENSG00000155508:154244752-154244945:target		7 9 15 0 36
2303	PIK3AP1	ENSG00000155629:98411266-98411408:source		23 0 23
2304	TTN	ENSG00000155657:179403267-179403566:target		46 0 46
2305	RASA2	ENSG00000155903:141205889-141206058:source		29 0 30
2306	ADK	ENSG00000156110:76360137-76360251:target		24 0 24
2307	DRAM2	ENSG00000156171:111661182-111661519:source		25 0 25
2308	DRAM2	ENSG00000156171:111662499-111662583:target		13 11 0 26
2309	WHAMM	ENSG00000156232:83488075-83488240:source		21 0 21
2310	WHAMM	ENSG00000156232:83491852-83492039:target		25 0 25
2311	BACH1	ENSG00000156273:30693542-30693835:target		44 0 34 5
2312	PTDSS1	ENSG00000156471:97332474-97332573:source		20 0 20
2313	ZDHHC5	ENSG00000156599:57466031-57466890:target		22 0 22
2314	KAT6B	ENSG00000156650:76735157-76736088:source		29 0 30
2315	MAPK13	ENSG00000156711:36104433-36104547:source		58 0 58
2316	MAPK13	ENSG00000156711:36106479-36106557:target		53 0 53
2317	MS4A1	ENSG00000156738:60223225-60223418:source		39 0 39
2318	ATAD2	ENSG00000156802:124348613-124348772:source		49 0 49
2319	ATAD2	ENSG00000156802:124351551-124351686:target		52 0 52

Gene LSV ID LSV Type ← More in Healthy | More in ALL →

#	Gene	LSV ID	LSV Type	← More in Healthy | More in ALL →
2320	ATAD2	ENSG00000156802:124358858-124358990:target		56 0 56
2321	ATAD2	ENSG00000156802:124382061-124382264:source		31 0 29
2322	ATAD2	ENSG00000156802:124383476-124383578:target		28 0 28
2323	FBRS	ENSG00000156860:30671584-30671902:source		20 0 20
2324	FBRS	ENSG00000156860:30673299-30673789:target		8 18 0 27
2325	HIAT1	ENSG00000156875:100533533-100533751:source		24 0 26
2326	HIAT1	ENSG00000156875:100535170-100535241:target		20 0 20
2327	EIF4A2	ENSG00000156976:186501992-186502302:source		32 0 34
2328	EIF4A2	ENSG00000156976:186502353-186502733:source		38 0 36
2329	EIF4A2	ENSG00000156976:186503672-186503702:target		30 6 0 39
2330	EIF4A2	ENSG00000156976:186505565-186506045:source		37 0 37
2331	EIF4A2	ENSG00000156976:186506914-186507689:target		53 0 51
2332	BRPF1	ENSG00000156983:9783709-9783855:source		25 0 25
2333	C1orf27	ENSG00000157181:186375215-186375400:source		29 0 29
2334	APPL1	ENSG00000157500:57286297-57286339:source		52 0 52
2335	APPL1	ENSG00000157500:57290985-57291079:target		45 0 45
2336	DYRK1A	ENSG00000157540:38791207-38792687:source		47 0 9 36
2337	DYRK1A	ENSG00000157540:38844986-38845182:target		44 0 26 14
2338	PALM2-AKAP2	ENSG00000157654:112898407-112900819:source		18 0 23
2339	PALM2-AKAP2	ENSG00000157654:112918599-112918777:target		26 0 26

| # | Gene | LSV ID | LSV Type | ← More in Healthy \| More in ALL → |

#	Gene	LSV ID	LSV Type	← More in Healthy | More in ALL →
2340	BRAF	ENSG00000157764:140507760-140507862:source	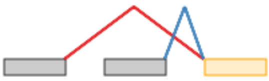	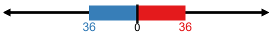
2341	BRAF	ENSG00000157764:140508692-140508795:target	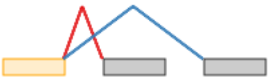	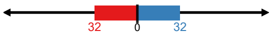
2342	AAED1	ENSG00000158122:99401859-99404168:source	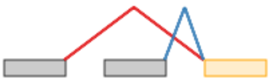	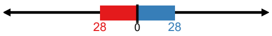
2343	AAED1	ENSG00000158122:99413672-99413777:target	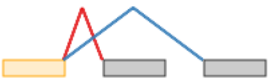	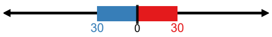
2344	ABHD3	ENSG00000158201:19281237-19282459:source	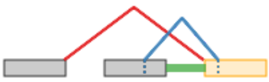	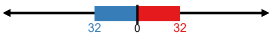
2345	AUTS2	ENSG00000158321:69364272-69364484:target	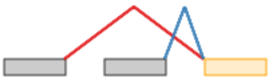	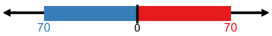
2346	NCF1	ENSG00000158517:74199528-74199892:source	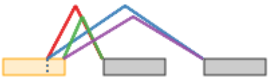	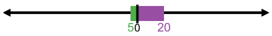
2347	NCF1	ENSG00000158517:74202328-74202432:target	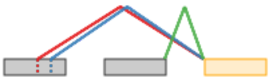	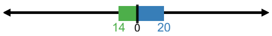
2348	ZC3H18	ENSG00000158545:88664586-88664734:target	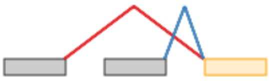	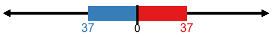
2349	USF1	ENSG00000158773:161015648-161015767:target	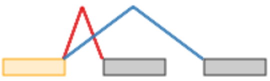	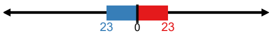
2350	ZNF276	ENSG00000158805:89804384-89807311:target	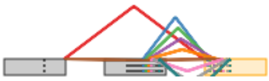	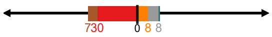
2351	CDC42SE2	ENSG00000158985:130651669-130651837:target	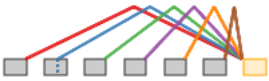	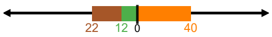
2352	CDC42SE2	ENSG00000158985:130726686-130726801:source	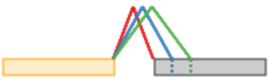	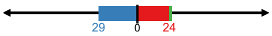
2353	EPB41	ENSG00000159023:29338377-29338419:target	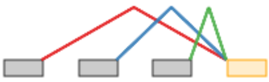	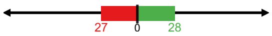
2354	IFNGR2	ENSG00000159128:34799191-34799339:source	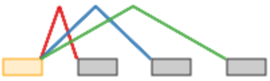	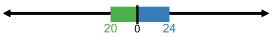
2355	IFNGR2	ENSG00000159128:34805021-34805178:source	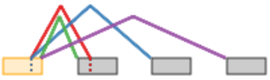	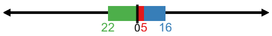
2356	IFNGR2	ENSG00000159128:34805021-34805178:target	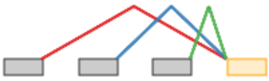	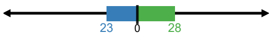
2357	SON	ENSG00000159140:34921782-34927697:target	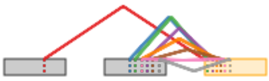	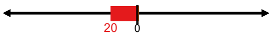
2358	SON	ENSG00000159140:34929462-34929622:source	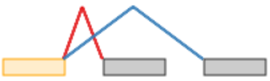	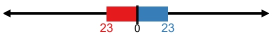
2359	SON	ENSG00000159140:34931893-34932081:source	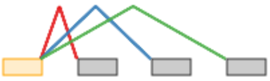	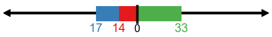

Gene LSV ID LSV Type ← More in Healthy | More in ALL →

#	Gene	LSV ID	LSV Type	← More in Healthy | More in ALL →
2360	SON	ENSG00000159140:34945331-34945761:target	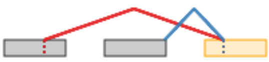	25 0 25
2361	DONSON	ENSG00000159147:34931848-34932081:source	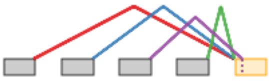	14 17 0 32
2362	DONSON	ENSG00000159147:34945596-34945761:target	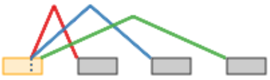	24 0 24
2363	RUNX1	ENSG00000159216:36205258-36206898:target	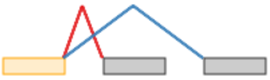	24 0 24
2364	RUNX1	ENSG00000159216:36421139-36421255:target	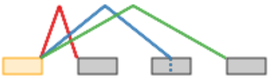	36 0 36
2365	ARHGAP27	ENSG00000159314:43483042-43483449:source	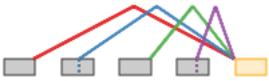	28 0 28
2366	ADPGK	ENSG00000159322:73043710-73045482:source	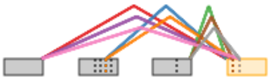	19 0 13 14
2367	ADPGK	ENSG00000159322:73052564-73052868:target	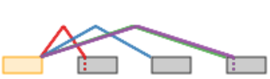	19 0 28
2368	ADPGK	ENSG00000159322:73064124-73064186:target	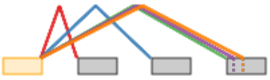	12 0 23
2369	ADPGK	ENSG00000159322:73075587-73076126:target	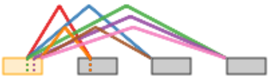	24 0 24
2370	ADIPOR1	ENSG00000159346:202920058-202920292:source	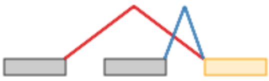	47 0 47
2371	PSMD4	ENSG00000159352:151237308-151237415:source	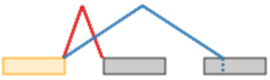	23 0 23
2372	PSMB4	ENSG00000159377:151372918-151373064:source	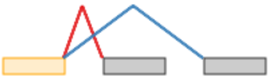	25 0 25
2373	PSMB4	ENSG00000159377:151373715-151373831:target	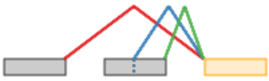	1027 0 40
2374	GPBP1L1	ENSG00000159592:46152084-46152325:target	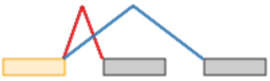	21 0 21
2375	EFCAB14	ENSG00000159658:47152456-47152601:source	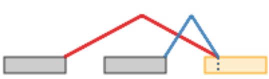	35 0 35
2376	EFCAB14	ENSG00000159658:47155259-47155363:target	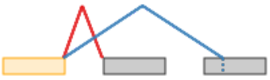	32 0 32
2377	CTBP1	ENSG00000159692:1231403-1232125:target	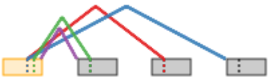	16 0 25
2378	ATP6V0D1	ENSG00000159720:67477002-67477310:source	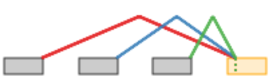	612 0 22
2379	ATP6V0D1	ENSG00000159720:67478431-67478609:source	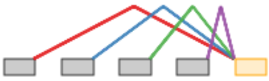	22 0 25

Gene LSV ID LSV Type ← More in Healthy | More in ALL →

#	Gene	LSV ID	LSV Type	← More in Healthy | More in ALL →
2380	ATP6V0D1	ENSG00000159720:67514860-67515140:target	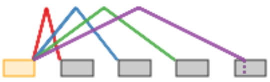	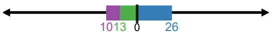 10 13 0 26
2381	ABR	ENSG00000159842:1003877-1003975:source	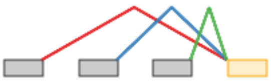	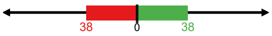 38 0 38
2382	GNE	ENSG00000159921:36233917-36234129:source	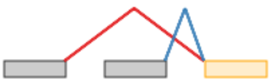	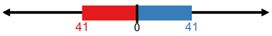 41 0 41
2383	BSDC1	ENSG00000160058:32849542-32849926:source	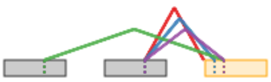	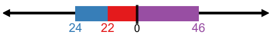 24 22 0 46
2384	BSDC1	ENSG00000160058:32852022-32852496:target	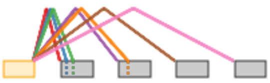	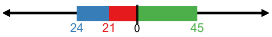 24 21 0 45
2385	SLC37A1	ENSG00000160190:43945886-43946148:source	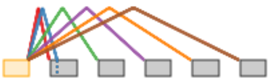	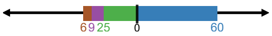 6 9 25 0 60
2386	SLC37A1	ENSG00000160190:43955582-43955660:target	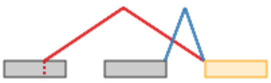	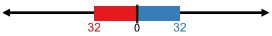 32 0 32
2387	SLC37A1	ENSG00000160190:43959622-43959757:source	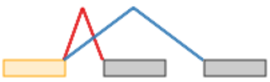	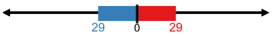 29 0 29
2388	SLC37A1	ENSG00000160190:43963546-43963712:target	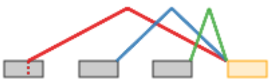	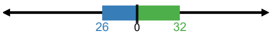 26 0 32
2389	SLC37A1	ENSG00000160190:43999846-43999910:target	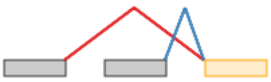	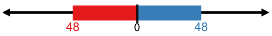 48 0 48
2390	U2AF1	ENSG00000160201:44514765-44514898:source	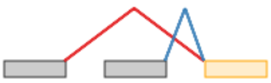	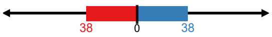 38 0 38
2391	U2AF1	ENSG00000160201:44515804-44516500:target	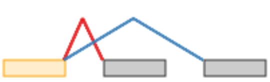	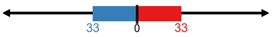 33 0 33
2392	U2AF1	ENSG00000160201:44521476-44522017:target	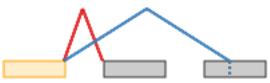	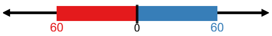 60 0 60
2393	U2AF1	ENSG00000160201:44524425-44525362:source	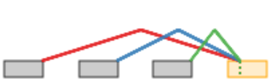	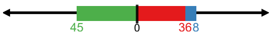 45 0 36 8
2394	TRAPPC10	ENSG00000160218:45483419-45483666:source	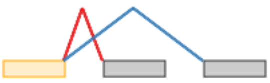	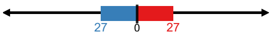 27 0 27
2395	TRAPPC10	ENSG00000160218:45494920-45495034:target	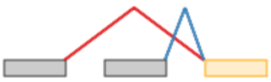	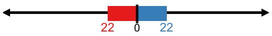 22 0 22
2396	TRAPPC10	ENSG00000160218:45502669-45503183:source	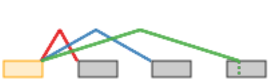	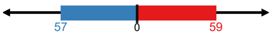 57 0 59
2397	TRAPPC10	ENSG00000160218:45506683-45506841:target	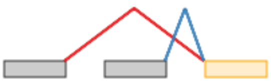	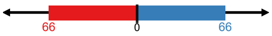 66 0 66
2398	ICOSLG	ENSG00000160223:45656750-45657100:source	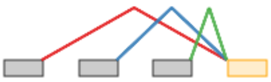	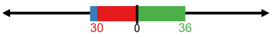 30 0 36
2399	ICOSLG	ENSG00000160223:45660687-45660849:target	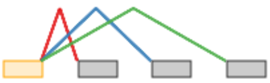	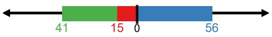 41 15 0 56
#	Gene	LSV ID	LSV Type	← More in Healthy | More in ALL →

#	Gene	LSV ID	LSV Type	← More in Healthy | More in ALL →
2400	RALGDS	ENSG00000160271:135982032-135982135:source		29 0 29
2401	ST6GALNAC6	ENSG00000160408:130658521-130658611:source		29 0 11 16
2402	SIK3	ENSG00000160584:116731990-116732100:source		48 0 48
2403	SIK3	ENSG00000160584:116732918-116733043:target		51 0 59
2404	SIK3	ENSG00000160584:116734384-116735784:source		29 7 0 39
2405	SIK3	ENSG00000160584:116746969-116747079:source		28 0 28
2406	SIK3	ENSG00000160584:116766969-116767092:target		36 0 40
2407	SIK3	ENSG00000160584:116797935-116798096:source		19 0 21
2408	SIK3	ENSG00000160584:116827664-116827780:target		23 0 31
2409	PCSK7	ENSG00000160613:117078686-117079204:source		22 0 22
2410	PCSK7	ENSG00000160613:117089182-117089471:target		22 0 29
2411	ADAR	ENSG00000160710:154558123-154558341:source		37 0 37
2412	ADAR	ENSG00000160710:154560601-154560756:target		30 0 30
2413	ADAR	ENSG00000160710:154572653-154574724:target		25 0 25
2414	ENSG00000160828	ENSG00000160828:74202328-74202432:target		14 0 20
2415	ENSG00000160828	ENSG00000160828:74299285-74299569:source		33 0 26
2416	ZNF394	ENSG00000160908:99090863-99092254:source		29 0 29
2417	ORAI2	ENSG00000160991:102086960-102097268:target		13 0 20
2418	C5orf45	ENSG00000161010:179274357-179275066:source		17 0 22
2419	SQSTM1	ENSG00000161011:179260587-179260782:target		25 0 26

| # | Gene | LSV ID | LSV Type | ← More in Healthy \| More in ALL → |

#	Gene	LSV ID	LSV Type	← More in Healthy | More in ALL →
2420	SAP30BP	ENSG00000161526:73698560-73698651:source	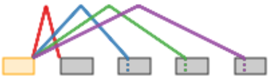	10 17 0 30
2421	SRSF2	ENSG00000161547:74730659-74731240:source	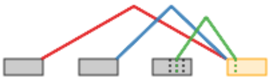	23 0 24
2422	GRASP	ENSG00000161835:52407656-52407727:source	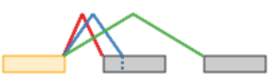	39 0 41
2423	LEMD2	ENSG00000161904:33738979-33740555:source	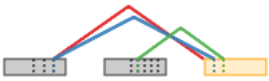	38 0 39
2424	CYB561A3	ENSG00000162144:61120428-61121034:source	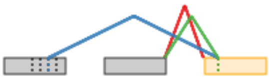	39 0 40
2425	NXF1	ENSG00000162231:62571264-62571450:source	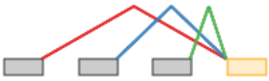	57 0 57
2426	STX5	ENSG00000162236:62592497-62592589:target	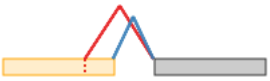	20 0 20
2427	MAGOH	ENSG00000162385:53694544-53694626:source	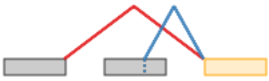	24 0 24
2428	MAGOH	ENSG00000162385:53701249-53701307:target	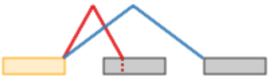	25 0 25
2429	USP24	ENSG00000162402:55569549-55569693:source	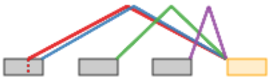	35 0 34
2430	USP24	ENSG00000162402:55590993-55591221:target	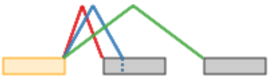	18 7 0 28
2431	USP24	ENSG00000162402:55608652-55608792:source	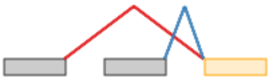	22 0 22
2432	USP24	ENSG00000162402:55611671-55611782:target	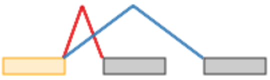	28 0 28
2433	GMEB1	ENSG00000162419:29010076-29010252:source	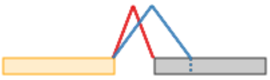	39 0 39
2434	JAK1	ENSG00000162434:65344708-65344831:source	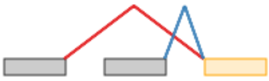	45 0 45
2435	JAK1	ENSG00000162434:65348960-65349158:target	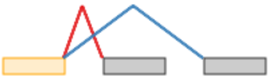	30 0 30
2436	LAPTM5	ENSG00000162511:31214487-31215100:source	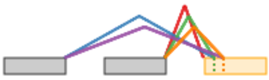	22 0 27
2437	LAPTM5	ENSG00000162511:31230506-31230667:target	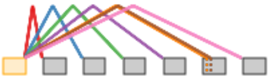	28 0 32
2438	TM2D1	ENSG00000162604:62148944-62149529:source	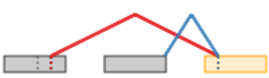	23 0 23
2439	FUBP1	ENSG00000162613:78409740-78409954:source	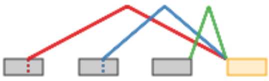	25 0 20

| # | Gene | LSV ID | LSV Type | ← More in Healthy \| More in ALL → |

#	Gene	LSV ID	LSV Type	← More in Healthy | More in ALL →
2440	FUBP1	ENSG00000162613:78433849-78433887:source	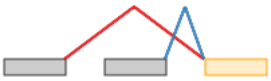	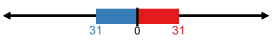
2441	FUBP1	ENSG00000162613:78435609-78435699:target	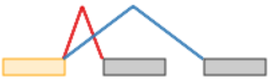	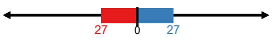
2442	PEA15	ENSG00000162734:160181333-160181506:source	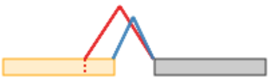	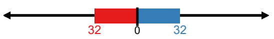
2443	DENND2D	ENSG00000162777:111730708-111730992:target	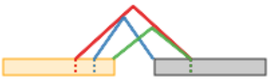	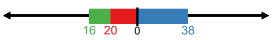
2444	WDR26	ENSG00000162923:224576838-224577562:source	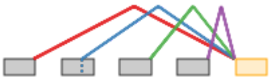	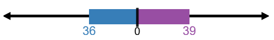
2445	WDR26	ENSG00000162923:224585429-224585928:source	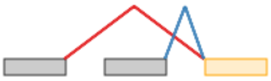	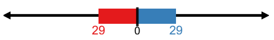
2446	WDR26	ENSG00000162923:224585429-224585928:target	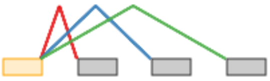	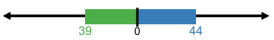
2447	WDR26	ENSG00000162923:224586591-224586736:target	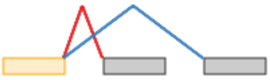	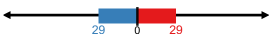
2448	WDR26	ENSG00000162923:224616999-224619283:target	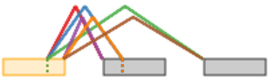	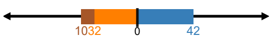
2449	REL	ENSG00000162924:61121532-61121680:source	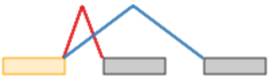	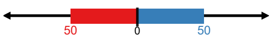
2450	MEMO1	ENSG00000162959:32142995-32143106:target	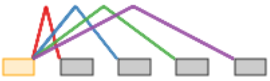	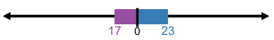
2451	DPY30	ENSG00000162961:32142995-32143106:target	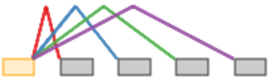	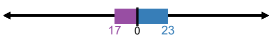
2452	SMC6	ENSG00000163029:17864905-17865030:target	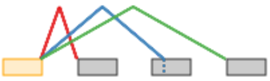	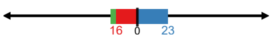
2453	SMC6	ENSG00000163029:17912345-17912406:target	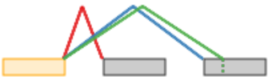	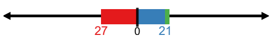
2454	RNF149	ENSG00000163162:101897955-101898519:source	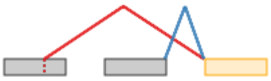	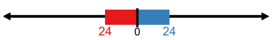
2455	RNF149	ENSG00000163162:101924303-101925163:target	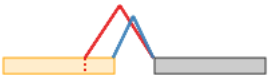	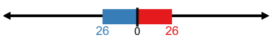
2456	ARHGAP25	ENSG00000163219:69009365-69009452:source	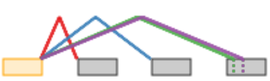	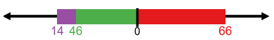
2457	ARHGAP25	ENSG00000163219:69034363-69034612:source	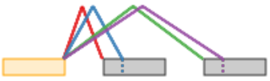	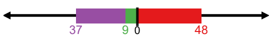
2458	ARHGAP25	ENSG00000163219:69034363-69034612:target	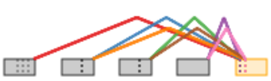	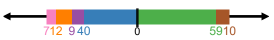
2459	ARHGAP25	ENSG00000163219:69043286-69043684:target	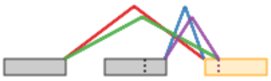	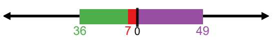

Gene LSV ID LSV Type ← More in Healthy | More in ALL →

#	Gene	LSV ID	LSV Type	← More in Healthy | More in ALL →
2460	HIPK1	ENSG00000163349:114495388-114495511:source	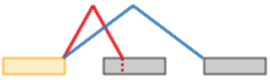	52 0 52
2461	HIPK1	ENSG00000163349:114498185-114498271:target	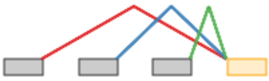	51 0 50
2462	HIPK1	ENSG00000163349:114512578-114512819:source	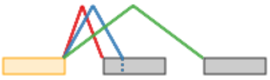	23 0 31
2463	HIPK1	ENSG00000163349:114515646-114520426:target	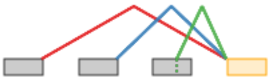	23 8 0 34
2464	YY1AP1	ENSG00000163374:155642063-155642548:target	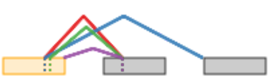	33 0 34
2465	ENSG00000163386	ENSG00000163386:145296357-145296571:source	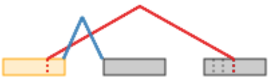	38 0 38
2466	ENSG00000163386	ENSG00000163386:145323654-145323705:source	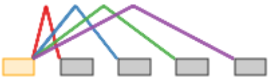	11 53 0 66
2467	ATP1A1	ENSG00000163399:116926636-116926746:target	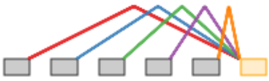	29 0 29
2468	ATP1A1	ENSG00000163399:116936153-116936345:source	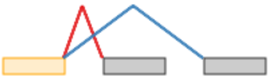	53 0 53
2469	ATP1A1	ENSG00000163399:116939220-116939356:target	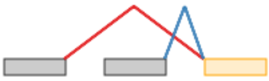	53 0 53
2470	SLC15A2	ENSG00000163406:121659678-121662949:target	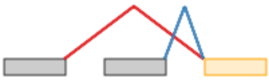	23 0 23
2471	ARPC2	ENSG00000163466:219090642-219090677:source	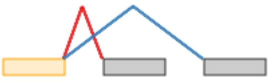	25 0 25
2472	ARPC2	ENSG00000163466:219093461-219094026:target	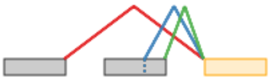	19 0 21
2473	ARPC2	ENSG00000163466:219103387-219103918:source	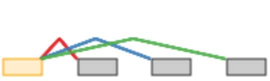	46 0 48
2474	ARPC2	ENSG00000163466:219103387-219103918:target	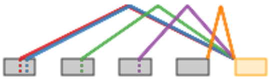	15 5 5 0 38
2475	ARPC2	ENSG00000163466:219110143-219110269:target	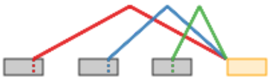	49 0 49
2476	STT3B	ENSG00000163527:31661168-31661322:source	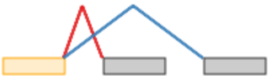	48 0 48
2477	SUCLG1	ENSG00000163541:84652538-84654060:target	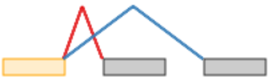	29 0 29
2478	SNHG16	ENSG00000163597:74553848-74553939:source	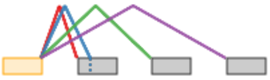	36 0 35
2479	SNHG16	ENSG00000163597:74557370-74557484:target	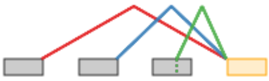	22 0 24
#	Gene	LSV ID	LSV Type	← More in Healthy | More in ALL →

#	Gene	LSV ID	LSV Type	← More in Healthy | More in ALL →
2480	RYBP	ENSG00000163602:72495647-72496069:target	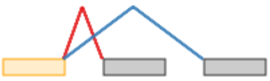	30 0 30
2481	PPP4R2	ENSG00000163605:73113154-73113297:target	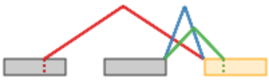	30 0 28
2482	ATXN7	ENSG00000163635:63981986-63982312:source	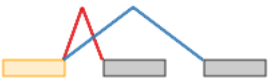	31 0 31
2483	ATXN7	ENSG00000163635:63985126-63989138:target	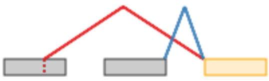	35 0 35
2484	PSMD6	ENSG00000163636:64003997-64004383:target	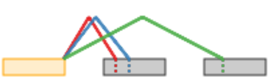	45 0 42
2485	PPM1K	ENSG00000163644:89196076-89198719:source	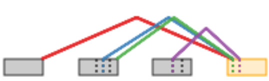	25 0 17
2486	PPM1K	ENSG00000163644:89205558-89205921:target	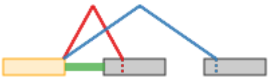	28 0 25
2487	CCNL1	ENSG00000163660:156869806-156870290:target	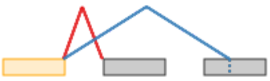	33 0 33
2488	CCNL1	ENSG00000163660:156876086-156877044:target	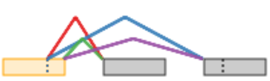	16 0 20
2489	SLMAP	ENSG00000163681:57850275-57850443:source	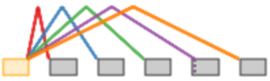	22 0 14
2490	SLMAP	ENSG00000163681:57875718-57876933:source	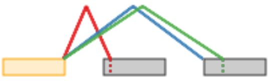	40 0 39
2491	U2SURP	ENSG00000163714:142741695-142742237:source	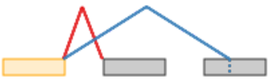	59 0 59
2492	U2SURP	ENSG00000163714:142744624-142746112:target	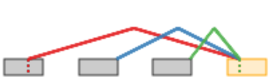	66 0 66
2493	MTMR14	ENSG00000163719:9714369-9714442:source	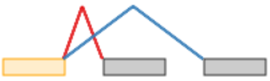	20 0 20
2494	MTMR14	ENSG00000163719:9719001-9719071:source	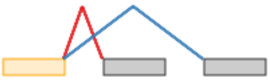	41 0 41
2495	MTMR14	ENSG00000163719:9724862-9725412:target	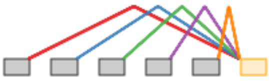	20 23 0 46
2496	MTMR14	ENSG00000163719:9730628-9730766:source	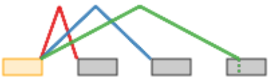	23 0 28
2497	MTMR14	ENSG00000163719:9739395-9739550:target	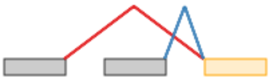	33 0 33
2498	TOPBP1	ENSG00000163781:133372189-133372368:target	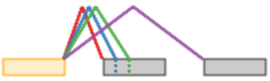	10 0 22
2499	RYK	ENSG00000163785:133913926-133914183:target	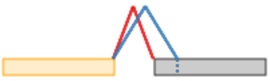	30 0 30

Gene LSV ID LSV Type ← More in Healthy | More in ALL →

#	Gene	LSV ID	LSV Type	← More in Healthy | More in ALL →
2500	WDR43	ENSG00000163811:29159788-29159819:source	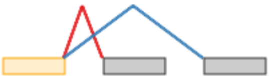	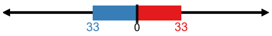 33 0 33
2501	WDR43	ENSG00000163811:29164327-29164440:target	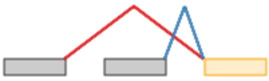	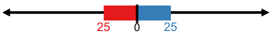 25 0 25
2502	RPN1	ENSG00000163902:128345576-128346109:source	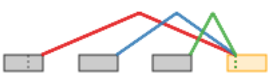	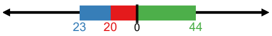 23 20 0 44
2503	RPN1	ENSG00000163902:128350791-128351000:target	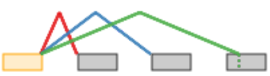	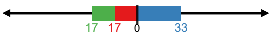 17 17 0 33
2504	SENP2	ENSG00000163904:185307902-185307957:source	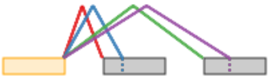	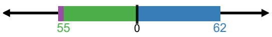 55 0 62
2505	SENP2	ENSG00000163904:185316746-185316812:target	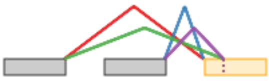	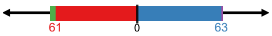 61 0 63
2506	SENP2	ENSG00000163904:185335268-185335399:source	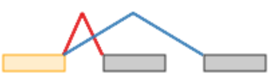	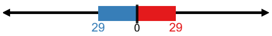 29 0 29
2507	SENP2	ENSG00000163904:185337087-185337307:source	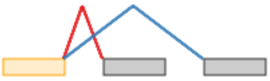	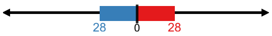 28 0 28
2508	SENP2	ENSG00000163904:185341786-185341870:target	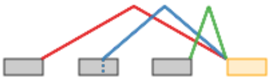	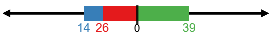 14 26 0 39
2509	BAP1	ENSG00000163930:52436795-52436956:source	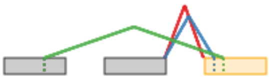	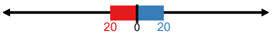 20 0 20
2510	BAP1	ENSG00000163930:52439781-52439928:target	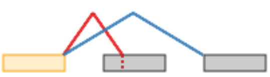	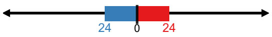 24 0 24
2511	FAM208A	ENSG00000163946:56694759-56694862:target	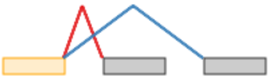	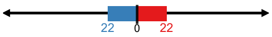 22 0 22
2512	SLBP	ENSG00000163950:1705323-1705427:source	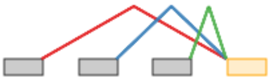	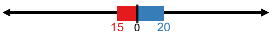 15 0 20
2513	DNAJB14	ENSG00000164031:100824883-100825055:target	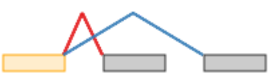	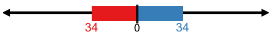 34 0 34
2514	WDR82	ENSG00000164091:52294401-52294517:source	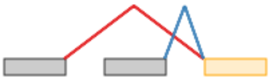	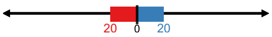 20 0 20
2515	WDR82	ENSG00000164091:52300968-52302154:target	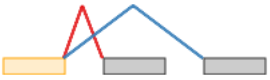	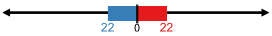 22 0 22
2516	OTUD4	ENSG00000164164:146077088-146077148:target	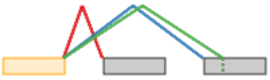	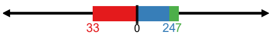 33 0 24 7
2517	NIPBL	ENSG00000164190:36970978-36971138:source	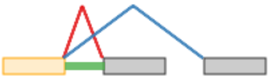	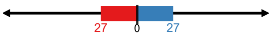 27 0 27
2518	NIPBL	ENSG00000164190:36995252-36995906:source	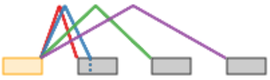	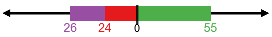 26 24 0 55
2519	NIPBL	ENSG00000164190:37000919-37000990:target	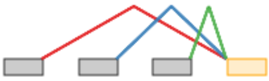	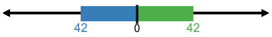 42 0 42
#	Gene	LSV ID	LSV Type	← More in Healthy | More in ALL →

#	Gene	LSV ID	LSV Type	← More in Healthy | More in ALL →
2520	NIPBL	ENSG00000164190:37057288-37057434:source	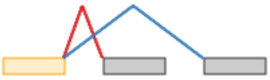	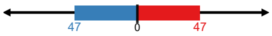
2521	NIPBL	ENSG00000164190:37060946-37061120:target	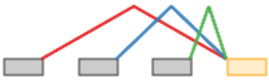	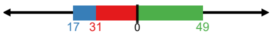
2522	ERAP2	ENSG00000164308:96235825-96235949:source	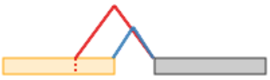	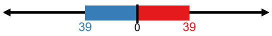
2523	ERAP2	ENSG00000164308:96244665-96244821:source	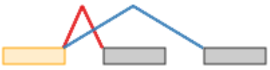	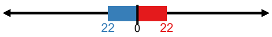
2524	ERAP2	ENSG00000164308:96248341-96248502:target	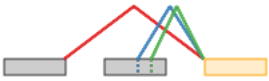	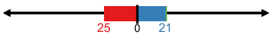
2525	PAPD4	ENSG00000164329:78944440-78944516:source	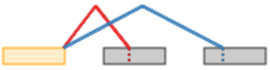	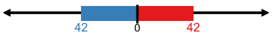
2526	PAPD4	ENSG00000164329:78952704-78952824:target	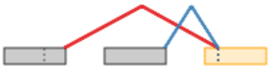	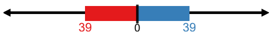
2527	EBF1	ENSG00000164330:158122928-158126150:source	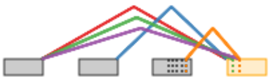	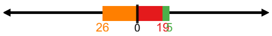
2528	EBF1	ENSG00000164330:158250184-158250325:target	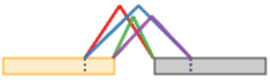	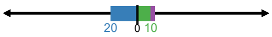
2529	EBF1	ENSG00000164330:158267037-158267118:source	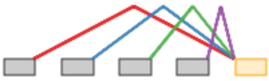	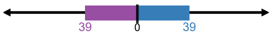
2530	EBF1	ENSG00000164330:158500404-158500472:target	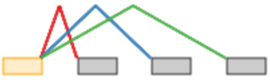	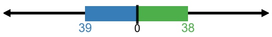
2531	EBF1	ENSG00000164330:158523982-158524138:source	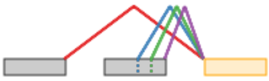	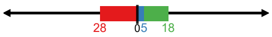
2532	GFM2	ENSG00000164347:74021766-74021951:source	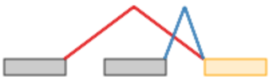	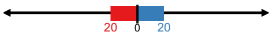
2533	GFM2	ENSG00000164347:74028847-74029213:target	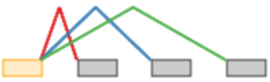	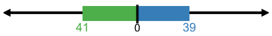
2534	CREBRF	ENSG00000164463:172513504-172513629:target	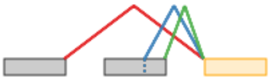	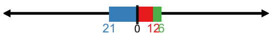
2535	CREBRF	ENSG00000164463:172560633-172566291:target	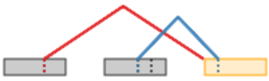	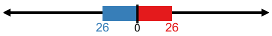
2536	TRA2A	ENSG00000164548:23546776-23547154:target	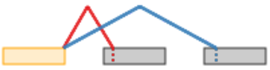	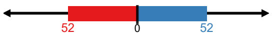
2537	TRA2A	ENSG00000164548:23561326-23561459:source	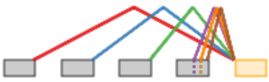	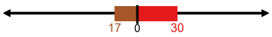
2538	TRA2A	ENSG00000164548:23571408-23571660:target	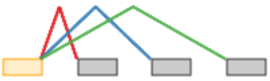	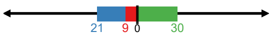
2539	RP9	ENSG00000164610:33139750-33140173:source	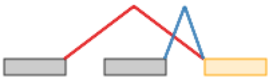	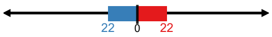
#	Gene	LSV ID	LSV Type	← More in Healthy | More in ALL →

#	Gene	LSV ID	LSV Type	← More in Healthy | More in ALL →
2540	CAMLG	ENSG00000164615:134076753-134077213:source	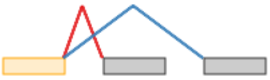	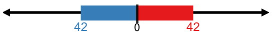
2541	CAMLG	ENSG00000164615:134086449-134087847:target	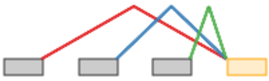	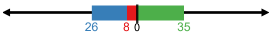
2542	CDCA7L	ENSG00000164649:21947748-21948125:source	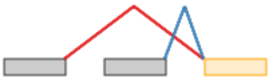	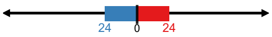
2543	CDCA7L	ENSG00000164649:21956372-21956515:target	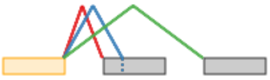	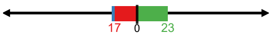
2544	MIOS	ENSG00000164654:7612067-7613400:target	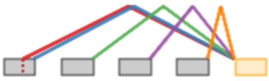	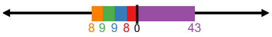
2545	MIOS	ENSG00000164654:7628129-7628194:source	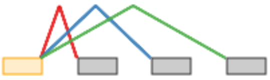	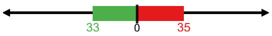
2546	MIOS	ENSG00000164654:7635888-7636092:target	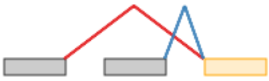	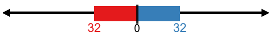
2547	MED30	ENSG00000164758:118540890-118541144:source	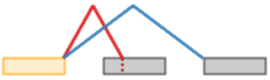	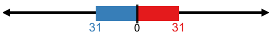
2548	MED30	ENSG00000164758:118552122-118552501:target	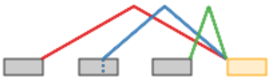	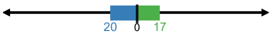
2549	FASTK	ENSG00000164896:150773711-150773919:source	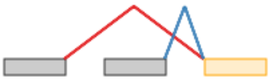	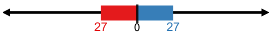
2550	PSIP1	ENSG00000164985:15478475-15478550:source	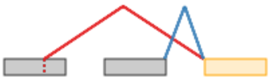	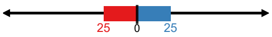
2551	UBAP1	ENSG00000165006:34179003-34179238:source	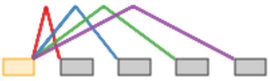	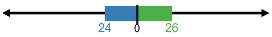
2552	UBAP1	ENSG00000165006:34220906-34220946:target	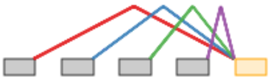	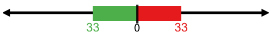
2553	HNRNPK	ENSG00000165119:86584411-86585246:target	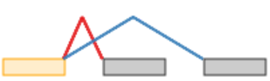	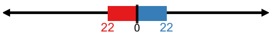
2554	HNRNPK	ENSG00000165119:86586188-86586271:source	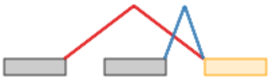	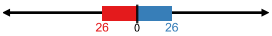
2555	HNRNPK	ENSG00000165119:86586797-86587104:target	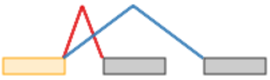	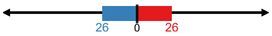
2556	STRBP	ENSG00000165209:125890493-125890537:target	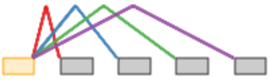	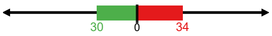
2557	STRBP	ENSG00000165209:125920611-125920708:source	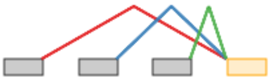	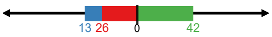
2558	STRBP	ENSG00000165209:125921375-125921486:source	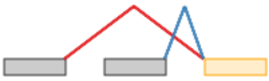	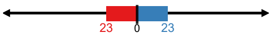
2559	STRBP	ENSG00000165209:125923256-125923347:target	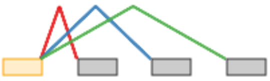	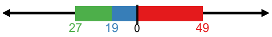

Gene LSV ID LSV Type ← More in Healthy | More in ALL →

#	Gene	LSV ID	LSV Type	← More in Healthy | More in ALL →
2560	STRBP	ENSG00000165209:125932181-125932325:target		30 0 39
2561	STRBP	ENSG00000165209:125946411-125946577:source		25 0 24
2562	GAPVD1	ENSG00000165219:128094789-128094908:source		66 0 66
2563	GAPVD1	ENSG00000165219:128098988-128099374:target		53 0 53
2564	GAPVD1	ENSG00000165219:128121699-128122126:source		29 0 29
2565	GAPVD1	ENSG00000165219:128124885-128129486:target		37 0 37
2566	ARHGAP12	ENSG00000165322:32120667-32120728:source		21 0 18
2567	WRN	ENSG00000165392:31014884-31015046:source		41 0 41
2568	MARCH8	ENSG00000165406:45984815-45984865:target		12 13 0 30
2569	SUGT1	ENSG00000165416:53236784-53236837:target		21 0 21
2570	INPPL1	ENSG00000165458:71944699-71944788:target		26 0 26
2571	PCF11	ENSG00000165494:82879470-82881037:target		5 29 0 35
2572	KLHDC2	ENSG00000165516:50246466-50246828:source		24 0 28
2573	NSD1	ENSG00000165671:176673679-176673797:source		24 0 24
2574	NSD1	ENSG00000165671:176675182-176675325:target		45 0 45
2575	NSD1	ENSG00000165671:176686990-176687169:source		26 0 26
2576	NSD1	ENSG00000165671:176694563-176694719:target		20 0 29
2577	GHITM	ENSG00000165678:85903751-85903862:source		14 17 0 34
2578	GHITM	ENSG00000165678:85908486-85908594:target		32 0 32
2579	PMPCA	ENSG00000165688:139307262-139307344:source		62 0 62

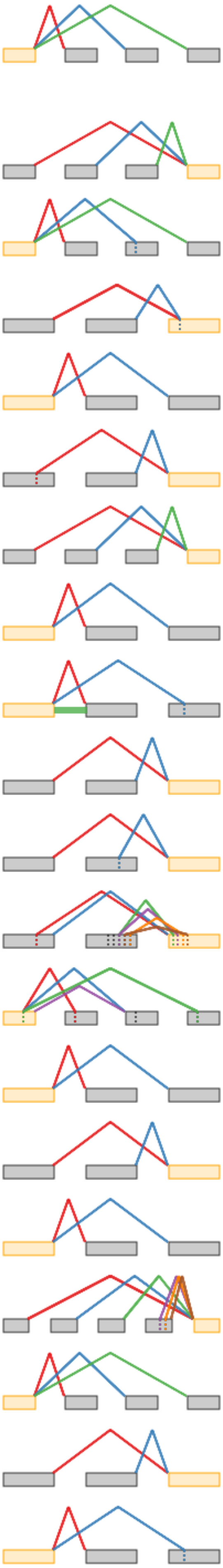

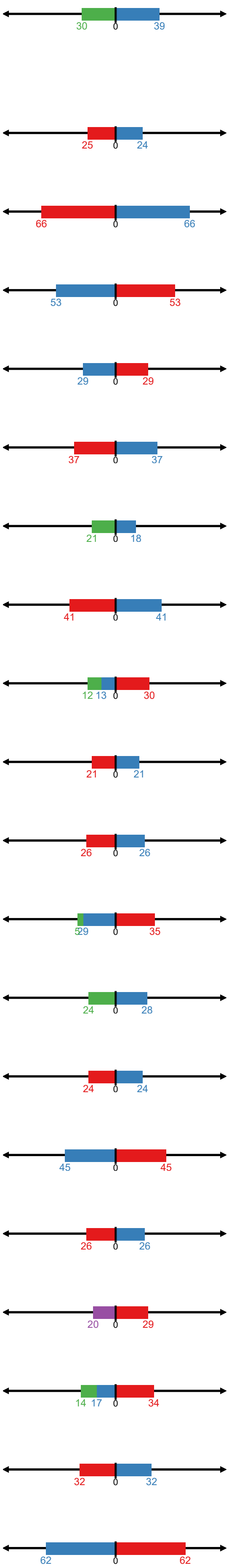

| # | Gene | LSV ID | LSV Type | ← More in Healthy \| More in ALL → |

#	Gene	LSV ID	LSV Type	← More in Healthy | More in ALL →
2580	PMPCA	ENSG00000165688:139310519-139311007:target		46 0 46
2581	DDX21	ENSG00000165732:70729957-70730106:source		5 23 0 33
2582	DDX21	ENSG00000165732:70733301-70733420:target		31 0 31
2583	TMEM55B	ENSG00000165782:20926545-20926861:source		20 0 20
2584	TMEM55B	ENSG00000165782:20927547-20927751:target		26 0 26
2585	TAF1D	ENSG00000166012:93470230-93470405:target		10 20 0 34
2586	CEP57	ENSG00000166037:95558847-95561191:target		54 0 9 42
2587	RAB8B	ENSG00000166128:63481668-63481947:source		19 0 21
2588	NDUFB8	ENSG00000166136:102285806-102286355:target		11 15 0 33
2589	BRD7	ENSG00000166164:50355893-50355949:source		37 0 37
2590	BRD7	ENSG00000166164:50359659-50359794:target		41 0 41
2591	NOLC1	ENSG00000166197:103916776-103916831:source		31 0 30
2592	NOLC1	ENSG00000166197:103916943-103917085:target		33 0 34
2593	COPS2	ENSG00000166200:49431455-49431850:target		27 0 27
2594	ARIH1	ENSG00000166233:72853793-72853890:source		29 0 29
2595	CUL5	ENSG00000166266:107916997-107917220:source		27 0 27
2596	CUL5	ENSG00000166266:107923387-107923528:target		30 0 30
2597	ILK	ENSG00000166333:6629831-6630020:source		60 13 0 75
2598	ILK	ENSG00000166333:6629831-6630020:target		77 0 77
2599	ILK	ENSG00000166333:6631139-6631266:target		51 10 0 64
#	Gene	LSV ID	LSV Type	← More in Healthy | More in ALL →

#	Gene	LSV ID	LSV Type	← More in Healthy | More in ALL →
2600	TAF10	ENSG00000166337:6627526-6632242:source	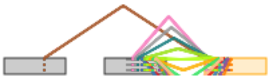	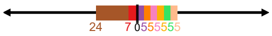
2601	TPP1	ENSG00000166340:6636673-6636991:source	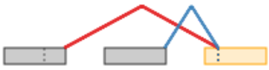	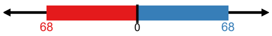
2602	TPP1	ENSG00000166340:6637546-6637734:target	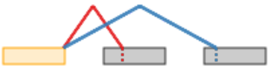	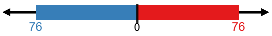
2603	TPP1	ENSG00000166340:6638857-6639007:source	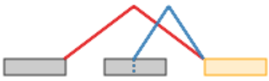	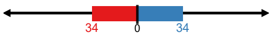
2604	ATP9B	ENSG00000166377:76873241-76873354:source	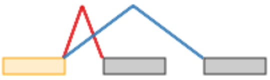	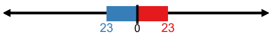
2605	KIAA0355	ENSG00000166398:34821094-34821252:source	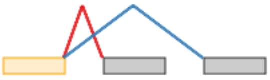	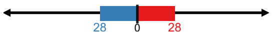
2606	KIAA0355	ENSG00000166398:34830772-34830897:target	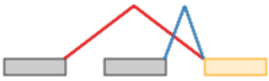	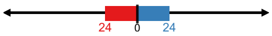
2607	TMX3	ENSG00000166479:66350162-66350218:target	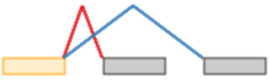	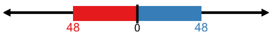
2608	MCM7	ENSG00000166508:99697637-99697716:source	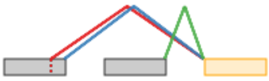	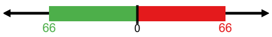
2609	RIMKLB	ENSG00000166532:8850496-8850893:source	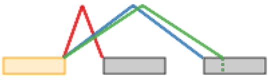	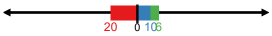
2610	RIMKLB	ENSG00000166532:8925917-8929787:source	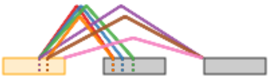	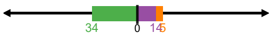
2611	RIMKLB	ENSG00000166532:8930186-8930813:source	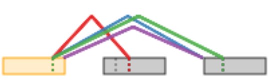	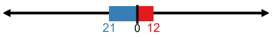
2612	RIMKLB	ENSG00000166532:8933171-8933335:target	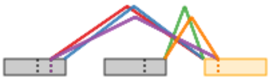	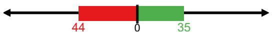
2613	ZNF592	ENSG00000166716:85307938-85308046:source	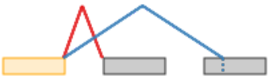	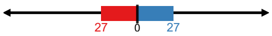
2614	CASC4	ENSG00000166734:44705534-44705741:target	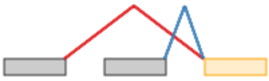	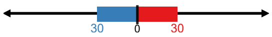
2615	AP1G1	ENSG00000166747:71792711-71792824:target	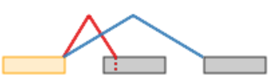	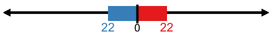
2616	C16orf45	ENSG00000166780:15715590-15715768:source	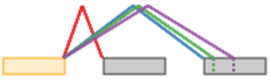	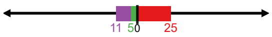
2617	KIAA0430	ENSG00000166783:15715350-15715768:source	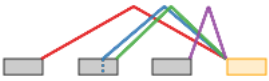	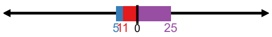
2618	KIAA0430	ENSG00000166783:15718626-15718784:target	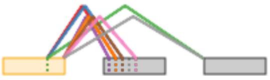	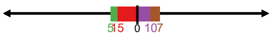
2619	FAM96A	ENSG00000166797:64380886-64381050:target	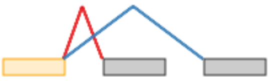	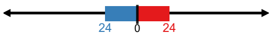

Gene LSV ID LSV Type ← More in Healthy | More in ALL →

#	Gene	LSV ID	LSV Type	← More in Healthy | More in ALL →
2620	VPS39	ENSG00000166887:42483459-42483758:source	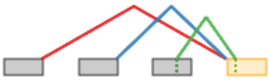	25 0 24
2621	VPS39	ENSG00000166887:42483459-42483758:target	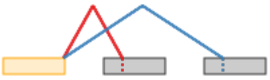	60 0 60
2622	STAT6	ENSG00000166888:57501014-57501097:source	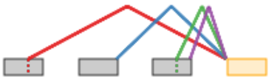	33 0 37
2623	PATL1	ENSG00000166889:59422996-59423213:source	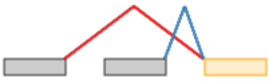	24 0 24
2624	PATL1	ENSG00000166889:59423972-59424097:target	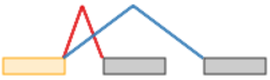	21 0 21
2625	MTMR10	ENSG00000166912:31260123-31260414:target	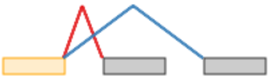	22 0 22
2626	YWHAB	ENSG00000166913:43530172-43530474:source	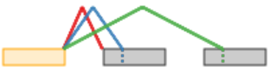	48 0 44
2627	YWHAB	ENSG00000166913:43532460-43532757:target	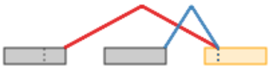	41 0 41
2628	CCNDBP1	ENSG00000166946:43478018-43478077:source	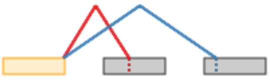	64 0 64
2629	CCNDBP1	ENSG00000166946:43480650-43481664:source	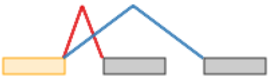	63 0 63
2630	CCNDBP1	ENSG00000166946:43480650-43481664:target	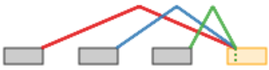	26 0 27
2631	CCNDBP1	ENSG00000166946:43482523-43482596:target	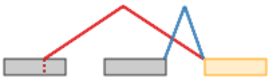	65 0 65
2632	EPB42	ENSG00000166947:43478018-43478077:source	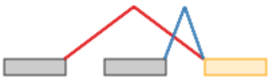	64 0 64
2633	EPB42	ENSG00000166947:43481397-43481478:source	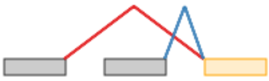	63 0 63
2634	EPB42	ENSG00000166947:43481397-43481478:target	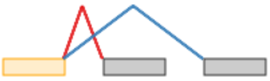	26 0 26
2635	EPB42	ENSG00000166947:43482523-43482596:target	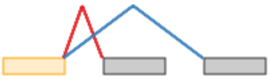	65 0 65
2636	MARS	ENSG00000166986:57882469-57882892:source	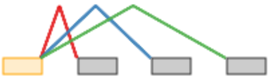	23 0 23
2637	MARS	ENSG00000166986:57883050-57883128:source	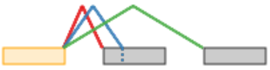	27 0 29
2638	MARS	ENSG00000166986:57883313-57883566:source	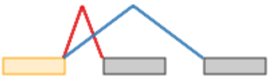	47 0 47
2639	MARS	ENSG00000166986:57883990-57884017:target	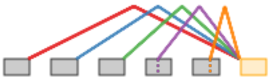	26 1015 0 51

| # | Gene | LSV ID | LSV Type | ← More in Healthy \| More in ALL → |

#	Gene	LSV ID	LSV Type	← More in Healthy | More in ALL →
2640	MARS	ENSG00000166986:57905791-57905937:source		37 0 34
2641	MARS	ENSG00000166986:57906274-57906747:target		32 0 32
2642	MARS	ENSG00000166986:57908933-57909450:source		26 0 26
2643	MARS	ENSG00000166986:57909703-57909774:source		20 0 20
2644	MARS	ENSG00000166986:57910028-57910120:target		32 0 32
2645	MARS	ENSG00000166986:57911052-57911378:source		42 0 6 36
2646	FAM102A	ENSG00000167106:130715548-130715883:source		33 0 33
2647	FAM129C	ENSG00000167483:17643072-17643262:source		29 0 37
2648	FAM129C	ENSG00000167483:17643072-17643262:target		26 0 26
2649	ANKRD11	ENSG00000167522:89337225-89337317:target		20 0 20
2650	ANKRD11	ENSG00000167522:89357380-89357591:source		22 0 22
2651	KMT2D	ENSG00000167548:49421792-49421924:source		21 0 21
2652	NFKBID	ENSG00000167604:36387628-36387733:source		30 0 30
2653	LENG8	ENSG00000167615:54970644-54970660:source		23 0 23
2654	TRAPPC9	ENSG00000167632:141370149-141370292:target		22 0 22
2655	SRP68	ENSG00000167881:74068389-74068734:target		15 0 21
2656	DDB1	ENSG00000167986:61077268-61077432:source		51 0 51
2657	DDB1	ENSG00000167986:61079256-61079367:target		16 51 0 69
2658	ATG16L2	ENSG00000168010:72534940-72535167:target		30 0 8 20
2659	CTNNB1	ENSG00000168036:41274832-41274935:target		26 0 26

Gene LSV ID LSV Type ← More in Healthy | More in ALL →

#	Gene	LSV ID	LSV Type	← More in Healthy | More in ALL →
2660	CTNNB1	ENSG00000168036:41280624-41280845:source		29 0 29
2661	SF1	ENSG00000168066:64535039-64535316:target		19 0 20
2662	RAB4A	ENSG00000168118:229434724-229434819:source		20 0 20
2663	SETD5	ENSG00000168137:9466151-9466210:source		22 0 22
2664	SETD5	ENSG00000168137:9466151-9466210:target		21 0 21
2665	SETD5	ENSG00000168137:9475961-9476169:source		35 0 37
2666	SETD5	ENSG00000168137:9477412-9477590:target		39 0 43
2667	SETD5	ENSG00000168137:9483812-9483929:source		28 0 28
2668	SETD5	ENSG00000168137:9486732-9486984:target		36 0 36
2669	SETD5	ENSG00000168137:9495423-9495552:source		22 0 22
2670	RBPJ	ENSG00000168214:26364058-26364250:source		20 0 20
2671	RBPJ	ENSG00000168214:26387975-26388013:target		33 0 28
2672	LMBRD1	ENSG00000168216:70490386-70490446:target		36 0 36
2673	TTC39C	ENSG00000168234:21660549-21660903:target		20 0 20
2674	PXK	ENSG00000168297:58368241-58368427:source		17 11 0 31
2675	PXK	ENSG00000168297:58398628-58400254:source		40 0 40
2676	PXK	ENSG00000168297:58410479-58411748:target		66 0 54
2677	PCMTD1	ENSG00000168300:52744004-52744127:target		48 0 48
2678	ARF4	ENSG00000168374:57569625-57569787:source		30 0 30
2679	ARF4	ENSG00000168374:57570111-57570191:target		29 0 30

| # | Gene | LSV ID | LSV Type | ← More in Healthy \| More in ALL → |

#	Gene	LSV ID	LSV Type	← More in Healthy | More in ALL →
2680	SEPT2	ENSG00000168385:242274541-242274627:source	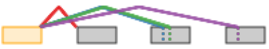	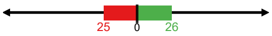
2681	SEPT2	ENSG00000168385:242275258-242275796:source	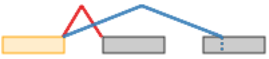	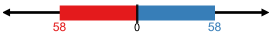
2682	TAP1	ENSG00000168394:32814845-32814981:source	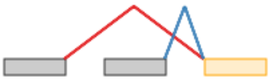	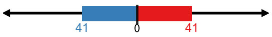
2683	TAP1	ENSG00000168394:32816429-32816617:source	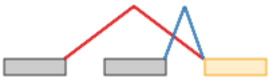	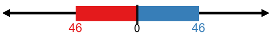
2684	TAP1	ENSG00000168394:32818097-32818294:target	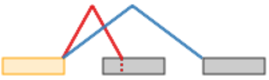	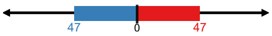
2685	ATG4B	ENSG00000168397:242590679-242590750:source	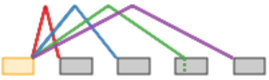	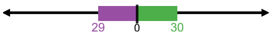
2686	ATG4B	ENSG00000168397:242593961-242594417:target	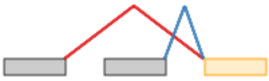	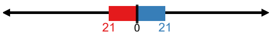
2687	ATXN2L	ENSG00000168488:28837561-28837926:source	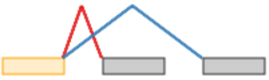	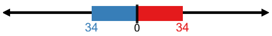
2688	ATXN2L	ENSG00000168488:28840722-28840813:target	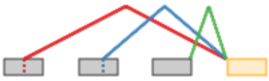	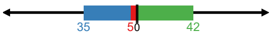
2689	ATXN2L	ENSG00000168488:28843508-28843720:source	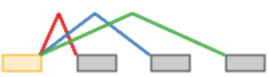	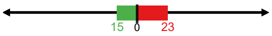
2690	ATXN2L	ENSG00000168488:28843817-28843888:source	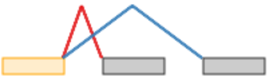	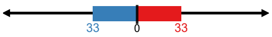
2691	ATXN2L	ENSG00000168488:28844380-28844675:target	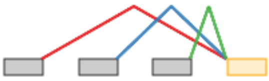	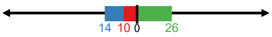
2692	ATXN2L	ENSG00000168488:28847254-28847497:source	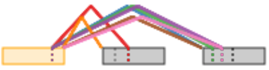	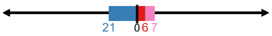
2693	ATXN2L	ENSG00000168488:28848049-28848558:target	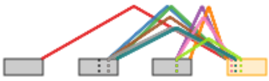	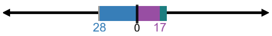
2694	FNTA	ENSG00000168522:42931790-42935584:source	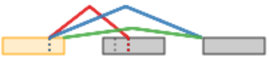	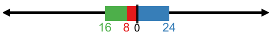
2695	STAT3	ENSG00000168610:40475591-40475643:target	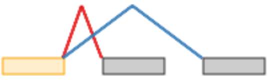	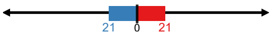
2696	AHCYL1	ENSG00000168710:110562170-110562248:source	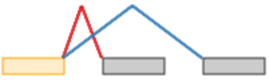	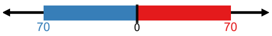
2697	AHCYL1	ENSG00000168710:110562170-110562248:target	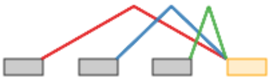	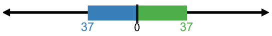
2698	AHCYL1	ENSG00000168710:110564296-110566357:target	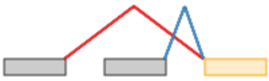	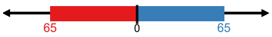
2699	ZNF608	ENSG00000168916:123972608-123973599:source	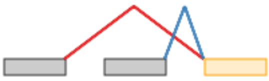	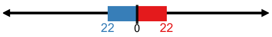

Gene LSV ID LSV Type ← More in Healthy | More in ALL →

#	Gene	LSV ID	LSV Type	← More in Healthy | More in ALL →
2700	ZNF608	ENSG00000168916:123974826-123974907:target		24 0 24
2701	ZNF608	ENSG00000168916:123985303-123985390:target		21 0 11
2702	CEP120	ENSG00000168944:122718680-122718776:source		24 0 24
2703	CEP120	ENSG00000168944:122722212-122722361:target		29 0 29
2704	MFF	ENSG00000168958:228205008-228205277:source		20 0 6 5
2705	HNRNPH1	ENSG00000169045:179045145-179045900:source		42 0 44
2706	HNRNPH1	ENSG00000169045:179047676-179048036:target		44 0 43
2707	HNRNPH1	ENSG00000169045:179049850-179050188:source		20 0 19
2708	HNRNPH1	ENSG00000169045:179050282-179050722:target		21 0 18
2709	XPO6	ENSG00000169180:28112376-28113009:target		23 0 23
2710	XPO6	ENSG00000169180:28177829-28177906:source		27 0 27
2711	SLC50A1	ENSG00000169241:155109304-155109607:source		20 0 20
2712	NPIPB3	ENSG00000169246:21413558-21414516:source		11 0 21
2713	NPIPB3	ENSG00000169246:21415177-21415374:source		41 0 30
2714	NPIPB3	ENSG00000169246:21415177-21415374:target		13 16 0 28
2715	NPIPB3	ENSG00000169246:21415375-21415557:target		38 0 8 27
2716	NPIPB3	ENSG00000169246:21416021-21416289:target		38 0 35
2717	NMD3	ENSG00000169251:160960296-160960441:target		30 0 30
2718	SIN3A	ENSG00000169375:75672970-75673429:source		31 0 31
2719	SIN3A	ENSG00000169375:75676349-75676778:target		36 0 38

| # | Gene | LSV ID | LSV Type | ← More in Healthy \| More in ALL → |

#	Gene	LSV ID	LSV Type	← More in Healthy | More in ALL →
2720	SIN3A	ENSG00000169375:75692381-75692497:source	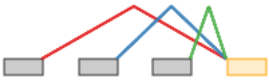	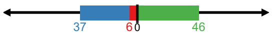
2721	SIN3A	ENSG00000169375:75694193-75694311:target	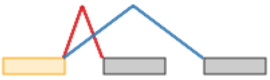	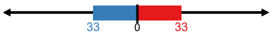
2722	SIN3A	ENSG00000169375:75722410-75722749:source	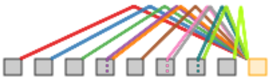	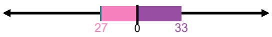
2723	PTK2	ENSG00000169398:141678368-141678527:source	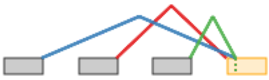	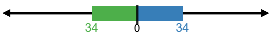
2724	PTK2	ENSG00000169398:141684397-141684503:target	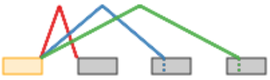	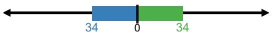
2725	PLEKHA2	ENSG00000169499:38759316-38759587:source	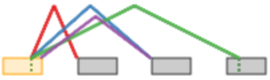	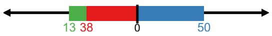
2726	PLEKHA2	ENSG00000169499:38775425-38775588:target	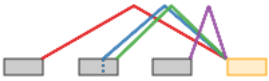	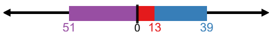
2727	ZEB2	ENSG00000169554:145153979-145154159:source	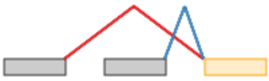	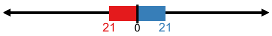
2728	ZEB2	ENSG00000169554:145158766-145158874:target	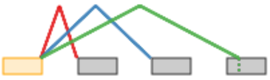	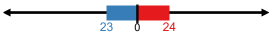
2729	ZEB2	ENSG00000169554:145277506-145277907:target	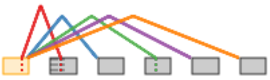	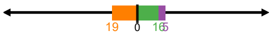
2730	INO80E	ENSG00000169592:30006615-30007712:source	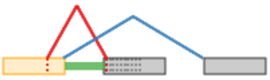	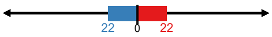
2731	TAPT1	ENSG00000169762:16168256-16168495:source	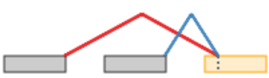	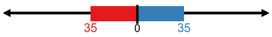
2732	TAPT1	ENSG00000169762:16175827-16176171:target	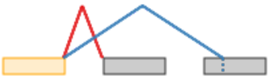	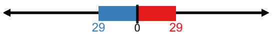
2733	CHD3	ENSG00000170004:7792982-7793094:target	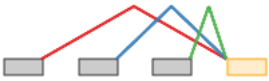	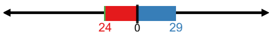
2734	SIMC1	ENSG00000170085:175749276-175749818:source	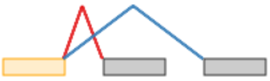	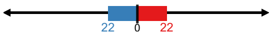
2735	UBE2E1	ENSG00000170142:23932000-23932807:target	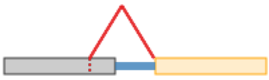	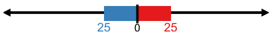
2736	HNRNPA3	ENSG00000170144:178081588-178081668:source	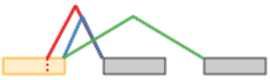	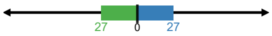
2737	HNRNPA3	ENSG00000170144:178083765-178083887:target	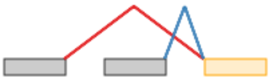	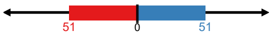
2738	USP38	ENSG00000170185:144124561-144124719:target	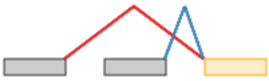	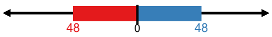
2739	USP38	ENSG00000170185:144127186-144127379:source	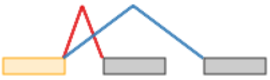	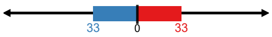

Gene LSV ID LSV Type ← More in Healthy | More in ALL →

#	Gene	LSV ID	LSV Type	← More in Healthy | More in ALL →
2740	USP38	ENSG00000170185:144133471-144133577:target	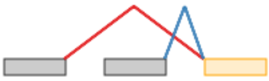	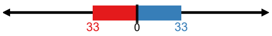 33 0 33
2741	ADPRM	ENSG00000170222:10608217-10609245:source	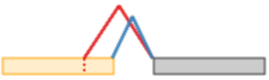	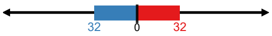 32 0 32
2742	USP47	ENSG00000170242:11862970-11863771:source	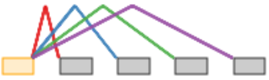	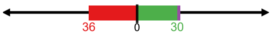 36 0 30
2743	RALGAPB	ENSG00000170471:37174886-37176749:source	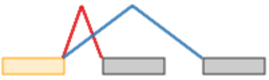	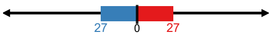 27 0 27
2744	RALGAPB	ENSG00000170471:37179714-37179866:target	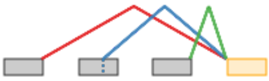	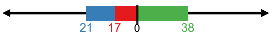 21 17 0 38
2745	MZB1	ENSG00000170476:138724306-138724544:source	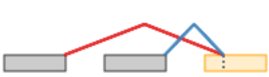	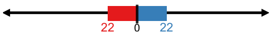 22 0 22
2746	MZB1	ENSG00000170476:138725369-138725770:target	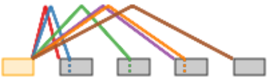	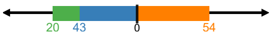 20 43 0 54
2747	SERPINB9	ENSG00000170542:2892067-2892222:source	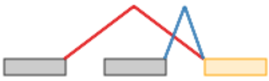	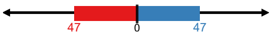 47 0 47
2748	SERPINB9	ENSG00000170542:2895625-2895742:target	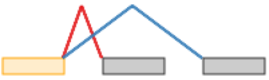	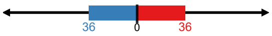 36 0 36
2749	STAT2	ENSG00000170581:56748203-56748291:target	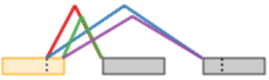	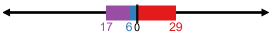 17 6 0 29
2750	KIF5B	ENSG00000170759:32337392-32337479:source	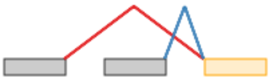	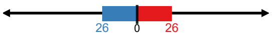 26 0 26
2751	AKAP13	ENSG00000170776:86207794-86207986:target	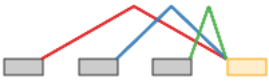	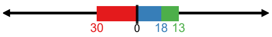 30 0 18 13
2752	AKAP13	ENSG00000170776:86236330-86236682:target	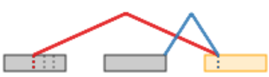	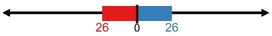 26 0 26
2753	AKAP13	ENSG00000170776:86260383-86260633:source	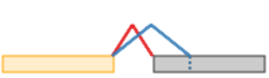	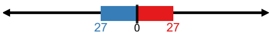 27 0 27
2754	AKAP13	ENSG00000170776:86283453-86283793:source	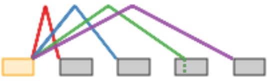	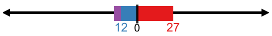 12 0 27
2755	KBTBD2	ENSG00000170852:32919128-32919554:source	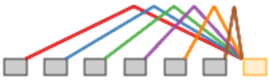	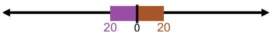 20 0 20
2756	KIAA0232	ENSG00000170871:6873301-6873409:target	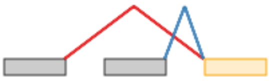	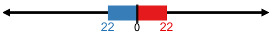 22 0 22
2757	MTSS1	ENSG00000170873:125567983-125568145:target	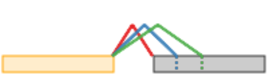	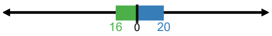 16 0 20
2758	MTSS1	ENSG00000170873:125568473-125568813:source	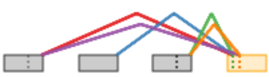	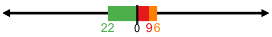 22 0 9 6
2759	TPT1-AS1	ENSG00000170919:45953410-45953470:source	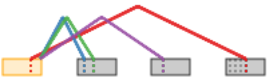	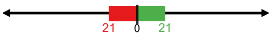 21 0 21

Gene LSV ID LSV Type ← More in Healthy | More in ALL →

#	Gene	LSV ID	LSV Type	← More in Healthy | More in ALL →
2760	PRKCE	ENSG00000171132:46070139-46070202:target		21 0 28
2761	PRKCE	ENSG00000171132:46234601-46234800:source		50 0 50
2762	CHD7	ENSG00000171316:61754406-61754611:source		23 0 23
2763	ASXL1	ENSG00000171456:31017141-31017234:source		55 0 55
2764	WIPF2	ENSG00000171475:38434437-38440388:target		25 0 25
2765	RAB4B-EGLN2	ENSG00000171570:41313714-41314103:target		41 0 41
2766	ZNF274	ENSG00000171606:58719464-58722075:source		45 13 0 58
2767	PIK3CD	ENSG00000171608:9770482-9770654:target		33 05 28
2768	PIK3CD	ENSG00000171608:9783183-9783350:source		31 0 31
2769	PIK3CD	ENSG00000171608:9784334-9784479:target		21 0 21
2770	BPTF	ENSG00000171634:65870933-65871136:source		37 0 22 15
2771	BPTF	ENSG00000171634:65889337-65889841:target		39 0 39
2772	BPTF	ENSG00000171634:65890150-65890281:source		24 0 29
2773	BPTF	ENSG00000171634:65960328-65960520:source		16 0 24
2774	ATF7IP	ENSG00000171681:14576695-14578407:source		20 0 20
2775	ATF7IP	ENSG00000171681:14587271-14587357:target		20 0 20
2776	HDAC3	ENSG00000171720:141005580-141005638:source		26 0 26
2777	CAMTA1	ENSG00000171735:6845384-6845635:source		22 0 21
2778	PTEN	ENSG00000171862:89692770-89693244:source		26 0 26
2779	PTEN	ENSG00000171862:89711875-89712016:target		24 0 24

| # | Gene | LSV ID | LSV Type | ← More in Healthy \| More in ALL → |

#	Gene	LSV ID	LSV Type	← More in Healthy | More in ALL →
2780	RNASEH1	ENSG00000171865:3599734-3599898:source		22 0 22
2781	JMJD1C	ENSG00000171988:64948928-64949206:target		25 5 0 32
2782	JMJD1C	ENSG00000171988:64953105-64953409:target		29 0 8 18
2783	JMJD1C	ENSG00000171988:64958273-64958487:source		29 0 29
2784	JMJD1C	ENSG00000171988:64966355-64968561:target		11 30 0 43
2785	SERF1A	ENSG00000172058:70197509-70197760:target		57 0 57
2786	SMN1	ENSG00000172062:70238545-70238697:target		29 0 29
2787	SMN1	ENSG00000172062:70241916-70242105:source		32 0 32
2788	SMN1	ENSG00000172062:70248266-70248867:target		27 0 28
2789	EIF2AK3	ENSG00000172071:88879036-88879487:source		14 6 0 30
2790	EIF2AK3	ENSG00000172071:88882273-88882342:target		11 14 0 24
2791	EIF2AK3	ENSG00000172071:88882948-88883060:target		26 0 26
2792	EIF2AK3	ENSG00000172071:88892790-88892923:target		21 0 21
2793	EIF2AK3	ENSG00000172071:88913242-88913371:target		21 0 21
2794	AFF1	ENSG00000172493:88052945-88053016:source		38 0 38
2795	AFF1	ENSG00000172493:88055623-88055846:target		25 0 25
2796	BANP	ENSG00000172530:88008654-88009289:source		6 0 22
2797	RASGRP1	ENSG00000172575:38794513-38794622:source		26 17 0 42
2798	RASGRP1	ENSG00000172575:38798041-38798121:target		46 0 46
2799	RASGRP1	ENSG00000172575:38852022-38852283:target		43 0 43

Gene LSV ID LSV Type ← More in Healthy | More in ALL →

#	Gene	LSV ID	LSV Type	← More in Healthy | More in ALL →
2800	KLHL6	ENSG00000172578:183211867-183212069:source		40 0 40
2801	KLHL6	ENSG00000172578:183225847-183226296:target		31 0 31
2802	MRPL52	ENSG00000172590:23299396-23299878:source		35 0 31
2803	MRPL52	ENSG00000172590:23302476-23302698:target		20 0 20
2804	ENSG00000172660	ENSG00000172660:34144720-34144759:target		31 0 31
2805	CBWD1	ENSG00000172785:151745-152078:target		49 0 33 13
2806	SP3	ENSG00000172845:174771187-174774985:source		35 0 41
2807	DMXL1	ENSG00000172869:118500202-118500371:source		24 0 24
2808	DMXL1	ENSG00000172869:118552596-118552682:source		20 0 20
2809	DMXL1	ENSG00000172869:118556167-118556288:target		25 0 25
2810	DMXL1	ENSG00000172869:118572009-118573109:source		49 17 0 66
2811	DMXL1	ENSG00000172869:118576114-118576166:target		52 0 52
2812	NADSYN1	ENSG00000172890:71185441-71186668:source		26 0 29
2813	OXSR1	ENSG00000172939:38266062-38266560:target		27 0 27
2814	KAT5	ENSG00000172977:65480393-65480790:source		32 0 31
2815	KAT5	ENSG00000172977:65481064-65481138:target		30 0 30
2816	RELA	ENSG00000173039:65423326-65423406:source		18 15 0 31
2817	RELA	ENSG00000173039:65426073-65426293:target		34 0 34
2818	RELA	ENSG00000173039:65428879-65429339:target		15 0 21
2819	KDM2A	ENSG00000173120:66982811-66983144:source		23 0 26

| # | Gene | LSV ID | LSV Type | ← More in Healthy \| More in ALL → |

#	Gene	LSV ID	LSV Type	← More in Healthy | More in ALL →
2820	KDM2A	ENSG00000173120:66985014-66985355:target	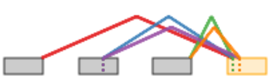	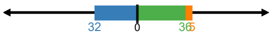
2821	KDM2A	ENSG00000173120:66999037-66999431:source	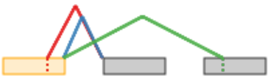	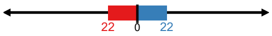
2822	PARP14	ENSG00000173193:122418237-122420482:source	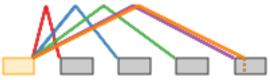	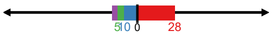
2823	PARP14	ENSG00000173193:122432112-122432420:target	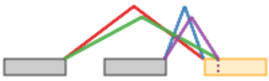	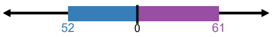
2824	PARP14	ENSG00000173193:122439102-122439235:source	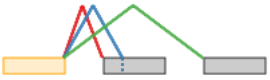	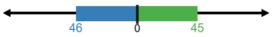
2825	PARP14	ENSG00000173193:122446659-122446833:target	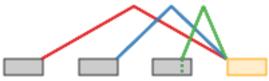	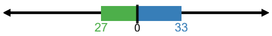
2826	PARP15	ENSG00000173200:122331888-122332115:source	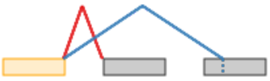	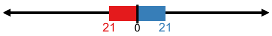
2827	GOLGB1	ENSG00000173230:121382046-121383461:source	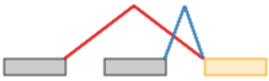	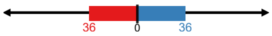
2828	GOLGB1	ENSG00000173230:121386323-121386457:target	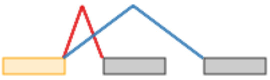	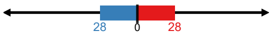
2829	MINOS1	ENSG00000173436:19952881-19956314:target	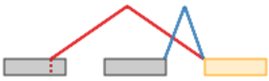	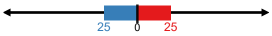
2830	SMARCC1	ENSG00000173473:47716965-47717078:source	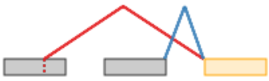	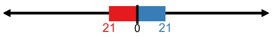
2831	SMARCC1	ENSG00000173473:47777524-47777616:target	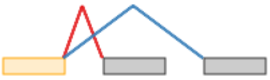	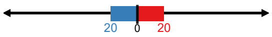
2832	CHD2	ENSG00000173575:93429399-93429484:source	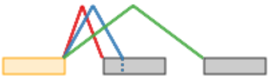	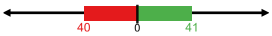
2833	CHD2	ENSG00000173575:93435234-93435302:target	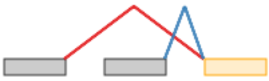	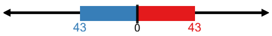
2834	CHD2	ENSG00000173575:93443058-93444054:source	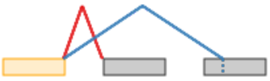	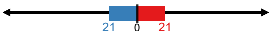
2835	CHD2	ENSG00000173575:93467548-93467782:target	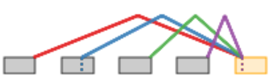	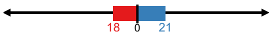
2836	CHD2	ENSG00000173575:93489268-93489642:source	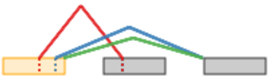	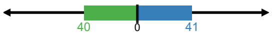
2837	CHD2	ENSG00000173575:93518109-93518180:source	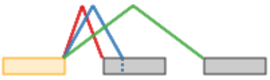	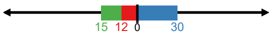
2838	CHD2	ENSG00000173575:93528728-93528903:source	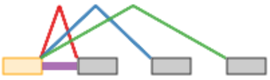	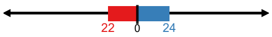
2839	CHD2	ENSG00000173575:93534706-93534747:source	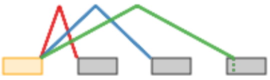	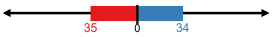

Gene LSV ID LSV Type ← More in Healthy | More in ALL →

#	Gene	LSV ID	LSV Type	← More in Healthy | More in ALL →
2840	CHD2	ENSG00000173575:93536089-93536344:target	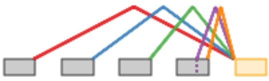	21 20 0 41
2841	RCE1	ENSG00000173653:66610306-66611111:source	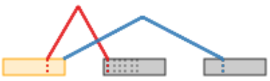	69 0 69
2842	UQCRH	ENSG00000173660:46775827-46775988:target	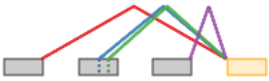	27 0 24
2843	AGFG1	ENSG00000173744:228389478-228389631:source	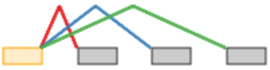	40 0 40
2844	AGFG1	ENSG00000173744:228395807-228395926:target	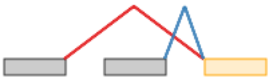	33 0 33
2845	AGFG1	ENSG00000173744:228416675-228416833:source	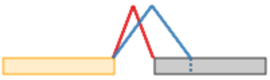	22 0 22
2846	CNP	ENSG00000173786:40125493-40129749:target	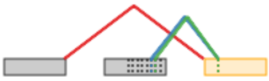	19 21 0 41
2847	EIF1	ENSG00000173812:39845137-39845517:source	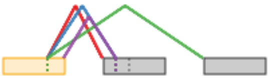	18 0 20
2848	RNF213	ENSG00000173821:78355086-78355988:source	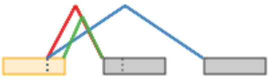	13 10 0 23
2849	RBM4	ENSG00000173933:66411221-66412103:source	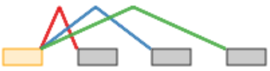	31 0 30
2850	RBM4	ENSG00000173933:66433327-66434153:target	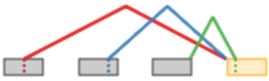	22 0 19
2851	ENSG00000174093	ENSG00000174093:36351796-36351996:source	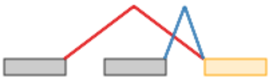	27 0 27
2852	LEMD3	ENSG00000174106:65639080-65639554:source	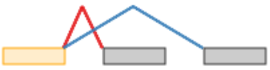	35 0 35
2853	LEMD3	ENSG00000174106:65639942-65642107:target	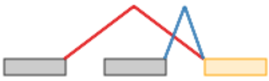	21 0 21
2854	MGA	ENSG00000174197:41990608-41991139:target	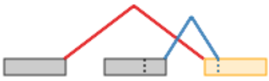	22 0 22
2855	PRPF8	ENSG00000174231:1580270-1580565:source	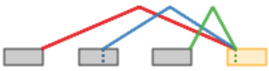	44 9 0 56
2856	PRPF8	ENSG00000174231:1581735-1581946:target	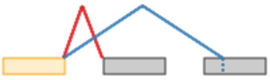	56 0 56
2857	PITPNA	ENSG00000174238:1438735-1438812:source	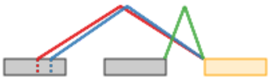	48 0 48
2858	PITPNA	ENSG00000174238:1441733-1442246:target	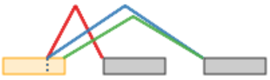	48 0 48
2859	DDX23	ENSG00000174243:49229904-49230049:target	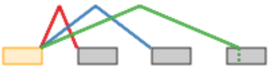	28 0 27

| # | Gene | LSV ID | LSV Type | ← More in Healthy \| More in ALL → |

#	Gene	LSV ID	LSV Type	← More in Healthy | More in ALL →
2860	C12orf76	ENSG00000174456:110468450-110468563:source	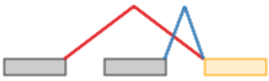	47 0 47
2861	C12orf76	ENSG00000174456:110471602-110471762:target	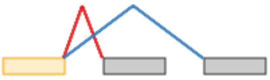	32 0 32
2862	DENND4A	ENSG00000174485:65998306-65998562:target	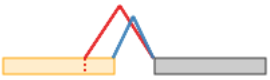	41 0 41
2863	RPL15	ENSG00000174748:23958622-23958935:source	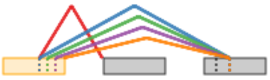	6 49 0 60
2864	RPL15	ENSG00000174748:23959196-23959538:target	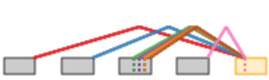	6 49 0 60
2865	CEP135	ENSG00000174799:56825848-56827091:source	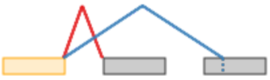	34 0 34
2866	CEP135	ENSG00000174799:56831032-56832746:source	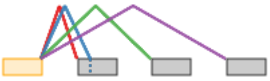	18 0 35
2867	CEP135	ENSG00000174799:56831032-56832746:target	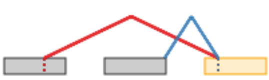	28 0 28
2868	DENND6A	ENSG00000174839:57678509-57678816:target	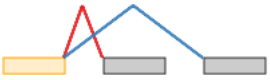	43 0 43
2869	DHX36	ENSG00000174953:153996191-153998458:target	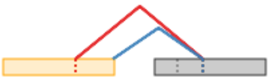	32 0 32
2870	DHX36	ENSG00000174953:154022883-154023126:source	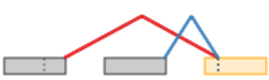	37 0 37
2871	DHX36	ENSG00000174953:154027442-154027612:target	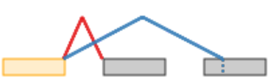	45 0 45
2872	ATR	ENSG00000175054:142168077-142168444:source	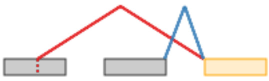	26 0 26
2873	ATR	ENSG00000175054:142266567-142266752:target	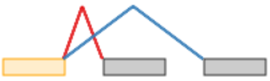	21 0 21
2874	TRAF6	ENSG00000175104:36516526-36516597:target	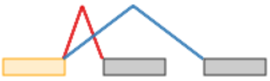	25 0 25
2875	PACS1	ENSG00000175115:65994975-65995055:source	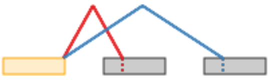	27 0 27
2876	PACS1	ENSG00000175115:65999265-65999747:target	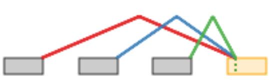	11 16 0 30
2877	DDIT3	ENSG00000175197:57911489-57911536:target	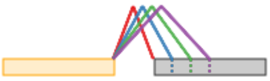	42 0 35 5
2878	PHYKPL	ENSG00000175309:177637554-177638971:target	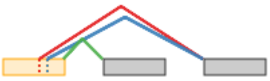	28 0 27
2879	PTPN2	ENSG00000175354:12817155-12817364:source	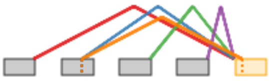	65 0 58

| # | Gene | LSV ID | LSV Type | ← More in Healthy \| More in ALL → |

#	Gene	LSV ID	LSV Type	← More in Healthy | More in ALL →
2880	PTPN2	ENSG00000175354:12836790-12836890:source		71 0 69
2881	SMAD2	ENSG00000175387:45391430-45391504:target		33 0 33
2882	CCDC14	ENSG00000175455:123667793-123668160:source		28 0 24
2883	POLD4	ENSG00000175482:67120364-67120631:source		11 12 0 20
2884	UCP2	ENSG00000175567:73687686-73687787:target		24 0 24
2885	RAB6A	ENSG00000175582:73431891-73431944:target		23 0 24
2886	MLXIP	ENSG00000175727:122620175-122620213:source		47 0 49
2887	MLXIP	ENSG00000175727:122622738-122622842:target		43 0 35
2888	CTDNEP1	ENSG00000175826:7149573-7149997:target		24 0 24
2889	UBE2O	ENSG00000175931:74385532-74387694:source		28 0 28
2890	UBE2O	ENSG00000175931:74387991-74388194:target		30 0 30
2891	SYNE3	ENSG00000176438:95904139-95905499:target		20 0 20
2892	C8orf59	ENSG00000176731:86126311-86126860:source		24 0 24
2893	FAM91A1	ENSG00000176853:124796710-124797035:source		24 0 24
2894	FAM91A1	ENSG00000176853:124799904-124799990:source		35 26 0 59
2895	FAM91A1	ENSG00000176853:124801853-124801985:target		43 0 43
2896	FAM91A1	ENSG00000176853:124810330-124810478:target		5 51 0 59
2897	SEC24C	ENSG00000176986:75506563-75506762:target		56 0 56
2898	SEC24C	ENSG00000176986:75525209-75525347:source		31 0 32
2899	SEC24C	ENSG00000176986:75525844-75525968:target		31 0 31
#	Gene	LSV ID	LSV Type	← More in Healthy | More in ALL →

#	Gene	LSV ID	LSV Type	← More in Healthy | More in ALL →
2900	CHD9	ENSG00000177200:53342598-53342766:target	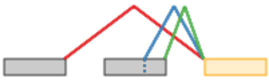	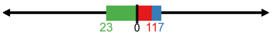 23 0 117
2901	CHD9	ENSG00000177200:53352124-53352252:source	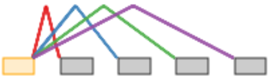	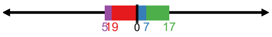 519 0 7 17
2902	ZFAS1	ENSG00000177410:47897022-47897107:source	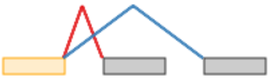	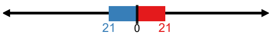 21 0 21
2903	ZFAS1	ENSG00000177410:47897440-47897501:source	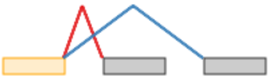	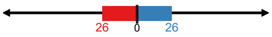 26 0 26
2904	ZFAS1	ENSG00000177410:47905582-47905797:target	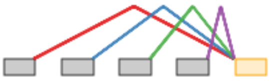	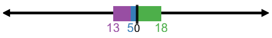 13 50 18
2905	CD19	ENSG00000177455:28944524-28944962:source	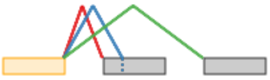	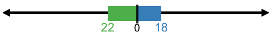 22 0 18
2906	CD19	ENSG00000177455:28947833-28947921:target	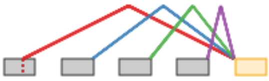	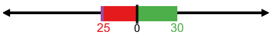 25 0 30
2907	ARIH2	ENSG00000177479:48965212-48965389:source	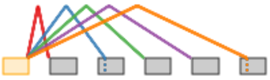	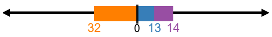 32 0 13 14
2908	ARIH2	ENSG00000177479:49002351-49002417:target	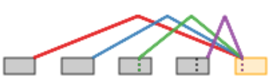	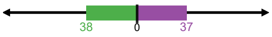 38 0 37
2909	RABEP2	ENSG00000177548:28944524-28944830:source	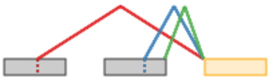	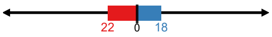 22 0 18
2910	RABEP2	ENSG00000177548:28947585-28947921:target	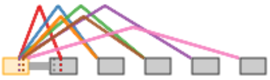	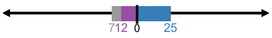 712 0 25
2911	AP3S1	ENSG00000177879:115230784-115230859:source	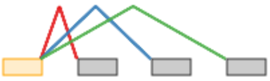	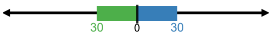 30 0 30
2912	AP3S1	ENSG00000177879:115249059-115249778:target	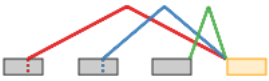	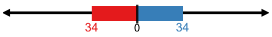 34 0 34
2913	GRB2	ENSG00000177885:73317740-73317834:target	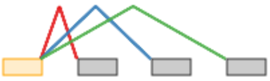	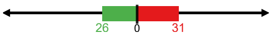 26 0 31
2914	UBE2N	ENSG00000177889:93804829-93805075:target	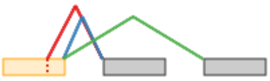	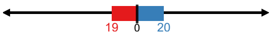 19 0 20
2915	RPS27	ENSG00000177954:153963591-153963699:target	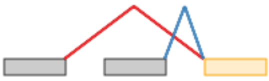	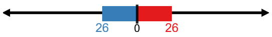 26 0 26
2916	NDUFV2	ENSG00000178127:9102628-9102795:source	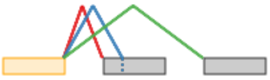	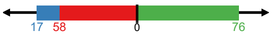 17 58 0 76
2917	ZNF114	ENSG00000178150:48744219-48744320:source	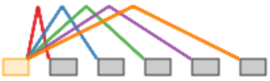	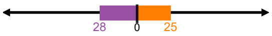 28 0 25
2918	ZNF114	ENSG00000178150:48753008-48753104:target	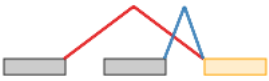	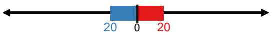 20 0 20
2919	PLEC	ENSG00000178209:145000952-145001050:target	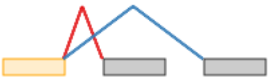	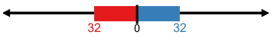 32 0 32

| # | Gene | LSV ID | LSV Type | ← More in Healthy \| More in ALL → |

#	Gene	LSV ID	LSV Type	← More in Healthy | More in ALL →
2920	PLEC	ENSG00000178209:145002022-145002691:source	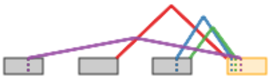	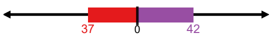
2921	PLEC	ENSG00000178209:145002844-145003449:target	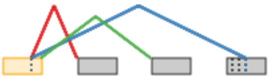	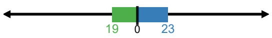
2922	ZFAND2A	ENSG00000178381:1195089-1195767:target	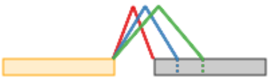	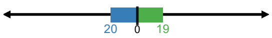
2923	SUZ12	ENSG00000178691:30300165-30300250:source	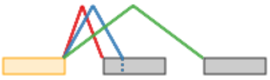	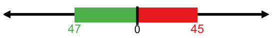
2924	SUZ12	ENSG00000178691:30303540-30303633:source	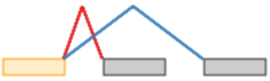	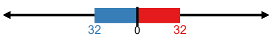
2925	SUZ12	ENSG00000178691:30303540-30303633:target	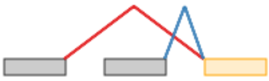	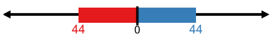
2926	APOLD1	ENSG00000178878:12967065-12967158:source	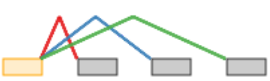	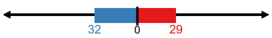
2927	APOLD1	ENSG00000178878:12974589-12974660:target	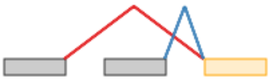	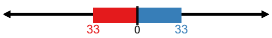
2928	APOLD1	ENSG00000178878:12976137-12976253:source	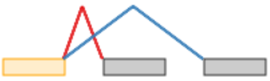	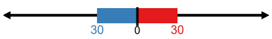
2929	GAK	ENSG00000178950:898335-898569:target	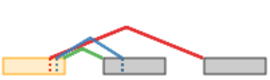	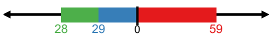
2930	RCC2	ENSG00000179051:17735172-17735690:source	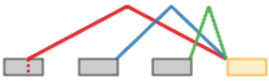	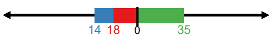
2931	PER1	ENSG00000179094:8052780-8052981:target	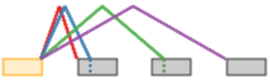	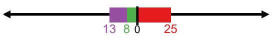
2932	RAD23A	ENSG00000179262:13058991-13059172:source	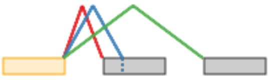	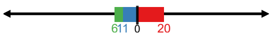
2933	CLK3	ENSG00000179335:74919880-74920009:source	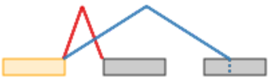	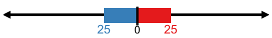
2934	HLA-DQB1	ENSG00000179344:32629606-32630130:target	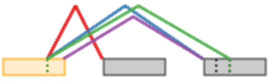	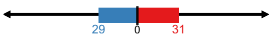
2935	ZBTB18	ENSG00000179456:244217090-244220778:target	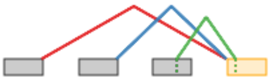	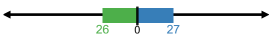
2936	PDXDC1	ENSG00000179889:15116561-15116611:source	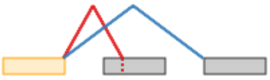	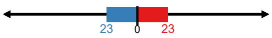
2937	PDXDC1	ENSG00000179889:15185172-15185228:target	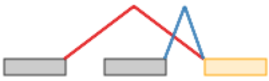	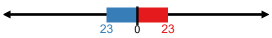
2938	SOCS4	ENSG00000180008:55509670-55516206:target	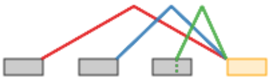	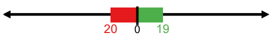
2939	PRKRA	ENSG00000180228:179309149-179309227:source	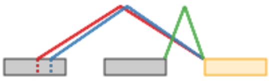	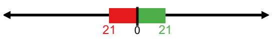
#	Gene	LSV ID	LSV Type	← More in Healthy | More in ALL →

#	Gene	LSV ID	LSV Type	← More in Healthy | More in ALL →
2940	ZNRF2	ENSG00000180233:30405556-30407308:target	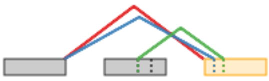	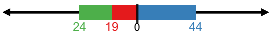
2941	HCLS1	ENSG00000180353:121355281-121355336:source	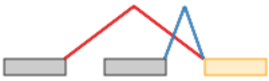	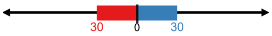
2942	HCLS1	ENSG00000180353:121361774-121361828:target	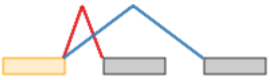	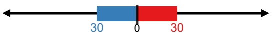
2943	MCFD2	ENSG00000180398:47135846-47136316:target	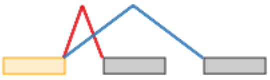	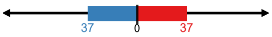
2944	ZDHHC20	ENSG00000180776:21955573-21955688:target	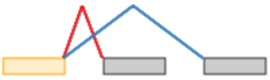	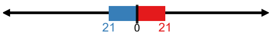
2945	ZDHHC20	ENSG00000180776:21957059-21957148:target	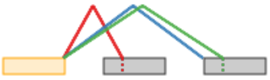	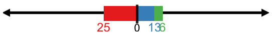
2946	PITPNB	ENSG00000180957:28247657-28249651:source	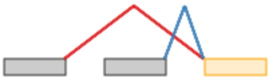	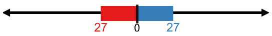
2947	PITPNB	ENSG00000180957:28254375-28254497:target	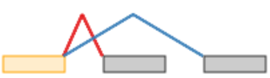	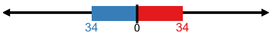
2948	EHMT1	ENSG00000181090:140657127-140657272:source	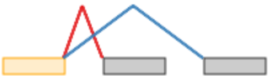	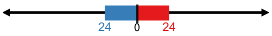
2949	EHMT1	ENSG00000181090:140671070-140671296:target	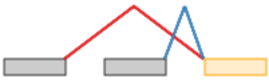	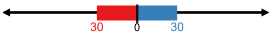
2950	EHMT1	ENSG00000181090:140676560-140676849:source	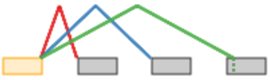	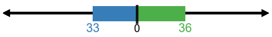
2951	SETD2	ENSG00000181555:47079155-47079267:source	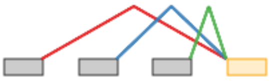	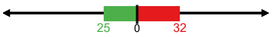
2952	RFX7	ENSG00000181827:56436599-56436681:target	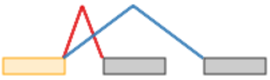	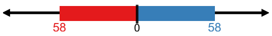
2953	RNF41	ENSG00000181852:56603821-56604352:source	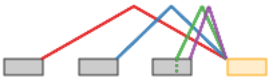	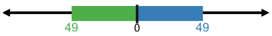
2954	RNF41	ENSG00000181852:56607742-56607854:target	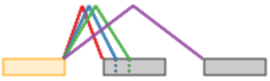	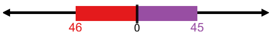
2955	TNRC18	ENSG00000182095:5399029-5399200:source	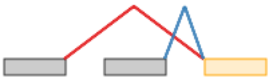	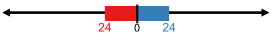
2956	TNRC18	ENSG00000182095:5401528-5401664:target	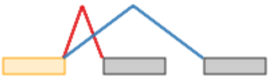	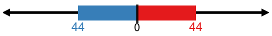
2957	IST1	ENSG00000182149:71957191-71957412:source	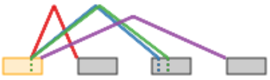	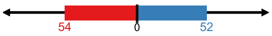
2958	IST1	ENSG00000182149:71958311-71958720:target	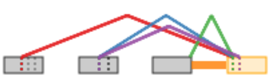	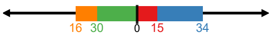
2959	SHMT2	ENSG00000182199:57624484-57624968:source	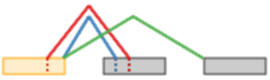	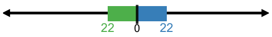

| # | Gene | LSV ID | LSV Type | ← More in Healthy \| More in ALL → |

#	Gene	LSV ID	LSV Type	← More in Healthy | More in ALL →
2960	NPLOC4	ENSG00000182446:79539043-79539134:source	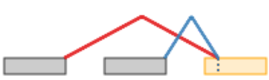	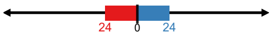
2961	NPLOC4	ENSG00000182446:79563142-79563268:target	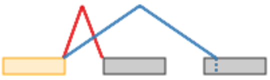	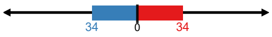
2962	SATB1	ENSG00000182568:18419662-18419819:target	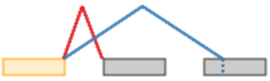	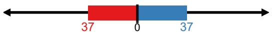
2963	TRAK1	ENSG00000182606:42055294-42055609:source	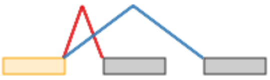	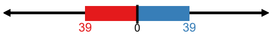
2964	TRAK1	ENSG00000182606:42128596-42128892:target	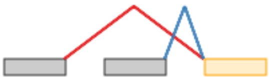	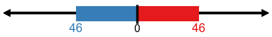
2965	RRP7BP	ENSG00000182841:42951229-42952680:source	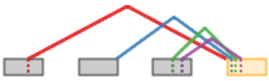	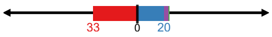
2966	RRP7BP	ENSG00000182841:42969250-42970824:target	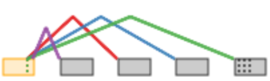	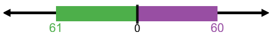
2967	SRPR	ENSG00000182934:126137444-126137607:source	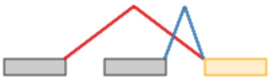	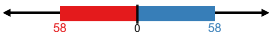
2968	SRPR	ENSG00000182934:126138583-126138877:target	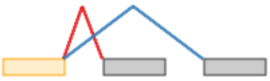	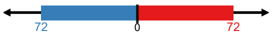
2969	EWSR1	ENSG00000182944:29682912-29683123:source	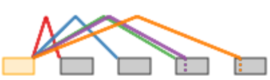	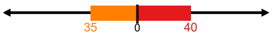
2970	EWSR1	ENSG00000182944:29687918-29688158:target	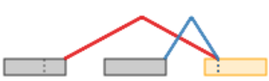	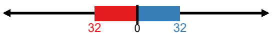
2971	EWSR1	ENSG00000182944:29695589-29695974:target	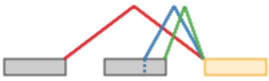	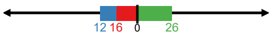
2972	CAMK1D	ENSG00000183049:12867572-12868142:source	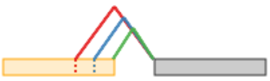	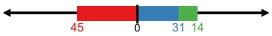
2973	SEP15	ENSG00000183291:87346345-87346408:source	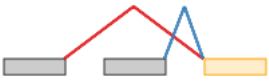	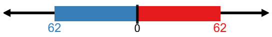
2974	SEP15	ENSG00000183291:87368964-87369131:source	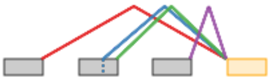	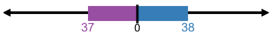
2975	SEP15	ENSG00000183291:87368964-87369131:target	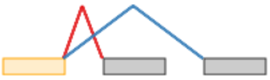	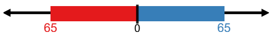
2976	SF3A3	ENSG00000183431:38450379-38450451:source	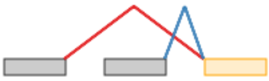	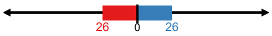
2977	HMCES	ENSG00000183624:128997799-128998054:source	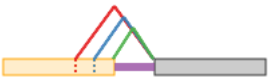	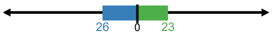
2978	HMCES	ENSG00000183624:128998553-128998758:target	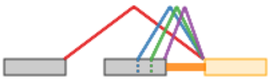	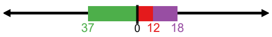
2979	AC138969.4	ENSG00000183889:16434163-16434358:target	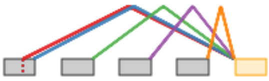	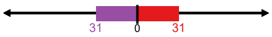

| # | Gene | LSV ID | LSV Type | ← More in Healthy \| More in ALL → |

#	Gene	LSV ID	LSV Type	← More in Healthy | More in ALL →
2980	DENND5A	ENSG00000184014:9167217-9167362:source		22 0 22
2981	DENND5A	ENSG00000184014:9171628-9171756:target		22 0 22
2982	DENND5A	ENSG00000184014:9199477-9199913:target		72 0 66
2983	DIABLO	ENSG00000184047:122700636-122701131:target		43 0 43
2984	CRELD2	ENSG00000184164:50313378-50313488:source		26 0 26
2985	CRELD2	ENSG00000184164:50313639-50313956:source		35 0 256
2986	CRELD2	ENSG00000184164:50315232-50315480:source		20 0 95
2987	CRELD2	ENSG00000184164:50316218-50316387:target		30 0 22
2988	CRELD2	ENSG00000184164:50319065-50320153:source		60 0 4310
2989	CRELD2	ENSG00000184164:50320903-50321188:target		63 0 10 3910
2990	UBE2F	ENSG00000184182:238903386-238903451:source		32 0 35
2991	UBE2F	ENSG00000184182:238933902-238934053:target		28 0 35
2992	CMSS1	ENSG00000184220:99536678-99536887:source		613 42 0 65
2993	PCDH9	ENSG00000184226:67798863-67802707:source		21 0 21
2994	RBM33	ENSG00000184863:155473284-155473602:source		22 0 22
2995	RBM33	ENSG00000184863:155537655-155538414:source		24 0 25
2996	RBM33	ENSG00000184863:155556503-155556712:target		24 0 24
2997	RBM33	ENSG00000184863:155559161-155559590:target		22 0 22
2998	FMNL1	ENSG00000184922:43322620-43322783:source		33 0 33
2999	FMNL1	ENSG00000184922:43323245-43323340:target		40 0 40

| # | Gene | LSV ID | LSV Type | ← More in Healthy \| More in ALL → |

#	Gene	LSV ID	LSV Type	← More in Healthy | More in ALL →
3000	NDUFA6	ENSG00000184983:42486610-42486959:target	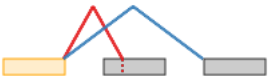	20 0 20
3001	HSF1	ENSG00000185122:145536727-145537302:target	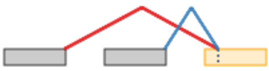	25 0 25
3002	PRPF39	ENSG00000185246:45566090-45566208:target	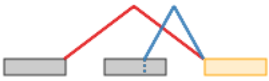	26 0 26
3003	PRPF39	ENSG00000185246:45579297-45579423:target	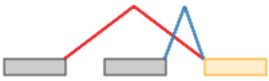	23 0 23
3004	SP140L	ENSG00000185404:231253274-231253501:source	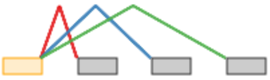	55 0 53
3005	SP140L	ENSG00000185404:231258129-231258176:target	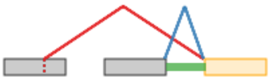	23 12 0 38
3006	PCGF3	ENSG00000185619:727461-727578:source	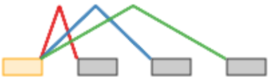	36 0 36
3007	P4HB	ENSG00000185624:79817166-79817606:source	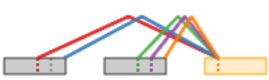	19 0 22
3008	PSMD13	ENSG00000185627:244041-244075:target	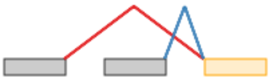	26 0 26
3009	PSMD13	ENSG00000185627:252505-252983:target	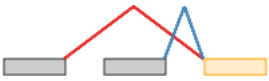	41 0 41
3010	UBE2L3	ENSG00000185651:21965146-21965332:source	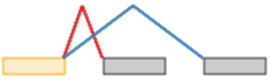	50 0 50
3011	UBE2L3	ENSG00000185651:21975804-21978323:target	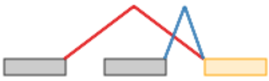	60 0 60
3012	BRWD1	ENSG00000185658:40684489-40684826:target	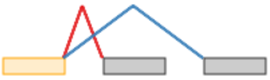	22 0 22
3013	ANKFY1	ENSG00000185722:4119812-4120413:target	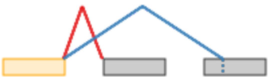	20 0 20
3014	YTHDF3	ENSG00000185728:64081946-64082050:source	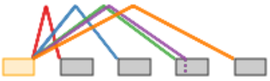	23 0 29
3015	YTHDF3	ENSG00000185728:64087890-64088259:source	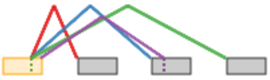	25 0 24
3016	YTHDF3	ENSG00000185728:64098706-64099121:target	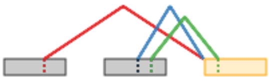	22 0 22
3017	MORF4L1	ENSG00000185787:79185881-79185969:source	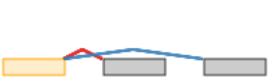	25 0 25
3018	MORF4L1	ENSG00000185787:79187162-79187250:target	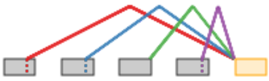	24 0 26
3019	IKZF1	ENSG00000185811:50435363-50437142:source	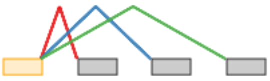	36 0 32

Gene LSV ID LSV Type ← More in Healthy | More in ALL →

#	Gene	LSV ID	LSV Type	← More in Healthy | More in ALL →
3020	IKZF1	ENSG00000185811:50450238-50450405:target		20 0 5 9
3021	IKZF1	ENSG00000185811:50467616-50472799:target		9 14 0 18
3022	LRCH3	ENSG00000186001:197553749-197554023:source		16 11 0 30
3023	LRCH3	ENSG00000186001:197598196-197598456:source		23 0 23
3024	GSAP	ENSG00000186088:76940068-76940766:source		33 0 33
3025	GSAP	ENSG00000186088:76941418-76941519:target		40 0 40
3026	GSAP	ENSG00000186088:76958844-76960035:target		42 0 43
3027	GSAP	ENSG00000186088:76984530-76984747:target		25 0 22
3028	GSAP	ENSG00000186088:77006603-77006707:source		50 0 50
3029	GSAP	ENSG00000186088:77010622-77010671:target		41 0 41
3030	GSAP	ENSG00000186088:77033876-77033932:target		30 0 30
3031	POLR1D	ENSG00000186184:28222147-28222590:source		29 0 29
3032	ENSG00000186275	ENSG00000186275:146404477-146404803:source		28 0 28
3033	ENSG00000186275	ENSG00000186275:146404477-146404803:target		24 0 24
3034	INSIG1	ENSG00000186480:155090271-155090407:source		38 0 35
3035	INSIG1	ENSG00000186480:155093961-155094127:source		14 23 0 37
3036	INSIG1	ENSG00000186480:155094457-155094728:target		29 0 31
3037	INSIG1	ENSG00000186480:155099951-155101945:target		5735 0 34 13
3038	ARHGAP30	ENSG00000186517:161039318-161039760:target		30 0 35
3039	GPATCH8	ENSG00000186566:42513846-42513913:source		27 0 24

| # | Gene | LSV ID | LSV Type | ← More in Healthy \| More in ALL → |

#	Gene	LSV ID	LSV Type	← More in Healthy | More in ALL →
3040	GPATCH8	ENSG00000186566:42551788-42552271:target		41 0 40
3041	UBE2H	ENSG00000186591:129479044-129479581:source		26 0 26
3042	ARAP1	ENSG00000186635:72421430-72421632:source		21 0 21
3043	ARAP1	ENSG00000186635:72422475-72422544:target		25 0 25
3044	TCL6	ENSG00000187621:96131199-96131413:source		6 14 0 24
3045	TCL6	ENSG00000187621:96135821-96136751:target		26 0 15 12
3046	TCEA1	ENSG00000187735:54891585-54891731:source		32 0 32
3047	TCEA1	ENSG00000187735:54899520-54899585:target		31 0 31
3048	FANCA	ENSG00000187741:89803957-89805116:source		22 0 29
3049	SECISBP2	ENSG00000187742:91947503-91947901:source		59 0 23 36
3050	SECISBP2	ENSG00000187742:91949194-91949645:target		62 0 25 35
3051	SECISBP2	ENSG00000187742:91956262-91956394:target		25 0 25
3052	SECISBP2	ENSG00000187742:91965547-91965767:source		36 0 38
3053	SECISBP2	ENSG00000187742:91972914-91973106:target		35 0 35
3054	TMEM220	ENSG00000187824:10609755-10609871:target		32 0 32
3055	HNRNPU-AS1	ENSG00000188206:245003940-245008646:source		56 0 40 16
3056	SRSF10	ENSG00000188529:24291294-24294446:source		46 0 39
3057	SRSF10	ENSG00000188529:24298063-24298116:target		28 0 25
3058	PTAR1	ENSG00000188647:72349066-72349174:source		23 0 22
3059	PTAR1	ENSG00000188647:72356432-72356774:target		25 0 25

| # | Gene | LSV ID | LSV Type | ← More in Healthy \| More in ALL → |

#	Gene	LSV ID	LSV Type	← More in Healthy | More in ALL →
3060	PTAR1	ENSG00000188647:72374769-72374875:target	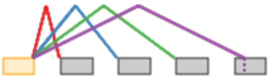	33 0 34
3061	RBM34	ENSG00000188739:235323826-235323962:target	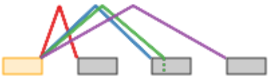	36 0 34
3062	ZNF548	ENSG00000188785:57905411-57905591:target	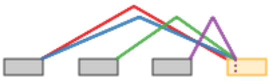	27 0 26
3063	RPL14	ENSG00000188846:40498783-40499238:source	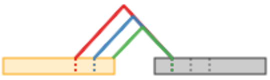	9 67 0 76
3064	ZNF292	ENSG00000188994:87928314-87928449:source	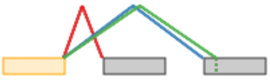	32 0 32
3065	ARID2	ENSG00000189079:46233112-46236017:source	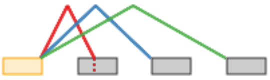	12 12 0 28
3066	ARID2	ENSG00000189079:46240639-46240720:target	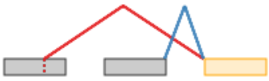	30 0 30
3067	SF3B3	ENSG00000189091:70572226-70572363:source	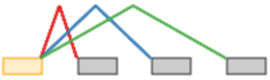	26 12 0 40
3068	SF3B3	ENSG00000189091:70578341-70578436:target	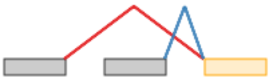	29 0 29
3069	SF3B3	ENSG00000189091:70582273-70582415:source	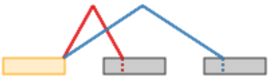	24 0 24
3070	SF3B3	ENSG00000189091:70588009-70588500:target	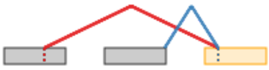	24 0 24
3071	SF3B3	ENSG00000189091:70590789-70590932:source	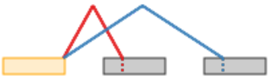	25 0 25
3072	ZNF600	ENSG00000189190:53267448-53271025:source	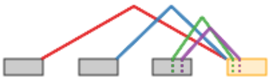	26 0 13 11
3073	SLC35E2B	ENSG00000189339:1601537-1601590:source	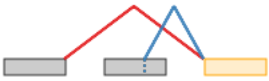	22 0 22
3074	SLC35E2B	ENSG00000189339:1606902-1607029:target	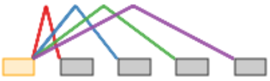	20 0 19
3075	HMGB1	ENSG00000189403:31032884-31035670:source	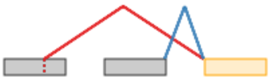	43 0 43
3076	HMGB1	ENSG00000189403:31036483-31036849:target	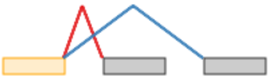	26 0 26
3077	PAX5	ENSG00000196092:36846840-36846926:source	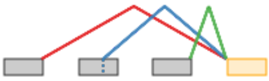	36 16 0 51
3078	PAX5	ENSG00000196092:36922691-36923481:target	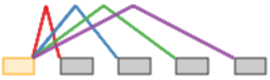	30 10 0 43
3079	PAX5	ENSG00000196092:36966546-36966721:target	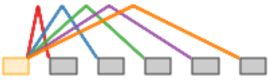	11 0 20

Gene LSV ID LSV Type ← More in Healthy | More in ALL →

#	Gene	LSV ID	LSV Type	← More in Healthy | More in ALL →
3080	HLA-DRB1	ENSG00000196126:32551886-32552155:target		39 0 39
3081	STK40	ENSG00000196182:36823840-36823983:source		14 10 0 27
3082	TMEM63A	ENSG00000196187:226034735-226035166:source		23 0 23
3083	TMEM63A	ENSG00000196187:226036193-226036255:target		22 0 23
3084	PPIA	ENSG00000196262:44837572-44838503:target		20 0 20
3085	GTF2IRD2	ENSG00000196275:74267663-74267847:target		20 0 20
3086	AC005154.6	ENSG00000196295:30601082-30601319:target		23 0 23
3087	IARS	ENSG00000196305:94991283-94991408:source		37 0 37
3088	IARS	ENSG00000196305:95003045-95003243:target		47 0 47
3089	IARS	ENSG00000196305:95018639-95019082:source		21 0 21
3090	POM121	ENSG00000196313:72361166-72361292:target		24 0 24
3091	POM121	ENSG00000196313:72361472-72361649:source		15 0 20
3092	POM121	ENSG00000196313:72397340-72397501:source		25 0 25
3093	CD55	ENSG00000196352:207510038-207510595:source		24 0 24
3094	TRRAP	ENSG00000196367:98554022-98554156:source		21 0 21
3095	TRRAP	ENSG00000196367:98580887-98581115:source		20 0 20
3096	XRCC6	ENSG00000196419:42052907-42053036:source		21 0 28
3097	XRCC6	ENSG00000196419:42057335-42057703:target		22 7 0 32
3098	TSC22D2	ENSG00000196428:150126122-150129095:source		20 0 20
3099	TSC22D2	ENSG00000196428:150174738-150174914:target		28 0 18

| # | Gene | LSV ID | LSV Type | ← More in Healthy \| More in ALL → |

#	Gene	LSV ID	LSV Type	← More in Healthy | More in ALL →
3100	ZNF775	ENSG00000196456:150107248-150109558:target	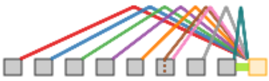	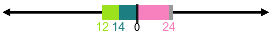
3101	NCOR2	ENSG00000196498:124821185-124821726:target	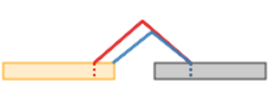	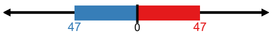
3102	NCOR2	ENSG00000196498:124826369-124826620:target	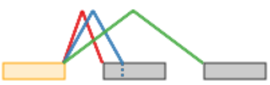	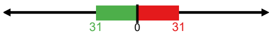
3103	PRPF40A	ENSG00000196504:153520652-153520766:target	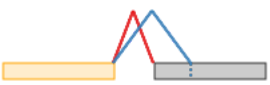	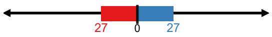
3104	PRPF40A	ENSG00000196504:153549552-153549625:source	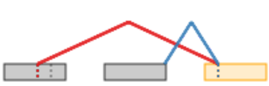	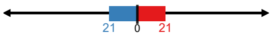
3105	PRPF40A	ENSG00000196504:153550577-153551136:target	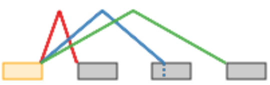	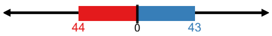
3106	MAN2A2	ENSG00000196547:91455413-91455509:source	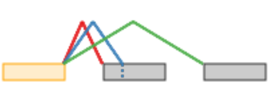	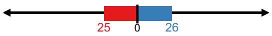
3107	PDXDC2P	ENSG00000196696:70055384-70055852:target	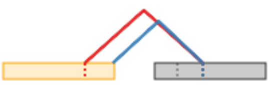	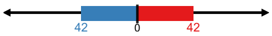
3108	AMZ2	ENSG00000196704:66246328-66246621:target	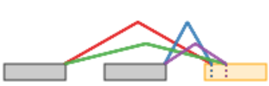	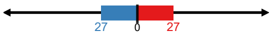
3109	HLA-DQA1	ENSG00000196735:32609730-32610030:source	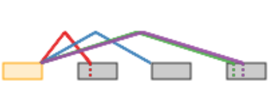	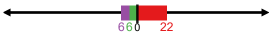
3110	SNHG17	ENSG00000196756:37055062-37055146:target	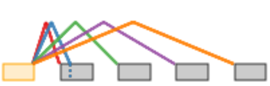	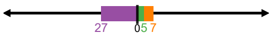
3111	CD47	ENSG00000196776:107762145-107766139:source	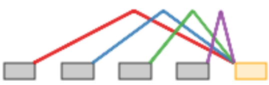	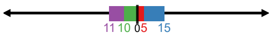
3112	CD47	ENSG00000196776:107770786-107773586:source	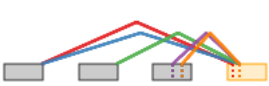	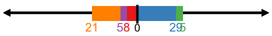
3113	CD47	ENSG00000196776:107770786-107773586:target	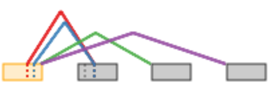	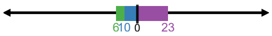
3114	TLE1	ENSG00000196781:84228292-84228437:source	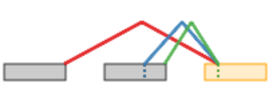	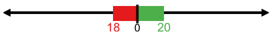
3115	TLE1	ENSG00000196781:84231561-84231614:target	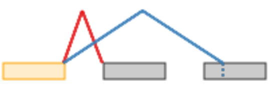	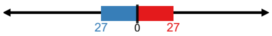
3116	C6orf106	ENSG00000196821:34622402-34622556:target	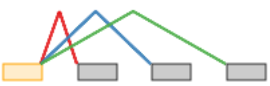	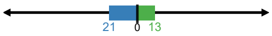
3117	ARID5A	ENSG00000196843:97215490-97215542:target	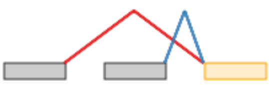	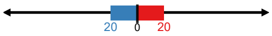
3118	ARID5A	ENSG00000196843:97215766-97216022:source	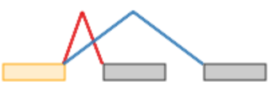	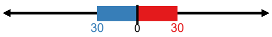
3119	PPTC7	ENSG00000196850:110975949-110976078:source	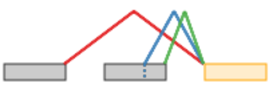	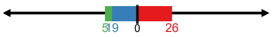

| # | Gene | LSV ID | LSV Type | ← More in Healthy \| More in ALL → |

#	Gene	LSV ID	LSV Type	← More in Healthy | More in ALL →
3120	PPTC7	ENSG00000196850:110977627-110977750:target	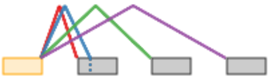	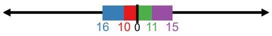
3121	FAM3C	ENSG00000196937:121000097-121000181:source	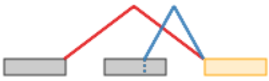	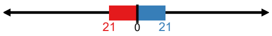
3122	ANXA6	ENSG00000197043:150497319-150497398:source	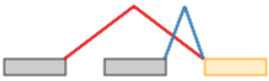	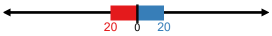
3123	ANXA6	ENSG00000197043:150501708-150501821:target	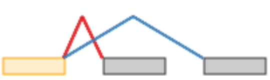	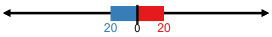
3124	IGF2R	ENSG00000197081:160490900-160491090:target	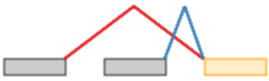	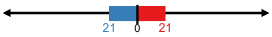
3125	DYNC1H1	ENSG00000197102:102500313-102500525:source	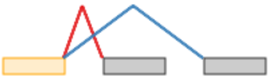	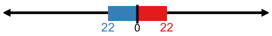
3126	DYNC1H1	ENSG00000197102:102502826-102502979:target	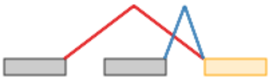	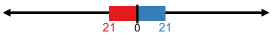
3127	DYNC1H1	ENSG00000197102:102506935-102507465:source	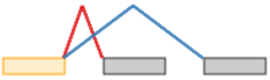	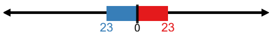
3128	PCBP2	ENSG00000197111:53848085-53848653:target	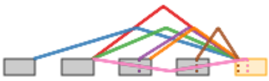	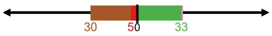
3129	ENTPD4	ENSG00000197217:23299082-23299236:source	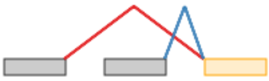	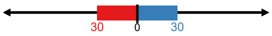
3130	ENTPD4	ENSG00000197217:23301363-23301466:target	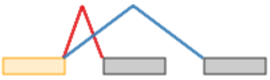	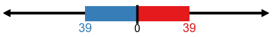
3131	DDI2	ENSG00000197312:15978201-15978390:source	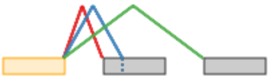	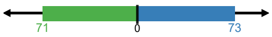
3132	DDI2	ENSG00000197312:15986332-15995539:target	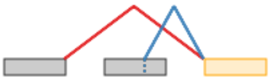	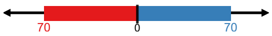
3133	TRIM33	ENSG00000197323:114940230-114940482:source	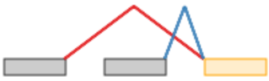	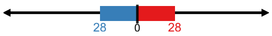
3134	TRIM33	ENSG00000197323:114942079-114942231:source	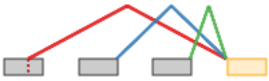	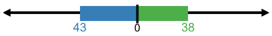
3135	TRIM33	ENSG00000197323:114942079-114942231:target	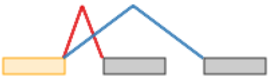	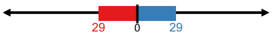
3136	TRIM33	ENSG00000197323:114945382-114945505:target	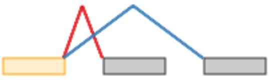	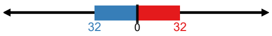
3137	ZNF655	ENSG00000197343:99159967-99160117:source	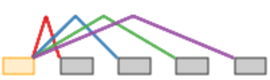	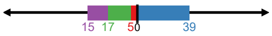
3138	ZNF655	ENSG00000197343:99169311-99169415:target	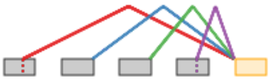	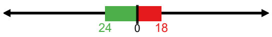
3139	MYO5A	ENSG00000197535:52646068-52646211:source	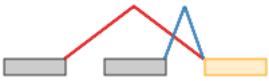	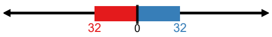

| # | Gene | LSV ID | LSV Type | ← More in Healthy \| More in ALL → |

#	Gene	LSV ID	LSV Type	← More in Healthy | More in ALL →
3140	MYO5A	ENSG00000197535:52656751-52656899:target		22 0 22
3141	C5orf56	ENSG00000197536:131785239-131785683:target		27 0 27
3142	SIPA1L1	ENSG00000197555:71979460-71979562:source		22 0 22
3143	SIPA1L1	ENSG00000197555:71996029-71996087:target		25 0 25
3144	SIPA1L1	ENSG00000197555:72085473-72085604:source		23 0 23
3145	SIPA1L1	ENSG00000197555:72090765-72090953:target		28 0 28
3146	SIPA1L1	ENSG00000197555:72169029-72169222:source		22 0 22
3147	SIPA1L1	ENSG00000197555:72171939-72172057:target		23 0 23
3148	CDC42SE1	ENSG00000197622:151028059-151028469:target		35 0 34
3149	ENSG00000197681	ENSG00000197681:36351936-36351996:source		26 0 26
3150	SPTAN1	ENSG00000197694:131353756-131353904:source		26 0 26
3151	SPTAN1	ENSG00000197694:131371401-131371568:source		26 0 26
3152	SPTAN1	ENSG00000197694:131373993-131374124:target		22 0 23
3153	SLC9A8	ENSG00000197818:48504366-48508779:target		29 0 29
3154	FAM49A	ENSG00000197872:16746914-16747035:target		15 0 22
3155	FAM49A	ENSG00000197872:16805132-16805287:target		14 0 24
3156	MYO1C	ENSG00000197879:1372084-1372908:target		26 0 26
3157	NKIRAS1	ENSG00000197885:23959341-23959522:target		49 6 0 60
3158	SPG7	ENSG00000197912:89592737-89592876:source		24 0 24
3159	SPG7	ENSG00000197912:89597091-89597216:target		27 0 24

| # | Gene | LSV ID | LSV Type | ← More in Healthy \| More in ALL → |

#	Gene	LSV ID	LSV Type	← More in Healthy | More in ALL →
3160	SPG7	ENSG00000197912:89616254-89617017:target	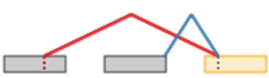	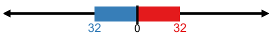
3161	PLCG2	ENSG00000197943:81888049-81888192:target	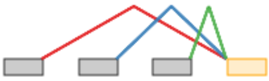	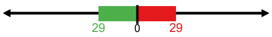
3162	PLCG2	ENSG00000197943:81964866-81965259:source	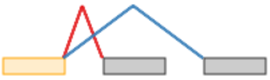	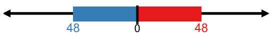
3163	PLCG2	ENSG00000197943:81969774-81969983:target	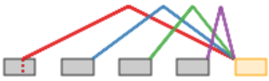	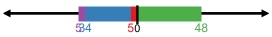
3164	ZNF121	ENSG00000197961:9679556-9679758:target	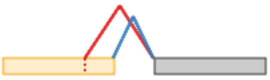	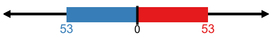
3165	SNHG12	ENSG00000197989:28906424-28906493:source	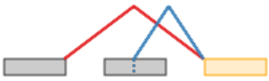	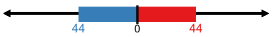
3166	SNHG12	ENSG00000197989:28907072-28907158:source	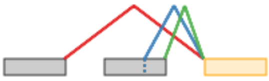	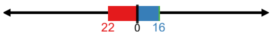
3167	SNHG12	ENSG00000197989:28908102-28908384:target	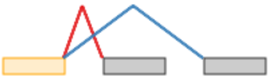	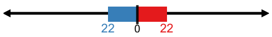
3168	NOL8	ENSG00000198000:95072835-95073107:source	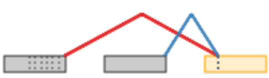	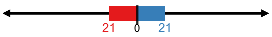
3169	NOL8	ENSG00000198000:95076549-95078421:target	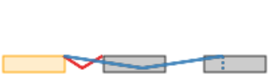	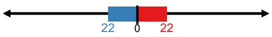
3170	NOL8	ENSG00000198000:95081503-95081638:source	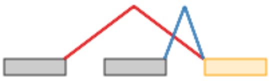	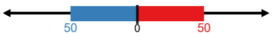
3171	IRAK4	ENSG00000198001:44165983-44166165:target	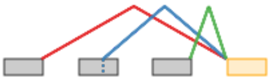	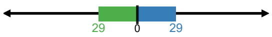
3172	GRK6	ENSG00000198055:176857677-176857968:source	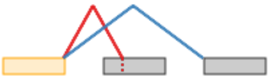	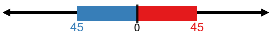
3173	GRK6	ENSG00000198055:176859234-176859311:target	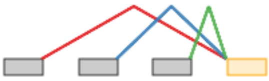	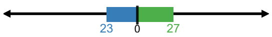
3174	SNX29P2	ENSG00000198106:29465337-29465434:target	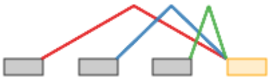	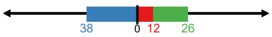
3175	SZT2	ENSG00000198198:43916008-43918321:target	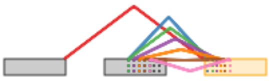	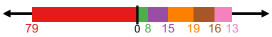
3176	QRICH1	ENSG00000198218:49114140-49114471:source	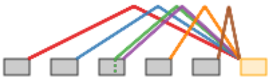	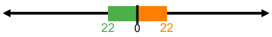
3177	DDX42	ENSG00000198231:61875398-61875556:source	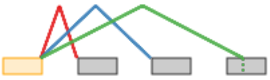	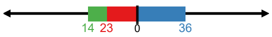
3178	DDX42	ENSG00000198231:61877511-61877977:target	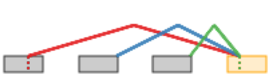	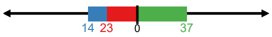
3179	HELZ	ENSG00000198265:65103285-65103801:source	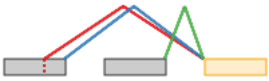	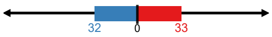
#	Gene	LSV ID	LSV Type	← More in Healthy | More in ALL →

#	Gene	LSV ID	LSV Type	← More in Healthy | More in ALL →
3180	HELZ	ENSG00000198265:65124611-65124971:source		49 0 49
3181	HELZ	ENSG00000198265:65134047-65134230:target		43 0 43
3182	HELZ	ENSG00000198265:65163579-65163912:source		43 0 43
3183	HELZ	ENSG00000198265:65174775-65175042:target		34 13 0 46
3184	HELZ	ENSG00000198265:65185705-65185812:source		15 0 20
3185	HELZ	ENSG00000198265:65186273-65186471:source		13 0 21
3186	HELZ	ENSG00000198265:65190083-65190158:target		28 0 28
3187	CARD11	ENSG00000198286:2959006-2959246:target		26 0 26
3188	UVRAG	ENSG00000198382:75826944-75827059:source		17 0 21
3189	ITSN2	ENSG00000198399:24483975-24484119:target		53 0 53
3190	MGEA5	ENSG00000198408:103556803-103557911:target		33 0 32
3191	HLA-DRB5	ENSG00000198502:32485120-32485529:source		26 0 238
3192	DDX39B	ENSG00000198563:31502588-31503262:target		24 27 0 52
3193	TLK1	ENSG00000198586:171871361-171871458:source		38 0 38
3194	TLK1	ENSG00000198586:171884849-171884915:target		27 0 35
3195	TLK1	ENSG00000198586:171974249-171974367:target		24 0 24
3196	LRBA	ENSG00000198589:151185594-151186964:source		30 0 30
3197	LRBA	ENSG00000198589:151203602-151203798:target		27 0 27
3198	BAZ1A	ENSG00000198604:35254983-35255297:source		47 0 47
3199	BAZ1A	ENSG00000198604:35261981-35262127:target		54 0 54

Gene LSV ID LSV Type ← More in Healthy | More in ALL →

#	Gene	LSV ID	LSV Type	← More in Healthy | More in ALL →
3200	MDM4	ENSG00000198625:204494612-204494724:target	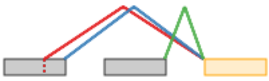	25 0 27
3201	TTC37	ENSG00000198677:94852615-94852764:target	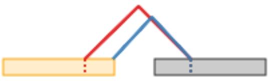	20 0 20
3202	FAN1	ENSG00000198690:31233769-31235311:target	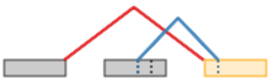	33 0 33
3203	LDB1	ENSG00000198728:103871191-103871293:source	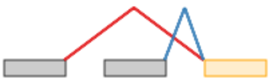	41 0 41
3204	RCSD1	ENSG00000198771:167663336-167663539:target	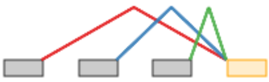	19 0 20
3205	MTOR	ENSG00000198793:11175453-11175720:source	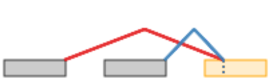	52 0 52
3206	MTOR	ENSG00000198793:11181303-11181425:target	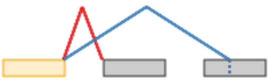	57 0 57
3207	OPA1	ENSG00000198836:193336616-193336726:target	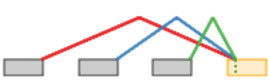	21 0 18
3208	OPA1	ENSG00000198836:193349401-193349454:source	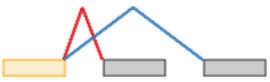	21 0 21
3209	DENND4B	ENSG00000198837:153911376-153911521:source	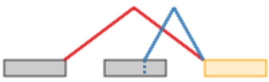	20 0 20
3210	SELT	ENSG00000198843:150340172-150340282:target	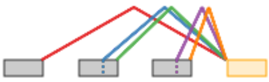	12 13 0 26
3211	R3HDM4	ENSG00000198858:900061-900146:target	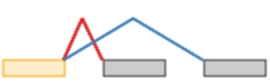	21 0 21
3212	R3HDM4	ENSG00000198858:901976-902130:source	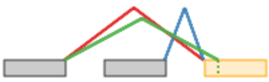	57 0 57
3213	R3HDM4	ENSG00000198858:913087-913240:target	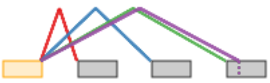	46 0 48
3214	GRK5	ENSG00000198873:121190899-121191039:source	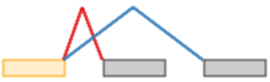	29 0 29
3215	GRK5	ENSG00000198873:121199243-121199280:source	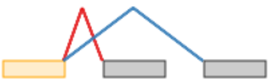	37 0 37
3216	GRK5	ENSG00000198873:121199243-121199280:target	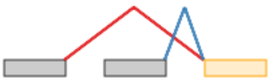	30 0 30
3217	GRK5	ENSG00000198873:121203056-121203264:target	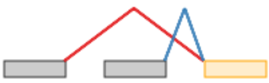	49 0 49
3218	SMC5	ENSG00000198887:72882839-72882891:source	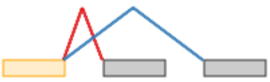	25 0 25
3219	SMC5	ENSG00000198887:72892226-72892388:target	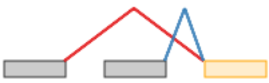	23 0 23

| # | Gene | LSV ID | LSV Type | ← More in Healthy \| More in ALL → |

#	Gene	LSV ID	LSV Type	← More in Healthy | More in ALL →
3220	SMC5	ENSG00000198887:72959060-72959590:source	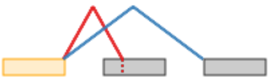	26 0 26
3221	TOP1	ENSG00000198900:39713102-39713208:source	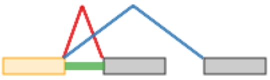	55 0 55
3222	TOP1	ENSG00000198900:39721112-39721227:target	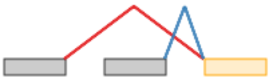	74 0 74
3223	MAP3K3	ENSG00000198909:61723394-61723434:target	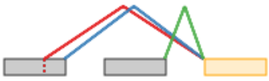	20 0 20
3224	MAP3K3	ENSG00000198909:61766102-61766241:target	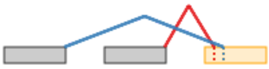	38 0 38
3225	MAP3K3	ENSG00000198909:61766536-61767832:source	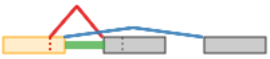	56 0 56
3226	MAP3K3	ENSG00000198909:61767624-61767772:target	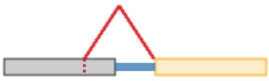	24 0 24
3227	SREBF2	ENSG00000198911:42271547-42271728:source	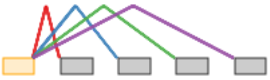	10 9 11 0 34
3228	SREBF2	ENSG00000198911:42273946-42274127:source	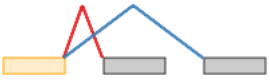	41 0 41
3229	SREBF2	ENSG00000198911:42276720-42276996:target	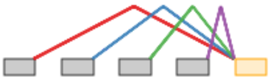	17 13 6 0 39
3230	SREBF2	ENSG00000198911:42290824-42290941:source	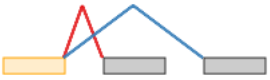	33 0 33
3231	SREBF2	ENSG00000198911:42296334-42296502:target	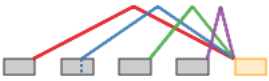	18 13 0 38
3232	ATG9A	ENSG00000198925:220085802-220086024:source	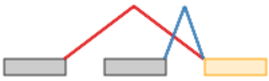	45 0 45
3233	ATG9A	ENSG00000198925:220086994-220087172:target	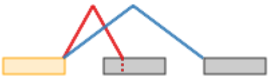	46 0 46
3234	ATG9A	ENSG00000198925:220092492-220092535:source	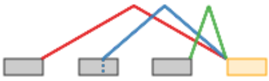	30 0 28
3235	SMG5	ENSG00000198952:156237261-156237469:target	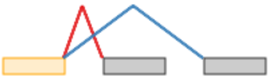	23 0 23
3236	MSTO2P	ENSG00000203761:155640111-155640255:source	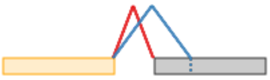	33 0 33
3237	ATP1A1-AS1	ENSG00000203865:116936153-116936345:source	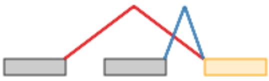	53 0 53
3238	ATP1A1-AS1	ENSG00000203865:116939220-116939356:target	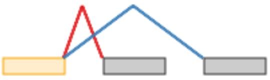	53 0 53
3239	GIGYF2	ENSG00000204120:233651860-233652039:target	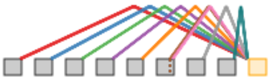	19 0 21

| # | Gene | LSV ID | LSV Type | ← More in Healthy \| More in ALL → |

#	Gene	LSV ID	LSV Type	← More in Healthy | More in ALL →
3240	PHACTR4	ENSG00000204138:28819527-28819603:source		56 0 56
3241	RXRB	ENSG00000204231:33162722-33163137:source		46 0 46
3242	RXRB	ENSG00000204231:33163347-33163479:target		40 0 22 18
3243	HLA-DMA	ENSG00000204257:32917861-32918580:source		22 0 32
3244	HLA-DMA	ENSG00000204257:32917861-32918580:target		8 14 0 17 5
3245	HLA-DMA	ENSG00000204257:32920726-32920899:target		23 0 23
3246	PSMB8	ENSG00000204264:32810719-32810866:source		51 0 51
3247	AGER	ENSG00000204305:32148745-32149016:source		21 0 21
3248	RNF5	ENSG00000204308:32148006-32149016:source		21 0 21
3249	C6orf48	ENSG00000204387:31804620-31805212:target		21 0 21
3250	CARD16	ENSG00000204397:104912053-104912446:source		21 0 21
3251	CARD16	ENSG00000204397:104914638-104915658:target		24 0 24
3252	BAG6	ENSG00000204463:31612723-31612975:target		8 18 0 30
3253	BAG6	ENSG00000204463:31619433-31619553:source		24 0 14
3254	PRRC2A	ENSG00000204469:31593237-31593388:source		52 0 52
3255	PRRC2A	ENSG00000204469:31593797-31593939:target		67 0 67
3256	PRRC2A	ENSG00000204469:31602530-31602754:target		25 0 21
3257	PRRC2A	ENSG00000204469:31603137-31603242:source		26 0 26
3258	PRRC2A	ENSG00000204469:31603744-31603826:target		24 0 24
3259	HLA-C	ENSG00000204525:31237270-31237564:source		21 0 20

| # | Gene | LSV ID | LSV Type | ← More in Healthy \| More in ALL → |

#	Gene	LSV ID	LSV Type	← More in Healthy | More in ALL →
3260	HLA-C	ENSG00000204525:31237743-31237862:source		38 0 37
3261	HLA-C	ENSG00000204525:31237987-31238204:source		5 15 0 20
3262	HLA-C	ENSG00000204525:31238850-31239349:source		24 0 8 15
3263	HLA-C	ENSG00000204525:31239376-31239645:source		23 6 0 30
3264	HLA-C	ENSG00000204525:31322705-31323369:target		26 0 15 18
3265	GNL1	ENSG00000204590:30514889-30515051:target		15 22 0 37
3266	HLA-E	ENSG00000204592:30458914-30459189:source		15 0 20
3267	E2F4	ENSG00000205250:67228589-67228821:target		23 0 23
3268	PDE7A	ENSG00000205268:66639405-66640392:source		26 0 27
3269	PDE7A	ENSG00000205268:66651737-66651832:target		28 0 37
3270	IPO7	ENSG00000205339:9438606-9438695:target		21 0 21
3271	NAP1L4	ENSG00000205531:2965667-2966876:source		21 0 21
3272	NAP1L4	ENSG00000205531:2972489-2972545:target		21 0 21
3273	NAP1L4	ENSG00000205531:2973010-2973039:source		13 17 0 31
3274	NAP1L4	ENSG00000205531:2975718-2975876:target		9 18 0 27
3275	NAP1L4	ENSG00000205531:2976974-2977020:source		49 0 49
3276	NAP1L4	ENSG00000205531:2979629-2980164:target		51 0 51
3277	HMGN1	ENSG00000205581:40717072-40717200:source		37 0 35
3278	HMGN1	ENSG00000205581:40719921-40720265:target		20 8 0 37
3279	HLA-H	ENSG00000206341:29855732-29856027:source		27 12 0 26 18

Gene LSV ID LSV Type ← More in Healthy | More in ALL →

#	Gene	LSV ID	LSV Type	← More in Healthy | More in ALL →
3280	HLA-H	ENSG00000206341:29858041-29858119:source		20 0 19
3281	HLA-H	ENSG00000206341:29910830-29911286:target		6 41 0 46
3282	RAB12	ENSG00000206418:8624936-8624996:source		46 0 46
3283	HLA-A	ENSG00000206503:29911899-29912174:source		21 9 0 38
3284	HLA-A	ENSG00000206503:29912277-29912411:source		14 0 20
3285	SFT2D2	ENSG00000213064:168200752-168200838:source		26 0 26
3286	CHUK	ENSG00000213341:101952216-101953188:target		23 14 0 37
3287	CHUK	ENSG00000213341:101964833-101964956:source		31 0 31
3288	CHUK	ENSG00000213341:101969352-101969546:target		36 0 36
3289	CHUK	ENSG00000213341:101978475-101978582:source		58 0 58
3290	CHUK	ENSG00000213341:101979027-101979116:target		56 0 56
3291	COG8	ENSG00000213380:69354043-69354949:source		23 0 23
3292	SIPA1	ENSG00000213445:65408272-65409071:source		27 0 27
3293	SIPA1	ENSG00000213445:65409934-65410110:target		38 0 38
3294	SRA1	ENSG00000213523:139918504-139918668:target		7 21 0 34
3295	DDAH2	ENSG00000213722:31696228-31696301:target		44 0 44
3296	DDX47	ENSG00000213782:12967065-12967592:source		32 0 28
3297	DDX47	ENSG00000213782:12974589-12974660:target		34 0 34
3298	DDX47	ENSG00000213782:12976137-12976253:source		30 0 30
3299	AP1G2	ENSG00000213983:24032589-24032711:target		23 0 22

Gene LSV ID LSV Type ← More in Healthy | More in ALL →

#	Gene	LSV ID	LSV Type	← More in Healthy | More in ALL →
3300	GANC	ENSG00000214013:42570680-42570790:source		24 0 147
3301	GANC	ENSG00000214013:42621504-42621702:target		30 0 30
3302	TTLL3	ENSG00000214021:9860987-9862725:target		12 0 21
3303	TTLL3	ENSG00000214021:9876365-9876591:target		25 0 22
3304	SMIM7	ENSG00000214046:16757818-16758072:source		20 0 23
3305	CPNE1	ENSG00000214078:34220717-34220845:source		20 10 0 1810
3306	LYRM4	ENSG00000214113:5108653-5109725:source		55 0 54
3307	PLEKHM1P	ENSG00000214176:62800948-62801218:source		27 0 27
3308	GTF2IRD2P1	ENSG00000214544:72680571-72680635:source		20 0 21
3309	ZSWIM8	ENSG00000214655:75550733-75550934:source		50 0 50
3310	ZSWIM8	ENSG00000214655:75551617-75552248:target		36 0 36
3311	HNRNPUL2	ENSG00000214753:62482972-62483079:source		35 0 35
3312	HNRNPUL2	ENSG00000214753:62484461-62484661:target		30 0 30
3313	PEX26	ENSG00000215193:18562640-18562780:target		51 0 51
3314	CTD-3092A11.1	ENSG00000215302:30771227-30771396:source		22 0 16
3315	RP13-104F24.2	ENSG00000215769:62758034-62758165:target		21 0 21
3316	NBPF1	ENSG00000219481:16907915-16908311:source		59 0 59
3317	PPP3R1	ENSG00000221823:68479257-68479663:target		20 0 20
3318	UBA52	ENSG00000221983:18682665-18682719:source		25 0 25
3319	UBA52	ENSG00000221983:18684075-18684213:target		24 0 31

Gene LSV ID LSV Type ← More in Healthy | More in ALL →

#	Gene	LSV ID	LSV Type	← More in Healthy | More in ALL →
3320	NUTM2A-AS1	ENSG00000223482:89086387-89086431:source		6 21 0 31
3321	AC008277.1	ENSG00000223642:160252280-160252345:source		50 0 50
3322	AC008277.1	ENSG00000223642:160253856-160253900:target		48 0 48
3323	SNHG14	ENSG00000224078:25599500-25599573:source		27 0 27
3324	SNHG14	ENSG00000224078:25601039-25601203:target		36 0 36
3325	LAMTOR5-AS1	ENSG00000224699:110948940-110949001:source		31 0 31
3326	HCG4P11	ENSG00000225864:29690758-29691748:source		9 0 20
3327	RP4-635E18.7	ENSG00000226849:11130716-11133154:source		24 0 22
3328	WASH7P	ENSG00000227232:16854-17055:source		24 0 24
3329	ENSG00000227558	ENSG00000227558:69084846-69084959:source		28 0 17 7
3330	RP11-69E11.8	ENSG00000228060:40035535-40035674:source		21 0 21
3331	EFCAB14-AS1	ENSG00000228237:47152456-47152542:source		35 0 35
3332	EFCAB14-AS1	ENSG00000228237:47155259-47155363:target		32 0 32
3333	ARL17B	ENSG00000228696:44414638-44414736:source		21 0 21
3334	HLA-DRB6	ENSG00000229391:32521633-32522271:source		6 29 0 35
3335	HLA-DRB6	ENSG00000229391:32522396-32523474:source		21 0 25
3336	HLA-DRB6	ENSG00000229391:32548572-32548582:target		40 0 40
3337	HLA-DRB6	ENSG00000229391:32549138-32549615:target		20 0 24
3338	HCG4P7	ENSG00000230521:29855071-29856045:source		23 0 10 15
3339	HLA-K	ENSG00000230795:29911701-29912070:target		49 0 46

| # | Gene | LSV ID | LSV Type | ← More in Healthy \| More in ALL → |

#	Gene	LSV ID	LSV Type	← More in Healthy | More in ALL →
3340	TAPBP	ENSG00000231925:33267471-33269548:source	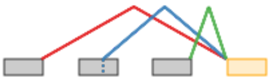	28 10 0 41
3341	TAPBP	ENSG00000231925:33272045-33272415:target	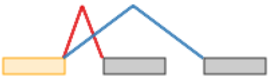	44 0 44
3342	GS1-124K5.12	ENSG00000232559:66038372-66038538:source	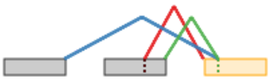	36 0 36
3343	ENSG00000232637	ENSG00000232637:146052552-146052763:source	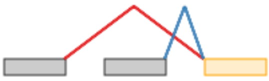	22 0 22
3344	ENSG00000232637	ENSG00000232637:146250092-146251009:target	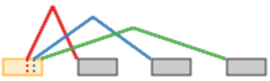	5 20 0 28
3345	IDI2-AS1	ENSG00000232656:1089424-1089535:target	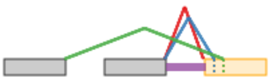	21 0 19
3346	CTD-2020K17.4	ENSG00000233483:43322553-43322783:source	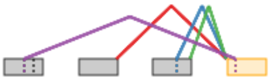	34 0 33
3347	CTD-2020K17.4	ENSG00000233483:43323245-43323340:target	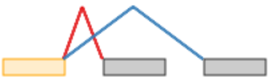	40 0 40
3348	TRIM26	ENSG00000234127:30156944-30156976:source	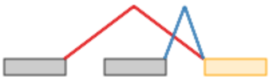	22 0 22
3349	RP11-166B2.1	ENSG00000234719:12021230-12021736:source	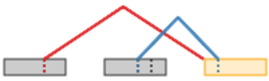	20 0 20
3350	HNRNPUL2-BSCL2	ENSG00000234857:62482972-62483079:source	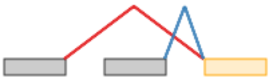	35 0 35
3351	HNRNPUL2-BSCL2	ENSG00000234857:62484461-62484661:target	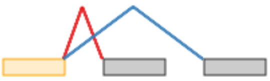	31 0 31
3352	AC009303.1	ENSG00000235066:118753840-118754186:source	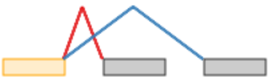	55 0 55
3353	AC009303.1	ENSG00000235066:118764298-118764392:target	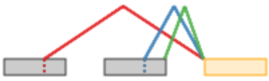	50 0 55
3354	MYCBP2-AS1	ENSG00000236051:77661619-77661913:source	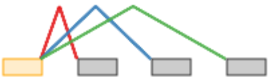	21 42 0 61
3355	MYCBP2-AS1	ENSG00000236051:77664241-77664398:target	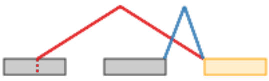	50 0 50
3356	AC002401.1	ENSG00000236472:48217458-48217724:source	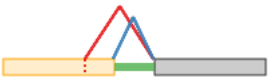	57 0 57
3357	AC002401.1	ENSG00000236472:48218634-48218738:target	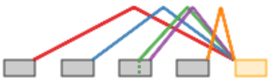	25 33 0 64
3358	AC107081.5	ENSG00000236498:62095567-62096000:source	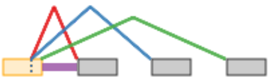	43 0 43
3359	AC107081.5	ENSG00000236498:62099217-62099451:target	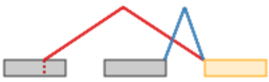	64 0 64

Gene LSV ID LSV Type ← More in Healthy | More in ALL →

#	Gene	LSV ID	LSV Type	← More in Healthy | More in ALL →
3360	TTN-AS1	ENSG00000237298:179402331-179402644:source		47 0 47
3361	TTN-AS1	ENSG00000237298:179403267-179404693:target		11 19 14 0 45
3362	TXNDC5	ENSG00000239264:7891854-7891969:target		23 0 23
3363	ASB14	ENSG00000239388:57302375-57303728:source		29 0 29
3364	WBP1	ENSG00000239779:74686770-74686874:target		29 9 0 34
3365	PSMB9	ENSG00000240065:32814845-32814981:source		41 0 41
3366	PSMB9	ENSG00000240065:32816429-32816617:source		46 0 46
3367	PSMB9	ENSG00000240065:32818097-32818294:target		47 0 47
3368	ACAD11	ENSG00000240303:132298023-132298402:target		26 0 26
3369	RP4-714D9.2	ENSG00000241073:100533533-100533751:source		24 0 26
3370	RP4-714D9.2	ENSG00000241073:100535170-100535241:target		20 0 20
3371	HLA-DOB	ENSG00000241106:32781217-32781248:source		31 0 31
3372	HLA-DOB	ENSG00000241106:32782097-32782378:target		37 0 37
3373	ENSG00000241287	ENSG00000241287:32629606-32630130:target		30 0 32
3374	PPP1R2P4	ENSG00000241353:46933621-46933766:source		15 0 20
3375	PPP1R2P4	ENSG00000241353:46935576-46935708:source		24 0 24
3376	PPP1R2P4	ENSG00000241353:46937250-46937348:target		15 13 0 30
3377	PPP1R2P4	ENSG00000241353:46942177-46942384:source		31 0 27
3378	PPP1R2P4	ENSG00000241353:46942868-46942950:target		30 0 30
3379	PPP1R2P4	ENSG00000241353:46946404-46946732:source		10 5 0 13 23

Gene LSV ID LSV Type ← More in Healthy | More in ALL →

#	Gene	LSV ID	LSV Type	← More in Healthy | More in ALL →
3380	PPP1R2P4	ENSG00000241353:46960453-46960860:target	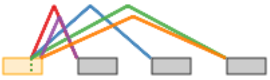	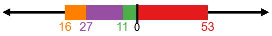 16 27 11 0 53
3381	ATP5J2	ENSG00000241468:99055813-99055978:source	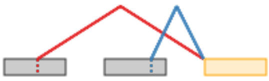	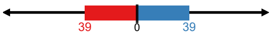 39 0 39
3382	ATP5J2	ENSG00000241468:99057278-99058122:target	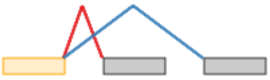	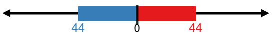 44 0 44
3383	PI4KA	ENSG00000241973:21064124-21064285:target	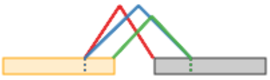	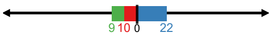 9 10 0 22
3384	AKAP2	ENSG00000241978:112900451-112900819:source	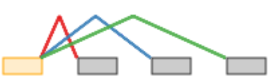	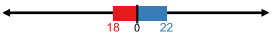 18 0 22
3385	AKAP2	ENSG00000241978:112918599-112918777:target	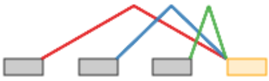	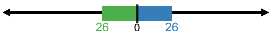 26 0 26
3386	HYPK	ENSG00000242028:44092509-44092982:source	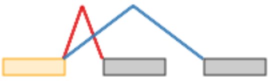	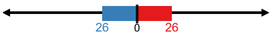 26 0 26
3387	HYPK	ENSG00000242028:44093716-44093788:target	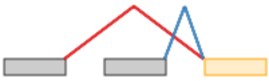	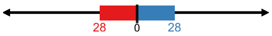 28 0 28
3388	C22orf39	ENSG00000242259:19373044-19373259:source	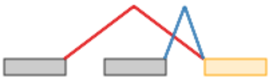	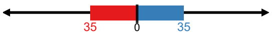 35 0 35
3389	C22orf39	ENSG00000242259:19376007-19376077:target	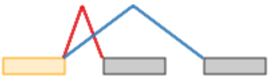	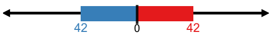 42 0 42
3390	C22orf39	ENSG00000242259:19393309-19393403:target	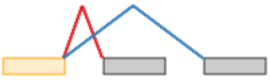	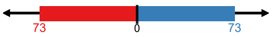 73 0 73
3391	C22orf39	ENSG00000242259:19395797-19396116:target	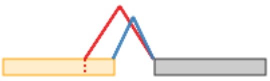	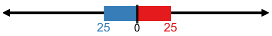 25 0 25
3392	RP11-274B21.14	ENSG00000242588:128266349-128266584:source	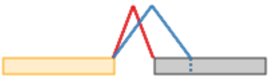	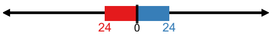 24 0 24
3393	MICAL3	ENSG00000243156:18273968-18274433:source	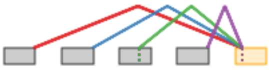	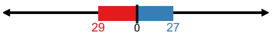 29 0 27
3394	MICAL3	ENSG00000243156:18293469-18293579:target	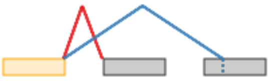	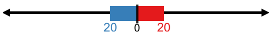 20 0 20
3395	MICAL3	ENSG00000243156:18383608-18383763:source	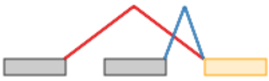	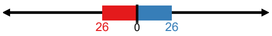 26 0 26
3396	ENSG00000243452	ENSG00000243452:148558169-148558286:source	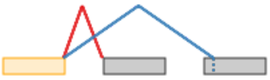	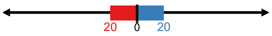 20 0 20
3397	NPIPB5	ENSG00000243716:22544947-22547842:target	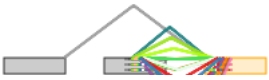	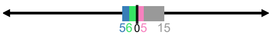 5 6 0 5 15
3398	RP11-148K1.12	ENSG00000244151:150773083-150773617:target	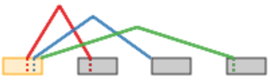	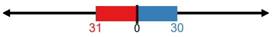 31 0 30
3399	CTD-2013N24.2	ENSG00000244513:69074711-69074863:source	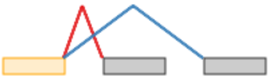	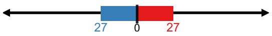 27 0 27

\# Gene LSV ID LSV Type ← More in Healthy | More in ALL →

#	Gene	LSV ID	LSV Type	← More in Healthy | More in ALL →
3400	CTD-2013N24.2	ENSG00000244513:69077051-69077143:target	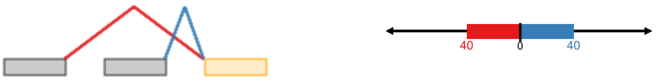	40 0 40
3401	CTD-2260A17.2	ENSG00000247121:96237210-96237385:target	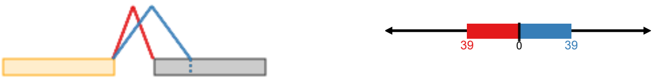	39 0 39
3402	CTD-2260A17.2	ENSG00000247121:96244665-96244821:source	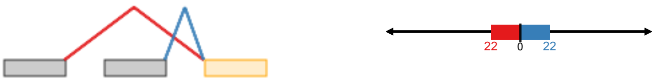	22 0 22
3403	CTD-2260A17.2	ENSG00000247121:96248341-96248502:target	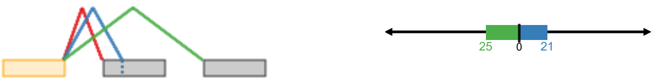	25 0 21
3404	RP5-940J5.6	ENSG00000247853:6690815-6690980:source	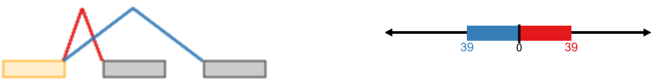	39 0 39
3405	XXbac-BPG181M17.5	ENSG00000248993:32917388-32918157:source	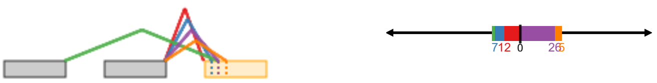	7 12 0 26 5
3406	XXbac-BPG181M17.5	ENSG00000248993:32918296-32918580:source	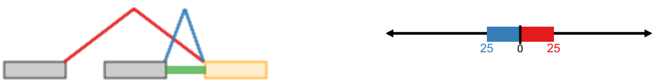	25 0 25
3407	XXbac-BPG181M17.5	ENSG00000248993:32918296-32918580:target	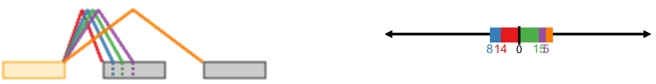	8 14 0 15 5
3408	XXbac-BPG181M17.5	ENSG00000248993:32920726-32920899:target	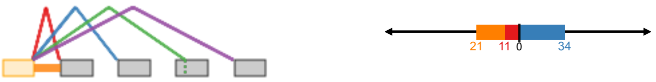	21 11 0 34
3409	NAIP	ENSG00000249437:70293948-70294650:target	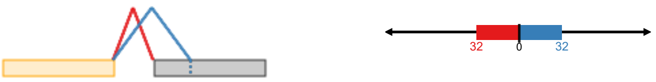	32 0 32
3410	ARPC4-TTLL3	ENSG00000250151:9839342-9839461:source	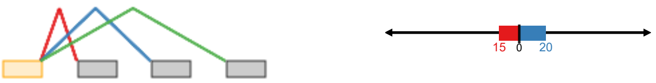	15 0 20
3411	ARPC4-TTLL3	ENSG00000250151:9845527-9845697:source	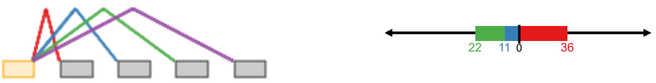	22 11 0 36
3412	ARPC4-TTLL3	ENSG00000250151:9854627-9854795:target	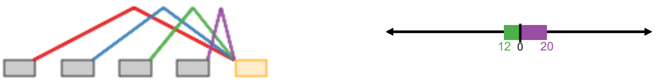	12 0 20
3413	ARPC4-TTLL3	ENSG00000250151:9862230-9862425:target	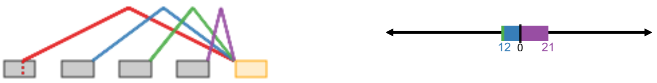	12 0 21
3414	ARPC4-TTLL3	ENSG00000250151:9876365-9877085:target	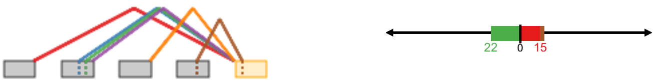	22 0 15
3415	THAP9-AS1	ENSG00000251022:83818916-83819215:source	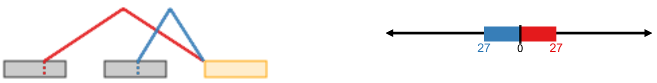	27 0 27
3416	THAP9-AS1	ENSG00000251022:83820976-83821376:target	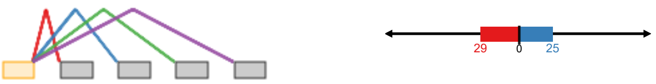	29 0 25
3417	MALAT1	ENSG00000251562:65265233-65269864:source	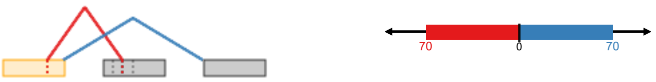	70 0 70
3418	MALAT1	ENSG00000251562:65270108-65273940:target	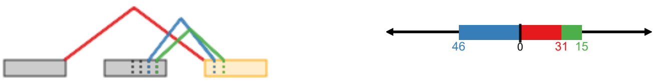	46 0 31 15
3419	ENSG00000251606	ENSG00000251606:96518712-96518944:target	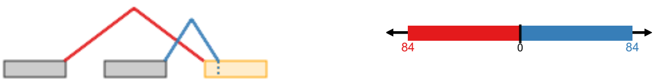	84 0 84
#	Gene	LSV ID	LSV Type	← More in Healthy | More in ALL →

#	Gene	LSV ID	LSV Type	← More in Healthy | More in ALL →
3420	TUG1	ENSG00000253352:31366663-31367765:source		30 0 10 19
3421	TUG1	ENSG00000253352:31368249-31369342:target		36 0 15 11 10
3422	RP11-557C18.3	ENSG00000253607:124031435-124031545:source		21 0 21
3423	PRKDC	ENSG00000253729:48690247-48690435:source		21 0 21
3424	PRKDC	ENSG00000253729:48805817-48805947:source		27 0 27
3425	LYN	ENSG00000254087:56859007-56859052:target		37 0 37
3426	LYN	ENSG00000254087:56879274-56879456:source		60 0 60
3427	LYN	ENSG00000254087:56882276-56882540:target		41 0 40
3428	CHKB-CPT1B	ENSG00000254413:51011949-51012144:source		20 0 20
3429	RP11-108K14.8	ENSG00000254536:135216145-135216277:source		21 0 21
3430	SMG1P6	ENSG00000254634:29465337-29465435:target		38 0 26 12
3431	RP11-1415C14.4	ENSG00000254701:69493082-69493223:target		27 0 32
3432	IGLL5	ENSG00000254709:23237555-23238287:target		22 0 22
3433	ATP6V1G2-DDX39B	ENSG00000254870:31503144-31503262:target		58 0 61
3434	STX16-NPEPL1	ENSG00000254995:57242857-57243183:source		23 0 23
3435	STX16-NPEPL1	ENSG00000254995:57266780-57267262:target		27 0 27
3436	ANKHD1-EIF4EBP3	ENSG00000254996:139889604-139889766:source		28 0 28
3437	ANKHD1-EIF4EBP3	ENSG00000254996:139892948-139893064:target		22 0 22
3438	ANKHD1-EIF4EBP3	ENSG00000254996:139917671-139917846:source		21 7 0 34
3439	ENSG00000255168	ENSG00000255168:145296357-145296571:source		38 0 38

Gene LSV ID LSV Type ← More in Healthy | More in ALL →

#	Gene	LSV ID	LSV Type	← More in Healthy | More in ALL →
3440	PDXDC2P	ENSG00000255185:70055384-70055852:target		41 0 41
3441	PSMA1	ENSG00000256206:14540543-14540587:source		28 24 0 52
3442	A2ML1-AS1	ENSG00000256661:8933171-8933335:target		44 0 35
3443	RP11-727F15.9	ENSG00000256690:62601035-62601515:source		20 0 20
3444	RP11-727F15.9	ENSG00000256690:62601035-62601515:target		42 0 22 20
3445	RP11-512M8.5	ENSG00000256861:122701035-122701131:target		43 0 43
3446	KIAA1147	ENSG00000257093:141356528-141362779:source		12 30 0 44
3447	LSM14A	ENSG00000257103:34699101-34699956:source		60 0 60
3448	LSM14A	ENSG00000257103:34706501-34706566:target		53 0 53
3449	RP11-571M6.7	ENSG00000257342:58112776-58112965:source		22 0 22
3450	RP11-203J24.9	ENSG00000257524:130636890-130636928:source		29 0 29
3451	PSMA3-AS1	ENSG00000257621:58764673-58764852:target		27 0 8 18
3452	CUX1	ENSG00000257923:101838787-101838883:source		48 0 7 38
3453	RP11-864J10.4	ENSG00000258136:108135998-108136125:source		9 6 0 25
3454	RP11-864J10.4	ENSG00000258136:108136974-108137059:source		21 0 21
3455	RP11-864J10.4	ENSG00000258136:108140052-108140201:target		16 6 0 22
3456	RP11-977G19.5	ENSG00000258199:56565055-56565216:source		26 0 26
3457	RP11-977G19.5	ENSG00000258199:56566212-56566274:target		26 0 26
3458	RP11-478C19.2	ENSG00000258210:110873915-110874009:source		10 28 0 39
3459	RP11-478C19.2	ENSG00000258210:110874889-110874957:target		38 0 38
#	Gene	LSV ID	LSV Type	← More in Healthy | More in ALL →

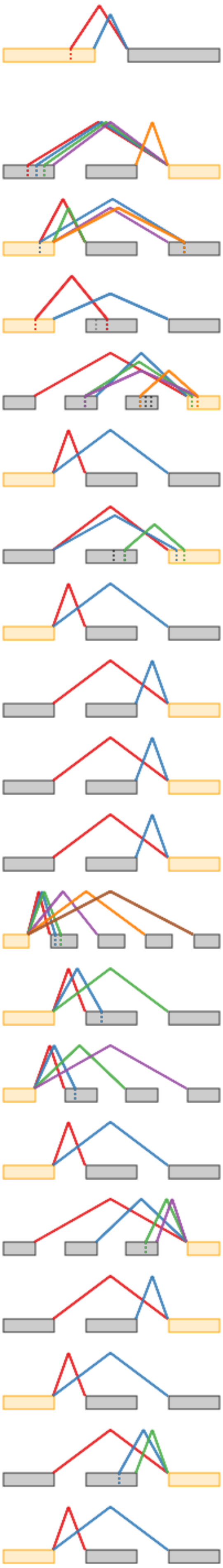

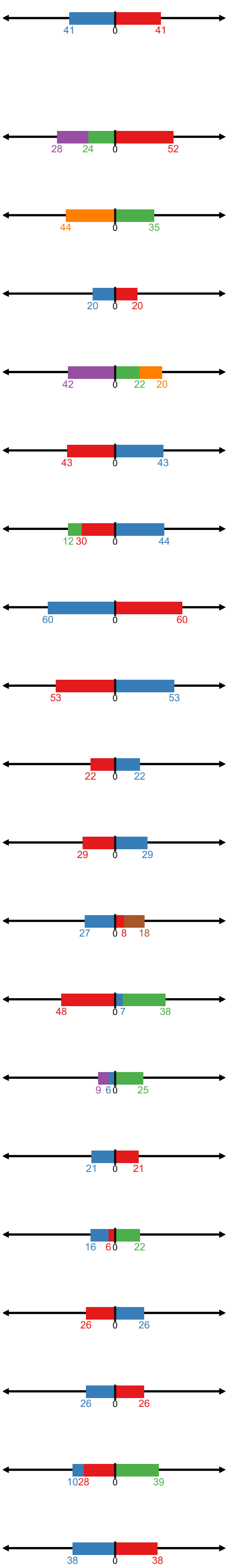

#	Gene	LSV ID	LSV Type	← More in Healthy | More in ALL →
3460	RP11-968A15.8	ENSG00000258344:54678042-54678101:source		26 0 26
3461	RP11-574F21.3	ENSG00000258465:160188639-160188758:source		43 0 44
3462	RP11-574F21.3	ENSG00000258465:160194232-160194339:source		34 0 34
3463	RP11-574F21.3	ENSG00000258465:160195381-160195453:target		37 0 37
3464	RP11-108O10.8	ENSG00000258529:111708191-111708338:source		25 0 25
3465	RP5-1021I20.4	ENSG00000258653:74387683-74387808:source		30 0 30
3466	RP5-1021I20.4	ENSG00000258653:74390098-74390225:target		24 0 23
3467	RP11-66N24.3	ENSG00000258727:24032589-24032711:target		23 0 22
3468	KIAA0391	ENSG00000258790:35777200-35777294:target		30 0 28
3469	KIAA0391	ENSG00000258790:35779946-35780101:source		21 0 21
3470	CEP95	ENSG00000258890:62522189-62522318:target		27 0 27
3471	RP5-1021I20.5	ENSG00000258891:74387683-74387808:source		31 0 31
3472	RP5-1021I20.5	ENSG00000258891:74390098-74390225:target		23 0 23
3473	RP11-1017G21.4	ENSG00000258959:102506935-102507010:source		23 0 23
3474	UBE2F-SCLY	ENSG00000258984:238903386-238903451:source		31 0 36
3475	UBE2F-SCLY	ENSG00000258984:238933983-238934053:target		28 0 35
3476	BLOC1S5-TXNDC5	ENSG00000259040:7891854-7891969:target		23 0 23
3477	RP11-1070N10.6	ENSG00000259084:96129593-96131413:source		6 20 0 10 17
3478	RP11-1070N10.6	ENSG00000259084:96135888-96135954:target		26 0 15 12
3479	RP11-815J21.4	ENSG00000259367:86207794-86207986:target		30 0 13 18

Gene LSV ID LSV Type ← More in Healthy | More in ALL →

#	Gene	LSV ID	LSV Type	← More in Healthy | More in ALL →
3480	TGIF2-C20orf24	ENSG00000259399:35236118-35236221:target	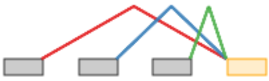	14 14 0 31
3481	RP11-317G6.1	ENSG00000259589:63998979-63999030:source	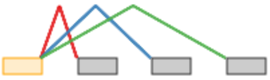	7 21 0 31
3482	RP11-382A20.5	ENSG00000259805:83698888-83699080:target	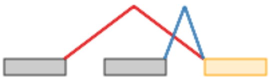	25 0 25
3483	RP11-24N18.1	ENSG00000260570:28843508-28843720:source	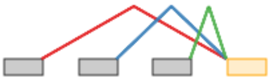	15 0 23
3484	RP11-24N18.1	ENSG00000260570:28843817-28843888:source	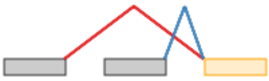	33 0 33
3485	RP11-24N18.1	ENSG00000260570:28844380-28844675:target	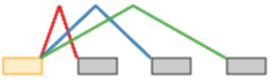	10 14 0 26
3486	RP11-382A20.7	ENSG00000260608:83698888-83699080:target	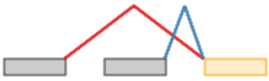	25 0 25
3487	RP11-23N2.4	ENSG00000260618:52876942-52877141:source	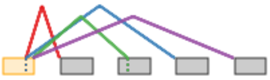	28 0 28
3488	RP11-23N2.4	ENSG00000260618:52885775-52885933:target	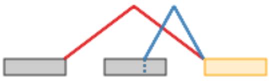	27 0 27
3489	AL136419.6	ENSG00000260669:24680617-24680798:target	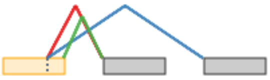	9 8 0 20
3490	ENSG00000261337	ENSG00000261337:71957191-71957283:source	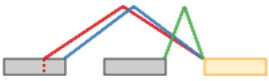	54 0 53
3491	ENSG00000261337	ENSG00000261337:71958672-71958720:target	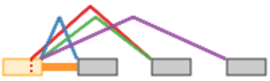	18 29 0 36 15
3492	RP11-345J4.5	ENSG00000261740:29458123-29458347:source	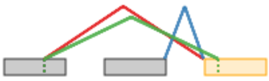	5 25 0 32
3493	RP11-345J4.5	ENSG00000261740:29463430-29465434:target	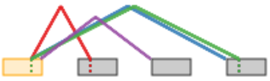	38 0 26 12
3494	CTD-2047H16.4	ENSG00000263069:78355086-78355943:source	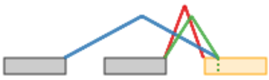	10 13 0 23
3495	UBBP4	ENSG00000263563:21731000-21731357:source	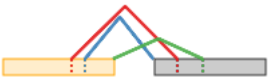	43 0 43
3496	RP11-45M22.3	ENSG00000263624:17076012-17076129:source	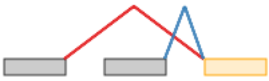	24 0 24
3497	RP11-45M22.3	ENSG00000263624:17077230-17077389:source	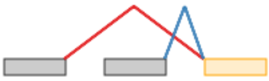	30 0 30
3498	RP11-45M22.3	ENSG00000263624:17079740-17079826:target	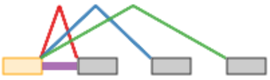	14 20 0 9 25
3499	ABHD15-AS1	ENSG00000264031:28003838-28003903:target	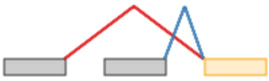	24 0 24

| # | Gene | LSV ID | LSV Type | ← More in Healthy \| More in ALL → |

#	Gene	LSV ID	LSV Type	← More in Healthy | More in ALL →
3500	BZRAP1-AS1	ENSG00000265148:56429360-56431080:target		19 0 17 5
3501	RP11-21J18.1	ENSG00000265257:9195549-9195696:source		23 0 27
3502	RP11-21J18.1	ENSG00000265257:9216756-9216898:source		25 0 25
3503	RP11-21J18.1	ENSG00000265257:9221850-9221997:target		25 0 25
3504	MIR1539	ENSG00000265496:47017996-47018248:target		9 12 0 13 8
3505	RPL17	ENSG00000265681:47017887-47017956:source		50 14 0 36 35
3506	RPL17	ENSG00000265681:47017996-47018210:target		29 0 13 16
3507	RP11-703M24.5	ENSG00000266049:2718313-2718635:source		29 0 34
3508	RP11-703M24.5	ENSG00000266049:2724897-2724993:target		23 0 25
3509	RP11-159D12.5	ENSG00000266086:56082284-56082614:target		66 0 62
3510	MYO15B	ENSG00000266714:73586707-73586770:target		35 0 35
3511	MYO15B	ENSG00000266714:73588319-73588382:source		16 0 21
3512	MYO15B	ENSG00000266714:73592541-73592636:target		37 0 11 28
3513	MYO15B	ENSG00000266714:73616974-73617256:target		23 0 17
3514	RP11-120M18.2	ENSG00000267009:66511535-66511717:target		21 0 22
3515	ENSG00000267025	ENSG00000267025:34161570-34161602:target		52 0 52
3516	CTD-2020K17.1	ENSG00000267121:43297634-43297821:source		30 0 30
3517	CTD-2020K17.1	ENSG00000267121:43298716-43299620:target		30 0 30
3518	AC010642.1	ENSG00000267216:58817454-58817582:source		33 0 33
3519	CTC-548K16.5	ENSG00000267379:14516560-14516751:source		31 0 31
#	Gene	LSV ID	LSV Type	← More in Healthy | More in ALL →

#	Gene	LSV ID	LSV Type	← More in Healthy | More in ALL →
3520	CTC-548K16.5	ENSG00000267379:14517681-14517772:source		26 0 36
3521	CTC-559E9.4	ENSG00000267581:19932410-19932525:target		22 0 22
3522	RP11-729L2.2	ENSG00000267699:48575665-48575694:source		6 26 0 34
3523	RP11-729L2.2	ENSG00000267699:48581151-48581363:target		20 0 16
3524	NAPA-AS1	ENSG00000268061:48003890-48004006:target		29 0 29
3525	AC137932.4	ENSG00000268218:89336345-89339452:target		17 8 0 27
3526	L34079.2	ENSG00000268361:44065062-44065172:source		25 0 25
3527	RP1-283E3.8	ENSG00000268575:1635263-1635434:source		29 0 29
3528	RP1-283E3.8	ENSG00000268575:1635662-1635783:target		21 0 21
3529	RP1-283E3.8	ENSG00000268575:1654027-1654270:target		23 0 28
3530	CTC-429P9.4	ENSG00000268790:16757907-16758072:source		23 0 23
3531	CTD-2207O23.3	ENSG00000268861:7447948-7448089:source		21 0 21
3532	ENSG00000269279	ENSG00000269279:116926634-116926746:target		29 0 29
3533	AC003002.6	ENSG00000269533:57905556-57905591:target		26 0 26
3534	EGLN2	ENSG00000269858:41313714-41314013:target		41 0 41
3535	SNHG8	ENSG00000269893:119200544-119200586:target		29 0 29
3536	MINOS1-NBL1	ENSG00000270136:19952881-19952970:target		25 0 25
3537	BNIP3P11	ENSG00000271550:64139332-64139714:source		26 0 19
3538	POLR2J4	ENSG00000272655:44053161-44054382:source		20 0 20
3539	STAG3L5P-PVRIG2P-PILRB	ENSG00000272752:99948875-99949033:source		33 0 33

| # | Gene | LSV ID | LSV Type | ← More in Healthy \| More in ALL → |

#	Gene	LSV ID	LSV Type	← More in Healthy | More in ALL →
3540	STAG3L5P-PVRIG2P-PILRB	ENSG00000272752:99949786-99949834:target		35 0 37
3541	CTD-2410N18.5	ENSG00000272772:133502861-133502934:source		31 0 31
3542	CTD-2410N18.5	ENSG00000272772:133509617-133509713:target		21 0 21
3543	CTD-2410N18.5	ENSG00000272772:133534777-133534895:source		48 0 48
3544	CTD-2410N18.5	ENSG00000272772:133536676-133536765:target		52 0 52
3545	LINC01578	ENSG00000272888:93425937-93426416:source		21 0 21
3546	LINC01578	ENSG00000272888:93426815-93426849:target		31 0 31
3547	LINC01578	ENSG00000272888:93429399-93430215:source		39 0 41
3548	LINC01578	ENSG00000272888:93435234-93435302:target		43 0 43
3549	RP1-309K20.6	ENSG00000272897:34220717-34220845:source		29 13 0 35
3550	RP11-574K11.31	ENSG00000272916:75561074-75562534:source		22 0 20
3551	C1QTNF3-AMACR	ENSG00000273294:34020692-34020847:source		
#	Gene	LSV ID	LSV Type	← More in Healthy | More in ALL →

10

© BIOCIPHERS · Department of Genetics Perelman School of M
Department of Computer and Information Science School of Eng
University of Pennsylvania Penn

LSV filters

- [x] 5-prime
- [x] 3-prime
- [x] Exon skipping
- [x] Single Source
- [x] Single Target

Number of junctions:
from:
to:

Number of exons:
from:
to:

Additional differentially spliced genes in ALL versus healthy pre-B cells (from HCB) which shared common exons with differentially spliced genes in DKO versus WT HCT116 cells.

MAJIQ has been conducted with 10 ALL selected in order to remove the 2 extreme samples (higher and lower) when considering the CD44 expression level (Sup Fig S5C).

Gene	LSV ID	LSV Type	More in healthy | More in leu	Coordinates Links
**NKTR**				
NKTR	ENSG00000114857:42660512-42660619:source		29 0 29	UCSC
NKTR	ENSG00000114857:42674093-42674604:source		34 0 34	UCSC
NKTR	ENSG00000114857:42672038-42672067:source		28 0 28	UCSC
NKTR	ENSG00000114857:42661156-42661200:target		23 0 23	UCSC
NKTR	ENSG00000114857:42674701-42675227:target		26 0 26	UCSC
**MALT1**				
MALT1	ENSG00000172175:56377208-56377304:source		21 0 21	UCSC
**RGS14**				
RGS14	ENSG00000169220:176798874-176799602:target		32 0 32	UCSC
**TYK2**				
TYK2	ENSG00000105397:10463602-10463932:source		30 0 33	UCSC
TYK2	ENSG00000105397:10464204-10464322:source		28 0 28	UCSC
TYK2	ENSG00000105397:10464204-10464322:target		34 0 34	UCSC
TYK2	ENSG00000105397:10475527-10475724:target		25 0 25	UCSC

NISCH

See the Figure 6
