# Supplementary material for: CD44 alternative splicing senses intragenic DNA methylation in tumors via direct and indirect mechanisms
Source: Nucleic Acids Res. 2021 Jun 4;49(11):6213–37. doi: 10.1093/nar/gkab437 (PMC8216461; doi:10.1093/nar/gkab437)
Supplement: gkab437_Supplemental_Files [file gkab437_supplemental_files.zip › Batsche_Sup Table S5_common splicing.pdf]

Common RNA events in DKO and in ALL

		Ensembl ID	Gene Name	Modified exon coordinates		altrernative splicing in DKO	in ALL	Differential expression ALL vs HCB log2FC	pval	RNA level log2 cpm	DKO vs WT log2FC	pval	RNA level log2 cpm
correlated alternative splicing with 5mC changes	DMR up in ALL	ENSG00000134905	CARS2	chr13:111296412-111296529	CE17	up	down			6.3			5.5
		ENSG00000079805	DNM2	chr19:10906737-10906875	CE9/CE10	down	up			8.4			7.4
		ENSG00000164068	RNF123	chr3:49758328-49758453	ri 38	up	down			5.4			5.0
		ENSG00000105397	TYK2	chr19:10464313-10464726	CE21	down	up			7.7			5.7
		ENSG00000124222	STX16	chr20:57244347-57244509	CE6	down	up			7.3	-0.6	2.3E-02	5.6
		ENSG00000003402	CFLAR	chr2:202000918-202003698	CE9	down	up	-1.4	5.1E-05	7.7			5.5
		ENSG00000054967	RELT	chr11:73102189-73102269	CE6	down	up	-1.8	7.8E-08	7.3			3.9
	DMR down in ALL	ENSG00000173209	AHSA2	chr2:61413115-61413216	CE7	down	down			7.4			5.7
		ENSG00000084234	APLP2	chr11:130007149-130007188	CE14	down	down			7.5			9.3
		ENSG00000149182	ARFGAP2	chr11:47197402-47197475	CE3	up	up			6.9			5.7
		ENSG00000165322	ARHGAP12	chr10:32142994-32143134	CE4	up	up			5.5			6.3
		ENSG00000026508	CD44	chr11:35211069-35235288	var. exons	down	down			7.3			8.1
		ENSG00000196776	CD47	chr3:107768466-107768519	CE9 CE10	down	down			8.8			5.8
		ENSG00000168958	MFF	chr2:228217230-228217289	CE10	up	up			6.2			6.0
		ENSG00000134186	PRPF38B	chr1:109240429-109240641	CE7a b	up	up			7.6			7.5
		ENSG00000136527	TRA2B	chr3:185649365-185649640	CE4	down	down			9.6			8.4
		ENSG00000132155	RAF1	chr3:12627180-12627298	CE15	down	down			7.7			6.8
		ENSG00000102531	FNDC3A	chr13:49749503-49749677	CE15	up	up			6.5	-0.7	3.5E-02	5.4
		ENSG00000169220	RGS14	chr5:176798474-176798591	CE14	up	up			4.7	1.7	4.9E-03	3.1
		ENSG00000198106	BOLA2/SMG1[illegible]	chr16:29464899-29465054	TE2	up	up	-3.0	2.0E-07	2.2			-1.8
		ENSG00000164548	TRA2A	chr7:23561734-23562057	CE3	down	down	-2.5	6.6E-08	8.9			7.4
		ENSG00000141480	ARRB2	chr17:4621556-4621640	CE9	up	up	1.3	2.5E-06	6.7			5.9
		ENSG00000166734	CASC4	chr15:44695085-44695252	CE9	down	down	1.1	4.1E-04	5.6			6.5
		ENSG00000010322	NISCH	chr3:52512463-52512597	E15-3'SS	L up	L up	1.4	1.8E-07	7.2			6.3
uncertain	DMRs	ENSG00000031003	FAM13B	chr5:137354644-137354835	CE3	up	up			6.5	0.6	4.1E-02	5.6
		ENSG00000168036	CTNNB1	chr3:41268700-41268843	TE17	up	down	-1.1	1.7E-04	8.6			8.8
		ENSG00000160584	SIK3	chr11:116738662-116738805	CE14	down	up	-2.5	2.9E-09	6.9			5.6
		ENSG00000163597	SNHG16	chr17:74555027-74555125	CE2	up	up	-1.5	3.7E-07	4.7			7.3
		ENSG00000073921	PICALM	chr11:85701293-85701421	CE13	up	down	1.1	6.0E-05	6.9			6.8
opposite 5mC changes	between DKO and ALL	ENSG00000074054	CLASP1	chr2:122203025-122203072	CE20	down	up			7.5			6.0
		ENSG00000047579	DTNBP1	chr6:15651544-15651639	CE3	down	up			5.3			4.7
		ENSG00000150712	MTMR12	chr5:32235068-32235235	CE14/15	down	up			6.2			5.4
		ENSG00000092841	MYL6	chr12:56554410-56554454	CE6	up	down			7.9			8.6
		ENSG00000153914	SREK1	chr5:65451894-65454058	TE6	up	down			9.0			7.7
		ENSG00000100241	SBF1	chr22:50895463-50895542	CE29	up	up			8.0			6.6
		ENSG00000142227	EMP3	chr19:48830087-48830179	CE3	down	up	-2.4	1.4E-05	6.9			5.9
		ENSG00000100084	HIRA	chr22:19392987-19393054	CE6	up	down	-1.6	1.2E-07	4.1			4.0
		ENSG00000119725	ZNF410	chr14:74354976-74355067	CE2	up	down	-3.5	4.0E-09	2.5			1.8
		ENSG00000112200	ZNF451	chr6:56965401-56974512	TE5	up	down	-2.1	3.1E-06	7.8			6.2
		ENSG00000184182	UBE2F	chr2:238925208-238925275	CE5	down	up	-1.4	1.5E-04	4.5	0.6	2.4E-02	4.5
		ENSG00000197694	SPTAN1	chr9:131355262-131355321	CE23	down	up	1.6	1.2E-05	8.9			7.9
5mC absent	or unmodified	ENSG00000114857	NKTR	chr3:42660621-42661155	ri5	up	down			9.7			7.7
		ENSG00000120694	HSPH1	chr13:31722511-31722617	CE10	down	up			6.6			6.1
		ENSG00000153250	RBMS1	chr2:161138769-161138816	CE9	down	up			6.2			4.8
		ENSG00000114062	UBE3A	chr15:25599675-25599830	CE10	down	up			7.1			6.9
		ENSG00000135164	DMTF1	chr7:86824000-86824144	CE19	up	down			8.2	-0.7	7.2E-03	6.2
		ENSG00000116679	IVNS1ABP	chr1:185275617-185275757	CE8	up	down			7.8	-0.7	1.8E-04	6.8
		ENSG00000172175	MALT1	chr18:56378153-56378185	CE7	down	up			6.5	-1.4	2.9E-03	6.5
		ENSG00000154473	BUB3	chr10:124923336-124923641	TE8	down	down	2.1	5.0E-11	6.7			8.3
	Alternative promoters	ENSG00000105643	ARRDC2	chr19:18118969-18119402	FE2	up	up			7.8			5.0
		ENSG00000183624	HMCES	chr3:128997788-128998056	FE2	up	different			7.2			6.2
		ENSG00000120071	KANSL1	chr17:44270189-44270273	FE2	up	down			7.6			6.7
		ENSG00000109180	OCIAD1	chr4:48833241-48833517	FE2	down	down			6.6			7.3
		ENSG00000175482	POLD4	chr11:67120771-67121009	FE2	up	up			4.0			1.1
		ENSG00000129353	SLC44A2	chr19:10736269-10736350	FE2	down	other FE1b			8.4			5.5
		ENSG00000156273	BACH1	chr21:30677518-30677646	FE3	up	down	-2.3	2.8E-05	7.8			6.2
		ENSG00000079332	SAR1A	chr10:71922434-71922803	FE2	down	down	-1.7	6.0E-05	7.6			7.2
		ENSG00000149925	ALDOA	chr16:30076990-30077253	FE9	up	up	2.2	1.9E-10	7.2			9.4
	Different events	ENSG00000107625	DDX50	chr10:70666465-70666770	CE2		different			5.8			5.6
		ENSG00000234719	RP11-166B2.1	chr16:12021225-12021748	TE11		different			-0.8			0.2
		ENSG00000127419	TMEM175	chr4:942198-942407	CE4		different			5.8			3.5
		ENSG00000166887	VPS39	chr15:42484264-42484296	CE3		different			7.3			6.0
		ENSG00000155287	SLC25A28	chr10:101373447-101373684	CE2		different	-1.4	3.8E-06	6.0			5.7
		ENSG00000145901	TNIP1	chr5:150460431-150461011	FE3	down	different	-1.3	2.1E-05	7.1	1.5	2.7E-02	6.0

CE = cassette exon ; FE = first exon ; TE = terminal exon ; SS = splice site ; ri =retention of intron

Supplementary Table S5 Common RNA splicing events in both comparisons : DKO vs WT and ALL vs HCB.
