# Supplementary material for: CD44 alternative splicing senses intragenic DNA methylation in tumors via direct and indirect mechanisms
Source: Nucleic Acids Res. 2021 Jun 4;49(11):6213–37. doi: 10.1093/nar/gkab437 (PMC8216461; doi:10.1093/nar/gkab437)
Supplement: gkab437_Supplemental_Files [file gkab437_supplemental_files.zip › Batsche_Sup Table S6_list of oligos.pdf]

Batsché et al. Supplementary Table S6

list of guide RNA, of siRNA and of primers for PCR

guide RNA for CRISPR/dCas9-DNMT3b			
		sequence (5'->3')	application
CD44-C2_g1	F	CACC GCAGGTTATATTCAAATCTAT	cloning
	R	AAAC ATAGATTTGAATATAACCTGC	
CD44-C2_g2	F	CACC GCATCTGGGCCATTGTGGGCA	cloning
	R	AAAC TGCCCACAATGGCCCAGATGC	
CD44-C5_g1	F	CACC GAAAGGAGCAGCACTTCAGG	cloning
	R	AAAC CCTGAAGTGCTGCTCCTTTC	
CD44-C5_g2	F	CACC GATGGGGTGTACAGTAGAAA	cloning
	R	AAAC TTTCTACTGTACACCCCATC	
CD44-V4_g1	F	CACC GGTTCCACTGGGTCCAGTCC	cloning
	R	AAAC GGACTGGACCCAGTGGAACC	
CD44-V4_g2	F	CACC GCAGACAACCACAAGGATGAC	cloning
	R	AAAC GTCATCCTTGTGGTTGTCTGC	
CD44-V6_g1	F	CACC GAACAGTGGTTTGGCAACAGA	cloning
	R	AAAC TCTGTTGCCAAACCACTGTTC	
CD44-V6_g2	F	CACC GTTTAACAAGTAAATTTGGA	cloning
	R	AAAC TCCAAATTTACTTGTTAAAC	
CD44-V8_g1	F	CACC GCACAGGTTTGGTGGAAGATT	cloning
	R	AAAC AATCTTCCACCAAACCTGTGC	
CD44-V8_g2	F	CACC GAGGAATACAAGTCAATAAAG	cloning
	R	AAAC CTTTATTGACTTGTATTCCTC	
H2BFWT_g1	F	CACC GCCAGGGCCTCAGCCTTTCCC	cloning
	R	AAAC GGGAAAGGCTGAGGCCCTGGC	
H2BFWT_g2	F	CACC GCCCAGAAGCAGAGCAAGCAG	cloning
	R	AAAC CTGCTTGCTCTGCTTCTGGGC	
Non-targeting	F	CACCG GCACTACCAGAGCTAACTCA	cloning
	R	AAAC TGAGTTAGCTCTGGTAGTGCC	

siRNA			
target genes	siRNA name	targeted sequences (5'->3')	use
DNMT1	siDNMT1_1	CCCAATGAGACTGACATCAAA	pool & individually
	siDNMT1_2	CGGGAAGTGAATGGACGTCTA	
DNMT3A	siDNMT3A_1	ACAAAGAAGTGTACACGGA	pool
	siDNMT3A_2	CTCCAGATGTTCTTCGCTA	
DNMT3B	siDNMT3B_1S	AGAUGACGGAUGCCUAGAG	pool
	siDNMT3B_2S	AAGCTCGTCTCCTATCGAA	
MeCP2	siMECP2_7	ACGGAGCGGATTGCAAAGCAA	pool
	siMECP2_8	CACCCAGGTCATGGTGATCAA	
	siMECP2_12	AGGCTGGACACGGAAGCTTAA	
MBD1	siMBD1_8	ACCGGGAACAGAGAATGTTTA	pool
	siMBD1_9	CGCGAAGTCTTTCGCAAGTCA	
	siMBD1_4	CAGGATCCGAAGCAAAGTTGA	
MBD2	siMBD2_8	AAGATGATGCCTAGTAAATTA	pool
	siMBD2_10	CGAAACGATCCTCTCAATCAA	
	siMBD2_7	TGGAAAGATGATGCCTAGTAA	
MBD3	siMBD3_5	GCCGGTGACCAAGATTACCAA	pool
	siMBD3_2	CCCGGAGATGGAGCACGTCTA	
	siMBD3_4	CGGGAAGAAGTTCCGCAGCAA	
MBD4	siMBD4_5	AAGCTTCTCATCGCTACTATA	pool
	siMBD4_6	CACGACGTAAAGCCTTTAAGA	
	siMBD4_3	CCGCCGAATGACCTCCGCAAA	
HP1g	siHP1g-1	ATCTGACAGTGAATCTGAT	pool
	siHP1g-2	AGTACTAGATCGACGTGTA	
ESRP1	siESRP1_3	CACAATGACAGAGTATTTA	pool
	siESRP1_4	CGGTATATTGAGGTTTACA	
ESRP2	siESRP2_1	AGCCCGAGGTGATAAAGCA	pool

	siESRP2_2	GACTTAATCCTCCTAGTTT	
SAM68	siSam_1	GCACCCATATGGACGTTAT	pool
	siSam_2	GAAAGAGCGAGTGCTGATA	
	siSam_3	TATGATGGATGATATCTGT	
	siSam_4	AAAGATCTCTGTATTGGGA	
TRA2B	siTRA2B_1	CCCGATCTGAATCTAGGTCTA	pool
	siTRA2B_2	CGGGACTACTATAGCAGATCA	
	siTRA2B_3	AACCGGGTGCTTCAAAGTACA	
GAPDH	siGAPD_1	CAACGGATTTGGTCGTATT	pool
	siGAPD_2	TGGTTTACATGTTCCAATA	
NT	siNT	TAGCAATGACGAATGCGTA	
PCR primers<br>target genes	primer name	Sequences (5'->3')	application<br>C = ChIP / M=MeDIP<br>R = RT-qPCR or PCR
controls			
RPLP0	PP-F	ACAGAGCGACACTCCGTCTCAAA	C M
	PP-R	ACCTGGCGAGCTCAGCAAACTAAA	
RPLP0	e6F	AGGTGTTCGACAATGGCAGCAT	R
	e7R	TGCAGACAGACACTGGCAACAT	
GAPDH	prom-F	CCCGGTTTCTATAAATTGAGCCCGCA	C M
	prom-R	AAAGAAGATGCGGCTGACTGTCG	
GAPDH	GAPDH-F	TCCCATCACCATCTTCCAGG	R
	GAPDH-R	CATCGCCCCACTTGATTTTG	
HPRT	HPRT-e2.3F	TATGGACAGGACTGAACGTCTTGC	R
	HPRT-e3R	TGAGCACACAGAGGGCTACAAT	
CDK9	CDK9 -e3F	TTACAGCCTTGCGGGAGATCAAGA	R
	CDK9 -e4R	AAGGTCATGCTCGCAGAAGTCGAA	
CCNT1	CCNT1 -e8e9F	CATTTGGAATTGGAGGGCATGCGA	R
	CCNT1 -e9R	GTTGTGTCTGAAGAGCTCTGGGAA	
H19	H19-e2F	AGTGGACTTGGTGACGCTGTAT	R
	H19-e2R	TGGATGCTGTACTGTCTGCCAA	
MAGEB6	Prom_F	CAACTCTGGACTCTCAGGGAATGA	C M
	Prom_R	GGACCCTTGGACTTCTCTATCTGTT	
H2BWT	TSH2B-e1F	ACTACGTCCCAGAAGCAGAGCAA	M
	TSH2B-e1R	AAGAATCCATGACACTCACGGCCT	
DNMTs			
DNMT1	DNMT1-e6F	ACCAGGCAAACCACCATCACATCT	R
	DNMT1-e7R	TCTTCCTGAGGTTTCCGTTTGGCA	
DNMT3A	DNMT3A-e10F	AGGTGCAGAACAAGCCCATGATTG	R
	DNMT3A-e11R	ACCCACATGTCCGTGTACACTTCT	
DNMT3B	DNMT3B-e6F	ACAGACGACACAGAGGACACACAT	R
	DNMT3B-e7R	CACCAGGAGAAGCCCTTGATCTTT	
MBDs			
MeCP2	MECP2-e3F	TGGACACGGAAGCTTAAGCAAAGG	R
	MECP2-e4R	TTCTTAGGTGGTTTCTGCTCTCGC	
MBD1	MBD1-e13F	ATGATTCTGCCTCCAAATTGGCCC	R
	MBD1-e14R	CTTGGCAACCAGACTGCTGTATCT	
MBD2	MBD2-e3F	CCCACAACGAATGAATGAACAGCC	R
	MBD2-e4R	AAGTCCTTGTAGCCTCTTCTCCCA	
MBD3	MBD3-e1F	TTTACTATAGCCCGAGCGGGAAGA	R
	MBD3-e2R	GCGGCTCTTGTTCATCTTGCTCAT	
MBD4	MBD4-e5F	AAGAAATGGACACCTCCTCGGTCA	R
	MBD4-e6R	ACATCTCTCCAGTCTGCGGTTCTT	
chromatin modifiers & others			
SUV39H1	SUV39H1-e5F	AGGCGAGGAGCTCACCTTTGATTA	R
	SUV39H1-e6R	AGAAGAGGTATTTGCGGCAGGACT	
SUV39H2	SUV39H2-e3F	TTTCGAACTAGCAATGGACGTGGC	R
	SUV39H2-e4R	ATGAGACACATTGCCGTATCGAGC	
SETDB1	SETDB1-e3F	ACGAATCTTCCCGGCCTACAGAAA	R

	SETDB1-e5R	TTAAGGCAGCCATAGCTTCACGGA	
EHMT1	EHMT1-e12F	ACCAGGGAAGGAAACCTTGGAGA	R
	EHMT1-e13R	AGAAGTACAGCTGCTTTGGGTGGA	
EHMT2	EHMT2-e18F	AATCGGGAACTTGGAGATGGTCAG	R
	EHMT2-e19R	ATCACCTCGATGTGCTTGTGCTCT	
EHMT2	hEhmt2-e10F	GCAAGGCCAAGAAGAAATGGCGAA	R
	hEhmt2-e12R	TCTCGCTGATGCGGTCAATCTT	alt. Splicing
HP1a	HP1a-F	AACAGTGCCGATGACATCAAA	R
	HP1a-R	GCCCCAATGATCTTTTCTGGT	
HP1b	HP1b-F	GCCGGAGCGGATTATTGGAG	R
	HP1b-R	GTGGGCACTTGACATTGGC	
HP1g	HP1g-F	TGCCAGAGGTCTTGATCCTGA	R
	HP1g-R	TCTTTCGCCAGCACCAAGTCT	
AGO1	AGO1-e15F	TGCGTGAGCTCCTCATCCAATTCT	R
	AGO1-e16R	GTGATGGCGTTTCTGCACCACAAT	
AGO2	AGO2-e15F	AGTTCTACAAGTCCACGCGCTTCA	R
	AGO2-e16R	TAGCTTGATACAGGCCTCACGGAT	
splicing factors			
Sam68	KHDRBS1.F1	GTCCTTCACTCACGCCAT	R
	KHDRBS1.R1	AAATAAATCCAAGTAATTCTCCTCATC	
SRSF3	SRSF3-F	AGCGTGTGGATTTGAGCC	R
	SRSF3-R	CATTGTTTCCAAGATTGCCT	
SRSF8	SFRS8-F1	AGGAGAGTCCTTTCAGTGTCG	R
	SFRS8-R1	GCCTATCGGGTGGTTTAACT	
RBFOX2	RBM9.F3	TGGAAATTAAGCCCAGTAGTTG	R
	RBM9.R3	TGATACCCCCTCTTCCTGATAG	
TRA2B	SFRS10.F4	CTAGGCGTTCAAGAGGATTTG	R
	SFRS10.R4	GTGTTGGCGTATGTGGTCTT	
hnRNPM	hnRNPM-e1e2F	ATAAAAAGAGGAGGCAATCGCT	R
	hnRNPM-e3e4R	CACATCCCCTTGACTTTCCTT	
hnRNPA1	HNRPA1.F2	GCAAAACCACGAAACCAAG	R
	HNRPA1.R2	AAAATTATGTCAACACACAAAAAGG	
ESRP1	ESRP1_e7F	CAGAGGCACAAACATCACATaaa	R
	ESRP1_e8R	AGAAACTGGGCTACCTCATTGG	
ESRP2	ESRP2-e1F	ATCCTCCTAGTTTGGCAAGTGG	R
	ESRP2-e3R	CAAAGCCACATCCCCGTTCA	
TIA1	TIA1-e2F.11	AACCTTTCCAGAGATGTGACAGAA	R
	TIA1-e3R.74	TACCCATTATCTTCCGTCCATTCA	
TIAL1	TIAL1-e4F.19	GCAACCACACCAAGTAGCCA	R
	TIAL1-e5R.11	GACTCAAATCCCCAACAAACACA	
alternatively spliced genes			
CyclinD1a	CCND1_e4F	AACTTCCTGTCCTACTACCGCCT	R
	CCND1_e5R	TTGACTCCAGCAGGGCTTCGAT	
CyclinD1b	CCND1_e4F	AACTTCCTGTCCTACTACCGCCT	R
	CCND1_i4R	GCATTTCCGTGGCACTAGGTGT	
PKM2	PKM_e10F	TAATCGTCCTCACCAAGTCTGGCA	R
	PKM_e11R	CATTCATGGCAAAGTTCACCCGGA	
PKM1	PKM_e8F	TGCTGGAGAGCATGATCAAGAAGC	R
	PKM_e9R	AGTTCTTCAAACAGCTTGCGGTGG	
CD44 primers			
Name	Localization	Sequence (5'-3')	application
R=Reverse	C=Constant exon		C = ChIP / M=MeDIP
F = forward	v = variant exon, i = intron		R = RT-qPCR or PCR
CD44-D-F	prom-1232	TGGCTGGGTGCATTTCTCTCAA	M
CD44-D-R	prom-1142	TGAAAGGAACCATGCCAGGACT	
CD44-PPF	C1	TGGCTGGGTGCATTTCTCTCAA	C M
CD44-PPR	C1	TGAAAGGAACCATGCCAGGACT	
CD44-i1F	i1	TGCAGCACATGGCAGAAAGTAACC	C M
CD44-i1R	i1	ACAGGAGCCTGTTAGTCCAGACA	

CD44-C2F	C2	TGCCGCTTTGCAGGTGTATT	C M R
CD44-C2R	C2	GGCAAGGTGCTATTGAAAGCCT	
CD44-i2F	i2	ACAACTGCCACTGGCCAATCAT	C M
CD44-i2R	i2	TAGGCAGGCTGTGTGTGCAAAT	
CD44-iC3F	i2-C3	ACAGGTATGGGTTCATAGAAGGGCAC	C M
CD44-C3R	C3	TGTGTCATACTGGGAGGTGTTGGA	C M R
CD44-i3F	i3	AGAGGGTGCCTTTCTATCTGGCAA	C M
CD44-i3R	i3	TGCTTGGAAAGCTAGACCTGGTGA	
CD44-C4F	C4	ACATCAGTCACAGACCTGCCCAAT	C M R
CD44-C4iR	i4	AACACACCTGAGCCCTTTCCC	C M
CD44-C5F	C5	AGCAGCACTTCAGGAGGTTACA	C M R
CD44-C5R	C5	TGATCCAGGGACTGTCTTCGT	
CD44-i5F	i5	CCAAGGCAAACAACCTGGTGGAAA	C M
CD44-i5R	i5	TGCAATGCATGAGGGAGGCTTT	
CD44-v2F	v2	CAGCAACTGAGACAGCAACCAA	C M R
CD44-v2R	v2	AACCAATCCCAGGTTTCTTGCC	
CD44-i7F	i7	GCAGTGTAGCTTCCATCTCTGTTTGG	C M
CD44-i7R	i7	TGGAAAGGTTTCACACGTGCCT	
CD44-v3F	v3	GGCTGGGAGCCAAATGAAGAAA	C M R
CD44-v3R	v3	CATCATCATCAATGCCTGATCCAGA	
CD44-i8F	i8	AGGTGCATCTCTTATTCCAGCCCT	C M
CD44-i8R	i8	TGAGCAAGCCATTCATAGCCAGTG	
CD44-v4F	v4	CAGTGGAACCCAAGCCATTCAA	C M R
CD44-v4R	v4	CCTTGTGGTTGTCTGAAGTAGCAC	
CD44-i9F	i9	TGGTTTGTGGTTCTGCCTTTGACA	C M
CD44-i9R	i9	AGTGTTACCTCTGAGCATCCACTCTC	
CD44-v5F	v5	GAAACTGGAACCCAGAAGCACA	C M R
CD44-v5R	v5	TGATGCTCATGGTGAATGAGGG	
CD44-i10F	i10	TAGGTGCCATGTGGACATCACCAA	C M
CD44-i10R	i10	ACACACTGGAAATGCACCAGCA	
CD44-v6F	v6	CAGAAGGAACAGTGGTTTGGCA	C M R
CD44-v6R	v6	GTCTTCTTTGGGTGTTTGGCGA	
CD44-i11F	i11	TGGCTCAGAATGAAGCAAGGCA	C M
CD44-i11R	i11	GGCTGGAAGTGATCTCAGGAAAGGAA	
CD44-v7F	v7	TGCAAGGAAGGACAACACCAAG	C M R
CD44-v7R	v7	GGGTGTGAGATTGGGTTGAAGA	
CD44-i12F	i12	TCTGGGATAACAGGGTCACCACAT	C M
CD44-i12R	i12	TGCACTGCAGCCTTTCAACACT	
CD44-v8F	v8	ACGCTTCAGCCTACTGCAAA	C M R
CD44-v8R	v8	AAGAGGTCCTGTCCTGTCCAAA	
CD44-i13F	i13	TTGCACCTGCAACTCAGGTCAGTA	C M
CD44-i13R	i13	ATCACTGCACCAAGGAAACGCT	
CD44-v9F	v9	GAGCTTCTCTACATCACATGAAGGC	C M R
CD44-v9R	v9	GTCAGAGTAGAAGTTGTTGGATGGTC	
CD44-i14F	i14	TTGCGCTGTGGTCCAATAGACTGT	C M
CD44-i14R	i14	ACCATGGAGGTCCAGAGGACAAA	
CD44-v10F	v10	ACCTCTCATTACCCACACACGA	C M R
CD44-v10R	v10	TAGCTGAGGTCACTGGGATGAA	
CD44-i15F	i15	TGCCACATGCTTGGCCTCATTT	C M
CD44-i15R	i15	TTCTTGAAGCTCTTGGGTAGGCCA	
CD44-i16F	i16	AGACATCTAGGGCACAAGGCAAGA	C M
CD44-i16R	i16	TGCGTGCAGCAAGATGAAACCT	
CD44-C17F		ATGGGAGTCAAGAAGGTGGAGCAA	C M R
CD44-i17R-109		GTTGGGCATTGTGGAGAATGTGCT	C M
CD44-i17F	i17	TGTCTCTGAAGCTCACGCATGTCA	C M
CD44-C18R	C18	CGACTGTTGACTGCAATGCAAACTGC	C M R
CD44-i18F	i18	AGGGAAATGCAGGCCAACCAAA	
CD44-i18R	i18	ATGGGTGGTTGAGGGATGGAATGA	
CD44-C20F	C20	TGATCAACAGTGGCAATGGAGC	C M R

CD44-C20R	C20	TCTGACGACTCCTTGTTCACCA	
CD44-C16F	C16	GTCCCATACCACTCATGGATCTGA	R
CD44-C17R	C17	GGTTGTGTTTGCTCCACCTTCT	R
CD44-C5v2F	junction	TACCACTTTGATGAGCACTAGT	R
CD44-v4v5R	junction	CTGTCTACATCAGTCATCCTTGTG	R
CD44-v10C16R	junction	AATGTGTCTTGGTCTCCTGATAAG	R
