# Supplementary material for: CD44 alternative splicing senses intragenic DNA methylation in tumors via direct and indirect mechanisms
Source: Nucleic Acids Res. 2021 Jun 4;49(11):6213–37. doi: 10.1093/nar/gkab437 (PMC8216461; doi:10.1093/nar/gkab437)
Supplement: gkab437_Supplemental_Files [file gkab437_supplemental_files.zip › Batsche_supplementary figures & tables.doc]

## SUPPLEMENTARY DATA

The full legend of the supplementary figures are in the pdf file of sup. Fig.

### Supplementary Figure S1 :

Double knock-out of DNMT1 and DNMT3b in HCT116 cells promotes epithelial-to-mesenchym transition (EMT).

### Supplementary Figure S2

Meta-analysis of gene expression in HCT116 DKO cells,

### Supplementary Figure S3

Distribution of epigenetic marks covering the CD44 gene in DKO and WT HCT116 cells

### Supplementary Figure S4

DNA methylation effects on alternative splicing in HeLa cells

### Supplementary Figure S5

knocking-down of MBD genes affect the CD44 alternative splicing in HeLa and HCT116 cells

### Supplementary Figure S6.

Analysis of expression pattern of Acute Leukemia (ALL).

### Supplementary Figure S7.

Pathway analysis of genes differentially spliced both in DKO and in ALL but not correlated with variation of local DNA methylation.

### Supplementary Table S1.

Meta-analysis of DKO and WT HCT116 RNA-seq as explained in Supplementary Figure S1D and S1F. Bam files have been realigned on hg19 using STAR, and reads were normalized using Rlog transformation by DESeq2 or log2 transformation. Statistical significance has been proceeded using paired Student’s t-test on Rlog counts or on Log2 counts. The fold change was calculated as the average of the paired differences between DKO vs WT for each normalization. The indicated fold change and p-value were the one for which the gene is predicted to be differentially expressed with the fold change >1.5 and the p-value <0.05. The differentially spliced genes predicted by MAJIQ (Vaquero-Garcia et al., 2016) with high confidence (|dPSI|>0.2)>0.95 are indicated. The corresponding RNA events can be found in Supplementary Table S2A. The genes considered as transcriptional regulator are noted “TF”, the genes considered as RNA-binding protein / splicing regulator are noted “RBP/SF”. The pseudogenes or the parent genes of pseudogenes (Pei et al., 2012) are indicated.

### Supplementary Table S2A

Meta-analysis of RNA-seq data to detect splicing differences between HCT116 and HCT116-DKO cells, using the MAJIQ/Voila package (Vaquero-Garcia et al., 2016), as explained in Supplementary Figure S1F. In order to take into account the statistical dispersion of the data due to the differences in RNA extraction methods, three different comparisons as indicated have been conducted. The comparison of the 4 total RNAs for each cell-types has been conducted using data from (Blattler et al., 2014; Maunakea et al., 2013; Schrijver et al., 2013). The comparison of the 2 poly-adenylated RNAs for each cell-types has been conducted using data from (Schrijver et al., 2013; Simmer et al., 2012). The comparison of all 6 RNA-seq available has also been calculated. The results were presented in the Tables generated by the Voila visualization output.

### Supplementary Table S2B

Differential expression and splicing of RNA-binding protein/splicing factors in DKO cells versus WT HCT116 cells. This Table is a hub of Supplementary Table S1 and Supplementary Table S2A for splicing factors.

### Supplementary Table S3

List of the genes differentially expressed or spliced in HeLa depleted of DNMT1 as described in Supplementary Figure S4F to S4H

### Supplementary Table S4

Analysis of RNA-seq data to detect splicing differences between ALL and normal pre-B cells (HCB), using the MAJIQ/Voila package (Vaquero-Garcia et al., 2016) and datasets selected in the Supplementary figure S6 and in the Figure 7.

### Supplementary Table S5.

Common RNA splicing events in both comparisons : DKO vs WT and ALL vs HCB. Genes are classified in the categories defined in the Figure 8A

### Supplementary Table S6.

List of oligonucleotides used for constructing guide RNA. List of siRNAs. List of PCR primers.

## REFERENCES

Blattler, A., Yao, L., Witt, H., Guo, Y., Nicolet, C.M., Berman, B.P., and Farnham, P.J. (2014). Global loss of DNA methylation uncovers intronic enhancers in genes showing expression changes. Genome Biol. *15*, 469.

Maunakea, A.K., Chepelev, I., Cui, K., and Zhao, K. (2013). Intragenic DNA methylation modulates alternative splicing by recruiting MeCP2 to promote exon recognition. Cell Res. *23*, 1256–1269.

Pei, B., Sisu, C., Frankish, A., Howald, C., Habegger, L., Mu, X.J., Harte, R., Balasubramanian, S., Tanzer, A., and Diekhans, M. (2012). The GENCODE pseudogene resource. Genome Biol. *13*, R51.

Schrijver, J., Trooskens, G., Bosch, L., Dehan, P., Carvalho, P., Van Neste, L., Meijer, G., Baylin, S., and Van Criekinge, W. (2013). Genome-wide total RNA and MBD-sequencing in HCT116 and DKO cells as a global re-expression model [RNA-Seq].

Simmer, F., Brinkman, A.B., Assenov, Y., Matarese, F., Kaan, A., Sabatino, L., Villanueva, A., Huertas, D., Esteller, M., Lengauer, T., et al. (2012). Comparative genome-wide DNA methylation analysis of colorectal tumor and matched normal tissues. Epigenetics *7*, 1355–1367.

Vaquero-Garcia, J., Barrera, A., Gazzara, M.R., Gonzalez-Vallinas, J., Lahens, N.F., Hogenesch, J.B., Lynch, K.W., and Barash, Y. (2016). A new view of transcriptome complexity and regulation through the lens of local splicing variations. Elife *5*, e11752.
